# Hunters or farmers? Microbiome characteristics help elucidate the diet composition in an aquatic carnivorous plant

**DOI:** 10.1186/s40168-018-0600-7

**Published:** 2018-12-17

**Authors:** Dagmara Sirová, Jiří Bárta, Karel Šimek, Thomas Posch, Jiří Pech, James Stone, Jakub Borovec, Lubomír Adamec, Jaroslav Vrba

**Affiliations:** 1 Biology Centre CAS, Institute of Hydrobiology, Na Sádkách 7, CZ-37005 České Budějovice, Czech Republic; 2 Faculty of Science, University of South Bohemia, Branišovská 1760, CZ-37005 České Budějovice, Czech Republic; 3 Limnological Station, Department of Plant and Microbial Biology, University of Zurich, CH-8802 Kilchberg, Switzerland; 4 Department of Biology and Wildlife, University of Alaska Fairbanks, Fairbanks, AK-99775 USA; 5 Institute of Experimental Botany CAS, Rozvojová 263, CZ-16502 Praha 6-Lysolaje, Czech Republic; 6 Institute of Botany CAS, Dukelská 135, CZ-37982 Třeboň, Czech Republic

**Keywords:** *Algae*, *Bacteria*, Ciliate bacterivory, Digestive mutualism, *Fungi*, Herbivory, Nutrient turnover, Plant–microbe interactions, Protists, *Utricularia* traps

## Abstract

**Background:**

*Utricularia* are rootless aquatic carnivorous plants which have recently attracted the attention of researchers due to the peculiarities of their miniaturized genomes. Here, we focus on a novel aspect of *Utricularia* ecophysiology—the interactions with and within the complex communities of microorganisms colonizing their traps and external surfaces.

**Results:**

Bacteria, fungi, algae, and protozoa inhabit the miniature ecosystem of the *Utricularia* trap lumen and are involved in the regeneration of nutrients from complex organic matter. By combining molecular methods, microscopy, and other approaches to assess the trap-associated microbial community structure, diversity, function, as well as the nutrient turn-over potential of bacterivory, we gained insight into the nutrient acquisition strategies of the *Utricularia* hosts.

**Conclusions:**

We conclude that *Utricularia* traps can, in terms of their ecophysiological function, be compared to microbial cultivators or farms, which center around complex microbial consortia acting synergistically to convert complex organic matter, often of algal origin, into a source of utilizable nutrients for the plants.

**Electronic supplementary material:**

The online version of this article (10.1186/s40168-018-0600-7) contains supplementary material, which is available to authorized users.

## Background

Plant-associated microorganisms have long been recognized as key partners in enhancing plant nutrient acquisition, mitigating plant stress, promoting growth, or facilitating successful defense mechanisms against pathogens or grazers [1]. Apart from the well-studied and close symbioses such as mycorrhizal and rhizobial interactions, there is a large pool of diverse microorganisms in varying degrees of association with different plant surfaces and tissues. These often highly complex microbial communities clearly play a significant role in plant ecophysiology, but many of the underlying mechanisms governing these looser associations still remain unexplored [2].

One example of such associations is that between rootless aquatic carnivorous plants from the genus *Utricularia* and the complex microbial communities actively colonizing their traps [3–5] and external leaf surfaces [6]. The exudation of large amounts of bioavailable photosynthates into *Utricularia* traps and their subsequent rapid utilization by the microorganisms present has been experimentally confirmed and represents a direct link between the plant host and associated microbiota [7, 8]. *Utricularia* are among the most numerous and cosmopolitan genera of carnivorous plants, attractive to researchers, due to their extremely small and unusual genomes [9–11]. Depending on the species and growth conditions, a single *Utricularia* plant may bear hundreds to thousands of traps, usually on highly segmented leaves (Additional file 1: Figure S1). These are tiny (1–5 mm long) liquid-filled bladders, whose lumen is completely isolated from the environment by a two-cell thick trap wall [12]. Due to high respiration rates of both plant tissues and microorganisms present, traps become deeply anoxic at night or during intensive organic matter digestion. Short bursts of oxygenated water from the outside transiently improve the oxygen conditions each time the trap fires [13].

*Utricularia* were long thought to be classical examples of the carnivorous habit (Darwin, 1875). However, their nutrient acquisition strategy is the subject of debate and the importance of carnivory in their nutrition has been questioned [14, 15]. During trap lifespan, only a minority of traps capture a macroscopic prey, while all of them contain communities of microbial commensals [14, 16]. It has therefore been proposed that algae, frequently observed and abundant in both the plant periphyton and traps [15, 17], rather than metazoan plankton, are the main source of nutrients for the plants [18, 19]. Over a hundred different species from virtually all major freshwater algal groups were detected inside the traps of *Utricularia* species at a particular location, with large differences among plant species or sampling locations (for review, see [13]). However, only a few of the genera, mainly species capable of osmotrophy (*Euglena* spp., *Phacus* spp., *Scenedesmus* spp.), are able to survive and propagate in the traps. The rest of the algal cells die and decay, representing a potentially abundant source of nutrients for the plant-microbe system. According to previously published research, shoots of aquatic *Utricularia* serve as a substrate for rich periphytic communities and are often colonized significantly more abundantly than other aquatic macrophytes at the same location [16]. Published data suggest that it is the periphyton itself, not the surrounding water, which is the main source of algal cells found inside of the traps [3].

It has been well established in both vertebrates and invertebrates that microbial community composition and diversity in the digestive tract reflects closely the composition of the prevailing food source [20–22]. Looking through this perspective, the traps of *Utricularia* species also function as sophisticated digestive systems, and the associated microbiome should reflect the main nutrient source, which has so far remained ambiguous and its direct verification under in situ conditions technically challenging [23]. Based on the observations of trap-associated microbial community structure and behavior and reports from literature, we hypothesize here that aquatic *Utricularia* traps are structures that are able to successfully digest and utilize organic material of algal (and/or plant) origin. Taking advantage of the knowledge base from studies focusing on the animal digestive system-associated microbiomes, we designed an experimental setup to test this hypothesis by assessing the following:

1. Microbial community structure and diversity. We present the results of the trap microbiome (amplicon and metatranscriptome) sequencing, distinguishing between the inner and periphytic communities associated with two *Utricularia* species—*U. australis* and *U. vulgaris*.
2. The nutrient recycling potential by trap-inhabiting bacterivorous protists as well as their importance for plant growth in a third *Utricularia* species, *U. reflexa*.
3. Functional capabilities of trap-associated microorganisms with respect to gene expression.
4. In addition, we compare the microbial community structure with similar datasets from pitcher traps of other carnivorous plants, gut microflora of various vertebrates and invertebrates, including carnivores and detritivores, the rumen microbiota of various herbivores, and environmental samples from the soil, rhizosphere, and freshwater.

By combining molecular methods, microscopy, and meta-analysis, we were able to shed light onto the ecology of this highly specific microbial niche which represents a unique system in the study of plant–microbe interactions.

## Results and discussion

When considering the overall activity of *U. vulgaris* trap-associated microbiota, inferred from the metatranscriptome analysis, our results confirm highly dynamic communities of both prokaryotes and eukaryotes (Table 1). Expression profiles indicate rapid growth, intensive protein metabolism, respiration, DNA synthesis, and motility. By combining the above metatranscriptomic data with results from amplicon sequencing, we were able to determine whether the taxa present are also participating in the metabolic activities of the microbiome.

**Table 1:**
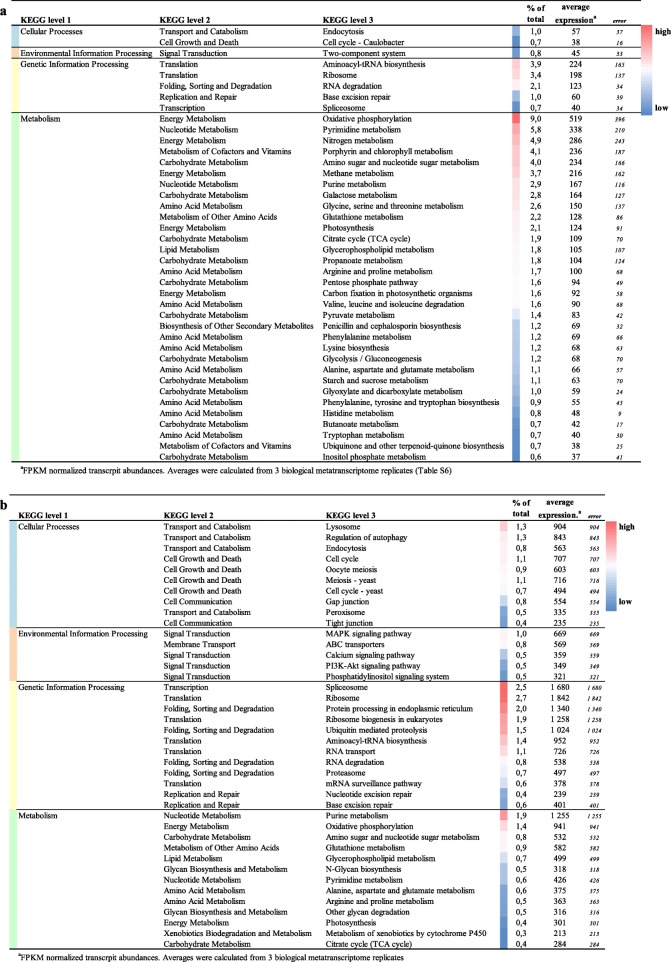
The 40 most expressed prokaryotic (a) and eukaryotic (b) genes found in *U. vulgaris* traps, grouped as functional KEGG modules (level 3). Functional assignments were done in MEGAN 6 using the KEGG database. Abundances were estimated in Trinity by mapping raw reads to assembled contigs using the *bowtie2* algorithm. For complete metatranscriptomic data, please refer to Additional file 3: Table S6 or the website http://utricularia.prf.jcu.cz/

^a^FPKM normalized transcript abundances. Averages were calculated from three biological metatranscriptome replicates (Additional file 3: Table S6)

### Unique trap-associated prokaryotic communities

**Fig. 1:**
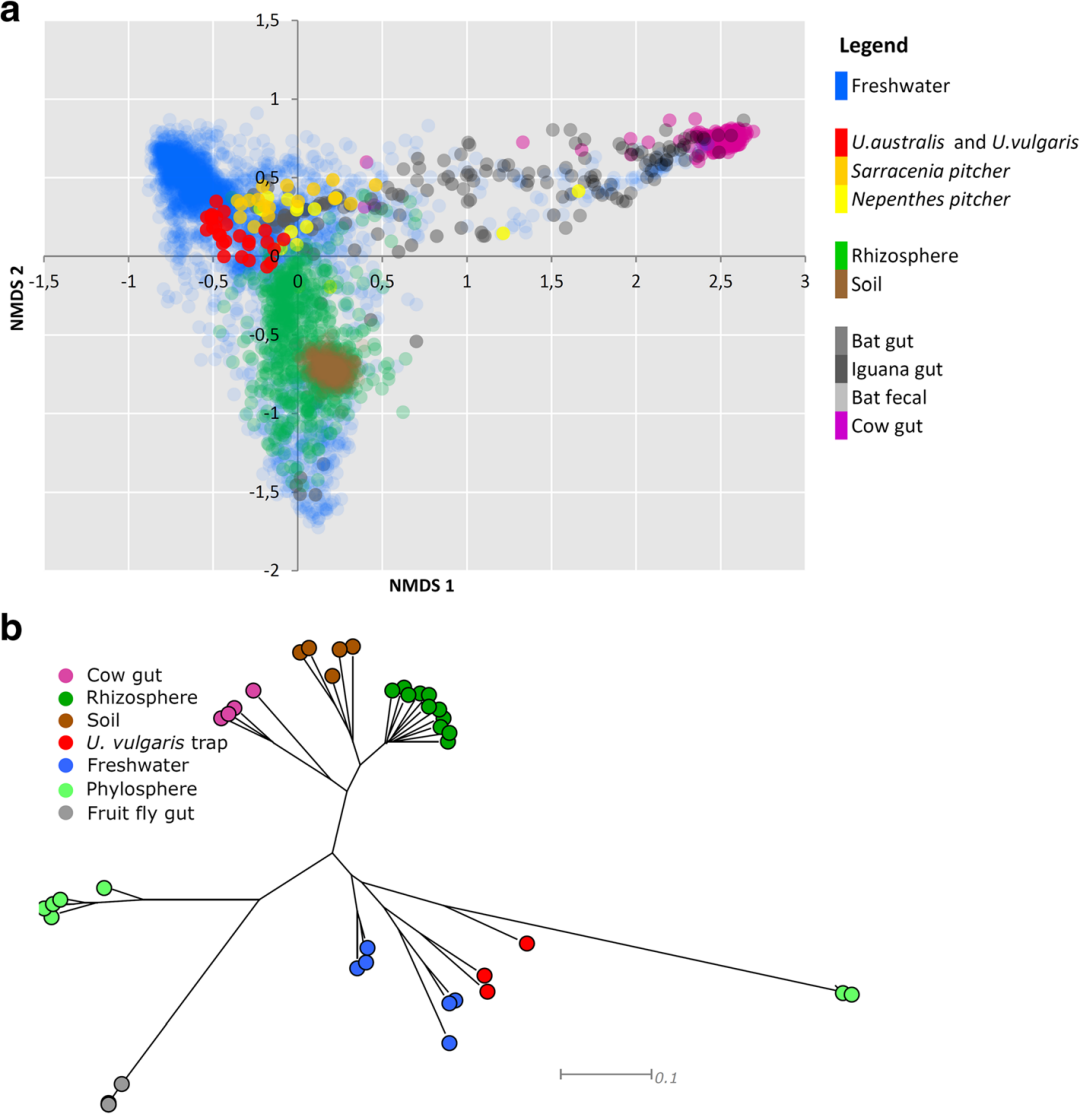
**a** Metanalyses comparing 4221 samples representing ten distinct microbiomes (including both *Utricularia* species). Data (results of 16S rRNA gene sequencing) were downloaded from the Qiita database (https://qiita.ucsd.edu/) and reanalyzed together with *Utricularia* microbiomes. Microbiomes were compared by non-metric multidimensional scaling (NMDS, stress 0.231). Both *U. vulgaris* and *U. australis* microbiomes cluster closely with those of the terrestrial pitcher plants of the genera *Sarracenia* and *Nephentes*. They are positioned between the freshwater and rhizosphere microbiomes, which reflects both the growing environment and the close relationship with their hosts. **b** Comparison of 43 samples representing seven distinct active microbiomes (i.e., transcribed genes), including those associated with *U. vulgaris*. Six metagenomes were obtained from MG-RAST (for details please see the “Methods” section). Neighbor-joining tree was chosen for the visualization. Active microbiome of *U.vulgaris* clustered again closely with the freshwater and phyllosphere microbiomes

Our results show that *Utricularia* plants harbor surprisingly diverse microbial communities: we have identified over 4500 distinct prokaryotic OTUs in the traps and periphyton (see the complete OTU list in Additional file 2: Table S5a). Prokaryotic alpha diversity associated with *U. vulgaris* and *U. australis* was significantly higher than that found associated with other carnivorous plants and, in the case of *U. australis* traps, was comparable to that observed in another high-diversity system—the rhizosphere (Additional file 1: Table S2). A comparison with datasets from different environments (Fig. 1a) revealed that the *U. australis* and *U. vulgaris* trap microbiomes, although species-specific, are highly similar to each other in terms of composition. Out of the different environments selected, *Utricularia* microbiomes most closely resembled microbial communities inhabiting the pitchers of a terrestrial carnivorous plant, *Sarracenia*. Both of these ecosystems are known to harbor microbiota that is able to effectively process complex material of plant origin and thus supply nutrients to the host [24, 25]. When comparing the metatranscriptomic data from the traps of *U. vulgaris* with other systems, the highest (in this case more function-related) similarity was, unsurprisingly, found in the freshwater microbial communities (Fig. 1b).

**Fig. 2:**
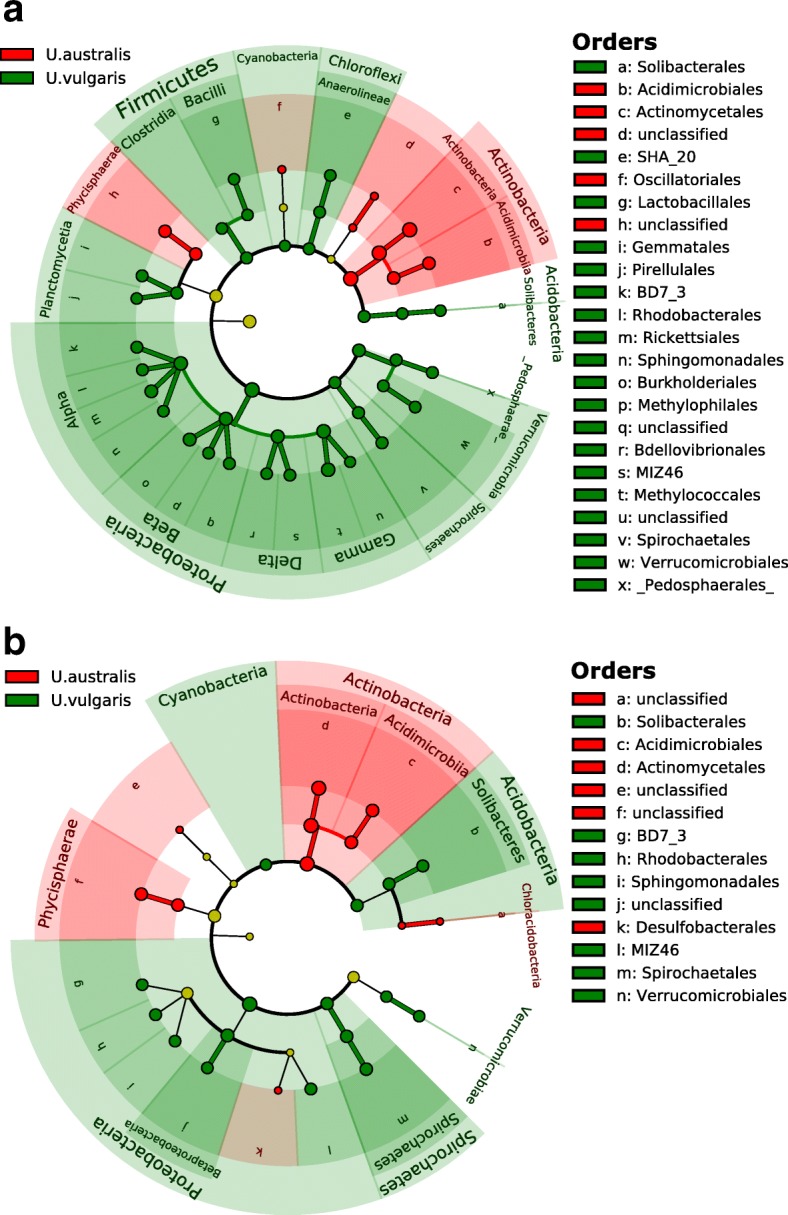
Compositional overlap in *Utricularia*-associated prokaryotic microbiomes **a** Comparison between *U. australis* and *U. vulgaris* trap microbiomes and **b** between the *U. australis* and *U. vulgaris* periphytic communities. The cladograms are the result of the linear discriminant effect size analysis (LEfSe) and show significantly differentially abundant taxa (> 3-fold change of relative OTU abundance) and taxonomy (i.e., going from the central circle in the following order: phylum-class-order). The circle color shows which branch of the phylogenetic tree more significantly represents one of the two studied *Utricularia* hosts (red or green), while circle size corresponds to the relative abundance of the taxon. Yellow color indicates no statistically significant difference between the compared microbiomes

Comparisons of the trap and periphytic communities associated with the same *Utricularia* species revealed that the bacterial communities in both environments were dominated by *Proteobacteria* (58% and 54% of assigned sequences in periphyton and trap, respectively). Over half of the taxa were shared even at the genus level (Additional file 1: Figure S2). *Actinobacteria* were significantly more abundant in the *U. australis* traps and periphyton while *Proteobacteria*, *Acidobacteria*, *Firmicutes*, *Chloroflexi*, *Cyanobacteria*, and *Verrucomicrobia* were significantly enriched in *U. vulgaris* trap and periphyton, respectively (Fig. 2a, b). The close similarity of periphytic and trap communities suggests strong links between *Utricularia* external surfaces and the internal trap environment, with periphyton being the most likely source of inoculum for microbial colonization of traps as well as the primary source of algae as potential nutrients [8, 15]. Looking more specifically at the composition of trap lumen bacterial communities, several taxa stand out. We found the family *Comamonadaceae* to be the most abundant group, constituting > 10% of the total community. Members of this family are strong competitors with flexible metabolism and are considered essential for the digestion of nutritionally poor diet of animal hosts [26, 27]. Another group of *Bacteria*, which was significantly more abundant in the *Utricularia* trap environment, was the family *Peptostreptococcaceae* (order *Clostridiales*, Additional file 2: Table S5a). In traps, this family represented approximately 2% while in periphyton only < 0.001%. The group represents anaerobic fermenters often associated with habitats such as animal guts and oral cavities, manure, soil, and sediments [28]. In the rumen ecosystem, *Peptostreptococcus* spp. produce high amounts of ammonia but are not able to hydrolyze intact proteins and do not use carbohydrates as a carbon source. Thus, they occupy a niche of peptide- and amino-acid-degrading microorganisms and depend on proteolytic *Bacteria* [29]. The genes expressed in the traps that were assigned to this family, such as pyrroline-5-carboxylate reductase [EC:1.5.1.2] or threonine aldolase [EC:4.1.2.5] (Additional file 3: Table S6), are all involved in the metabolism of amino acids. The trap lumen contains very high concentrations of ammonium (2.0–4.3 mg l^−1^ NH_4_-N) [30], and many bacteria that are able to hydrolyze proteins are present (Additional file 3: Table S6); it is therefore likely that a process analogous to that involving *Peptostreptococcaceae* in the rumen is taking place therein.

We have also detected the family *Bacteriovoracaceae* in the lumen, whose members are known for being obligatory predators of other, especially Gram-negative, bacteria. There have been few studies of the ecological roles of predatory bacteria. They are, however, present in diverse habitats, which indicates that, like viruses, they are important determinants of microbial community dynamics and functioning [31]. In the *Utricularia* traps, *Bacteriovoraceae* together with *Myxococcales*, another potentially predatory bacterial group, represent almost 5% (Additional file 2: Table S5a); both groups are metabolically active (Additional file 3: Table S6) and, therefore, likely to selectively influence the trap microbial community dynamics through enhanced mortality rates of particular bacterial species in this isolated environment [32].

Deeper analyses of our sequencing data revealed other interesting microbial functional guilds in the traps. These included the cellulolytic species capable of degrading complex organic material of plant (algal) origin (Additional file 2: Table S5a), for example, *Clostridium*, *Ruminococcus*, *Caldicellulosiruptor*, *Butyrivibrio*, *Acetivibrio*, *Cellulomonas*, *Erwinia*, *Thermobifida*, *Fibrobacter*, *Cytophaga*, and *Sporocytophaga*. Also notable is the significant presence of active myxobacteria (*Cystobacter* spp.), which are known to include cellulolytic species and are frequently isolated from systems with high decomposing plant material content [33]. Overall the cellulolytic bacteria represented approx. 10% of the total bacterial community (Table 3, Additional file 2: Table S5a). The transcriptomic analysis offers several clues indicating the ability of these microbes to degrade complex organic material of algal origin. Algal cell walls and other cellular structures are composed of various monosaccharides, derived from glucose, linked into polymers (cellulose and hemicellulose). These monosaccharides also include D-galactose [34]. The α- and β-galactosidases catalyze the cleavage of the terminal D-galactosyl residues of plant and algal hemicelluloses and their activity is often associated with herbivore digestive systems [35, 36]. These were among the most expressed prokaryotic genes in Utricularia traps (Additional file 4: Table S7), together with UDP-glucose 4-epimerase, which performs the final step in the Leloir pathway catalyzing the reversible conversion of UDP-galactose to UDP-glucose. The high expression levels of these enzymes underscore the importance of microbial galactose metabolism in the traps and are a further indication that the trap microbes, especially bacteria, actively degrade complex algal/plant material. Other enzymes, such as those belonging to the families of glycoside hydrolases, cellulases, peroxidases, and xylanases, were also found to be expressed in the trap lumen (Additional file 4: Table S7).

Looking at *Utricularia*-associated microbiome structure, despite of the high similarity between the trap lumen and the periphyton in terms of prokaryotic community composition, when we compare the microbial co-occurrence analyses, we see two strikingly different systems with distinct potential “keystone” taxa (Additional file 1: Table S4) and a distinct degree of interconnectedness (Additional file 1: Figures S3 and S4) in each of the two environments. While the periphytic communities show a co-occurrence pattern typically observed in highly spatially and functionally interconnected microbial biofilms (Additional file 1: Figure S3), the co-occurrence pattern of the trap community consists of several smaller, mutually disconnected microbial networks and implies a much more heterogeneous and fragmented environment inside the trap lumen (Additional file 1: Figure S4). This result is consistent with previously published observations showing progressing degree of microbial aggregation into flocks and multispecies biofilms with progressing *Utricularia* trap development [15]. Further support is provided by the metatranscriptomic data. The high expression of bacterial UDP-glucose 6-dehydrogenase, which has been linked to the environmentally regulated biosynthesis of exopolysaccharides [37], or that of transaldolase, which is also one of the highly expressed proteins in the trap fluid and has been shown to be an important colonization factor favoring the establishment of bacteria in the gut [38], provides further support for bacterial aggregation and attachment to organic particles in the trap lumen. This activity is typical for the gut environment of herbivores and suggests that metabolically related organisms in the *Utricularia* trap lumen associate with their preferred substrates and produce the myriad of enzymes necessary for the digestion of chemically and structurally complex particles, hence creating a system of mutually disconnected micro-environments.

### Traps as methane sources?

Herbivore gut ecosystems generally tend to produce copious amounts of methane as a result of the anaerobic respiration activity by the strictly anaerobic methanogenic *Archaea* [39]. Using gas chromatography, we have not detected the release of methane gas from the *Utricularia* traps (data not shown), and methanogens were not detected in our trap fluid samples using the qPCR assay (Additional file 1: Table S3). However, significant amounts of diverse methanotrophs were found in the traps, constituting up to 40% of the total prokaryotic community (Additional file 1: Table S3, Additional file 2: Table S5b. These included active obligate methanotrophs, for example, from the genus *Methylococcus* (*Gammaproteobacteria*, Additional file 2: Table S5). Moreover, methane metabolism was also found to be one of the most expressed prokaryotic modules (KEGG) in the trap fluid metatranscriptome from *U. vulgaris* (Table 1). This raises a question of where the methanotrophs acquire methane since there are no active methanogens. These somewhat paradoxical results may be explained by a recent discovery [40] that the degradation of mainly polysaccharides and their derivates in the aquatic environment by commonly occurring bacteria, e.g., *Pseudomonas* spp., can, even in the presence of oxygen, result in the release of methane, ethylene, and propylene.

We speculate that this process can explain the presence and activity of methanotrophs in the *Utricularia* traps, which may metabolize all of the produced methane, thus preventing its detection. This hypothesis, however, needs to be experimentally verified.

### Trap-associated eukaryotic communities

Compared to the prokaryotes, the eukaryotic communities were relatively poor in diversity. The most diverse and abundant group were the *Algae*, whose species composition and richness was described in detail elsewhere [18, 24]. However, we also found the *Fungi* to be present in the traps as a significant proportion of the total microbial community, as quantified by qPCR (Additional file 1: Table S3). Many fungal taxa, whose presence inside of the *Utricularia* traps was determined by SSU rRNA sequencing (e.g., *Chrysomphalina* sp., *Agaricales*, *Basidiomycota*, Additional file 2: Table S5c), were most probably entrapped as spores from the ambient environment and do not represent trap-associated microbes as such, but rather a potential source of nutrients. Others, most notably the saprotrophic *Basidiobolus* sp. (*Basidiobolales*, *Zygomycota*) abundant in the traps of all ages (up to 45% of total OTUs), or species belonging to the *Chytridiomycota* (Additional file 2: Table S5c), found to be actively growing in the traps (Additional file 3: Table S6), are likely a component of the trap microbial network and contribute to the nutrient release and assimilation by the plant host.

Protists are another group of eukaryotes living inside of traps. Although low in diversity, they are key players in the trap microbial food webs. Their numbers in the studied *U. reflexa* traps are immense, rising steadily with increasing trap age, up to approximately 50,000 cells of ciliates alone per millilitre of trap fluid in the oldest traps. This means that a single trap, depending on age, harbored tens to hundreds of individuals (Table 2). Such high population densities are unheard of in natural environments and are comparable only to those found in the mammalian rumen or in the activated sludge systems [41, 42]. The protist community consisted of various euglenid species and a monoculture of a conspicuous zoochlorellae-bearing ciliate (Additional file 1: Figure S5). This organism has recently been described as a new species—*Tetrahymena utriculariae—*and has not yet been found in any other environment [43, 44]. Ciliates are important bacterial predators, mainly in nutrient-rich freshwater environments [45–47], and are generally considered one of the key agents ensuring nutrient recycling and transfer to other trophic levels (e.g., [48]). We have confirmed and quantified bacterivory in *T. utriculariae*, and found the grazing rates to be comparable to literature reports [49]. Due to their abundance, the ciliates were able to turn over the entire bacterial standing stock in the trap fluid extremely fast: four to five times in 24 h in the younger traps (Table 3). Turnover time increased markedly in the old traps, due to the large increase in bacterial numbers. Obviously, the turnover of microbial mass in the *U. reflexa* traps and the release of soluble mineral nutrients from microbial cells with their subsequent uptake by the plant from the trap fluid are, to a large extent, facilitated by protist predation on bacteria. Although *T. utriculariae* has only been found in the *U. reflexa* species, other species of bacterivorous protozoa in high numbers have been observed in the traps of all aquatic *Utricularia* traps studied by us so far. It can therefore be concluded that *Utricularia* plants apparently depend on microbial activity for the supply of nutrients and thus the amount of bacterial mass produced and turned over in the traps likely is as important to the plant host as the amount of organic matter digested.

**Table 2:** The estimates of bacterial and ciliate numbers, the individual grazing rate (IGR) and total grazing rate (TGR) of ciliates and the turnover rate of bacterial standing stock (turnover) in *U. reflexa* traps of different age is presented. Means of three technical replicates are shown for IGR

Trap age	Bacteria [10^6^ ml^−1^]	Ciliates [10^3^ ml^−1^]	IGR [bact. prot.^− 1^ h^− 1^]	TGR [10^6^ bact. ml^−1^ d^− 1^]	Turnover [day^−1^]
Young	44.5	35.4	273.4	223.1	5.0
Mature	65.3	46.5	263.1	290.0	4.4
Old	266.9	50.8	342.1	411.8	1.5

**Table 3:** Relative proportions (%) of selected ecologically relevant prokaryotic functional guilds in different *U. vulgaris* trap ages (young, mature, and old)

Functional guild	Young	Mature	Old	All
	%			
Cellulolytic bacteria*	11	10	9	10
Methanotrophs^‡^	15	15	14	15
Polyphenol degraders^‡^	25	28	29	27
Nitrate reducers^‡^	27	30	28	28
Denitrifiers^‡^	16	17	17	16
N_2_ fixators^‡^	14	17	18	16
DNRA (*nrfA* gene)	7	8	6	7
Urea decomposers (*ureA* gene)	29	34	35	33
Sulfate reducers^‡^	2	2	2	2

Differences between trap ages were not statistically significant for all functional guilds (ANOVA, Tukey’s post hoc test, alpha 0.05, *n* = 3)

*Cellulolytic bacteria were determined based on known cellulolytic families [50]. Functional genes were annotated based on RDP Fungene database ver. 8.3; DNRA—dissimilatory nitrate reduction to ammonium. For the functional annotation of all OTUs, please refer to Additional file 5: Table S8

^‡^Averages of relative gene abundance were calculated for those functional groups where more than one gene was responsible for the metabolic pathway; methanotrophs (*pmoA* and *mmoX*), polyphenol degraders (*laccase_Asco*, *laccase_Basidio*, *lip*, *ppo*), nitrate reducers (*narG*, *napA*), denitrifiers (*nirS*, *nirK*, *norB*, *nosZ*), N_2_ fixators (*nifH*, *nifD*), and sulfate reducers (*dsrA*, *dsrB*)

## Conclusions

**Fig. 3:**
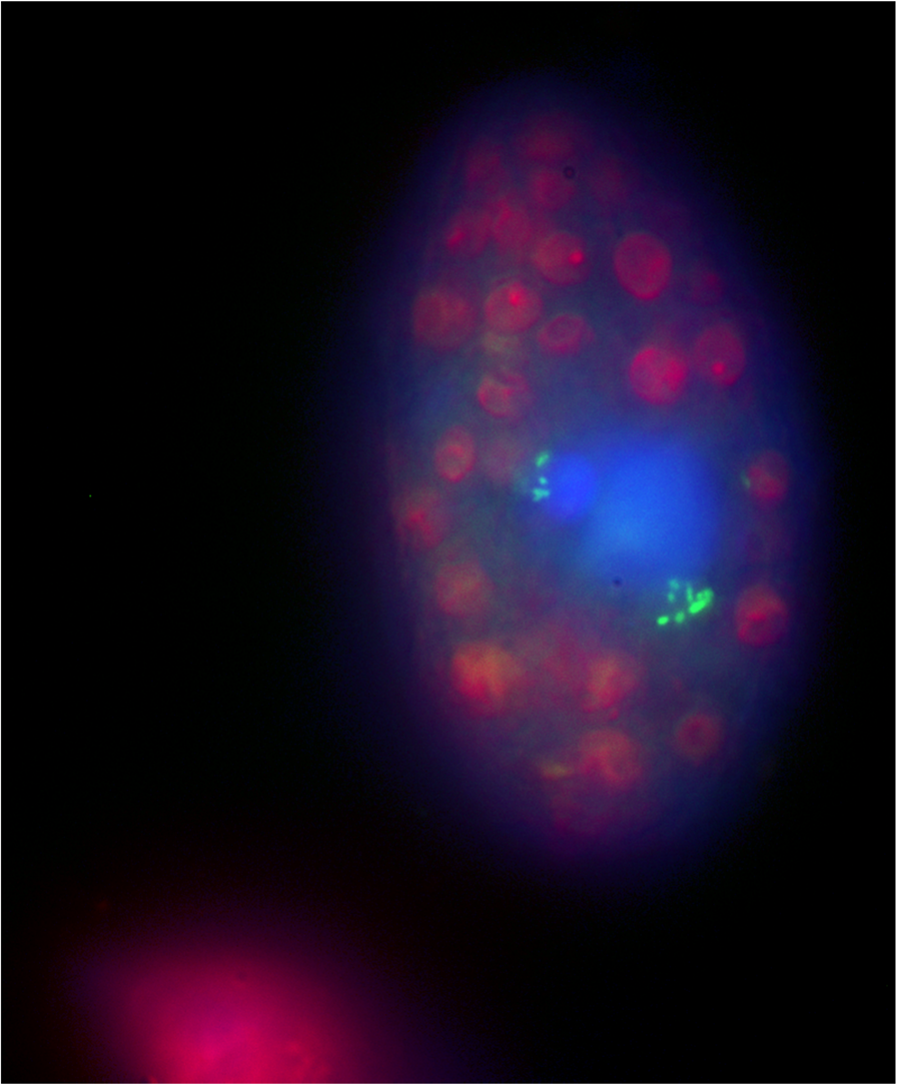
Conceptual representation of the *Utricularia* trap ecophysiology: main microbe–microbe and plant–microbe interactions are shown

We conclude that the aquatic *Utricularia* plants support the development of diverse and sophisticated microbial ecosystems, both in their traps and on their external surfaces, which allows them to cultivate, “harvest,” digest, and utilize complex organic material, often of algal origin, and thus thrive even at highly oligotrophic sites. Here, although metazoan prey is usually scarce and prey capture rates correspondingly low [15], the supply of algae growing in close proximity to the traps as part of the *Utricularia* periphytic “gardens” is continuous and abundant [17]. Youngest traps, which start their development as sterile, receive copious amounts of photosynthates (up to 25% of plant primary production [7]) that stimulate the rapid growth of microorganisms in the lumen. In analogy to the digestive tracts of animals, feedstuffs enter the mature traps and are degraded to various extents by the microbial populations colonizing them. Like the gut/ruminal ecosystems, traps harbor a symbiotic population of bacteria and fungi that have adapted for survival under low oxygen supply, high cell densities, and predation by protozoa. Moreover, they have evolved the capacity for efficient utilization of complex and often recalcitrant plant polymers, such as cellulose and hemicellulose. The protozoan predation is a key process by which essential nutrients are regenerated from microbial biomass and made available for plant uptake (Fig. 3). Omnivory, rather than carnivory alone, appears to be the most probable mode of nutrition in *Utricularia*, at least for aquatic species. The short (few weeks) species-specific trap life cycle has a profound influence on the development of the associated microbial community, which develops through periods of organic matter accumulation, rapid decomposition, mineral nutrient absorption by both the host and the microorganisms, which may result in competition [13], and, finally, through the decoupling of the plant–microbe interaction in senescent traps. The *Utricularia*–microbe systems thus represent unique biodiversity and activity hotspots within the nutrient-poor, dystrophic environments, in which they grow, and should be considered as synchronized, mutually dependent biological, ecological, and perhaps even evolutionary units in future research.

## Methods

### Experimental design, plant material, and sampling

To assess the presence and diversity of trap associated microbiota, the ecophysiologically well characterized aquatic species *U. vulgaris* and *U. australis* were selected. Adult *U. australis* plants (40–50 cm in length) were collected from a mesotrophic bog at Ruda fishpond (South Bohemia, see [7] for details). The 0.8 m^2^ polypropylene experimental container, where *U. vulgaris* plants were cultivated, contained *Carex* sp. litter and approximately 250 l of dystrophic water, closely simulating natural conditions [51, 52]. For the assessment of microbial community structure, *U. vulgaris* and *U. australis* plants were divided into three sections of increasing age (young, mature, old). From each of these segments, approximately 70 larger, excised, functional traps without visible metazoan prey and trapless leaves with periphyton were collected (approximately 200 mg fresh weight).

To assess the actively transcribed microbial gene pool, excised *U. vulgaris* traps from the entire shoot without visible metazoan prey were collected randomly (approximately 250 mg fresh weight), with pooled traps from a single plant considered a replicate; three biological replicates were collected in total. All collected plant material was immediately placed into liquid N_2_ and samples were stored at −80 °C until further processing.

For the estimation of the protozoan grazing rates, a different species with larger traps—the tropical *U. reflexa*—was selected, because relatively large volumes of trap fluid are needed for this analysis. The plants were cultivated indoors in 3-l aquaria, in dystrophic cultivation water closely simulating natural conditions (see [12]).

### DNA extraction from *Utricularia* trap fluid and the taxonomical evaluation of the *Utricularia*-associated microorganisms

Nucleic acid extractions were conducted according to a modified bead-beating protocol [53]. Approximately 500 μl of pooled trap fluid were extracted for each sample. Total DNA was quantified fluorometrically using SybrGreen and StepOne (Applied Biosystems, USA) instrument in “fluorescence reading mode” [54]. The PCR primers (515F/806R) targeted the V4 region of the SSU rRNA, previously shown to yield accurate phylogenetic information and to have only a few biases against any bacterial taxa [55–57]. Each sample was amplified in triplicate, combined, and quantified using Invitrogen PicoGreen and a plate reader, and equal amounts of DNA from each amplicon were pooled into a single 1.5-ml microcentrifuge tube. Once pooled, amplicons were cleaned up using the UltraClean PCR Clean-up kit (MO BIO Laboratories). Amplicons were sequenced on the Illumina MiSeq platform at the Institute of Genomics and Systems Biology, Argonne National Laboratory, Argonne (Chicago, USA). Paired-end reads were joined using Perl scripts yielding approximately 253 bp amplicons. Approximately 1.8 million paired-end reads were obtained with average 66.000 reads per sample. Quality filtering of reads and chimera check (UCHIME algorithm in de novo mode) was applied as described previously [58]. Reads were assigned to operational taxonomic units (OTUs, cutoff 97% sequence identity) using an open-reference OTU picking protocol with QIIME implemented scripts [58]. Reads were taxonomically assigned using Green Genes database, release 13.08 as reference. Those reads which were assigned as “chloroplast” and “mitochondrion” were excluded from further analyses. For the estimation of unique microbial taxa in *U. australis* and *U. vulgaris* traps and periphyton, the OTUs were grouped at the genus level. Genera presented only in *U. australis* or *U. vulgaris* and respective trap or periphyton samples were classified as unique for each microbiome. Differences between the various prokaryotic communities were tested with PERMANOVA and the nonparametric method adonis in QIIME 1.9.0.

### Comparative meta-analyses of prokaryotic communities from different habitats

To compare the composition of *Utricularia* trap-associated microbial communities, data (OTU tables in biom format) from nine relevant studies representing different habitats were analyzed. Seven of the studies were obtained from Qiita (https://qiita.ucsd.edu/) database (Additional file 1: Table S1). The remaining two studies were obtained from NCBI Genebank: the 16S rRNA sequences of the *Nepenthes* pitcher microbiome from the SRA archive (project ID PRJNA225539) and *Sarracenia* pitcher sequences from the Genebank database (accession numbers JF745346–JF745532 and JN368236–JN368422) (Additional file 1: Table S1). Altogether, 4221 samples were included in the meta-analysis. The Qiita database works with the closed-reference OTU picking algorithm; we have therefore processed our sequences and also the sequences from *Nepenthes* and *Sarracenia* pitcher microbiomes in the same way as the Qiita pipeline, to ensure comparability with Qiita OTU tables. All OTU tables were then merged together using Qiime scripts and analyzed as one dataset. Non-metric multidimensional scaling (NMDS) using Bray-Curtis dissimilatory metrics was used for computing distances between samples (Fig. 1a). LDA Effect Size (LEfSe) based on the relative abundances of the microbial taxa was calculated to identify the corresponding taxa with higher abundance in *U.australis* and *U.vulgaris* samples [59]. Analysis of LEfSe was performed according to the instructions on the website (http://huttenhower.sph.harvard.edu/galaxy).To obtain a more function-related point of view, three *Utricularia vulgaris* metatranscriptomes were compared to 43 metagenomes and/or metatranscriptomes available from six different habitats (Fig. 1b). The sequences from these studies were obtained from the MG-RAST server (Additional file 1: Table S1).

### RNA-seq analysis for functional profiling of the *U. vulgaris* trap-associated microbiome

To assess the actively transcribed microbial gene pool, total RNA was extracted from the *U. vulgaris* trap samples (*n* = 3), using the protocol identical to that described in detail previously [30]. Briefly, DNA was removed from the extracts and two transcriptomic libraries, eukaryotic and prokaryotic, were created at the Institute of Genomics and Systems Biology, Argonne National Laboratory, Argonne (Chicago, USA) using standard Illumina TruSeq RNA library preparation kits. The ribosomal RNA as well as eukaryotic (plant) RNA fraction was removed in order to enrich prokaryotic transcripts, and, vice versa, eukaryotic transcript enrichment was performed in parallel, to capture transcripts from fungi, protists, and other eukaryotic microorganisms. Enriched mRNA from both libraries was reverse transcribed to create metatranscriptomic libraries and sequenced using Illumina HiSeq platform (100 × 100 cycle paired-end run). We obtained approximately 40 million paired-end reads per sample. Reads were quality checked; low quality reads and reads with ambiguous bases were filtered out. Reads from all three replicates (approx. 120 million sequences) were then assembled with Velvet Optimizer [60] which resulted in approximately 500,000 contigs. In this step, we filtered out the potential *Utricularia* transcripts by blasting contigs against our *Utricularia* reference transcriptome [61]. All contigs which gave significant hit (*e* value < 0.0001, min score 80) were excluded from further analyses and considered as *Utricularia* transcripts. Contigs were also blasted against the SILVA database (release 111) to identify ribosomal RNAs (rRNAs). Those sequences that gave BLAST bit score greater than 80 were marked as rRNAs and extracted from the dataset. Reads were then mapped back onto the remaining contigs using Trinity package (bowtie algorithm with default parameters [62] with FPKM gene transcript abundance normalization). To identify potential prokaryotic functional gene transcripts, the remaining contigs without rRNAs were blasted against the nr database with *e* value of 0.001 using diamond algorithm [63]. Annotation was done in MEGAN 6 software [64]. Genes below hit score 50 were manually excluded from the analyses (Additional file 4: Table S7).

The lists of bacterial and archaeal species for each ecologically relevant functional gene was downloaded from the FunGene database [65] from which a local database was created (Additional file 5: Table S8). The OTUs which were taxonomically annotated to genus level were scanned against this database for the presence of the specific functional genes (Additional file 5: Table S8).

### Microbial network analyses in *U. vulgaris* and *U. australis* traps and periphyton

The relative abundances of OTUs were square-root transformed [66] (Additional file 6). To avoid spurious correlations caused by the presence of rare OTUs, we chose only those OTUs which were found in at least five out of nine traps and five out of eight *U.vulgaris* periphyton samples, and their sum of abundance was at least 20 and 16 sequences out of 1000, respectively. The resulting OTU tables, separately for the trap (15 samples) and the periphyton (16 samples), were used for microbial network analyses [67]. We performed the recommended calculations (*n*_eff_, sparsity) regarding the composition of prokaryotic filtered OTU tables. Based on the sparsity of filtered OTU tables, we chose the CoNet network algorithm as the relevant calculation method [68, 69]. The parameters and settings for network analyses in the CoNET application were as follow: -parent_child_exclusion, -row_minocc 5, -correlations (Spearman, Pearson, mutual information, Bray-Curtis and Kullback-Leibler dissimilatory). The threshold for edge selection was set to 1000 top and bottom. During randomization, 100 iterations were calculated for edge scores. In the following bootstrap step, 100 iterations were calculated, and unstable edges were filtered out (p-level threshold of 0.05). The Brown method was chosen as the *P* value merging method, and the Benjamini–Hochberg procedure was selected for multiple test correction. The resulting network was visualized and analyzed (i.e., degree of nodes, betweenness centrality, closeness centrality) in Cytoscape 3.0.2. Potential keystone OTUs were identified [66].

### Sequence deposition

Raw sequences of 16S rRNA, ITS1 amplicons, and raw sequences of all three metatranscriptomes were deposited in European Nucleotide Archive (ENA) under study ID PRJEB25993. Annotated metatranscriptomic sequences were deposited at the following website: http://utricularia.prf.jcu.cz/

### Quantification of trap-associated bacterial, fungal, methanogenic, and methanotrophic communities in *U. vulgaris* and *U. australis*

For quantification of bacterial, fungal, methanogenic and methanotrophic communities, the quantitative PCR (qPCR) of targeted 16S rRNA, 18S rRNA, mcrA, and pmoA gene was used, respectively. For detail description, please see Additional files 1, 2, 3, 4, 5, and 6.

### Bacterial and protozoan enumeration and the estimation of protozoan grazing rates in *U. reflexa*

Ten *U. reflexa* plants were divided into three segments of increasing age (young, mature, old). Each segment contained six leaf whorls. Trap fluid was collected from the traps in each segment (see [3]), and a pooled sample (~ 750 μl for each age category) from all ten plants was made. Bacterial and protozoan counts in the trap fluid samples were estimated using epifluorescence microscopy, according to methods described previously [45].

The protist grazing rates were estimated using fluorescently labeled bacteria (FLB) as a tracer. The FLB were prepared from the strain *Limnohabitans planktonicus*, as detailed in [44]. Cell sizes of the strain are comparable to that of bacterial cells commonly occurring within the *U. reflexa* traps. The FLB uptake rates were determined in short-term triplicate experiments, where tracer FLB were added to the trap-fluid samples to constitute 6–8% of the total bacterial concentration. For further details on sample fixation, protist staining and enumeration, and tracer ingestion determinations, see [44]. At least 45 ciliates were inspected for FLB ingestion in each replicate sample. To estimate total protist grazing, we multiplied average uptake rates of protozoa by their in situ abundances as previously described [45].

## Additional files

Additional file 1: **Table S1.** Source and identification of studies used for comparative meta-analyses in Fig. 1a, b and Additional file 1 Figure S2. **Table S2.** Comparison of selected alpha diversity indexes for various 16S datasets from different habitats (*N* = number of samples in study). All datasets were subsampled to 2000 sequences prior to analyses, more information on the data used can be found in Additional file 1: **Table S1.** **Table S3.** Comparison of the abundance of total bacteria, methanotrophs, methanogens, fungi, and fungal to bacterial ratio (F/B) in the trap and periphyton of *Utricularia* species (data are averages from *U. australis* and *U. vulgaris* samples). Quantification of bacteria, fungi, methanotrophs and methanogens was done using the 16SrDNA, 18SrDNA, pmoA, and mcrA gene copy numbers, respectively. Quantity was normalized to total amount of DNA. **Table S4.** The 5 most important potential keystone taxa in the *Utricularia*-associated microbiomes, based on network analyses. **Figure S1.** Experimental *Utricularia vulgaris* shoot on a Petri dish. Segmented leaves bearing traps and the growth tip are visible. **Figure S2.** Compositional overlap in *Utricularia*-associated prokaryotic microbiomes at the genus level. (a) Comparison between *U. australis* and *U. vulgaris* microbiomes and (b) between the *U. australis* and *U. vulgaris* periphyton and trap environments. **Figure S3.** Co-occurrence network for the prokaryotic community in the periphyton of *Utricularia vulgaris,* constructed from QIIME 16S data. **Figure S4.** Co-occurrence network for the prokaryotic community in the trap fluid of *Utricularia vulgaris*, constructed from QIIME 16S data*.* **Figure S5.** *Tetrahymena utriculariae* under the epifluorescence microscope. Zoochlorellae are visible in purple, the nucleus is stained blue, and fluorescently labeled bacteria in food vacuoles show green fluorescence. (DOCX 5108 kb)

Additional file 2: **Table S5.** a Prokaryotic OTUs distribution in samples. b Methanotropic OTUs distribution in samples (external file). c Fungal OTUs distribution in samples. (XLSX 930 kb)

Additional file 3: **Table S6.** Summary of metatranscriptomic analyses. (XLSX 1461 kb)

Additional file 4: **Table S7.** Summary of selected protein families (Pfam) based on rpstblastx algorithm. (PDF 410 kb)

Additional file 5: **Table S8.** Functional annotation of OTUs (only for those with genus assigment) genes. Annotation was based on RDP Fungene database ver. 8.3. (PDF 673 kb)

Additional file 6: **Table S9.** Results of the adonis analysis for *U. australis* and U.vulgaris prokaryotic community based on 16S rDNA gene sequencing. (PDF 459 kb)
