# Correction to: Hunters or farmers? Microbiome characteristics help elucidate the diet composition in an aquatic carnivorous plant

**DOI:** 10.1186/s40168-018-0617-y

**Published:** 2019-01-05

**Authors:** Dagmara Sirová, Jiří Bárta, Karel Šimek, Thomas Posch, Jiří Pech, James Stone, Jakub Borovec, Lubomír Adamec, Jaroslav Vrba

**Affiliations:** 1 Biology Centre CAS, Institute of Hydrobiology, Na Sádkách 7, CZ-37005 České Budějovice, Czech Republic; 2 Faculty of Science, University of South Bohemia, Branišovská 1760, CZ-37005 České Budějovice, Czech Republic; 3 Limnological Station, Department of Plant and Microbial Biology, University of Zurich, CH-8802 Kilchberg, Switzerland; 4 Department of Biology and Wildlife, University of Alaska Fairbanks, Fairbanks, AK-99775 USA; 5 Institute of Experimental Botany CAS, Rozvojová 263, CZ-16502 Praha 6-Lysolaje, Czech Republic; 6 Institute of Botany CAS, Dukelská 135, CZ-37982 Třeboň, Czech Republic

**Correction to: Microbiome (2018) 6:225**

**https://doi.org/10.1186/s40168-018-0600-7**

Following publication of the original article [1], the author reported an error in Fig. 3. The correct Fig. 3 is shown here.

**Fig. 3:**
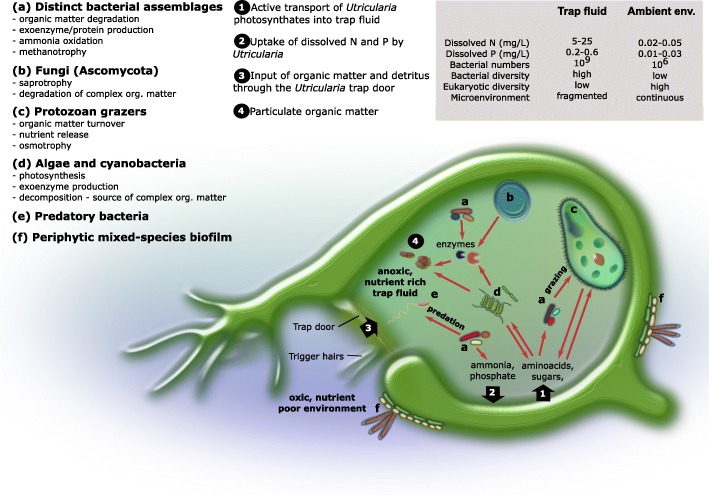
Conceptual representation of the *Utricularia* trap ecophysiology: main microbe–microbe and plant–microbe interactions are shown

The publishers apologise for this error. The original article [1] has been updated.
