# Supplementary material for: Hunters or farmers? Microbiome characteristics help elucidate the diet composition in an aquatic carnivorous plant
Source: Microbiome. 2018 Dec 17;6:225. doi: 10.1186/s40168-018-0600-7 (PMC6297986; doi:10.1186/s40168-018-0600-7)
Supplement: Supplementary file 1 — Table S1. Source and identification of studies used for comparative meta-analyses in Fig. 1a, b and Additional file 1 Figure S2. Table S2. Comparison of selected alpha diversity indexes for various 16S datasets from different habitats (N = number of samples in study). All datasets were subsampled to 2000 sequences prior to analyses, more information on the data used can be found in Additional file 1: Table S1. Table S3. Comparison of the abundance of total bacteria, methanotrophs, methanogens, fungi, and fungal to bacterial ratio (F/B) in the trap and periphyton of Utricularia species (data are averages from U. australis and U. vulgaris samples). Quantification of bacteria, fungi, methanotrophs and methanogens was done using the 16SrDNA, 18SrDNA, pmoA, and mcrA gene copy numbers, respectively. Quantity was normalized to total amount of DNA. Table S4. The 5 most important potential keystone taxa in the Utricularia-associated microbiomes, based on network analyses. Figure S1. Experimental Utricularia vulgaris shoot on a Petri dish. Segmented leaves bearing traps and the growth tip are visible. Figure S2. Compositional overlap in Utricularia-associated prokaryotic microbiomes at the genus level. (a) Comparison between U. australis and U. vulgaris microbiomes and (b) between the U. australis and U. vulgaris periphyton and trap environments. Figure S3. Co-occurrence network for the prokaryotic community in the periphyton of Utricularia vulgaris, constructed from QIIME 16S data. Figure S4. Co-occurrence network for the prokaryotic community in the trap fluid of Utricularia vulgaris, constructed from QIIME 16S data. Figure S5. Tetrahymena utriculariae under the epifluorescence microscope. Zoochlorellae are visible in purple, the nucleus is stained blue, and fluorescently labeled bacteria in food vacuoles show green fluorescence. (DOCX 5108 kb) [file 40168_2018_600_MOESM1_ESM.docx]

Additional file for:

“Hunters or farmers? Microbiome characteristics help elucidate the diet composition in an aquatic carnivorous plant.”

Dagmara Sirová٭, Jiří Bárta, Karel Šimek, Thomas Posch, Jiří Pech, James Stone, Jakub Borovec, Lubomír Adamec, and Jaroslav Vrba

* Correspondence: dagmara_sirova@hotmail.com

**Supplementary material and methods**

Quantification of bacterial and fungal SSU rRNA genes from *U. australis* (n = 12) and *U. vulgaris* (n = 12) was performed using the FastStart SybrGREEN Roche^®^ Supermix and Step One system (Life Technologies, USA). Each reaction mixture (20 µl) contained 2 µl DNA template (~1–2 ng DNA), 1 µl each primer (0.5 pmol µl^−1^ each, final concentration), 6 µl dH_2_O, 10 µl FastStart SybrGREEN Roche^®^ Supermix (Roche, France) and 1 µl BSA (Fermentas, 20 µg µl^−1^). The qPCR conditions for bacterial quantification were as follows: initial denaturation (3 min, 95°C) was followed by 30 cycles of 30 s at 95°C, 30 s at 62°C, 15 s at 72°C, and completed by fluorescence data acquisition at 80°C used for target quantification. Product specificity was confirmed by melting point analysis (52°C to 95°C with a temperature increase of 0.3°C per second and fluorescence reading every 15s) and amplicon size was verified with agarose gel electrophoresis.

Bacterial DNA standards consisted of a dilution series (ranging from 10^1^ to 10^9^ gene copies µl^−1^) of purified PCR product obtained from genomic *Escherichia coli* *ATCC 9637* DNA by using the SSU gene-specific primers 341F/534R [1], which were the same as for real samples. The R^2^ values for the standard curves were >0.99. The amplification efficiency was 92.8 ± 0.5%. The qPCR conditions for fungal quantification were as follows: initial denaturation (10 min, 95°C) followed by 40 cycles of 1 min at 95°C, 1 min at 56°C, 1 min at 72°C, and completed by fluorescence data acquisition at 72°C used for target quantification. Fungal DNA standards consisted of a dilution series (ranging from 10^1^ to 10^7^ gene copies µl^−1^) of purified PCR product obtained from genomic *Aspergillus niger* DNA by using the SSU gene-specific primers nu-SSU-0817-5’ and nu-SSU1196-3’ [2], which were the same as for real samples. R^2^ values for the fungal standard curves were > 0.99. The amplification efficiency was 94.3 ± 1.1%.

Detection limits for the various assays (i.e. lowest standard concentration that is significantly different from the non-template controls) were less than 100 gene copies for each of the genes per assay. Samples, standards, and non-template controls were run in duplicates. To deal with potential inhibition during PCR, the enhancers (BSA, DMSO) were added to the PCR mixture. Also several dilutions (10×, 20×, 50×, 100×, and 1000×) for each sample were tested to see the dilution effect on Ct values. Also artificial DNA was spiked into the PCR reaction mixture in order to find the most suitable dillution with minimum impact of PCR inhibitors.

*Quantification of trap-associated methanogenic and methanotrophic communities*

Quantification of the *mcrA* gene (methanogens) was performed using the FastStart SybrGREEN Roche^®^ Supermix and Step One system (Life Technologies, USA). Each reaction mixture (20 µl) contained 2 µl DNA template (~1–2 ng DNA), 0.1 µl each primer (0.3 pmol.µl^-1^ each, final concentration), 6 µl dH_2_O, 10 µl FastStart SybrGREEN Roche^®^ Supermix (Roche, France) and 0.4 µl BSA (Fermentas, 20 µg.µl^-1^). Primers ME1 (5‘-GCM ATG CAR ATH GGW ATG TC-3‘) a MCR1R (5‘-ARC CAD ATY TGR TCR TA-3‘) producing amplicon length of 280bp [3]. The qPCR conditions for *mcrA* gene were as follows: initial denaturation (10 min, 95°C) followed by 35 cycles of 30s at 95°C, 1 min at 60°C, 1 min at 72°C, and completed by fluorescence data acquisition at 72°C used for target quantification. Standards consisted of a dilution series (ranging from 10^1^ to 10^7^ gene copies µl^−1^) of purified PCR product obtained from genomic DNA of *Methanosarcina barkeri* DSM-800, with the same primers as for real samples*.*

Quantification of *pmoA* gene (methanotrophs) was performed using the FastStart SybrGREEN Roche® Supermix and Step One system (Life Technologies, USA). Each reaction mixture (20 µl) contained 2 µl DNA template (~1–2 ng DNA), 0.24 µl each primer (0.5 pmol µl^−1^ each, final concentration), 5.6 µl dH_2_O, 10 µl FastStart SybrGREEN Roche® Supermix (Roche, France), 0.5 µl DMSO and 0.4 µl BSA (Fermentas, 20 µg µl^−1^). Primers A189-F (5‘-GGNGACTGGGACTTCTGG-3‘) a Mb661-R (5‘-GGTAARGACGTTGCNCCGG-3‘) producing amplicon length of 491bp [4]. The qPCR conditions for *pmoA* gene were as follows: initial denaturation (10 min, 95°C) followed by 35 cycles of 30 s at 95°C, 20 s at 57°C, 45 s at 72°C, and completed by fluorescence data acquisition at 72°C used for target quantification. Standards consisted of a dilution series (ranging from 10^1^ to 10^7^ gene copies µl^−1^) of purified PCR product obtained from genomic DNA of *Methylobacter luteus,* with the same primers as for real samples.

**Table captions:**

Table S1: Source and identification of studies used for comparative metananalyses in Figure 1a,b and Figure S2

Table S2: Comparison of selected alpha diversity indexes for various 16S datasets from different habitats (N = number of samples in study). All datasets were subsampled to 2000 sequences prior to analyses, more information on the data used can be found in Supplementary Table 1.

Table S3: Comparison of the abundance of total bacteria, methanothrophs, methanogens, fungi, and fungal to bacterial ratio (F/B) in the trap and periphyton of *Utricularia* species (data are averages from *U. australis* and *U. vulgaris* samples). Quantification of bacteria, fungi, methanotrophs and methanogens was done using the 16SrDNA, 18SrDNA, pmoA, and mcrA gene copy numbers, respectively. Quantity was normalized to total amount of DNA.

Table S4: The 5 most important potential keystone taxa in the *Utricularia*-associated microbiomes, based on network analyses.

Table S5a: Prokaryotic OTUs distribution in samples (external file)

Table S5b: Methanotropic OTUs distribution in samples (external file)

Table S5c: Fungal OTUs distribution in samples (external file)

Table S6: Summary of metatranscriptomic analyses (external file)

Table S7: Summary of selected protein families (Pfam) based on rpstblastx algorithm (external file)

Table S8: Functional annotation of OTUs (only for those with genus assigment) genes. Annotation was based on RDP Fungene database ver. 8.3 (external file)

Table S9: Results of the adonis analysis for U. australis and U.vulgaris prokaryotic community based on 16S rDNA gene sequencing (external file)

Table S1: Source and identification of studies used for comparative metananalyses in Figure 1a,b and Figure S2

| **Qiita study ID** | **Study topic** | **Number of samples** | **Reference** |
| --- | --- | --- | --- |
|  |  |  |  |
| 963 | Intergenerational lizard lounges do not explain variation in the gut microbiomes of green iguanas | 100 | NA |
| 945 | Long-term seasonal development in selected lakes of Northeast Germany | 1147 | NA |
| 809 | Prokaryote populations of extant microbialites along a depth gradient in Pavilion Lake, British Columbia, Canada | 21 | NA |
| 1792 | Diversity and heritability of the maize rhizosphere microbiome under field conditions | 464 | [5] |
| 1734 | Gut microbiota of phyllostomid bats that span a breadth of diets | 96 | NA |
| 1642 | Microbial community of the bulk soil and rhizosphere of rice plants over its lifecycle | 646 | NA |
| 1621 | Time series to determine the effect of Monensin on microbial hindgut bacterial composition | 192 | NA |
| 1288 | Bacterial community spatial and temporal variation in a north temperate bog Lake | 1506 | NA |
| 1041 | Great Lake microbiome | 49 | NA |
|  |  |  |  |
| **SRA ID** |  |  |  |
| PRJNA225539 | *Nepenthes* fluid microbial diversity and composition | 16 | [6] |
|  |  |  |  |
| **GenBank ID** |  |  |  |
| JF745346–JF745532 and JN368236–JN368422 | Bacterial diversity in three distinct sub-habitats within the pitchers of the northern pitcher plant, *Sarracenia purpurea* | 372 | [7] |
| **MG-RAST ID** | **Study topic** | **Number of samples** | **Reference** |
| mgm4441205.3, mgm4447811.3, mgm4447810.3, mgm4449956.3, mgm4450328.3 | Rhizosphere (metagenome) | 5 | NA |
| mgm4597886.3, mgm4597885.3, mgm4597884.3, mgm4597883.3, mgm4597881.3, mgm4597882.3 | Freshwater (metatranscriptome) | 6 | NA |
| mgm4493655.3, mgm4493654.3, mgm4493653.3, mgm4493652.3, mgm4493651.3, mgm4493650.3, mgm4493649.3, mgm4493549.3, mgm4493548.3, mgm4493547.3, mgm4493546.3, mgm4493545.3, mgm4493544.3 | Soil (metagenome) | 13 | NA |
| mgm4441679.3, mgm4441680.3, mgm4441681.3, mgm4441682.3, | Cow rumen (metagenome) | 4 | NA |
| mgm4527734.3, mgm4628078.3, mgm4628073.3, mgm4627695.3, mgm4627680.3, mgm4627584.3, mgm4627651.3 | Phylosphere (metatranscriptome) | 7 | NA |
| mgm4503983.3, mgm4503982.3, mgm4503981.3, mgm4503980.3, mgm4503979.3, mgm4503978.3, mgm4503977.3, mgm4503976.3 | Fruit fly (metatranscriptome) | 8 | NA |

Table S2: Comparison of selected alpha diversity indexes for various 16S datasets from different habitats (N = number of samples in study). All datasets were subsampled to 2000 sequences prior to analyses, more information on the data used can be found in Supplementary Table 1.

| **Habitat** | ***N*** | **PD whole tree** | **ANOVA** | **Chao1 index** | **ANOVA** | **Obs. sp.** | **ANOVA** | **Shannon index** | **ANOVA** |
| --- | --- | --- | --- | --- | --- | --- | --- | --- | --- |
| Soil | 309 | 74.2 | a | 2238.5 | a | 1043.2 | a | 9.6 | a |
| Rhizosphere | 751 | 49.6 | b | 1505.3 | b | 675.5 | b | 7.3 | b |
| Cow gut | 188 | 35.0 | c | 778.5 | c | 480.1 | c | 7.6 | b |
| *U. vulgaris* (trap) | 8 | 31.5 | c | 545.7 | c | 345.0 | c | 6.9 | b |
| *U. vulgaris* (periphyton) | 4 | 33.1 | c | 651.1 | c | 373.3 | c | 6.9 | b |
| *U. australis* (trap) | 8 | 38.4 | c | 1172.7 | c | 533.5 | c | 7.3 | b |
| *U. australis* (periphyton) | 3 | 31.8 | c | 782.6 | c | 394.3 | c | 7.0 | b |
| Iguana gut | 91 | 22.6 | b | 359.3 | d | 218.3 | d | 5.4 | c |
| Freshwater | 2635 | 21.1 | b | 262.9 | d | 206.9 | d | 5.4 | c |
| *Sarracenia* pitchers | 17 | 15.4 | bd | 270.1 | d | 169.4 | de | 5.3 | c |
| *Nepenthes* pitchers | 16 | 13.1 | bd | 211.2 | d | 132.8 | de | 4.5 | c |
| Bat gut | 51 | 11.9 | d | 185.4 | d | 108.4 | e | 3.0 | de |
| Bat fecal | 13 | 10.3 | bd | 163.4 | d | 87.8 | de | 2.6 | e |

Table S3: Comparison of the abundance of total bacteria, methanothrophs, methanogens, fungi, and fungal to bacterial ratio (F/B) in the trap and periphyton of *Utricularia* species (data are averages from *U. australis* and *U. vulgaris* samples). Quantification of bacteria, fungi, methanotrophs and methanogens was done using the 16SrDNA, 18SrDNA, pmoA, and mcrA gene copy numbers, respectively. Quantity was normalized to total amount of DNA.

|  |  |  |  |  |  |
| --- | --- | --- | --- | --- | --- |
|  | **Bacteria** (10^6^) | **Fungi** (10^3^) | **Methanotrophs**  (10^6^) | **Methanotrophs/Bacteria** | **Methanogens** |
| **Trap** (n =16) | 1.6 | 7.7 | 0.34 | 0.22 | B.D. |
| *St.dev* | *1.9* | *5.3* | *0.4* | *0.20* |  |
| **Periphyton** (n = 6) | 2.9 | 41 | 0.41 | 0.13 | B.D. |
| *St.dev* | *4.3* | *79* | *0.21* | *0.05* |  |

B.D. – below detection

Table S4: The 5 most important potential keystone taxa in the *Utricularia*-associated microbiomes, based on network analyses.

| ***Utricularia* trap** | | | | |
| --- | --- | --- | --- | --- |
| **Phylum** | **Order** | **Degree** | **Betweenness Centrality** | **Closeness Centrality** |
| *Actinobacteria* | *Actinomycetales* | 10 | 0.423 | 0.474 |
| *Proteobacteria* | *Rhodospirillales* | 9 | 0.112 | 0.100 |
| *Proteobacteria* | *Rhodospirillales* | 9 | 0.175 | 0.128 |
| *Bacteroidetes* | *Cytophagales* | 9 | 0.330 | 0.140 |
| *Verrucomicrobia* | *[Pedosphaerales]* | 9 | 0.075 | 0.136 |
|  |  |  |  |  |
| ***Utricularia* periphyton** | | | | |
| **Phylum** | **Order** | **Degree** | **Betweenness Centrality** | **Closeness Centrality** |
| *Uncultured bacterium** | *N.A.* | 72 | 0.408 | 0.349 |
| *Proteobacteria* | *Pseudomonadales* | 30 | 0.190 | 0.278 |
| *Actinobacteria* | *Actinomycetales* | 29 | 0.029 | 0.298 |
| *Bacteroidetes* | *Sphingobacteriales* | 28 | 0.027 | 0.291 |
| *Proteobacteria* | *Rhizobiales* | 28 | 0.158 | 0.324 |

*Uncultured bacterium clone CF69, rape phyllosphere

**Figure captions**

Figure S1: Experimental *Utricularia vulgaris* shoot on a Petri dish. Segmented leaves bearing traps and the growth tip are visible.

Figure S2: Compositional overlap in *Utricularia*-associated prokaryotic microbiomes at the genus level. (a) Comparison between *U. australis* and *U. vulgaris* microbiomes and (b) between the *U. australis* and *U. vulgaris* periphyton and trap environments.

Figure S3: Co-occurrence network for the prokaryotic community in the **periphyton** of *Utricularia vulgaris,* constructed from QIIME 16S data.

Figure S4: Co-occurrence network for the prokaryotic community in the **trap** fluid of *Utricularia vulgaris*, constructed from QIIME 16S data*.*

Figure S5: *Tetrahymena utriculariae* under the epifluorescence microscope**.** Zoochlorellae are visible in purple, the nucleus is stained blue, and fluorescently labeled bacteria in food vacuoles show green fluorescence.


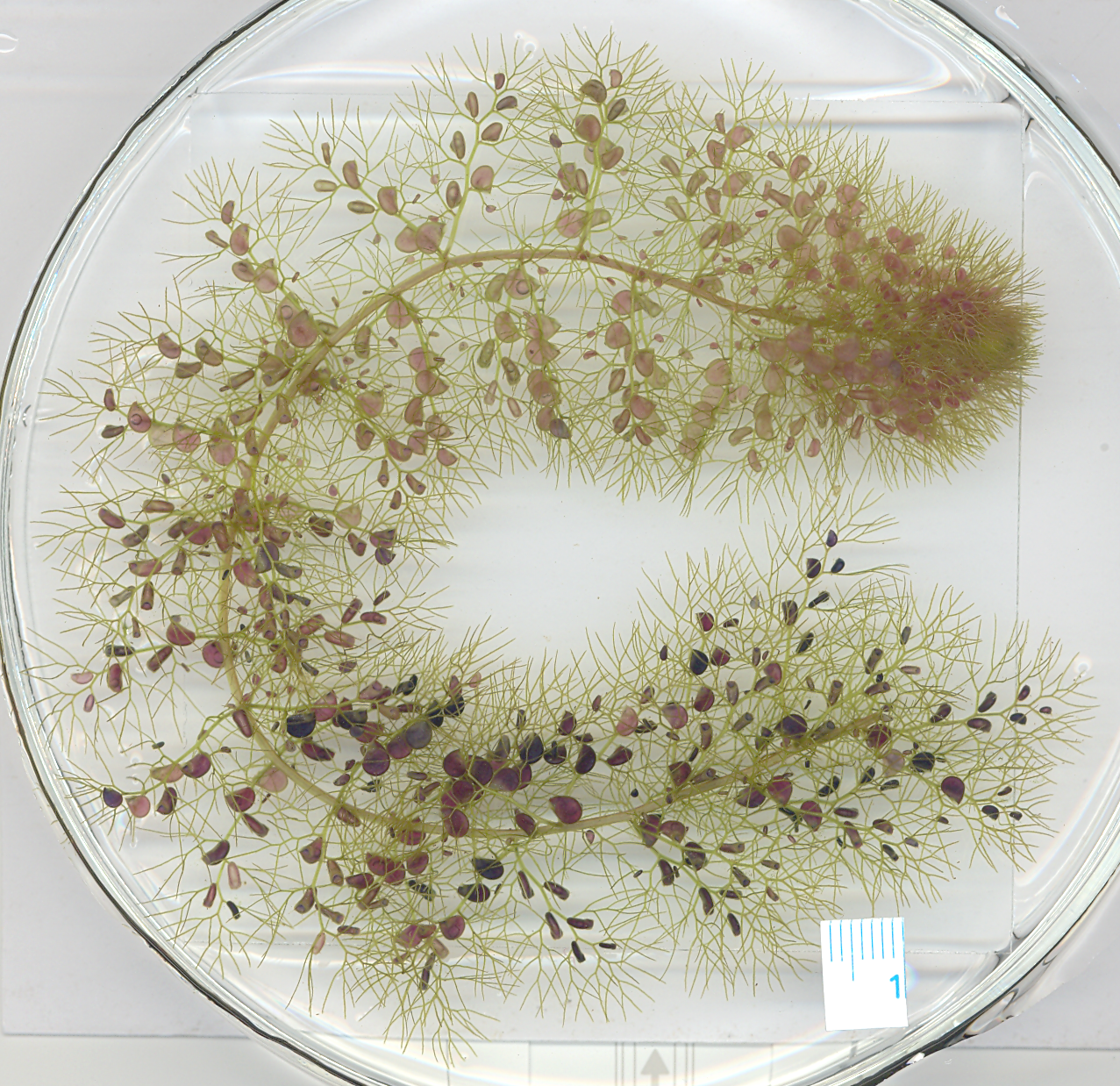
Figure S1

Figure S2


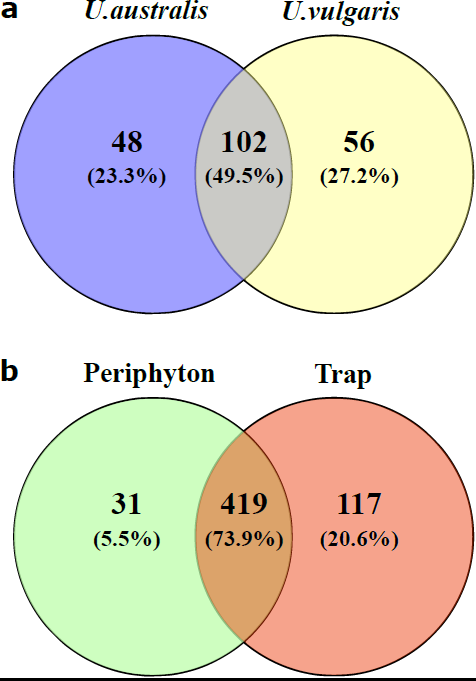


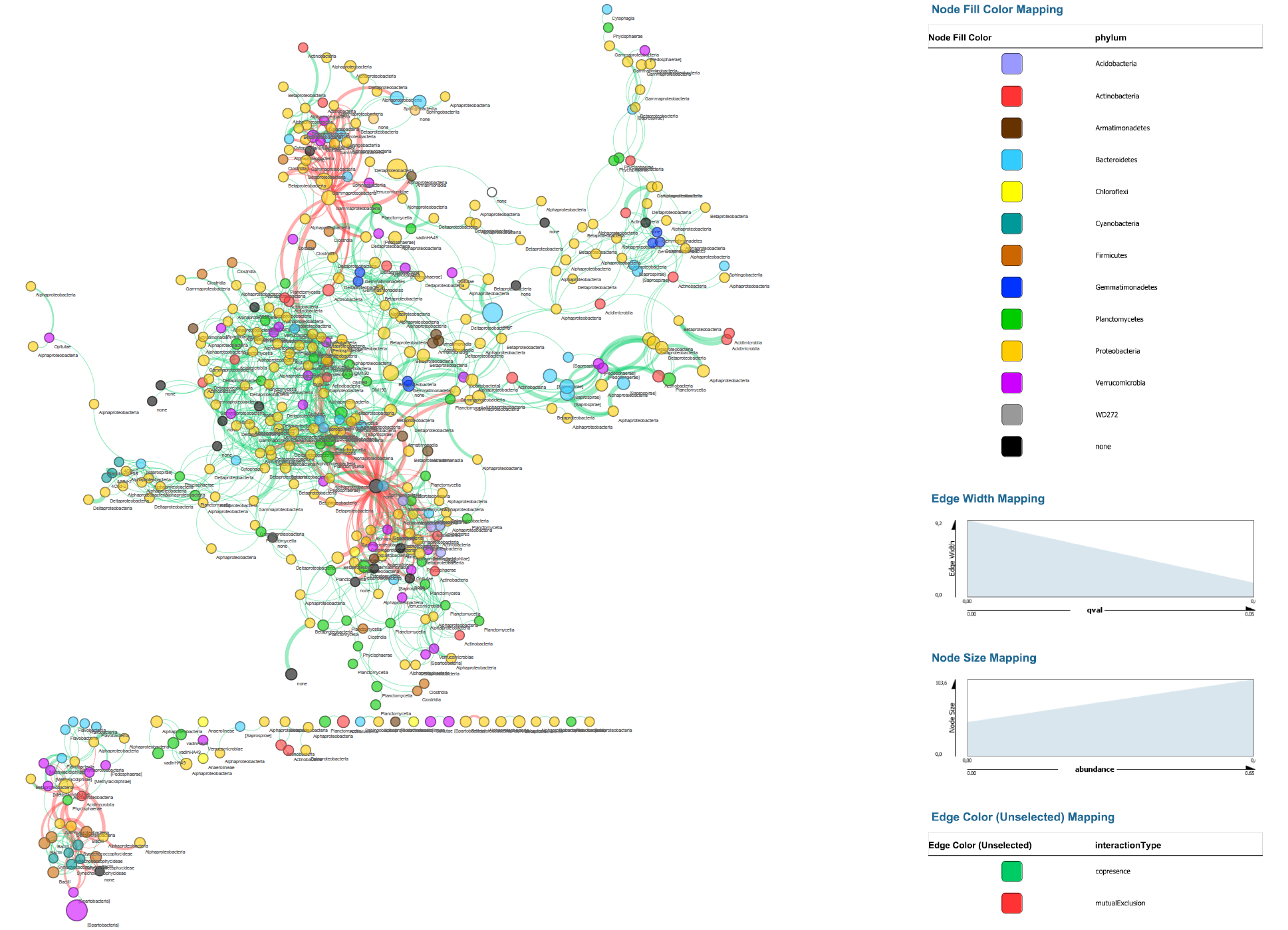
Figure S3

Figure S4
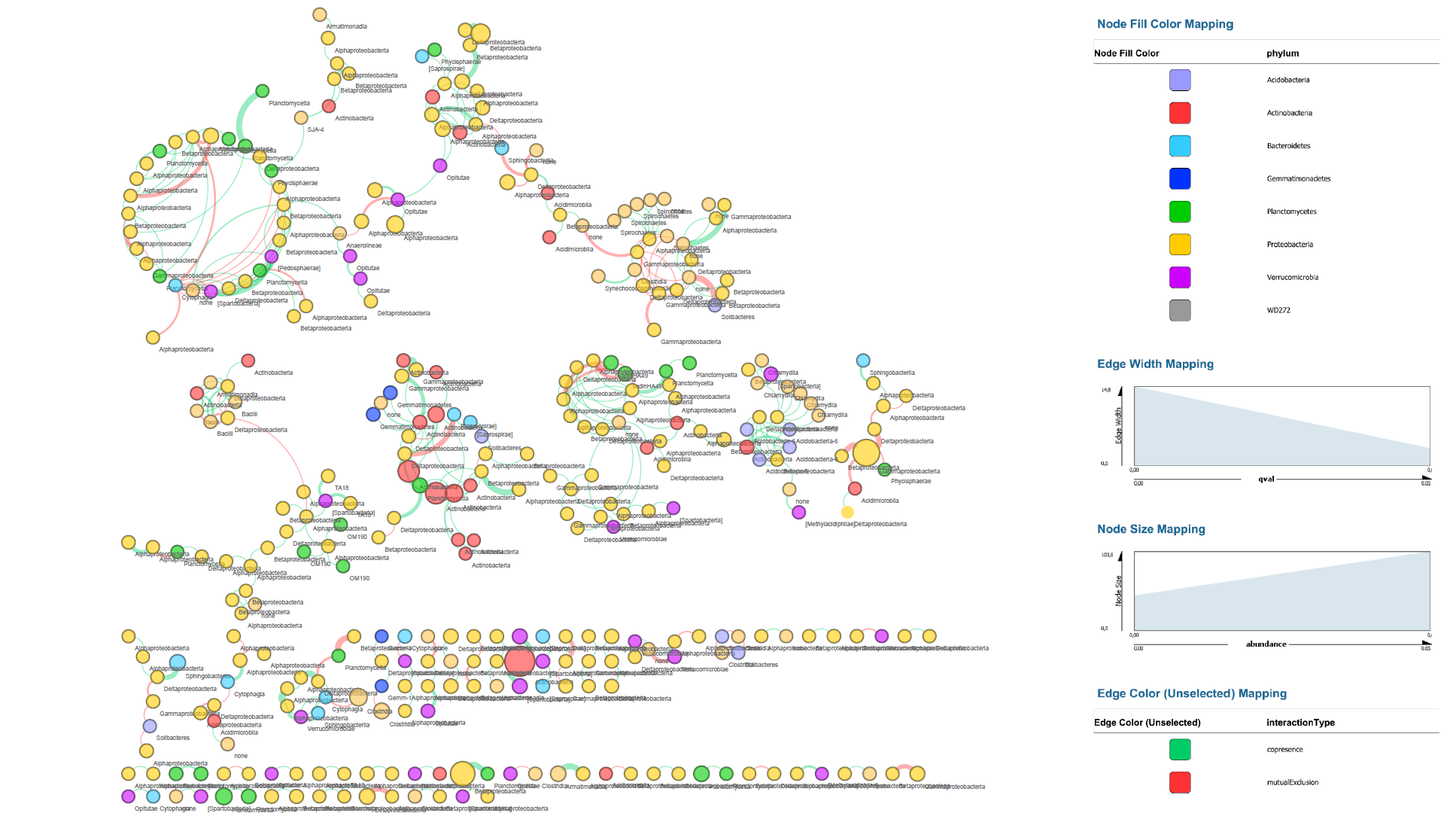


**Figure S5**

**
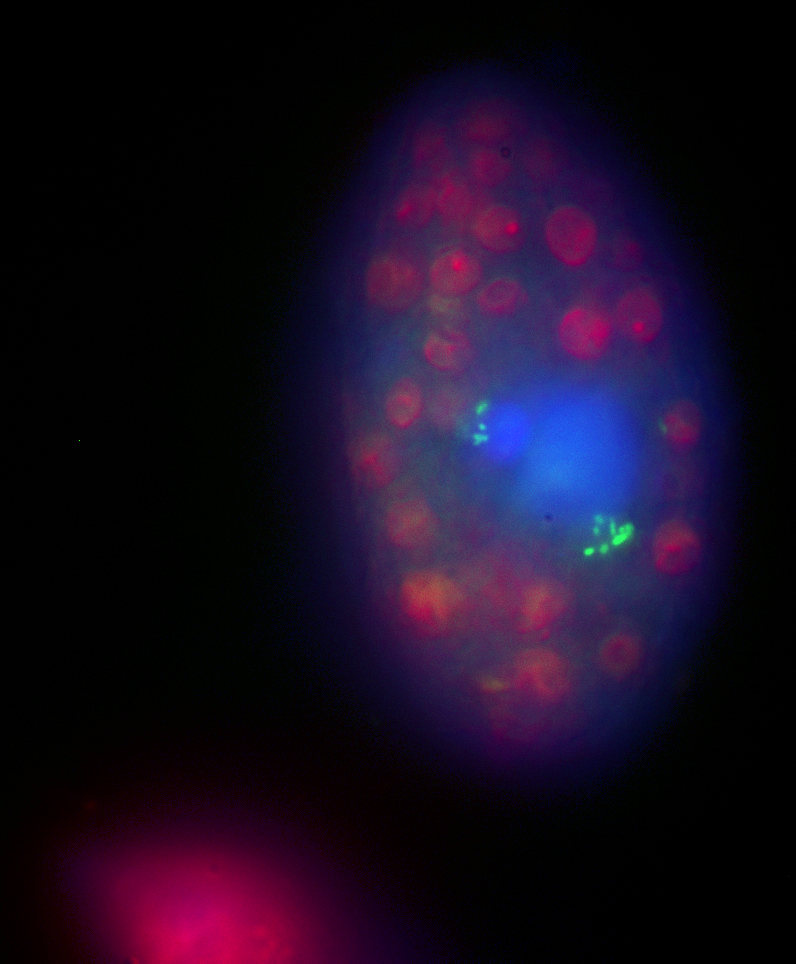
**

**Supplementary references:**

1. Muyzer, G., E.C. Dewaal, and A.G. Uitterlinden, *Profiling of Complex Microbial-Populations by Denaturing Gradient Gel-Electrophoresis Analysis of Polymerase Chain Reaction-Amplified Genes-Coding for 16s Ribosomal-Rna.* Applied and Environmental Microbiology, 1993. **59**(3): p. 695-700.

2. Borneman, J. and R.J. Hartin, *PCR primers that amplify fungal rRNA genes from environmental samples.* Applied and Environmental Microbiology, 2000. **66**(10): p. 4356-4360.

3. Hales, B.A., et al., *Isolation and identification of methanogen-specific DNA from blanket bog feat by PCR amplification and sequence analysis.* Applied and Environmental Microbiology, 1996. **62**(2): p. 668-675.

4. Kolb, S., et al., *Quantitative detection of methanotrophs in soil by novel pmoA-targeted real-time PCR assays.* Applied and Environmental Microbiology, 2003. **69**(5): p. 2423-2429.

5. Peiffer, J.A., et al., *Diversity and heritability of the maize rhizosphere microbiome under field conditions.* Proceedings of the National Academy of Sciences of the United States of America, 2013. **110**(16): p. 6548-6553.

6. Takeuchi, Y., et al., *Bacterial diversity and composition in the fluid of pitcher plants of the genus Nepenthes.* Systematic and Applied Microbiology, 2015. **38**(5): p. 330-339.

7. Krieger, J.R. and P.S. Kourtev, *Bacterial diversity in three distinct sub-habitats within the pitchers of the northern pitcher plant, Sarracenia purpurea.* Fems Microbiology Ecology, 2012. **79**(3): p. 555-567.
