# Supplementary material for: Hunters or farmers? Microbiome characteristics help elucidate the diet composition in an aquatic carnivorous plant
Source: Microbiome. 2018 Dec 17;6:225. doi: 10.1186/s40168-018-0600-7 (PMC6297986; doi:10.1186/s40168-018-0600-7)
Supplement: Supplementary file 4 — Table S7. Summary of selected protein families (Pfam) based on rpstblastx algorithm. (PDF 410 kb) [file 40168_2018_600_MOESM4_ESM.pdf]

Table S7: Summary of selected protein families (Pfam) based on rpstblastx algorithm

Contig ID	Pfam ID	Short Descrip	AK7	AK8	AK9	Average
Contig_572332	pfam07477	Glyco_hydro_67C,	206,9	317,8	244,5	256,4
Contig_357571	pfam03662	Glyco_hydro_79n,	156,5	151,5	157,9	155,3
Contig_574695	pfam00332	Glyco_hydro_17,	126,0	71,4	239,5	145,6
Contig_535137	pfam00295	Glyco_hydro_28,	100,4	127,1	171,4	133,0
Contig_576640	pfam00840	Glyco_hydro_7,	95,0	111,8	191,0	132,6
Contig_521848	pfam13199	Glyco_hydro_66,	83,9	80,5	134,1	99,5
Contig_553302	pfam08307	Glyco_hydro_98C,	78,8	93,6	83,4	85,3
Contig_574594	pfam08307	Glyco_hydro_98C,	70,0	63,4	115,8	83,1
Contig_541205	pfam00728	Glyco_hydro_20,	47,8	83,9	111,3	81,0
Contig_494797	pfam01532	Glyco_hydro_47,	51,5	50,7	140,5	80,9
Contig_571849	pfam03639	Glyco_hydro_81,	66,7	65,4	109,1	80,4
Contig_524909	pfam01074	Glyco_hydro_38,	84,8	64,0	88,7	79,1
Contig_547962	pfam01301	Glyco_hydro_35,	59,3	77,0	96,8	77,7
Contig_376460	pfam10566	Glyco_hydro_97,	69,1	35,9	125,3	76,8
Contig_576917	pfam01630	Glyco_hydro_56,	100,2	94,4	34,4	76,3
Contig_316388	pfam01532	Glyco_hydro_47,	50,3	50,9	122,8	74,6
Contig_576781	pfam13199	Glyco_hydro_66,	66,9	39,6	113,7	73,4
Contig_482124	pfam12899	Glyco_hydro_100,	69,5	99,0	49,8	72,8
Contig_183429	pfam04616	Glyco_hydro_43,	56,0	53,0	106,1	71,7
Contig_522669	pfam00232	Glyco_hydro_1,	69,7	92,9	52,1	71,6
Contig_218199	pfam01374	Glyco_hydro_46,	71,1	84,0	56,7	70,6
Contig_29471	pfam03639	Glyco_hydro_81,	39,0	20,3	147,3	68,9
Contig_543127	pfam03632	Glyco_hydro_65m,	65,1	48,0	92,4	68,5
Contig_261422	pfam02446	Glyco_hydro_77,	41,2	72,6	82,1	65,3
Contig_394985	pfam02837	Glyco_hydro_2_N,	58,0	37,5	99,3	64,9
Contig_553969	pfam00295	Glyco_hydro_28,	77,9	77,1	36,1	63,7
Contig_213848	pfam01532	Glyco_hydro_47,	39,3	64,7	81,9	62,0
Contig_505343	pfam03198	Glyco_hydro_72,	51,6	74,1	58,6	61,4
Contig_502657	pfam12899	Glyco_hydro_100,	71,4	71,5	40,5	61,1
Contig_543811	pfam03512	Glyco_hydro_52,	52,5	34,7	95,6	60,9
Contig_190423	pfam03639	Glyco_hydro_81,	32,2	46,4	103,3	60,6
Contig_558363	pfam01229	Glyco_hydro_39,	61,4	58,8	60,2	60,1
Contig_391816	pfam04616	Glyco_hydro_43,	77,3	81,4	20,7	59,8
Contig_526118	pfam01074	Glyco_hydro_38,	93,8	58,2	27,3	59,8
Contig_527940	pfam00722	Glyco_hydro_16,	48,3	49,3	81,0	59,5
Contig_574420	pfam12899	Glyco_hydro_100,	33,0	54,1	90,9	59,3
Contig_530013	pfam03639	Glyco_hydro_81,	55,1	62,9	56,1	58,0
Contig_500972	pfam13199	Glyco_hydro_66,	40,3	42,1	91,1	57,8
Contig_432010	pfam01301	Glyco_hydro_35,	49,0	31,3	90,5	57,0
Contig_393729	pfam00728	Glyco_hydro_20,	66,7	61,3	42,4	56,8
Contig_398126	pfam03663	Glyco_hydro_76,	43,0	67,7	59,1	56,6
Contig_541160	pfam02324	Glyco_hydro_70,	55,2	43,7	69,9	56,3
Contig_428252	pfam03065	Glyco_hydro_57,	60,4	46,5	61,1	56,0
Contig_551212	pfam03633	Glyco_hydro_65C,	73,9	71,9	20,2	55,3
Contig_575179	pfam07971	Glyco_hydro_92,	62,6	49,4	52,6	54,9
Contig_556036	pfam00295	Glyco_hydro_28,	27,9	61,4	74,2	54,5
Contig_530425	pfam07488	Glyco_hydro_67M,	68,7	43,7	50,3	54,2
Contig_470338	pfam02446	Glyco_hydro_77,	63,2	47,0	51,7	54,0
Contig_420403	pfam05838	Glyco_hydro_108,	66,3	54,5	40,7	53,8
Contig_548188	pfam00251	Glyco_hydro_32N,	59,4	39,0	62,6	53,7
Contig_569843	pfam03639	Glyco_hydro_81,	50,1	36,4	74,0	53,5
Contig_543634	pfam00704	Glyco_hydro_18,	47,8	68,5	43,1	53,1
Contig_248122	pfam01055	Glyco_hydro_31,	61,5	37,5	59,9	53,0
Contig_248731	pfam00759	Glyco_hydro_9,	58,6	35,3	59,9	51,3
Contig_577378	pfam07971	Glyco_hydro_92,	63,4	65,8	24,5	51,2
Contig_541091	pfam01630	Glyco_hydro_56,	42,6	49,1	60,9	50,8
Contig_571329	pfam02435	Glyco_hydro_68,	36,5	43,0	70,4	50,0
Contig_392151	pfam02446	Glyco_hydro_77,	67,8	44,8	36,5	49,7
Contig_523039	pfam07745	Glyco_hydro_53,	61,5	61,3	25,1	49,3
Contig_197284	pfam01630	Glyco_hydro_56,	42,9	31,5	73,1	49,2
Contig_287949	pfam00251	Glyco_hydro_32N,	57,2	39,2	50,4	48,9
Contig_26676	pfam03659	Glyco_hydro_71,	55,0	62,6	26,1	47,9
Contig_386666	pfam00232	Glyco_hydro_1,	47,4	36,6	59,0	47,7
Contig_313919	pfam03644	Glyco_hydro_85,	50,2	54,2	38,4	47,6
Contig_577086	pfam00232	Glyco_hydro_1,	57,5	42,5	42,3	47,4
Contig_331434	pfam07470	Glyco_hydro_88,	60,8	44,6	35,0	46,8
Contig_532816	pfam01055	Glyco_hydro_31,	53,1	36,5	50,4	46,7
Contig_535360	pfam00295	Glyco_hydro_28,	46,1	45,4	47,0	46,2
Contig_555069	pfam03644	Glyco_hydro_85,	24,3	37,1	75,2	45,5

Total expressed genes	4784132
Sum of expressed* glycoside hydrolase genes	30954
**% of glycoside hydrolase families**	**0,65**

* FPKM normalized

Contig_342334	pfam01055	Glyco_hydro_31,	35,7	32,1	68,6	45,4
Contig_352397	pfam00933	Glyco_hydro_3,	26,8	37,6	72,0	45,4
Contig_430957	pfam12899	Glyco_hydro_100,	36,9	47,4	51,9	45,4
Contig_555007	pfam00728	Glyco_hydro_20,	52,9	39,2	43,1	45,0
Contig_42645	pfam03200	Glyco_hydro_63,	48,3	22,0	64,8	45,0
Contig_523098	pfam01301	Glyco_hydro_35,	24,5	45,3	65,2	45,0
Contig_126100	pfam07470	Glyco_hydro_88,	50,5	57,0	26,5	44,7
Contig_383172	pfam12905	Glyco_hydro_101,	40,4	45,1	47,5	44,3
Contig_440123	pfam13199	Glyco_hydro_66,	39,1	43,3	49,7	44,1
Contig_300174	pfam01055	Glyco_hydro_31,	48,6	35,3	47,9	43,9
Contig_534922	pfam02449	Glyco_hydro_42,	42,1	43,6	45,3	43,7
Contig_154554	pfam07477	Glyco_hydro_67C,	25,1	45,4	59,8	43,4
Contig_184781	pfam02324	Glyco_hydro_70,	34,3	50,3	45,4	43,3
Contig_478616	pfam03639	Glyco_hydro_81,	39,7	34,1	55,8	43,2
Contig_205006	pfam01055	Glyco_hydro_31,	52,2	32,5	43,8	42,8
Contig_293418	pfam01630	Glyco_hydro_56,	55,3	56,2	16,6	42,7
Contig_164335	pfam01229	Glyco_hydro_39,	44,2	47,7	35,8	42,6
Contig_568030	pfam00251	Glyco_hydro_32N,	36,9	27,5	61,1	41,8
Contig_394321	pfam02449	Glyco_hydro_42,	36,9	49,4	37,6	41,3
Contig_443013	pfam01229	Glyco_hydro_39,	29,0	33,8	60,6	41,1
Contig_524025	pfam01915	Glyco_hydro_3_C,	32,0	25,6	65,2	40,9
Contig_574747	pfam02324	Glyco_hydro_70,	60,4	43,7	18,1	40,7
Contig_524895	pfam00251	Glyco_hydro_32N,	39,0	35,8	46,9	40,6
Contig_188577	pfam02324	Glyco_hydro_70,	28,7	37,1	55,7	40,5
Contig_574890	pfam04616	Glyco_hydro_43,	52,6	32,1	36,2	40,3
Contig_445013	pfam03659	Glyco_hydro_71,	23,6	30,2	66,6	40,1
Contig_467894	pfam01630	Glyco_hydro_56,	26,7	66,0	26,7	39,8
Contig_406610	pfam01915	Glyco_hydro_3_C,	48,7	37,4	32,1	39,4
Contig_334103	pfam03200	Glyco_hydro_63,	8,1	25,8	83,9	39,3
Contig_532502	pfam00722	Glyco_hydro_16,	49,3	42,7	25,1	39,1
Contig_196790	pfam08244	Glyco_hydro_32C,	29,9	30,5	55,4	38,6
Contig_552750	pfam00722	Glyco_hydro_16,	26,4	52,6	36,4	38,5
Contig_476228	pfam12899	Glyco_hydro_100,	49,5	44,5	21,4	38,4
Contig_424287	pfam00232	Glyco_hydro_1,	52,4	26,0	36,0	38,1
Contig_361457	pfam07745	Glyco_hydro_53,	45,5	42,9	26,0	38,1
Contig_163643	pfam03644	Glyco_hydro_85,	45,8	55,3	12,8	38,0
Contig_507574	pfam00232	Glyco_hydro_1,	1,4	6,0	106,4	37,9
Contig_553692	pfam11790	Glyco_hydro_cc,	56,5	53,6	3,4	37,8
Contig_380422	pfam07488	Glyco_hydro_67M,	50,8	21,9	40,0	37,5
Contig_207817	pfam12899	Glyco_hydro_100,	21,5	21,3	69,5	37,4
Contig_294232	pfam01301	Glyco_hydro_35,	46,0	36,8	29,4	37,4
Contig_292633	pfam03512	Glyco_hydro_52,	30,2	35,6	46,0	37,3
Contig_227031	pfam07488	Glyco_hydro_67M,	44,5	27,6	39,3	37,2
Contig_547603	pfam02446	Glyco_hydro_77,	38,8	26,0	45,8	36,9
Contig_497209	pfam03662	Glyco_hydro_79n,	37,9	37,9	34,7	36,9
Contig_561670	pfam02324	Glyco_hydro_70,	38,0	41,3	30,6	36,6
Contig_169905	pfam00840	Glyco_hydro_7,	21,5	37,3	50,5	36,4
Contig_576662	pfam00232	Glyco_hydro_1,	47,1	43,2	18,7	36,3
Contig_556595	pfam00232	Glyco_hydro_1,	49,7	37,5	21,6	36,3
Contig_336122	pfam00331	Glyco_hydro_10,	31,0	37,4	40,3	36,2
Contig_319255	pfam03659	Glyco_hydro_71,	40,6	34,1	33,7	36,1
Contig_555383	pfam07971	Glyco_hydro_92,	46,3	58,9	3,2	36,1
Contig_446691	pfam03200	Glyco_hydro_63,	36,2	45,8	26,3	36,1
Contig_570486	pfam02836	Glyco_hydro_2_C,	19,3	41,2	47,7	36,0
Contig_526434	pfam07748	Glyco_hydro_38C,	48,9	38,5	20,4	36,0
Contig_561874	pfam00295	Glyco_hydro_28,	47,5	54,6	5,5	35,9
Contig_526141	pfam02837	Glyco_hydro_2_N,	29,7	27,7	48,9	35,4
Contig_533468	pfam03200	Glyco_hydro_63,	28,1	42,9	35,0	35,3
Contig_434854	pfam02446	Glyco_hydro_77,	35,9	26,8	43,0	35,2
Contig_79129	pfam07971	Glyco_hydro_92,	18,5	16,4	70,5	35,1
Contig_280820	pfam13199	Glyco_hydro_66,	4,7	24,1	76,5	35,1
Contig_179242	pfam00295	Glyco_hydro_28,	25,5	27,2	52,0	34,9
Contig_433208	pfam03512	Glyco_hydro_52,	25,6	38,2	40,5	34,7
Contig_529904	pfam03198	Glyco_hydro_72,	35,7	16,2	52,3	34,7
Contig_291736	pfam02324	Glyco_hydro_70,	33,3	39,0	31,8	34,7
Contig_542281	pfam00759	Glyco_hydro_9,	20,1	40,4	42,9	34,5
Contig_178287	pfam03644	Glyco_hydro_85,	29,3	33,1	40,6	34,3
Contig_524703	pfam03512	Glyco_hydro_52,	21,5	37,4	43,9	34,3
Contig_36831	pfam02324	Glyco_hydro_70,	38,5	33,1	31,0	34,2
Contig_446118	pfam08244	Glyco_hydro_32C,	44,0	33,5	24,7	34,1
Contig_391600	pfam00232	Glyco_hydro_1,	67,4	34,8	0,0	34,1

Contig_427633	pfam12891	Glyco_hydro_44,	25,2	29,8	47,0	34,0
Contig_379105	pfam00722	Glyco_hydro_16,	33,9	35,9	32,1	34,0
Contig_474158	pfam02011	Glyco_hydro_48,	27,4	32,2	42,2	34,0
Contig_374899	pfam01532	Glyco_hydro_47,	61,0	34,8	5,3	33,7
Contig_68884	pfam00933	Glyco_hydro_3,	48,5	25,8	26,5	33,6
Contig_540413	pfam03639	Glyco_hydro_81,	37,6	37,3	25,9	33,6
Contig_575028	pfam00295	Glyco_hydro_28,	24,6	22,6	53,4	33,5
Contig_526033	pfam03648	Glyco_hydro_67N,	42,1	52,5	5,9	33,5
Contig_32494	pfam03644	Glyco_hydro_85,	11,7	5,9	82,5	33,4
Contig_159338	pfam00728	Glyco_hydro_20,	27,5	30,0	42,6	33,4
Contig_534250	pfam00295	Glyco_hydro_28,	50,2	24,2	24,9	33,1
Contig_122340	pfam00232	Glyco_hydro_1,	28,3	26,2	44,1	32,9
Contig_424670	pfam03200	Glyco_hydro_63,	27,1	23,8	47,2	32,7
Contig_420620	pfam03198	Glyco_hydro_72,	17,4	31,5	49,1	32,7
Contig_206360	pfam00331	Glyco_hydro_10,	21,5	12,2	64,1	32,6
Contig_272768	pfam07971	Glyco_hydro_92,	41,2	54,1	2,5	32,6
Contig_24743	pfam03632	Glyco_hydro_65m,	19,9	25,4	52,5	32,6
Contig_481804	pfam01374	Glyco_hydro_46,	19,3	36,4	41,8	32,5
Contig_525804	pfam00295	Glyco_hydro_28,	29,8	47,7	19,5	32,3
Contig_541967	pfam02055	Glyco_hydro_30,	20,4	29,0	47,6	32,3
Contig_562173	pfam03662	Glyco_hydro_79n,	45,0	38,1	13,7	32,3
Contig_530021	pfam01055	Glyco_hydro_31,	29,5	52,5	14,8	32,2
Contig_583572	pfam01301	Glyco_hydro_35,	24,7	34,8	37,3	32,2
Contig_438191	pfam03200	Glyco_hydro_63,	11,9	16,9	67,3	32,0
Contig_554885	pfam00295	Glyco_hydro_28,	28,9	37,6	29,3	31,9
Contig_2562	pfam01915	Glyco_hydro_3_C,	19,2	8,5	67,9	31,9
Contig_205549	pfam00457	Glyco_hydro_11,	37,4	50,1	8,2	31,9
Contig_522656	pfam00182	Glyco_hydro_19,	24,6	38,3	32,5	31,8
Contig_423351	pfam11790	Glyco_hydro_cc,	18,8	14,1	61,9	31,6
Contig_45479	pfam00295	Glyco_hydro_28,	3,2	0,7	90,8	31,6
Contig_283844	pfam00933	Glyco_hydro_3,	36,8	36,0	21,4	31,4
Contig_46582	pfam00232	Glyco_hydro_1,	27,1	36,6	30,2	31,3
Contig_410483	pfam07488	Glyco_hydro_67M,	11,1	29,5	52,9	31,1
Contig_88747	pfam00295	Glyco_hydro_28,	15,8	29,4	48,0	31,1
Contig_388745	pfam02446	Glyco_hydro_77,	5,6	12,6	73,8	30,7
Contig_57088	pfam00232	Glyco_hydro_1,	17,7	23,6	50,1	30,5
Contig_581888	pfam01074	Glyco_hydro_38,	25,0	13,3	53,0	30,4
Contig_546399	pfam03662	Glyco_hydro_79n,	23,6	21,5	45,0	30,1
Contig_562178	pfam03200	Glyco_hydro_63,	34,5	38,7	16,8	30,0
Contig_327660	pfam01630	Glyco_hydro_56,	4,9	8,8	76,2	30,0
Contig_184977	pfam02837	Glyco_hydro_2_N,	27,7	31,2	30,9	29,9
Contig_58573	pfam02057	Glyco_hydro_59,	22,3	23,5	44,0	29,9
Contig_214314	pfam13199	Glyco_hydro_66,	32,8	32,6	24,3	29,9
Contig_192365	pfam13199	Glyco_hydro_66,	21,0	28,8	39,7	29,8
Contig_562198	pfam10566	Glyco_hydro_97,	23,0	28,8	37,3	29,7
Contig_471076	pfam00457	Glyco_hydro_11,	43,3	36,1	9,4	29,6
Contig_216706	pfam03639	Glyco_hydro_81,	32,2	32,1	24,3	29,5
Contig_273252	pfam01074	Glyco_hydro_38,	43,9	30,6	13,8	29,4
Contig_88069	pfam00723	Glyco_hydro_15,	29,6	44,0	14,2	29,3
Contig_425541	pfam07745	Glyco_hydro_53,	28,6	31,5	27,6	29,2
Contig_230358	pfam01532	Glyco_hydro_47,	9,8	55,8	21,9	29,1
Contig_575306	pfam00331	Glyco_hydro_10,	34,5	28,2	24,5	29,1
Contig_236204	pfam00232	Glyco_hydro_1,	26,0	33,7	26,8	28,8
Contig_275106	pfam02446	Glyco_hydro_77,	14,1	16,9	55,4	28,8
Contig_477405	pfam02449	Glyco_hydro_42,	27,9	32,4	25,8	28,7
Contig_146589	pfam03718	Glyco_hydro_49,	22,2	27,7	36,1	28,7
Contig_583297	pfam00295	Glyco_hydro_28,	17,7	20,1	47,4	28,4
Contig_443612	pfam03644	Glyco_hydro_85,	37,8	38,2	9,1	28,4
Contig_525873	pfam03639	Glyco_hydro_81,	35,8	45,2	4,1	28,3
Contig_336931	pfam02446	Glyco_hydro_77,	22,9	22,6	39,4	28,3
Contig_540878	pfam01270	Glyco_hydro_8,	11,9	4,3	68,3	28,2
Contig_398645	pfam01229	Glyco_hydro_39,	20,0	16,6	47,9	28,1
Contig_473890	pfam03200	Glyco_hydro_63,	24,3	36,6	23,3	28,1
Contig_436187	pfam01301	Glyco_hydro_35,	15,8	30,5	37,7	28,0
Contig_210067	pfam00722	Glyco_hydro_16,	23,3	35,2	25,1	27,9
Contig_441913	pfam12899	Glyco_hydro_100,	20,0	30,0	33,1	27,7
Contig_435772	pfam03659	Glyco_hydro_71,	23,4	20,8	38,7	27,6
Contig_425480	pfam00759	Glyco_hydro_9,	14,6	18,1	49,9	27,5
Contig_327882	pfam00332	Glyco_hydro_17,	30,4	24,3	27,5	27,4
Contig_254759	pfam03639	Glyco_hydro_81,	26,6	31,9	23,6	27,4
Contig_220308	pfam00295	Glyco_hydro_28,	22,8	36,8	21,9	27,2

Contig_85876	pfam00759	Glyco_hydro_9,	1,3	32,4	46,9	26,9
Contig_576157	pfam00232	Glyco_hydro_1,	27,0	25,8	27,5	26,8
Contig_398080	pfam00232	Glyco_hydro_1,	23,6	27,3	29,3	26,7
Contig_343395	pfam08307	Glyco_hydro_98C,	32,6	29,1	18,1	26,6
Contig_188200	pfam08307	Glyco_hydro_98C,	31,4	44,8	3,6	26,6
Contig_466230	pfam04616	Glyco_hydro_43,	18,4	23,1	38,1	26,6
Contig_88696	pfam02156	Glyco_hydro_26,	22,1	21,6	35,9	26,5
Contig_6240	pfam02057	Glyco_hydro_59,	23,3	19,3	36,6	26,4
Contig_581971	pfam02449	Glyco_hydro_42,	33,3	36,7	8,6	26,2
Contig_544966	pfam00232	Glyco_hydro_1,	36,6	33,2	8,4	26,0
Contig_481598	pfam02055	Glyco_hydro_30,	22,3	24,7	31,0	26,0
Contig_228315	pfam01373	Glyco_hydro_14,	14,9	26,3	36,8	26,0
Contig_330846	pfam00332	Glyco_hydro_17,	26,9	16,4	34,7	26,0
Contig_529742	pfam00295	Glyco_hydro_28,	20,9	24,4	32,5	25,9
Contig_217723	pfam03198	Glyco_hydro_72,	26,8	38,8	12,1	25,9
Contig_440697	pfam01183	Glyco_hydro_25,	31,8	22,8	22,3	25,7
Contig_182605	pfam02057	Glyco_hydro_59,	15,2	29,6	32,1	25,6
Contig_423718	pfam03659	Glyco_hydro_71,	20,2	28,8	27,8	25,6
Contig_436393	pfam00295	Glyco_hydro_28,	18,1	13,1	45,1	25,4
Contig_226418	pfam02055	Glyco_hydro_30,	7,5	11,8	56,7	25,3
Contig_282472	pfam02156	Glyco_hydro_26,	25,7	22,0	28,2	25,3
Contig_541535	pfam10566	Glyco_hydro_97,	10,3	33,3	32,2	25,3
Contig_45697	pfam07971	Glyco_hydro_92,	4,9	4,9	65,4	25,1
Contig_15661	pfam03198	Glyco_hydro_72,	19,6	14,8	40,6	25,0
Contig_442678	pfam01670	Glyco_hydro_12,	23,6	31,3	19,8	24,9
Contig_265873	pfam03662	Glyco_hydro_79n,	28,3	26,6	19,8	24,9
Contig_193951	pfam02449	Glyco_hydro_42,	18,2	31,0	25,2	24,8
Contig_188135	pfam00232	Glyco_hydro_1,	24,9	13,5	36,0	24,8
Contig_449840	pfam00182	Glyco_hydro_19,	22,7	41,7	9,9	24,8
Contig_468759	pfam00295	Glyco_hydro_28,	24,0	40,2	10,1	24,7
Contig_309647	pfam00232	Glyco_hydro_1,	21,0	43,7	9,5	24,7
Contig_358026	pfam03639	Glyco_hydro_81,	25,4	19,1	29,6	24,7
Contig_68544	pfam01532	Glyco_hydro_47,	24,4	37,5	12,2	24,7
Contig_544805	pfam07745	Glyco_hydro_53,	22,7	23,2	28,1	24,7
Contig_559206	pfam12905	Glyco_hydro_101,	22,3	31,7	19,7	24,6
Contig_541179	pfam03632	Glyco_hydro_65m,	10,7	36,2	26,7	24,5
Contig_506912	pfam01229	Glyco_hydro_39,	13,5	16,2	43,9	24,5
Contig_192013	pfam03639	Glyco_hydro_81,	28,6	31,7	12,9	24,4
Contig_445287	pfam03200	Glyco_hydro_63,	28,4	21,5	23,3	24,4
Contig_290973	pfam00232	Glyco_hydro_1,	22,1	15,9	35,2	24,4
Contig_195571	pfam00251	Glyco_hydro_32N,	19,2	24,1	29,6	24,3
Contig_430515	pfam00232	Glyco_hydro_1,	10,4	26,9	35,4	24,2
Contig_277711	pfam03639	Glyco_hydro_81,	39,3	33,2	0,0	24,2
Contig_95291	pfam03200	Glyco_hydro_63,	45,0	17,8	9,7	24,2
Contig_315327	pfam03662	Glyco_hydro_79n,	22,8	26,8	22,8	24,1
Contig_311638	pfam00722	Glyco_hydro_16,	27,7	22,8	21,7	24,0
Contig_521704	pfam00759	Glyco_hydro_9,	23,1	17,8	31,1	24,0
Contig_339458	pfam02837	Glyco_hydro_2_N,	24,8	26,7	20,5	24,0
Contig_374518	pfam01055	Glyco_hydro_31,	28,1	22,2	21,7	24,0
Contig_272077	pfam00232	Glyco_hydro_1,	18,9	21,2	31,8	24,0
Contig_430669	pfam00331	Glyco_hydro_10,	24,0	30,2	17,6	24,0
Contig_558947	pfam00232	Glyco_hydro_1,	20,5	13,8	37,3	23,9
Contig_39761	pfam01301	Glyco_hydro_35,	17,7	18,0	35,8	23,8
Contig_312306	pfam01229	Glyco_hydro_39,	6,2	11,1	54,1	23,8
Contig_469379	pfam00295	Glyco_hydro_28,	17,0	31,9	22,5	23,8
Contig_128350	pfam01630	Glyco_hydro_56,	10,0	14,3	46,9	23,8
Contig_301563	pfam03200	Glyco_hydro_63,	12,3	28,2	30,7	23,7
Contig_342921	pfam03200	Glyco_hydro_63,	16,5	23,9	30,3	23,6
Contig_433809	pfam02449	Glyco_hydro_42,	20,4	23,0	27,3	23,5
Contig_497801	pfam02837	Glyco_hydro_2_N,	29,6	19,6	21,4	23,5
Contig_522482	pfam00295	Glyco_hydro_28,	27,9	40,5	2,2	23,5
Contig_29297	pfam00457	Glyco_hydro_11,	18,7	21,5	29,9	23,4
Contig_220313	pfam07477	Glyco_hydro_67C,	28,8	24,3	17,0	23,4
Contig_570414	pfam02057	Glyco_hydro_59,	18,9	13,8	37,1	23,3
Contig_562982	pfam01229	Glyco_hydro_39,	30,4	31,0	8,2	23,2
Contig_401627	pfam00728	Glyco_hydro_20,	20,1	31,3	18,1	23,2
Contig_273067	pfam00331	Glyco_hydro_10,	14,5	12,0	43,0	23,2
Contig_523771	pfam00232	Glyco_hydro_1,	11,3	13,0	45,2	23,1
Contig_522326	pfam07971	Glyco_hydro_92,	33,2	17,4	18,7	23,1
Contig_583837	pfam01373	Glyco_hydro_14,	37,3	29,8	2,2	23,1
Contig_191022	pfam03065	Glyco_hydro_57,	15,7	26,5	26,9	23,0

Contig_20970	pfam00232	Glyco_hydro_1,	10,5	9,5	49,2	23,0
Contig_575037	pfam01532	Glyco_hydro_47,	31,6	34,3	3,2	23,0
Contig_546997	pfam01055	Glyco_hydro_31,	22,2	21,7	25,1	23,0
Contig_535026	pfam01630	Glyco_hydro_56,	28,3	19,3	21,1	22,9
Contig_178877	pfam00933	Glyco_hydro_3,	24,9	23,5	20,2	22,9
Contig_183682	pfam00295	Glyco_hydro_28,	16,3	23,6	28,6	22,8
Contig_195183	pfam02449	Glyco_hydro_42,	24,7	23,9	19,8	22,8
Contig_178741	pfam00331	Glyco_hydro_10,	24,9	27,9	15,5	22,8
Contig_438145	pfam01915	Glyco_hydro_3_C,	5,8	14,5	47,5	22,6
Contig_601	pfam03632	Glyco_hydro_65m,	12,4	14,1	41,3	22,6
Contig_504509	pfam02324	Glyco_hydro_70,	27,3	32,0	8,1	22,5
Contig_472567	pfam02836	Glyco_hydro_2_C,	13,6	25,4	27,9	22,3
Contig_202237	pfam03718	Glyco_hydro_49,	23,1	28,4	15,5	22,3
Contig_30355	pfam07971	Glyco_hydro_92,	12,7	22,2	31,9	22,3
Contig_305632	pfam07971	Glyco_hydro_92,	23,5	14,2	29,1	22,3
Contig_383462	pfam00251	Glyco_hydro_32N,	43,9	18,7	4,1	22,2
Contig_35976	pfam00759	Glyco_hydro_9,	4,6	0,0	61,8	22,1
Contig_193222	pfam03662	Glyco_hydro_79n,	20,7	24,5	21,2	22,1
Contig_470862	pfam02435	Glyco_hydro_68,	19,9	30,0	16,5	22,1
Contig_437219	pfam00232	Glyco_hydro_1,	28,0	30,2	8,1	22,1
Contig_218898	pfam02056	Glyco_hydro_4,	29,9	17,3	18,9	22,1
Contig_497811	pfam01532	Glyco_hydro_47,	42,1	21,1	2,9	22,0
Contig_210773	pfam02836	Glyco_hydro_2_C,	26,3	10,6	29,1	22,0
Contig_333497	pfam01630	Glyco_hydro_56,	18,6	25,3	22,1	22,0
Contig_225473	pfam00232	Glyco_hydro_1,	7,7	15,7	42,4	21,9
Contig_561846	pfam00723	Glyco_hydro_15,	26,0	39,7	0,0	21,9
Contig_137686	pfam00182	Glyco_hydro_19,	19,9	23,0	22,8	21,9
Contig_448599	pfam07477	Glyco_hydro_67C,	18,4	21,6	25,5	21,9
Contig_423513	pfam03512	Glyco_hydro_52,	22,7	19,3	23,4	21,8
Contig_429476	pfam13199	Glyco_hydro_66,	22,0	17,4	25,6	21,7
Contig_420243	pfam03718	Glyco_hydro_49,	8,6	17,4	38,8	21,6
Contig_129406	pfam01630	Glyco_hydro_56,	27,5	21,2	15,8	21,5
Contig_350113	pfam03644	Glyco_hydro_85,	21,5	25,5	17,3	21,4
Contig_499943	pfam12899	Glyco_hydro_100,	1,3	21,5	41,4	21,4
Contig_429732	pfam07748	Glyco_hydro_38C,	15,4	30,7	18,0	21,4
Contig_24914	pfam02057	Glyco_hydro_59,	6,1	4,6	53,5	21,4
Contig_549942	pfam03200	Glyco_hydro_63,	43,5	20,6	0,0	21,4
Contig_446271	pfam01374	Glyco_hydro_46,	15,1	19,0	30,0	21,3
Contig_314449	pfam03659	Glyco_hydro_71,	19,7	24,4	19,8	21,3
Contig_558863	pfam08307	Glyco_hydro_98C,	27,2	36,6	0,0	21,2
Contig_468389	pfam03633	Glyco_hydro_65C,	12,6	19,7	31,1	21,2
Contig_576026	pfam02324	Glyco_hydro_70,	15,0	15,8	32,6	21,1
Contig_199429	pfam01532	Glyco_hydro_47,	32,6	20,5	10,2	21,1
Contig_467250	pfam13199	Glyco_hydro_66,	22,5	33,8	7,0	21,1
Contig_201482	pfam03443	Glyco_hydro_61,	5,2	5,5	52,4	21,1
Contig_456614	pfam03198	Glyco_hydro_72,	16,5	16,6	30,0	21,0
Contig_420988	pfam01055	Glyco_hydro_31,	16,8	22,9	23,3	21,0
Contig_551322	pfam03659	Glyco_hydro_71,	35,8	16,2	11,1	21,0
Contig_181623	pfam03200	Glyco_hydro_63,	11,8	11,2	40,0	21,0
Contig_100247	pfam13199	Glyco_hydro_66,	2,4	2,2	57,9	20,8
Contig_311825	pfam00232	Glyco_hydro_1,	26,1	36,2	0,0	20,8
Contig_8155	pfam03198	Glyco_hydro_72,	12,1	21,7	28,5	20,8
Contig_439333	pfam03443	Glyco_hydro_61,	14,4	25,5	22,3	20,8
Contig_424545	pfam00182	Glyco_hydro_19,	13,2	42,2	6,6	20,7
Contig_544974	pfam13199	Glyco_hydro_66,	18,5	35,4	8,1	20,7
Contig_445902	pfam08307	Glyco_hydro_98C,	33,1	28,6	0,0	20,6
Contig_383044	pfam02449	Glyco_hydro_42,	29,2	13,0	19,3	20,5
Contig_509102	pfam04616	Glyco_hydro_43,	22,4	22,1	17,0	20,5
Contig_218012	pfam00295	Glyco_hydro_28,	12,7	24,5	24,2	20,5
Contig_548416	pfam08307	Glyco_hydro_98C,	21,6	13,7	26,0	20,4
Contig_12475	pfam07488	Glyco_hydro_67M,	6,7	0,0	54,5	20,4
Contig_122021	pfam03663	Glyco_hydro_76,	21,8	35,6	3,6	20,4
Contig_188183	pfam00232	Glyco_hydro_1,	18,5	25,3	17,1	20,3
Contig_431877	pfam02449	Glyco_hydro_42,	20,2	16,5	23,8	20,1
Contig_74837	pfam01301	Glyco_hydro_35,	17,0	7,7	35,5	20,0
Contig_543086	pfam12899	Glyco_hydro_100,	36,1	18,7	5,0	19,9
Contig_198164	pfam02446	Glyco_hydro_77,	11,4	24,5	23,8	19,9
Contig_32867	pfam13199	Glyco_hydro_66,	17,0	21,8	20,9	19,9
Contig_39141	pfam01270	Glyco_hydro_8,	10,5	15,6	33,6	19,9
Contig_183235	pfam07477	Glyco_hydro_67C,	23,1	28,4	8,2	19,9
Contig_547014	pfam03663	Glyco_hydro_76,	18,8	24,4	16,4	19,9

Contig_473444	pfam00251	Glyco_hydro_32N,	14,5	12,9	32,2	19,9
Contig_116683	pfam02055	Glyco_hydro_30,	11,3	3,9	44,5	19,9
Contig_209029	pfam12905	Glyco_hydro_101,	22,2	20,2	17,2	19,8
Contig_214770	pfam02449	Glyco_hydro_42,	27,9	20,6	11,0	19,8
Contig_169361	pfam01055	Glyco_hydro_31,	14,5	22,9	22,1	19,8
Contig_387334	pfam00251	Glyco_hydro_32N,	9,4	10,4	39,5	19,8
Contig_445873	pfam02449	Glyco_hydro_42,	14,1	33,0	12,2	19,8
Contig_506682	pfam03200	Glyco_hydro_63,	19,0	28,9	11,3	19,7
Contig_7967	pfam00332	Glyco_hydro_17,	9,7	0,8	48,7	19,7
Contig_103615	pfam03662	Glyco_hydro_79n,	0,0	0,0	59,2	19,7
Contig_504827	pfam02837	Glyco_hydro_2_N,	18,4	21,3	19,3	19,7
Contig_182753	pfam00232	Glyco_hydro_1,	19,3	13,0	26,8	19,7
Contig_52595	pfam12899	Glyco_hydro_100,	22,9	14,7	21,5	19,7
Contig_8514	pfam03659	Glyco_hydro_71,	0,0	1,5	57,4	19,6
Contig_312599	pfam01270	Glyco_hydro_8,	20,7	15,0	23,1	19,6
Contig_191093	pfam07470	Glyco_hydro_88,	12,2	15,0	31,5	19,6
Contig_297651	pfam07748	Glyco_hydro_38C,	12,0	13,7	32,9	19,5
Contig_138001	pfam00332	Glyco_hydro_17,	9,3	14,1	35,2	19,5
Contig_285191	pfam02449	Glyco_hydro_42,	18,0	19,2	21,3	19,5
Contig_271027	pfam00728	Glyco_hydro_20,	25,8	25,2	7,5	19,5
Contig_432885	pfam00295	Glyco_hydro_28,	22,6	29,5	6,4	19,5
Contig_268318	pfam12899	Glyco_hydro_100,	19,1	17,9	21,2	19,4
Contig_344029	pfam00182	Glyco_hydro_19,	23,2	17,5	17,4	19,3
Contig_204567	pfam01055	Glyco_hydro_31,	14,9	25,6	17,2	19,3
Contig_45130	pfam00933	Glyco_hydro_3,	4,4	5,3	48,0	19,2
Contig_172445	pfam01074	Glyco_hydro_38,	14,8	16,0	26,9	19,2
Contig_18626	pfam00332	Glyco_hydro_17,	17,4	19,2	21,1	19,2
Contig_219713	pfam03662	Glyco_hydro_79n,	15,0	27,1	15,5	19,2
Contig_442301	pfam00933	Glyco_hydro_3,	15,1	14,6	27,7	19,1
Contig_213472	pfam07477	Glyco_hydro_67C,	27,9	24,8	4,7	19,1
Contig_227047	pfam03662	Glyco_hydro_79n,	12,0	30,3	15,0	19,1
Contig_244322	pfam00723	Glyco_hydro_15,	8,4	18,8	29,9	19,0
Contig_18390	pfam02446	Glyco_hydro_77,	18,3	31,2	7,5	19,0
Contig_523438	pfam03639	Glyco_hydro_81,	21,6	12,2	23,3	19,0
Contig_195121	pfam03633	Glyco_hydro_65C,	13,7	18,9	24,3	19,0
Contig_237671	pfam00232	Glyco_hydro_1,	9,3	9,3	38,3	19,0
Contig_452943	pfam00704	Glyco_hydro_18,	21,6	24,4	10,9	19,0
Contig_471286	pfam00332	Glyco_hydro_17,	22,7	19,7	14,2	18,9
Contig_528003	pfam00182	Glyco_hydro_19,	37,3	19,3	0,0	18,9
Contig_302522	pfam02435	Glyco_hydro_68,	21,7	18,1	16,6	18,8
Contig_52145	pfam01915	Glyco_hydro_3_C,	12,3	18,9	25,2	18,8
Contig_246679	pfam01373	Glyco_hydro_14,	14,3	20,7	21,4	18,8
Contig_100965	pfam00759	Glyco_hydro_9,	3,3	0,0	53,2	18,8
Contig_132086	pfam03443	Glyco_hydro_61,	6,3	12,7	37,5	18,8
Contig_358641	pfam12899	Glyco_hydro_100,	6,4	10,3	39,8	18,8
Contig_19283	pfam01055	Glyco_hydro_31,	4,2	4,1	48,1	18,8
Contig_178460	pfam03662	Glyco_hydro_79n,	24,8	31,6	0,0	18,8
Contig_229992	pfam00251	Glyco_hydro_32N,	13,2	31,1	12,0	18,7
Contig_543307	pfam12891	Glyco_hydro_44,	16,6	31,3	8,3	18,7
Contig_431026	pfam00251	Glyco_hydro_32N,	19,0	23,3	13,8	18,7
Contig_432544	pfam00331	Glyco_hydro_10,	11,1	19,5	25,5	18,7
Contig_472429	pfam00457	Glyco_hydro_11,	14,9	15,5	25,3	18,6
Contig_238007	pfam00251	Glyco_hydro_32N,	20,9	11,8	22,9	18,6
Contig_126901	pfam00232	Glyco_hydro_1,	9,8	15,4	30,5	18,6
Contig_230940	pfam03639	Glyco_hydro_81,	24,5	20,9	10,0	18,5
Contig_12874	pfam00295	Glyco_hydro_28,	13,7	18,3	23,3	18,4
Contig_1644	pfam01055	Glyco_hydro_31,	14,2	0,0	41,0	18,4
Contig_353414	pfam03198	Glyco_hydro_72,	23,5	16,9	14,8	18,4
Contig_395469	pfam01532	Glyco_hydro_47,	11,2	15,2	28,8	18,4
Contig_445313	pfam07488	Glyco_hydro_67M,	10,6	29,6	15,0	18,4
Contig_562883	pfam04616	Glyco_hydro_43,	32,2	20,9	2,1	18,4
Contig_504330	pfam01301	Glyco_hydro_35,	12,3	18,7	24,1	18,4
Contig_7278	pfam00722	Glyco_hydro_16,	6,5	0,5	48,1	18,4
Contig_21097	pfam07488	Glyco_hydro_67M,	11,2	3,1	40,7	18,3
Contig_424963	pfam00728	Glyco_hydro_20,	14,2	15,3	25,3	18,3
Contig_31153	pfam00295	Glyco_hydro_28,	21,9	13,6	19,4	18,3
Contig_33437	pfam03644	Glyco_hydro_85,	4,3	2,0	48,5	18,2
Contig_155439	pfam01532	Glyco_hydro_47,	21,9	13,7	18,8	18,1
Contig_386963	pfam01301	Glyco_hydro_35,	26,4	22,2	5,7	18,1
Contig_9124	pfam02836	Glyco_hydro_2_C,	7,7	25,9	20,7	18,1
Contig_107762	pfam01229	Glyco_hydro_39,	0,0	0,0	54,2	18,1

Contig_261859	pfam12899	Glyco_hydro_100,	8,8	30,2	14,8	18,0
Contig_417434	pfam00704	Glyco_hydro_18,	25,5	9,2	19,1	17,9
Contig_240465	pfam03200	Glyco_hydro_63,	19,0	18,5	16,2	17,9
Contig_844	pfam00723	Glyco_hydro_15,	7,2	9,4	37,1	17,9
Contig_63400	pfam00331	Glyco_hydro_10,	17,4	13,4	22,9	17,9
Contig_66680	pfam00251	Glyco_hydro_32N,	0,0	12,1	41,6	17,9
Contig_295211	pfam01229	Glyco_hydro_39,	22,2	18,0	13,4	17,9
Contig_175068	pfam01301	Glyco_hydro_35,	31,1	22,5	0,0	17,9
Contig_501983	pfam00232	Glyco_hydro_1,	15,1	16,2	22,1	17,8
Contig_47532	pfam02836	Glyco_hydro_2_C,	13,5	8,2	31,6	17,7
Contig_194469	pfam03200	Glyco_hydro_63,	11,5	16,9	24,8	17,7
Contig_453621	pfam01915	Glyco_hydro_3_C,	13,7	9,2	30,1	17,7
Contig_384709	pfam03200	Glyco_hydro_63,	22,2	18,3	12,6	17,7
Contig_533085	pfam03639	Glyco_hydro_81,	23,6	29,4	0,0	17,7
Contig_579585	pfam12891	Glyco_hydro_44,	51,0	2,0	0,0	17,7
Contig_62377	pfam00759	Glyco_hydro_9,	7,1	20,7	25,1	17,6
Contig_466110	pfam00722	Glyco_hydro_16,	12,5	7,0	33,4	17,6
Contig_552950	pfam03662	Glyco_hydro_79n,	26,6	26,1	0,0	17,5
Contig_16977	pfam01373	Glyco_hydro_14,	8,1	9,4	35,1	17,5
Contig_104953	pfam02449	Glyco_hydro_42,	12,8	15,3	24,3	17,4
Contig_183247	pfam03512	Glyco_hydro_52,	32,2	10,5	9,3	17,3
Contig_521518	pfam03664	Glyco_hydro_62,	26,7	17,9	7,4	17,3
Contig_388982	pfam01630	Glyco_hydro_56,	27,5	12,6	11,8	17,3
Contig_476340	pfam03443	Glyco_hydro_61,	26,4	8,8	16,5	17,2
Contig_31785	pfam00232	Glyco_hydro_1,	14,6	6,6	30,5	17,2
Contig_14455	pfam00840	Glyco_hydro_7,	13,6	0,8	37,2	17,2
Contig_177964	pfam01301	Glyco_hydro_35,	1,0	9,9	40,7	17,2
Contig_85148	pfam00332	Glyco_hydro_17,	15,3	24,9	11,2	17,1
Contig_102801	pfam02156	Glyco_hydro_26,	6,7	5,4	39,2	17,1
Contig_44124	pfam02156	Glyco_hydro_26,	6,2	0,5	44,6	17,1
Contig_281197	pfam03065	Glyco_hydro_57,	11,8	16,3	23,1	17,1
Contig_289455	pfam00295	Glyco_hydro_28,	16,5	28,4	6,2	17,0
Contig_349941	pfam00722	Glyco_hydro_16,	11,4	17,2	22,3	17,0
Contig_472056	pfam01373	Glyco_hydro_14,	3,3	14,1	33,5	17,0
Contig_265510	pfam01341	Glyco_hydro_6,	8,0	4,8	38,1	16,9
Contig_420271	pfam01074	Glyco_hydro_38,	12,6	18,9	19,1	16,9
Contig_715	pfam00232	Glyco_hydro_1,	1,6	3,9	45,0	16,9
Contig_366264	pfam03512	Glyco_hydro_52,	17,7	2,3	30,5	16,8
Contig_46127	pfam04616	Glyco_hydro_43,	0,0	0,0	50,3	16,8
Contig_527481	pfam03200	Glyco_hydro_63,	15,7	13,3	21,2	16,8
Contig_4316	pfam03639	Glyco_hydro_81,	7,4	4,0	38,8	16,7
Contig_51018	pfam00704	Glyco_hydro_18,	4,7	1,9	43,5	16,7
Contig_266271	pfam01229	Glyco_hydro_39,	12,3	20,6	17,0	16,7
Contig_438317	pfam00251	Glyco_hydro_32N,	23,9	19,7	6,4	16,7
Contig_37032	pfam02055	Glyco_hydro_30,	0,0	0,8	49,1	16,7
Contig_186452	pfam03639	Glyco_hydro_81,	19,5	14,2	16,1	16,6
Contig_337168	pfam01532	Glyco_hydro_47,	25,2	18,6	6,1	16,6
Contig_82045	pfam00251	Glyco_hydro_32N,	9,4	13,8	26,6	16,6
Contig_547563	pfam03644	Glyco_hydro_85,	13,9	10,7	25,1	16,6
Contig_8881	pfam02836	Glyco_hydro_2_C,	2,5	9,4	37,6	16,5
Contig_328671	pfam00331	Glyco_hydro_10,	28,3	18,7	2,4	16,4
Contig_201707	pfam03664	Glyco_hydro_62,	4,9	4,5	40,0	16,4
Contig_308957	pfam03200	Glyco_hydro_63,	12,2	19,7	17,4	16,4
Contig_191056	pfam03659	Glyco_hydro_71,	21,7	19,6	8,0	16,4
Contig_203222	pfam00251	Glyco_hydro_32N,	18,7	12,4	18,1	16,4
Contig_48100	pfam01532	Glyco_hydro_47,	12,1	14,6	22,5	16,4
Contig_19914	pfam03639	Glyco_hydro_81,	13,9	6,2	29,1	16,4
Contig_559607	pfam07748	Glyco_hydro_38C,	19,2	29,8	0,0	16,3
Contig_168006	pfam03198	Glyco_hydro_72,	10,2	24,5	14,1	16,3
Contig_1497	pfam01229	Glyco_hydro_39,	1,1	0,0	47,7	16,3
Contig_71209	pfam01630	Glyco_hydro_56,	0,6	10,2	37,9	16,2
Contig_560024	pfam01055	Glyco_hydro_31,	13,7	10,5	24,5	16,2
Contig_16036	pfam02838	Glyco_hydro_20b,	5,7	11,1	31,9	16,2
Contig_304919	pfam07971	Glyco_hydro_92,	30,9	17,7	0,0	16,2
Contig_209639	pfam03632	Glyco_hydro_65m,	17,1	26,9	4,5	16,1
Contig_540773	pfam00232	Glyco_hydro_1,	27,4	9,2	11,7	16,1
Contig_437079	pfam01074	Glyco_hydro_38,	16,2	23,3	8,8	16,1
Contig_28306	pfam03200	Glyco_hydro_63,	8,8	3,3	36,2	16,1
Contig_212646	pfam03198	Glyco_hydro_72,	23,3	21,1	3,7	16,0
Contig_50486	pfam00933	Glyco_hydro_3,	9,3	23,8	14,6	15,9
Contig_535214	pfam07745	Glyco_hydro_53,	12,6	18,1	16,9	15,8

Contig_346818	pfam11790	Glyco_hydro_cc,	16,6	22,6	8,3	15,8
Contig_424387	pfam02055	Glyco_hydro_30,	4,4	21,1	21,9	15,8
Contig_20356	pfam02156	Glyco_hydro_26,	8,3	9,5	29,4	15,8
Contig_428060	pfam01074	Glyco_hydro_38,	7,4	15,0	24,8	15,7
Contig_446755	pfam02324	Glyco_hydro_70,	18,5	26,9	1,6	15,7
Contig_576166	pfam03200	Glyco_hydro_63,	11,2	35,8	0,0	15,7
Contig_287396	pfam08306	Glyco_hydro_98M,	7,2	24,2	15,4	15,6
Contig_36107	pfam03065	Glyco_hydro_57,	0,0	0,0	46,9	15,6
Contig_339541	pfam03200	Glyco_hydro_63,	21,2	25,6	0,0	15,6
Contig_521212	pfam02836	Glyco_hydro_2_C,	22,6	17,1	7,1	15,6
Contig_269357	pfam02446	Glyco_hydro_77,	3,1	8,4	35,2	15,6
Contig_530127	pfam01630	Glyco_hydro_56,	11,0	11,6	24,1	15,6
Contig_476428	pfam00704	Glyco_hydro_18,	0,0	8,3	38,3	15,5
Contig_215589	pfam01374	Glyco_hydro_46,	12,1	7,3	27,2	15,5
Contig_148671	pfam03644	Glyco_hydro_85,	6,3	27,1	13,1	15,5
Contig_301095	pfam11975	Glyco_hydro_4C,	12,2	14,7	19,4	15,5
Contig_83119	pfam01183	Glyco_hydro_25,	16,8	15,6	14,0	15,5
Contig_39009	pfam03200	Glyco_hydro_63,	0,0	5,9	40,4	15,4
Contig_131883	pfam12899	Glyco_hydro_100,	18,6	20,4	7,1	15,4
Contig_535474	pfam07971	Glyco_hydro_92,	7,7	18,0	20,3	15,3
Contig_498736	pfam01532	Glyco_hydro_47,	17,0	7,7	21,3	15,3
Contig_55858	pfam03200	Glyco_hydro_63,	22,3	21,3	2,3	15,3
Contig_153010	pfam03644	Glyco_hydro_85,	21,5	0,9	23,2	15,2
Contig_473756	pfam00295	Glyco_hydro_28,	13,7	29,2	2,6	15,2
Contig_182312	pfam08307	Glyco_hydro_98C,	15,7	24,3	5,6	15,2
Contig_26046	pfam02435	Glyco_hydro_68,	5,6	9,4	30,6	15,2
Contig_395750	pfam03198	Glyco_hydro_72,	5,4	17,5	22,5	15,1
Contig_204531	pfam01915	Glyco_hydro_3_C,	9,7	26,6	9,0	15,1
Contig_310006	pfam03662	Glyco_hydro_79n,	11,8	6,1	27,3	15,1
Contig_14020	pfam02056	Glyco_hydro_4,	1,5	5,5	38,2	15,1
Contig_11898	pfam03644	Glyco_hydro_85,	7,9	20,7	16,5	15,1
Contig_46374	pfam01055	Glyco_hydro_31,	4,8	0,0	40,4	15,1
Contig_200055	pfam03632	Glyco_hydro_65m,	17,8	27,4	0,0	15,1
Contig_297183	pfam03644	Glyco_hydro_85,	14,3	13,2	17,6	15,0
Contig_208830	pfam00840	Glyco_hydro_7,	9,2	10,7	25,2	15,0
Contig_2705	pfam01301	Glyco_hydro_35,	3,6	0,0	41,6	15,0
Contig_545936	pfam02449	Glyco_hydro_42,	25,7	19,4	0,0	15,0
Contig_124364	pfam00704	Glyco_hydro_18,	8,9	0,0	36,1	15,0
Contig_391064	pfam07748	Glyco_hydro_38C,	19,3	18,1	7,5	15,0
Contig_46751	pfam03662	Glyco_hydro_79n,	4,3	19,1	21,5	15,0
Contig_467630	pfam13199	Glyco_hydro_66,	15,3	11,6	18,0	14,9
Contig_582321	pfam04616	Glyco_hydro_43,	27,4	17,4	0,0	14,9
Contig_100135	pfam00457	Glyco_hydro_11,	0,0	2,8	42,0	14,9
Contig_548247	pfam00722	Glyco_hydro_16,	16,8	20,2	7,8	14,9
Contig_48952	pfam01229	Glyco_hydro_39,	8,7	10,1	25,9	14,9
Contig_179983	pfam01301	Glyco_hydro_35,	3,7	13,2	27,4	14,8
Contig_554313	pfam03200	Glyco_hydro_63,	3,6	9,2	31,4	14,7
Contig_296261	pfam07971	Glyco_hydro_92,	17,2	23,8	3,1	14,7
Contig_20198	pfam03659	Glyco_hydro_71,	9,5	10,6	24,0	14,7
Contig_182591	pfam01270	Glyco_hydro_8,	11,8	16,7	15,6	14,7
Contig_453955	pfam03644	Glyco_hydro_85,	9,8	10,7	23,4	14,6
Contig_130037	pfam00332	Glyco_hydro_17,	5,9	11,1	26,9	14,6
Contig_302513	pfam02449	Glyco_hydro_42,	12,7	13,7	17,5	14,6
Contig_439362	pfam03662	Glyco_hydro_79n,	11,5	32,3	0,0	14,6
Contig_475916	pfam03200	Glyco_hydro_63,	13,7	15,8	14,2	14,6
Contig_176586	pfam01074	Glyco_hydro_38,	6,9	17,2	19,6	14,5
Contig_388133	pfam00840	Glyco_hydro_7,	6,9	22,6	13,9	14,5
Contig_406142	pfam00251	Glyco_hydro_32N,	19,7	3,0	20,7	14,5
Contig_87347	pfam01630	Glyco_hydro_56,	1,1	2,7	39,5	14,5
Contig_118054	pfam07971	Glyco_hydro_92,	5,1	2,5	35,7	14,4
Contig_62100	pfam10566	Glyco_hydro_97,	11,4	2,1	29,7	14,4
Contig_124187	pfam07748	Glyco_hydro_38C,	2,9	11,3	29,0	14,4
Contig_122463	pfam02324	Glyco_hydro_70,	8,4	16,9	17,8	14,4
Contig_49637	pfam00251	Glyco_hydro_32N,	0,0	0,0	43,0	14,3
Contig_85856	pfam00728	Glyco_hydro_20,	10,4	2,7	29,9	14,3
Contig_433604	pfam00251	Glyco_hydro_32N,	16,5	26,3	0,0	14,3
Contig_428062	pfam00232	Glyco_hydro_1,	20,8	17,2	4,8	14,2
Contig_226556	pfam00933	Glyco_hydro_3,	12,3	18,5	11,8	14,2
Contig_41145	pfam00728	Glyco_hydro_20,	5,5	10,8	26,2	14,2
Contig_342768	pfam00251	Glyco_hydro_32N,	4,4	5,3	32,9	14,2
Contig_82975	pfam01074	Glyco_hydro_38,	13,5	6,4	22,7	14,2

Contig_328168	pfam02011	Glyco_hydro_48,	17,9	12,8	11,8	14,2
Contig_8788	pfam07748	Glyco_hydro_38C,	5,5	10,8	26,2	14,2
Contig_111905	pfam03198	Glyco_hydro_72,	5,8	11,3	25,3	14,1
Contig_265140	pfam01915	Glyco_hydro_3_C,	5,1	18,2	19,1	14,1
Contig_5047	pfam01630	Glyco_hydro_56,	15,5	11,0	15,9	14,1
Contig_186334	pfam03659	Glyco_hydro_71,	11,4	15,4	15,6	14,1
Contig_318668	pfam03662	Glyco_hydro_79n,	18,8	11,3	12,2	14,1
Contig_478497	pfam01301	Glyco_hydro_35,	6,3	11,8	24,2	14,1
Contig_176691	pfam00933	Glyco_hydro_3,	10,6	18,2	13,4	14,1
Contig_344487	pfam12899	Glyco_hydro_100,	9,6	15,2	17,4	14,1
Contig_172040	pfam07488	Glyco_hydro_67M,	17,4	23,1	1,7	14,1
Contig_23510	pfam03065	Glyco_hydro_57,	0,0	8,9	33,1	14,0
Contig_23796	pfam07971	Glyco_hydro_92,	10,1	5,4	26,4	14,0
Contig_530689	pfam00251	Glyco_hydro_32N,	19,6	18,6	3,5	13,9
Contig_180679	pfam01373	Glyco_hydro_14,	8,1	11,0	22,6	13,9
Contig_284212	pfam00182	Glyco_hydro_19,	10,7	17,6	13,3	13,9
Contig_5149	pfam02446	Glyco_hydro_77,	7,0	2,8	31,8	13,9
Contig_138894	pfam03636	Glyco_hydro_65N,	7,1	10,8	23,6	13,8
Contig_166431	pfam00728	Glyco_hydro_20,	24,7	13,3	3,4	13,8
Contig_583264	pfam11790	Glyco_hydro_cc,	16,5	0,8	24,1	13,8
Contig_233904	pfam01915	Glyco_hydro_3_C,	4,4	6,1	30,7	13,7
Contig_394013	pfam02446	Glyco_hydro_77,	13,9	15,8	11,5	13,7
Contig_426780	pfam00295	Glyco_hydro_28,	11,6	19,9	9,7	13,7
Contig_574	pfam07971	Glyco_hydro_92,	1,5	1,8	37,8	13,7
Contig_126847	pfam03644	Glyco_hydro_85,	11,4	23,4	6,2	13,7
Contig_177456	pfam00251	Glyco_hydro_32N,	3,1	6,6	31,3	13,6
Contig_502538	pfam08307	Glyco_hydro_98C,	10,2	30,6	0,0	13,6
Contig_202336	pfam02011	Glyco_hydro_48,	11,1	6,7	23,0	13,6
Contig_503203	pfam00251	Glyco_hydro_32N,	14,4	15,3	11,0	13,6
Contig_123320	pfam00332	Glyco_hydro_17,	0,0	0,0	40,7	13,6
Contig_99386	pfam07748	Glyco_hydro_38C,	7,6	5,9	27,1	13,5
Contig_455704	pfam02011	Glyco_hydro_48,	10,5	15,5	14,6	13,5
Contig_142940	pfam01183	Glyco_hydro_25,	3,3	0,0	37,3	13,5
Contig_548471	pfam00840	Glyco_hydro_7,	15,0	25,4	0,0	13,5
Contig_282896	pfam02446	Glyco_hydro_77,	8,5	3,4	28,4	13,4
Contig_503504	pfam03718	Glyco_hydro_49,	9,7	30,6	0,0	13,4
Contig_475268	pfam03200	Glyco_hydro_63,	12,9	12,7	14,7	13,4
Contig_504632	pfam01532	Glyco_hydro_47,	0,0	6,5	33,7	13,4
Contig_17015	pfam03664	Glyco_hydro_62,	12,4	14,9	12,8	13,4
Contig_180267	pfam02324	Glyco_hydro_70,	12,5	24,7	2,9	13,4
Contig_38213	pfam01055	Glyco_hydro_31,	6,3	5,3	28,4	13,4
Contig_123620	pfam00759	Glyco_hydro_9,	0,0	7,0	33,1	13,4
Contig_235071	pfam00457	Glyco_hydro_11,	17,3	15,9	6,8	13,4
Contig_16367	pfam00704	Glyco_hydro_18,	8,8	1,3	29,9	13,3
Contig_386972	pfam01229	Glyco_hydro_39,	2,1	6,4	31,6	13,3
Contig_385341	pfam01373	Glyco_hydro_14,	21,5	18,5	0,0	13,3
Contig_472104	pfam00232	Glyco_hydro_1,	5,5	5,9	28,7	13,3
Contig_507298	pfam01532	Glyco_hydro_47,	9,9	20,2	9,9	13,3
Contig_583884	pfam01532	Glyco_hydro_47,	20,6	19,4	0,0	13,3
Contig_401043	pfam03639	Glyco_hydro_81,	19,9	18,5	1,5	13,3
Contig_115628	pfam04616	Glyco_hydro_43,	5,6	2,7	31,7	13,3
Contig_582089	pfam12891	Glyco_hydro_44,	30,3	9,6	0,0	13,3
Contig_366741	pfam00728	Glyco_hydro_20,	13,6	15,0	11,3	13,3
Contig_177544	pfam00332	Glyco_hydro_17,	21,2	18,5	0,0	13,2
Contig_225002	pfam00232	Glyco_hydro_1,	14,7	13,5	11,5	13,2
Contig_184166	pfam08306	Glyco_hydro_98M,	14,6	20,0	5,0	13,2
Contig_277811	pfam00704	Glyco_hydro_18,	23,6	13,6	2,4	13,2
Contig_345612	pfam07748	Glyco_hydro_38C,	13,2	16,7	9,6	13,2
Contig_488859	pfam03718	Glyco_hydro_49,	16,9	22,7	0,0	13,2
Contig_344103	pfam03632	Glyco_hydro_65m,	11,6	5,4	22,5	13,2
Contig_542187	pfam13199	Glyco_hydro_66,	15,4	24,1	0,0	13,2
Contig_552824	pfam02324	Glyco_hydro_70,	10,0	2,2	27,2	13,2
Contig_532603	pfam02324	Glyco_hydro_70,	14,5	0,7	24,2	13,1
Contig_675	pfam03644	Glyco_hydro_85,	2,8	0,0	36,5	13,1
Contig_256078	pfam01301	Glyco_hydro_35,	16,2	23,1	0,0	13,1
Contig_14549	pfam01532	Glyco_hydro_47,	6,6	9,3	23,4	13,1
Contig_35977	pfam00759	Glyco_hydro_9,	6,2	6,6	26,4	13,1
Contig_470097	pfam00182	Glyco_hydro_19,	14,0	11,7	13,5	13,1
Contig_38534	pfam03065	Glyco_hydro_57,	11,9	13,7	13,6	13,1
Contig_141974	pfam01915	Glyco_hydro_3_C,	10,3	17,5	11,4	13,1
Contig_188588	pfam00759	Glyco_hydro_9,	7,9	4,7	26,5	13,0

Contig_423994	pfam00759	Glyco_hydro_9,	21,5	17,5	0,0	13,0
Contig_30406	pfam00295	Glyco_hydro_28,	4,6	0,0	34,4	13,0
Contig_214530	pfam03443	Glyco_hydro_61,	5,3	30,3	3,3	13,0
Contig_19889	pfam03662	Glyco_hydro_79n,	16,3	22,6	0,0	12,9
Contig_22666	pfam04616	Glyco_hydro_43,	5,3	13,6	19,9	12,9
Contig_17080	pfam12899	Glyco_hydro_100,	17,7	3,2	17,7	12,9
Contig_3249	pfam01055	Glyco_hydro_31,	6,9	0,0	31,8	12,9
Contig_1818	pfam12899	Glyco_hydro_100,	18,1	4,0	16,6	12,9
Contig_152752	pfam00232	Glyco_hydro_1,	6,4	12,1	19,9	12,8
Contig_61282	pfam00457	Glyco_hydro_11,	8,8	3,6	25,9	12,8
Contig_130008	pfam13199	Glyco_hydro_66,	0,0	6,6	31,6	12,7
Contig_23976	pfam00840	Glyco_hydro_7,	11,3	11,3	15,6	12,7
Contig_422768	pfam00759	Glyco_hydro_9,	22,5	10,3	5,3	12,7
Contig_581131	pfam13199	Glyco_hydro_66,	19,8	18,4	0,0	12,7
Contig_62341	pfam01055	Glyco_hydro_31,	1,5	6,0	30,6	12,7
Contig_214946	pfam01074	Glyco_hydro_38,	22,7	15,2	0,0	12,6
Contig_568148	pfam08306	Glyco_hydro_98M,	26,8	11,1	0,0	12,6
Contig_177038	pfam00295	Glyco_hydro_28,	12,2	25,7	0,0	12,6
Contig_481180	pfam03200	Glyco_hydro_63,	19,8	8,7	9,3	12,6
Contig_209037	pfam03198	Glyco_hydro_72,	11,2	24,6	1,9	12,5
Contig_19806	pfam00232	Glyco_hydro_1,	0,0	14,9	22,7	12,5
Contig_582147	pfam01630	Glyco_hydro_56,	18,3	15,1	4,2	12,5
Contig_205464	pfam01074	Glyco_hydro_38,	5,6	14,4	17,6	12,5
Contig_320084	pfam00232	Glyco_hydro_1,	9,3	16,7	11,5	12,5
Contig_203167	pfam12899	Glyco_hydro_100,	13,5	12,7	11,3	12,5
Contig_497940	pfam08306	Glyco_hydro_98M,	16,1	21,3	0,0	12,5
Contig_8205	pfam01074	Glyco_hydro_38,	0,0	1,2	36,0	12,4
Contig_174610	pfam00232	Glyco_hydro_1,	8,2	29,0	0,0	12,4
Contig_215129	pfam00251	Glyco_hydro_32N,	16,5	17,7	3,0	12,4
Contig_13040	pfam12899	Glyco_hydro_100,	5,0	1,5	30,7	12,4
Contig_454911	pfam03639	Glyco_hydro_81,	16,9	0,9	19,3	12,4
Contig_145568	pfam08307	Glyco_hydro_98C,	8,5	10,3	18,3	12,4
Contig_457033	pfam12905	Glyco_hydro_101,	9,4	9,9	17,7	12,3
Contig_16493	pfam00759	Glyco_hydro_9,	20,5	16,5	0,0	12,3
Contig_58499	pfam01301	Glyco_hydro_35,	11,6	13,0	12,3	12,3
Contig_303976	pfam03644	Glyco_hydro_85,	16,1	20,7	0,0	12,3
Contig_480812	pfam03644	Glyco_hydro_85,	10,2	19,0	7,6	12,3
Contig_306645	pfam00933	Glyco_hydro_3,	12,9	13,0	10,8	12,2
Contig_435374	pfam00331	Glyco_hydro_10,	4,6	17,4	14,7	12,2
Contig_89840	pfam00331	Glyco_hydro_10,	2,0	4,8	29,8	12,2
Contig_260155	pfam13199	Glyco_hydro_66,	3,3	8,0	25,1	12,2
Contig_247578	pfam00840	Glyco_hydro_7,	4,6	9,4	22,5	12,1
Contig_57758	pfam03065	Glyco_hydro_57,	6,6	5,0	24,8	12,1
Contig_583108	pfam00331	Glyco_hydro_10,	18,5	13,1	4,8	12,1
Contig_141154	pfam02449	Glyco_hydro_42,	4,7	0,0	31,6	12,1
Contig_99556	pfam00840	Glyco_hydro_7,	6,8	0,0	29,5	12,1
Contig_52870	pfam03200	Glyco_hydro_63,	11,3	10,0	14,9	12,1
Contig_23977	pfam01301	Glyco_hydro_35,	6,2	7,5	22,5	12,1
Contig_64976	pfam00704	Glyco_hydro_18,	6,1	11,0	19,1	12,1
Contig_116230	pfam03443	Glyco_hydro_61,	4,2	0,0	31,9	12,1
Contig_4521	pfam00759	Glyco_hydro_9,	19,0	14,6	2,4	12,0
Contig_103014	pfam01301	Glyco_hydro_35,	12,2	9,9	13,8	12,0
Contig_69972	pfam03663	Glyco_hydro_76,	0,0	3,2	32,8	12,0
Contig_124526	pfam01074	Glyco_hydro_38,	18,0	0,0	18,0	12,0
Contig_221172	pfam03200	Glyco_hydro_63,	17,4	18,6	0,0	12,0
Contig_15680	pfam00232	Glyco_hydro_1,	7,7	9,1	19,1	12,0
Contig_59211	pfam01301	Glyco_hydro_35,	12,8	12,4	10,7	12,0
Contig_114738	pfam07745	Glyco_hydro_53,	5,8	1,5	28,6	11,9
Contig_11283	pfam02838	Glyco_hydro_20b,	12,5	12,0	11,3	11,9
Contig_141061	pfam00704	Glyco_hydro_18,	11,9	5,3	18,6	11,9
Contig_181995	pfam03200	Glyco_hydro_63,	13,8	17,3	4,7	11,9
Contig_196547	pfam01532	Glyco_hydro_47,	5,8	5,3	24,6	11,9
Contig_507967	pfam02449	Glyco_hydro_42,	14,1	15,4	6,2	11,9
Contig_145294	pfam00759	Glyco_hydro_9,	8,0	6,2	21,3	11,9
Contig_39506	pfam07748	Glyco_hydro_38C,	7,9	5,5	22,0	11,8
Contig_233766	pfam00759	Glyco_hydro_9,	4,9	13,9	16,6	11,8
Contig_280177	pfam01630	Glyco_hydro_56,	16,0	19,4	0,0	11,8
Contig_110336	pfam01373	Glyco_hydro_14,	8,2	0,3	26,9	11,8
Contig_565398	pfam07488	Glyco_hydro_67M,	8,6	0,0	26,7	11,8
Contig_141119	pfam07971	Glyco_hydro_92,	1,8	8,3	25,1	11,8
Contig_306	pfam12899	Glyco_hydro_100,	10,4	9,5	15,3	11,7

Contig_29586	pfam03632	Glyco_hydro_65m,	5,9	9,8	19,4	11,7
Contig_227733	pfam00295	Glyco_hydro_28,	12,0	15,7	7,4	11,7
Contig_288099	pfam00704	Glyco_hydro_18,	16,7	18,4	0,0	11,7
Contig_26443	pfam01532	Glyco_hydro_47,	11,7	5,4	18,0	11,7
Contig_310749	pfam00332	Glyco_hydro_17,	15,9	19,1	0,0	11,7
Contig_110963	pfam00840	Glyco_hydro_7,	2,0	0,0	32,9	11,7
Contig_266916	pfam01270	Glyco_hydro_8,	6,8	11,2	17,0	11,7
Contig_76739	pfam00295	Glyco_hydro_28,	7,5	11,1	16,3	11,6
Contig_433370	pfam01301	Glyco_hydro_35,	6,9	9,1	18,8	11,6
Contig_568162	pfam02446	Glyco_hydro_77,	16,2	7,0	11,6	11,6
Contig_43647	pfam01915	Glyco_hydro_3_C,	7,9	13,6	13,2	11,6
Contig_117295	pfam01074	Glyco_hydro_38,	2,3	2,4	30,0	11,6
Contig_576480	pfam00840	Glyco_hydro_7,	23,7	9,0	2,0	11,6
Contig_406590	pfam01074	Glyco_hydro_38,	15,4	14,2	5,1	11,6
Contig_332404	pfam03200	Glyco_hydro_63,	7,6	10,2	16,8	11,5
Contig_52662	pfam03065	Glyco_hydro_57,	3,9	2,7	28,0	11,5
Contig_213693	pfam08306	Glyco_hydro_98M,	10,1	19,5	5,0	11,5
Contig_128095	pfam00704	Glyco_hydro_18,	5,2	11,1	18,3	11,5
Contig_142526	pfam07488	Glyco_hydro_67M,	2,8	8,8	22,8	11,5
Contig_95944	pfam03663	Glyco_hydro_76,	5,2	0,0	29,2	11,5
Contig_11039	pfam00251	Glyco_hydro_32N,	5,2	9,5	19,7	11,5
Contig_350308	pfam00933	Glyco_hydro_3,	17,8	16,6	0,0	11,5
Contig_38531	pfam02057	Glyco_hydro_59,	6,4	0,0	27,9	11,4
Contig_190712	pfam01074	Glyco_hydro_38,	19,1	9,0	6,2	11,4
Contig_434238	pfam00704	Glyco_hydro_18,	3,3	3,9	27,1	11,4
Contig_19364	pfam07477	Glyco_hydro_67C,	4,4	4,3	25,6	11,4
Contig_280004	pfam12899	Glyco_hydro_100,	6,4	23,9	3,9	11,4
Contig_73415	pfam00703	Glyco_hydro_2,	13,7	13,2	7,2	11,4
Contig_40421	pfam02837	Glyco_hydro_2_N,	10,2	1,4	22,6	11,4
Contig_469121	pfam02011	Glyco_hydro_48,	28,9	5,2	0,0	11,4
Contig_138582	pfam04616	Glyco_hydro_43,	13,9	17,4	2,8	11,4
Contig_448118	pfam01532	Glyco_hydro_47,	14,4	16,1	3,6	11,4
Contig_74854	pfam00331	Glyco_hydro_10,	5,3	6,9	21,8	11,3
Contig_233076	pfam07488	Glyco_hydro_67M,	0,0	10,5	23,4	11,3
Contig_11337	pfam00840	Glyco_hydro_7,	2,7	10,7	20,4	11,3
Contig_118950	pfam00759	Glyco_hydro_9,	5,0	12,6	16,2	11,3
Contig_35369	pfam02057	Glyco_hydro_59,	5,2	5,5	22,9	11,2
Contig_28662	pfam01301	Glyco_hydro_35,	3,3	3,3	27,1	11,2
Contig_132796	pfam00759	Glyco_hydro_9,	1,1	0,0	32,6	11,2
Contig_468043	pfam13199	Glyco_hydro_66,	8,5	20,7	4,4	11,2
Contig_393339	pfam01055	Glyco_hydro_31,	10,3	11,8	11,5	11,2
Contig_8315	pfam02449	Glyco_hydro_42,	2,4	16,0	15,1	11,2
Contig_7950	pfam02446	Glyco_hydro_77,	6,2	0,0	27,3	11,2
Contig_30026	pfam08532	Glyco_hydro_42M,	0,0	0,0	33,5	11,2
Contig_178456	pfam01055	Glyco_hydro_31,	2,0	7,1	24,5	11,2
Contig_48116	pfam01532	Glyco_hydro_47,	12,1	0,0	21,3	11,1
Contig_60613	pfam00840	Glyco_hydro_7,	3,3	0,8	29,3	11,1
Contig_411456	pfam08532	Glyco_hydro_42M,	11,6	16,5	5,3	11,1
Contig_288631	pfam01301	Glyco_hydro_35,	11,5	21,9	0,0	11,1
Contig_111792	pfam07971	Glyco_hydro_92,	0,0	2,5	30,9	11,1
Contig_148834	pfam01055	Glyco_hydro_31,	11,6	12,5	9,1	11,1
Contig_133061	pfam00457	Glyco_hydro_11,	5,4	8,6	19,2	11,1
Contig_99776	pfam03659	Glyco_hydro_71,	9,4	0,0	23,7	11,0
Contig_12392	pfam00840	Glyco_hydro_7,	11,7	9,5	11,9	11,0
Contig_217492	pfam12899	Glyco_hydro_100,	5,8	21,0	6,2	11,0
Contig_52868	pfam03648	Glyco_hydro_67N,	7,7	0,0	25,3	11,0
Contig_65066	pfam07748	Glyco_hydro_38C,	0,0	12,1	21,0	11,0
Contig_40588	pfam01074	Glyco_hydro_38,	0,0	1,4	31,6	11,0
Contig_67610	pfam02836	Glyco_hydro_2_C,	7,8	7,2	17,9	11,0
Contig_109848	pfam03198	Glyco_hydro_72,	7,2	11,3	14,4	11,0
Contig_229431	pfam01055	Glyco_hydro_31,	9,0	13,9	9,8	10,9
Contig_229931	pfam03663	Glyco_hydro_76,	3,1	7,1	22,6	10,9
Contig_289982	pfam02836	Glyco_hydro_2_C,	14,7	5,8	12,1	10,8
Contig_452856	pfam13199	Glyco_hydro_66,	10,9	5,3	16,3	10,8
Contig_56477	pfam02011	Glyco_hydro_48,	3,3	2,8	26,3	10,8
Contig_133873	pfam00840	Glyco_hydro_7,	11,3	0,0	21,1	10,8
Contig_211418	pfam00251	Glyco_hydro_32N,	15,0	17,3	0,0	10,8
Contig_421250	pfam11790	Glyco_hydro_cc,	8,6	10,3	13,4	10,7
Contig_99089	pfam00722	Glyco_hydro_16,	8,5	6,5	17,2	10,7
Contig_3179	pfam03648	Glyco_hydro_67N,	9,7	6,9	15,6	10,7
Contig_51160	pfam02156	Glyco_hydro_26,	4,5	0,6	27,1	10,7

Contig_42823	pfam03200	Glyco_hydro_63,	8,3	12,9	10,9	10,7
Contig_427039	pfam00295	Glyco_hydro_28,	8,4	23,7	0,0	10,7
Contig_553270	pfam03443	Glyco_hydro_61,	6,1	14,6	11,3	10,7
Contig_238998	pfam07748	Glyco_hydro_38C,	11,3	8,6	12,1	10,7
Contig_423745	pfam03639	Glyco_hydro_81,	9,3	6,3	16,3	10,6
Contig_242952	pfam00704	Glyco_hydro_18,	12,3	7,9	11,7	10,6
Contig_4881	pfam00933	Glyco_hydro_3,	11,6	16,1	4,2	10,6
Contig_100971	pfam03200	Glyco_hydro_63,	13,1	1,9	17,0	10,6
Contig_13370	pfam01532	Glyco_hydro_47,	7,9	0,8	23,2	10,6
Contig_353644	pfam01055	Glyco_hydro_31,	4,7	3,8	23,4	10,6
Contig_388094	pfam01229	Glyco_hydro_39,	18,2	13,6	0,0	10,6
Contig_420780	pfam03644	Glyco_hydro_85,	17,8	10,8	3,2	10,6
Contig_99899	pfam00722	Glyco_hydro_16,	4,3	6,0	21,4	10,6
Contig_51761	pfam01055	Glyco_hydro_31,	0,0	3,9	27,8	10,6
Contig_39581	pfam01055	Glyco_hydro_31,	3,0	1,8	26,7	10,5
Contig_512871	pfam00295	Glyco_hydro_28,	17,9	13,7	0,0	10,5
Contig_202223	pfam02324	Glyco_hydro_70,	6,4	6,3	18,8	10,5
Contig_5813	pfam00759	Glyco_hydro_9,	9,2	1,9	20,4	10,5
Contig_14046	pfam00840	Glyco_hydro_7,	10,1	2,3	19,1	10,5
Contig_504024	pfam03065	Glyco_hydro_57,	12,6	3,0	15,8	10,5
Contig_101664	pfam01055	Glyco_hydro_31,	8,2	9,5	13,7	10,5
Contig_151786	pfam01229	Glyco_hydro_39,	31,3	0,0	0,0	10,4
Contig_187561	pfam03200	Glyco_hydro_63,	17,7	6,9	6,6	10,4
Contig_333129	pfam03443	Glyco_hydro_61,	3,3	13,5	14,2	10,3
Contig_343892	pfam10566	Glyco_hydro_97,	7,0	4,1	19,9	10,3
Contig_180171	pfam03639	Glyco_hydro_81,	10,8	17,9	2,2	10,3
Contig_309333	pfam00295	Glyco_hydro_28,	7,8	1,6	21,6	10,3
Contig_279717	pfam00759	Glyco_hydro_9,	10,9	10,5	9,5	10,3
Contig_29743	pfam01373	Glyco_hydro_14,	8,1	5,1	17,7	10,3
Contig_145196	pfam03664	Glyco_hydro_62,	13,1	0,0	17,8	10,3
Contig_164952	pfam10566	Glyco_hydro_97,	4,6	9,0	17,2	10,3
Contig_107782	pfam00295	Glyco_hydro_28,	10,6	1,9	18,3	10,3
Contig_116399	pfam01532	Glyco_hydro_47,	1,3	0,0	29,5	10,3
Contig_269462	pfam02324	Glyco_hydro_70,	10,8	8,4	11,5	10,2
Contig_222433	pfam03512	Glyco_hydro_52,	10,5	0,5	19,6	10,2
Contig_15414	pfam00251	Glyco_hydro_32N,	0,0	4,1	26,5	10,2
Contig_97356	pfam00728	Glyco_hydro_20,	4,8	1,9	24,0	10,2
Contig_142547	pfam00840	Glyco_hydro_7,	0,0	11,5	19,1	10,2
Contig_105016	pfam02837	Glyco_hydro_2_N,	6,5	0,7	23,4	10,2
Contig_143245	pfam03639	Glyco_hydro_81,	8,8	8,7	13,2	10,2
Contig_256188	pfam00251	Glyco_hydro_32N,	9,5	14,0	7,2	10,2
Contig_474791	pfam03200	Glyco_hydro_63,	22,3	8,2	0,0	10,2
Contig_106818	pfam00295	Glyco_hydro_28,	6,2	7,8	16,6	10,2
Contig_105333	pfam03662	Glyco_hydro_79n,	4,0	6,7	19,7	10,1
Contig_425976	pfam00722	Glyco_hydro_16,	14,1	16,2	0,0	10,1
Contig_178885	pfam02446	Glyco_hydro_77,	18,0	12,3	0,0	10,1
Contig_42870	pfam00840	Glyco_hydro_7,	8,7	0,0	21,6	10,1
Contig_115088	pfam03639	Glyco_hydro_81,	0,0	9,4	20,7	10,0
Contig_409191	pfam00759	Glyco_hydro_9,	1,3	4,4	24,4	10,0
Contig_567033	pfam03718	Glyco_hydro_49,	3,0	8,2	18,9	10,0
Contig_146867	pfam03198	Glyco_hydro_72,	3,6	7,3	19,3	10,0
Contig_131525	pfam01055	Glyco_hydro_31,	6,1	3,7	20,3	10,0
Contig_194792	pfam11975	Glyco_hydro_4C,	3,2	16,1	10,7	10,0
Contig_134982	pfam00295	Glyco_hydro_28,	3,8	0,0	26,2	10,0
Contig_464524	pfam00722	Glyco_hydro_16,	17,7	12,3	0,0	10,0
Contig_29316	pfam00251	Glyco_hydro_32N,	1,4	1,6	27,0	10,0
Contig_6476	pfam00332	Glyco_hydro_17,	1,7	5,7	22,5	10,0
Contig_70659	pfam03718	Glyco_hydro_49,	0,0	0,6	29,3	10,0
Contig_120302	pfam03065	Glyco_hydro_57,	1,6	0,0	28,3	10,0
Contig_280124	pfam00759	Glyco_hydro_9,	10,5	12,9	6,4	9,9
Contig_345967	pfam01229	Glyco_hydro_39,	14,9	14,8	0,0	9,9
Contig_176254	pfam00232	Glyco_hydro_1,	11,4	2,0	16,4	9,9
Contig_227115	pfam12899	Glyco_hydro_100,	2,9	10,5	16,3	9,9
Contig_235187	pfam03198	Glyco_hydro_72,	9,0	16,2	4,4	9,9
Contig_29971	pfam03633	Glyco_hydro_65C,	3,8	1,4	24,5	9,9
Contig_265309	pfam00457	Glyco_hydro_11,	12,1	14,6	3,0	9,9
Contig_104202	pfam01301	Glyco_hydro_35,	5,7	3,0	21,0	9,9
Contig_121110	pfam12899	Glyco_hydro_100,	2,6	8,1	19,0	9,9
Contig_15687	pfam00704	Glyco_hydro_18,	12,7	4,0	12,9	9,9
Contig_81729	pfam03644	Glyco_hydro_85,	10,4	11,7	7,5	9,9
Contig_4574	pfam02446	Glyco_hydro_77,	15,1	1,8	12,6	9,8

Contig_109097	pfam00251	Glyco_hydro_32N,	8,5	2,9	18,1	9,8
Contig_215365	pfam02449	Glyco_hydro_42,	11,7	12,0	5,8	9,8
Contig_37404	pfam00295	Glyco_hydro_28,	2,0	4,4	23,1	9,8
Contig_299152	pfam01301	Glyco_hydro_35,	9,2	17,8	2,5	9,8
Contig_261410	pfam03639	Glyco_hydro_81,	12,1	13,7	3,5	9,8
Contig_22200	pfam12905	Glyco_hydro_101,	8,5	5,8	15,1	9,8
Contig_206175	pfam13199	Glyco_hydro_66,	6,8	5,7	16,8	9,8
Contig_21291	pfam01074	Glyco_hydro_38,	10,7	6,6	12,0	9,8
Contig_272151	pfam00232	Glyco_hydro_1,	2,8	12,5	13,9	9,7
Contig_62690	pfam03065	Glyco_hydro_57,	4,9	0,0	24,3	9,7
Contig_55044	pfam01532	Glyco_hydro_47,	0,0	2,7	26,5	9,7
Contig_13703	pfam03200	Glyco_hydro_63,	1,9	1,9	25,2	9,7
Contig_299479	pfam02449	Glyco_hydro_42,	1,5	27,6	0,0	9,7
Contig_8320	pfam07971	Glyco_hydro_92,	0,0	0,0	29,0	9,7
Contig_139325	pfam02324	Glyco_hydro_70,	2,6	0,0	26,2	9,6
Contig_48403	pfam00704	Glyco_hydro_18,	7,3	0,0	21,5	9,6
Contig_105019	pfam02324	Glyco_hydro_70,	1,4	2,4	25,0	9,6
Contig_484315	pfam03512	Glyco_hydro_52,	7,3	21,4	0,0	9,6
Contig_261180	pfam02324	Glyco_hydro_70,	7,2	6,5	15,0	9,6
Contig_69891	pfam00722	Glyco_hydro_16,	14,8	6,2	7,6	9,5
Contig_426616	pfam00232	Glyco_hydro_1,	4,9	10,6	13,1	9,5
Contig_3004	pfam03200	Glyco_hydro_63,	8,2	0,0	20,4	9,5
Contig_528023	pfam11790	Glyco_hydro_cc,	24,4	1,1	3,1	9,5
Contig_111534	pfam01074	Glyco_hydro_38,	0,0	4,1	24,5	9,5
Contig_141541	pfam04616	Glyco_hydro_43,	4,9	6,9	16,8	9,5
Contig_29417	pfam02449	Glyco_hydro_42,	4,9	20,6	3,1	9,5
Contig_15796	pfam03659	Glyco_hydro_71,	0,5	3,8	24,2	9,5
Contig_117001	pfam13199	Glyco_hydro_66,	7,0	7,0	14,5	9,5
Contig_339921	pfam01229	Glyco_hydro_39,	8,1	6,3	14,1	9,5
Contig_328863	pfam03639	Glyco_hydro_81,	5,8	7,9	14,6	9,5
Contig_184589	pfam01373	Glyco_hydro_14,	6,7	19,2	2,5	9,5
Contig_1813	pfam02324	Glyco_hydro_70,	3,7	1,7	23,0	9,5
Contig_22287	pfam07971	Glyco_hydro_92,	6,0	8,4	14,0	9,4
Contig_117203	pfam00704	Glyco_hydro_18,	3,2	4,9	20,2	9,4
Contig_377198	pfam00723	Glyco_hydro_15,	13,0	11,0	4,3	9,4
Contig_181223	pfam03200	Glyco_hydro_63,	9,0	15,6	3,7	9,4
Contig_350459	pfam00232	Glyco_hydro_1,	0,0	10,9	17,4	9,4
Contig_365539	pfam01301	Glyco_hydro_35,	7,8	5,9	14,5	9,4
Contig_94004	pfam00728	Glyco_hydro_20,	3,9	9,4	14,9	9,4
Contig_60095	pfam03718	Glyco_hydro_49,	17,8	3,3	6,9	9,3
Contig_58634	pfam00251	Glyco_hydro_32N,	25,0	3,0	0,0	9,3
Contig_139270	pfam10566	Glyco_hydro_97,	4,1	5,2	18,6	9,3
Contig_37855	pfam00933	Glyco_hydro_3,	1,1	5,2	21,5	9,3
Contig_107521	pfam07971	Glyco_hydro_92,	5,8	7,5	14,5	9,3
Contig_222628	pfam00295	Glyco_hydro_28,	6,9	20,8	0,0	9,3
Contig_20160	pfam02156	Glyco_hydro_26,	0,0	4,0	23,7	9,2
Contig_131216	pfam12899	Glyco_hydro_100,	9,3	0,0	18,4	9,2
Contig_213599	pfam03659	Glyco_hydro_71,	1,2	14,4	12,0	9,2
Contig_151644	pfam01532	Glyco_hydro_47,	0,0	10,4	17,2	9,2
Contig_471241	pfam00331	Glyco_hydro_10,	23,4	4,3	0,0	9,2
Contig_184227	pfam02055	Glyco_hydro_30,	9,7	5,8	12,1	9,2
Contig_3619	pfam02324	Glyco_hydro_70,	0,0	7,9	19,7	9,2
Contig_43607	pfam02446	Glyco_hydro_77,	8,0	4,8	14,8	9,2
Contig_197976	pfam01074	Glyco_hydro_38,	7,0	3,3	17,3	9,2
Contig_499510	pfam03663	Glyco_hydro_76,	13,5	6,4	7,6	9,2
Contig_288386	pfam03200	Glyco_hydro_63,	12,9	12,5	2,1	9,1
Contig_470163	pfam01301	Glyco_hydro_35,	8,0	6,0	13,3	9,1
Contig_391145	pfam00933	Glyco_hydro_3,	7,2	3,9	16,2	9,1
Contig_503275	pfam00728	Glyco_hydro_20,	7,4	8,9	11,1	9,1
Contig_375087	pfam00759	Glyco_hydro_9,	9,1	18,3	0,0	9,1
Contig_56761	pfam13199	Glyco_hydro_66,	2,1	1,9	23,3	9,1
Contig_308436	pfam04616	Glyco_hydro_43,	7,1	2,7	17,5	9,1
Contig_529155	pfam02836	Glyco_hydro_2_C,	6,6	2,9	17,8	9,1
Contig_106966	pfam07477	Glyco_hydro_67C,	2,4	4,3	20,6	9,1
Contig_382681	pfam00331	Glyco_hydro_10,	15,4	11,8	0,0	9,1
Contig_104242	pfam01670	Glyco_hydro_12,	7,6	11,9	7,6	9,1
Contig_370442	pfam01055	Glyco_hydro_31,	12,3	14,9	0,0	9,0
Contig_188451	pfam07748	Glyco_hydro_38C,	9,7	15,6	1,8	9,0
Contig_15187	pfam03639	Glyco_hydro_81,	4,7	2,2	20,3	9,0
Contig_85496	pfam02836	Glyco_hydro_2_C,	4,0	2,1	21,1	9,0
Contig_60102	pfam07335	Glyco_hydro_75,	10,0	1,1	16,0	9,0

Contig_162395	pfam07971	Glyco_hydro_92,	13,2	11,6	2,2	9,0
Contig_131008	pfam03632	Glyco_hydro_65m,	4,7	6,6	15,6	9,0
Contig_350891	pfam07745	Glyco_hydro_53,	15,3	11,6	0,0	9,0
Contig_85850	pfam04616	Glyco_hydro_43,	11,7	3,0	12,2	9,0
Contig_109675	pfam01055	Glyco_hydro_31,	4,7	1,8	20,4	9,0
Contig_194512	pfam00332	Glyco_hydro_17,	7,2	16,5	3,2	8,9
Contig_114059	pfam00759	Glyco_hydro_9,	0,0	3,1	23,8	8,9
Contig_74582	pfam01532	Glyco_hydro_47,	5,9	0,0	20,9	8,9
Contig_142557	pfam00331	Glyco_hydro_10,	1,7	0,0	25,1	8,9
Contig_239513	pfam03648	Glyco_hydro_67N,	19,7	7,0	0,0	8,9
Contig_314711	pfam00232	Glyco_hydro_1,	13,4	13,3	0,0	8,9
Contig_32706	pfam00295	Glyco_hydro_28,	2,1	5,8	18,7	8,9
Contig_288379	pfam04616	Glyco_hydro_43,	4,2	4,6	17,8	8,9
Contig_224015	pfam07748	Glyco_hydro_38C,	10,9	12,3	3,4	8,9
Contig_54253	pfam03718	Glyco_hydro_49,	2,0	4,3	20,3	8,9
Contig_473983	pfam00295	Glyco_hydro_28,	11,9	7,2	7,4	8,9
Contig_34075	pfam02836	Glyco_hydro_2_C,	0,0	7,0	19,5	8,8
Contig_120646	pfam03200	Glyco_hydro_63,	8,6	0,0	17,9	8,8
Contig_503074	pfam00759	Glyco_hydro_9,	18,5	8,0	0,0	8,8
Contig_66840	pfam03200	Glyco_hydro_63,	4,5	1,6	20,3	8,8
Contig_39452	pfam03659	Glyco_hydro_71,	4,4	8,5	13,6	8,8
Contig_62051	pfam03198	Glyco_hydro_72,	4,7	0,6	21,1	8,8
Contig_104203	pfam01532	Glyco_hydro_47,	3,6	7,3	15,5	8,8
Contig_299278	pfam02837	Glyco_hydro_2_N,	7,0	10,4	9,0	8,8
Contig_134004	pfam03200	Glyco_hydro_63,	8,2	18,2	0,0	8,8
Contig_41956	pfam03639	Glyco_hydro_81,	6,4	0,8	19,1	8,8
Contig_471967	pfam00331	Glyco_hydro_10,	13,8	12,5	0,0	8,8
Contig_49428	pfam03200	Glyco_hydro_63,	0,7	2,2	23,4	8,8
Contig_112382	pfam00723	Glyco_hydro_15,	0,0	1,7	24,6	8,8
Contig_133433	pfam12899	Glyco_hydro_100,	3,9	3,1	19,3	8,8
Contig_509840	pfam02011	Glyco_hydro_48,	16,4	9,9	0,0	8,7
Contig_121609	pfam03663	Glyco_hydro_76,	0,0	1,0	25,1	8,7
Contig_51400	pfam02055	Glyco_hydro_30,	0,0	6,6	19,5	8,7
Contig_347074	pfam07488	Glyco_hydro_67M,	2,3	10,3	13,5	8,7
Contig_156213	pfam00232	Glyco_hydro_1,	5,0	8,9	12,2	8,7
Contig_190350	pfam01301	Glyco_hydro_35,	1,5	10,9	13,7	8,7
Contig_315501	pfam00232	Glyco_hydro_1,	11,4	2,9	11,8	8,7
Contig_21233	pfam03065	Glyco_hydro_57,	14,3	0,0	11,8	8,7
Contig_578253	pfam12899	Glyco_hydro_100,	6,0	0,0	20,0	8,7
Contig_206262	pfam01074	Glyco_hydro_38,	9,5	15,0	1,5	8,7
Contig_279087	pfam02435	Glyco_hydro_68,	0,0	2,8	23,2	8,7
Contig_10841	pfam00332	Glyco_hydro_17,	8,4	0,0	17,4	8,6
Contig_58726	pfam00704	Glyco_hydro_18,	1,0	1,9	23,0	8,6
Contig_338545	pfam00251	Glyco_hydro_32N,	0,0	4,0	21,9	8,6
Contig_169304	pfam00295	Glyco_hydro_28,	11,3	14,5	0,0	8,6
Contig_80207	pfam03664	Glyco_hydro_62,	0,0	3,6	22,2	8,6
Contig_575107	pfam03065	Glyco_hydro_57,	18,5	7,3	0,0	8,6
Contig_519259	pfam02011	Glyco_hydro_48,	20,6	1,7	3,4	8,6
Contig_148847	pfam01055	Glyco_hydro_31,	15,0	0,0	10,7	8,6
Contig_30923	pfam00232	Glyco_hydro_1,	7,6	5,0	13,1	8,6
Contig_134366	pfam00704	Glyco_hydro_18,	10,4	13,1	2,1	8,5
Contig_148499	pfam02837	Glyco_hydro_2_N,	13,2	0,0	12,4	8,5
Contig_462880	pfam00251	Glyco_hydro_32N,	4,6	0,6	20,4	8,5
Contig_8717	pfam01374	Glyco_hydro_46,	9,9	5,4	10,2	8,5
Contig_204148	pfam01055	Glyco_hydro_31,	12,2	3,3	10,0	8,5
Contig_544502	pfam01532	Glyco_hydro_47,	22,0	0,0	3,4	8,5
Contig_59866	pfam03512	Glyco_hydro_52,	1,9	9,2	14,3	8,5
Contig_828	pfam01915	Glyco_hydro_3_C,	2,8	6,2	16,3	8,4
Contig_501881	pfam02435	Glyco_hydro_68,	10,5	14,8	0,0	8,4
Contig_153538	pfam02057	Glyco_hydro_59,	0,0	0,0	25,3	8,4
Contig_575439	pfam03662	Glyco_hydro_79n,	18,3	1,6	5,3	8,4
Contig_21302	pfam00722	Glyco_hydro_16,	4,1	0,8	20,3	8,4
Contig_109600	pfam00332	Glyco_hydro_17,	1,4	2,6	21,2	8,4
Contig_563102	pfam07477	Glyco_hydro_67C,	24,5	0,7	0,0	8,4
Contig_187675	pfam03662	Glyco_hydro_79n,	2,2	23,0	0,0	8,4
Contig_580810	pfam00457	Glyco_hydro_11,	9,9	10,8	4,5	8,4
Contig_544319	pfam01301	Glyco_hydro_35,	18,4	6,8	0,0	8,4
Contig_19685	pfam00704	Glyco_hydro_18,	1,2	2,2	21,7	8,4
Contig_118127	pfam00759	Glyco_hydro_9,	2,4	2,1	20,6	8,4
Contig_52688	pfam00704	Glyco_hydro_18,	4,2	0,0	20,9	8,4
Contig_469668	pfam00704	Glyco_hydro_18,	14,7	9,1	1,3	8,4

Contig_99623	pfam00759	Glyco_hydro_9,	4,8	0,0	20,2	8,3
Contig_914	pfam01630	Glyco_hydro_56,	9,3	1,9	13,7	8,3
Contig_366228	pfam01532	Glyco_hydro_47,	9,6	12,8	2,6	8,3
Contig_428050	pfam03662	Glyco_hydro_79n,	12,2	12,8	0,0	8,3
Contig_11858	pfam01670	Glyco_hydro_12,	5,4	1,6	17,9	8,3
Contig_430971	pfam01532	Glyco_hydro_47,	8,8	12,9	3,2	8,3
Contig_425600	pfam03644	Glyco_hydro_85,	0,0	2,1	22,8	8,3
Contig_465992	pfam04616	Glyco_hydro_43,	4,9	0,8	19,1	8,3
Contig_294445	pfam00933	Glyco_hydro_3,	6,0	16,3	2,5	8,3
Contig_390183	pfam01915	Glyco_hydro_3_C,	10,9	7,1	6,8	8,3
Contig_106894	pfam03663	Glyco_hydro_76,	0,0	3,9	20,9	8,3
Contig_25804	pfam02446	Glyco_hydro_77,	3,3	0,0	21,5	8,3
Contig_49933	pfam00704	Glyco_hydro_18,	3,7	0,6	20,6	8,3
Contig_220722	pfam01074	Glyco_hydro_38,	6,7	18,1	0,0	8,2
Contig_36491	pfam10566	Glyco_hydro_97,	1,5	0,5	22,8	8,2
Contig_1893	pfam01074	Glyco_hydro_38,	2,9	1,0	20,8	8,2
Contig_297646	pfam01055	Glyco_hydro_31,	14,5	8,2	2,0	8,2
Contig_301884	pfam00295	Glyco_hydro_28,	4,4	20,2	0,0	8,2
Contig_216638	pfam02011	Glyco_hydro_48,	11,4	6,5	6,7	8,2
Contig_106356	pfam00295	Glyco_hydro_28,	15,8	0,2	8,6	8,2
Contig_140356	pfam00704	Glyco_hydro_18,	12,1	0,3	12,2	8,2
Contig_472903	pfam08306	Glyco_hydro_98M,	6,1	5,3	13,1	8,2
Contig_505166	pfam00251	Glyco_hydro_32N,	5,7	4,6	14,2	8,2
Contig_544169	pfam01301	Glyco_hydro_35,	10,9	13,6	0,0	8,2
Contig_43507	pfam02449	Glyco_hydro_42,	2,4	0,7	21,3	8,2
Contig_221206	pfam00251	Glyco_hydro_32N,	10,7	13,8	0,0	8,2
Contig_147881	pfam04616	Glyco_hydro_43,	4,8	3,7	16,1	8,2
Contig_579392	pfam03659	Glyco_hydro_71,	9,7	3,5	11,2	8,2
Contig_174094	pfam02057	Glyco_hydro_59,	5,2	12,8	6,4	8,1
Contig_67769	pfam01074	Glyco_hydro_38,	2,3	5,1	17,1	8,1
Contig_472872	pfam10566	Glyco_hydro_97,	11,6	12,9	0,0	8,1
Contig_567893	pfam03644	Glyco_hydro_85,	11,1	13,3	0,0	8,1
Contig_68610	pfam04616	Glyco_hydro_43,	7,8	1,2	15,4	8,1
Contig_136418	pfam00728	Glyco_hydro_20,	0,9	2,3	21,1	8,1
Contig_59588	pfam03659	Glyco_hydro_71,	7,0	0,0	17,4	8,1
Contig_112809	pfam00759	Glyco_hydro_9,	6,5	9,8	8,0	8,1
Contig_350987	pfam10566	Glyco_hydro_97,	17,0	7,3	0,0	8,1
Contig_98033	pfam00728	Glyco_hydro_20,	3,3	0,0	21,0	8,1
Contig_495094	pfam02446	Glyco_hydro_77,	17,3	7,0	0,0	8,1
Contig_448171	pfam00759	Glyco_hydro_9,	8,0	11,8	4,5	8,1
Contig_121234	pfam02055	Glyco_hydro_30,	1,3	0,0	22,9	8,1
Contig_479962	pfam00251	Glyco_hydro_32N,	7,9	16,3	0,0	8,1
Contig_5640	pfam03065	Glyco_hydro_57,	6,8	5,3	12,1	8,1
Contig_58431	pfam03659	Glyco_hydro_71,	0,9	0,0	23,3	8,1
Contig_76844	pfam01055	Glyco_hydro_31,	0,0	3,2	21,0	8,1
Contig_128850	pfam01229	Glyco_hydro_39,	3,5	0,0	20,6	8,0
Contig_17154	pfam03200	Glyco_hydro_63,	5,7	4,3	14,2	8,0
Contig_339872	pfam03659	Glyco_hydro_71,	17,3	6,7	0,0	8,0
Contig_449622	pfam03644	Glyco_hydro_85,	22,0	2,1	0,0	8,0
Contig_298439	pfam00759	Glyco_hydro_9,	0,8	15,3	7,9	8,0
Contig_204578	pfam01074	Glyco_hydro_38,	9,5	10,1	4,3	8,0
Contig_277290	pfam03200	Glyco_hydro_63,	4,8	19,2	0,0	8,0
Contig_23311	pfam04616	Glyco_hydro_43,	0,0	2,2	21,7	8,0
Contig_261265	pfam02324	Glyco_hydro_70,	12,8	7,2	4,0	8,0
Contig_186791	pfam12899	Glyco_hydro_100,	6,7	17,2	0,0	8,0
Contig_2960	pfam03633	Glyco_hydro_65C,	7,4	7,4	9,1	8,0
Contig_214927	pfam03639	Glyco_hydro_81,	0,0	5,8	18,1	8,0
Contig_547619	pfam01055	Glyco_hydro_31,	23,9	0,0	0,0	8,0
Contig_19214	pfam02837	Glyco_hydro_2_N,	4,0	6,5	13,3	8,0
Contig_420270	pfam04616	Glyco_hydro_43,	8,1	6,1	9,6	7,9
Contig_76443	pfam02156	Glyco_hydro_26,	7,8	13,0	3,0	7,9
Contig_118481	pfam03512	Glyco_hydro_52,	1,9	1,1	20,8	7,9
Contig_236045	pfam02446	Glyco_hydro_77,	6,7	17,1	0,0	7,9
Contig_158155	pfam12899	Glyco_hydro_100,	8,1	12,1	3,6	7,9
Contig_181478	pfam11790	Glyco_hydro_cc,	9,4	14,4	0,0	7,9
Contig_308871	pfam04616	Glyco_hydro_43,	18,9	4,9	0,0	7,9
Contig_10324	pfam01301	Glyco_hydro_35,	2,4	5,8	15,4	7,9
Contig_58683	pfam02055	Glyco_hydro_30,	0,0	8,9	14,8	7,9
Contig_45784	pfam03200	Glyco_hydro_63,	2,6	1,6	19,5	7,9
Contig_89208	pfam00295	Glyco_hydro_28,	11,6	12,0	0,0	7,9
Contig_536259	pfam12899	Glyco_hydro_100,	11,1	12,5	0,0	7,9

Contig_136457	pfam04616	Glyco_hydro_43,	5,4	4,6	13,6	7,9
Contig_333710	pfam00332	Glyco_hydro_17,	8,3	11,1	4,2	7,9
Contig_457180	pfam01373	Glyco_hydro_14,	8,0	15,5	0,0	7,9
Contig_517707	pfam00722	Glyco_hydro_16,	7,4	8,3	7,9	7,8
Contig_407346	pfam02011	Glyco_hydro_48,	15,1	8,4	0,0	7,8
Contig_61015	pfam01055	Glyco_hydro_31,	3,8	6,9	12,8	7,8
Contig_107477	pfam12891	Glyco_hydro_44,	8,1	1,5	13,9	7,8
Contig_471287	pfam03664	Glyco_hydro_62,	20,5	2,9	0,0	7,8
Contig_104802	pfam07488	Glyco_hydro_67M,	2,9	4,4	16,1	7,8
Contig_56195	pfam12899	Glyco_hydro_100,	7,5	4,5	11,3	7,8
Contig_497148	pfam10566	Glyco_hydro_97,	9,3	14,1	0,0	7,8
Contig_12712	pfam00759	Glyco_hydro_9,	6,5	10,1	6,7	7,8
Contig_32450	pfam07745	Glyco_hydro_53,	9,5	6,1	7,8	7,8
Contig_550764	pfam07745	Glyco_hydro_53,	12,4	0,0	11,0	7,8
Contig_260876	pfam00933	Glyco_hydro_3,	17,9	5,4	0,0	7,8
Contig_27023	pfam03200	Glyco_hydro_63,	1,3	2,0	19,9	7,8
Contig_200209	pfam03200	Glyco_hydro_63,	9,9	13,3	0,0	7,8
Contig_217876	pfam02446	Glyco_hydro_77,	6,1	7,4	9,7	7,7
Contig_106249	pfam00840	Glyco_hydro_7,	0,0	6,7	16,5	7,7
Contig_67831	pfam02435	Glyco_hydro_68,	3,3	3,0	16,8	7,7
Contig_468065	pfam01301	Glyco_hydro_35,	5,5	10,2	7,4	7,7
Contig_476450	pfam02446	Glyco_hydro_77,	3,5	9,2	10,3	7,7
Contig_8886	pfam10566	Glyco_hydro_97,	0,0	10,9	12,1	7,7
Contig_43177	pfam00723	Glyco_hydro_15,	0,0	1,3	21,7	7,7
Contig_499609	pfam01532	Glyco_hydro_47,	4,3	3,7	15,1	7,7
Contig_546339	pfam07745	Glyco_hydro_53,	10,8	4,2	8,0	7,7
Contig_433272	pfam00722	Glyco_hydro_16,	9,9	13,1	0,0	7,7
Contig_20986	pfam03718	Glyco_hydro_49,	11,6	5,6	5,8	7,7
Contig_361787	pfam00251	Glyco_hydro_32N,	8,7	8,1	6,2	7,6
Contig_401673	pfam02836	Glyco_hydro_2_C,	11,0	12,0	0,0	7,6
Contig_47112	pfam13199	Glyco_hydro_66,	1,3	2,3	19,3	7,6
Contig_414671	pfam01373	Glyco_hydro_14,	13,7	2,5	6,8	7,6
Contig_135843	pfam12905	Glyco_hydro_101,	5,0	5,4	12,4	7,6
Contig_42264	pfam02838	Glyco_hydro_20b,	0,0	6,7	16,1	7,6
Contig_578186	pfam00759	Glyco_hydro_9,	11,7	7,8	3,3	7,6
Contig_72220	pfam03200	Glyco_hydro_63,	0,0	0,7	22,1	7,6
Contig_38082	pfam07488	Glyco_hydro_67M,	4,0	1,2	17,5	7,6
Contig_232274	pfam01270	Glyco_hydro_8,	6,2	9,6	6,9	7,6
Contig_55194	pfam01532	Glyco_hydro_47,	0,0	12,8	9,9	7,6
Contig_221384	pfam07477	Glyco_hydro_67C,	2,9	8,8	11,0	7,6
Contig_307712	pfam03200	Glyco_hydro_63,	11,1	11,6	0,0	7,6
Contig_176059	pfam00728	Glyco_hydro_20,	11,5	8,3	2,9	7,6
Contig_248427	pfam08306	Glyco_hydro_98M,	7,5	11,2	4,0	7,5
Contig_181234	pfam13199	Glyco_hydro_66,	8,2	7,7	6,8	7,5
Contig_73978	pfam01630	Glyco_hydro_56,	8,3	6,0	8,3	7,5
Contig_177932	pfam02324	Glyco_hydro_70,	8,8	13,7	0,0	7,5
Contig_40988	pfam00759	Glyco_hydro_9,	0,0	0,0	22,5	7,5
Contig_292224	pfam13199	Glyco_hydro_66,	6,0	9,6	7,0	7,5
Contig_43295	pfam03664	Glyco_hydro_62,	16,6	1,6	4,3	7,5
Contig_102437	pfam00295	Glyco_hydro_28,	1,9	1,6	18,9	7,5
Contig_202444	pfam00331	Glyco_hydro_10,	8,5	5,5	8,5	7,5
Contig_384812	pfam03200	Glyco_hydro_63,	10,5	12,0	0,0	7,5
Contig_276531	pfam07971	Glyco_hydro_92,	4,7	17,8	0,0	7,5
Contig_177059	pfam01532	Glyco_hydro_47,	6,3	16,1	0,0	7,5
Contig_210991	pfam03644	Glyco_hydro_85,	7,5	7,9	7,0	7,5
Contig_18836	pfam01055	Glyco_hydro_31,	0,7	2,4	19,4	7,5
Contig_5196	pfam03198	Glyco_hydro_72,	7,9	0,0	14,4	7,4
Contig_131676	pfam00232	Glyco_hydro_1,	14,1	0,4	7,8	7,4
Contig_47225	pfam01630	Glyco_hydro_56,	8,1	4,2	10,0	7,4
Contig_2372	pfam01301	Glyco_hydro_35,	4,9	2,2	15,2	7,4
Contig_132564	pfam03065	Glyco_hydro_57,	0,0	1,7	20,6	7,4
Contig_423655	pfam00295	Glyco_hydro_28,	8,5	3,2	10,6	7,4
Contig_9692	pfam03065	Glyco_hydro_57,	0,7	4,5	17,0	7,4
Contig_49541	pfam01301	Glyco_hydro_35,	0,9	2,8	18,5	7,4
Contig_74401	pfam12899	Glyco_hydro_100,	10,2	1,8	10,3	7,4
Contig_521149	pfam03200	Glyco_hydro_63,	13,6	8,7	0,0	7,4
Contig_19246	pfam03659	Glyco_hydro_71,	0,0	3,7	18,5	7,4
Contig_41473	pfam02057	Glyco_hydro_59,	4,8	6,8	10,6	7,4
Contig_175726	pfam10566	Glyco_hydro_97,	4,5	14,6	3,2	7,4
Contig_176453	pfam02446	Glyco_hydro_77,	5,8	16,3	0,0	7,4
Contig_1945	pfam03718	Glyco_hydro_49,	0,0	0,7	21,4	7,4

Contig_39578	pfam00704	Glyco_hydro_18,	1,6	0,0	20,5	7,4
Contig_574268	pfam13199	Glyco_hydro_66,	22,1	0,0	0,0	7,4
Contig_294548	pfam04616	Glyco_hydro_43,	8,4	13,7	0,0	7,4
Contig_49166	pfam00704	Glyco_hydro_18,	9,1	1,8	11,2	7,4
Contig_239227	pfam01301	Glyco_hydro_35,	8,1	10,9	3,1	7,3
Contig_38726	pfam00251	Glyco_hydro_32N,	0,0	10,8	11,2	7,3
Contig_338306	pfam00251	Glyco_hydro_32N,	5,3	16,7	0,0	7,3
Contig_176466	pfam02836	Glyco_hydro_2_C,	8,3	12,0	1,7	7,3
Contig_118097	pfam10566	Glyco_hydro_97,	10,1	5,4	6,5	7,3
Contig_109236	pfam02449	Glyco_hydro_42,	2,8	3,8	15,5	7,3
Contig_62336	pfam02838	Glyco_hydro_20b,	7,3	1,0	13,7	7,3
Contig_55096	pfam00759	Glyco_hydro_9,	4,2	0,0	17,7	7,3
Contig_12040	pfam00232	Glyco_hydro_1,	5,7	4,8	11,4	7,3
Contig_578570	pfam03200	Glyco_hydro_63,	0,0	0,0	22,0	7,3
Contig_18547	pfam01270	Glyco_hydro_8,	1,4	2,6	17,9	7,3
Contig_9579	pfam00723	Glyco_hydro_15,	6,0	4,5	11,3	7,3
Contig_31963	pfam00232	Glyco_hydro_1,	4,9	0,0	17,0	7,3
Contig_82327	pfam02057	Glyco_hydro_59,	3,0	1,2	17,6	7,3
Contig_539648	pfam03639	Glyco_hydro_81,	18,3	3,5	0,0	7,3
Contig_582108	pfam01532	Glyco_hydro_47,	8,5	4,0	9,4	7,3
Contig_42850	pfam12899	Glyco_hydro_100,	1,8	9,0	11,0	7,3
Contig_246001	pfam00722	Glyco_hydro_16,	4,1	9,7	8,0	7,3
Contig_19837	pfam01074	Glyco_hydro_38,	0,7	0,4	20,6	7,3
Contig_183634	pfam10566	Glyco_hydro_97,	9,0	12,7	0,0	7,2
Contig_311151	pfam01074	Glyco_hydro_38,	4,8	1,0	16,0	7,2
Contig_371214	pfam00295	Glyco_hydro_28,	7,8	1,8	12,1	7,2
Contig_112676	pfam00933	Glyco_hydro_3,	1,4	2,6	17,7	7,2
Contig_24407	pfam02446	Glyco_hydro_77,	3,9	1,6	16,2	7,2
Contig_516999	pfam02446	Glyco_hydro_77,	12,5	7,1	2,1	7,2
Contig_49064	pfam00704	Glyco_hydro_18,	11,4	0,0	10,2	7,2
Contig_135854	pfam00933	Glyco_hydro_3,	4,9	1,5	15,2	7,2
Contig_196465	pfam10566	Glyco_hydro_97,	8,2	12,1	1,3	7,2
Contig_178491	pfam03662	Glyco_hydro_79n,	4,9	9,4	7,3	7,2
Contig_54549	pfam02837	Glyco_hydro_2_N,	6,0	3,0	12,5	7,2
Contig_421298	pfam03200	Glyco_hydro_63,	0,9	12,2	8,4	7,2
Contig_142884	pfam02011	Glyco_hydro_48,	0,0	2,1	19,4	7,2
Contig_112434	pfam12899	Glyco_hydro_100,	1,4	3,3	16,9	7,2
Contig_138299	pfam12899	Glyco_hydro_100,	0,0	0,8	20,6	7,2
Contig_312501	pfam00722	Glyco_hydro_16,	12,3	9,1	0,0	7,2
Contig_311677	pfam00331	Glyco_hydro_10,	11,8	7,6	2,1	7,2
Contig_471750	pfam03632	Glyco_hydro_65m,	14,4	7,0	0,0	7,1
Contig_105092	pfam03659	Glyco_hydro_71,	3,9	4,7	12,9	7,1
Contig_67577	pfam00251	Glyco_hydro_32N,	10,6	10,8	0,0	7,1
Contig_240101	pfam00759	Glyco_hydro_9,	4,8	10,5	6,0	7,1
Contig_155477	pfam03648	Glyco_hydro_67N,	8,7	6,7	5,9	7,1
Contig_148624	pfam12891	Glyco_hydro_44,	3,9	0,6	16,9	7,1
Contig_274872	pfam03663	Glyco_hydro_76,	9,6	6,7	5,0	7,1
Contig_162396	pfam02324	Glyco_hydro_70,	14,2	3,1	3,9	7,1
Contig_138856	pfam01074	Glyco_hydro_38,	7,3	4,9	9,0	7,1
Contig_118854	pfam03659	Glyco_hydro_71,	3,4	1,0	16,8	7,1
Contig_8946	pfam08306	Glyco_hydro_98M,	6,9	1,4	12,9	7,1
Contig_20546	pfam00933	Glyco_hydro_3,	2,9	3,6	14,7	7,1
Contig_228602	pfam04616	Glyco_hydro_43,	7,3	13,8	0,0	7,1
Contig_78432	pfam01532	Glyco_hydro_47,	0,8	12,8	7,6	7,0
Contig_250469	pfam01055	Glyco_hydro_31,	7,9	9,1	4,2	7,0
Contig_216574	pfam03662	Glyco_hydro_79n,	10,1	3,6	7,4	7,0
Contig_429919	pfam02324	Glyco_hydro_70,	4,4	5,9	10,9	7,0
Contig_449037	pfam00332	Glyco_hydro_17,	6,7	7,7	6,7	7,0
Contig_402236	pfam02324	Glyco_hydro_70,	9,4	10,2	1,6	7,0
Contig_117414	pfam02449	Glyco_hydro_42,	3,9	10,7	6,4	7,0
Contig_131940	pfam00331	Glyco_hydro_10,	0,6	2,8	17,6	7,0
Contig_93063	pfam00182	Glyco_hydro_19,	2,7	18,2	0,0	7,0
Contig_142151	pfam04616	Glyco_hydro_43,	4,4	7,5	9,1	7,0
Contig_120050	pfam02435	Glyco_hydro_68,	10,9	0,3	9,7	7,0
Contig_197183	pfam00933	Glyco_hydro_3,	10,6	10,3	0,0	7,0
Contig_540784	pfam10566	Glyco_hydro_97,	9,2	8,1	3,5	7,0
Contig_286427	pfam03639	Glyco_hydro_81,	8,7	12,2	0,0	7,0
Contig_8295	pfam01074	Glyco_hydro_38,	7,4	7,4	6,1	7,0
Contig_217651	pfam01183	Glyco_hydro_25,	3,1	13,9	3,9	7,0
Contig_20362	pfam01532	Glyco_hydro_47,	0,6	3,1	17,1	6,9
Contig_57304	pfam02435	Glyco_hydro_68,	4,8	4,1	11,9	6,9

Contig_543934	pfam12899	Glyco_hydro_100,	20,8	0,0	0,0	6,9
Contig_344866	pfam00722	Glyco_hydro_16,	7,1	13,7	0,0	6,9
Contig_578163	pfam03644	Glyco_hydro_85,	10,9	9,8	0,0	6,9
Contig_43989	pfam00933	Glyco_hydro_3,	1,1	3,3	16,3	6,9
Contig_144798	pfam02837	Glyco_hydro_2_N,	1,9	9,4	9,4	6,9
Contig_7206	pfam01630	Glyco_hydro_56,	16,5	4,1	0,0	6,9
Contig_7631	pfam02449	Glyco_hydro_42,	6,7	3,5	10,4	6,9
Contig_581186	pfam02324	Glyco_hydro_70,	4,7	10,0	5,9	6,9
Contig_116662	pfam03065	Glyco_hydro_57,	16,4	4,2	0,0	6,9
Contig_68849	pfam02055	Glyco_hydro_30,	11,2	0,0	9,3	6,8
Contig_141416	pfam00295	Glyco_hydro_28,	0,0	8,1	12,3	6,8
Contig_264661	pfam02435	Glyco_hydro_68,	8,7	6,1	5,7	6,8
Contig_102936	pfam01074	Glyco_hydro_38,	0,0	3,4	16,9	6,8
Contig_367743	pfam02836	Glyco_hydro_2_C,	12,4	6,1	1,8	6,8
Contig_383842	pfam00295	Glyco_hydro_28,	6,2	14,1	0,0	6,8
Contig_113669	pfam00704	Glyco_hydro_18,	8,2	12,1	0,0	6,8
Contig_24282	pfam00723	Glyco_hydro_15,	1,7	4,0	14,6	6,8
Contig_468650	pfam00295	Glyco_hydro_28,	14,7	5,6	0,0	6,8
Contig_82014	pfam01301	Glyco_hydro_35,	4,3	2,6	13,4	6,7
Contig_301822	pfam01055	Glyco_hydro_31,	6,7	10,0	3,5	6,7
Contig_393455	pfam02056	Glyco_hydro_4,	13,1	7,1	0,0	6,7
Contig_364283	pfam00251	Glyco_hydro_32N,	3,5	12,4	4,3	6,7
Contig_208353	pfam00295	Glyco_hydro_28,	10,2	10,0	0,0	6,7
Contig_303735	pfam00759	Glyco_hydro_9,	5,0	8,3	6,8	6,7
Contig_398465	pfam01074	Glyco_hydro_38,	8,5	11,5	0,0	6,7
Contig_63477	pfam03639	Glyco_hydro_81,	10,8	0,4	8,9	6,7
Contig_1176	pfam07470	Glyco_hydro_88,	6,1	11,0	2,9	6,7
Contig_387821	pfam03663	Glyco_hydro_76,	9,0	3,6	7,4	6,7
Contig_134573	pfam01055	Glyco_hydro_31,	8,9	5,0	6,1	6,7
Contig_4873	pfam07470	Glyco_hydro_88,	9,9	6,0	4,1	6,7
Contig_59301	pfam00704	Glyco_hydro_18,	12,1	0,7	7,1	6,6
Contig_63154	pfam02435	Glyco_hydro_68,	10,0	2,4	7,5	6,6
Contig_69052	pfam11975	Glyco_hydro_4C,	9,0	7,4	3,6	6,6
Contig_129523	pfam00728	Glyco_hydro_20,	5,8	2,8	11,4	6,6
Contig_83819	pfam00759	Glyco_hydro_9,	6,8	8,7	4,4	6,6
Contig_18457	pfam02446	Glyco_hydro_77,	8,7	6,4	4,8	6,6
Contig_5429	pfam13199	Glyco_hydro_66,	3,4	1,2	15,2	6,6
Contig_391126	pfam00332	Glyco_hydro_17,	14,4	0,6	4,8	6,6
Contig_7580	pfam13199	Glyco_hydro_66,	1,0	6,7	12,0	6,6
Contig_18401	pfam00331	Glyco_hydro_10,	7,0	7,5	5,3	6,6
Contig_387798	pfam01055	Glyco_hydro_31,	19,7	0,0	0,0	6,6
Contig_43553	pfam12899	Glyco_hydro_100,	2,8	4,3	12,6	6,6
Contig_153156	pfam01074	Glyco_hydro_38,	0,0	9,7	10,0	6,6
Contig_202410	pfam02011	Glyco_hydro_48,	4,5	11,7	3,4	6,6
Contig_64125	pfam01055	Glyco_hydro_31,	0,0	4,5	15,1	6,6
Contig_141181	pfam01183	Glyco_hydro_25,	0,0	2,3	17,3	6,5
Contig_301193	pfam01055	Glyco_hydro_31,	19,6	0,0	0,0	6,5
Contig_339144	pfam07971	Glyco_hydro_92,	8,2	7,7	3,7	6,5
Contig_147877	pfam02449	Glyco_hydro_42,	7,4	1,5	10,7	6,5
Contig_165435	pfam00704	Glyco_hydro_18,	4,3	9,9	5,3	6,5
Contig_272722	pfam00759	Glyco_hydro_9,	8,9	10,7	0,0	6,5
Contig_361821	pfam02838	Glyco_hydro_20b,	7,9	8,7	3,0	6,5
Contig_332021	pfam02838	Glyco_hydro_20b,	13,7	5,8	0,0	6,5
Contig_66637	pfam03065	Glyco_hydro_57,	2,3	5,7	11,5	6,5
Contig_302732	pfam00722	Glyco_hydro_16,	7,9	10,7	0,8	6,5
Contig_475734	pfam00933	Glyco_hydro_3,	7,6	3,0	8,9	6,5
Contig_3936	pfam10566	Glyco_hydro_97,	1,7	5,1	12,6	6,5
Contig_498405	pfam07470	Glyco_hydro_88,	8,5	10,9	0,0	6,5
Contig_537705	pfam00759	Glyco_hydro_9,	18,9	0,4	0,0	6,5
Contig_466125	pfam00704	Glyco_hydro_18,	2,8	4,6	12,0	6,5
Contig_105532	pfam00759	Glyco_hydro_9,	5,0	2,1	12,3	6,5
Contig_270316	pfam00332	Glyco_hydro_17,	7,7	11,6	0,0	6,4
Contig_559258	pfam07971	Glyco_hydro_92,	19,3	0,0	0,0	6,4
Contig_61417	pfam01915	Glyco_hydro_3_C,	5,3	0,8	13,2	6,4
Contig_53672	pfam03198	Glyco_hydro_72,	0,0	0,0	19,3	6,4
Contig_427519	pfam00295	Glyco_hydro_28,	7,3	7,3	4,7	6,4
Contig_158700	pfam00331	Glyco_hydro_10,	10,0	5,9	3,4	6,4
Contig_150897	pfam00722	Glyco_hydro_16,	3,0	2,5	13,8	6,4
Contig_46511	pfam02055	Glyco_hydro_30,	7,0	0,7	11,6	6,4
Contig_310099	pfam01915	Glyco_hydro_3_C,	8,3	4,8	6,0	6,4
Contig_468959	pfam03639	Glyco_hydro_81,	7,3	11,9	0,0	6,4

Contig_216593	pfam12899	Glyco_hydro_100,	17,1	2,1	0,0	6,4
Contig_238573	pfam12899	Glyco_hydro_100,	1,7	6,2	11,3	6,4
Contig_41538	pfam03065	Glyco_hydro_57,	1,8	3,8	13,5	6,4
Contig_84973	pfam00331	Glyco_hydro_10,	0,0	2,1	17,1	6,4
Contig_14592	pfam07477	Glyco_hydro_67C,	0,8	2,2	16,1	6,4
Contig_119996	pfam12899	Glyco_hydro_100,	3,2	0,0	15,9	6,4
Contig_145155	pfam01532	Glyco_hydro_47,	7,2	0,0	11,9	6,4
Contig_313827	pfam07488	Glyco_hydro_67M,	1,7	9,0	8,4	6,4
Contig_474066	pfam03718	Glyco_hydro_49,	3,6	7,8	7,7	6,4
Contig_568260	pfam03718	Glyco_hydro_49,	18,1	1,0	0,0	6,4
Contig_61555	pfam00728	Glyco_hydro_20,	15,1	0,8	3,2	6,4
Contig_59365	pfam00728	Glyco_hydro_20,	6,2	8,9	3,9	6,4
Contig_67902	pfam03644	Glyco_hydro_85,	10,3	2,3	6,4	6,3
Contig_496082	pfam03663	Glyco_hydro_76,	16,7	2,3	0,0	6,3
Contig_30951	pfam00182	Glyco_hydro_19,	4,3	2,6	12,1	6,3
Contig_340252	pfam07335	Glyco_hydro_75,	7,0	12,0	0,0	6,3
Contig_358115	pfam03718	Glyco_hydro_49,	13,1	5,9	0,0	6,3
Contig_579665	pfam03644	Glyco_hydro_85,	2,2	2,6	14,2	6,3
Contig_113104	pfam01055	Glyco_hydro_31,	8,8	7,7	2,4	6,3
Contig_261152	pfam00331	Glyco_hydro_10,	7,8	0,7	10,4	6,3
Contig_546462	pfam02055	Glyco_hydro_30,	15,3	3,6	0,0	6,3
Contig_435037	pfam07971	Glyco_hydro_92,	18,9	0,0	0,0	6,3
Contig_2344	pfam01301	Glyco_hydro_35,	1,9	3,1	13,9	6,3
Contig_314612	pfam03662	Glyco_hydro_79n,	16,4	2,5	0,0	6,3
Contig_261086	pfam12899	Glyco_hydro_100,	8,9	10,0	0,0	6,3
Contig_285254	pfam01301	Glyco_hydro_35,	6,2	4,8	7,8	6,3
Contig_571358	pfam00933	Glyco_hydro_3,	11,0	7,9	0,0	6,3
Contig_35992	pfam00232	Glyco_hydro_1,	6,3	3,0	9,5	6,3
Contig_343673	pfam11975	Glyco_hydro_4C,	3,4	5,3	10,1	6,3
Contig_1007	pfam00759	Glyco_hydro_9,	9,3	4,9	4,6	6,3
Contig_129933	pfam01055	Glyco_hydro_31,	11,2	7,6	0,0	6,3
Contig_143540	pfam13199	Glyco_hydro_66,	0,0	4,8	14,0	6,3
Contig_54119	pfam00251	Glyco_hydro_32N,	0,0	8,3	10,5	6,3
Contig_305194	pfam12899	Glyco_hydro_100,	5,2	13,6	0,0	6,3
Contig_109132	pfam02838	Glyco_hydro_20b,	6,6	2,0	10,2	6,3
Contig_538983	pfam03659	Glyco_hydro_71,	16,9	1,9	0,0	6,3
Contig_314897	pfam01630	Glyco_hydro_56,	5,4	13,3	0,0	6,2
Contig_380685	pfam00759	Glyco_hydro_9,	6,0	12,7	0,0	6,2
Contig_107209	pfam01074	Glyco_hydro_38,	8,2	6,0	4,5	6,2
Contig_291775	pfam01055	Glyco_hydro_31,	0,0	18,7	0,0	6,2
Contig_95031	pfam00704	Glyco_hydro_18,	14,8	1,9	1,9	6,2
Contig_79759	pfam03718	Glyco_hydro_49,	8,2	1,8	8,7	6,2
Contig_19282	pfam01055	Glyco_hydro_31,	0,0	2,4	16,2	6,2
Contig_96650	pfam12899	Glyco_hydro_100,	2,5	0,7	15,4	6,2
Contig_36888	pfam02435	Glyco_hydro_68,	6,4	0,0	12,1	6,2
Contig_568765	pfam02449	Glyco_hydro_42,	17,7	0,9	0,0	6,2
Contig_6909	pfam02449	Glyco_hydro_42,	5,1	7,5	6,0	6,2
Contig_4498	pfam01532	Glyco_hydro_47,	0,3	2,2	16,1	6,2
Contig_18579	pfam02324	Glyco_hydro_70,	6,2	3,5	8,8	6,2
Contig_209930	pfam00722	Glyco_hydro_16,	7,9	10,6	0,0	6,2
Contig_233453	pfam03200	Glyco_hydro_63,	14,8	3,7	0,0	6,2
Contig_82809	pfam03663	Glyco_hydro_76,	3,0	4,4	11,1	6,2
Contig_289779	pfam00722	Glyco_hydro_16,	7,4	2,0	9,1	6,2
Contig_122537	pfam03200	Glyco_hydro_63,	12,4	2,0	4,1	6,2
Contig_278164	pfam01301	Glyco_hydro_35,	5,7	12,8	0,0	6,2
Contig_383702	pfam00182	Glyco_hydro_19,	5,7	12,8	0,0	6,2
Contig_569426	pfam07477	Glyco_hydro_67C,	17,2	1,2	0,0	6,1
Contig_220453	pfam02324	Glyco_hydro_70,	9,3	9,1	0,0	6,1
Contig_453352	pfam02838	Glyco_hydro_20b,	2,3	11,2	4,8	6,1
Contig_52815	pfam04616	Glyco_hydro_43,	3,2	10,0	5,2	6,1
Contig_440094	pfam07477	Glyco_hydro_67C,	4,7	13,6	0,0	6,1
Contig_27945	pfam03200	Glyco_hydro_63,	11,8	5,4	1,2	6,1
Contig_33623	pfam07748	Glyco_hydro_38C,	1,9	0,0	16,4	6,1
Contig_204776	pfam01229	Glyco_hydro_39,	3,5	6,2	8,6	6,1
Contig_535294	pfam11790	Glyco_hydro_cc,	7,2	11,1	0,0	6,1
Contig_564274	pfam00251	Glyco_hydro_32N,	7,9	10,4	0,0	6,1
Contig_383267	pfam01229	Glyco_hydro_39,	8,1	2,0	8,2	6,1
Contig_265953	pfam00251	Glyco_hydro_32N,	11,0	7,3	0,0	6,1
Contig_307089	pfam12899	Glyco_hydro_100,	6,5	11,8	0,0	6,1
Contig_291031	pfam03200	Glyco_hydro_63,	2,4	6,1	9,8	6,1
Contig_544588	pfam00723	Glyco_hydro_15,	12,5	0,0	5,7	6,1

Contig_30590	pfam08307	Glyco_hydro_98C,	5,2	3,2	9,8	6,1
Contig_47946	pfam00728	Glyco_hydro_20,	8,9	0,3	8,9	6,1
Contig_130439	pfam00840	Glyco_hydro_7,	1,5	12,9	3,8	6,1
Contig_64592	pfam01074	Glyco_hydro_38,	0,6	0,7	16,9	6,1
Contig_32894	pfam12899	Glyco_hydro_100,	0,0	7,6	10,5	6,1
Contig_153299	pfam07477	Glyco_hydro_67C,	7,3	3,5	7,3	6,0
Contig_79324	pfam02449	Glyco_hydro_42,	1,1	1,3	15,8	6,0
Contig_192250	pfam00332	Glyco_hydro_17,	4,7	13,4	0,0	6,0
Contig_348278	pfam00722	Glyco_hydro_16,	7,9	7,4	2,8	6,0
Contig_288433	pfam00933	Glyco_hydro_3,	6,5	11,6	0,0	6,0
Contig_43572	pfam01532	Glyco_hydro_47,	0,0	4,2	13,9	6,0
Contig_4706	pfam02057	Glyco_hydro_59,	6,1	2,9	9,1	6,0
Contig_528046	pfam00728	Glyco_hydro_20,	17,0	1,1	0,0	6,0
Contig_46644	pfam07488	Glyco_hydro_67M,	1,7	1,5	14,8	6,0
Contig_138710	pfam03659	Glyco_hydro_71,	5,4	3,2	9,5	6,0
Contig_430252	pfam00251	Glyco_hydro_32N,	7,1	10,9	0,0	6,0
Contig_99228	pfam03636	Glyco_hydro_65N,	0,0	0,0	18,0	6,0
Contig_120916	pfam01055	Glyco_hydro_31,	0,7	2,4	14,9	6,0
Contig_407188	pfam00232	Glyco_hydro_1,	11,5	3,1	3,4	6,0
Contig_274955	pfam12899	Glyco_hydro_100,	5,5	12,4	0,0	6,0
Contig_170363	pfam11975	Glyco_hydro_4C,	4,6	13,3	0,0	6,0
Contig_207409	pfam03644	Glyco_hydro_85,	7,1	10,7	0,0	6,0
Contig_113140	pfam03512	Glyco_hydro_52,	7,9	0,0	9,9	6,0
Contig_297061	pfam01301	Glyco_hydro_35,	9,9	4,7	3,3	6,0
Contig_102102	pfam01074	Glyco_hydro_38,	0,7	1,7	15,5	5,9
Contig_220477	pfam00704	Glyco_hydro_18,	5,4	10,2	2,2	5,9
Contig_276291	pfam02055	Glyco_hydro_30,	13,7	4,1	0,0	5,9
Contig_572703	pfam01270	Glyco_hydro_8,	15,0	2,7	0,0	5,9
Contig_582972	pfam00182	Glyco_hydro_19,	5,4	12,4	0,0	5,9
Contig_71496	pfam00704	Glyco_hydro_18,	0,0	4,1	13,6	5,9
Contig_53527	pfam03718	Glyco_hydro_49,	7,4	10,4	0,0	5,9
Contig_556160	pfam07477	Glyco_hydro_67C,	14,5	3,2	0,0	5,9
Contig_290819	pfam02449	Glyco_hydro_42,	3,0	4,8	9,9	5,9
Contig_159849	pfam07971	Glyco_hydro_92,	3,6	6,9	7,2	5,9
Contig_181675	pfam00728	Glyco_hydro_20,	8,4	9,3	0,0	5,9
Contig_563279	pfam02446	Glyco_hydro_77,	2,3	6,9	8,5	5,9
Contig_531003	pfam00704	Glyco_hydro_18,	17,7	0,0	0,0	5,9
Contig_114666	pfam00722	Glyco_hydro_16,	4,3	11,2	2,1	5,9
Contig_28423	pfam00840	Glyco_hydro_7,	7,1	1,8	8,8	5,9
Contig_243349	pfam00251	Glyco_hydro_32N,	15,7	1,9	0,0	5,9
Contig_96929	pfam03200	Glyco_hydro_63,	5,4	1,3	10,9	5,9
Contig_29694	pfam03200	Glyco_hydro_63,	1,3	6,9	9,5	5,9
Contig_489953	pfam01301	Glyco_hydro_35,	16,2	1,3	0,0	5,8
Contig_149864	pfam00295	Glyco_hydro_28,	5,9	11,5	0,0	5,8
Contig_212674	pfam02156	Glyco_hydro_26,	4,5	13,0	0,0	5,8
Contig_36366	pfam03639	Glyco_hydro_81,	6,4	2,9	8,1	5,8
Contig_190167	pfam00722	Glyco_hydro_16,	15,8	1,6	0,0	5,8
Contig_11525	pfam03065	Glyco_hydro_57,	8,2	0,3	9,0	5,8
Contig_18510	pfam03200	Glyco_hydro_63,	8,3	1,8	7,3	5,8
Contig_36929	pfam01055	Glyco_hydro_31,	6,0	9,5	1,9	5,8
Contig_92438	pfam00933	Glyco_hydro_3,	1,8	0,0	15,6	5,8
Contig_12642	pfam02446	Glyco_hydro_77,	4,2	0,9	12,3	5,8
Contig_569577	pfam00331	Glyco_hydro_10,	12,2	5,1	0,0	5,8
Contig_185353	pfam00704	Glyco_hydro_18,	5,2	12,1	0,0	5,8
Contig_168429	pfam02015	Glyco_hydro_45,	10,0	7,3	0,0	5,7
Contig_514587	pfam01373	Glyco_hydro_14,	12,0	5,2	0,0	5,7
Contig_5984	pfam00728	Glyco_hydro_20,	5,4	0,5	11,4	5,7
Contig_57285	pfam00182	Glyco_hydro_19,	4,7	4,1	8,4	5,7
Contig_52956	pfam02055	Glyco_hydro_30,	3,7	2,2	11,3	5,7
Contig_230259	pfam02449	Glyco_hydro_42,	6,7	10,5	0,0	5,7
Contig_246298	pfam01301	Glyco_hydro_35,	0,7	3,5	13,0	5,7
Contig_73080	pfam00232	Glyco_hydro_1,	0,0	2,4	14,8	5,7
Contig_395702	pfam03632	Glyco_hydro_65m,	2,5	8,5	6,2	5,7
Contig_76805	pfam04616	Glyco_hydro_43,	10,1	2,1	5,0	5,7
Contig_423210	pfam07971	Glyco_hydro_92,	12,7	0,9	3,6	5,7
Contig_400468	pfam00332	Glyco_hydro_17,	13,0	4,1	0,0	5,7
Contig_97848	pfam03065	Glyco_hydro_57,	6,5	0,0	10,6	5,7
Contig_356871	pfam01055	Glyco_hydro_31,	3,1	6,2	7,8	5,7
Contig_522356	pfam01055	Glyco_hydro_31,	16,4	0,7	0,0	5,7
Contig_312295	pfam00232	Glyco_hydro_1,	11,3	5,8	0,0	5,7
Contig_262405	pfam01229	Glyco_hydro_39,	10,6	6,4	0,0	5,7

Contig_50890	pfam03718	Glyco_hydro_49,	3,8	1,8	11,4	5,7
Contig_496742	pfam01074	Glyco_hydro_38,	7,6	0,0	9,5	5,7
Contig_142289	pfam02324	Glyco_hydro_70,	6,9	1,7	8,5	5,7
Contig_197174	pfam02057	Glyco_hydro_59,	2,3	14,7	0,0	5,7
Contig_380556	pfam02011	Glyco_hydro_48,	2,1	14,8	0,0	5,7
Contig_311493	pfam02449	Glyco_hydro_42,	0,5	4,6	11,9	5,7
Contig_475857	pfam00933	Glyco_hydro_3,	16,2	0,8	0,0	5,7
Contig_124148	pfam01630	Glyco_hydro_56,	7,6	4,9	4,4	5,6
Contig_53185	pfam01532	Glyco_hydro_47,	1,3	0,0	15,7	5,6
Contig_244185	pfam00331	Glyco_hydro_10,	6,0	4,2	6,6	5,6
Contig_10036	pfam07477	Glyco_hydro_67C,	0,0	0,0	16,9	5,6
Contig_162687	pfam00933	Glyco_hydro_3,	2,0	0,0	14,9	5,6
Contig_334634	pfam08244	Glyco_hydro_32C,	8,7	8,2	0,0	5,6
Contig_22330	pfam03200	Glyco_hydro_63,	2,7	4,1	10,1	5,6
Contig_125193	pfam03659	Glyco_hydro_71,	12,1	0,5	4,3	5,6
Contig_150565	pfam01532	Glyco_hydro_47,	0,0	2,5	14,4	5,6
Contig_290125	pfam12891	Glyco_hydro_44,	4,4	12,4	0,0	5,6
Contig_542948	pfam02057	Glyco_hydro_59,	4,7	12,2	0,0	5,6
Contig_10852	pfam02435	Glyco_hydro_68,	11,9	2,4	2,5	5,6
Contig_194702	pfam00331	Glyco_hydro_10,	5,6	7,1	4,2	5,6
Contig_323363	pfam03644	Glyco_hydro_85,	1,2	12,5	3,1	5,6
Contig_132730	pfam03644	Glyco_hydro_85,	1,1	2,0	13,7	5,6
Contig_119931	pfam00704	Glyco_hydro_18,	5,9	4,0	6,8	5,6
Contig_554507	pfam03718	Glyco_hydro_49,	10,8	5,9	0,0	5,6
Contig_353047	pfam01055	Glyco_hydro_31,	5,9	10,7	0,0	5,6
Contig_146301	pfam03663	Glyco_hydro_76,	0,0	0,0	16,7	5,6
Contig_37302	pfam00704	Glyco_hydro_18,	7,3	0,7	8,7	5,6
Contig_20811	pfam01270	Glyco_hydro_8,	8,0	4,9	3,7	5,6
Contig_141920	pfam00840	Glyco_hydro_7,	2,3	0,0	14,4	5,6
Contig_97525	pfam03200	Glyco_hydro_63,	2,3	8,7	5,7	5,5
Contig_194674	pfam07470	Glyco_hydro_88,	5,1	11,5	0,0	5,5
Contig_561870	pfam01055	Glyco_hydro_31,	3,6	6,4	6,6	5,5
Contig_331457	pfam01915	Glyco_hydro_3_C,	8,9	7,7	0,0	5,5
Contig_148869	pfam01229	Glyco_hydro_39,	4,7	0,0	11,9	5,5
Contig_54334	pfam03662	Glyco_hydro_79n,	11,9	4,6	0,0	5,5
Contig_37323	pfam01270	Glyco_hydro_8,	1,2	4,7	10,5	5,5
Contig_37410	pfam01055	Glyco_hydro_31,	4,0	2,4	10,0	5,5
Contig_83713	pfam11790	Glyco_hydro_cc,	1,6	7,1	7,8	5,5
Contig_135460	pfam00332	Glyco_hydro_17,	6,4	3,4	6,6	5,5
Contig_248901	pfam00232	Glyco_hydro_1,	0,6	15,8	0,0	5,5
Contig_99532	pfam03718	Glyco_hydro_49,	1,4	3,3	11,8	5,5
Contig_538170	pfam00331	Glyco_hydro_10,	16,4	0,0	0,0	5,5
Contig_182166	pfam03663	Glyco_hydro_76,	10,5	5,9	0,0	5,5
Contig_456196	pfam11790	Glyco_hydro_cc,	11,5	4,9	0,0	5,4
Contig_80997	pfam00232	Glyco_hydro_1,	0,0	1,8	14,6	5,4
Contig_316340	pfam00331	Glyco_hydro_10,	6,4	6,7	3,3	5,4
Contig_6419	pfam13199	Glyco_hydro_66,	0,0	6,9	9,5	5,4
Contig_419341	pfam01630	Glyco_hydro_56,	6,6	7,4	2,4	5,4
Contig_229604	pfam03200	Glyco_hydro_63,	7,1	9,2	0,0	5,4
Contig_96857	pfam07745	Glyco_hydro_53,	8,7	7,6	0,0	5,4
Contig_440735	pfam03200	Glyco_hydro_63,	5,6	9,5	1,2	5,4
Contig_474487	pfam07748	Glyco_hydro_38C,	15,6	0,7	0,0	5,4
Contig_245755	pfam00704	Glyco_hydro_18,	5,6	3,8	6,9	5,4
Contig_142735	pfam03644	Glyco_hydro_85,	0,0	5,7	10,5	5,4
Contig_26918	pfam01532	Glyco_hydro_47,	7,3	0,5	8,5	5,4
Contig_86996	pfam00759	Glyco_hydro_9,	7,8	8,4	0,0	5,4
Contig_268491	pfam00232	Glyco_hydro_1,	1,5	14,7	0,0	5,4
Contig_41926	pfam00933	Glyco_hydro_3,	1,9	5,0	9,3	5,4
Contig_58049	pfam00232	Glyco_hydro_1,	0,0	3,1	13,0	5,4
Contig_92747	pfam01055	Glyco_hydro_31,	3,8	5,4	7,0	5,4
Contig_227454	pfam08244	Glyco_hydro_32C,	10,6	3,0	2,5	5,4
Contig_523381	pfam03200	Glyco_hydro_63,	16,1	0,0	0,0	5,4
Contig_59364	pfam00728	Glyco_hydro_20,	3,9	1,6	10,6	5,4
Contig_472550	pfam02837	Glyco_hydro_2_N,	9,1	6,9	0,0	5,3
Contig_397933	pfam08306	Glyco_hydro_98M,	13,9	2,1	0,0	5,3
Contig_506698	pfam01301	Glyco_hydro_35,	11,4	2,0	2,7	5,3
Contig_150886	pfam04616	Glyco_hydro_43,	4,1	5,0	6,9	5,3
Contig_8417	pfam10566	Glyco_hydro_97,	5,5	0,0	10,4	5,3
Contig_123785	pfam01270	Glyco_hydro_8,	0,9	4,6	10,5	5,3
Contig_299594	pfam03644	Glyco_hydro_85,	1,2	14,8	0,0	5,3
Contig_546782	pfam00704	Glyco_hydro_18,	15,9	0,0	0,0	5,3

Contig_28045	pfam00251	Glyco_hydro_32N,	8,7	6,1	1,2	5,3
Contig_277822	pfam12899	Glyco_hydro_100,	3,1	12,9	0,0	5,3
Contig_311869	pfam07488	Glyco_hydro_67M,	4,7	2,2	9,0	5,3
Contig_276586	pfam01270	Glyco_hydro_8,	0,0	11,4	4,5	5,3
Contig_440013	pfam01341	Glyco_hydro_6,	9,0	6,8	0,0	5,3
Contig_301388	pfam00704	Glyco_hydro_18,	8,1	7,8	0,0	5,3
Contig_133877	pfam01074	Glyco_hydro_38,	4,9	6,9	4,1	5,3
Contig_143867	pfam00704	Glyco_hydro_18,	3,2	1,9	10,7	5,3
Contig_203906	pfam01301	Glyco_hydro_35,	6,6	4,5	4,7	5,3
Contig_94870	pfam10566	Glyco_hydro_97,	3,7	12,1	0,0	5,3
Contig_411706	pfam03663	Glyco_hydro_76,	12,0	3,8	0,0	5,3
Contig_202651	pfam02449	Glyco_hydro_42,	10,4	5,4	0,0	5,3
Contig_280045	pfam10566	Glyco_hydro_97,	4,6	8,0	3,2	5,3
Contig_384981	pfam00722	Glyco_hydro_16,	12,7	3,1	0,0	5,3
Contig_432536	pfam03198	Glyco_hydro_72,	5,3	10,5	0,0	5,3
Contig_382320	pfam07488	Glyco_hydro_67M,	4,3	5,5	6,0	5,3
Contig_346339	pfam01055	Glyco_hydro_31,	5,4	8,1	2,2	5,3
Contig_39860	pfam00759	Glyco_hydro_9,	13,2	0,0	2,5	5,3
Contig_88787	pfam02055	Glyco_hydro_30,	3,2	5,8	6,7	5,2
Contig_307771	pfam00840	Glyco_hydro_7,	5,6	10,1	0,0	5,2
Contig_296255	pfam00840	Glyco_hydro_7,	7,7	8,0	0,0	5,2
Contig_293091	pfam00232	Glyco_hydro_1,	4,8	5,0	5,9	5,2
Contig_175037	pfam07971	Glyco_hydro_92,	14,3	1,4	0,0	5,2
Contig_381866	pfam01301	Glyco_hydro_35,	5,3	10,4	0,0	5,2
Contig_368020	pfam12899	Glyco_hydro_100,	1,1	12,0	2,6	5,2
Contig_33554	pfam01055	Glyco_hydro_31,	0,0	2,6	13,0	5,2
Contig_140056	pfam10566	Glyco_hydro_97,	1,2	0,0	14,5	5,2
Contig_41972	pfam02015	Glyco_hydro_45,	1,3	3,6	10,7	5,2
Contig_384600	pfam02015	Glyco_hydro_45,	4,9	5,9	4,9	5,2
Contig_293554	pfam00759	Glyco_hydro_9,	3,3	5,4	6,9	5,2
Contig_342472	pfam03633	Glyco_hydro_65C,	5,0	10,6	0,0	5,2
Contig_441661	pfam02324	Glyco_hydro_70,	4,3	11,3	0,0	5,2
Contig_333678	pfam04616	Glyco_hydro_43,	7,3	8,2	0,0	5,2
Contig_30343	pfam02446	Glyco_hydro_77,	0,0	0,0	15,5	5,2
Contig_140763	pfam00728	Glyco_hydro_20,	0,0	0,0	15,5	5,2
Contig_278423	pfam03718	Glyco_hydro_49,	9,4	6,2	0,0	5,2
Contig_44023	pfam12891	Glyco_hydro_44,	5,7	0,4	9,4	5,2
Contig_229366	pfam02324	Glyco_hydro_70,	4,9	10,6	0,0	5,2
Contig_8087	pfam01532	Glyco_hydro_47,	2,5	5,5	7,5	5,2
Contig_97298	pfam02837	Glyco_hydro_2_N,	0,0	4,0	11,5	5,1
Contig_519878	pfam01374	Glyco_hydro_46,	12,8	0,5	2,1	5,1
Contig_55148	pfam01915	Glyco_hydro_3_C,	4,0	3,2	8,2	5,1
Contig_300270	pfam00723	Glyco_hydro_15,	3,0	12,4	0,0	5,1
Contig_487885	pfam01055	Glyco_hydro_31,	13,7	1,7	0,0	5,1
Contig_98756	pfam13199	Glyco_hydro_66,	2,4	13,0	0,0	5,1
Contig_396723	pfam00704	Glyco_hydro_18,	7,4	8,0	0,0	5,1
Contig_400334	pfam00295	Glyco_hydro_28,	11,8	3,6	0,0	5,1
Contig_462378	pfam02449	Glyco_hydro_42,	3,0	4,9	7,4	5,1
Contig_422660	pfam03659	Glyco_hydro_71,	14,0	1,3	0,0	5,1
Contig_131181	pfam02838	Glyco_hydro_20b,	1,5	7,0	6,8	5,1
Contig_142447	pfam03200	Glyco_hydro_63,	7,2	8,1	0,0	5,1
Contig_18087	pfam13199	Glyco_hydro_66,	8,7	1,8	4,8	5,1
Contig_290715	pfam02156	Glyco_hydro_26,	15,3	0,0	0,0	5,1
Contig_50424	pfam03200	Glyco_hydro_63,	6,1	9,2	0,0	5,1
Contig_64060	pfam00331	Glyco_hydro_10,	8,6	3,8	2,8	5,1
Contig_59924	pfam00723	Glyco_hydro_15,	4,1	0,8	10,3	5,1
Contig_548470	pfam03198	Glyco_hydro_72,	4,4	0,0	10,9	5,1
Contig_85145	pfam03200	Glyco_hydro_63,	3,4	0,7	11,1	5,1
Contig_55754	pfam01055	Glyco_hydro_31,	5,5	5,2	4,5	5,1
Contig_464967	pfam01301	Glyco_hydro_35,	8,4	6,8	0,0	5,1
Contig_125434	pfam00728	Glyco_hydro_20,	3,5	3,1	8,6	5,0
Contig_199276	pfam03662	Glyco_hydro_79n,	8,6	6,5	0,0	5,0
Contig_293846	pfam01301	Glyco_hydro_35,	5,2	9,9	0,0	5,0
Contig_425981	pfam00704	Glyco_hydro_18,	2,2	5,7	7,2	5,0
Contig_71961	pfam03512	Glyco_hydro_52,	1,8	4,3	9,0	5,0
Contig_210585	pfam12899	Glyco_hydro_100,	4,1	11,0	0,0	5,0
Contig_270478	pfam00759	Glyco_hydro_9,	3,4	11,7	0,0	5,0
Contig_543568	pfam03659	Glyco_hydro_71,	2,2	2,3	10,6	5,0
Contig_343108	pfam02156	Glyco_hydro_26,	12,3	2,8	0,0	5,0
Contig_288058	pfam03633	Glyco_hydro_65C,	8,9	6,1	0,0	5,0
Contig_84794	pfam02011	Glyco_hydro_48,	4,3	0,0	10,8	5,0

Contig_500226	pfam00332	Glyco_hydro_17,	13,4	1,6	0,0	5,0
Contig_235607	pfam03200	Glyco_hydro_63,	11,5	3,5	0,0	5,0
Contig_32338	pfam01532	Glyco_hydro_47,	9,4	4,5	1,2	5,0
Contig_404883	pfam00728	Glyco_hydro_20,	7,1	7,9	0,0	5,0
Contig_371315	pfam00232	Glyco_hydro_1,	2,9	1,3	10,8	5,0
Contig_54940	pfam02057	Glyco_hydro_59,	5,0	8,2	1,8	5,0
Contig_467826	pfam03664	Glyco_hydro_62,	4,1	8,9	2,0	5,0
Contig_31248	pfam00840	Glyco_hydro_7,	1,5	6,0	7,4	5,0
Contig_363454	pfam07488	Glyco_hydro_67M,	10,8	4,1	0,0	5,0
Contig_400168	pfam00332	Glyco_hydro_17,	12,2	2,8	0,0	5,0
Contig_268265	pfam01341	Glyco_hydro_6,	7,4	7,5	0,0	5,0
Contig_327269	pfam01055	Glyco_hydro_31,	4,9	8,5	1,5	5,0
Contig_141453	pfam03718	Glyco_hydro_49,	6,6	1,1	7,1	5,0
Contig_133038	pfam01532	Glyco_hydro_47,	1,7	13,2	0,0	5,0
Contig_20026	pfam01055	Glyco_hydro_31,	5,2	7,2	2,5	4,9
Contig_164163	pfam01229	Glyco_hydro_39,	11,0	1,9	2,0	4,9
Contig_302287	pfam03663	Glyco_hydro_76,	2,6	12,3	0,0	4,9
Contig_270870	pfam01229	Glyco_hydro_39,	1,1	8,1	5,6	4,9
Contig_563606	pfam03663	Glyco_hydro_76,	14,8	0,0	0,0	4,9
Contig_361713	pfam01373	Glyco_hydro_14,	9,2	5,6	0,0	4,9
Contig_18391	pfam02446	Glyco_hydro_77,	4,6	10,2	0,0	4,9
Contig_380864	pfam04616	Glyco_hydro_43,	9,1	4,0	1,7	4,9
Contig_287601	pfam02836	Glyco_hydro_2_C,	7,7	4,4	2,6	4,9
Contig_142284	pfam01301	Glyco_hydro_35,	7,1	3,2	4,4	4,9
Contig_478410	pfam00232	Glyco_hydro_1,	1,7	13,1	0,0	4,9
Contig_277197	pfam01055	Glyco_hydro_31,	6,3	7,1	1,3	4,9
Contig_261906	pfam02057	Glyco_hydro_59,	10,8	3,9	0,0	4,9
Contig_272210	pfam01373	Glyco_hydro_14,	6,2	8,5	0,0	4,9
Contig_143582	pfam03644	Glyco_hydro_85,	12,0	0,0	2,7	4,9
Contig_290557	pfam01229	Glyco_hydro_39,	2,7	7,6	4,4	4,9
Contig_29546	pfam12899	Glyco_hydro_100,	0,6	1,5	12,5	4,9
Contig_239512	pfam02836	Glyco_hydro_2_C,	7,3	4,6	2,8	4,9
Contig_210003	pfam00933	Glyco_hydro_3,	5,7	8,9	0,0	4,9
Contig_23379	pfam01055	Glyco_hydro_31,	1,2	4,1	9,3	4,9
Contig_365952	pfam00728	Glyco_hydro_20,	8,0	6,6	0,0	4,9
Contig_361761	pfam01301	Glyco_hydro_35,	9,8	4,8	0,0	4,9
Contig_332463	pfam01532	Glyco_hydro_47,	14,6	0,0	0,0	4,9
Contig_845	pfam03200	Glyco_hydro_63,	3,7	2,6	8,3	4,9
Contig_60593	pfam00232	Glyco_hydro_1,	1,1	2,6	10,8	4,9
Contig_299745	pfam00457	Glyco_hydro_11,	12,9	1,7	0,0	4,9
Contig_204900	pfam00232	Glyco_hydro_1,	8,2	6,4	0,0	4,9
Contig_542880	pfam11790	Glyco_hydro_cc,	12,4	0,0	2,2	4,9
Contig_95479	pfam00728	Glyco_hydro_20,	7,0	5,9	1,7	4,9
Contig_233980	pfam01055	Glyco_hydro_31,	1,6	4,9	8,1	4,9
Contig_125369	pfam01532	Glyco_hydro_47,	9,2	0,0	5,3	4,8
Contig_554714	pfam01183	Glyco_hydro_25,	13,0	1,6	0,0	4,8
Contig_104405	pfam00251	Glyco_hydro_32N,	5,6	2,1	6,9	4,8
Contig_320361	pfam00232	Glyco_hydro_1,	0,0	14,5	0,0	4,8
Contig_550934	pfam01055	Glyco_hydro_31,	14,5	0,0	0,0	4,8
Contig_420634	pfam03198	Glyco_hydro_72,	6,4	0,0	8,1	4,8
Contig_59854	pfam07745	Glyco_hydro_53,	6,2	8,4	0,0	4,8
Contig_499729	pfam01301	Glyco_hydro_35,	0,0	7,1	7,4	4,8
Contig_348026	pfam00295	Glyco_hydro_28,	6,6	7,9	0,0	4,8
Contig_539075	pfam02055	Glyco_hydro_30,	14,5	0,0	0,0	4,8
Contig_529846	pfam00759	Glyco_hydro_9,	3,1	3,7	7,7	4,8
Contig_277809	pfam07971	Glyco_hydro_92,	11,1	3,4	0,0	4,8
Contig_268773	pfam00728	Glyco_hydro_20,	6,1	8,3	0,0	4,8
Contig_475345	pfam00232	Glyco_hydro_1,	14,5	0,0	0,0	4,8
Contig_502882	pfam13199	Glyco_hydro_66,	10,5	4,0	0,0	4,8
Contig_62747	pfam07748	Glyco_hydro_38C,	4,5	1,1	8,9	4,8
Contig_343385	pfam13199	Glyco_hydro_66,	11,8	2,7	0,0	4,8
Contig_537385	pfam00759	Glyco_hydro_9,	11,0	0,0	3,4	4,8
Contig_575660	pfam03662	Glyco_hydro_79n,	14,4	0,0	0,0	4,8
Contig_461486	pfam12899	Glyco_hydro_100,	11,0	3,4	0,0	4,8
Contig_275644	pfam00722	Glyco_hydro_16,	5,8	1,9	6,7	4,8
Contig_400403	pfam01301	Glyco_hydro_35,	11,5	2,9	0,0	4,8
Contig_195443	pfam03632	Glyco_hydro_65m,	9,1	5,3	0,0	4,8
Contig_451877	pfam00182	Glyco_hydro_19,	11,3	3,1	0,0	4,8
Contig_434080	pfam07470	Glyco_hydro_88,	12,7	1,6	0,0	4,8
Contig_440168	pfam00759	Glyco_hydro_9,	10,0	4,4	0,0	4,8
Contig_502298	pfam00704	Glyco_hydro_18,	11,7	2,6	0,0	4,8

Contig_475557	pfam00457	Glyco_hydro_11,	13,4	0,9	0,0	4,8
Contig_29045	pfam00331	Glyco_hydro_10,	2,3	2,1	9,9	4,8
Contig_84203	pfam03639	Glyco_hydro_81,	10,5	0,0	3,7	4,8
Contig_330411	pfam04616	Glyco_hydro_43,	5,5	7,7	1,1	4,7
Contig_221832	pfam10566	Glyco_hydro_97,	3,3	7,6	3,3	4,7
Contig_86283	pfam01301	Glyco_hydro_35,	14,2	0,0	0,0	4,7
Contig_37600	pfam03639	Glyco_hydro_81,	1,6	1,0	11,7	4,7
Contig_200667	pfam00295	Glyco_hydro_28,	3,1	11,1	0,0	4,7
Contig_272472	pfam00704	Glyco_hydro_18,	0,0	14,2	0,0	4,7
Contig_314664	pfam00704	Glyco_hydro_18,	4,2	10,0	0,0	4,7
Contig_48356	pfam03512	Glyco_hydro_52,	4,0	6,8	3,5	4,7
Contig_220346	pfam07335	Glyco_hydro_75,	5,4	2,2	6,7	4,7
Contig_532464	pfam03639	Glyco_hydro_81,	14,2	0,0	0,0	4,7
Contig_101327	pfam02446	Glyco_hydro_77,	4,1	3,3	6,8	4,7
Contig_53814	pfam00295	Glyco_hydro_28,	3,4	4,7	6,0	4,7
Contig_261765	pfam00232	Glyco_hydro_1,	6,7	4,7	2,8	4,7
Contig_30787	pfam03639	Glyco_hydro_81,	1,4	0,5	12,3	4,7
Contig_429036	pfam01055	Glyco_hydro_31,	10,6	3,6	0,0	4,7
Contig_501671	pfam01074	Glyco_hydro_38,	9,8	4,3	0,0	4,7
Contig_507538	pfam02057	Glyco_hydro_59,	12,3	1,9	0,0	4,7
Contig_509441	pfam01229	Glyco_hydro_39,	11,3	2,8	0,0	4,7
Contig_524294	pfam03198	Glyco_hydro_72,	14,1	0,0	0,0	4,7
Contig_65106	pfam01074	Glyco_hydro_38,	2,0	3,3	8,8	4,7
Contig_79795	pfam00704	Glyco_hydro_18,	6,3	1,1	6,7	4,7
Contig_268299	pfam00759	Glyco_hydro_9,	7,7	6,4	0,0	4,7
Contig_10662	pfam01630	Glyco_hydro_56,	0,0	3,3	10,8	4,7
Contig_350528	pfam03200	Glyco_hydro_63,	1,4	12,7	0,0	4,7
Contig_183613	pfam00728	Glyco_hydro_20,	2,3	3,1	8,7	4,7
Contig_58131	pfam03663	Glyco_hydro_76,	0,0	9,6	4,4	4,7
Contig_311036	pfam13199	Glyco_hydro_66,	5,7	8,3	0,0	4,7
Contig_491379	pfam00704	Glyco_hydro_18,	9,5	4,6	0,0	4,7
Contig_46275	pfam00933	Glyco_hydro_3,	0,0	4,6	9,5	4,7
Contig_377097	pfam03659	Glyco_hydro_71,	9,8	4,2	0,0	4,7
Contig_412421	pfam04616	Glyco_hydro_43,	13,4	0,7	0,0	4,7
Contig_142501	pfam12905	Glyco_hydro_101,	0,9	4,3	8,8	4,7
Contig_402688	pfam01074	Glyco_hydro_38,	9,9	0,0	4,1	4,7
Contig_558908	pfam12905	Glyco_hydro_101,	12,2	1,8	0,0	4,7
Contig_131588	pfam07745	Glyco_hydro_53,	5,8	0,0	8,2	4,7
Contig_134482	pfam01055	Glyco_hydro_31,	6,2	4,0	3,8	4,7
Contig_158157	pfam01341	Glyco_hydro_6,	7,8	6,2	0,0	4,7
Contig_173391	pfam00295	Glyco_hydro_28,	2,5	3,1	8,4	4,7
Contig_220429	pfam07748	Glyco_hydro_38C,	5,8	8,1	0,0	4,7
Contig_449423	pfam04616	Glyco_hydro_43,	12,4	1,6	0,0	4,7
Contig_362287	pfam02446	Glyco_hydro_77,	7,5	6,4	0,0	4,7
Contig_40319	pfam01532	Glyco_hydro_47,	6,7	0,0	7,2	4,6
Contig_341010	pfam00331	Glyco_hydro_10,	9,6	4,3	0,0	4,6
Contig_31634	pfam02836	Glyco_hydro_2_C,	0,9	1,7	11,3	4,6
Contig_86209	pfam07971	Glyco_hydro_92,	6,3	4,1	3,6	4,6
Contig_288884	pfam01373	Glyco_hydro_14,	8,0	5,9	0,0	4,6
Contig_108742	pfam07477	Glyco_hydro_67C,	9,7	4,2	0,0	4,6
Contig_347692	pfam00933	Glyco_hydro_3,	3,2	10,7	0,0	4,6
Contig_300088	pfam05838	Glyco_hydro_108,	8,9	5,0	0,0	4,6
Contig_118294	pfam01301	Glyco_hydro_35,	3,5	1,6	8,7	4,6
Contig_269767	pfam03200	Glyco_hydro_63,	8,6	5,2	0,0	4,6
Contig_245543	pfam00251	Glyco_hydro_32N,	1,5	8,8	3,6	4,6
Contig_160468	pfam01055	Glyco_hydro_31,	0,0	13,8	0,0	4,6
Contig_732	pfam07971	Glyco_hydro_92,	4,2	1,9	7,8	4,6
Contig_44087	pfam00704	Glyco_hydro_18,	2,4	1,4	9,9	4,6
Contig_144514	pfam02156	Glyco_hydro_26,	8,0	2,5	3,3	4,6
Contig_235358	pfam03639	Glyco_hydro_81,	9,2	2,0	2,5	4,6
Contig_414940	pfam00759	Glyco_hydro_9,	10,4	3,3	0,0	4,6
Contig_284603	pfam03443	Glyco_hydro_61,	3,4	7,5	2,8	4,6
Contig_335626	pfam12899	Glyco_hydro_100,	6,6	7,1	0,0	4,6
Contig_82505	pfam13199	Glyco_hydro_66,	1,0	0,0	12,7	4,6
Contig_163855	pfam00251	Glyco_hydro_32N,	9,8	3,9	0,0	4,6
Contig_331715	pfam01270	Glyco_hydro_8,	2,8	8,5	2,4	4,6
Contig_375746	pfam03065	Glyco_hydro_57,	8,9	2,0	2,8	4,6
Contig_176757	pfam08532	Glyco_hydro_42M,	6,2	7,5	0,0	4,6
Contig_98142	pfam01055	Glyco_hydro_31,	0,0	0,9	12,8	4,6
Contig_577951	pfam01074	Glyco_hydro_38,	11,9	1,8	0,0	4,6
Contig_560295	pfam00332	Glyco_hydro_17,	13,6	0,0	0,0	4,5

Contig_122757	pfam03644	Glyco_hydro_85,	0,0	1,0	12,6	4,5
Contig_270310	pfam03639	Glyco_hydro_81,	8,2	3,4	2,0	4,5
Contig_557174	pfam13647	Glyco_hydro_80,	11,4	2,3	0,0	4,5
Contig_302486	pfam00728	Glyco_hydro_20,	3,2	7,7	2,7	4,5
Contig_139539	pfam01270	Glyco_hydro_8,	3,9	0,0	9,7	4,5
Contig_25730	pfam03662	Glyco_hydro_79n,	0,0	0,0	13,6	4,5
Contig_427428	pfam00331	Glyco_hydro_10,	3,3	10,3	0,0	4,5
Contig_39248	pfam07748	Glyco_hydro_38C,	2,1	4,8	6,6	4,5
Contig_211019	pfam00232	Glyco_hydro_1,	4,2	9,4	0,0	4,5
Contig_360603	pfam02449	Glyco_hydro_42,	2,8	5,3	5,5	4,5
Contig_136365	pfam00704	Glyco_hydro_18,	3,7	0,7	9,2	4,5
Contig_38895	pfam01630	Glyco_hydro_56,	8,4	2,4	2,8	4,5
Contig_341174	pfam04616	Glyco_hydro_43,	9,3	4,3	0,0	4,5
Contig_64365	pfam03663	Glyco_hydro_76,	2,7	0,0	10,8	4,5
Contig_486453	pfam02449	Glyco_hydro_42,	7,3	6,3	0,0	4,5
Contig_97382	pfam02449	Glyco_hydro_42,	1,5	8,2	3,8	4,5
Contig_48105	pfam00840	Glyco_hydro_7,	0,0	1,2	12,3	4,5
Contig_34576	pfam00933	Glyco_hydro_3,	2,4	5,2	5,9	4,5
Contig_56098	pfam04616	Glyco_hydro_43,	8,5	3,1	1,9	4,5
Contig_116453	pfam02055	Glyco_hydro_30,	3,1	0,0	10,4	4,5
Contig_196798	pfam13647	Glyco_hydro_80,	11,4	2,1	0,0	4,5
Contig_355185	pfam00840	Glyco_hydro_7,	4,2	9,3	0,0	4,5
Contig_76653	pfam01055	Glyco_hydro_31,	0,6	2,8	10,0	4,5
Contig_79633	pfam00704	Glyco_hydro_18,	7,5	1,9	4,0	4,5
Contig_111135	pfam02055	Glyco_hydro_30,	8,7	0,7	4,1	4,5
Contig_388701	pfam01229	Glyco_hydro_39,	0,0	13,4	0,0	4,5
Contig_165614	pfam01915	Glyco_hydro_3_C,	2,4	2,6	8,4	4,5
Contig_254218	pfam02435	Glyco_hydro_68,	6,8	6,7	0,0	4,5
Contig_579015	pfam07971	Glyco_hydro_92,	6,4	7,0	0,0	4,5
Contig_228053	pfam07477	Glyco_hydro_67C,	5,9	7,6	0,0	4,5
Contig_261985	pfam03644	Glyco_hydro_85,	6,7	6,7	0,0	4,5
Contig_79912	pfam03644	Glyco_hydro_85,	3,5	1,3	8,6	4,5
Contig_488056	pfam00457	Glyco_hydro_11,	6,9	6,5	0,0	4,5
Contig_81309	pfam02056	Glyco_hydro_4,	5,7	2,9	4,8	4,5
Contig_99359	pfam01055	Glyco_hydro_31,	0,6	4,2	8,6	4,5
Contig_232401	pfam01055	Glyco_hydro_31,	7,0	6,3	0,0	4,5
Contig_119550	pfam00295	Glyco_hydro_28,	8,3	5,0	0,0	4,4
Contig_245649	pfam03632	Glyco_hydro_65m,	8,3	5,0	0,0	4,4
Contig_280053	pfam01055	Glyco_hydro_31,	5,0	8,3	0,0	4,4
Contig_134092	pfam00933	Glyco_hydro_3,	3,8	2,3	7,3	4,4
Contig_135397	pfam01915	Glyco_hydro_3_C,	5,4	4,9	3,1	4,4
Contig_569362	pfam03662	Glyco_hydro_79n,	13,3	0,0	0,0	4,4
Contig_67607	pfam00759	Glyco_hydro_9,	2,7	3,8	6,8	4,4
Contig_123969	pfam02837	Glyco_hydro_2_N,	2,7	1,6	9,0	4,4
Contig_18462	pfam02837	Glyco_hydro_2_N,	1,2	2,1	10,0	4,4
Contig_35438	pfam00722	Glyco_hydro_16,	6,0	7,3	0,0	4,4
Contig_68253	pfam01055	Glyco_hydro_31,	5,3	8,0	0,0	4,4
Contig_404187	pfam02449	Glyco_hydro_42,	3,1	10,2	0,0	4,4
Contig_296993	pfam00232	Glyco_hydro_1,	9,9	3,3	0,0	4,4
Contig_286983	pfam00728	Glyco_hydro_20,	4,7	8,5	0,0	4,4
Contig_441972	pfam07745	Glyco_hydro_53,	5,3	8,0	0,0	4,4
Contig_95162	pfam01055	Glyco_hydro_31,	2,6	0,8	9,8	4,4
Contig_90182	pfam02836	Glyco_hydro_2_C,	6,6	0,0	6,6	4,4
Contig_89779	pfam03065	Glyco_hydro_57,	6,5	4,5	2,3	4,4
Contig_228729	pfam07470	Glyco_hydro_88,	0,0	13,2	0,0	4,4
Contig_221943	pfam03200	Glyco_hydro_63,	8,2	5,0	0,0	4,4
Contig_108606	pfam07971	Glyco_hydro_92,	4,8	3,7	4,7	4,4
Contig_30820	pfam07470	Glyco_hydro_88,	2,5	4,5	6,2	4,4
Contig_101907	pfam00704	Glyco_hydro_18,	3,3	2,7	7,1	4,4
Contig_162300	pfam07488	Glyco_hydro_67M,	3,9	7,9	1,5	4,4
Contig_156261	pfam12899	Glyco_hydro_100,	9,9	3,3	0,0	4,4
Contig_24948	pfam03200	Glyco_hydro_63,	8,8	3,2	1,2	4,4
Contig_5623	pfam03648	Glyco_hydro_67N,	7,9	3,3	2,0	4,4
Contig_121951	pfam12899	Glyco_hydro_100,	3,8	0,0	9,4	4,4
Contig_443065	pfam03644	Glyco_hydro_85,	7,5	5,6	0,0	4,4
Contig_74985	pfam02449	Glyco_hydro_42,	0,0	9,8	3,4	4,4
Contig_94546	pfam12899	Glyco_hydro_100,	10,3	0,0	2,8	4,4
Contig_440179	pfam07971	Glyco_hydro_92,	7,6	5,5	0,0	4,4
Contig_339722	pfam01630	Glyco_hydro_56,	5,9	7,2	0,0	4,4
Contig_543400	pfam00933	Glyco_hydro_3,	13,1	0,0	0,0	4,4
Contig_57505	pfam00704	Glyco_hydro_18,	9,7	1,3	2,1	4,4

Contig_259816	pfam07748	Glyco_hydro_38C,	3,5	7,4	2,2	4,4
Contig_129797	pfam00295	Glyco_hydro_28,	3,1	3,5	6,5	4,4
Contig_348075	pfam00704	Glyco_hydro_18,	2,8	6,8	3,5	4,4
Contig_149855	pfam00703	Glyco_hydro_2,	3,4	4,1	5,6	4,4
Contig_195285	pfam01915	Glyco_hydro_3_C,	6,0	7,0	0,0	4,3
Contig_204474	pfam03662	Glyco_hydro_79n,	7,4	5,6	0,0	4,3
Contig_281642	pfam01074	Glyco_hydro_38,	3,1	10,0	0,0	4,3
Contig_192530	pfam02057	Glyco_hydro_59,	10,4	2,6	0,0	4,3
Contig_77795	pfam00728	Glyco_hydro_20,	0,9	3,2	8,9	4,3
Contig_230965	pfam00295	Glyco_hydro_28,	7,2	5,8	0,0	4,3
Contig_76409	pfam03662	Glyco_hydro_79n,	0,9	6,4	5,7	4,3
Contig_294926	pfam00722	Glyco_hydro_16,	7,7	5,3	0,0	4,3
Contig_444591	pfam00840	Glyco_hydro_7,	13,0	0,0	0,0	4,3
Contig_541864	pfam03639	Glyco_hydro_81,	13,0	0,0	0,0	4,3
Contig_154700	pfam07971	Glyco_hydro_92,	2,7	3,7	6,6	4,3
Contig_133001	pfam00251	Glyco_hydro_32N,	3,2	3,5	6,3	4,3
Contig_225748	pfam03718	Glyco_hydro_49,	1,6	11,3	0,0	4,3
Contig_155306	pfam01915	Glyco_hydro_3_C,	6,8	1,5	4,6	4,3
Contig_464606	pfam12891	Glyco_hydro_44,	2,7	10,2	0,0	4,3
Contig_531802	pfam02446	Glyco_hydro_77,	9,8	0,0	3,1	4,3
Contig_477780	pfam00840	Glyco_hydro_7,	10,4	2,5	0,0	4,3
Contig_493841	pfam00704	Glyco_hydro_18,	11,5	1,4	0,0	4,3
Contig_343626	pfam12891	Glyco_hydro_44,	6,5	6,3	0,0	4,3
Contig_120406	pfam08533	Glyco_hydro_42C,	1,6	3,4	7,9	4,3
Contig_130183	pfam01229	Glyco_hydro_39,	6,0	2,6	4,3	4,3
Contig_118951	pfam00759	Glyco_hydro_9,	2,9	3,8	6,2	4,3
Contig_6549	pfam01532	Glyco_hydro_47,	3,6	2,2	7,1	4,3
Contig_579521	pfam01301	Glyco_hydro_35,	3,7	0,0	9,1	4,3
Contig_307473	pfam01055	Glyco_hydro_31,	9,1	3,7	0,0	4,3
Contig_526041	pfam03443	Glyco_hydro_61,	11,7	1,1	0,0	4,3
Contig_537532	pfam00232	Glyco_hydro_1,	12,4	0,4	0,0	4,3
Contig_200018	pfam01055	Glyco_hydro_31,	4,4	8,4	0,0	4,3
Contig_6000	pfam00704	Glyco_hydro_18,	11,2	1,6	0,0	4,3
Contig_71297	pfam03644	Glyco_hydro_85,	7,9	4,8	0,0	4,2
Contig_110311	pfam00704	Glyco_hydro_18,	0,0	2,5	10,3	4,2
Contig_321332	pfam02015	Glyco_hydro_45,	9,5	1,0	2,2	4,2
Contig_318918	pfam01055	Glyco_hydro_31,	4,9	7,9	0,0	4,2
Contig_381119	pfam03065	Glyco_hydro_57,	0,0	12,7	0,0	4,2
Contig_139890	pfam01270	Glyco_hydro_8,	5,0	5,0	2,7	4,2
Contig_324760	pfam03639	Glyco_hydro_81,	4,5	8,2	0,0	4,2
Contig_290291	pfam01532	Glyco_hydro_47,	11,0	1,7	0,0	4,2
Contig_493823	pfam00723	Glyco_hydro_15,	7,0	5,6	0,0	4,2
Contig_494141	pfam00704	Glyco_hydro_18,	11,0	1,7	0,0	4,2
Contig_87117	pfam07971	Glyco_hydro_92,	3,8	4,0	4,8	4,2
Contig_231078	pfam00295	Glyco_hydro_28,	6,2	6,4	0,0	4,2
Contig_297525	pfam02156	Glyco_hydro_26,	12,6	0,0	0,0	4,2
Contig_381081	pfam00232	Glyco_hydro_1,	6,3	6,3	0,0	4,2
Contig_161728	pfam03639	Glyco_hydro_81,	4,8	7,8	0,0	4,2
Contig_215764	pfam00722	Glyco_hydro_16,	4,8	7,8	0,0	4,2
Contig_276394	pfam00232	Glyco_hydro_1,	4,8	7,8	0,0	4,2
Contig_130311	pfam01055	Glyco_hydro_31,	4,6	1,1	6,9	4,2
Contig_319207	pfam01630	Glyco_hydro_56,	9,6	2,9	0,0	4,2
Contig_369998	pfam07488	Glyco_hydro_67M,	4,6	1,1	6,9	4,2
Contig_87585	pfam00723	Glyco_hydro_15,	11,1	1,5	0,0	4,2
Contig_99407	pfam00759	Glyco_hydro_9,	0,0	0,4	12,2	4,2
Contig_206505	pfam04616	Glyco_hydro_43,	1,9	10,6	0,0	4,2
Contig_335634	pfam01630	Glyco_hydro_56,	4,6	7,9	0,0	4,2
Contig_525082	pfam00232	Glyco_hydro_1,	12,5	0,0	0,0	4,2
Contig_20636	pfam13199	Glyco_hydro_66,	0,0	2,4	10,1	4,2
Contig_304282	pfam03663	Glyco_hydro_76,	7,5	5,0	0,0	4,2
Contig_492259	pfam01532	Glyco_hydro_47,	11,8	0,7	0,0	4,2
Contig_5184	pfam00759	Glyco_hydro_9,	1,0	3,7	7,7	4,2
Contig_114309	pfam02055	Glyco_hydro_30,	0,0	2,9	9,6	4,2
Contig_177752	pfam01270	Glyco_hydro_8,	2,4	10,1	0,0	4,2
Contig_117861	pfam00704	Glyco_hydro_18,	0,0	4,1	8,4	4,2
Contig_382354	pfam03639	Glyco_hydro_81,	8,2	0,8	3,4	4,2
Contig_39582	pfam01055	Glyco_hydro_31,	2,4	2,0	8,1	4,2
Contig_98094	pfam02446	Glyco_hydro_77,	9,0	3,4	0,0	4,2
Contig_166787	pfam02057	Glyco_hydro_59,	5,4	7,1	0,0	4,2
Contig_184713	pfam03659	Glyco_hydro_71,	1,2	5,0	6,3	4,1
Contig_560499	pfam01055	Glyco_hydro_31,	9,3	3,2	0,0	4,1

Contig_78906	pfam00759	Glyco_hydro_9,	1,1	0,0	11,3	4,1
Contig_196247	pfam02837	Glyco_hydro_2_N,	0,0	12,4	0,0	4,1
Contig_332456	pfam07748	Glyco_hydro_38C,	1,1	11,4	0,0	4,1
Contig_433836	pfam01183	Glyco_hydro_25,	6,3	6,1	0,0	4,1
Contig_266266	pfam01229	Glyco_hydro_39,	6,5	5,9	0,0	4,1
Contig_287189	pfam07971	Glyco_hydro_92,	10,2	2,2	0,0	4,1
Contig_164812	pfam01055	Glyco_hydro_31,	3,0	9,4	0,0	4,1
Contig_278342	pfam01183	Glyco_hydro_25,	7,9	4,4	0,0	4,1
Contig_470427	pfam01532	Glyco_hydro_47,	1,9	10,4	0,0	4,1
Contig_8473	pfam03639	Glyco_hydro_81,	3,0	5,0	4,4	4,1
Contig_27058	pfam10566	Glyco_hydro_97,	1,1	5,5	5,7	4,1
Contig_80837	pfam02156	Glyco_hydro_26,	2,4	0,7	9,1	4,1
Contig_483533	pfam00722	Glyco_hydro_16,	4,2	8,1	0,0	4,1
Contig_83778	pfam00251	Glyco_hydro_32N,	6,4	3,2	2,7	4,1
Contig_117813	pfam00704	Glyco_hydro_18,	6,4	5,9	0,0	4,1
Contig_156938	pfam00331	Glyco_hydro_10,	9,9	2,4	0,0	4,1
Contig_180051	pfam03636	Glyco_hydro_65N,	3,7	8,6	0,0	4,1
Contig_134305	pfam03639	Glyco_hydro_81,	5,8	0,7	5,8	4,1
Contig_441223	pfam02156	Glyco_hydro_26,	8,7	3,5	0,0	4,1
Contig_425000	pfam07971	Glyco_hydro_92,	0,6	11,6	0,0	4,1
Contig_537603	pfam02324	Glyco_hydro_70,	8,7	0,0	3,6	4,1
Contig_75478	pfam01270	Glyco_hydro_8,	2,3	9,9	0,0	4,1
Contig_328803	pfam02011	Glyco_hydro_48,	7,1	5,1	0,0	4,1
Contig_550543	pfam00295	Glyco_hydro_28,	11,5	0,7	0,0	4,1
Contig_261818	pfam02449	Glyco_hydro_42,	6,4	5,8	0,0	4,1
Contig_29317	pfam00251	Glyco_hydro_32N,	2,4	3,7	6,0	4,0
Contig_152405	pfam07971	Glyco_hydro_92,	3,2	1,0	8,0	4,0
Contig_502479	pfam00232	Glyco_hydro_1,	5,1	7,0	0,0	4,0
Contig_100848	pfam03639	Glyco_hydro_81,	2,8	5,5	3,9	4,0
Contig_6694	pfam02055	Glyco_hydro_30,	3,4	1,0	7,7	4,0
Contig_236198	pfam02055	Glyco_hydro_30,	5,7	6,4	0,0	4,0
Contig_283780	pfam00840	Glyco_hydro_7,	5,9	6,2	0,0	4,0
Contig_528536	pfam13199	Glyco_hydro_66,	12,1	0,0	0,0	4,0
Contig_449929	pfam00759	Glyco_hydro_9,	3,5	5,4	3,2	4,0
Contig_493538	pfam00232	Glyco_hydro_1,	11,3	0,9	0,0	4,0
Contig_140194	pfam02055	Glyco_hydro_30,	0,0	1,7	10,4	4,0
Contig_11913	pfam02324	Glyco_hydro_70,	0,0	5,5	6,6	4,0
Contig_303769	pfam07745	Glyco_hydro_53,	6,1	5,9	0,0	4,0
Contig_3079	pfam00722	Glyco_hydro_16,	6,3	5,7	0,0	4,0
Contig_581863	pfam04616	Glyco_hydro_43,	7,1	5,0	0,0	4,0
Contig_72867	pfam03718	Glyco_hydro_49,	5,3	3,2	3,6	4,0
Contig_310134	pfam12905	Glyco_hydro_101,	1,3	10,8	0,0	4,0
Contig_318388	pfam02015	Glyco_hydro_45,	8,0	4,0	0,0	4,0
Contig_152532	pfam01915	Glyco_hydro_3_C,	4,7	7,3	0,0	4,0
Contig_289115	pfam12891	Glyco_hydro_44,	2,1	10,0	0,0	4,0
Contig_367390	pfam07488	Glyco_hydro_67M,	10,3	1,7	0,0	4,0
Contig_55985	pfam02837	Glyco_hydro_2_N,	1,7	4,7	5,6	4,0
Contig_289188	pfam00722	Glyco_hydro_16,	7,9	4,1	0,0	4,0
Contig_138759	pfam00722	Glyco_hydro_16,	0,0	1,8	10,1	4,0
Contig_231030	pfam10566	Glyco_hydro_97,	6,3	5,7	0,0	4,0
Contig_126404	pfam02057	Glyco_hydro_59,	0,0	5,0	7,0	4,0
Contig_325958	pfam08307	Glyco_hydro_98C,	5,8	4,4	1,8	4,0
Contig_163601	pfam03659	Glyco_hydro_71,	6,9	5,0	0,0	4,0
Contig_113131	pfam02055	Glyco_hydro_30,	7,0	4,9	0,0	4,0
Contig_41477	pfam08307	Glyco_hydro_98C,	7,0	0,0	4,9	4,0
Contig_133451	pfam02449	Glyco_hydro_42,	1,4	0,0	10,5	4,0
Contig_372034	pfam01074	Glyco_hydro_38,	7,4	4,5	0,0	4,0
Contig_43098	pfam01055	Glyco_hydro_31,	7,1	3,0	1,8	4,0
Contig_482427	pfam01532	Glyco_hydro_47,	4,8	5,8	1,3	4,0
Contig_142791	pfam07477	Glyco_hydro_67C,	4,1	0,0	7,7	4,0
Contig_341795	pfam00295	Glyco_hydro_28,	7,1	4,8	0,0	4,0
Contig_532753	pfam13199	Glyco_hydro_66,	8,2	0,3	3,4	4,0
Contig_279278	pfam03659	Glyco_hydro_71,	9,9	2,0	0,0	3,9
Contig_147843	pfam00331	Glyco_hydro_10,	0,0	4,4	7,4	3,9
Contig_346804	pfam00759	Glyco_hydro_9,	3,7	6,1	2,0	3,9
Contig_247199	pfam00457	Glyco_hydro_11,	7,5	2,6	1,8	3,9
Contig_234922	pfam02057	Glyco_hydro_59,	4,7	7,1	0,0	3,9
Contig_301948	pfam03443	Glyco_hydro_61,	4,7	7,1	0,0	3,9
Contig_365841	pfam07477	Glyco_hydro_67C,	11,2	0,6	0,0	3,9
Contig_243015	pfam03644	Glyco_hydro_85,	7,0	4,8	0,0	3,9
Contig_581423	pfam00295	Glyco_hydro_28,	2,0	3,2	6,6	3,9

Contig_293636	pfam01301	Glyco_hydro_35,	9,8	2,0	0,0	3,9
Contig_23112	pfam04616	Glyco_hydro_43,	0,8	1,0	10,0	3,9
Contig_74155	pfam00722	Glyco_hydro_16,	1,7	8,0	2,1	3,9
Contig_258319	pfam08244	Glyco_hydro_32C,	6,2	5,6	0,0	3,9
Contig_222273	pfam10566	Glyco_hydro_97,	7,1	4,6	0,0	3,9
Contig_227105	pfam01055	Glyco_hydro_31,	6,8	4,9	0,0	3,9
Contig_66231	pfam00704	Glyco_hydro_18,	5,2	0,0	6,5	3,9
Contig_460869	pfam00728	Glyco_hydro_20,	11,4	0,3	0,0	3,9
Contig_105573	pfam00704	Glyco_hydro_18,	6,7	0,5	4,4	3,9
Contig_139482	pfam00840	Glyco_hydro_7,	9,7	2,0	0,0	3,9
Contig_348173	pfam12891	Glyco_hydro_44,	8,3	3,4	0,0	3,9
Contig_386377	pfam03662	Glyco_hydro_79n,	0,0	11,7	0,0	3,9
Contig_277273	pfam07971	Glyco_hydro_92,	4,3	7,3	0,0	3,9
Contig_314974	pfam02055	Glyco_hydro_30,	10,2	1,4	0,0	3,9
Contig_248751	pfam00728	Glyco_hydro_20,	9,4	2,3	0,0	3,9
Contig_448633	pfam10566	Glyco_hydro_97,	6,4	5,3	0,0	3,9
Contig_322370	pfam07488	Glyco_hydro_67M,	3,9	7,8	0,0	3,9
Contig_447321	pfam01532	Glyco_hydro_47,	8,1	3,5	0,0	3,9
Contig_188403	pfam00840	Glyco_hydro_7,	5,5	6,1	0,0	3,9
Contig_217341	pfam01074	Glyco_hydro_38,	5,9	1,5	4,2	3,9
Contig_78731	pfam10566	Glyco_hydro_97,	2,5	3,0	6,2	3,9
Contig_460909	pfam03198	Glyco_hydro_72,	8,6	3,0	0,0	3,9
Contig_266246	pfam07488	Glyco_hydro_67M,	2,5	9,1	0,0	3,9
Contig_338883	pfam03639	Glyco_hydro_81,	4,0	7,6	0,0	3,9
Contig_94062	pfam00704	Glyco_hydro_18,	5,9	0,8	4,9	3,8
Contig_110786	pfam03639	Glyco_hydro_81,	7,7	0,0	3,9	3,8
Contig_496367	pfam07971	Glyco_hydro_92,	11,5	0,0	0,0	3,8
Contig_402748	pfam07971	Glyco_hydro_92,	11,5	0,0	0,0	3,8
Contig_110785	pfam12899	Glyco_hydro_100,	0,0	6,8	4,7	3,8
Contig_302973	pfam00933	Glyco_hydro_3,	10,0	1,5	0,0	3,8
Contig_416609	pfam13199	Glyco_hydro_66,	7,6	3,8	0,0	3,8
Contig_11524	pfam03065	Glyco_hydro_57,	4,4	3,4	3,7	3,8
Contig_51179	pfam01532	Glyco_hydro_47,	2,6	5,6	3,3	3,8
Contig_300558	pfam00232	Glyco_hydro_1,	3,4	8,1	0,0	3,8
Contig_447166	pfam07748	Glyco_hydro_38C,	7,2	4,3	0,0	3,8
Contig_84107	pfam00728	Glyco_hydro_20,	3,8	2,9	4,8	3,8
Contig_470456	pfam07971	Glyco_hydro_92,	2,9	8,5	0,0	3,8
Contig_251371	pfam01532	Glyco_hydro_47,	4,6	6,9	0,0	3,8
Contig_399435	pfam08307	Glyco_hydro_98C,	7,1	4,3	0,0	3,8
Contig_407756	pfam00722	Glyco_hydro_16,	1,4	10,0	0,0	3,8
Contig_564891	pfam00722	Glyco_hydro_16,	11,4	0,0	0,0	3,8
Contig_105146	pfam00722	Glyco_hydro_16,	2,7	8,7	0,0	3,8
Contig_121454	pfam01055	Glyco_hydro_31,	11,4	0,0	0,0	3,8
Contig_380821	pfam00232	Glyco_hydro_1,	9,0	2,4	0,0	3,8
Contig_517096	pfam00723	Glyco_hydro_15,	11,4	0,0	0,0	3,8
Contig_39044	pfam01183	Glyco_hydro_25,	5,7	5,6	0,0	3,8
Contig_413047	pfam00759	Glyco_hydro_9,	2,7	8,7	0,0	3,8
Contig_128577	pfam01270	Glyco_hydro_8,	0,6	2,6	8,2	3,8
Contig_367109	pfam00232	Glyco_hydro_1,	1,1	10,2	0,0	3,8
Contig_156165	pfam03200	Glyco_hydro_63,	2,3	5,2	3,9	3,8
Contig_450618	pfam04616	Glyco_hydro_43,	3,1	8,2	0,0	3,8
Contig_457754	pfam01532	Glyco_hydro_47,	10,8	0,5	0,0	3,8
Contig_153377	pfam03632	Glyco_hydro_65m,	6,2	1,3	3,9	3,8
Contig_494461	pfam03443	Glyco_hydro_61,	10,5	0,8	0,0	3,8
Contig_304010	pfam03718	Glyco_hydro_49,	0,0	11,3	0,0	3,8
Contig_298970	pfam02057	Glyco_hydro_59,	0,5	5,8	5,0	3,8
Contig_87000	pfam01183	Glyco_hydro_25,	0,5	10,8	0,0	3,8
Contig_109814	pfam03200	Glyco_hydro_63,	0,0	1,6	9,7	3,8
Contig_244740	pfam01532	Glyco_hydro_47,	4,3	7,0	0,0	3,8
Contig_570183	pfam02836	Glyco_hydro_2_C,	8,9	2,4	0,0	3,8
Contig_141136	pfam03659	Glyco_hydro_71,	4,5	0,0	6,8	3,8
Contig_318983	pfam07748	Glyco_hydro_38C,	4,8	6,5	0,0	3,8
Contig_139064	pfam02836	Glyco_hydro_2_C,	6,0	3,1	2,1	3,8
Contig_34787	pfam01532	Glyco_hydro_47,	9,0	2,3	0,0	3,8
Contig_125447	pfam00232	Glyco_hydro_1,	3,7	3,4	4,2	3,8
Contig_503683	pfam02011	Glyco_hydro_48,	1,1	4,7	5,5	3,8
Contig_403777	pfam03632	Glyco_hydro_65m,	4,8	6,4	0,0	3,8
Contig_548405	pfam03200	Glyco_hydro_63,	11,3	0,0	0,0	3,8
Contig_428918	pfam03065	Glyco_hydro_57,	10,0	1,3	0,0	3,7
Contig_564271	pfam00840	Glyco_hydro_7,	11,2	0,0	0,0	3,7
Contig_265508	pfam02836	Glyco_hydro_2_C,	5,9	5,3	0,0	3,7

Contig_350083	pfam01532	Glyco_hydro_47,	9,2	2,0	0,0	3,7
Contig_279400	pfam00251	Glyco_hydro_32N,	10,3	0,9	0,0	3,7
Contig_314661	pfam03639	Glyco_hydro_81,	0,0	11,2	0,0	3,7
Contig_377894	pfam00331	Glyco_hydro_10,	9,3	1,9	0,0	3,7
Contig_366525	pfam00722	Glyco_hydro_16,	4,4	6,8	0,0	3,7
Contig_87336	pfam00331	Glyco_hydro_10,	11,2	0,0	0,0	3,7
Contig_391737	pfam07488	Glyco_hydro_67M,	4,3	6,9	0,0	3,7
Contig_335589	pfam00933	Glyco_hydro_3,	7,6	3,6	0,0	3,7
Contig_3081	pfam12899	Glyco_hydro_100,	0,0	2,9	8,2	3,7
Contig_37133	pfam12899	Glyco_hydro_100,	2,8	3,0	5,3	3,7
Contig_309627	pfam04616	Glyco_hydro_43,	5,8	5,3	0,0	3,7
Contig_385876	pfam03200	Glyco_hydro_63,	7,4	3,7	0,0	3,7
Contig_492598	pfam00232	Glyco_hydro_1,	10,5	0,6	0,0	3,7
Contig_307749	pfam01301	Glyco_hydro_35,	8,0	3,1	0,0	3,7
Contig_419878	pfam00722	Glyco_hydro_16,	9,2	1,9	0,0	3,7
Contig_273505	pfam02055	Glyco_hydro_30,	1,4	9,7	0,0	3,7
Contig_306284	pfam03636	Glyco_hydro_65N,	8,2	2,8	0,0	3,7
Contig_378998	pfam05838	Glyco_hydro_108,	8,5	2,6	0,0	3,7
Contig_170566	pfam12905	Glyco_hydro_101,	4,5	6,6	0,0	3,7
Contig_75247	pfam11790	Glyco_hydro_cc,	7,8	0,0	3,2	3,7
Contig_85315	pfam01055	Glyco_hydro_31,	1,9	4,5	4,7	3,7
Contig_277032	pfam00728	Glyco_hydro_20,	3,2	7,8	0,0	3,7
Contig_493013	pfam12899	Glyco_hydro_100,	6,9	4,2	0,0	3,7
Contig_573676	pfam03200	Glyco_hydro_63,	11,0	0,0	0,0	3,7
Contig_50656	pfam03639	Glyco_hydro_81,	2,2	3,3	5,5	3,7
Contig_129918	pfam02837	Glyco_hydro_2_N,	7,2	1,2	2,6	3,7
Contig_379752	pfam12891	Glyco_hydro_44,	5,0	6,0	0,0	3,7
Contig_534170	pfam01532	Glyco_hydro_47,	11,0	0,0	0,0	3,7
Contig_308936	pfam07745	Glyco_hydro_53,	5,3	5,7	0,0	3,7
Contig_86681	pfam01532	Glyco_hydro_47,	4,3	6,7	0,0	3,7
Contig_173685	pfam02838	Glyco_hydro_20b,	10,0	1,0	0,0	3,7
Contig_203638	pfam02055	Glyco_hydro_30,	10,0	1,0	0,0	3,7
Contig_493334	pfam00759	Glyco_hydro_9,	4,2	3,4	3,5	3,7
Contig_514046	pfam00232	Glyco_hydro_1,	11,0	0,0	0,0	3,7
Contig_328139	pfam01074	Glyco_hydro_38,	1,2	9,7	0,0	3,7
Contig_476300	pfam00704	Glyco_hydro_18,	10,0	0,9	0,0	3,7
Contig_323897	pfam03065	Glyco_hydro_57,	0,0	11,0	0,0	3,7
Contig_430223	pfam01301	Glyco_hydro_35,	7,2	1,2	2,6	3,7
Contig_101563	pfam01055	Glyco_hydro_31,	7,8	3,2	0,0	3,6
Contig_125822	pfam00295	Glyco_hydro_28,	5,0	5,2	0,7	3,6
Contig_127209	pfam12899	Glyco_hydro_100,	1,3	0,0	9,7	3,6
Contig_251300	pfam01630	Glyco_hydro_56,	1,1	9,8	0,0	3,6
Contig_434758	pfam00182	Glyco_hydro_19,	3,1	0,0	7,8	3,6
Contig_434927	pfam02837	Glyco_hydro_2_N,	7,4	3,6	0,0	3,6
Contig_350406	pfam02156	Glyco_hydro_26,	5,7	5,2	0,0	3,6
Contig_182891	pfam01055	Glyco_hydro_31,	3,6	7,3	0,0	3,6
Contig_266395	pfam00728	Glyco_hydro_20,	7,6	3,3	0,0	3,6
Contig_337576	pfam00704	Glyco_hydro_18,	6,5	3,5	0,9	3,6
Contig_8793	pfam00704	Glyco_hydro_18,	0,7	0,0	10,2	3,6
Contig_350436	pfam00232	Glyco_hydro_1,	8,4	2,5	0,0	3,6
Contig_12170	pfam01074	Glyco_hydro_38,	1,5	0,4	9,0	3,6
Contig_128711	pfam01074	Glyco_hydro_38,	0,0	2,1	8,8	3,6
Contig_234798	pfam00759	Glyco_hydro_9,	10,9	0,0	0,0	3,6
Contig_321284	pfam01183	Glyco_hydro_25,	10,9	0,0	0,0	3,6
Contig_491736	pfam01301	Glyco_hydro_35,	10,9	0,0	0,0	3,6
Contig_514504	pfam03663	Glyco_hydro_76,	10,9	0,0	0,0	3,6
Contig_546007	pfam03659	Glyco_hydro_71,	10,9	0,0	0,0	3,6
Contig_534177	pfam00704	Glyco_hydro_18,	10,3	0,6	0,0	3,6
Contig_559416	pfam00759	Glyco_hydro_9,	10,0	0,9	0,0	3,6
Contig_10281	pfam03644	Glyco_hydro_85,	9,3	1,6	0,0	3,6
Contig_72777	pfam03639	Glyco_hydro_81,	0,7	1,7	8,5	3,6
Contig_283507	pfam00728	Glyco_hydro_20,	2,0	8,9	0,0	3,6
Contig_322978	pfam00728	Glyco_hydro_20,	7,1	3,8	0,0	3,6
Contig_220472	pfam00933	Glyco_hydro_3,	1,1	4,8	4,9	3,6
Contig_78739	pfam13199	Glyco_hydro_66,	0,5	2,8	7,5	3,6
Contig_305790	pfam00759	Glyco_hydro_9,	3,1	5,1	2,6	3,6
Contig_268063	pfam02449	Glyco_hydro_42,	7,3	3,5	0,0	3,6
Contig_566995	pfam00728	Glyco_hydro_20,	10,8	0,0	0,0	3,6
Contig_149124	pfam00295	Glyco_hydro_28,	3,4	6,1	1,4	3,6
Contig_91437	pfam01532	Glyco_hydro_47,	0,0	2,9	7,9	3,6
Contig_360999	pfam00332	Glyco_hydro_17,	4,5	6,3	0,0	3,6

Contig_359759	pfam00704	Glyco_hydro_18,	9,7	1,0	0,0	3,6
Contig_135163	pfam07971	Glyco_hydro_92,	6,8	3,9	0,0	3,6
Contig_216966	pfam08244	Glyco_hydro_32C,	3,4	4,4	3,0	3,6
Contig_275988	pfam01670	Glyco_hydro_12,	10,1	0,6	0,0	3,6
Contig_543618	pfam04616	Glyco_hydro_43,	9,6	1,1	0,0	3,6
Contig_454643	pfam03200	Glyco_hydro_63,	6,2	4,5	0,0	3,6
Contig_201419	pfam03662	Glyco_hydro_79n,	2,3	8,4	0,0	3,6
Contig_401538	pfam02837	Glyco_hydro_2_N,	7,5	3,2	0,0	3,6
Contig_171358	pfam04616	Glyco_hydro_43,	5,1	5,6	0,0	3,6
Contig_257980	pfam02324	Glyco_hydro_70,	4,7	6,0	0,0	3,6
Contig_399931	pfam03662	Glyco_hydro_79n,	5,1	5,6	0,0	3,6
Contig_208063	pfam00704	Glyco_hydro_18,	3,6	7,1	0,0	3,6
Contig_338305	pfam00251	Glyco_hydro_32N,	3,3	2,0	5,4	3,6
Contig_126684	pfam02837	Glyco_hydro_2_N,	3,8	4,8	2,0	3,6
Contig_89729	pfam01341	Glyco_hydro_6,	1,4	2,5	6,8	3,5
Contig_61354	pfam13199	Glyco_hydro_66,	7,1	0,0	3,6	3,5
Contig_477990	pfam02324	Glyco_hydro_70,	9,5	1,1	0,0	3,5
Contig_491832	pfam03633	Glyco_hydro_65C,	8,8	1,8	0,0	3,5
Contig_499935	pfam03663	Glyco_hydro_76,	2,9	7,8	0,0	3,5
Contig_573262	pfam02324	Glyco_hydro_70,	8,2	2,5	0,0	3,5
Contig_344724	pfam00728	Glyco_hydro_20,	9,2	1,4	0,0	3,5
Contig_140792	pfam00331	Glyco_hydro_10,	0,0	2,1	8,5	3,5
Contig_480197	pfam07470	Glyco_hydro_88,	8,8	1,8	0,0	3,5
Contig_313074	pfam07748	Glyco_hydro_38C,	7,1	3,5	0,0	3,5
Contig_331733	pfam01301	Glyco_hydro_35,	9,9	0,7	0,0	3,5
Contig_317357	pfam00232	Glyco_hydro_1,	9,2	1,4	0,0	3,5
Contig_383621	pfam01055	Glyco_hydro_31,	4,4	4,3	2,0	3,5
Contig_105788	pfam01301	Glyco_hydro_35,	3,6	5,4	1,6	3,5
Contig_136663	pfam02449	Glyco_hydro_42,	6,2	0,0	4,4	3,5
Contig_145394	pfam00759	Glyco_hydro_9,	2,9	2,2	5,4	3,5
Contig_268572	pfam00332	Glyco_hydro_17,	10,6	0,0	0,0	3,5
Contig_311771	pfam00232	Glyco_hydro_1,	7,0	3,6	0,0	3,5
Contig_33376	pfam01532	Glyco_hydro_47,	10,6	0,0	0,0	3,5
Contig_361546	pfam00933	Glyco_hydro_3,	5,3	5,2	0,0	3,5
Contig_218177	pfam01055	Glyco_hydro_31,	0,0	10,6	0,0	3,5
Contig_290410	pfam08244	Glyco_hydro_32C,	6,6	4,0	0,0	3,5
Contig_572182	pfam03659	Glyco_hydro_71,	10,0	0,6	0,0	3,5
Contig_414199	pfam00295	Glyco_hydro_28,	4,8	5,8	0,0	3,5
Contig_560497	pfam03443	Glyco_hydro_61,	10,5	0,0	0,0	3,5
Contig_583109	pfam03200	Glyco_hydro_63,	10,5	0,0	0,0	3,5
Contig_135808	pfam03198	Glyco_hydro_72,	2,7	5,9	1,9	3,5
Contig_491820	pfam03644	Glyco_hydro_85,	6,1	4,4	0,0	3,5
Contig_338863	pfam03639	Glyco_hydro_81,	6,9	3,6	0,0	3,5
Contig_418649	pfam01229	Glyco_hydro_39,	4,8	5,7	0,0	3,5
Contig_267541	pfam01301	Glyco_hydro_35,	4,9	5,6	0,0	3,5
Contig_93851	pfam08532	Glyco_hydro_42M,	1,8	3,2	5,5	3,5
Contig_275891	pfam02055	Glyco_hydro_30,	7,8	2,0	0,7	3,5
Contig_335701	pfam00295	Glyco_hydro_28,	2,1	6,7	1,7	3,5
Contig_399005	pfam03200	Glyco_hydro_63,	0,0	10,5	0,0	3,5
Contig_42995	pfam02449	Glyco_hydro_42,	2,2	2,7	5,5	3,5
Contig_49862	pfam00704	Glyco_hydro_18,	10,4	0,0	0,0	3,5
Contig_559371	pfam13199	Glyco_hydro_66,	10,4	0,0	0,0	3,5
Contig_242599	pfam07971	Glyco_hydro_92,	5,0	5,4	0,0	3,5
Contig_170910	pfam10566	Glyco_hydro_97,	2,2	8,3	0,0	3,5
Contig_356774	pfam01055	Glyco_hydro_31,	4,3	6,1	0,0	3,5
Contig_507922	pfam02435	Glyco_hydro_68,	5,7	4,8	0,0	3,5
Contig_537082	pfam12899	Glyco_hydro_100,	10,4	0,0	0,0	3,5
Contig_188819	pfam01055	Glyco_hydro_31,	5,8	3,1	1,6	3,5
Contig_344813	pfam13647	Glyco_hydro_80,	1,8	8,6	0,0	3,5
Contig_582571	pfam03639	Glyco_hydro_81,	8,7	1,7	0,0	3,5
Contig_205661	pfam02446	Glyco_hydro_77,	0,0	10,4	0,0	3,5
Contig_238646	pfam01074	Glyco_hydro_38,	6,7	3,6	0,0	3,5
Contig_410018	pfam03200	Glyco_hydro_63,	5,8	0,9	3,7	3,5
Contig_255234	pfam01630	Glyco_hydro_56,	7,6	2,8	0,0	3,5
Contig_286473	pfam01055	Glyco_hydro_31,	5,3	5,1	0,0	3,5
Contig_145349	pfam03659	Glyco_hydro_71,	0,9	1,0	8,5	3,4
Contig_45467	pfam01630	Glyco_hydro_56,	1,3	2,4	6,6	3,4
Contig_320292	pfam00295	Glyco_hydro_28,	1,2	9,2	0,0	3,4
Contig_292555	pfam00723	Glyco_hydro_15,	2,0	8,3	0,0	3,4
Contig_546937	pfam03632	Glyco_hydro_65m,	6,0	4,3	0,0	3,4
Contig_64609	pfam00728	Glyco_hydro_20,	0,0	1,0	9,3	3,4

Contig_396321	pfam00704	Glyco_hydro_18,	7,0	3,4	0,0	3,4
Contig_65801	pfam00331	Glyco_hydro_10,	4,9	3,4	2,0	3,4
Contig_579182	pfam01630	Glyco_hydro_56,	7,9	0,0	2,4	3,4
Contig_203168	pfam07745	Glyco_hydro_53,	5,3	4,9	0,0	3,4
Contig_349738	pfam00295	Glyco_hydro_28,	3,5	4,7	2,1	3,4
Contig_47313	pfam00728	Glyco_hydro_20,	1,3	6,3	2,6	3,4
Contig_232912	pfam05838	Glyco_hydro_108,	8,5	1,7	0,0	3,4
Contig_176389	pfam01532	Glyco_hydro_47,	3,3	7,0	0,0	3,4
Contig_351795	pfam10566	Glyco_hydro_97,	1,0	4,2	5,0	3,4
Contig_544050	pfam01301	Glyco_hydro_35,	8,2	2,0	0,0	3,4
Contig_411675	pfam07488	Glyco_hydro_67M,	9,8	0,4	0,0	3,4
Contig_435144	pfam12891	Glyco_hydro_44,	0,0	2,0	8,2	3,4
Contig_576888	pfam03633	Glyco_hydro_65C,	9,6	0,6	0,0	3,4
Contig_3008	pfam03198	Glyco_hydro_72,	6,0	0,0	4,2	3,4
Contig_128828	pfam08532	Glyco_hydro_42M,	0,6	5,6	4,1	3,4
Contig_318172	pfam00251	Glyco_hydro_32N,	9,4	0,8	0,0	3,4
Contig_327938	pfam01229	Glyco_hydro_39,	4,6	5,6	0,0	3,4
Contig_49948	pfam03512	Glyco_hydro_52,	0,0	0,5	9,7	3,4
Contig_334863	pfam01055	Glyco_hydro_31,	8,7	1,4	0,0	3,4
Contig_492359	pfam00703	Glyco_hydro_2,	9,0	1,2	0,0	3,4
Contig_248136	pfam00332	Glyco_hydro_17,	2,9	7,3	0,0	3,4
Contig_353405	pfam12905	Glyco_hydro_101,	3,9	6,3	0,0	3,4
Contig_215040	pfam07971	Glyco_hydro_92,	2,6	7,5	0,0	3,4
Contig_339563	pfam10566	Glyco_hydro_97,	0,0	10,1	0,0	3,4
Contig_466957	pfam08244	Glyco_hydro_32C,	3,8	6,4	0,0	3,4
Contig_70565	pfam01373	Glyco_hydro_14,	3,3	1,3	5,5	3,4
Contig_146035	pfam00704	Glyco_hydro_18,	7,6	0,0	2,6	3,4
Contig_88912	pfam00704	Glyco_hydro_18,	2,9	0,0	7,2	3,4
Contig_185346	pfam02056	Glyco_hydro_4,	5,0	5,2	0,0	3,4
Contig_506510	pfam01074	Glyco_hydro_38,	3,3	6,9	0,0	3,4
Contig_376640	pfam03644	Glyco_hydro_85,	2,0	8,1	0,0	3,4
Contig_403667	pfam11790	Glyco_hydro_cc,	9,5	0,7	0,0	3,4
Contig_519221	pfam03200	Glyco_hydro_63,	10,1	0,0	0,0	3,4
Contig_236754	pfam01055	Glyco_hydro_31,	7,0	1,8	1,2	3,4
Contig_377620	pfam07971	Glyco_hydro_92,	8,9	1,2	0,0	3,4
Contig_365242	pfam03065	Glyco_hydro_57,	2,8	7,3	0,0	3,4
Contig_435605	pfam00295	Glyco_hydro_28,	4,6	5,5	0,0	3,4
Contig_32583	pfam03200	Glyco_hydro_63,	4,0	0,5	5,5	3,4
Contig_310943	pfam03065	Glyco_hydro_57,	4,9	4,2	0,9	3,4
Contig_382337	pfam08532	Glyco_hydro_42M,	7,4	2,7	0,0	3,3
Contig_156391	pfam12899	Glyco_hydro_100,	5,3	4,8	0,0	3,3
Contig_538630	pfam00759	Glyco_hydro_9,	10,0	0,0	0,0	3,3
Contig_111149	pfam00728	Glyco_hydro_20,	1,8	1,6	6,6	3,3
Contig_83351	pfam04616	Glyco_hydro_43,	1,0	1,8	7,3	3,3
Contig_313782	pfam01074	Glyco_hydro_38,	5,0	5,0	0,0	3,3
Contig_378774	pfam02449	Glyco_hydro_42,	8,5	1,5	0,0	3,3
Contig_368997	pfam00332	Glyco_hydro_17,	9,4	0,6	0,0	3,3
Contig_519137	pfam01055	Glyco_hydro_31,	7,1	2,9	0,0	3,3
Contig_100033	pfam07488	Glyco_hydro_67M,	1,0	1,5	7,5	3,3
Contig_152419	pfam00704	Glyco_hydro_18,	3,1	3,7	3,2	3,3
Contig_340555	pfam01301	Glyco_hydro_35,	5,8	4,2	0,0	3,3
Contig_152956	pfam01532	Glyco_hydro_47,	1,5	4,6	3,8	3,3
Contig_283755	pfam00728	Glyco_hydro_20,	8,1	1,8	0,0	3,3
Contig_229223	pfam02057	Glyco_hydro_59,	5,4	4,5	0,0	3,3
Contig_367931	pfam03512	Glyco_hydro_52,	2,7	2,7	4,5	3,3
Contig_71289	pfam03065	Glyco_hydro_57,	1,8	3,8	4,4	3,3
Contig_343975	pfam02837	Glyco_hydro_2_N,	4,1	5,8	0,0	3,3
Contig_477942	pfam12899	Glyco_hydro_100,	1,8	8,1	0,0	3,3
Contig_143361	pfam01183	Glyco_hydro_25,	0,4	2,1	7,5	3,3
Contig_276534	pfam03644	Glyco_hydro_85,	0,0	9,9	0,0	3,3
Contig_153676	pfam03198	Glyco_hydro_72,	5,6	4,3	0,0	3,3
Contig_282089	pfam03644	Glyco_hydro_85,	8,1	1,8	0,0	3,3
Contig_42305	pfam03200	Glyco_hydro_63,	0,0	1,9	8,0	3,3
Contig_306188	pfam00840	Glyco_hydro_7,	4,4	1,8	3,7	3,3
Contig_567884	pfam02011	Glyco_hydro_48,	4,9	5,0	0,0	3,3
Contig_502672	pfam11790	Glyco_hydro_cc,	7,0	2,9	0,0	3,3
Contig_249010	pfam03200	Glyco_hydro_63,	5,2	4,7	0,0	3,3
Contig_308662	pfam07971	Glyco_hydro_92,	0,0	9,9	0,0	3,3
Contig_439008	pfam03718	Glyco_hydro_49,	2,1	7,7	0,0	3,3
Contig_351766	pfam03198	Glyco_hydro_72,	9,9	0,0	0,0	3,3
Contig_286539	pfam02449	Glyco_hydro_42,	4,5	5,4	0,0	3,3

Contig_349932	pfam11790	Glyco_hydro_cc,	3,9	3,5	2,4	3,3
Contig_232397	pfam00728	Glyco_hydro_20,	7,2	2,7	0,0	3,3
Contig_1284	pfam00232	Glyco_hydro_1,	4,0	2,2	3,6	3,3
Contig_139782	pfam00722	Glyco_hydro_16,	0,0	4,1	5,7	3,3
Contig_242013	pfam01915	Glyco_hydro_3_C,	3,7	6,1	0,0	3,3
Contig_545803	pfam01074	Glyco_hydro_38,	9,8	0,0	0,0	3,3
Contig_89093	pfam07748	Glyco_hydro_38C,	3,0	3,9	2,9	3,3
Contig_418095	pfam01341	Glyco_hydro_6,	9,8	0,0	0,0	3,3
Contig_280869	pfam01915	Glyco_hydro_3_C,	1,6	6,3	1,9	3,3
Contig_48585	pfam01373	Glyco_hydro_14,	2,9	1,3	5,5	3,3
Contig_71643	pfam01055	Glyco_hydro_31,	0,0	4,4	5,4	3,3
Contig_231981	pfam02837	Glyco_hydro_2_N,	0,0	9,8	0,0	3,3
Contig_490304	pfam02836	Glyco_hydro_2_C,	4,4	5,3	0,0	3,3
Contig_404967	pfam07335	Glyco_hydro_75,	5,7	4,1	0,0	3,2
Contig_201847	pfam02156	Glyco_hydro_26,	4,6	5,1	0,0	3,2
Contig_509170	pfam03633	Glyco_hydro_65C,	7,1	2,6	0,0	3,2
Contig_16996	pfam04616	Glyco_hydro_43,	5,3	4,5	0,0	3,2
Contig_412853	pfam01373	Glyco_hydro_14,	9,7	0,0	0,0	3,2
Contig_334421	pfam12899	Glyco_hydro_100,	5,1	4,6	0,0	3,2
Contig_551241	pfam01532	Glyco_hydro_47,	9,7	0,0	0,0	3,2
Contig_557357	pfam04616	Glyco_hydro_43,	9,7	0,0	0,0	3,2
Contig_213375	pfam00704	Glyco_hydro_18,	2,8	3,4	3,5	3,2
Contig_578032	pfam07971	Glyco_hydro_92,	9,7	0,0	0,0	3,2
Contig_198728	pfam02449	Glyco_hydro_42,	2,8	6,8	0,0	3,2
Contig_92087	pfam03065	Glyco_hydro_57,	0,5	1,5	7,6	3,2
Contig_313494	pfam07971	Glyco_hydro_92,	4,8	4,9	0,0	3,2
Contig_430665	pfam03718	Glyco_hydro_49,	8,2	1,5	0,0	3,2
Contig_210021	pfam00933	Glyco_hydro_3,	3,0	6,6	0,0	3,2
Contig_162029	pfam00728	Glyco_hydro_20,	6,8	2,8	0,0	3,2
Contig_122213	pfam00728	Glyco_hydro_20,	3,5	3,2	2,9	3,2
Contig_360645	pfam02056	Glyco_hydro_4,	0,0	9,6	0,0	3,2
Contig_161052	pfam00332	Glyco_hydro_17,	1,3	5,1	3,2	3,2
Contig_236058	pfam13199	Glyco_hydro_66,	2,0	7,6	0,0	3,2
Contig_303330	pfam01055	Glyco_hydro_31,	0,0	9,6	0,0	3,2
Contig_331753	pfam07470	Glyco_hydro_88,	0,0	9,6	0,0	3,2
Contig_387701	pfam02836	Glyco_hydro_2_C,	6,6	3,0	0,0	3,2
Contig_240438	pfam00704	Glyco_hydro_18,	9,4	0,2	0,0	3,2
Contig_158344	pfam00251	Glyco_hydro_32N,	5,0	4,5	0,0	3,2
Contig_232786	pfam01915	Glyco_hydro_3_C,	2,3	7,3	0,0	3,2
Contig_319440	pfam02449	Glyco_hydro_42,	2,4	7,2	0,0	3,2
Contig_180999	pfam03644	Glyco_hydro_85,	5,4	4,1	0,0	3,2
Contig_349689	pfam12899	Glyco_hydro_100,	5,0	4,5	0,0	3,2
Contig_535698	pfam00704	Glyco_hydro_18,	9,5	0,0	0,0	3,2
Contig_556394	pfam01532	Glyco_hydro_47,	8,7	0,9	0,0	3,2
Contig_303022	pfam00182	Glyco_hydro_19,	2,3	7,2	0,0	3,2
Contig_329886	pfam01532	Glyco_hydro_47,	4,3	5,2	0,0	3,2
Contig_30661	pfam03632	Glyco_hydro_65m,	8,3	1,2	0,0	3,2
Contig_135316	pfam00728	Glyco_hydro_20,	3,5	6,0	0,0	3,2
Contig_327141	pfam03636	Glyco_hydro_65N,	0,0	9,5	0,0	3,2
Contig_400249	pfam02057	Glyco_hydro_59,	9,5	0,0	0,0	3,2
Contig_152426	pfam07745	Glyco_hydro_53,	5,7	0,5	3,3	3,2
Contig_357348	pfam01915	Glyco_hydro_3_C,	2,6	6,9	0,0	3,2
Contig_344262	pfam10566	Glyco_hydro_97,	2,6	6,8	0,0	3,2
Contig_362278	pfam00232	Glyco_hydro_1,	5,9	3,6	0,0	3,2
Contig_83881	pfam00704	Glyco_hydro_18,	1,6	4,7	3,2	3,1
Contig_403334	pfam01915	Glyco_hydro_3_C,	5,4	4,1	0,0	3,1
Contig_113040	pfam10566	Glyco_hydro_97,	0,0	2,3	7,1	3,1
Contig_216670	pfam00331	Glyco_hydro_10,	1,9	7,5	0,0	3,1
Contig_241400	pfam00704	Glyco_hydro_18,	6,1	3,3	0,0	3,1
Contig_303869	pfam03512	Glyco_hydro_52,	5,2	4,2	0,0	3,1
Contig_506248	pfam03659	Glyco_hydro_71,	8,6	0,9	0,0	3,1
Contig_564363	pfam07470	Glyco_hydro_88,	9,4	0,0	0,0	3,1
Contig_283654	pfam00182	Glyco_hydro_19,	2,9	1,7	4,8	3,1
Contig_168153	pfam02449	Glyco_hydro_42,	1,3	8,1	0,0	3,1
Contig_319856	pfam07745	Glyco_hydro_53,	2,3	7,0	0,0	3,1
Contig_466871	pfam00295	Glyco_hydro_28,	2,0	7,4	0,0	3,1
Contig_361120	pfam00723	Glyco_hydro_15,	2,5	6,9	0,0	3,1
Contig_153419	pfam04616	Glyco_hydro_43,	3,1	5,0	1,3	3,1
Contig_179331	pfam01229	Glyco_hydro_39,	5,8	3,5	0,0	3,1
Contig_579973	pfam01630	Glyco_hydro_56,	9,4	0,0	0,0	3,1
Contig_515068	pfam03663	Glyco_hydro_76,	4,7	4,7	0,0	3,1

Contig_110280	pfam00704	Glyco_hydro_18,	8,1	0,0	1,3	3,1
Contig_470915	pfam00332	Glyco_hydro_17,	5,3	4,0	0,0	3,1
Contig_353030	pfam00840	Glyco_hydro_7,	7,2	2,2	0,0	3,1
Contig_509103	pfam00728	Glyco_hydro_20,	9,3	0,0	0,0	3,1
Contig_548458	pfam03198	Glyco_hydro_72,	9,3	0,0	0,0	3,1
Contig_566232	pfam03443	Glyco_hydro_61,	9,3	0,0	0,0	3,1
Contig_284958	pfam07488	Glyco_hydro_67M,	1,3	8,0	0,0	3,1
Contig_299072	pfam00759	Glyco_hydro_9,	2,7	6,6	0,0	3,1
Contig_349686	pfam01074	Glyco_hydro_38,	1,4	7,8	0,0	3,1
Contig_165495	pfam07477	Glyco_hydro_67C,	6,3	3,0	0,0	3,1
Contig_174986	pfam00759	Glyco_hydro_9,	3,0	6,3	0,0	3,1
Contig_195681	pfam00251	Glyco_hydro_32N,	4,2	5,1	0,0	3,1
Contig_405861	pfam01301	Glyco_hydro_35,	4,8	4,0	0,5	3,1
Contig_199904	pfam07488	Glyco_hydro_67M,	0,0	6,1	3,2	3,1
Contig_453944	pfam11975	Glyco_hydro_4C,	8,4	0,8	0,0	3,1
Contig_177042	pfam03664	Glyco_hydro_62,	4,6	4,6	0,0	3,1
Contig_367476	pfam07488	Glyco_hydro_67M,	4,0	5,2	0,0	3,1
Contig_524135	pfam03644	Glyco_hydro_85,	5,1	0,0	4,2	3,1
Contig_217161	pfam03636	Glyco_hydro_65N,	5,4	3,9	0,0	3,1
Contig_502026	pfam01915	Glyco_hydro_3_C,	4,2	5,0	0,0	3,1
Contig_32594	pfam07971	Glyco_hydro_92,	5,4	3,8	0,0	3,1
Contig_388361	pfam00840	Glyco_hydro_7,	2,3	6,9	0,0	3,1
Contig_172928	pfam03065	Glyco_hydro_57,	6,2	3,0	0,0	3,1
Contig_451360	pfam08532	Glyco_hydro_42M,	2,4	6,8	0,0	3,1
Contig_512953	pfam02449	Glyco_hydro_42,	7,6	1,5	0,0	3,1
Contig_92514	pfam01341	Glyco_hydro_6,	0,0	1,8	7,4	3,1
Contig_313545	pfam02837	Glyco_hydro_2_N,	3,0	6,2	0,0	3,1
Contig_497151	pfam03200	Glyco_hydro_63,	3,7	5,5	0,0	3,1
Contig_316391	pfam07488	Glyco_hydro_67M,	5,3	3,8	0,0	3,1
Contig_305859	pfam01301	Glyco_hydro_35,	3,3	5,9	0,0	3,0
Contig_87522	pfam03200	Glyco_hydro_63,	0,0	3,4	5,7	3,0
Contig_474601	pfam03663	Glyco_hydro_76,	3,8	5,3	0,0	3,0
Contig_21325	pfam00723	Glyco_hydro_15,	3,7	0,0	5,5	3,0
Contig_142107	pfam01055	Glyco_hydro_31,	2,2	2,6	4,3	3,0
Contig_58641	pfam00251	Glyco_hydro_32N,	0,0	2,4	6,7	3,0
Contig_296053	pfam03663	Glyco_hydro_76,	0,0	9,1	0,0	3,0
Contig_311947	pfam10566	Glyco_hydro_97,	3,4	3,9	1,9	3,0
Contig_84160	pfam07971	Glyco_hydro_92,	2,5	0,4	6,2	3,0
Contig_153008	pfam01055	Glyco_hydro_31,	1,0	0,6	7,5	3,0
Contig_248537	pfam03632	Glyco_hydro_65m,	4,4	4,7	0,0	3,0
Contig_69495	pfam00933	Glyco_hydro_3,	2,1	7,0	0,0	3,0
Contig_300455	pfam08307	Glyco_hydro_98C,	4,0	4,4	0,8	3,0
Contig_319493	pfam02836	Glyco_hydro_2_C,	0,0	9,1	0,0	3,0
Contig_462265	pfam00251	Glyco_hydro_32N,	6,7	2,4	0,0	3,0
Contig_584499	pfam07488	Glyco_hydro_67M,	1,6	7,5	0,0	3,0
Contig_141764	pfam03636	Glyco_hydro_65N,	3,5	5,6	0,0	3,0
Contig_261356	pfam02057	Glyco_hydro_59,	6,3	2,7	0,0	3,0
Contig_214455	pfam02324	Glyco_hydro_70,	7,7	1,3	0,0	3,0
Contig_217149	pfam03200	Glyco_hydro_63,	5,1	4,0	0,0	3,0
Contig_292627	pfam02836	Glyco_hydro_2_C,	3,5	5,5	0,0	3,0
Contig_572842	pfam05838	Glyco_hydro_108,	5,7	3,4	0,0	3,0
Contig_455932	pfam02011	Glyco_hydro_48,	4,5	4,5	0,0	3,0
Contig_572064	pfam00704	Glyco_hydro_18,	2,4	6,6	0,0	3,0
Contig_152362	pfam07488	Glyco_hydro_67M,	1,6	1,5	6,0	3,0
Contig_144845	pfam02057	Glyco_hydro_59,	1,4	2,5	5,2	3,0
Contig_544087	pfam07745	Glyco_hydro_53,	8,3	0,7	0,0	3,0
Contig_3514	pfam01183	Glyco_hydro_25,	3,1	3,7	2,3	3,0
Contig_237842	pfam00933	Glyco_hydro_3,	4,9	4,1	0,0	3,0
Contig_357674	pfam00251	Glyco_hydro_32N,	0,0	9,0	0,0	3,0
Contig_371815	pfam01055	Glyco_hydro_31,	4,9	4,2	0,0	3,0
Contig_484524	pfam00759	Glyco_hydro_9,	6,2	2,8	0,0	3,0
Contig_530905	pfam03648	Glyco_hydro_67N,	9,0	0,0	0,0	3,0
Contig_437513	pfam02156	Glyco_hydro_26,	3,1	5,9	0,0	3,0
Contig_33461	pfam00933	Glyco_hydro_3,	3,5	1,1	4,4	3,0
Contig_346482	pfam13199	Glyco_hydro_66,	6,1	2,9	0,0	3,0
Contig_394484	pfam07971	Glyco_hydro_92,	2,6	4,5	1,9	3,0
Contig_385020	pfam00704	Glyco_hydro_18,	5,2	3,8	0,0	3,0
Contig_78113	pfam02446	Glyco_hydro_77,	5,7	1,5	1,8	3,0
Contig_78192	pfam01301	Glyco_hydro_35,	7,2	1,7	0,0	3,0
Contig_279158	pfam01301	Glyco_hydro_35,	0,0	9,0	0,0	3,0
Contig_365119	pfam00933	Glyco_hydro_3,	2,6	6,3	0,0	3,0

Contig_105224	pfam00933	Glyco_hydro_3,	2,7	3,6	2,7	3,0
Contig_440978	pfam02449	Glyco_hydro_42,	0,0	8,9	0,0	3,0
Contig_341953	pfam02324	Glyco_hydro_70,	0,0	8,9	0,0	3,0
Contig_79192	pfam00295	Glyco_hydro_28,	2,6	0,0	6,4	3,0
Contig_234544	pfam03200	Glyco_hydro_63,	0,9	8,0	0,0	3,0
Contig_90827	pfam00728	Glyco_hydro_20,	6,6	1,0	1,4	3,0
Contig_191856	pfam07470	Glyco_hydro_88,	3,3	1,5	4,1	3,0
Contig_415057	pfam03200	Glyco_hydro_63,	0,0	8,9	0,0	3,0
Contig_499081	pfam12899	Glyco_hydro_100,	5,5	3,4	0,0	3,0
Contig_517643	pfam01915	Glyco_hydro_3_C,	7,1	1,8	0,0	3,0
Contig_244296	pfam01055	Glyco_hydro_31,	7,2	1,6	0,0	3,0
Contig_507905	pfam00723	Glyco_hydro_15,	5,1	3,8	0,0	3,0
Contig_166945	pfam01055	Glyco_hydro_31,	0,0	0,7	8,2	3,0
Contig_412758	pfam07477	Glyco_hydro_67C,	7,6	1,3	0,0	3,0
Contig_511810	pfam00723	Glyco_hydro_15,	8,9	0,0	0,0	3,0
Contig_348558	pfam10566	Glyco_hydro_97,	8,8	0,0	0,0	2,9
Contig_76730	pfam13199	Glyco_hydro_66,	0,9	7,9	0,0	2,9
Contig_245145	pfam00933	Glyco_hydro_3,	4,9	3,9	0,0	2,9
Contig_268270	pfam00232	Glyco_hydro_1,	4,1	4,7	0,0	2,9
Contig_325280	pfam00933	Glyco_hydro_3,	3,1	5,7	0,0	2,9
Contig_488270	pfam04616	Glyco_hydro_43,	8,8	0,0	0,0	2,9
Contig_544580	pfam03659	Glyco_hydro_71,	8,8	0,0	0,0	2,9
Contig_555469	pfam00704	Glyco_hydro_18,	8,8	0,0	0,0	2,9
Contig_72138	pfam03200	Glyco_hydro_63,	0,8	2,0	6,1	2,9
Contig_172509	pfam02156	Glyco_hydro_26,	5,1	3,7	0,0	2,9
Contig_159231	pfam01532	Glyco_hydro_47,	0,0	8,8	0,0	2,9
Contig_285928	pfam13199	Glyco_hydro_66,	0,0	8,8	0,0	2,9
Contig_97800	pfam00704	Glyco_hydro_18,	2,0	4,3	2,5	2,9
Contig_318382	pfam02446	Glyco_hydro_77,	7,9	1,0	0,0	2,9
Contig_28429	pfam03198	Glyco_hydro_72,	4,0	2,4	2,5	2,9
Contig_202379	pfam02324	Glyco_hydro_70,	3,9	4,9	0,0	2,9
Contig_197223	pfam13199	Glyco_hydro_66,	3,3	5,5	0,0	2,9
Contig_302977	pfam02057	Glyco_hydro_59,	5,8	3,0	0,0	2,9
Contig_392286	pfam02837	Glyco_hydro_2_N,	0,0	8,8	0,0	2,9
Contig_211544	pfam03200	Glyco_hydro_63,	1,5	7,3	0,0	2,9
Contig_305833	pfam01183	Glyco_hydro_25,	6,7	2,0	0,0	2,9
Contig_311283	pfam03512	Glyco_hydro_52,	3,7	5,0	0,0	2,9
Contig_91812	pfam02055	Glyco_hydro_30,	5,4	0,0	3,4	2,9
Contig_476974	pfam03200	Glyco_hydro_63,	0,9	7,9	0,0	2,9
Contig_488809	pfam01915	Glyco_hydro_3_C,	7,3	1,5	0,0	2,9
Contig_495460	pfam01055	Glyco_hydro_31,	3,1	5,6	0,0	2,9
Contig_221574	pfam03639	Glyco_hydro_81,	2,0	6,8	0,0	2,9
Contig_270833	pfam03639	Glyco_hydro_81,	2,9	5,8	0,0	2,9
Contig_458340	pfam07477	Glyco_hydro_67C,	3,3	2,7	2,7	2,9
Contig_513341	pfam03663	Glyco_hydro_76,	6,2	2,5	0,0	2,9
Contig_310092	pfam00723	Glyco_hydro_15,	3,0	5,7	0,0	2,9
Contig_527516	pfam01055	Glyco_hydro_31,	7,4	1,3	0,0	2,9
Contig_368476	pfam02055	Glyco_hydro_30,	0,0	8,7	0,0	2,9
Contig_415407	pfam03639	Glyco_hydro_81,	7,8	0,9	0,0	2,9
Contig_165322	pfam00331	Glyco_hydro_10,	5,2	3,5	0,0	2,9
Contig_369729	pfam01532	Glyco_hydro_47,	8,2	0,5	0,0	2,9
Contig_290320	pfam12899	Glyco_hydro_100,	4,2	4,5	0,0	2,9
Contig_71537	pfam00232	Glyco_hydro_1,	0,9	5,7	2,1	2,9
Contig_128190	pfam03659	Glyco_hydro_71,	2,9	5,7	0,0	2,9
Contig_250561	pfam01301	Glyco_hydro_35,	4,2	4,5	0,0	2,9
Contig_564958	pfam02057	Glyco_hydro_59,	7,7	1,0	0,0	2,9
Contig_223287	pfam00722	Glyco_hydro_16,	0,0	8,7	0,0	2,9
Contig_466178	pfam00295	Glyco_hydro_28,	3,3	5,3	0,0	2,9
Contig_502729	pfam01074	Glyco_hydro_38,	7,2	1,4	0,0	2,9
Contig_515653	pfam01915	Glyco_hydro_3_C,	6,2	2,4	0,0	2,9
Contig_181297	pfam03632	Glyco_hydro_65m,	5,0	3,6	0,0	2,9
Contig_375306	pfam00295	Glyco_hydro_28,	6,4	2,2	0,0	2,9
Contig_570574	pfam01055	Glyco_hydro_31,	5,4	3,3	0,0	2,9
Contig_279334	pfam00332	Glyco_hydro_17,	0,0	8,6	0,0	2,9
Contig_313635	pfam07745	Glyco_hydro_53,	0,0	8,6	0,0	2,9
Contig_321394	pfam01532	Glyco_hydro_47,	0,0	8,6	0,0	2,9
Contig_281405	pfam00332	Glyco_hydro_17,	2,8	5,8	0,0	2,9
Contig_394986	pfam00457	Glyco_hydro_11,	2,7	5,9	0,0	2,9
Contig_45	pfam01074	Glyco_hydro_38,	2,0	6,6	0,0	2,9
Contig_188179	pfam00251	Glyco_hydro_32N,	6,6	2,0	0,0	2,9
Contig_491052	pfam03662	Glyco_hydro_79n,	6,6	2,0	0,0	2,9

Contig_191154	pfam02055	Glyco_hydro_30,	7,1	1,4	0,0	2,9
Contig_561431	pfam00457	Glyco_hydro_11,	5,9	2,7	0,0	2,9
Contig_194688	pfam00840	Glyco_hydro_7,	5,3	3,2	0,0	2,9
Contig_231271	pfam02057	Glyco_hydro_59,	0,0	8,6	0,0	2,9
Contig_217805	pfam00759	Glyco_hydro_9,	0,9	7,6	0,0	2,9
Contig_77156	pfam02156	Glyco_hydro_26,	4,9	3,7	0,0	2,8
Contig_81049	pfam02446	Glyco_hydro_77,	0,0	5,9	2,7	2,8
Contig_119407	pfam00331	Glyco_hydro_10,	0,0	1,7	6,9	2,8
Contig_214961	pfam07971	Glyco_hydro_92,	1,4	3,5	3,6	2,8
Contig_394404	pfam00704	Glyco_hydro_18,	2,9	5,6	0,0	2,8
Contig_431469	pfam08532	Glyco_hydro_42M,	2,2	6,3	0,0	2,8
Contig_343233	pfam07748	Glyco_hydro_38C,	0,7	7,8	0,0	2,8
Contig_400819	pfam03639	Glyco_hydro_81,	0,0	8,5	0,0	2,8
Contig_518582	pfam03639	Glyco_hydro_81,	6,9	1,7	0,0	2,8
Contig_311568	pfam07748	Glyco_hydro_38C,	3,9	4,7	0,0	2,8
Contig_71906	pfam02011	Glyco_hydro_48,	4,5	4,0	0,0	2,8
Contig_239330	pfam00232	Glyco_hydro_1,	2,7	5,9	0,0	2,8
Contig_295521	pfam12891	Glyco_hydro_44,	3,5	4,9	0,0	2,8
Contig_381373	pfam00703	Glyco_hydro_2,	0,0	8,5	0,0	2,8
Contig_538418	pfam03718	Glyco_hydro_49,	8,5	0,0	0,0	2,8
Contig_485116	pfam00759	Glyco_hydro_9,	0,0	8,5	0,0	2,8
Contig_99761	pfam07470	Glyco_hydro_88,	4,0	4,5	0,0	2,8
Contig_134863	pfam01055	Glyco_hydro_31,	0,0	2,8	5,7	2,8
Contig_511191	pfam13199	Glyco_hydro_66,	6,2	2,2	0,0	2,8
Contig_135283	pfam00728	Glyco_hydro_20,	7,0	1,4	0,0	2,8
Contig_190510	pfam11790	Glyco_hydro_cc,	0,0	8,4	0,0	2,8
Contig_306177	pfam07477	Glyco_hydro_67C,	2,6	5,9	0,0	2,8
Contig_494086	pfam00933	Glyco_hydro_3,	3,5	4,9	0,0	2,8
Contig_132854	pfam01341	Glyco_hydro_6,	7,6	0,8	0,0	2,8
Contig_444003	pfam03200	Glyco_hydro_63,	7,2	1,2	0,0	2,8
Contig_209154	pfam02836	Glyco_hydro_2_C,	7,3	1,1	0,0	2,8
Contig_211701	pfam01183	Glyco_hydro_25,	2,0	6,4	0,0	2,8
Contig_311602	pfam01301	Glyco_hydro_35,	3,5	4,9	0,0	2,8
Contig_473723	pfam07745	Glyco_hydro_53,	8,4	0,0	0,0	2,8
Contig_224557	pfam00704	Glyco_hydro_18,	6,0	2,3	0,0	2,8
Contig_314376	pfam00332	Glyco_hydro_17,	6,0	2,4	0,0	2,8
Contig_333772	pfam12905	Glyco_hydro_101,	4,6	3,7	0,0	2,8
Contig_47622	pfam07477	Glyco_hydro_67C,	4,5	3,8	0,0	2,8
Contig_478180	pfam00232	Glyco_hydro_1,	0,0	8,4	0,0	2,8
Contig_571000	pfam07971	Glyco_hydro_92,	4,2	0,3	3,9	2,8
Contig_226829	pfam12899	Glyco_hydro_100,	1,3	7,0	0,0	2,8
Contig_410111	pfam02055	Glyco_hydro_30,	7,3	1,0	0,0	2,8
Contig_452160	pfam01074	Glyco_hydro_38,	4,9	3,4	0,0	2,8
Contig_98566	pfam03200	Glyco_hydro_63,	0,8	4,4	3,1	2,8
Contig_573390	pfam01532	Glyco_hydro_47,	8,3	0,0	0,0	2,8
Contig_216592	pfam03662	Glyco_hydro_79n,	2,0	6,3	0,0	2,8
Contig_241905	pfam02838	Glyco_hydro_20b,	4,5	3,8	0,0	2,8
Contig_571018	pfam01341	Glyco_hydro_6,	0,0	5,9	2,4	2,8
Contig_539410	pfam01341	Glyco_hydro_6,	3,8	0,7	3,8	2,8
Contig_454087	pfam04616	Glyco_hydro_43,	6,1	2,2	0,0	2,8
Contig_45942	pfam01055	Glyco_hydro_31,	3,1	1,4	3,8	2,8
Contig_398090	pfam01055	Glyco_hydro_31,	4,4	3,9	0,0	2,8
Contig_220009	pfam00331	Glyco_hydro_10,	3,3	5,0	0,0	2,8
Contig_295779	pfam08306	Glyco_hydro_98M,	3,8	4,5	0,0	2,8
Contig_122679	pfam00232	Glyco_hydro_1,	0,0	6,0	2,3	2,8
Contig_170408	pfam03632	Glyco_hydro_65m,	2,5	5,7	0,0	2,8
Contig_211115	pfam00722	Glyco_hydro_16,	2,4	5,8	0,0	2,8
Contig_280414	pfam01670	Glyco_hydro_12,	7,8	0,4	0,0	2,7
Contig_545578	pfam03065	Glyco_hydro_57,	8,2	0,0	0,0	2,7
Contig_181643	pfam01183	Glyco_hydro_25,	3,9	4,3	0,0	2,7
Contig_366119	pfam00182	Glyco_hydro_19,	0,0	8,2	0,0	2,7
Contig_570214	pfam00759	Glyco_hydro_9,	8,2	0,0	0,0	2,7
Contig_571872	pfam07477	Glyco_hydro_67C,	7,2	1,1	0,0	2,7
Contig_475374	pfam00704	Glyco_hydro_18,	2,6	5,6	0,0	2,7
Contig_397886	pfam01532	Glyco_hydro_47,	4,8	3,5	0,0	2,7
Contig_57181	pfam03065	Glyco_hydro_57,	5,6	0,9	1,7	2,7
Contig_322311	pfam01183	Glyco_hydro_25,	3,1	5,1	0,0	2,7
Contig_110362	pfam03632	Glyco_hydro_65m,	2,3	3,1	2,8	2,7
Contig_9936	pfam00723	Glyco_hydro_15,	2,9	5,3	0,0	2,7
Contig_260322	pfam03659	Glyco_hydro_71,	0,0	8,2	0,0	2,7
Contig_511524	pfam03664	Glyco_hydro_62,	6,3	1,9	0,0	2,7

Contig_146529	pfam12899	Glyco_hydro_100,	2,3	0,0	5,8	2,7
Contig_224210	pfam03200	Glyco_hydro_63,	2,0	6,1	0,0	2,7
Contig_364687	pfam02446	Glyco_hydro_77,	0,6	6,3	1,4	2,7
Contig_419906	pfam02055	Glyco_hydro_30,	8,2	0,0	0,0	2,7
Contig_52837	pfam12899	Glyco_hydro_100,	0,0	4,7	3,5	2,7
Contig_92861	pfam03639	Glyco_hydro_81,	3,4	1,9	2,8	2,7
Contig_344400	pfam00332	Glyco_hydro_17,	3,0	5,1	0,0	2,7
Contig_494572	pfam01532	Glyco_hydro_47,	3,7	4,5	0,0	2,7
Contig_25478	pfam01341	Glyco_hydro_6,	0,0	2,5	5,7	2,7
Contig_179723	pfam00933	Glyco_hydro_3,	6,0	2,1	0,0	2,7
Contig_549352	pfam02057	Glyco_hydro_59,	8,1	0,0	0,0	2,7
Contig_10778	pfam00704	Glyco_hydro_18,	7,8	0,4	0,0	2,7
Contig_209510	pfam02836	Glyco_hydro_2_C,	0,0	0,9	7,2	2,7
Contig_261773	pfam02156	Glyco_hydro_26,	1,2	6,9	0,0	2,7
Contig_313797	pfam10566	Glyco_hydro_97,	6,0	2,2	0,0	2,7
Contig_341902	pfam10566	Glyco_hydro_97,	0,0	8,1	0,0	2,7
Contig_407472	pfam03200	Glyco_hydro_63,	0,0	2,6	5,5	2,7
Contig_305895	pfam02837	Glyco_hydro_2_N,	6,8	1,3	0,0	2,7
Contig_54579	pfam01915	Glyco_hydro_3_C,	1,4	3,3	3,4	2,7
Contig_527380	pfam10566	Glyco_hydro_97,	8,1	0,0	0,0	2,7
Contig_196102	pfam00704	Glyco_hydro_18,	6,0	2,1	0,0	2,7
Contig_249540	pfam01229	Glyco_hydro_39,	6,2	1,9	0,0	2,7
Contig_276155	pfam00704	Glyco_hydro_18,	8,1	0,0	0,0	2,7
Contig_361322	pfam10566	Glyco_hydro_97,	0,0	8,1	0,0	2,7
Contig_214132	pfam00331	Glyco_hydro_10,	6,5	1,6	0,0	2,7
Contig_203444	pfam08532	Glyco_hydro_42M,	1,3	6,8	0,0	2,7
Contig_324763	pfam00728	Glyco_hydro_20,	0,0	8,0	0,0	2,7
Contig_135308	pfam02446	Glyco_hydro_77,	4,3	3,7	0,0	2,7
Contig_471288	pfam03664	Glyco_hydro_62,	5,7	2,3	0,0	2,7
Contig_193386	pfam00251	Glyco_hydro_32N,	1,3	6,7	0,0	2,7
Contig_312522	pfam03639	Glyco_hydro_81,	4,2	3,8	0,0	2,7
Contig_460811	pfam07335	Glyco_hydro_75,	5,0	3,0	0,0	2,7
Contig_461955	pfam00182	Glyco_hydro_19,	8,0	0,0	0,0	2,7
Contig_29664	pfam03198	Glyco_hydro_72,	4,2	3,8	0,0	2,7
Contig_220625	pfam00232	Glyco_hydro_1,	4,0	4,0	0,0	2,7
Contig_261420	pfam03632	Glyco_hydro_65m,	5,3	2,7	0,0	2,7
Contig_458425	pfam07971	Glyco_hydro_92,	5,0	3,0	0,0	2,7
Contig_261639	pfam02015	Glyco_hydro_45,	6,2	1,9	0,0	2,7
Contig_316000	pfam01055	Glyco_hydro_31,	3,4	4,6	0,0	2,7
Contig_407183	pfam07470	Glyco_hydro_88,	3,4	4,6	0,0	2,7
Contig_324360	pfam03198	Glyco_hydro_72,	2,6	5,4	0,0	2,7
Contig_358162	pfam01670	Glyco_hydro_12,	4,6	3,4	0,0	2,7
Contig_65203	pfam02324	Glyco_hydro_70,	5,4	2,6	0,0	2,7
Contig_71116	pfam07470	Glyco_hydro_88,	5,7	2,3	0,0	2,7
Contig_157091	pfam12899	Glyco_hydro_100,	4,4	3,6	0,0	2,7
Contig_248922	pfam07477	Glyco_hydro_67C,	6,0	1,9	0,0	2,7
Contig_356410	pfam00232	Glyco_hydro_1,	3,2	4,8	0,0	2,7
Contig_34960	pfam01532	Glyco_hydro_47,	0,0	4,6	3,4	2,7
Contig_279965	pfam07971	Glyco_hydro_92,	1,1	6,9	0,0	2,7
Contig_163501	pfam01915	Glyco_hydro_3_C,	5,5	2,5	0,0	2,7
Contig_248230	pfam03639	Glyco_hydro_81,	6,3	1,7	0,0	2,7
Contig_367049	pfam03718	Glyco_hydro_49,	0,0	8,0	0,0	2,7
Contig_75421	pfam01915	Glyco_hydro_3_C,	0,0	2,6	5,4	2,7
Contig_180478	pfam00251	Glyco_hydro_32N,	1,7	6,2	0,0	2,7
Contig_113249	pfam01532	Glyco_hydro_47,	7,9	0,0	0,0	2,6
Contig_182473	pfam01301	Glyco_hydro_35,	3,7	4,2	0,0	2,6
Contig_381177	pfam00232	Glyco_hydro_1,	3,8	3,2	0,9	2,6
Contig_294376	pfam00182	Glyco_hydro_19,	5,4	2,6	0,0	2,6
Contig_301498	pfam00722	Glyco_hydro_16,	2,3	5,6	0,0	2,6
Contig_405371	pfam11790	Glyco_hydro_cc,	6,3	1,6	0,0	2,6
Contig_540335	pfam12899	Glyco_hydro_100,	4,5	3,4	0,0	2,6
Contig_5965	pfam03718	Glyco_hydro_49,	4,3	1,5	2,1	2,6
Contig_100663	pfam03662	Glyco_hydro_79n,	1,3	0,0	6,6	2,6
Contig_307122	pfam10566	Glyco_hydro_97,	4,9	3,0	0,0	2,6
Contig_473111	pfam11790	Glyco_hydro_cc,	5,2	2,7	0,0	2,6
Contig_82748	pfam10566	Glyco_hydro_97,	2,1	1,9	3,9	2,6
Contig_319700	pfam01055	Glyco_hydro_31,	5,4	2,5	0,0	2,6
Contig_226708	pfam03636	Glyco_hydro_65N,	4,8	3,1	0,0	2,6
Contig_210954	pfam02449	Glyco_hydro_42,	3,8	4,1	0,0	2,6
Contig_440709	pfam04616	Glyco_hydro_43,	3,1	4,7	0,0	2,6
Contig_229767	pfam04616	Glyco_hydro_43,	0,0	7,9	0,0	2,6

Contig_297326	pfam10566	Glyco_hydro_97,	0,0	7,9	0,0	2,6
Contig_398275	pfam07971	Glyco_hydro_92,	2,0	5,9	0,0	2,6
Contig_576451	pfam00704	Glyco_hydro_18,	7,2	0,7	0,0	2,6
Contig_128970	pfam00759	Glyco_hydro_9,	1,3	0,0	6,6	2,6
Contig_133209	pfam10566	Glyco_hydro_97,	0,6	2,7	4,5	2,6
Contig_239345	pfam01270	Glyco_hydro_8,	0,9	7,0	0,0	2,6
Contig_410983	pfam03065	Glyco_hydro_57,	6,7	1,2	0,0	2,6
Contig_483877	pfam12899	Glyco_hydro_100,	7,9	0,0	0,0	2,6
Contig_82730	pfam00232	Glyco_hydro_1,	3,8	0,0	4,0	2,6
Contig_198837	pfam00933	Glyco_hydro_3,	0,0	7,8	0,0	2,6
Contig_466965	pfam04616	Glyco_hydro_43,	1,7	6,1	0,0	2,6
Contig_173555	pfam02837	Glyco_hydro_2_N,	3,0	4,8	0,0	2,6
Contig_533984	pfam00251	Glyco_hydro_32N,	7,8	0,0	0,0	2,6
Contig_83867	pfam02324	Glyco_hydro_70,	0,0	4,9	2,9	2,6
Contig_105176	pfam00251	Glyco_hydro_32N,	6,2	1,6	0,0	2,6
Contig_476504	pfam03200	Glyco_hydro_63,	7,8	0,0	0,0	2,6
Contig_515252	pfam01630	Glyco_hydro_56,	7,1	0,7	0,0	2,6
Contig_573474	pfam00723	Glyco_hydro_15,	6,4	1,4	0,0	2,6
Contig_442947	pfam01532	Glyco_hydro_47,	7,3	0,4	0,0	2,6
Contig_319616	pfam08307	Glyco_hydro_98C,	0,0	7,8	0,0	2,6
Contig_569693	pfam02446	Glyco_hydro_77,	7,8	0,0	0,0	2,6
Contig_80626	pfam11975	Glyco_hydro_4C,	0,0	2,2	5,6	2,6
Contig_358646	pfam03512	Glyco_hydro_52,	3,5	4,2	0,0	2,6
Contig_581345	pfam00933	Glyco_hydro_3,	5,3	2,4	0,0	2,6
Contig_140235	pfam04616	Glyco_hydro_43,	4,8	2,9	0,0	2,6
Contig_328306	pfam00722	Glyco_hydro_16,	2,2	5,6	0,0	2,6
Contig_366258	pfam02156	Glyco_hydro_26,	3,5	4,2	0,0	2,6
Contig_471396	pfam02011	Glyco_hydro_48,	7,7	0,0	0,0	2,6
Contig_492158	pfam03200	Glyco_hydro_63,	4,8	2,9	0,0	2,6
Contig_539315	pfam01183	Glyco_hydro_25,	7,7	0,0	0,0	2,6
Contig_78330	pfam01532	Glyco_hydro_47,	3,5	0,8	3,4	2,6
Contig_411604	pfam03200	Glyco_hydro_63,	6,9	0,8	0,0	2,6
Contig_565121	pfam01301	Glyco_hydro_35,	7,7	0,0	0,0	2,6
Contig_300801	pfam03663	Glyco_hydro_76,	7,1	0,7	0,0	2,6
Contig_356054	pfam01183	Glyco_hydro_25,	2,5	5,2	0,0	2,6
Contig_580762	pfam03512	Glyco_hydro_52,	7,7	0,0	0,0	2,6
Contig_407020	pfam00704	Glyco_hydro_18,	6,7	1,0	0,0	2,6
Contig_407757	pfam00332	Glyco_hydro_17,	6,2	1,5	0,0	2,6
Contig_413061	pfam12899	Glyco_hydro_100,	3,5	4,2	0,0	2,6
Contig_350366	pfam02055	Glyco_hydro_30,	0,0	7,7	0,0	2,6
Contig_544642	pfam00722	Glyco_hydro_16,	7,7	0,0	0,0	2,6
Contig_299493	pfam03664	Glyco_hydro_62,	3,5	4,2	0,0	2,6
Contig_353728	pfam07470	Glyco_hydro_88,	2,3	3,9	1,5	2,6
Contig_355919	pfam07745	Glyco_hydro_53,	0,0	7,7	0,0	2,6
Contig_559170	pfam01301	Glyco_hydro_35,	7,7	0,0	0,0	2,6
Contig_60701	pfam01373	Glyco_hydro_14,	3,5	4,2	0,0	2,6
Contig_518138	pfam00232	Glyco_hydro_1,	4,8	2,9	0,0	2,6
Contig_119871	pfam00704	Glyco_hydro_18,	4,5	0,0	3,2	2,5
Contig_299433	pfam00703	Glyco_hydro_2,	1,2	6,5	0,0	2,5
Contig_268327	pfam00232	Glyco_hydro_1,	7,6	0,0	0,0	2,5
Contig_359281	pfam00704	Glyco_hydro_18,	0,0	7,6	0,0	2,5
Contig_152479	pfam01055	Glyco_hydro_31,	0,8	0,5	6,3	2,5
Contig_159753	pfam00332	Glyco_hydro_17,	0,0	7,6	0,0	2,5
Contig_295559	pfam00704	Glyco_hydro_18,	6,1	1,6	0,0	2,5
Contig_496076	pfam00704	Glyco_hydro_18,	6,8	0,8	0,0	2,5
Contig_562053	pfam01915	Glyco_hydro_3_C,	3,8	3,8	0,0	2,5
Contig_157968	pfam07971	Glyco_hydro_92,	0,0	7,6	0,0	2,5
Contig_542555	pfam12899	Glyco_hydro_100,	7,6	0,0	0,0	2,5
Contig_83461	pfam01301	Glyco_hydro_35,	0,0	4,8	2,8	2,5
Contig_552097	pfam02055	Glyco_hydro_30,	7,6	0,0	0,0	2,5
Contig_139401	pfam03664	Glyco_hydro_62,	1,2	0,4	6,0	2,5
Contig_56566	pfam13647	Glyco_hydro_80,	7,6	0,0	0,0	2,5
Contig_116920	pfam00182	Glyco_hydro_19,	2,5	5,1	0,0	2,5
Contig_579374	pfam03198	Glyco_hydro_72,	7,6	0,0	0,0	2,5
Contig_231674	pfam01532	Glyco_hydro_47,	2,7	4,9	0,0	2,5
Contig_415491	pfam00722	Glyco_hydro_16,	2,5	2,7	2,4	2,5
Contig_108818	pfam02055	Glyco_hydro_30,	1,4	2,6	3,5	2,5
Contig_438535	pfam07971	Glyco_hydro_92,	0,0	7,5	0,0	2,5
Contig_332966	pfam02011	Glyco_hydro_48,	1,6	5,9	0,0	2,5
Contig_537880	pfam03512	Glyco_hydro_52,	7,5	0,0	0,0	2,5
Contig_47883	pfam02449	Glyco_hydro_42,	6,5	1,1	0,0	2,5

Contig_87673	pfam03639	Glyco_hydro_81,	0,0	4,1	3,4	2,5
Contig_191958	pfam00251	Glyco_hydro_32N,	0,0	7,5	0,0	2,5
Contig_580488	pfam13199	Glyco_hydro_66,	6,7	0,8	0,0	2,5
Contig_43667	pfam03639	Glyco_hydro_81,	0,8	0,5	6,2	2,5
Contig_153615	pfam07748	Glyco_hydro_38C,	7,5	0,0	0,0	2,5
Contig_243282	pfam01630	Glyco_hydro_56,	0,0	7,5	0,0	2,5
Contig_278205	pfam03636	Glyco_hydro_65N,	4,3	3,2	0,0	2,5
Contig_330247	pfam00704	Glyco_hydro_18,	3,6	3,9	0,0	2,5
Contig_440056	pfam13199	Glyco_hydro_66,	3,1	4,4	0,0	2,5
Contig_512213	pfam00232	Glyco_hydro_1,	7,5	0,0	0,0	2,5
Contig_556649	pfam03659	Glyco_hydro_71,	7,5	0,0	0,0	2,5
Contig_243745	pfam00295	Glyco_hydro_28,	0,6	6,9	0,0	2,5
Contig_255812	pfam00728	Glyco_hydro_20,	0,0	7,5	0,0	2,5
Contig_304445	pfam07748	Glyco_hydro_38C,	1,3	6,2	0,0	2,5
Contig_391253	pfam02837	Glyco_hydro_2_N,	6,5	1,0	0,0	2,5
Contig_399708	pfam00331	Glyco_hydro_10,	3,0	4,5	0,0	2,5
Contig_430461	pfam00331	Glyco_hydro_10,	5,1	2,3	0,0	2,5
Contig_483307	pfam02324	Glyco_hydro_70,	5,1	2,3	0,0	2,5
Contig_317731	pfam03065	Glyco_hydro_57,	6,7	0,8	0,0	2,5
Contig_439697	pfam07748	Glyco_hydro_38C,	5,5	2,0	0,0	2,5
Contig_579659	pfam03662	Glyco_hydro_79n,	5,5	2,0	0,0	2,5
Contig_492729	pfam00933	Glyco_hydro_3,	5,1	2,3	0,0	2,5
Contig_271013	pfam01532	Glyco_hydro_47,	0,0	7,5	0,0	2,5
Contig_407549	pfam00759	Glyco_hydro_9,	0,0	7,4	0,0	2,5
Contig_566847	pfam01055	Glyco_hydro_31,	7,4	0,0	0,0	2,5
Contig_514672	pfam01532	Glyco_hydro_47,	1,6	5,8	0,0	2,5
Contig_379799	pfam00251	Glyco_hydro_32N,	6,0	1,4	0,0	2,5
Contig_416957	pfam00251	Glyco_hydro_32N,	5,0	2,4	0,0	2,5
Contig_494420	pfam04616	Glyco_hydro_43,	3,3	0,0	4,1	2,5
Contig_541133	pfam03659	Glyco_hydro_71,	5,3	2,1	0,0	2,5
Contig_376118	pfam03639	Glyco_hydro_81,	0,8	6,6	0,0	2,5
Contig_396409	pfam04616	Glyco_hydro_43,	2,6	4,8	0,0	2,5
Contig_460407	pfam03648	Glyco_hydro_67N,	0,0	7,4	0,0	2,5
Contig_412645	pfam03200	Glyco_hydro_63,	4,1	3,3	0,0	2,5
Contig_389116	pfam11790	Glyco_hydro_cc,	5,8	1,6	0,0	2,5
Contig_77631	pfam01532	Glyco_hydro_47,	1,3	3,0	3,1	2,5
Contig_172015	pfam01229	Glyco_hydro_39,	4,8	2,6	0,0	2,5
Contig_180860	pfam03663	Glyco_hydro_76,	3,1	4,3	0,0	2,5
Contig_407399	pfam03632	Glyco_hydro_65m,	4,0	3,3	0,0	2,5
Contig_447978	pfam00704	Glyco_hydro_18,	5,7	1,7	0,0	2,5
Contig_327489	pfam02156	Glyco_hydro_26,	3,5	3,9	0,0	2,5
Contig_255564	pfam00759	Glyco_hydro_9,	6,8	0,6	0,0	2,5
Contig_425132	pfam01055	Glyco_hydro_31,	0,0	7,4	0,0	2,5
Contig_254449	pfam03718	Glyco_hydro_49,	0,7	6,6	0,0	2,4
Contig_503242	pfam03718	Glyco_hydro_49,	4,3	3,0	0,0	2,4
Contig_207138	pfam03200	Glyco_hydro_63,	0,0	7,3	0,0	2,4
Contig_383888	pfam03200	Glyco_hydro_63,	0,0	7,3	0,0	2,4
Contig_393149	pfam07971	Glyco_hydro_92,	7,3	0,0	0,0	2,4
Contig_549240	pfam03644	Glyco_hydro_85,	7,3	0,0	0,0	2,4
Contig_320032	pfam02449	Glyco_hydro_42,	5,1	2,2	0,0	2,4
Contig_311734	pfam02446	Glyco_hydro_77,	1,3	6,0	0,0	2,4
Contig_457235	pfam07470	Glyco_hydro_88,	5,8	1,5	0,0	2,4
Contig_182238	pfam03636	Glyco_hydro_65N,	7,3	0,0	0,0	2,4
Contig_482992	pfam02446	Glyco_hydro_77,	0,0	5,2	2,1	2,4
Contig_168774	pfam01532	Glyco_hydro_47,	1,6	5,7	0,0	2,4
Contig_347829	pfam07470	Glyco_hydro_88,	4,0	3,2	0,0	2,4
Contig_363012	pfam04616	Glyco_hydro_43,	2,6	4,7	0,0	2,4
Contig_245282	pfam01532	Glyco_hydro_47,	2,1	5,2	0,0	2,4
Contig_328680	pfam01074	Glyco_hydro_38,	2,3	4,9	0,0	2,4
Contig_102436	pfam00704	Glyco_hydro_18,	6,5	0,8	0,0	2,4
Contig_240573	pfam00704	Glyco_hydro_18,	0,0	7,3	0,0	2,4
Contig_305010	pfam02837	Glyco_hydro_2_N,	7,3	0,0	0,0	2,4
Contig_478874	pfam02055	Glyco_hydro_30,	7,3	0,0	0,0	2,4
Contig_77555	pfam07488	Glyco_hydro_67M,	0,0	0,0	7,2	2,4
Contig_253082	pfam03718	Glyco_hydro_49,	4,5	2,7	0,0	2,4
Contig_19602	pfam11790	Glyco_hydro_cc,	1,2	1,8	4,3	2,4
Contig_89546	pfam03200	Glyco_hydro_63,	0,0	4,0	3,3	2,4
Contig_169256	pfam00295	Glyco_hydro_28,	6,3	1,0	0,0	2,4
Contig_503779	pfam07971	Glyco_hydro_92,	3,2	0,0	4,0	2,4
Contig_88949	pfam01532	Glyco_hydro_47,	4,8	0,3	2,2	2,4
Contig_51028	pfam01532	Glyco_hydro_47,	4,0	3,2	0,0	2,4

Contig_483654	pfam02446	Glyco_hydro_77,	2,6	4,7	0,0	2,4
Contig_129805	pfam01229	Glyco_hydro_39,	3,5	3,7	0,0	2,4
Contig_261382	pfam02449	Glyco_hydro_42,	1,1	6,1	0,0	2,4
Contig_306248	pfam03636	Glyco_hydro_65N,	0,0	7,2	0,0	2,4
Contig_565179	pfam02836	Glyco_hydro_2_C,	7,2	0,0	0,0	2,4
Contig_100570	pfam01229	Glyco_hydro_39,	0,0	7,2	0,0	2,4
Contig_412806	pfam12899	Glyco_hydro_100,	3,3	3,9	0,0	2,4
Contig_295660	pfam03632	Glyco_hydro_65m,	3,0	4,2	0,0	2,4
Contig_389722	pfam00457	Glyco_hydro_11,	0,0	7,2	0,0	2,4
Contig_231882	pfam02057	Glyco_hydro_59,	3,8	3,4	0,0	2,4
Contig_289182	pfam04616	Glyco_hydro_43,	1,9	5,3	0,0	2,4
Contig_244682	pfam02836	Glyco_hydro_2_C,	3,1	4,1	0,0	2,4
Contig_323496	pfam01055	Glyco_hydro_31,	5,3	1,9	0,0	2,4
Contig_445624	pfam02156	Glyco_hydro_26,	6,3	0,9	0,0	2,4
Contig_171164	pfam07971	Glyco_hydro_92,	6,1	1,1	0,0	2,4
Contig_220051	pfam03512	Glyco_hydro_52,	2,7	4,5	0,0	2,4
Contig_495983	pfam02449	Glyco_hydro_42,	6,8	0,4	0,0	2,4
Contig_300489	pfam02446	Glyco_hydro_77,	2,6	4,6	0,0	2,4
Contig_457191	pfam03662	Glyco_hydro_79n,	4,5	2,7	0,0	2,4
Contig_354002	pfam00840	Glyco_hydro_7,	3,5	3,7	0,0	2,4
Contig_491829	pfam03659	Glyco_hydro_71,	4,9	2,2	0,0	2,4
Contig_565263	pfam01532	Glyco_hydro_47,	3,9	0,0	3,3	2,4
Contig_238513	pfam00759	Glyco_hydro_9,	5,4	1,7	0,0	2,4
Contig_2877	pfam00232	Glyco_hydro_1,	0,0	0,0	7,1	2,4
Contig_545560	pfam00704	Glyco_hydro_18,	5,7	1,4	0,0	2,4
Contig_557358	pfam00232	Glyco_hydro_1,	7,1	0,0	0,0	2,4
Contig_340801	pfam03443	Glyco_hydro_61,	6,6	0,5	0,0	2,4
Contig_99817	pfam00331	Glyco_hydro_10,	7,1	0,0	0,0	2,4
Contig_536761	pfam00759	Glyco_hydro_9,	6,7	0,4	0,0	2,4
Contig_392931	pfam00722	Glyco_hydro_16,	3,8	0,5	2,8	2,4
Contig_248570	pfam02838	Glyco_hydro_20b,	0,0	7,1	0,0	2,4
Contig_434201	pfam00728	Glyco_hydro_20,	4,4	2,7	0,0	2,4
Contig_1543	pfam01055	Glyco_hydro_31,	7,1	0,0	0,0	2,4
Contig_552044	pfam01055	Glyco_hydro_31,	7,1	0,0	0,0	2,4
Contig_63730	pfam03065	Glyco_hydro_57,	4,8	2,3	0,0	2,4
Contig_484823	pfam02057	Glyco_hydro_59,	6,3	0,8	0,0	2,4
Contig_262073	pfam01915	Glyco_hydro_3_C,	3,0	4,1	0,0	2,4
Contig_177826	pfam00933	Glyco_hydro_3,	2,9	1,4	2,8	2,3
Contig_284935	pfam00232	Glyco_hydro_1,	2,7	4,3	0,0	2,3
Contig_300757	pfam00704	Glyco_hydro_18,	0,0	7,0	0,0	2,3
Contig_487167	pfam04616	Glyco_hydro_43,	0,0	7,0	0,0	2,3
Contig_97783	pfam01183	Glyco_hydro_25,	1,1	4,5	1,4	2,3
Contig_161916	pfam03718	Glyco_hydro_49,	0,0	7,0	0,0	2,3
Contig_291331	pfam03200	Glyco_hydro_63,	5,6	1,4	0,0	2,3
Contig_434849	pfam03633	Glyco_hydro_65C,	2,5	4,5	0,0	2,3
Contig_294638	pfam13199	Glyco_hydro_66,	6,0	1,0	0,0	2,3
Contig_452499	pfam04616	Glyco_hydro_43,	4,8	2,2	0,0	2,3
Contig_84790	pfam03663	Glyco_hydro_76,	4,3	0,5	2,2	2,3
Contig_221375	pfam00332	Glyco_hydro_17,	0,0	7,0	0,0	2,3
Contig_308221	pfam03644	Glyco_hydro_85,	4,4	2,6	0,0	2,3
Contig_327764	pfam00251	Glyco_hydro_32N,	6,1	0,9	0,0	2,3
Contig_557608	pfam00331	Glyco_hydro_10,	6,0	1,0	0,0	2,3
Contig_218601	pfam00295	Glyco_hydro_28,	0,0	7,0	0,0	2,3
Contig_365322	pfam12899	Glyco_hydro_100,	1,2	5,8	0,0	2,3
Contig_328691	pfam00759	Glyco_hydro_9,	3,5	3,5	0,0	2,3
Contig_405675	pfam12899	Glyco_hydro_100,	3,5	3,5	0,0	2,3
Contig_257292	pfam00722	Glyco_hydro_16,	3,0	4,0	0,0	2,3
Contig_341885	pfam03198	Glyco_hydro_72,	0,0	7,0	0,0	2,3
Contig_267258	pfam01270	Glyco_hydro_8,	5,6	1,4	0,0	2,3
Contig_159008	pfam02057	Glyco_hydro_59,	5,2	1,8	0,0	2,3
Contig_506384	pfam03512	Glyco_hydro_52,	5,5	1,4	0,0	2,3
Contig_73816	pfam01055	Glyco_hydro_31,	3,8	0,0	3,2	2,3
Contig_490998	pfam10566	Glyco_hydro_97,	7,0	0,0	0,0	2,3
Contig_252267	pfam01055	Glyco_hydro_31,	1,7	5,2	0,0	2,3
Contig_445115	pfam07971	Glyco_hydro_92,	0,0	0,0	6,9	2,3
Contig_511414	pfam02011	Glyco_hydro_48,	5,3	1,6	0,0	2,3
Contig_511255	pfam10566	Glyco_hydro_97,	3,7	3,3	0,0	2,3
Contig_174019	pfam03632	Glyco_hydro_65m,	2,4	1,5	3,0	2,3
Contig_393487	pfam00232	Glyco_hydro_1,	2,2	4,7	0,0	2,3
Contig_459517	pfam01055	Glyco_hydro_31,	4,7	2,3	0,0	2,3
Contig_488817	pfam03663	Glyco_hydro_76,	3,1	3,8	0,0	2,3

Contig_560530	pfam10566	Glyco_hydro_97,	6,9	0,0	0,0	2,3
Contig_513416	pfam01055	Glyco_hydro_31,	6,6	0,4	0,0	2,3
Contig_401338	pfam01341	Glyco_hydro_6,	2,5	4,5	0,0	2,3
Contig_443562	pfam03644	Glyco_hydro_85,	3,5	3,5	0,0	2,3
Contig_66609	pfam03639	Glyco_hydro_81,	0,0	3,8	3,1	2,3
Contig_494258	pfam03639	Glyco_hydro_81,	6,0	0,9	0,0	2,3
Contig_430478	pfam03200	Glyco_hydro_63,	0,6	6,3	0,0	2,3
Contig_202614	pfam01229	Glyco_hydro_39,	2,6	3,1	1,3	2,3
Contig_210223	pfam03639	Glyco_hydro_81,	4,3	2,6	0,0	2,3
Contig_215522	pfam00722	Glyco_hydro_16,	0,0	6,9	0,0	2,3
Contig_573659	pfam02055	Glyco_hydro_30,	6,9	0,0	0,0	2,3
Contig_568867	pfam01183	Glyco_hydro_25,	3,4	3,4	0,0	2,3
Contig_121379	pfam07335	Glyco_hydro_75,	6,0	0,9	0,0	2,3
Contig_541459	pfam02435	Glyco_hydro_68,	6,0	0,9	0,0	2,3
Contig_82084	pfam11790	Glyco_hydro_cc,	0,0	2,5	4,4	2,3
Contig_512511	pfam00332	Glyco_hydro_17,	4,6	2,2	0,0	2,3
Contig_43437	pfam01183	Glyco_hydro_25,	2,5	1,3	3,1	2,3
Contig_291162	pfam00704	Glyco_hydro_18,	1,3	5,5	0,0	2,3
Contig_315596	pfam02836	Glyco_hydro_2_C,	2,8	4,0	0,0	2,3
Contig_406255	pfam03718	Glyco_hydro_49,	2,4	4,4	0,0	2,3
Contig_473616	pfam03663	Glyco_hydro_76,	6,3	0,5	0,0	2,3
Contig_80582	pfam03639	Glyco_hydro_81,	4,7	1,3	0,9	2,3
Contig_374806	pfam07971	Glyco_hydro_92,	5,0	1,8	0,0	2,3
Contig_415926	pfam13647	Glyco_hydro_80,	0,0	6,8	0,0	2,3
Contig_458829	pfam00332	Glyco_hydro_17,	5,8	1,0	0,0	2,3
Contig_13595	pfam10566	Glyco_hydro_97,	2,4	2,9	1,5	2,3
Contig_154450	pfam03662	Glyco_hydro_79n,	2,7	4,1	0,0	2,3
Contig_339920	pfam01229	Glyco_hydro_39,	1,7	5,1	0,0	2,3
Contig_406599	pfam02324	Glyco_hydro_70,	1,5	5,3	0,0	2,3
Contig_493773	pfam00331	Glyco_hydro_10,	6,8	0,0	0,0	2,3
Contig_431088	pfam01373	Glyco_hydro_14,	2,4	4,4	0,0	2,3
Contig_217520	pfam00933	Glyco_hydro_3,	5,2	1,6	0,0	2,3
Contig_285283	pfam00933	Glyco_hydro_3,	6,8	0,0	0,0	2,3
Contig_269395	pfam02838	Glyco_hydro_20b,	3,1	3,7	0,0	2,3
Contig_284124	pfam00759	Glyco_hydro_9,	5,9	0,9	0,0	2,3
Contig_369224	pfam00728	Glyco_hydro_20,	3,1	3,7	0,0	2,3
Contig_455262	pfam02838	Glyco_hydro_20b,	4,7	2,1	0,0	2,3
Contig_460259	pfam02837	Glyco_hydro_2_N,	4,7	2,1	0,0	2,3
Contig_163444	pfam02836	Glyco_hydro_2_C,	0,7	4,3	1,8	2,3
Contig_323489	pfam00251	Glyco_hydro_32N,	0,0	6,8	0,0	2,3
Contig_330565	pfam00728	Glyco_hydro_20,	0,0	6,8	0,0	2,3
Contig_355652	pfam07748	Glyco_hydro_38C,	0,0	6,8	0,0	2,3
Contig_392672	pfam00232	Glyco_hydro_1,	0,0	6,8	0,0	2,3
Contig_401236	pfam03200	Glyco_hydro_63,	0,0	6,8	0,0	2,3
Contig_486348	pfam07971	Glyco_hydro_92,	4,8	1,9	0,0	2,3
Contig_492505	pfam07488	Glyco_hydro_67M,	4,6	2,2	0,0	2,3
Contig_153384	pfam12899	Glyco_hydro_100,	2,9	0,3	3,6	2,3
Contig_157923	pfam02446	Glyco_hydro_77,	5,2	1,6	0,0	2,3
Contig_407392	pfam00331	Glyco_hydro_10,	1,0	5,8	0,0	2,3
Contig_59091	pfam02057	Glyco_hydro_59,	1,2	2,5	3,0	2,2
Contig_293567	pfam03443	Glyco_hydro_61,	3,8	2,9	0,0	2,2
Contig_363026	pfam01532	Glyco_hydro_47,	3,7	3,1	0,0	2,2
Contig_370872	pfam07470	Glyco_hydro_88,	6,5	0,3	0,0	2,2
Contig_479556	pfam03659	Glyco_hydro_71,	4,7	2,0	0,0	2,2
Contig_535150	pfam02057	Glyco_hydro_59,	6,4	0,4	0,0	2,2
Contig_163282	pfam03200	Glyco_hydro_63,	3,1	3,7	0,0	2,2
Contig_307949	pfam03632	Glyco_hydro_65m,	4,2	2,5	0,0	2,2
Contig_326607	pfam01055	Glyco_hydro_31,	2,4	4,3	0,0	2,2
Contig_538246	pfam00232	Glyco_hydro_1,	6,7	0,0	0,0	2,2
Contig_487299	pfam07748	Glyco_hydro_38C,	4,6	2,1	0,0	2,2
Contig_354400	pfam01074	Glyco_hydro_38,	0,5	6,3	0,0	2,2
Contig_335776	pfam01301	Glyco_hydro_35,	5,8	0,9	0,0	2,2
Contig_174117	pfam00331	Glyco_hydro_10,	1,7	5,0	0,0	2,2
Contig_559477	pfam00232	Glyco_hydro_1,	6,7	0,0	0,0	2,2
Contig_65289	pfam00703	Glyco_hydro_2,	4,0	2,7	0,0	2,2
Contig_353591	pfam01074	Glyco_hydro_38,	0,0	6,7	0,0	2,2
Contig_388182	pfam02324	Glyco_hydro_70,	5,6	1,1	0,0	2,2
Contig_540876	pfam00232	Glyco_hydro_1,	6,7	0,0	0,0	2,2
Contig_74727	pfam01055	Glyco_hydro_31,	2,0	3,3	1,4	2,2
Contig_233498	pfam08306	Glyco_hydro_98M,	1,4	5,2	0,0	2,2
Contig_245346	pfam00332	Glyco_hydro_17,	0,7	6,0	0,0	2,2

Contig_253742	pfam00295	Glyco_hydro_28,	2,8	3,8	0,0	2,2
Contig_450848	pfam02011	Glyco_hydro_48,	1,4	3,6	1,7	2,2
Contig_95451	pfam00331	Glyco_hydro_10,	0,0	3,1	3,6	2,2
Contig_160363	pfam01055	Glyco_hydro_31,	5,6	1,0	0,0	2,2
Contig_315019	pfam03659	Glyco_hydro_71,	0,0	6,7	0,0	2,2
Contig_560003	pfam10566	Glyco_hydro_97,	6,7	0,0	0,0	2,2
Contig_52802	pfam00331	Glyco_hydro_10,	2,2	4,4	0,0	2,2
Contig_341023	pfam00723	Glyco_hydro_15,	1,1	5,5	0,0	2,2
Contig_134197	pfam12899	Glyco_hydro_100,	0,0	6,6	0,0	2,2
Contig_500558	pfam04616	Glyco_hydro_43,	4,8	1,8	0,0	2,2
Contig_501606	pfam01055	Glyco_hydro_31,	5,1	1,5	0,0	2,2
Contig_242063	pfam02449	Glyco_hydro_42,	1,5	5,1	0,0	2,2
Contig_397305	pfam00722	Glyco_hydro_16,	0,0	6,6	0,0	2,2
Contig_449071	pfam00723	Glyco_hydro_15,	6,6	0,0	0,0	2,2
Contig_115680	pfam02011	Glyco_hydro_48,	2,1	4,5	0,0	2,2
Contig_281401	pfam00232	Glyco_hydro_1,	4,7	1,9	0,0	2,2
Contig_492882	pfam00232	Glyco_hydro_1,	4,7	1,9	0,0	2,2
Contig_45328	pfam07745	Glyco_hydro_53,	0,8	5,8	0,0	2,2
Contig_171413	pfam07748	Glyco_hydro_38C,	4,1	2,5	0,0	2,2
Contig_261075	pfam00332	Glyco_hydro_17,	5,9	0,7	0,0	2,2
Contig_286401	pfam03659	Glyco_hydro_71,	0,0	6,6	0,0	2,2
Contig_421998	pfam12891	Glyco_hydro_44,	5,9	0,7	0,0	2,2
Contig_427148	pfam00840	Glyco_hydro_7,	0,0	3,6	3,0	2,2
Contig_512210	pfam00759	Glyco_hydro_9,	4,1	2,5	0,0	2,2
Contig_548752	pfam02449	Glyco_hydro_42,	6,6	0,0	0,0	2,2
Contig_575626	pfam03659	Glyco_hydro_71,	6,6	0,0	0,0	2,2
Contig_413827	pfam02837	Glyco_hydro_2_N,	0,0	6,6	0,0	2,2
Contig_457783	pfam07488	Glyco_hydro_67M,	5,3	1,3	0,0	2,2
Contig_327201	pfam01074	Glyco_hydro_38,	5,1	1,5	0,0	2,2
Contig_247504	pfam00728	Glyco_hydro_20,	2,6	4,0	0,0	2,2
Contig_313725	pfam07971	Glyco_hydro_92,	2,6	4,0	0,0	2,2
Contig_410683	pfam08532	Glyco_hydro_42M,	1,0	5,6	0,0	2,2
Contig_452180	pfam01055	Glyco_hydro_31,	2,3	4,2	0,0	2,2
Contig_507532	pfam03065	Glyco_hydro_57,	2,6	4,0	0,0	2,2
Contig_534271	pfam00232	Glyco_hydro_1,	0,0	6,6	0,0	2,2
Contig_302507	pfam01532	Glyco_hydro_47,	0,0	6,6	0,0	2,2
Contig_405968	pfam03636	Glyco_hydro_65N,	0,0	6,6	0,0	2,2
Contig_84390	pfam00704	Glyco_hydro_18,	5,5	1,1	0,0	2,2
Contig_190756	pfam00295	Glyco_hydro_28,	4,1	2,5	0,0	2,2
Contig_396315	pfam07971	Glyco_hydro_92,	4,7	1,9	0,0	2,2
Contig_415288	pfam03639	Glyco_hydro_81,	2,5	4,0	0,0	2,2
Contig_418953	pfam02449	Glyco_hydro_42,	4,7	1,9	0,0	2,2
Contig_534478	pfam00704	Glyco_hydro_18,	6,5	0,0	0,0	2,2
Contig_575335	pfam02011	Glyco_hydro_48,	6,5	0,0	0,0	2,2
Contig_464466	pfam00251	Glyco_hydro_32N,	3,3	3,2	0,0	2,2
Contig_487314	pfam04616	Glyco_hydro_43,	4,1	2,5	0,0	2,2
Contig_156887	pfam03639	Glyco_hydro_81,	0,0	6,5	0,0	2,2
Contig_158025	pfam00232	Glyco_hydro_1,	3,0	3,6	0,0	2,2
Contig_273257	pfam03639	Glyco_hydro_81,	0,0	6,5	0,0	2,2
Contig_321706	pfam01055	Glyco_hydro_31,	1,4	5,1	0,0	2,2
Contig_350595	pfam01229	Glyco_hydro_39,	5,0	1,5	0,0	2,2
Contig_400175	pfam01055	Glyco_hydro_31,	0,0	6,5	0,0	2,2
Contig_40657	pfam00232	Glyco_hydro_1,	0,6	2,7	3,2	2,2
Contig_83729	pfam00332	Glyco_hydro_17,	0,5	4,8	1,2	2,2
Contig_105068	pfam07745	Glyco_hydro_53,	4,8	1,7	0,0	2,2
Contig_197738	pfam00728	Glyco_hydro_20,	0,0	6,5	0,0	2,2
Contig_515245	pfam02446	Glyco_hydro_77,	6,5	0,0	0,0	2,2
Contig_305316	pfam00295	Glyco_hydro_28,	0,0	6,5	0,0	2,2
Contig_307776	pfam00704	Glyco_hydro_18,	2,9	3,6	0,0	2,2
Contig_156880	pfam12899	Glyco_hydro_100,	6,5	0,0	0,0	2,2
Contig_508807	pfam03639	Glyco_hydro_81,	2,5	4,0	0,0	2,2
Contig_168799	pfam07488	Glyco_hydro_67M,	6,5	0,0	0,0	2,2
Contig_422992	pfam01270	Glyco_hydro_8,	6,5	0,0	0,0	2,2
Contig_471133	pfam03644	Glyco_hydro_85,	1,6	4,9	0,0	2,2
Contig_280008	pfam07470	Glyco_hydro_88,	6,1	0,4	0,0	2,2
Contig_325904	pfam03648	Glyco_hydro_67N,	2,0	4,4	0,0	2,1
Contig_364469	pfam01055	Glyco_hydro_31,	4,0	2,4	0,0	2,1
Contig_469699	pfam07745	Glyco_hydro_53,	4,3	2,2	0,0	2,1
Contig_581435	pfam00704	Glyco_hydro_18,	6,4	0,0	0,0	2,1
Contig_228192	pfam00840	Glyco_hydro_7,	0,0	2,1	4,3	2,1
Contig_405503	pfam03512	Glyco_hydro_52,	0,0	6,4	0,0	2,1

Contig_572202	pfam01301	Glyco_hydro_35,	6,4	0,0	0,0	2,1
Contig_210982	pfam07470	Glyco_hydro_88,	3,5	3,0	0,0	2,1
Contig_308357	pfam03198	Glyco_hydro_72,	2,1	4,4	0,0	2,1
Contig_365568	pfam03639	Glyco_hydro_81,	4,7	1,7	0,0	2,1
Contig_568263	pfam02156	Glyco_hydro_26,	5,7	0,8	0,0	2,1
Contig_149846	pfam00232	Glyco_hydro_1,	5,5	0,9	0,0	2,1
Contig_560172	pfam07748	Glyco_hydro_38C,	5,7	0,7	0,0	2,1
Contig_278705	pfam01532	Glyco_hydro_47,	1,7	4,7	0,0	2,1
Contig_323592	pfam00722	Glyco_hydro_16,	1,6	4,8	0,0	2,1
Contig_454291	pfam00182	Glyco_hydro_19,	1,6	4,8	0,0	2,1
Contig_372373	pfam01532	Glyco_hydro_47,	0,0	6,4	0,0	2,1
Contig_480061	pfam00722	Glyco_hydro_16,	4,0	2,4	0,0	2,1
Contig_139462	pfam00232	Glyco_hydro_1,	5,5	0,8	0,0	2,1
Contig_149497	pfam03718	Glyco_hydro_49,	0,0	2,7	3,7	2,1
Contig_186228	pfam01074	Glyco_hydro_38,	0,9	5,5	0,0	2,1
Contig_550728	pfam03200	Glyco_hydro_63,	6,4	0,0	0,0	2,1
Contig_25545	pfam00703	Glyco_hydro_2,	2,4	3,1	0,9	2,1
Contig_452710	pfam03664	Glyco_hydro_62,	6,4	0,0	0,0	2,1
Contig_534679	pfam00704	Glyco_hydro_18,	6,4	0,0	0,0	2,1
Contig_38930	pfam03659	Glyco_hydro_71,	6,4	0,0	0,0	2,1
Contig_412465	pfam00251	Glyco_hydro_32N,	3,6	2,8	0,0	2,1
Contig_415901	pfam13199	Glyco_hydro_66,	4,0	2,4	0,0	2,1
Contig_107337	pfam00933	Glyco_hydro_3,	1,4	3,3	1,7	2,1
Contig_313673	pfam07971	Glyco_hydro_92,	1,1	5,2	0,0	2,1
Contig_495067	pfam12905	Glyco_hydro_101,	4,5	1,8	0,0	2,1
Contig_248347	pfam00722	Glyco_hydro_16,	0,0	6,3	0,0	2,1
Contig_112295	pfam01055	Glyco_hydro_31,	2,9	3,5	0,0	2,1
Contig_163031	pfam03200	Glyco_hydro_63,	0,9	5,4	0,0	2,1
Contig_314937	pfam08532	Glyco_hydro_42M,	3,1	3,2	0,0	2,1
Contig_401775	pfam07748	Glyco_hydro_38C,	2,9	3,5	0,0	2,1
Contig_491265	pfam03639	Glyco_hydro_81,	2,1	4,2	0,0	2,1
Contig_299074	pfam00728	Glyco_hydro_20,	2,3	4,1	0,0	2,1
Contig_312310	pfam03718	Glyco_hydro_49,	1,2	5,1	0,0	2,1
Contig_29596	pfam00295	Glyco_hydro_28,	1,9	4,5	0,0	2,1
Contig_161655	pfam00728	Glyco_hydro_20,	6,3	0,0	0,0	2,1
Contig_316402	pfam01074	Glyco_hydro_38,	0,0	6,3	0,0	2,1
Contig_403598	pfam01055	Glyco_hydro_31,	4,7	1,0	0,6	2,1
Contig_309945	pfam03200	Glyco_hydro_63,	3,9	2,4	0,0	2,1
Contig_446303	pfam01074	Glyco_hydro_38,	0,0	6,3	0,0	2,1
Contig_166592	pfam00704	Glyco_hydro_18,	2,9	3,4	0,0	2,1
Contig_254725	pfam04616	Glyco_hydro_43,	0,0	6,3	0,0	2,1
Contig_363139	pfam01055	Glyco_hydro_31,	1,5	4,8	0,0	2,1
Contig_455930	pfam12891	Glyco_hydro_44,	5,8	0,5	0,0	2,1
Contig_188362	pfam01630	Glyco_hydro_56,	5,9	0,4	0,0	2,1
Contig_360863	pfam01055	Glyco_hydro_31,	0,0	6,3	0,0	2,1
Contig_508032	pfam03200	Glyco_hydro_63,	0,0	1,7	4,6	2,1
Contig_554164	pfam03639	Glyco_hydro_81,	3,6	2,7	0,0	2,1
Contig_269644	pfam10566	Glyco_hydro_97,	6,3	0,0	0,0	2,1
Contig_325950	pfam01915	Glyco_hydro_3_C,	3,4	2,9	0,0	2,1
Contig_334525	pfam00295	Glyco_hydro_28,	4,6	1,7	0,0	2,1
Contig_346688	pfam00840	Glyco_hydro_7,	0,0	6,3	0,0	2,1
Contig_364391	pfam02837	Glyco_hydro_2_N,	0,0	6,3	0,0	2,1
Contig_92263	pfam01532	Glyco_hydro_47,	2,8	2,4	1,0	2,1
Contig_342708	pfam00704	Glyco_hydro_18,	2,8	3,4	0,0	2,1
Contig_78458	pfam00704	Glyco_hydro_18,	3,8	0,0	2,4	2,1
Contig_368894	pfam00722	Glyco_hydro_16,	4,8	1,4	0,0	2,1
Contig_264764	pfam03639	Glyco_hydro_81,	3,5	2,8	0,0	2,1
Contig_323114	pfam00704	Glyco_hydro_18,	3,3	3,0	0,0	2,1
Contig_366420	pfam02435	Glyco_hydro_68,	1,4	4,9	0,0	2,1
Contig_303045	pfam01373	Glyco_hydro_14,	2,6	3,6	0,0	2,1
Contig_371903	pfam03065	Glyco_hydro_57,	1,0	5,2	0,0	2,1
Contig_577452	pfam01532	Glyco_hydro_47,	6,2	0,0	0,0	2,1
Contig_172307	pfam00728	Glyco_hydro_20,	0,0	6,2	0,0	2,1
Contig_239935	pfam00728	Glyco_hydro_20,	0,0	6,2	0,0	2,1
Contig_537032	pfam00704	Glyco_hydro_18,	6,2	0,0	0,0	2,1
Contig_566055	pfam00457	Glyco_hydro_11,	6,2	0,0	0,0	2,1
Contig_117931	pfam01055	Glyco_hydro_31,	5,4	0,8	0,0	2,1
Contig_39827	pfam01074	Glyco_hydro_38,	2,8	3,4	0,0	2,1
Contig_508566	pfam03065	Glyco_hydro_57,	0,0	6,2	0,0	2,1
Contig_266160	pfam00332	Glyco_hydro_17,	0,0	6,2	0,0	2,1
Contig_298905	pfam01532	Glyco_hydro_47,	2,5	3,7	0,0	2,1

Contig_363679	pfam10566	Glyco_hydro_97,	0,0	6,2	0,0	2,1
Contig_244950	pfam03200	Glyco_hydro_63,	1,2	5,0	0,0	2,1
Contig_32284	pfam00728	Glyco_hydro_20,	2,3	3,0	0,9	2,1
Contig_503752	pfam00232	Glyco_hydro_1,	4,4	1,8	0,0	2,1
Contig_532692	pfam01301	Glyco_hydro_35,	6,1	0,0	0,0	2,0
Contig_536910	pfam00759	Glyco_hydro_9,	6,1	0,0	0,0	2,0
Contig_144694	pfam02836	Glyco_hydro_2_C,	0,0	4,3	1,8	2,0
Contig_385988	pfam07477	Glyco_hydro_67C,	4,7	1,4	0,0	2,0
Contig_409900	pfam00232	Glyco_hydro_1,	4,4	1,8	0,0	2,0
Contig_448823	pfam02055	Glyco_hydro_30,	3,2	2,9	0,0	2,0
Contig_275590	pfam03639	Glyco_hydro_81,	1,5	2,8	1,8	2,0
Contig_298142	pfam07748	Glyco_hydro_38C,	3,1	3,1	0,0	2,0
Contig_325371	pfam03200	Glyco_hydro_63,	3,6	2,6	0,0	2,0
Contig_558634	pfam00704	Glyco_hydro_18,	6,1	0,0	0,0	2,0
Contig_194374	pfam02057	Glyco_hydro_59,	1,1	5,1	0,0	2,0
Contig_246425	pfam00704	Glyco_hydro_18,	1,2	4,9	0,0	2,0
Contig_263773	pfam03718	Glyco_hydro_49,	0,0	6,1	0,0	2,0
Contig_329659	pfam07470	Glyco_hydro_88,	3,4	2,7	0,0	2,0
Contig_369761	pfam01301	Glyco_hydro_35,	3,5	2,6	0,0	2,0
Contig_406861	pfam03636	Glyco_hydro_65N,	4,1	1,9	0,0	2,0
Contig_446367	pfam02055	Glyco_hydro_30,	4,7	1,4	0,0	2,0
Contig_538619	pfam07971	Glyco_hydro_92,	6,1	0,0	0,0	2,0
Contig_578593	pfam03198	Glyco_hydro_72,	1,4	3,0	1,7	2,0
Contig_7352	pfam03200	Glyco_hydro_63,	3,2	1,0	2,0	2,0
Contig_252548	pfam02446	Glyco_hydro_77,	2,5	3,6	0,0	2,0
Contig_277945	pfam01074	Glyco_hydro_38,	3,0	3,0	0,0	2,0
Contig_151929	pfam03200	Glyco_hydro_63,	0,6	5,5	0,0	2,0
Contig_355440	pfam00728	Glyco_hydro_20,	0,7	5,4	0,0	2,0
Contig_6845	pfam00840	Glyco_hydro_7,	1,6	0,5	4,0	2,0
Contig_48461	pfam00704	Glyco_hydro_18,	0,0	6,0	0,0	2,0
Contig_396808	pfam02836	Glyco_hydro_2_C,	0,0	6,0	0,0	2,0
Contig_506924	pfam00295	Glyco_hydro_28,	0,0	6,0	0,0	2,0
Contig_515557	pfam07971	Glyco_hydro_92,	5,2	0,8	0,0	2,0
Contig_234945	pfam00331	Glyco_hydro_10,	2,4	3,6	0,0	2,0
Contig_292199	pfam00759	Glyco_hydro_9,	5,5	0,6	0,0	2,0
Contig_543189	pfam08306	Glyco_hydro_98M,	6,0	0,0	0,0	2,0
Contig_265811	pfam07488	Glyco_hydro_67M,	4,8	1,2	0,0	2,0
Contig_570547	pfam03200	Glyco_hydro_63,	6,0	0,0	0,0	2,0
Contig_333575	pfam07488	Glyco_hydro_67M,	1,2	4,9	0,0	2,0
Contig_140183	pfam02446	Glyco_hydro_77,	0,0	0,0	6,0	2,0
Contig_454427	pfam07971	Glyco_hydro_92,	2,7	3,3	0,0	2,0
Contig_512239	pfam02156	Glyco_hydro_26,	6,0	0,0	0,0	2,0
Contig_141602	pfam00759	Glyco_hydro_9,	0,0	2,5	3,5	2,0
Contig_327380	pfam00728	Glyco_hydro_20,	5,2	0,8	0,0	2,0
Contig_496300	pfam00232	Glyco_hydro_1,	5,2	0,8	0,0	2,0
Contig_198212	pfam01532	Glyco_hydro_47,	0,0	6,0	0,0	2,0
Contig_233146	pfam00232	Glyco_hydro_1,	0,0	6,0	0,0	2,0
Contig_163280	pfam03200	Glyco_hydro_63,	3,7	2,2	0,0	2,0
Contig_310432	pfam00457	Glyco_hydro_11,	1,0	4,9	0,0	2,0
Contig_505322	pfam07971	Glyco_hydro_92,	0,0	6,0	0,0	2,0
Contig_92182	pfam01055	Glyco_hydro_31,	2,6	0,0	3,3	2,0
Contig_92487	pfam00295	Glyco_hydro_28,	6,0	0,0	0,0	2,0
Contig_153770	pfam07748	Glyco_hydro_38C,	1,3	4,7	0,0	2,0
Contig_517375	pfam00332	Glyco_hydro_17,	6,0	0,0	0,0	2,0
Contig_534430	pfam01074	Glyco_hydro_38,	6,0	0,0	0,0	2,0
Contig_526810	pfam01301	Glyco_hydro_35,	3,0	3,0	0,0	2,0
Contig_241546	pfam00332	Glyco_hydro_17,	1,3	4,7	0,0	2,0
Contig_193768	pfam07748	Glyco_hydro_38C,	4,6	1,4	0,0	2,0
Contig_322306	pfam00759	Glyco_hydro_9,	4,6	1,4	0,0	2,0
Contig_453599	pfam02837	Glyco_hydro_2_N,	2,6	3,4	0,0	2,0
Contig_146000	pfam13199	Glyco_hydro_66,	1,0	2,5	2,4	2,0
Contig_91679	pfam07971	Glyco_hydro_92,	0,0	3,9	2,0	2,0
Contig_301126	pfam00295	Glyco_hydro_28,	1,1	2,0	2,8	2,0
Contig_368722	pfam08307	Glyco_hydro_98C,	5,3	0,6	0,0	2,0
Contig_75890	pfam01630	Glyco_hydro_56,	4,2	1,7	0,0	2,0
Contig_163910	pfam03663	Glyco_hydro_76,	5,9	0,0	0,0	2,0
Contig_327084	pfam12905	Glyco_hydro_101,	0,0	5,9	0,0	2,0
Contig_336346	pfam01229	Glyco_hydro_39,	5,9	0,0	0,0	2,0
Contig_377563	pfam00332	Glyco_hydro_17,	0,0	5,9	0,0	2,0
Contig_490488	pfam02055	Glyco_hydro_30,	3,4	2,5	0,0	2,0
Contig_544137	pfam00840	Glyco_hydro_7,	5,9	0,0	0,0	2,0

Contig_402148	pfam02836	Glyco_hydro_2_C,	5,1	0,8	0,0	2,0
Contig_203458	pfam04616	Glyco_hydro_43,	3,3	2,6	0,0	2,0
Contig_113502	pfam02449	Glyco_hydro_42,	0,0	0,8	5,1	2,0
Contig_344836	pfam02837	Glyco_hydro_2_N,	5,1	0,8	0,0	2,0
Contig_427814	pfam00840	Glyco_hydro_7,	5,9	0,0	0,0	2,0
Contig_252631	pfam03639	Glyco_hydro_81,	0,0	5,9	0,0	2,0
Contig_482505	pfam00722	Glyco_hydro_16,	3,1	2,8	0,0	2,0
Contig_255798	pfam01055	Glyco_hydro_31,	4,7	1,1	0,0	1,9
Contig_359571	pfam00332	Glyco_hydro_17,	1,5	4,4	0,0	1,9
Contig_497171	pfam00232	Glyco_hydro_1,	3,2	2,6	0,0	1,9
Contig_24677	pfam02324	Glyco_hydro_70,	0,0	5,8	0,0	1,9
Contig_468639	pfam02837	Glyco_hydro_2_N,	5,1	0,8	0,0	1,9
Contig_520374	pfam00704	Glyco_hydro_18,	5,8	0,0	0,0	1,9
Contig_539212	pfam00704	Glyco_hydro_18,	5,8	0,0	0,0	1,9
Contig_294988	pfam00759	Glyco_hydro_9,	3,9	1,9	0,0	1,9
Contig_331839	pfam03662	Glyco_hydro_79n,	0,0	5,8	0,0	1,9
Contig_121262	pfam00182	Glyco_hydro_19,	1,0	4,8	0,0	1,9
Contig_252575	pfam01055	Glyco_hydro_31,	0,0	5,8	0,0	1,9
Contig_307006	pfam02836	Glyco_hydro_2_C,	0,0	5,8	0,0	1,9
Contig_394777	pfam03718	Glyco_hydro_49,	0,0	5,8	0,0	1,9
Contig_253179	pfam02015	Glyco_hydro_45,	3,0	2,8	0,0	1,9
Contig_284097	pfam03639	Glyco_hydro_81,	0,8	5,0	0,0	1,9
Contig_332158	pfam02435	Glyco_hydro_68,	3,0	2,7	0,0	1,9
Contig_353637	pfam01532	Glyco_hydro_47,	1,3	4,5	0,0	1,9
Contig_564951	pfam13647	Glyco_hydro_80,	5,8	0,0	0,0	1,9
Contig_324377	pfam02324	Glyco_hydro_70,	2,9	2,9	0,0	1,9
Contig_408910	pfam03198	Glyco_hydro_72,	2,6	3,2	0,0	1,9
Contig_298504	pfam07748	Glyco_hydro_38C,	0,0	5,8	0,0	1,9
Contig_395233	pfam02446	Glyco_hydro_77,	3,8	2,0	0,0	1,9
Contig_429138	pfam00457	Glyco_hydro_11,	4,0	1,8	0,0	1,9
Contig_435240	pfam07971	Glyco_hydro_92,	0,0	5,7	0,0	1,9
Contig_449484	pfam00728	Glyco_hydro_20,	4,3	1,4	0,0	1,9
Contig_492413	pfam02449	Glyco_hydro_42,	5,2	0,5	0,0	1,9
Contig_217523	pfam02837	Glyco_hydro_2_N,	4,0	1,7	0,0	1,9
Contig_287117	pfam01055	Glyco_hydro_31,	3,0	2,7	0,0	1,9
Contig_495387	pfam07971	Glyco_hydro_92,	5,7	0,0	0,0	1,9
Contig_259732	pfam00295	Glyco_hydro_28,	0,0	5,7	0,0	1,9
Contig_343243	pfam00251	Glyco_hydro_32N,	3,6	2,1	0,0	1,9
Contig_580523	pfam02449	Glyco_hydro_42,	5,7	0,0	0,0	1,9
Contig_3783	pfam07971	Glyco_hydro_92,	5,7	0,0	0,0	1,9
Contig_238114	pfam11975	Glyco_hydro_4C,	0,0	5,7	0,0	1,9
Contig_325638	pfam02011	Glyco_hydro_48,	4,2	1,5	0,0	1,9
Contig_133556	pfam03200	Glyco_hydro_63,	0,7	1,8	3,3	1,9
Contig_211793	pfam00704	Glyco_hydro_18,	0,7	5,0	0,0	1,9
Contig_196787	pfam01670	Glyco_hydro_12,	0,0	5,7	0,0	1,9
Contig_264130	pfam00728	Glyco_hydro_20,	1,4	4,3	0,0	1,9
Contig_359619	pfam07745	Glyco_hydro_53,	0,0	5,7	0,0	1,9
Contig_559040	pfam04616	Glyco_hydro_43,	5,7	0,0	0,0	1,9
Contig_178252	pfam01532	Glyco_hydro_47,	3,0	2,7	0,0	1,9
Contig_554311	pfam07748	Glyco_hydro_38C,	5,4	0,3	0,0	1,9
Contig_31885	pfam10566	Glyco_hydro_97,	4,9	0,7	0,0	1,9
Contig_60365	pfam00182	Glyco_hydro_19,	1,9	3,8	0,0	1,9
Contig_215640	pfam02057	Glyco_hydro_59,	3,1	2,5	0,0	1,9
Contig_492273	pfam01055	Glyco_hydro_31,	4,7	1,0	0,0	1,9
Contig_493449	pfam00232	Glyco_hydro_1,	4,0	1,6	0,0	1,9
Contig_441448	pfam03065	Glyco_hydro_57,	5,7	0,0	0,0	1,9
Contig_476457	pfam12899	Glyco_hydro_100,	5,7	0,0	0,0	1,9
Contig_576140	pfam03663	Glyco_hydro_76,	5,7	0,0	0,0	1,9
Contig_197562	pfam07971	Glyco_hydro_92,	0,0	5,6	0,0	1,9
Contig_274423	pfam07470	Glyco_hydro_88,	0,0	5,6	0,0	1,9
Contig_346342	pfam07971	Glyco_hydro_92,	0,0	5,6	0,0	1,9
Contig_404992	pfam03659	Glyco_hydro_71,	1,2	4,4	0,0	1,9
Contig_163682	pfam03632	Glyco_hydro_65m,	0,0	5,6	0,0	1,9
Contig_387133	pfam01055	Glyco_hydro_31,	0,0	5,6	0,0	1,9
Contig_391947	pfam03200	Glyco_hydro_63,	3,2	2,4	0,0	1,9
Contig_460351	pfam02156	Glyco_hydro_26,	0,0	5,6	0,0	1,9
Contig_277196	pfam02056	Glyco_hydro_4,	3,1	2,5	0,0	1,9
Contig_518226	pfam13199	Glyco_hydro_66,	4,8	0,8	0,0	1,9
Contig_147693	pfam01532	Glyco_hydro_47,	1,4	4,2	0,0	1,9
Contig_414348	pfam11790	Glyco_hydro_cc,	2,6	3,1	0,0	1,9
Contig_413316	pfam00332	Glyco_hydro_17,	4,0	1,6	0,0	1,9

Contig_556909	pfam03644	Glyco_hydro_85,	4,3	1,3	0,0	1,9
Contig_356270	pfam00232	Glyco_hydro_1,	0,0	5,6	0,0	1,9
Contig_362744	pfam03639	Glyco_hydro_81,	0,0	5,6	0,0	1,9
Contig_387620	pfam00703	Glyco_hydro_2,	1,0	4,6	0,0	1,9
Contig_507547	pfam00728	Glyco_hydro_20,	0,0	5,6	0,0	1,9
Contig_533618	pfam03200	Glyco_hydro_63,	5,1	0,5	0,0	1,9
Contig_83304	pfam02057	Glyco_hydro_59,	3,5	2,1	0,0	1,9
Contig_344172	pfam03200	Glyco_hydro_63,	2,7	2,9	0,0	1,9
Contig_357706	pfam13199	Glyco_hydro_66,	2,5	3,0	0,0	1,9
Contig_458518	pfam08244	Glyco_hydro_32C,	3,2	2,4	0,0	1,9
Contig_486362	pfam03639	Glyco_hydro_81,	4,1	1,5	0,0	1,9
Contig_23293	pfam07748	Glyco_hydro_38C,	0,0	2,3	3,2	1,9
Contig_91203	pfam00722	Glyco_hydro_16,	4,0	1,6	0,0	1,9
Contig_93087	pfam03198	Glyco_hydro_72,	1,1	1,9	2,6	1,9
Contig_450816	pfam03200	Glyco_hydro_63,	3,8	1,7	0,0	1,9
Contig_144025	pfam00723	Glyco_hydro_15,	5,5	0,0	0,0	1,8
Contig_248983	pfam07971	Glyco_hydro_92,	2,2	3,3	0,0	1,8
Contig_400989	pfam03644	Glyco_hydro_85,	1,0	4,5	0,0	1,8
Contig_469467	pfam03200	Glyco_hydro_63,	3,1	2,5	0,0	1,8
Contig_505449	pfam00704	Glyco_hydro_18,	4,4	1,2	0,0	1,8
Contig_551838	pfam00933	Glyco_hydro_3,	5,5	0,0	0,0	1,8
Contig_328040	pfam02057	Glyco_hydro_59,	0,0	5,5	0,0	1,8
Contig_248307	pfam00933	Glyco_hydro_3,	2,9	2,6	0,0	1,8
Contig_256302	pfam01301	Glyco_hydro_35,	3,1	2,4	0,0	1,8
Contig_348062	pfam00759	Glyco_hydro_9,	1,1	4,5	0,0	1,8
Contig_410851	pfam00331	Glyco_hydro_10,	0,0	5,5	0,0	1,8
Contig_195377	pfam00295	Glyco_hydro_28,	1,8	3,7	0,0	1,8
Contig_236424	pfam00728	Glyco_hydro_20,	2,9	2,6	0,0	1,8
Contig_322353	pfam03200	Glyco_hydro_63,	5,5	0,0	0,0	1,8
Contig_449905	pfam03659	Glyco_hydro_71,	2,2	3,3	0,0	1,8
Contig_580651	pfam03639	Glyco_hydro_81,	5,5	0,0	0,0	1,8
Contig_94609	pfam07971	Glyco_hydro_92,	0,0	3,0	2,5	1,8
Contig_154573	pfam01630	Glyco_hydro_56,	3,4	0,0	2,1	1,8
Contig_163143	pfam02836	Glyco_hydro_2_C,	1,4	4,1	0,0	1,8
Contig_384358	pfam10566	Glyco_hydro_97,	2,9	2,6	0,0	1,8
Contig_94053	pfam00232	Glyco_hydro_1,	4,1	0,0	1,4	1,8
Contig_127985	pfam02837	Glyco_hydro_2_N,	4,6	0,9	0,0	1,8
Contig_321570	pfam00704	Glyco_hydro_18,	3,1	2,4	0,0	1,8
Contig_307218	pfam00182	Glyco_hydro_19,	4,1	1,4	0,0	1,8
Contig_65502	pfam00704	Glyco_hydro_18,	1,6	0,0	3,9	1,8
Contig_309159	pfam03512	Glyco_hydro_52,	0,0	5,5	0,0	1,8
Contig_319629	pfam02324	Glyco_hydro_70,	3,9	1,6	0,0	1,8
Contig_584211	pfam01074	Glyco_hydro_38,	4,2	1,3	0,0	1,8
Contig_415978	pfam01055	Glyco_hydro_31,	5,4	0,0	0,0	1,8
Contig_23382	pfam00703	Glyco_hydro_2,	1,9	1,2	2,4	1,8
Contig_66299	pfam02449	Glyco_hydro_42,	0,4	4,2	0,9	1,8
Contig_379814	pfam01532	Glyco_hydro_47,	1,6	3,8	0,0	1,8
Contig_385001	pfam03200	Glyco_hydro_63,	0,0	5,4	0,0	1,8
Contig_421878	pfam00933	Glyco_hydro_3,	0,0	5,4	0,0	1,8
Contig_24244	pfam07971	Glyco_hydro_92,	0,4	5,0	0,0	1,8
Contig_185854	pfam02156	Glyco_hydro_26,	0,0	5,4	0,0	1,8
Contig_393845	pfam07745	Glyco_hydro_53,	2,5	3,0	0,0	1,8
Contig_253989	pfam03662	Glyco_hydro_79n,	5,4	0,0	0,0	1,8
Contig_339212	pfam00933	Glyco_hydro_3,	0,0	5,4	0,0	1,8
Contig_335373	pfam00728	Glyco_hydro_20,	3,9	1,6	0,0	1,8
Contig_437473	pfam00722	Glyco_hydro_16,	2,2	3,2	0,0	1,8
Contig_496035	pfam02837	Glyco_hydro_2_N,	0,8	4,6	0,0	1,8
Contig_354604	pfam01301	Glyco_hydro_35,	0,0	5,4	0,0	1,8
Contig_442219	pfam01270	Glyco_hydro_8,	0,0	5,4	0,0	1,8
Contig_537611	pfam01055	Glyco_hydro_31,	5,4	0,0	0,0	1,8
Contig_574595	pfam04616	Glyco_hydro_43,	5,4	0,0	0,0	1,8
Contig_309162	pfam01915	Glyco_hydro_3_C,	4,5	0,9	0,0	1,8
Contig_361556	pfam00232	Glyco_hydro_1,	1,3	0,8	3,3	1,8
Contig_314654	pfam01630	Glyco_hydro_56,	2,4	2,9	0,0	1,8
Contig_342443	pfam00759	Glyco_hydro_9,	4,9	0,4	0,0	1,8
Contig_166491	pfam13199	Glyco_hydro_66,	3,7	1,6	0,0	1,8
Contig_491706	pfam03639	Glyco_hydro_81,	1,0	4,4	0,0	1,8
Contig_550513	pfam01532	Glyco_hydro_47,	3,1	2,3	0,0	1,8
Contig_473485	pfam02011	Glyco_hydro_48,	3,3	2,0	0,0	1,8
Contig_116432	pfam03644	Glyco_hydro_85,	3,3	2,0	0,0	1,8
Contig_259269	pfam00182	Glyco_hydro_19,	0,8	4,6	0,0	1,8

Contig_264432	pfam02055	Glyco_hydro_30,	0,0	5,3	0,0	1,8
Contig_221782	pfam03718	Glyco_hydro_49,	3,3	2,0	0,0	1,8
Contig_250884	pfam00722	Glyco_hydro_16,	1,2	4,2	0,0	1,8
Contig_466839	pfam03659	Glyco_hydro_71,	4,1	1,2	0,0	1,8
Contig_367775	pfam04616	Glyco_hydro_43,	0,5	4,8	0,0	1,8
Contig_474199	pfam03198	Glyco_hydro_72,	2,9	2,4	0,0	1,8
Contig_398466	pfam00704	Glyco_hydro_18,	1,7	3,6	0,0	1,8
Contig_324239	pfam02836	Glyco_hydro_2_C,	0,0	5,3	0,0	1,8
Contig_325019	pfam01055	Glyco_hydro_31,	0,0	5,3	0,0	1,8
Contig_454011	pfam03200	Glyco_hydro_63,	3,3	2,0	0,0	1,8
Contig_536235	pfam01301	Glyco_hydro_35,	5,3	0,0	0,0	1,8
Contig_579724	pfam00251	Glyco_hydro_32N,	0,0	5,3	0,0	1,8
Contig_472308	pfam00722	Glyco_hydro_16,	2,8	2,5	0,0	1,8
Contig_487486	pfam03659	Glyco_hydro_71,	2,8	2,5	0,0	1,8
Contig_52351	pfam00704	Glyco_hydro_18,	4,5	0,7	0,0	1,8
Contig_312110	pfam00840	Glyco_hydro_7,	0,8	4,5	0,0	1,8
Contig_54664	pfam02449	Glyco_hydro_42,	1,7	0,5	3,1	1,8
Contig_437261	pfam03200	Glyco_hydro_63,	3,0	2,3	0,0	1,8
Contig_330242	pfam13199	Glyco_hydro_66,	1,1	4,1	0,0	1,8
Contig_409067	pfam07748	Glyco_hydro_38C,	3,8	1,5	0,0	1,8
Contig_73193	pfam01183	Glyco_hydro_25,	2,8	1,0	1,4	1,8
Contig_213544	pfam01532	Glyco_hydro_47,	2,7	2,6	0,0	1,8
Contig_450539	pfam07477	Glyco_hydro_67C,	0,0	2,2	3,1	1,8
Contig_264173	pfam03200	Glyco_hydro_63,	5,2	0,0	0,0	1,7
Contig_470768	pfam00232	Glyco_hydro_1,	1,7	3,6	0,0	1,7
Contig_173959	pfam03639	Glyco_hydro_81,	2,7	2,5	0,0	1,7
Contig_516304	pfam07971	Glyco_hydro_92,	3,7	1,5	0,0	1,7
Contig_172583	pfam00933	Glyco_hydro_3,	1,9	3,4	0,0	1,7
Contig_193371	pfam13199	Glyco_hydro_66,	3,0	2,2	0,0	1,7
Contig_263256	pfam13199	Glyco_hydro_66,	4,4	0,9	0,0	1,7
Contig_275063	pfam01341	Glyco_hydro_6,	1,0	4,2	0,0	1,7
Contig_147152	pfam02055	Glyco_hydro_30,	3,0	2,2	0,0	1,7
Contig_312321	pfam07488	Glyco_hydro_67M,	0,0	5,2	0,0	1,7
Contig_534427	pfam00332	Glyco_hydro_17,	3,3	2,0	0,0	1,7
Contig_303746	pfam03200	Glyco_hydro_63,	0,0	5,2	0,0	1,7
Contig_485944	pfam12899	Glyco_hydro_100,	0,0	5,2	0,0	1,7
Contig_386601	pfam01229	Glyco_hydro_39,	3,0	2,2	0,0	1,7
Contig_473988	pfam02446	Glyco_hydro_77,	1,9	3,3	0,0	1,7
Contig_490550	pfam07971	Glyco_hydro_92,	2,7	2,5	0,0	1,7
Contig_583100	pfam04616	Glyco_hydro_43,	0,0	5,2	0,0	1,7
Contig_415290	pfam00704	Glyco_hydro_18,	0,0	5,2	0,0	1,7
Contig_502615	pfam13199	Glyco_hydro_66,	0,0	5,2	0,0	1,7
Contig_353657	pfam02838	Glyco_hydro_20b,	1,8	3,3	0,0	1,7
Contig_447822	pfam02055	Glyco_hydro_30,	5,2	0,0	0,0	1,7
Contig_522976	pfam00840	Glyco_hydro_7,	5,2	0,0	0,0	1,7
Contig_425808	pfam02011	Glyco_hydro_48,	3,2	1,9	0,0	1,7
Contig_559031	pfam00295	Glyco_hydro_28,	3,2	1,9	0,0	1,7
Contig_309868	pfam03065	Glyco_hydro_57,	0,0	5,1	0,0	1,7
Contig_90286	pfam11790	Glyco_hydro_cc,	1,7	3,5	0,0	1,7
Contig_118076	pfam02057	Glyco_hydro_59,	0,0	1,0	4,1	1,7
Contig_158935	pfam01055	Glyco_hydro_31,	3,7	1,5	0,0	1,7
Contig_173150	pfam03512	Glyco_hydro_52,	2,7	2,4	0,0	1,7
Contig_211773	pfam02156	Glyco_hydro_26,	4,7	0,5	0,0	1,7
Contig_470393	pfam03663	Glyco_hydro_76,	1,5	3,6	0,0	1,7
Contig_2593	pfam01229	Glyco_hydro_39,	0,0	5,1	0,0	1,7
Contig_268702	pfam01074	Glyco_hydro_38,	0,9	0,0	4,3	1,7
Contig_320495	pfam12899	Glyco_hydro_100,	1,0	4,1	0,0	1,7
Contig_190223	pfam03065	Glyco_hydro_57,	4,6	0,6	0,0	1,7
Contig_161066	pfam00251	Glyco_hydro_32N,	1,0	4,1	0,0	1,7
Contig_248025	pfam13199	Glyco_hydro_66,	0,0	5,1	0,0	1,7
Contig_94993	pfam12899	Glyco_hydro_100,	1,9	1,6	1,6	1,7
Contig_110119	pfam00232	Glyco_hydro_1,	3,6	1,5	0,0	1,7
Contig_161067	pfam00722	Glyco_hydro_16,	1,9	3,2	0,0	1,7
Contig_466405	pfam10566	Glyco_hydro_97,	2,5	2,6	0,0	1,7
Contig_112968	pfam00331	Glyco_hydro_10,	0,0	5,1	0,0	1,7
Contig_340295	pfam02449	Glyco_hydro_42,	0,7	4,4	0,0	1,7
Contig_151455	pfam04616	Glyco_hydro_43,	1,5	0,0	3,6	1,7
Contig_263430	pfam00704	Glyco_hydro_18,	4,2	0,9	0,0	1,7
Contig_511003	pfam03639	Glyco_hydro_81,	3,2	1,9	0,0	1,7
Contig_292433	pfam00251	Glyco_hydro_32N,	0,0	5,1	0,0	1,7
Contig_317344	pfam12899	Glyco_hydro_100,	0,0	5,1	0,0	1,7

Contig_392925	pfam03659	Glyco_hydro_71,	4,6	0,5	0,0	1,7
Contig_223201	pfam00759	Glyco_hydro_9,	3,1	1,9	0,0	1,7
Contig_282134	pfam01532	Glyco_hydro_47,	3,1	1,9	0,0	1,7
Contig_368609	pfam03200	Glyco_hydro_63,	1,5	3,6	0,0	1,7
Contig_88746	pfam03644	Glyco_hydro_85,	0,4	2,4	2,2	1,7
Contig_92476	pfam02057	Glyco_hydro_59,	2,3	2,8	0,0	1,7
Contig_473326	pfam12891	Glyco_hydro_44,	3,6	1,4	0,0	1,7
Contig_261518	pfam01055	Glyco_hydro_31,	0,0	5,0	0,0	1,7
Contig_285832	pfam00722	Glyco_hydro_16,	1,3	3,8	0,0	1,7
Contig_364134	pfam00728	Glyco_hydro_20,	0,0	5,0	0,0	1,7
Contig_457379	pfam13199	Glyco_hydro_66,	0,0	5,0	0,0	1,7
Contig_489363	pfam00759	Glyco_hydro_9,	0,0	5,0	0,0	1,7
Contig_47610	pfam02446	Glyco_hydro_77,	4,5	0,5	0,0	1,7
Contig_89852	pfam12899	Glyco_hydro_100,	0,0	5,0	0,0	1,7
Contig_402993	pfam12899	Glyco_hydro_100,	0,0	5,0	0,0	1,7
Contig_517625	pfam03639	Glyco_hydro_81,	5,0	0,0	0,0	1,7
Contig_494612	pfam01055	Glyco_hydro_31,	4,5	0,5	0,0	1,7
Contig_100791	pfam03512	Glyco_hydro_52,	5,0	0,0	0,0	1,7
Contig_538703	pfam00251	Glyco_hydro_32N,	5,0	0,0	0,0	1,7
Contig_278337	pfam03632	Glyco_hydro_65m,	1,0	4,0	0,0	1,7
Contig_219655	pfam13199	Glyco_hydro_66,	0,0	5,0	0,0	1,7
Contig_237905	pfam07488	Glyco_hydro_67M,	0,0	5,0	0,0	1,7
Contig_458050	pfam01055	Glyco_hydro_31,	0,0	5,0	0,0	1,7
Contig_479732	pfam01630	Glyco_hydro_56,	3,8	1,2	0,0	1,7
Contig_504880	pfam03718	Glyco_hydro_49,	0,0	5,0	0,0	1,7
Contig_93506	pfam00728	Glyco_hydro_20,	1,4	0,0	3,6	1,7
Contig_343821	pfam03648	Glyco_hydro_67N,	1,8	3,2	0,0	1,7
Contig_269008	pfam07971	Glyco_hydro_92,	2,1	2,9	0,0	1,7
Contig_323202	pfam00232	Glyco_hydro_1,	1,3	3,7	0,0	1,7
Contig_325921	pfam01532	Glyco_hydro_47,	2,6	2,4	0,0	1,7
Contig_419012	pfam07971	Glyco_hydro_92,	0,0	4,9	0,0	1,6
Contig_568724	pfam01055	Glyco_hydro_31,	4,9	0,0	0,0	1,6
Contig_241318	pfam03639	Glyco_hydro_81,	4,9	0,0	0,0	1,6
Contig_414889	pfam07477	Glyco_hydro_67C,	3,1	1,9	0,0	1,6
Contig_449258	pfam00295	Glyco_hydro_28,	2,5	2,5	0,0	1,6
Contig_482227	pfam07748	Glyco_hydro_38C,	4,9	0,0	0,0	1,6
Contig_515589	pfam07971	Glyco_hydro_92,	4,9	0,0	0,0	1,6
Contig_499997	pfam12899	Glyco_hydro_100,	0,0	4,9	0,0	1,6
Contig_78460	pfam03200	Glyco_hydro_63,	0,0	3,2	1,7	1,6
Contig_130554	pfam02057	Glyco_hydro_59,	0,0	4,9	0,0	1,6
Contig_152676	pfam01301	Glyco_hydro_35,	4,9	0,0	0,0	1,6
Contig_449902	pfam03200	Glyco_hydro_63,	2,2	2,7	0,0	1,6
Contig_484573	pfam02324	Glyco_hydro_70,	2,6	2,3	0,0	1,6
Contig_410947	pfam00840	Glyco_hydro_7,	0,7	4,2	0,0	1,6
Contig_134699	pfam00232	Glyco_hydro_1,	4,9	0,0	0,0	1,6
Contig_417093	pfam02837	Glyco_hydro_2_N,	2,0	2,9	0,0	1,6
Contig_254747	pfam03200	Glyco_hydro_63,	2,2	2,7	0,0	1,6
Contig_381929	pfam01074	Glyco_hydro_38,	4,9	0,0	0,0	1,6
Contig_505601	pfam03663	Glyco_hydro_76,	0,0	4,9	0,0	1,6
Contig_86950	pfam13199	Glyco_hydro_66,	1,9	2,9	0,0	1,6
Contig_263889	pfam04616	Glyco_hydro_43,	0,6	4,2	0,0	1,6
Contig_288035	pfam03718	Glyco_hydro_49,	1,1	3,7	0,0	1,6
Contig_313404	pfam02446	Glyco_hydro_77,	0,0	4,9	0,0	1,6
Contig_323353	pfam00759	Glyco_hydro_9,	0,0	3,1	1,8	1,6
Contig_395982	pfam01301	Glyco_hydro_35,	0,0	4,9	0,0	1,6
Contig_163459	pfam00933	Glyco_hydro_3,	3,7	1,1	0,0	1,6
Contig_98708	pfam00933	Glyco_hydro_3,	0,0	2,0	2,8	1,6
Contig_149296	pfam02056	Glyco_hydro_4,	2,4	0,0	2,4	1,6
Contig_161695	pfam02057	Glyco_hydro_59,	0,8	4,0	0,0	1,6
Contig_220764	pfam03659	Glyco_hydro_71,	0,0	4,8	0,0	1,6
Contig_427632	pfam00182	Glyco_hydro_19,	2,2	2,6	0,0	1,6
Contig_86569	pfam01301	Glyco_hydro_35,	0,0	4,8	0,0	1,6
Contig_87743	pfam02324	Glyco_hydro_70,	4,8	0,0	0,0	1,6
Contig_396515	pfam03632	Glyco_hydro_65m,	4,2	0,6	0,0	1,6
Contig_250917	pfam01074	Glyco_hydro_38,	1,9	2,9	0,0	1,6
Contig_411540	pfam00704	Glyco_hydro_18,	0,0	4,8	0,0	1,6
Contig_142496	pfam03639	Glyco_hydro_81,	0,8	0,0	4,0	1,6
Contig_347635	pfam03639	Glyco_hydro_81,	4,8	0,0	0,0	1,6
Contig_355038	pfam10566	Glyco_hydro_97,	2,4	2,4	0,0	1,6
Contig_560423	pfam03663	Glyco_hydro_76,	4,8	0,0	0,0	1,6
Contig_254771	pfam07971	Glyco_hydro_92,	0,0	4,8	0,0	1,6

Contig_460243	pfam00232	Glyco_hydro_1,	0,0	4,8	0,0	1,6
Contig_557782	pfam13199	Glyco_hydro_66,	2,2	2,6	0,0	1,6
Contig_360963	pfam03639	Glyco_hydro_81,	0,0	4,8	0,0	1,6
Contig_480917	pfam01373	Glyco_hydro_14,	3,3	1,5	0,0	1,6
Contig_383124	pfam00840	Glyco_hydro_7,	2,5	2,3	0,0	1,6
Contig_490095	pfam03644	Glyco_hydro_85,	0,0	4,8	0,0	1,6
Contig_469630	pfam02446	Glyco_hydro_77,	4,8	0,0	0,0	1,6
Contig_152932	pfam03639	Glyco_hydro_81,	4,7	0,0	0,0	1,6
Contig_548119	pfam00295	Glyco_hydro_28,	4,7	0,0	0,0	1,6
Contig_72702	pfam08307	Glyco_hydro_98C,	0,0	1,5	3,2	1,6
Contig_438645	pfam00728	Glyco_hydro_20,	3,0	1,8	0,0	1,6
Contig_192185	pfam12899	Glyco_hydro_100,	1,7	3,0	0,0	1,6
Contig_316550	pfam01301	Glyco_hydro_35,	1,4	3,3	0,0	1,6
Contig_147873	pfam00840	Glyco_hydro_7,	4,7	0,0	0,0	1,6
Contig_161789	pfam07971	Glyco_hydro_92,	4,7	0,0	0,0	1,6
Contig_181007	pfam00840	Glyco_hydro_7,	0,0	4,7	0,0	1,6
Contig_192783	pfam07470	Glyco_hydro_88,	0,0	4,7	0,0	1,6
Contig_412692	pfam07477	Glyco_hydro_67C,	0,0	4,7	0,0	1,6
Contig_494549	pfam07971	Glyco_hydro_92,	4,1	0,6	0,0	1,6
Contig_313051	pfam03662	Glyco_hydro_79n,	2,9	1,8	0,0	1,6
Contig_399890	pfam00759	Glyco_hydro_9,	4,7	0,0	0,0	1,6
Contig_443195	pfam02449	Glyco_hydro_42,	2,9	1,8	0,0	1,6
Contig_461671	pfam02836	Glyco_hydro_2_C,	3,9	0,8	0,0	1,6
Contig_338994	pfam00703	Glyco_hydro_2,	0,0	4,7	0,0	1,6
Contig_162372	pfam00759	Glyco_hydro_9,	2,1	2,5	0,0	1,6
Contig_499323	pfam07748	Glyco_hydro_38C,	0,0	4,7	0,0	1,6
Contig_496450	pfam00759	Glyco_hydro_9,	3,3	1,3	0,0	1,5
Contig_187540	pfam03644	Glyco_hydro_85,	0,0	4,6	0,0	1,5
Contig_286023	pfam03632	Glyco_hydro_65m,	0,0	4,6	0,0	1,5
Contig_213239	pfam07748	Glyco_hydro_38C,	3,2	1,4	0,0	1,5
Contig_314467	pfam00704	Glyco_hydro_18,	4,6	0,0	0,0	1,5
Contig_151111	pfam01229	Glyco_hydro_39,	3,1	1,5	0,0	1,5
Contig_462229	pfam02446	Glyco_hydro_77,	1,6	3,0	0,0	1,5
Contig_497993	pfam00295	Glyco_hydro_28,	0,0	4,6	0,0	1,5
Contig_504595	pfam03198	Glyco_hydro_72,	1,0	3,6	0,0	1,5
Contig_395851	pfam01055	Glyco_hydro_31,	0,0	4,6	0,0	1,5
Contig_467013	pfam03200	Glyco_hydro_63,	0,0	4,6	0,0	1,5
Contig_296060	pfam02446	Glyco_hydro_77,	1,2	3,5	0,0	1,5
Contig_439853	pfam02011	Glyco_hydro_48,	2,4	2,2	0,0	1,5
Contig_481858	pfam02446	Glyco_hydro_77,	2,4	2,2	0,0	1,5
Contig_210010	pfam01532	Glyco_hydro_47,	0,9	3,7	0,0	1,5
Contig_579524	pfam00704	Glyco_hydro_18,	4,1	0,5	0,0	1,5
Contig_338112	pfam03644	Glyco_hydro_85,	2,9	1,7	0,0	1,5
Contig_477380	pfam00840	Glyco_hydro_7,	1,0	3,6	0,0	1,5
Contig_164062	pfam03662	Glyco_hydro_79n,	2,9	1,7	0,0	1,5
Contig_280742	pfam02156	Glyco_hydro_26,	2,9	1,7	0,0	1,5
Contig_578667	pfam11790	Glyco_hydro_cc,	4,6	0,0	0,0	1,5
Contig_359534	pfam00728	Glyco_hydro_20,	2,2	2,4	0,0	1,5
Contig_404805	pfam12899	Glyco_hydro_100,	2,4	2,2	0,0	1,5
Contig_406971	pfam02837	Glyco_hydro_2_N,	0,0	4,6	0,0	1,5
Contig_174320	pfam03663	Glyco_hydro_76,	2,9	1,7	0,0	1,5
Contig_347602	pfam00933	Glyco_hydro_3,	2,6	2,0	0,0	1,5
Contig_571704	pfam01301	Glyco_hydro_35,	4,6	0,0	0,0	1,5
Contig_504571	pfam03200	Glyco_hydro_63,	1,3	3,2	0,0	1,5
Contig_196649	pfam03065	Glyco_hydro_57,	0,6	4,0	0,0	1,5
Contig_383226	pfam02435	Glyco_hydro_68,	0,0	4,6	0,0	1,5
Contig_376046	pfam07971	Glyco_hydro_92,	0,0	4,5	0,0	1,5
Contig_441227	pfam00331	Glyco_hydro_10,	0,0	4,5	0,0	1,5
Contig_451454	pfam04616	Glyco_hydro_43,	2,4	2,2	0,0	1,5
Contig_435496	pfam00331	Glyco_hydro_10,	0,6	3,9	0,0	1,5
Contig_487478	pfam00232	Glyco_hydro_1,	2,5	2,0	0,0	1,5
Contig_536426	pfam03639	Glyco_hydro_81,	4,5	0,0	0,0	1,5
Contig_29398	pfam01074	Glyco_hydro_38,	4,5	0,0	0,0	1,5
Contig_312078	pfam00251	Glyco_hydro_32N,	3,5	1,0	0,0	1,5
Contig_38214	pfam01055	Glyco_hydro_31,	1,1	3,4	0,0	1,5
Contig_247530	pfam01055	Glyco_hydro_31,	0,0	4,5	0,0	1,5
Contig_433297	pfam03644	Glyco_hydro_85,	2,4	2,1	0,0	1,5
Contig_477882	pfam02324	Glyco_hydro_70,	0,0	4,5	0,0	1,5
Contig_158925	pfam01055	Glyco_hydro_31,	2,0	2,5	0,0	1,5
Contig_313536	pfam13199	Glyco_hydro_66,	0,0	4,5	0,0	1,5
Contig_338706	pfam02324	Glyco_hydro_70,	2,6	1,9	0,0	1,5

Contig_486001	pfam01341	Glyco_hydro_6,	0,0	4,5	0,0	1,5
Contig_43316	pfam00728	Glyco_hydro_20,	0,9	3,6	0,0	1,5
Contig_138824	pfam11790	Glyco_hydro_cc,	0,0	0,0	4,5	1,5
Contig_324281	pfam03198	Glyco_hydro_72,	1,6	2,9	0,0	1,5
Contig_367213	pfam03639	Glyco_hydro_81,	1,6	2,9	0,0	1,5
Contig_502116	pfam07470	Glyco_hydro_88,	1,6	2,9	0,0	1,5
Contig_512955	pfam07477	Glyco_hydro_67C,	3,7	0,8	0,0	1,5
Contig_85849	pfam04616	Glyco_hydro_43,	4,5	0,0	0,0	1,5
Contig_442282	pfam02057	Glyco_hydro_59,	2,8	1,7	0,0	1,5
Contig_566554	pfam03200	Glyco_hydro_63,	4,5	0,0	0,0	1,5
Contig_347940	pfam08532	Glyco_hydro_42M,	2,5	2,0	0,0	1,5
Contig_162017	pfam07971	Glyco_hydro_92,	1,0	3,5	0,0	1,5
Contig_276239	pfam00295	Glyco_hydro_28,	3,7	0,7	0,0	1,5
Contig_368011	pfam03639	Glyco_hydro_81,	2,2	2,2	0,0	1,5
Contig_373634	pfam01055	Glyco_hydro_31,	2,0	2,4	0,0	1,5
Contig_433675	pfam08244	Glyco_hydro_32C,	2,3	2,1	0,0	1,5
Contig_56774	pfam01301	Glyco_hydro_35,	2,9	0,3	1,2	1,5
Contig_457288	pfam03639	Glyco_hydro_81,	1,5	2,9	0,0	1,5
Contig_293868	pfam02837	Glyco_hydro_2_N,	3,1	1,3	0,0	1,5
Contig_458256	pfam00704	Glyco_hydro_18,	1,0	3,5	0,0	1,5
Contig_462351	pfam03065	Glyco_hydro_57,	3,7	0,7	0,0	1,5
Contig_434762	pfam00723	Glyco_hydro_15,	2,6	1,8	0,0	1,5
Contig_110151	pfam03718	Glyco_hydro_49,	1,0	3,4	0,0	1,5
Contig_293825	pfam00332	Glyco_hydro_17,	0,0	4,4	0,0	1,5
Contig_448971	pfam00232	Glyco_hydro_1,	0,0	4,4	0,0	1,5
Contig_528178	pfam00704	Glyco_hydro_18,	4,4	0,0	0,0	1,5
Contig_158463	pfam03512	Glyco_hydro_52,	1,6	2,8	0,0	1,5
Contig_167793	pfam03644	Glyco_hydro_85,	1,1	3,3	0,0	1,5
Contig_363499	pfam03662	Glyco_hydro_79n,	1,7	2,6	0,0	1,5
Contig_369554	pfam03632	Glyco_hydro_65m,	2,7	1,6	0,0	1,5
Contig_245781	pfam12899	Glyco_hydro_100,	0,0	4,4	0,0	1,5
Contig_452617	pfam04616	Glyco_hydro_43,	2,7	1,6	0,0	1,5
Contig_192069	pfam03200	Glyco_hydro_63,	0,0	4,4	0,0	1,5
Contig_206048	pfam02446	Glyco_hydro_77,	0,0	4,4	0,0	1,5
Contig_272925	pfam01532	Glyco_hydro_47,	1,6	2,8	0,0	1,5
Contig_277437	pfam10566	Glyco_hydro_97,	3,6	0,7	0,0	1,5
Contig_331670	pfam00331	Glyco_hydro_10,	0,0	4,4	0,0	1,5
Contig_401913	pfam00332	Glyco_hydro_17,	0,0	4,4	0,0	1,5
Contig_168976	pfam00704	Glyco_hydro_18,	1,7	2,6	0,0	1,4
Contig_358651	pfam01532	Glyco_hydro_47,	0,0	4,3	0,0	1,4
Contig_419940	pfam00728	Glyco_hydro_20,	1,1	3,3	0,0	1,4
Contig_551473	pfam02324	Glyco_hydro_70,	3,3	1,0	0,0	1,4
Contig_573601	pfam01055	Glyco_hydro_31,	4,3	0,0	0,0	1,4
Contig_274757	pfam03636	Glyco_hydro_65N,	1,7	2,7	0,0	1,4
Contig_306884	pfam03200	Glyco_hydro_63,	0,5	3,2	0,7	1,4
Contig_380100	pfam11790	Glyco_hydro_cc,	2,2	1,1	1,1	1,4
Contig_212130	pfam00933	Glyco_hydro_3,	2,6	1,7	0,0	1,4
Contig_328744	pfam07477	Glyco_hydro_67C,	0,0	4,3	0,0	1,4
Contig_414433	pfam00759	Glyco_hydro_9,	0,0	4,3	0,0	1,4
Contig_462501	pfam07971	Glyco_hydro_92,	4,3	0,0	0,0	1,4
Contig_397487	pfam00728	Glyco_hydro_20,	2,3	2,0	0,0	1,4
Contig_429308	pfam03200	Glyco_hydro_63,	0,0	4,3	0,0	1,4
Contig_554938	pfam00728	Glyco_hydro_20,	3,1	1,2	0,0	1,4
Contig_171929	pfam02324	Glyco_hydro_70,	0,0	4,3	0,0	1,4
Contig_227432	pfam07971	Glyco_hydro_92,	2,7	1,6	0,0	1,4
Contig_238121	pfam10566	Glyco_hydro_97,	4,3	0,0	0,0	1,4
Contig_262121	pfam00251	Glyco_hydro_32N,	4,3	0,0	0,0	1,4
Contig_489271	pfam10566	Glyco_hydro_97,	4,3	0,0	0,0	1,4
Contig_568433	pfam02324	Glyco_hydro_70,	3,3	1,0	0,0	1,4
Contig_463083	pfam00182	Glyco_hydro_19,	1,5	2,8	0,0	1,4
Contig_515088	pfam12905	Glyco_hydro_101,	0,9	3,3	0,0	1,4
Contig_75099	pfam00232	Glyco_hydro_1,	3,7	0,6	0,0	1,4
Contig_147057	pfam00704	Glyco_hydro_18,	4,3	0,0	0,0	1,4
Contig_70230	pfam07748	Glyco_hydro_38C,	0,5	3,7	0,0	1,4
Contig_157508	pfam07748	Glyco_hydro_38C,	1,0	3,2	0,0	1,4
Contig_290663	pfam00728	Glyco_hydro_20,	2,8	1,5	0,0	1,4
Contig_493362	pfam03639	Glyco_hydro_81,	4,2	0,0	0,0	1,4
Contig_353488	pfam00251	Glyco_hydro_32N,	4,2	0,0	0,0	1,4
Contig_515440	pfam11975	Glyco_hydro_4C,	0,0	4,2	0,0	1,4
Contig_518983	pfam01532	Glyco_hydro_47,	2,6	1,6	0,0	1,4
Contig_49401	pfam01055	Glyco_hydro_31,	1,5	2,7	0,0	1,4

Contig_414382	pfam02837	Glyco_hydro_2_N,	0,0	4,2	0,0	1,4
Contig_422644	pfam00728	Glyco_hydro_20,	0,0	4,2	0,0	1,4
Contig_481464	pfam03200	Glyco_hydro_63,	0,0	4,2	0,0	1,4
Contig_549619	pfam02055	Glyco_hydro_30,	4,2	0,0	0,0	1,4
Contig_172318	pfam01915	Glyco_hydro_3_C,	0,0	4,2	0,0	1,4
Contig_256087	pfam12899	Glyco_hydro_100,	0,0	4,2	0,0	1,4
Contig_573233	pfam00251	Glyco_hydro_32N,	4,2	0,0	0,0	1,4
Contig_32399	pfam07971	Glyco_hydro_92,	1,7	2,5	0,0	1,4
Contig_164699	pfam00331	Glyco_hydro_10,	0,0	4,2	0,0	1,4
Contig_266919	pfam01055	Glyco_hydro_31,	3,7	0,5	0,0	1,4
Contig_538160	pfam12899	Glyco_hydro_100,	4,2	0,0	0,0	1,4
Contig_96462	pfam00182	Glyco_hydro_19,	3,2	1,0	0,0	1,4
Contig_237074	pfam01532	Glyco_hydro_47,	0,0	4,2	0,0	1,4
Contig_378476	pfam02837	Glyco_hydro_2_N,	1,9	2,3	0,0	1,4
Contig_574183	pfam01532	Glyco_hydro_47,	4,2	0,0	0,0	1,4
Contig_253259	pfam11790	Glyco_hydro_cc,	0,0	4,2	0,0	1,4
Contig_256887	pfam01915	Glyco_hydro_3_C,	2,2	2,0	0,0	1,4
Contig_321258	pfam00723	Glyco_hydro_15,	4,2	0,0	0,0	1,4
Contig_121970	pfam00331	Glyco_hydro_10,	0,7	3,5	0,0	1,4
Contig_298345	pfam00728	Glyco_hydro_20,	3,0	1,1	0,0	1,4
Contig_418821	pfam02156	Glyco_hydro_26,	3,6	0,5	0,0	1,4
Contig_349308	pfam03659	Glyco_hydro_71,	0,0	4,1	0,0	1,4
Contig_450379	pfam03639	Glyco_hydro_81,	4,1	0,0	0,0	1,4
Contig_79105	pfam00331	Glyco_hydro_10,	1,2	2,9	0,0	1,4
Contig_233756	pfam03644	Glyco_hydro_85,	2,8	1,3	0,0	1,4
Contig_411600	pfam01630	Glyco_hydro_56,	4,1	0,0	0,0	1,4
Contig_516372	pfam10566	Glyco_hydro_97,	1,9	2,3	0,0	1,4
Contig_565604	pfam12905	Glyco_hydro_101,	4,1	0,0	0,0	1,4
Contig_503971	pfam00295	Glyco_hydro_28,	2,2	2,0	0,0	1,4
Contig_518721	pfam01630	Glyco_hydro_56,	2,2	2,0	0,0	1,4
Contig_330504	pfam07477	Glyco_hydro_67C,	0,7	3,4	0,0	1,4
Contig_496623	pfam01074	Glyco_hydro_38,	3,5	0,6	0,0	1,4
Contig_125989	pfam04616	Glyco_hydro_43,	2,2	1,9	0,0	1,4
Contig_173776	pfam01055	Glyco_hydro_31,	0,0	4,1	0,0	1,4
Contig_181083	pfam00332	Glyco_hydro_17,	0,0	4,1	0,0	1,4
Contig_296200	pfam00332	Glyco_hydro_17,	0,0	4,1	0,0	1,4
Contig_371353	pfam03644	Glyco_hydro_85,	0,0	4,1	0,0	1,4
Contig_285817	pfam03512	Glyco_hydro_52,	0,0	4,1	0,0	1,4
Contig_329809	pfam00759	Glyco_hydro_9,	2,6	1,5	0,0	1,4
Contig_480175	pfam02011	Glyco_hydro_48,	2,6	1,5	0,0	1,4
Contig_483313	pfam00251	Glyco_hydro_32N,	1,2	2,9	0,0	1,4
Contig_496777	pfam01532	Glyco_hydro_47,	3,8	0,3	0,0	1,4
Contig_575209	pfam03512	Glyco_hydro_52,	0,0	4,1	0,0	1,4
Contig_428989	pfam03639	Glyco_hydro_81,	4,1	0,0	0,0	1,4
Contig_446871	pfam01301	Glyco_hydro_35,	0,0	4,1	0,0	1,4
Contig_488890	pfam07748	Glyco_hydro_38C,	4,1	0,0	0,0	1,4
Contig_505023	pfam00251	Glyco_hydro_32N,	0,0	4,1	0,0	1,4
Contig_143990	pfam00332	Glyco_hydro_17,	0,8	3,2	0,0	1,4
Contig_225339	pfam02836	Glyco_hydro_2_C,	1,6	2,4	0,0	1,4
Contig_160139	pfam00182	Glyco_hydro_19,	3,5	0,5	0,0	1,3
Contig_400689	pfam00840	Glyco_hydro_7,	4,0	0,0	0,0	1,3
Contig_566687	pfam01630	Glyco_hydro_56,	4,0	0,0	0,0	1,3
Contig_159570	pfam02324	Glyco_hydro_70,	0,0	4,0	0,0	1,3
Contig_86476	pfam03662	Glyco_hydro_79n,	1,4	2,6	0,0	1,3
Contig_299050	pfam00295	Glyco_hydro_28,	0,0	4,0	0,0	1,3
Contig_353021	pfam03639	Glyco_hydro_81,	0,0	4,0	0,0	1,3
Contig_358845	pfam12905	Glyco_hydro_101,	0,0	4,0	0,0	1,3
Contig_459937	pfam12905	Glyco_hydro_101,	0,0	4,0	0,0	1,3
Contig_74678	pfam07971	Glyco_hydro_92,	0,0	4,0	0,0	1,3
Contig_418603	pfam02836	Glyco_hydro_2_C,	2,9	1,2	0,0	1,3
Contig_461063	pfam00722	Glyco_hydro_16,	0,8	3,2	0,0	1,3
Contig_303975	pfam13199	Glyco_hydro_66,	3,1	0,9	0,0	1,3
Contig_488507	pfam00295	Glyco_hydro_28,	4,0	0,0	0,0	1,3
Contig_572527	pfam08244	Glyco_hydro_32C,	4,0	0,0	0,0	1,3
Contig_413531	pfam01915	Glyco_hydro_3_C,	0,0	4,0	0,0	1,3
Contig_171119	pfam03512	Glyco_hydro_52,	0,0	4,0	0,0	1,3
Contig_181527	pfam00251	Glyco_hydro_32N,	0,0	4,0	0,0	1,3
Contig_219503	pfam00704	Glyco_hydro_18,	0,0	4,0	0,0	1,3
Contig_458801	pfam07745	Glyco_hydro_53,	2,5	1,5	0,0	1,3
Contig_316858	pfam03200	Glyco_hydro_63,	0,0	4,0	0,0	1,3
Contig_145808	pfam04616	Glyco_hydro_43,	0,0	2,5	1,5	1,3

Contig_164098	pfam03198	Glyco_hydro_72,	2,0	2,0	0,0	1,3
Contig_459132	pfam01630	Glyco_hydro_56,	3,5	0,5	0,0	1,3
Contig_461364	pfam03200	Glyco_hydro_63,	2,5	1,5	0,0	1,3
Contig_461850	pfam07748	Glyco_hydro_38C,	1,8	2,2	0,0	1,3
Contig_280720	pfam07477	Glyco_hydro_67C,	3,0	0,9	0,0	1,3
Contig_535447	pfam03664	Glyco_hydro_62,	4,0	0,0	0,0	1,3
Contig_293721	pfam03200	Glyco_hydro_63,	0,4	3,5	0,0	1,3
Contig_499041	pfam00251	Glyco_hydro_32N,	1,8	2,2	0,0	1,3
Contig_256452	pfam00331	Glyco_hydro_10,	0,0	3,9	0,0	1,3
Contig_487535	pfam00759	Glyco_hydro_9,	0,0	3,9	0,0	1,3
Contig_30470	pfam01055	Glyco_hydro_31,	0,0	2,9	1,1	1,3
Contig_344659	pfam03659	Glyco_hydro_71,	2,0	2,0	0,0	1,3
Contig_168161	pfam03198	Glyco_hydro_72,	2,0	2,0	0,0	1,3
Contig_415172	pfam02435	Glyco_hydro_68,	0,0	3,9	0,0	1,3
Contig_70692	pfam00704	Glyco_hydro_18,	1,8	2,1	0,0	1,3
Contig_487580	pfam07748	Glyco_hydro_38C,	2,1	1,9	0,0	1,3
Contig_455972	pfam03718	Glyco_hydro_49,	3,9	0,0	0,0	1,3
Contig_81699	pfam00933	Glyco_hydro_3,	0,0	0,8	3,1	1,3
Contig_105722	pfam01055	Glyco_hydro_31,	0,0	3,9	0,0	1,3
Contig_381551	pfam00704	Glyco_hydro_18,	0,0	3,9	0,0	1,3
Contig_511007	pfam03718	Glyco_hydro_49,	2,4	1,5	0,0	1,3
Contig_94886	pfam03443	Glyco_hydro_61,	0,0	1,3	2,6	1,3
Contig_373217	pfam02836	Glyco_hydro_2_C,	2,0	1,8	0,0	1,3
Contig_459674	pfam00704	Glyco_hydro_18,	1,1	2,7	0,0	1,3
Contig_466165	pfam00722	Glyco_hydro_16,	1,0	2,9	0,0	1,3
Contig_413236	pfam00332	Glyco_hydro_17,	1,4	2,5	0,0	1,3
Contig_438335	pfam00840	Glyco_hydro_7,	3,9	0,0	0,0	1,3
Contig_170008	pfam00722	Glyco_hydro_16,	0,0	3,8	0,0	1,3
Contig_361969	pfam10566	Glyco_hydro_97,	0,0	3,8	0,0	1,3
Contig_479069	pfam00933	Glyco_hydro_3,	0,0	3,8	0,0	1,3
Contig_495057	pfam00759	Glyco_hydro_9,	3,8	0,0	0,0	1,3
Contig_260683	pfam00232	Glyco_hydro_1,	1,4	2,5	0,0	1,3
Contig_277527	pfam02156	Glyco_hydro_26,	0,8	3,0	0,0	1,3
Contig_101745	pfam03198	Glyco_hydro_72,	3,8	0,0	0,0	1,3
Contig_196631	pfam02015	Glyco_hydro_45,	1,7	2,1	0,0	1,3
Contig_258320	pfam00251	Glyco_hydro_32N,	0,0	3,8	0,0	1,3
Contig_550285	pfam03200	Glyco_hydro_63,	3,8	0,0	0,0	1,3
Contig_195175	pfam03644	Glyco_hydro_85,	1,0	2,9	0,0	1,3
Contig_318906	pfam07971	Glyco_hydro_92,	0,8	3,0	0,0	1,3
Contig_235063	pfam02011	Glyco_hydro_48,	2,4	1,4	0,0	1,3
Contig_172297	pfam02055	Glyco_hydro_30,	0,8	3,0	0,0	1,3
Contig_279720	pfam12905	Glyco_hydro_101,	0,0	3,8	0,0	1,3
Contig_499613	pfam12899	Glyco_hydro_100,	0,0	3,8	0,0	1,3
Contig_31858	pfam03648	Glyco_hydro_67N,	1,1	2,7	0,0	1,3
Contig_363688	pfam01055	Glyco_hydro_31,	1,1	2,7	0,0	1,3
Contig_228626	pfam08244	Glyco_hydro_32C,	0,7	3,1	0,0	1,3
Contig_317474	pfam00331	Glyco_hydro_10,	0,0	3,8	0,0	1,3
Contig_132096	pfam03663	Glyco_hydro_76,	3,8	0,0	0,0	1,3
Contig_336766	pfam00704	Glyco_hydro_18,	2,3	1,4	0,0	1,3
Contig_395410	pfam04616	Glyco_hydro_43,	3,8	0,0	0,0	1,3
Contig_194189	pfam02836	Glyco_hydro_2_C,	1,1	2,6	0,0	1,2
Contig_318728	pfam00723	Glyco_hydro_15,	1,3	2,4	0,0	1,2
Contig_513470	pfam01301	Glyco_hydro_35,	0,0	3,7	0,0	1,2
Contig_484617	pfam00704	Glyco_hydro_18,	3,0	0,7	0,0	1,2
Contig_84659	pfam00722	Glyco_hydro_16,	3,7	0,0	0,0	1,2
Contig_281265	pfam00840	Glyco_hydro_7,	0,0	3,7	0,0	1,2
Contig_489087	pfam07745	Glyco_hydro_53,	2,3	1,4	0,0	1,2
Contig_538756	pfam01670	Glyco_hydro_12,	2,9	0,9	0,0	1,2
Contig_452034	pfam01074	Glyco_hydro_38,	0,0	3,7	0,0	1,2
Contig_87240	pfam02837	Glyco_hydro_2_N,	0,5	2,1	1,1	1,2
Contig_416129	pfam00728	Glyco_hydro_20,	3,1	0,6	0,0	1,2
Contig_256620	pfam02446	Glyco_hydro_77,	0,0	3,7	0,0	1,2
Contig_359872	pfam01532	Glyco_hydro_47,	1,3	2,4	0,0	1,2
Contig_488851	pfam01341	Glyco_hydro_6,	3,7	0,0	0,0	1,2
Contig_489622	pfam01301	Glyco_hydro_35,	0,0	3,7	0,0	1,2
Contig_422185	pfam00331	Glyco_hydro_10,	1,7	2,0	0,0	1,2
Contig_11271	pfam02449	Glyco_hydro_42,	0,0	3,7	0,0	1,2
Contig_84258	pfam01270	Glyco_hydro_8,	1,7	2,0	0,0	1,2
Contig_191830	pfam07470	Glyco_hydro_88,	0,4	3,3	0,0	1,2
Contig_341936	pfam03200	Glyco_hydro_63,	3,7	0,0	0,0	1,2
Contig_398606	pfam03718	Glyco_hydro_49,	1,3	2,4	0,0	1,2

Contig_448622	pfam03512	Glyco_hydro_52,	0,0	3,7	0,0	1,2
Contig_52829	pfam00728	Glyco_hydro_20,	1,6	0,0	2,0	1,2
Contig_54303	pfam01630	Glyco_hydro_56,	0,0	3,6	0,0	1,2
Contig_121596	pfam08532	Glyco_hydro_42M,	1,6	2,0	0,0	1,2
Contig_312339	pfam02449	Glyco_hydro_42,	0,0	3,6	0,0	1,2
Contig_329055	pfam03639	Glyco_hydro_81,	0,0	3,6	0,0	1,2
Contig_381238	pfam03065	Glyco_hydro_57,	0,0	3,6	0,0	1,2
Contig_422397	pfam02446	Glyco_hydro_77,	0,0	3,6	0,0	1,2
Contig_458357	pfam00704	Glyco_hydro_18,	0,0	3,6	0,0	1,2
Contig_416790	pfam00232	Glyco_hydro_1,	0,8	2,8	0,0	1,2
Contig_424091	pfam01055	Glyco_hydro_31,	2,0	1,6	0,0	1,2
Contig_456075	pfam03644	Glyco_hydro_85,	0,0	3,6	0,0	1,2
Contig_483068	pfam02836	Glyco_hydro_2_C,	1,1	2,6	0,0	1,2
Contig_95698	pfam00182	Glyco_hydro_19,	0,0	3,6	0,0	1,2
Contig_573128	pfam00232	Glyco_hydro_1,	3,6	0,0	0,0	1,2
Contig_131469	pfam04616	Glyco_hydro_43,	2,2	1,4	0,0	1,2
Contig_360629	pfam00232	Glyco_hydro_1,	2,6	1,0	0,0	1,2
Contig_164054	pfam01055	Glyco_hydro_31,	1,3	2,3	0,0	1,2
Contig_428644	pfam13199	Glyco_hydro_66,	0,0	3,6	0,0	1,2
Contig_476829	pfam02011	Glyco_hydro_48,	1,3	2,3	0,0	1,2
Contig_419061	pfam02837	Glyco_hydro_2_N,	1,9	1,7	0,0	1,2
Contig_485768	pfam01055	Glyco_hydro_31,	0,0	3,6	0,0	1,2
Contig_179363	pfam00840	Glyco_hydro_7,	2,7	0,8	0,0	1,2
Contig_298501	pfam07470	Glyco_hydro_88,	0,0	3,5	0,0	1,2
Contig_381574	pfam00295	Glyco_hydro_28,	0,0	3,5	0,0	1,2
Contig_303321	pfam12899	Glyco_hydro_100,	0,9	2,6	0,0	1,2
Contig_435284	pfam00722	Glyco_hydro_16,	1,9	1,7	0,0	1,2
Contig_135911	pfam13199	Glyco_hydro_66,	1,6	1,9	0,0	1,2
Contig_339053	pfam01055	Glyco_hydro_31,	2,9	0,6	0,0	1,2
Contig_412408	pfam00728	Glyco_hydro_20,	3,1	0,5	0,0	1,2
Contig_456322	pfam00728	Glyco_hydro_20,	0,5	3,0	0,0	1,2
Contig_508455	pfam02446	Glyco_hydro_77,	0,0	3,5	0,0	1,2
Contig_109608	pfam01301	Glyco_hydro_35,	3,5	0,0	0,0	1,2
Contig_462540	pfam00723	Glyco_hydro_15,	2,2	1,3	0,0	1,2
Contig_155038	pfam13199	Glyco_hydro_66,	2,4	1,1	0,0	1,2
Contig_211799	pfam01229	Glyco_hydro_39,	0,0	3,5	0,0	1,2
Contig_227124	pfam01532	Glyco_hydro_47,	0,0	3,5	0,0	1,2
Contig_313502	pfam03659	Glyco_hydro_71,	0,0	3,5	0,0	1,2
Contig_573264	pfam07745	Glyco_hydro_53,	3,5	0,0	0,0	1,2
Contig_191399	pfam02449	Glyco_hydro_42,	0,0	3,5	0,0	1,2
Contig_321633	pfam00704	Glyco_hydro_18,	0,0	3,5	0,0	1,2
Contig_341501	pfam03198	Glyco_hydro_72,	0,0	3,5	0,0	1,2
Contig_564502	pfam00704	Glyco_hydro_18,	2,9	0,6	0,0	1,2
Contig_147123	pfam01074	Glyco_hydro_38,	0,0	3,5	0,0	1,2
Contig_162757	pfam10566	Glyco_hydro_97,	0,0	3,5	0,0	1,2
Contig_228366	pfam02156	Glyco_hydro_26,	0,0	3,5	0,0	1,2
Contig_292036	pfam10566	Glyco_hydro_97,	1,8	1,6	0,0	1,2
Contig_412468	pfam00232	Glyco_hydro_1,	0,0	3,5	0,0	1,2
Contig_249558	pfam02055	Glyco_hydro_30,	0,0	3,4	0,0	1,1
Contig_73392	pfam02324	Glyco_hydro_70,	2,5	1,0	0,0	1,1
Contig_495326	pfam02836	Glyco_hydro_2_C,	2,1	1,3	0,0	1,1
Contig_172731	pfam00722	Glyco_hydro_16,	0,0	3,4	0,0	1,1
Contig_365290	pfam01055	Glyco_hydro_31,	1,9	1,5	0,0	1,1
Contig_397244	pfam00251	Glyco_hydro_32N,	1,9	1,5	0,0	1,1
Contig_107241	pfam02836	Glyco_hydro_2_C,	0,0	0,0	3,4	1,1
Contig_161039	pfam01074	Glyco_hydro_38,	0,0	3,4	0,0	1,1
Contig_215466	pfam07477	Glyco_hydro_67C,	0,0	3,4	0,0	1,1
Contig_250278	pfam13199	Glyco_hydro_66,	0,9	2,6	0,0	1,1
Contig_256941	pfam00232	Glyco_hydro_1,	2,1	1,3	0,0	1,1
Contig_264889	pfam00332	Glyco_hydro_17,	0,0	3,4	0,0	1,1
Contig_512849	pfam04616	Glyco_hydro_43,	2,1	1,3	0,0	1,1
Contig_253890	pfam04616	Glyco_hydro_43,	2,1	1,3	0,0	1,1
Contig_389501	pfam01532	Glyco_hydro_47,	1,5	1,9	0,0	1,1
Contig_454209	pfam07971	Glyco_hydro_92,	3,4	0,0	0,0	1,1
Contig_300269	pfam00723	Glyco_hydro_15,	0,0	3,4	0,0	1,1
Contig_354981	pfam02011	Glyco_hydro_48,	0,0	3,4	0,0	1,1
Contig_371763	pfam01055	Glyco_hydro_31,	2,9	0,5	0,0	1,1
Contig_463450	pfam01074	Glyco_hydro_38,	0,0	3,4	0,0	1,1
Contig_472973	pfam11790	Glyco_hydro_cc,	0,0	3,4	0,0	1,1
Contig_506998	pfam01301	Glyco_hydro_35,	0,0	3,4	0,0	1,1
Contig_295826	pfam07477	Glyco_hydro_67C,	2,4	1,0	0,0	1,1

Contig_385864	pfam11790	Glyco_hydro_cc,	1,4	2,0	0,0	1,1
Contig_411420	pfam00759	Glyco_hydro_9,	0,0	0,0	3,4	1,1
Contig_328663	pfam00295	Glyco_hydro_28,	2,1	1,3	0,0	1,1
Contig_405881	pfam03200	Glyco_hydro_63,	0,0	3,4	0,0	1,1
Contig_195840	pfam07470	Glyco_hydro_88,	0,0	3,4	0,0	1,1
Contig_360314	pfam01074	Glyco_hydro_38,	0,0	3,4	0,0	1,1
Contig_187	pfam03198	Glyco_hydro_72,	1,5	0,0	1,9	1,1
Contig_170880	pfam03639	Glyco_hydro_81,	3,3	0,0	0,0	1,1
Contig_433284	pfam03664	Glyco_hydro_62,	0,0	3,3	0,0	1,1
Contig_462354	pfam10566	Glyco_hydro_97,	1,2	2,2	0,0	1,1
Contig_439178	pfam00722	Glyco_hydro_16,	0,0	3,3	0,0	1,1
Contig_82456	pfam03718	Glyco_hydro_49,	3,3	0,0	0,0	1,1
Contig_322894	pfam03662	Glyco_hydro_79n,	1,2	2,1	0,0	1,1
Contig_343163	pfam00933	Glyco_hydro_3,	0,0	3,3	0,0	1,1
Contig_465597	pfam00232	Glyco_hydro_1,	0,0	3,3	0,0	1,1
Contig_437040	pfam01270	Glyco_hydro_8,	0,6	2,7	0,0	1,1
Contig_494200	pfam01532	Glyco_hydro_47,	0,0	3,3	0,0	1,1
Contig_163450	pfam03659	Glyco_hydro_71,	2,5	0,8	0,0	1,1
Contig_399254	pfam00933	Glyco_hydro_3,	1,5	1,8	0,0	1,1
Contig_502932	pfam07477	Glyco_hydro_67C,	0,0	0,6	2,7	1,1
Contig_207829	pfam02449	Glyco_hydro_42,	0,0	3,3	0,0	1,1
Contig_395286	pfam00840	Glyco_hydro_7,	0,0	3,3	0,0	1,1
Contig_132243	pfam00704	Glyco_hydro_18,	2,8	0,5	0,0	1,1
Contig_552205	pfam04616	Glyco_hydro_43,	3,3	0,0	0,0	1,1
Contig_503778	pfam00295	Glyco_hydro_28,	0,0	3,3	0,0	1,1
Contig_513856	pfam00723	Glyco_hydro_15,	0,8	2,4	0,0	1,1
Contig_564814	pfam03200	Glyco_hydro_63,	3,3	0,0	0,0	1,1
Contig_515390	pfam00728	Glyco_hydro_20,	3,2	0,0	0,0	1,1
Contig_38055	pfam00728	Glyco_hydro_20,	0,0	3,2	0,0	1,1
Contig_255789	pfam01055	Glyco_hydro_31,	1,7	1,5	0,0	1,1
Contig_273148	pfam07488	Glyco_hydro_67M,	0,9	2,3	0,0	1,1
Contig_162384	pfam07971	Glyco_hydro_92,	0,8	2,4	0,0	1,1
Contig_371436	pfam00331	Glyco_hydro_10,	0,0	3,2	0,0	1,1
Contig_438462	pfam12899	Glyco_hydro_100,	3,2	0,0	0,0	1,1
Contig_498728	pfam00704	Glyco_hydro_18,	2,3	0,9	0,0	1,1
Contig_248311	pfam01301	Glyco_hydro_35,	0,9	2,3	0,0	1,1
Contig_159948	pfam07477	Glyco_hydro_67C,	0,9	2,2	0,0	1,1
Contig_259482	pfam07477	Glyco_hydro_67C,	0,9	2,2	0,0	1,1
Contig_346127	pfam03644	Glyco_hydro_85,	0,9	2,2	0,0	1,1
Contig_19539	pfam02837	Glyco_hydro_2_N,	0,5	2,7	0,0	1,1
Contig_418724	pfam01055	Glyco_hydro_31,	2,4	0,7	0,0	1,1
Contig_370262	pfam12899	Glyco_hydro_100,	0,0	3,2	0,0	1,1
Contig_156544	pfam02055	Glyco_hydro_30,	1,1	2,0	0,0	1,0
Contig_499978	pfam01229	Glyco_hydro_39,	3,1	0,0	0,0	1,0
Contig_93755	pfam04616	Glyco_hydro_43,	0,0	3,1	0,0	1,0
Contig_52007	pfam00704	Glyco_hydro_18,	1,4	1,7	0,0	1,0
Contig_573310	pfam07748	Glyco_hydro_38C,	3,1	0,0	0,0	1,0
Contig_42141	pfam00232	Glyco_hydro_1,	0,0	3,1	0,0	1,0
Contig_277899	pfam03200	Glyco_hydro_63,	0,0	3,1	0,0	1,0
Contig_167910	pfam00759	Glyco_hydro_9,	1,4	1,7	0,0	1,0
Contig_383123	pfam03632	Glyco_hydro_65m,	1,6	1,5	0,0	1,0
Contig_417308	pfam00759	Glyco_hydro_9,	0,0	3,1	0,0	1,0
Contig_428378	pfam01915	Glyco_hydro_3_C,	0,0	3,1	0,0	1,0
Contig_516024	pfam00457	Glyco_hydro_11,	2,4	0,7	0,0	1,0
Contig_209089	pfam03663	Glyco_hydro_76,	0,0	3,1	0,0	1,0
Contig_219104	pfam07971	Glyco_hydro_92,	3,1	0,0	0,0	1,0
Contig_378822	pfam00704	Glyco_hydro_18,	0,0	3,1	0,0	1,0
Contig_462899	pfam02055	Glyco_hydro_30,	2,4	0,7	0,0	1,0
Contig_167056	pfam01055	Glyco_hydro_31,	1,4	0,6	1,1	1,0
Contig_262170	pfam07748	Glyco_hydro_38C,	1,9	1,1	0,0	1,0
Contig_428753	pfam03443	Glyco_hydro_61,	0,0	3,0	0,0	1,0
Contig_452734	pfam07477	Glyco_hydro_67C,	3,0	0,0	0,0	1,0
Contig_341425	pfam02055	Glyco_hydro_30,	1,1	2,0	0,0	1,0
Contig_461133	pfam07971	Glyco_hydro_92,	3,0	0,0	0,0	1,0
Contig_45581	pfam01301	Glyco_hydro_35,	0,6	2,4	0,0	1,0
Contig_510157	pfam03639	Glyco_hydro_81,	0,0	3,0	0,0	1,0
Contig_518447	pfam03659	Glyco_hydro_71,	0,0	3,0	0,0	1,0
Contig_20948	pfam03633	Glyco_hydro_65C,	0,0	3,0	0,0	1,0
Contig_559707	pfam03664	Glyco_hydro_62,	3,0	0,0	0,0	1,0
Contig_244360	pfam01915	Glyco_hydro_3_C,	2,3	0,7	0,0	1,0
Contig_451044	pfam00703	Glyco_hydro_2,	0,0	3,0	0,0	1,0

Contig_235723	pfam01301	Glyco_hydro_35,	0,0	3,0	0,0	1,0
Contig_474407	pfam01670	Glyco_hydro_12,	0,0	3,0	0,0	1,0
Contig_495503	pfam13647	Glyco_hydro_80,	1,6	1,4	0,0	1,0
Contig_186385	pfam13199	Glyco_hydro_66,	2,3	0,7	0,0	1,0
Contig_400440	pfam00704	Glyco_hydro_18,	0,9	2,1	0,0	1,0
Contig_124287	pfam07745	Glyco_hydro_53,	3,0	0,0	0,0	1,0
Contig_251143	pfam00728	Glyco_hydro_20,	0,0	3,0	0,0	1,0
Contig_318792	pfam00703	Glyco_hydro_2,	1,1	1,9	0,0	1,0
Contig_416647	pfam01055	Glyco_hydro_31,	0,0	3,0	0,0	1,0
Contig_444102	pfam03644	Glyco_hydro_85,	1,4	1,6	0,0	1,0
Contig_153721	pfam02449	Glyco_hydro_42,	2,3	0,7	0,0	1,0
Contig_486175	pfam01270	Glyco_hydro_8,	0,0	3,0	0,0	1,0
Contig_391353	pfam07971	Glyco_hydro_92,	2,2	0,8	0,0	1,0
Contig_110081	pfam02446	Glyco_hydro_77,	0,0	2,9	0,0	1,0
Contig_358001	pfam00759	Glyco_hydro_9,	0,0	2,9	0,0	1,0
Contig_416084	pfam13199	Glyco_hydro_66,	0,0	2,9	0,0	1,0
Contig_420662	pfam01532	Glyco_hydro_47,	0,0	2,9	0,0	1,0
Contig_456045	pfam07488	Glyco_hydro_67M,	1,1	1,9	0,0	1,0
Contig_515356	pfam01055	Glyco_hydro_31,	0,0	2,9	0,0	1,0
Contig_362207	pfam02055	Glyco_hydro_30,	0,0	2,9	0,0	1,0
Contig_395185	pfam00251	Glyco_hydro_32N,	2,9	0,0	0,0	1,0
Contig_413952	pfam03065	Glyco_hydro_57,	0,0	2,9	0,0	1,0
Contig_339415	pfam00933	Glyco_hydro_3,	0,0	2,9	0,0	1,0
Contig_45230	pfam07971	Glyco_hydro_92,	0,0	2,9	0,0	1,0
Contig_257881	pfam03198	Glyco_hydro_72,	1,0	1,9	0,0	1,0
Contig_372686	pfam01670	Glyco_hydro_12,	0,0	2,9	0,0	1,0
Contig_431730	pfam07470	Glyco_hydro_88,	1,2	1,7	0,0	1,0
Contig_251924	pfam07470	Glyco_hydro_88,	1,2	1,7	0,0	1,0
Contig_437667	pfam00295	Glyco_hydro_28,	0,0	2,9	0,0	1,0
Contig_284244	pfam01055	Glyco_hydro_31,	1,3	1,6	0,0	1,0
Contig_468948	pfam02449	Glyco_hydro_42,	0,6	2,3	0,0	1,0
Contig_11156	pfam02836	Glyco_hydro_2_C,	1,1	1,7	0,0	1,0
Contig_85371	pfam02449	Glyco_hydro_42,	0,0	2,8	0,0	0,9
Contig_307970	pfam00728	Glyco_hydro_20,	0,0	2,8	0,0	0,9
Contig_371388	pfam01532	Glyco_hydro_47,	2,8	0,0	0,0	0,9
Contig_95039	pfam02055	Glyco_hydro_30,	1,3	1,6	0,0	0,9
Contig_132916	pfam01532	Glyco_hydro_47,	2,2	0,7	0,0	0,9
Contig_93872	pfam02836	Glyco_hydro_2_C,	0,8	2,0	0,0	0,9
Contig_237692	pfam02449	Glyco_hydro_42,	1,0	1,8	0,0	0,9
Contig_272716	pfam02837	Glyco_hydro_2_N,	0,0	2,8	0,0	0,9
Contig_362927	pfam01373	Glyco_hydro_14,	1,6	1,3	0,0	0,9
Contig_418011	pfam07488	Glyco_hydro_67M,	0,0	2,8	0,0	0,9
Contig_489630	pfam00840	Glyco_hydro_7,	2,8	0,0	0,0	0,9
Contig_494422	pfam07971	Glyco_hydro_92,	2,8	0,0	0,0	0,9
Contig_262644	pfam00232	Glyco_hydro_1,	0,9	1,9	0,0	0,9
Contig_300255	pfam03632	Glyco_hydro_65m,	0,0	2,8	0,0	0,9
Contig_370115	pfam00232	Glyco_hydro_1,	2,1	0,7	0,0	0,9
Contig_512383	pfam03663	Glyco_hydro_76,	1,5	1,3	0,0	0,9
Contig_225667	pfam00295	Glyco_hydro_28,	1,1	1,6	0,0	0,9
Contig_453087	pfam00722	Glyco_hydro_16,	0,0	2,8	0,0	0,9
Contig_560439	pfam13199	Glyco_hydro_66,	2,8	0,0	0,0	0,9
Contig_37004	pfam01532	Glyco_hydro_47,	2,0	0,8	0,0	0,9
Contig_368780	pfam07470	Glyco_hydro_88,	1,1	1,7	0,0	0,9
Contig_456325	pfam00331	Glyco_hydro_10,	0,0	2,8	0,0	0,9
Contig_145480	pfam08306	Glyco_hydro_98M,	2,7	0,0	0,0	0,9
Contig_565877	pfam03065	Glyco_hydro_57,	2,7	0,0	0,0	0,9
Contig_88647	pfam03663	Glyco_hydro_76,	2,7	0,0	0,0	0,9
Contig_147125	pfam01373	Glyco_hydro_14,	2,7	0,0	0,0	0,9
Contig_159589	pfam02446	Glyco_hydro_77,	2,7	0,0	0,0	0,9
Contig_167615	pfam07470	Glyco_hydro_88,	2,7	0,0	0,0	0,9
Contig_300931	pfam01630	Glyco_hydro_56,	0,0	2,7	0,0	0,9
Contig_483376	pfam00232	Glyco_hydro_1,	2,7	0,0	0,0	0,9
Contig_516422	pfam03644	Glyco_hydro_85,	2,7	0,0	0,0	0,9
Contig_550922	pfam02449	Glyco_hydro_42,	2,7	0,0	0,0	0,9
Contig_237411	pfam08244	Glyco_hydro_32C,	0,0	2,6	0,0	0,9
Contig_250725	pfam03639	Glyco_hydro_81,	2,6	0,0	0,0	0,9
Contig_320002	pfam12899	Glyco_hydro_100,	2,6	0,0	0,0	0,9
Contig_461749	pfam02324	Glyco_hydro_70,	0,0	2,6	0,0	0,9
Contig_540782	pfam12899	Glyco_hydro_100,	2,6	0,0	0,0	0,9
Contig_293560	pfam03663	Glyco_hydro_76,	1,4	1,3	0,0	0,9
Contig_317832	pfam00840	Glyco_hydro_7,	0,9	1,7	0,0	0,9

Contig_90968	pfam03639	Glyco_hydro_81,	0,0	0,0	2,6	0,9
Contig_214215	pfam08532	Glyco_hydro_42M,	0,9	1,7	0,0	0,9
Contig_575021	pfam00704	Glyco_hydro_18,	2,0	0,6	0,0	0,9
Contig_389808	pfam00728	Glyco_hydro_20,	0,0	2,6	0,0	0,9
Contig_569840	pfam00722	Glyco_hydro_16,	2,6	0,0	0,0	0,9
Contig_36144	pfam00332	Glyco_hydro_17,	1,6	1,0	0,0	0,9
Contig_109730	pfam01183	Glyco_hydro_25,	0,0	2,6	0,0	0,9
Contig_207230	pfam07971	Glyco_hydro_92,	0,0	2,6	0,0	0,9
Contig_506536	pfam03200	Glyco_hydro_63,	2,6	0,0	0,0	0,9
Contig_289539	pfam02446	Glyco_hydro_77,	1,2	1,4	0,0	0,9
Contig_313108	pfam00232	Glyco_hydro_1,	0,0	2,6	0,0	0,9
Contig_403788	pfam07488	Glyco_hydro_67M,	0,9	1,7	0,0	0,9
Contig_482494	pfam00331	Glyco_hydro_10,	0,0	2,6	0,0	0,9
Contig_270641	pfam07745	Glyco_hydro_53,	0,0	2,6	0,0	0,9
Contig_434112	pfam03200	Glyco_hydro_63,	0,0	2,6	0,0	0,9
Contig_112480	pfam03200	Glyco_hydro_63,	2,6	0,0	0,0	0,9
Contig_518894	pfam01055	Glyco_hydro_31,	2,6	0,0	0,0	0,9
Contig_32354	pfam04616	Glyco_hydro_43,	0,0	2,6	0,0	0,9
Contig_318245	pfam02011	Glyco_hydro_48,	0,0	2,6	0,0	0,9
Contig_384326	pfam02837	Glyco_hydro_2_N,	0,0	2,6	0,0	0,9
Contig_427109	pfam07477	Glyco_hydro_67C,	1,4	1,2	0,0	0,9
Contig_464418	pfam03659	Glyco_hydro_71,	2,5	0,0	0,0	0,8
Contig_174592	pfam00933	Glyco_hydro_3,	0,0	2,5	0,0	0,8
Contig_234072	pfam02057	Glyco_hydro_59,	0,0	2,5	0,0	0,8
Contig_327257	pfam03632	Glyco_hydro_65m,	0,0	2,5	0,0	0,8
Contig_76563	pfam11790	Glyco_hydro_cc,	0,0	2,5	0,0	0,8
Contig_325799	pfam12899	Glyco_hydro_100,	0,0	2,5	0,0	0,8
Contig_390507	pfam03198	Glyco_hydro_72,	0,0	2,5	0,0	0,8
Contig_487692	pfam03065	Glyco_hydro_57,	2,1	0,4	0,0	0,8
Contig_283864	pfam13199	Glyco_hydro_66,	0,9	1,6	0,0	0,8
Contig_246279	pfam00728	Glyco_hydro_20,	0,0	2,5	0,0	0,8
Contig_510669	pfam00840	Glyco_hydro_7,	0,0	2,5	0,0	0,8
Contig_14127	pfam03718	Glyco_hydro_49,	0,0	2,5	0,0	0,8
Contig_148704	pfam00704	Glyco_hydro_18,	1,5	0,9	0,0	0,8
Contig_372446	pfam07748	Glyco_hydro_38C,	0,9	1,6	0,0	0,8
Contig_116308	pfam00933	Glyco_hydro_3,	0,0	2,5	0,0	0,8
Contig_250382	pfam01055	Glyco_hydro_31,	0,7	1,7	0,0	0,8
Contig_504920	pfam07971	Glyco_hydro_92,	2,5	0,0	0,0	0,8
Contig_348878	pfam12899	Glyco_hydro_100,	1,1	1,3	0,0	0,8
Contig_36815	pfam07745	Glyco_hydro_53,	0,0	2,4	0,0	0,8
Contig_361974	pfam03718	Glyco_hydro_49,	0,0	2,4	0,0	0,8
Contig_383821	pfam01915	Glyco_hydro_3_C,	0,0	2,4	0,0	0,8
Contig_423033	pfam00232	Glyco_hydro_1,	1,1	1,3	0,0	0,8
Contig_450031	pfam00704	Glyco_hydro_18,	2,2	0,2	0,0	0,8
Contig_107277	pfam11790	Glyco_hydro_cc,	2,4	0,0	0,0	0,8
Contig_147008	pfam03718	Glyco_hydro_49,	2,4	0,0	0,0	0,8
Contig_199082	pfam00251	Glyco_hydro_32N,	0,0	2,4	0,0	0,8
Contig_234365	pfam03198	Glyco_hydro_72,	1,7	0,7	0,0	0,8
Contig_306861	pfam01055	Glyco_hydro_31,	0,0	2,4	0,0	0,8
Contig_367917	pfam01055	Glyco_hydro_31,	0,0	2,4	0,0	0,8
Contig_116430	pfam01532	Glyco_hydro_47,	1,0	1,4	0,0	0,8
Contig_482714	pfam01373	Glyco_hydro_14,	0,0	2,4	0,0	0,8
Contig_512962	pfam00840	Glyco_hydro_7,	1,5	0,9	0,0	0,8
Contig_2356	pfam03065	Glyco_hydro_57,	0,0	2,4	0,0	0,8
Contig_458291	pfam07748	Glyco_hydro_38C,	0,0	2,4	0,0	0,8
Contig_235361	pfam07971	Glyco_hydro_92,	1,5	0,9	0,0	0,8
Contig_269053	pfam00759	Glyco_hydro_9,	0,0	2,4	0,0	0,8
Contig_401212	pfam04616	Glyco_hydro_43,	0,0	2,4	0,0	0,8
Contig_365960	pfam03639	Glyco_hydro_81,	2,4	0,0	0,0	0,8
Contig_471853	pfam00722	Glyco_hydro_16,	1,9	0,5	0,0	0,8
Contig_487695	pfam10566	Glyco_hydro_97,	0,0	2,4	0,0	0,8
Contig_141999	pfam03636	Glyco_hydro_65N,	0,0	2,4	0,0	0,8
Contig_239966	pfam01074	Glyco_hydro_38,	0,0	2,4	0,0	0,8
Contig_518076	pfam04616	Glyco_hydro_43,	0,0	2,4	0,0	0,8
Contig_544041	pfam00722	Glyco_hydro_16,	2,3	0,0	0,0	0,8
Contig_242337	pfam00182	Glyco_hydro_19,	0,0	2,3	0,0	0,8
Contig_142396	pfam00704	Glyco_hydro_18,	2,3	0,0	0,0	0,8
Contig_385359	pfam02446	Glyco_hydro_77,	0,4	2,0	0,0	0,8
Contig_48869	pfam02837	Glyco_hydro_2_N,	0,0	2,3	0,0	0,8
Contig_272492	pfam01074	Glyco_hydro_38,	0,0	2,3	0,0	0,8
Contig_110456	pfam00232	Glyco_hydro_1,	0,0	2,3	0,0	0,8

Contig_362402	pfam07477	Glyco_hydro_67C,	0,0	2,3	0,0	0,8
Contig_452745	pfam03644	Glyco_hydro_85,	2,3	0,0	0,0	0,8
Contig_419270	pfam01183	Glyco_hydro_25,	1,4	0,9	0,0	0,8
Contig_131473	pfam02446	Glyco_hydro_77,	2,2	0,0	0,0	0,7
Contig_124305	pfam00704	Glyco_hydro_18,	2,2	0,0	0,0	0,7
Contig_393394	pfam00840	Glyco_hydro_7,	0,0	2,2	0,0	0,7
Contig_209530	pfam02446	Glyco_hydro_77,	0,0	2,2	0,0	0,7
Contig_255142	pfam01055	Glyco_hydro_31,	0,0	2,2	0,0	0,7
Contig_147030	pfam00251	Glyco_hydro_32N,	2,2	0,0	0,0	0,7
Contig_490027	pfam03659	Glyco_hydro_71,	0,0	2,2	0,0	0,7
Contig_84634	pfam00722	Glyco_hydro_16,	0,0	0,9	1,3	0,7
Contig_366615	pfam03718	Glyco_hydro_49,	0,0	2,2	0,0	0,7
Contig_371887	pfam00728	Glyco_hydro_20,	1,4	0,8	0,0	0,7
Contig_10190	pfam00759	Glyco_hydro_9,	0,0	2,2	0,0	0,7
Contig_410433	pfam00332	Glyco_hydro_17,	1,0	1,2	0,0	0,7
Contig_171195	pfam00933	Glyco_hydro_3,	0,4	1,7	0,0	0,7
Contig_314492	pfam03200	Glyco_hydro_63,	0,0	2,2	0,0	0,7
Contig_414032	pfam08533	Glyco_hydro_42C,	0,0	2,1	0,0	0,7
Contig_377699	pfam00722	Glyco_hydro_16,	0,0	2,1	0,0	0,7
Contig_429357	pfam01055	Glyco_hydro_31,	2,1	0,0	0,0	0,7
Contig_165541	pfam02446	Glyco_hydro_77,	0,0	2,1	0,0	0,7
Contig_312092	pfam01532	Glyco_hydro_47,	0,0	2,1	0,0	0,7
Contig_418906	pfam01055	Glyco_hydro_31,	0,0	2,1	0,0	0,7
Contig_240627	pfam00457	Glyco_hydro_11,	0,0	2,1	0,0	0,7
Contig_493269	pfam04616	Glyco_hydro_43,	0,0	2,1	0,0	0,7
Contig_171513	pfam07745	Glyco_hydro_53,	0,0	2,1	0,0	0,7
Contig_374545	pfam00722	Glyco_hydro_16,	0,0	2,1	0,0	0,7
Contig_385531	pfam02011	Glyco_hydro_48,	1,6	0,5	0,0	0,7
Contig_458450	pfam01532	Glyco_hydro_47,	0,0	2,1	0,0	0,7
Contig_482344	pfam02156	Glyco_hydro_26,	0,0	2,1	0,0	0,7
Contig_354656	pfam12905	Glyco_hydro_101,	1,3	0,8	0,0	0,7
Contig_87968	pfam02446	Glyco_hydro_77,	0,6	1,4	0,0	0,7
Contig_101471	pfam03644	Glyco_hydro_85,	0,0	2,0	0,0	0,7
Contig_227678	pfam12905	Glyco_hydro_101,	0,0	2,0	0,0	0,7
Contig_510329	pfam00759	Glyco_hydro_9,	0,0	2,0	0,0	0,7
Contig_93539	pfam03632	Glyco_hydro_65m,	0,0	0,0	2,0	0,7
Contig_366198	pfam01532	Glyco_hydro_47,	1,7	0,3	0,0	0,7
Contig_273773	pfam00728	Glyco_hydro_20,	0,0	2,0	0,0	0,7
Contig_321892	pfam00232	Glyco_hydro_1,	1,2	0,8	0,0	0,7
Contig_389463	pfam03644	Glyco_hydro_85,	2,0	0,0	0,0	0,7
Contig_418922	pfam04616	Glyco_hydro_43,	0,0	2,0	0,0	0,7
Contig_453488	pfam02836	Glyco_hydro_2_C,	0,0	2,0	0,0	0,7
Contig_482860	pfam02838	Glyco_hydro_20b,	0,0	2,0	0,0	0,7
Contig_144407	pfam01055	Glyco_hydro_31,	1,5	0,5	0,0	0,7
Contig_163396	pfam12899	Glyco_hydro_100,	0,0	2,0	0,0	0,7
Contig_486647	pfam00722	Glyco_hydro_16,	0,0	2,0	0,0	0,7
Contig_418775	pfam00295	Glyco_hydro_28,	1,5	0,5	0,0	0,7
Contig_150534	pfam01183	Glyco_hydro_25,	0,0	1,9	0,0	0,6
Contig_415696	pfam00232	Glyco_hydro_1,	0,0	1,9	0,0	0,6
Contig_183559	pfam03644	Glyco_hydro_85,	0,5	1,4	0,0	0,6
Contig_330559	pfam02055	Glyco_hydro_30,	0,0	1,9	0,0	0,6
Contig_397082	pfam07745	Glyco_hydro_53,	0,0	1,9	0,0	0,6
Contig_572691	pfam02055	Glyco_hydro_30,	1,9	0,0	0,0	0,6
Contig_190114	pfam07488	Glyco_hydro_67M,	0,0	1,9	0,0	0,6
Contig_318794	pfam01374	Glyco_hydro_46,	1,9	0,0	0,0	0,6
Contig_345636	pfam03512	Glyco_hydro_52,	0,0	1,9	0,0	0,6
Contig_134931	pfam00704	Glyco_hydro_18,	0,9	1,0	0,0	0,6
Contig_189400	pfam01074	Glyco_hydro_38,	0,0	1,9	0,0	0,6
Contig_461155	pfam02449	Glyco_hydro_42,	0,0	1,9	0,0	0,6
Contig_415799	pfam02449	Glyco_hydro_42,	0,0	1,9	0,0	0,6
Contig_329686	pfam00704	Glyco_hydro_18,	0,0	1,8	0,0	0,6
Contig_415295	pfam03662	Glyco_hydro_79n,	0,0	1,8	0,0	0,6
Contig_113905	pfam03198	Glyco_hydro_72,	0,0	1,8	0,0	0,6
Contig_280358	pfam04616	Glyco_hydro_43,	0,0	1,8	0,0	0,6
Contig_496183	pfam02055	Glyco_hydro_30,	0,0	1,8	0,0	0,6
Contig_413581	pfam02836	Glyco_hydro_2_C,	0,0	1,8	0,0	0,6
Contig_300086	pfam13199	Glyco_hydro_66,	0,5	1,3	0,0	0,6
Contig_153138	pfam03644	Glyco_hydro_85,	1,8	0,0	0,0	0,6
Contig_496176	pfam00332	Glyco_hydro_17,	0,0	1,8	0,0	0,6
Contig_167394	pfam07477	Glyco_hydro_67C,	0,0	1,8	0,0	0,6
Contig_165596	pfam02449	Glyco_hydro_42,	1,1	0,7	0,0	0,6

Contig_284623	pfam01183	Glyco_hydro_25,	0,0	1,8	0,0	0,6
Contig_447234	pfam00840	Glyco_hydro_7,	0,0	1,7	0,0	0,6
Contig_279409	pfam13199	Glyco_hydro_66,	0,0	1,7	0,0	0,6
Contig_311762	pfam03663	Glyco_hydro_76,	0,0	1,7	0,0	0,6
Contig_375594	pfam00728	Glyco_hydro_20,	0,0	1,7	0,0	0,6
Contig_456825	pfam07971	Glyco_hydro_92,	1,7	0,0	0,0	0,6
Contig_477789	pfam01532	Glyco_hydro_47,	1,7	0,0	0,0	0,6
Contig_571777	pfam03200	Glyco_hydro_63,	1,7	0,0	0,0	0,6
Contig_349157	pfam00331	Glyco_hydro_10,	1,0	0,6	0,0	0,5
Contig_56133	pfam02836	Glyco_hydro_2_C,	0,0	1,6	0,0	0,5
Contig_397103	pfam01373	Glyco_hydro_14,	0,0	1,6	0,0	0,5
Contig_572161	pfam00933	Glyco_hydro_3,	0,5	1,2	0,0	0,5
Contig_20491	pfam01074	Glyco_hydro_38,	0,0	1,6	0,0	0,5
Contig_94685	pfam03639	Glyco_hydro_81,	0,0	1,6	0,0	0,5
Contig_464185	pfam07488	Glyco_hydro_67M,	0,0	1,6	0,0	0,5
Contig_515775	pfam00232	Glyco_hydro_1,	1,0	0,6	0,0	0,5
Contig_245855	pfam00331	Glyco_hydro_10,	0,0	1,6	0,0	0,5
Contig_456863	pfam08244	Glyco_hydro_32C,	1,6	0,0	0,0	0,5
Contig_95484	pfam00331	Glyco_hydro_10,	1,5	0,0	0,0	0,5
Contig_547112	pfam03200	Glyco_hydro_63,	1,5	0,0	0,0	0,5
Contig_513912	pfam00232	Glyco_hydro_1,	0,0	1,5	0,0	0,5
Contig_120721	pfam02055	Glyco_hydro_30,	1,2	0,4	0,0	0,5
Contig_323924	pfam04616	Glyco_hydro_43,	0,0	1,5	0,0	0,5
Contig_450119	pfam00759	Glyco_hydro_9,	1,5	0,0	0,0	0,5
Contig_414640	pfam03632	Glyco_hydro_65m,	1,5	0,0	0,0	0,5
Contig_150899	pfam02838	Glyco_hydro_20b,	0,0	1,5	0,0	0,5
Contig_168966	pfam01229	Glyco_hydro_39,	0,0	1,5	0,0	0,5
Contig_367792	pfam02015	Glyco_hydro_45,	0,0	1,5	0,0	0,5
Contig_300343	pfam07470	Glyco_hydro_88,	0,0	1,5	0,0	0,5
Contig_223032	pfam01055	Glyco_hydro_31,	0,0	1,5	0,0	0,5
Contig_128628	pfam13199	Glyco_hydro_66,	0,0	1,5	0,0	0,5
Contig_213369	pfam00232	Glyco_hydro_1,	0,0	1,5	0,0	0,5
Contig_321142	pfam00704	Glyco_hydro_18,	0,0	1,5	0,0	0,5
Contig_117719	pfam03636	Glyco_hydro_65N,	0,0	1,4	0,0	0,5
Contig_292310	pfam07477	Glyco_hydro_67C,	0,0	1,4	0,0	0,5
Contig_389824	pfam07745	Glyco_hydro_53,	0,0	1,4	0,0	0,5
Contig_344009	pfam02435	Glyco_hydro_68,	1,4	0,0	0,0	0,5
Contig_437113	pfam03718	Glyco_hydro_49,	0,0	1,4	0,0	0,5
Contig_102069	pfam03718	Glyco_hydro_49,	1,4	0,0	0,0	0,5
Contig_324189	pfam07488	Glyco_hydro_67M,	0,0	1,4	0,0	0,5
Contig_303843	pfam02446	Glyco_hydro_77,	0,0	1,4	0,0	0,5
Contig_422067	pfam00722	Glyco_hydro_16,	1,4	0,0	0,0	0,5
Contig_271251	pfam01532	Glyco_hydro_47,	0,0	1,4	0,0	0,5
Contig_263790	pfam00759	Glyco_hydro_9,	0,0	1,3	0,0	0,4
Contig_396968	pfam01229	Glyco_hydro_39,	0,0	1,3	0,0	0,4
Contig_6853	pfam00332	Glyco_hydro_17,	0,0	1,3	0,0	0,4
Contig_39899	pfam03200	Glyco_hydro_63,	1,3	0,0	0,0	0,4
Contig_519772	pfam03639	Glyco_hydro_81,	0,0	1,3	0,0	0,4
Contig_204024	pfam02435	Glyco_hydro_68,	0,5	0,8	0,0	0,4
Contig_231344	pfam00232	Glyco_hydro_1,	0,0	1,3	0,0	0,4
Contig_509792	pfam02435	Glyco_hydro_68,	1,3	0,0	0,0	0,4
Contig_174833	pfam07477	Glyco_hydro_67C,	0,8	0,5	0,0	0,4
Contig_1258	pfam07477	Glyco_hydro_67C,	1,2	0,0	0,0	0,4
Contig_208233	pfam03662	Glyco_hydro_79n,	0,0	1,2	0,0	0,4
Contig_304547	pfam03443	Glyco_hydro_61,	0,0	1,2	0,0	0,4
Contig_299638	pfam00457	Glyco_hydro_11,	1,2	0,0	0,0	0,4
Contig_574243	pfam07971	Glyco_hydro_92,	1,2	0,0	0,0	0,4
Contig_338648	pfam00933	Glyco_hydro_3,	0,0	1,2	0,0	0,4
Contig_283272	pfam07971	Glyco_hydro_92,	0,0	1,1	0,0	0,4
Contig_461826	pfam07971	Glyco_hydro_92,	0,0	1,1	0,0	0,4
Contig_105991	pfam02156	Glyco_hydro_26,	0,0	1,1	0,0	0,4
Contig_147680	pfam01074	Glyco_hydro_38,	1,1	0,0	0,0	0,4
Contig_251554	pfam03639	Glyco_hydro_81,	0,0	1,0	0,0	0,3
Contig_258578	pfam13199	Glyco_hydro_66,	0,0	1,0	0,0	0,3
Contig_117072	pfam01229	Glyco_hydro_39,	0,0	1,0	0,0	0,3
Contig_373323	pfam11790	Glyco_hydro_cc,	0,0	1,0	0,0	0,3
Contig_87966	pfam03639	Glyco_hydro_81,	0,0	1,0	0,0	0,3
Contig_372749	pfam00295	Glyco_hydro_28,	0,0	1,0	0,0	0,3
Contig_160963	pfam00182	Glyco_hydro_19,	0,0	1,0	0,0	0,3
Contig_238288	pfam00251	Glyco_hydro_32N,	0,0	1,0	0,0	0,3
Contig_319904	pfam01630	Glyco_hydro_56,	0,0	1,0	0,0	0,3

Contig_343216	pfam02324	Glyco_hydro_70,	0,0	1,0	0,0	0,3
Contig_303833	pfam02449	Glyco_hydro_42,	0,0	0,9	0,0	0,3
Contig_73733	pfam03200	Glyco_hydro_63,	0,0	0,9	0,0	0,3
Contig_263879	pfam02011	Glyco_hydro_48,	0,0	0,9	0,0	0,3
Contig_313787	pfam02324	Glyco_hydro_70,	0,0	0,9	0,0	0,3
Contig_354949	pfam00759	Glyco_hydro_9,	0,0	0,9	0,0	0,3
Contig_382819	pfam00759	Glyco_hydro_9,	0,0	0,9	0,0	0,3
Contig_391170	pfam02324	Glyco_hydro_70,	0,0	0,9	0,0	0,3
Contig_139906	pfam02324	Glyco_hydro_70,	0,0	0,9	0,0	0,3
Contig_196777	pfam01055	Glyco_hydro_31,	0,0	0,9	0,0	0,3
Contig_215963	pfam00251	Glyco_hydro_32N,	0,0	0,9	0,0	0,3
Contig_42975	pfam00759	Glyco_hydro_9,	0,9	0,0	0,0	0,3
Contig_302958	pfam03644	Glyco_hydro_85,	0,0	0,9	0,0	0,3
Contig_489558	pfam01229	Glyco_hydro_39,	0,0	0,8	0,0	0,3
Contig_573552	pfam00933	Glyco_hydro_3,	0,0	0,8	0,0	0,3
Contig_290428	pfam00295	Glyco_hydro_28,	0,0	0,8	0,0	0,3
Contig_402073	pfam00232	Glyco_hydro_1,	0,0	0,8	0,0	0,3
Contig_146656	pfam02324	Glyco_hydro_70,	0,0	0,8	0,0	0,3
Contig_215994	pfam00332	Glyco_hydro_17,	0,0	0,8	0,0	0,3
Contig_432876	pfam00457	Glyco_hydro_11,	0,0	0,8	0,0	0,3
Contig_407543	pfam02449	Glyco_hydro_42,	0,0	0,8	0,0	0,3
Contig_240247	pfam01301	Glyco_hydro_35,	0,0	0,8	0,0	0,3
Contig_160138	pfam01055	Glyco_hydro_31,	0,0	0,8	0,0	0,3
Contig_552197	pfam01630	Glyco_hydro_56,	0,8	0,0	0,0	0,3
Contig_408597	pfam13199	Glyco_hydro_66,	0,0	0,8	0,0	0,3
Contig_434775	pfam08307	Glyco_hydro_98C,	0,0	0,8	0,0	0,3
Contig_284066	pfam01074	Glyco_hydro_38,	0,0	0,7	0,0	0,2
Contig_443722	pfam00933	Glyco_hydro_3,	0,0	0,7	0,0	0,2
Contig_512550	pfam01055	Glyco_hydro_31,	0,0	0,7	0,0	0,2
Contig_573671	pfam00295	Glyco_hydro_28,	0,0	0,7	0,0	0,2
Contig_268390	pfam07745	Glyco_hydro_53,	0,0	0,6	0,0	0,2
Contig_253421	pfam00723	Glyco_hydro_15,	0,0	0,6	0,0	0,2
Contig_303709	pfam07477	Glyco_hydro_67C,	0,0	0,6	0,0	0,2
Contig_168547	pfam00704	Glyco_hydro_18,	0,0	0,6	0,0	0,2
Contig_502006	pfam11790	Glyco_hydro_cc,	0,0	0,6	0,0	0,2
Contig_449395	pfam00704	Glyco_hydro_18,	0,0	0,5	0,0	0,2
Contig_397983	pfam01055	Glyco_hydro_31,	0,0	0,4	0,0	0,1
Contig_376287	pfam01074	Glyco_hydro_38,	0,0	0,3	0,0	0,1
Contig_565543	pfam12899	Glyco_hydro_100,	0,0	0,3	0,0	0,1
Contig_44781	pfam00722	Glyco_hydro_16,	0,0	0,0	0,0	0,0
Contig_50612	pfam01074	Glyco_hydro_38,	0,0	0,0	0,0	0,0
Contig_54954	pfam07477	Glyco_hydro_67C,	0,0	0,0	0,0	0,0
Contig_57226	pfam00722	Glyco_hydro_16,	0,0	0,0	0,0	0,0
Contig_92038	pfam01270	Glyco_hydro_8,	0,0	0,0	0,0	0,0
Contig_95323	pfam01532	Glyco_hydro_47,	0,0	0,0	0,0	0,0
Contig_98788	pfam03639	Glyco_hydro_81,	0,0	0,0	0,0	0,0
Contig_106012	pfam04616	Glyco_hydro_43,	0,0	0,0	0,0	0,0
Contig_107744	pfam00704	Glyco_hydro_18,	0,0	0,0	0,0	0,0
Contig_110962	pfam00728	Glyco_hydro_20,	0,0	0,0	0,0	0,0
Contig_122435	pfam02446	Glyco_hydro_77,	0,0	0,0	0,0	0,0
Contig_123818	pfam03644	Glyco_hydro_85,	0,0	0,0	0,0	0,0
Contig_128525	pfam01229	Glyco_hydro_39,	0,0	0,0	0,0	0,0
Contig_134388	pfam03659	Glyco_hydro_71,	0,0	0,0	0,0	0,0
Contig_151181	pfam00722	Glyco_hydro_16,	0,0	0,0	0,0	0,0
Contig_167902	pfam03200	Glyco_hydro_63,	0,0	0,0	0,0	0,0
Contig_184801	pfam00759	Glyco_hydro_9,	0,0	0,0	0,0	0,0
Contig_189462	pfam07971	Glyco_hydro_92,	0,0	0,0	0,0	0,0
Contig_196748	pfam03663	Glyco_hydro_76,	0,0	0,0	0,0	0,0
Contig_202939	pfam03648	Glyco_hydro_67N,	0,0	0,0	0,0	0,0
Contig_206720	pfam03065	Glyco_hydro_57,	0,0	0,0	0,0	0,0
Contig_206736	pfam07971	Glyco_hydro_92,	0,0	0,0	0,0	0,0
Contig_206971	pfam01670	Glyco_hydro_12,	0,0	0,0	0,0	0,0
Contig_210406	pfam00295	Glyco_hydro_28,	0,0	0,0	0,0	0,0
Contig_221113	pfam02449	Glyco_hydro_42,	0,0	0,0	0,0	0,0
Contig_244274	pfam02449	Glyco_hydro_42,	0,0	0,0	0,0	0,0
Contig_261898	pfam13199	Glyco_hydro_66,	0,0	0,0	0,0	0,0
Contig_281218	pfam03065	Glyco_hydro_57,	0,0	0,0	0,0	0,0
Contig_302194	pfam00704	Glyco_hydro_18,	0,0	0,0	0,0	0,0
Contig_322032	pfam07971	Glyco_hydro_92,	0,0	0,0	0,0	0,0
Contig_324487	pfam01055	Glyco_hydro_31,	0,0	0,0	0,0	0,0
Contig_327179	pfam00251	Glyco_hydro_32N,	0,0	0,0	0,0	0,0

Contig_330997	pfam01055	Glyco_hydro_31,	0,0	0,0	0,0	0,0
Contig_337411	pfam03659	Glyco_hydro_71,	0,0	0,0	0,0	0,0
Contig_360341	pfam01074	Glyco_hydro_38,	0,0	0,0	0,0	0,0
Contig_377282	pfam07971	Glyco_hydro_92,	0,0	0,0	0,0	0,0
Contig_381993	pfam07477	Glyco_hydro_67C,	0,0	0,0	0,0	0,0
Contig_419189	pfam03200	Glyco_hydro_63,	0,0	0,0	0,0	0,0
Contig_441667	pfam02057	Glyco_hydro_59,	0,0	0,0	0,0	0,0
Contig_446792	pfam03200	Glyco_hydro_63,	0,0	0,0	0,0	0,0
Contig_454027	pfam02057	Glyco_hydro_59,	0,0	0,0	0,0	0,0
Contig_460861	pfam01074	Glyco_hydro_38,	0,0	0,0	0,0	0,0
Contig_479647	pfam03639	Glyco_hydro_81,	0,0	0,0	0,0	0,0
Contig_482410	pfam00728	Glyco_hydro_20,	0,0	0,0	0,0	0,0
Contig_487595	pfam01630	Glyco_hydro_56,	0,0	0,0	0,0	0,0
Contig_496326	pfam03659	Glyco_hydro_71,	0,0	0,0	0,0	0,0
Contig_502349	pfam01055	Glyco_hydro_31,	0,0	0,0	0,0	0,0
Contig_556000	pfam07470	Glyco_hydro_88,	0,0	0,0	0,0	0,0
Contig_557955	pfam03639	Glyco_hydro_81,	0,0	0,0	0,0	0,0
Contig_564113	pfam13199	Glyco_hydro_66,	0,0	0,0	0,0	0,0
Contig_565932	pfam00331	Glyco_hydro_10,	0,0	0,0	0,0	0,0
Contig_576908	pfam02156	Glyco_hydro_26,	0,0	0,0	0,0	0,0
Contig_580018	pfam01630	Glyco_hydro_56,	0,0	0,0	0,0	0,0
Contig_580085	pfam07470	Glyco_hydro_88,	0,0	0,0	0,0	0,0
Contig_584028	pfam02837	Glyco_hydro_2_N,	0,0	0,0	0,0	0,0
Contig_584409	pfam02015	Glyco_hydro_45,	0,0	0,0	0,0	0,0

**Contig ID**	**Pfam ID**	**Short Descrip**	**AK7**	**AK8**	**AK9**	**Average**
Contig_192071	pfam00150	Cellulase,	39,6	27,1	92,7	53,1
Contig_233281	pfam00150	Cellulase,	30,8	38,0	41,8	36,9
Contig_429541	pfam00150	Cellulase,	26,6	23,9	40,4	30,3
Contig_77808	pfam00150	Cellulase,	19,3	19,3	31,5	23,4
Contig_482108	pfam00150	Cellulase,	16,5	25,1	21,6	21,0
Contig_93747	pfam00150	Cellulase,	6,6	12,0	43,0	20,5
Contig_101242	pfam00150	Cellulase,	1,4	6,8	52,7	20,3
Contig_81676	pfam00150	Cellulase,	4,3	13,0	19,3	12,2
Contig_229168	pfam00150	Cellulase,	11,7	8,5	14,6	11,6
Contig_137022	pfam00150	Cellulase,	11,4	2,4	20,6	11,5
Contig_206753	pfam00150	Cellulase,	10,3	17,5	2,3	10,0
Contig_76049	pfam00150	Cellulase,	18,5	7,1	2,1	9,2
Contig_177505	pfam00150	Cellulase,	13,8	10,8	1,5	8,7
Contig_216732	pfam00150	Cellulase,	4,0	20,7	0,0	8,2
Contig_278831	pfam00150	Cellulase,	6,9	10,4	5,7	7,7
Contig_75991	pfam00150	Cellulase,	4,6	2,8	15,5	7,6
Contig_17304	pfam00150	Cellulase,	2,5	4,6	12,6	6,6
Contig_67767	pfam00150	Cellulase,	0,0	0,9	18,3	6,4
Contig_117463	pfam00150	Cellulase,	9,7	4,9	3,7	6,1
Contig_97649	pfam00150	Cellulase,	1,9	5,8	9,6	5,8
Contig_9369	pfam00150	Cellulase,	2,2	8,5	5,4	5,4
Contig_147832	pfam00150	Cellulase,	3,7	0,0	12,4	5,4
Contig_75207	pfam00150	Cellulase,	9,4	6,6	0,0	5,3
Contig_443695	pfam00150	Cellulase,	11,4	4,4	0,0	5,2
Contig_513932	pfam00150	Cellulase,	6,7	7,7	0,0	4,8
Contig_75205	pfam00150	Cellulase,	10,7	3,5	0,0	4,7
Contig_221830	pfam00150	Cellulase,	2,1	5,6	5,1	4,2
Contig_217621	pfam00150	Cellulase,	4,1	7,3	0,0	3,8
Contig_177504	pfam00150	Cellulase,	9,1	2,0	0,0	3,7
Contig_63315	pfam00150	Cellulase,	4,8	6,1	0,0	3,6
Contig_477866	pfam00150	Cellulase,	2,2	4,8	3,6	3,5
Contig_408909	pfam00150	Cellulase,	6,4	3,8	0,0	3,4
Contig_280349	pfam00150	Cellulase,	6,2	3,8	0,0	3,3
Contig_277194	pfam00150	Cellulase,	4,1	5,8	0,0	3,3
Contig_481744	pfam00150	Cellulase,	3,8	5,8	0,0	3,2
Contig_182726	pfam00150	Cellulase,	2,3	6,9	0,0	3,1
Contig_302228	pfam00150	Cellulase,	1,3	7,6	0,0	3,0
Contig_518762	pfam00150	Cellulase,	2,8	5,9	0,0	2,9
Contig_75051	pfam00150	Cellulase,	2,1	3,7	2,6	2,8
Contig_492665	pfam00150	Cellulase,	7,3	0,9	0,0	2,7
Contig_299965	pfam00150	Cellulase,	4,5	3,6	0,0	2,7
Contig_92932	pfam00150	Cellulase,	3,8	4,3	0,0	2,7
Contig_151706	pfam00150	Cellulase,	0,0	2,3	5,7	2,7
Contig_319969	pfam00150	Cellulase,	5,9	1,6	0,0	2,5
Contig_468776	pfam00150	Cellulase,	4,8	2,3	0,0	2,4
Contig_216273	pfam00150	Cellulase,	3,8	3,1	0,0	2,3

Total expressed genes	4784132
Sum of expressed* cellulase genes	440
**% of cellulase family**	**0,01**

* FPKM normalized

Contig_399144	pfam00150	Cellulase,	0,0	3,3	3,4	2,3
Contig_251339	pfam00150	Cellulase,	3,2	3,4	0,0	2,2
Contig_551350	pfam00150	Cellulase,	6,2	0,0	0,0	2,1
Contig_157206	pfam00150	Cellulase,	2,4	3,2	0,5	2,1
Contig_202341	pfam00150	Cellulase,	0,0	5,9	0,0	2,0
Contig_443842	pfam00150	Cellulase,	0,0	5,7	0,0	1,9
Contig_376353	pfam00150	Cellulase,	1,6	3,8	0,0	1,8
Contig_156574	pfam00150	Cellulase,	1,1	3,8	0,0	1,6
Contig_323173	pfam00150	Cellulase,	0,0	2,8	1,9	1,6
Contig_329613	pfam00150	Cellulase,	4,6	0,0	0,0	1,5
Contig_379766	pfam00150	Cellulase,	4,1	0,4	0,0	1,5
Contig_127823	pfam00150	Cellulase,	3,0	1,5	0,0	1,5
Contig_137901	pfam00150	Cellulase,	1,7	0,5	2,2	1,5
Contig_211438	pfam00150	Cellulase,	4,2	0,0	0,0	1,4
Contig_370779	pfam00150	Cellulase,	0,5	3,7	0,0	1,4
Contig_320733	pfam00150	Cellulase,	2,4	1,5	0,0	1,3
Contig_492531	pfam00150	Cellulase,	1,4	1,7	0,0	1,0
Contig_507895	pfam00150	Cellulase,	1,7	1,0	0,0	0,9
Contig_128588	pfam00150	Cellulase,	0,0	1,4	0,0	0,5
Contig_468902	pfam00150	Cellulase,	0,0	1,1	0,0	0,4
Contig_468902	pfam00150	Cellulase,	0,0	1,1	0,0	0,4

**Contig ID**	**Pfam ID**	**Short Descrip**	**AK7**	**AK8**	**AK9**	**Average**
Contig_525118	pfam13201	Xylanase,	113,3	85,9	56,0	85,1
Contig_226673	pfam13201	Xylanase,	37,1	46,5	143,3	75,7
Contig_521167	pfam13201	Xylanase,	41,3	34,4	56,0	43,9
Contig_267093	pfam13201	Xylanase,	26,5	27,1	41,7	31,8
Contig_554146	pfam13201	Xylanase,	4,1	14,9	37,8	19,0
Contig_567527	pfam13201	Xylanase,	8,7	25,7	15,9	16,7
Contig_427862	pfam13201	Xylanase,	13,5	13,5	14,0	13,7
Contig_542099	pfam13201	Xylanase,	7,9	0,0	31,6	13,2
Contig_201617	pfam13201	Xylanase,	7,6	11,5	19,1	12,8
Contig_98393	pfam13201	Xylanase,	0,0	0,0	32,7	10,9
Contig_254436	pfam13201	Xylanase,	3,0	18,2	11,3	10,8
Contig_93637	pfam13201	Xylanase,	1,4	2,5	23,7	9,2
Contig_444767	pfam13201	Xylanase,	3,2	6,2	11,8	7,1
Contig_53552	pfam13201	Xylanase,	6,9	1,8	10,6	6,4
Contig_335299	pfam13201	Xylanase,	17,1	2,2	0,0	6,4
Contig_341306	pfam13201	Xylanase,	11,1	5,9	0,0	5,6
Contig_37796	pfam13201	Xylanase,	4,8	0,0	12,0	5,6
Contig_150692	pfam13201	Xylanase,	2,3	2,4	8,4	4,3
Contig_499055	pfam13201	Xylanase,	9,3	2,4	0,0	3,9
Contig_26399	pfam13201	Xylanase,	1,9	3,3	6,1	3,8
Contig_249033	pfam13201	Xylanase,	1,2	9,7	0,0	3,6
Contig_32136	pfam13201	Xylanase,	0,0	8,0	2,5	3,5
Contig_249709	pfam13201	Xylanase,	7,4	2,4	0,0	3,3
Contig_341782	pfam13201	Xylanase,	7,3	2,5	0,0	3,3
Contig_282275	pfam13201	Xylanase,	7,4	2,1	0,0	3,2
Contig_448908	pfam13201	Xylanase,	2,5	5,9	0,0	2,8
Contig_510167	pfam13201	Xylanase,	3,5	4,9	0,0	2,8
Contig_478403	pfam13201	Xylanase,	5,5	2,7	0,0	2,7
Contig_316917	pfam13201	Xylanase,	3,8	4,1	0,0	2,6
Contig_127073	pfam13201	Xylanase,	0,0	0,8	6,7	2,5
Contig_260015	pfam13201	Xylanase,	6,6	0,8	0,0	2,5
Contig_476174	pfam13201	Xylanase,	2,3	4,9	0,0	2,4
Contig_226632	pfam13201	Xylanase,	4,4	2,7	0,0	2,4
Contig_493995	pfam13201	Xylanase,	0,0	6,4	0,0	2,1
Contig_572162	pfam13201	Xylanase,	6,2	0,0	0,0	2,1
Contig_327732	pfam13201	Xylanase,	2,7	3,3	0,0	2,0
Contig_171462	pfam13201	Xylanase,	2,5	3,5	0,0	2,0
Contig_177698	pfam13201	Xylanase,	0,0	5,9	0,0	2,0
Contig_512993	pfam13201	Xylanase,	2,8	2,3	0,0	1,7
Contig_14138	pfam13201	Xylanase,	1,6	2,0	1,4	1,7
Contig_164276	pfam13201	Xylanase,	1,1	3,8	0,0	1,6
Contig_481079	pfam13201	Xylanase,	1,7	1,5	0,0	1,1
Contig_325493	pfam13201	Xylanase,	0,0	3,0	0,0	1,0
Contig_426639	pfam13201	Xylanase,	0,0	3,0	0,0	1,0
Contig_156176	pfam13201	Xylanase,	0,0	2,8	0,0	0,9
Contig_492075	pfam13201	Xylanase,	2,1	0,6	0,0	0,9
Contig_414329	pfam13201	Xylanase,	0,0	2,2	0,0	0,7
Contig_100201	pfam13201	Xylanase,	1,2	1,0	0,0	0,7
Contig_106410	pfam13201	Xylanase,	0,9	1,1	0,0	0,7

Total expressed genes	4784132
Sum of expressed* xylanase genes	447
**% of xylanase family**	**0,01**

* FPKM normalized

Contig_506180	pfam13201	Xylanase,	1,3	0,8	0,0	0,7
Contig_349766	pfam13201	Xylanase,	1,2	0,7	0,0	0,7
Contig_251268	pfam13201	Xylanase,	0,0	1,5	0,0	0,5
Contig_295965	pfam13201	Xylanase,	0,0	0,7	0,0	0,2
Contig_484164	pfam13201	Xylanase,	0,0	0,0	0,0	0,0

**Contig ID**	**Pfam ID**	**Short Descrip**	**AK7**	**AK8**	**AK9**	**Average**
Contig_12446	pfam00141	peroxidase,	0,0	4,3	0,0	1,4
Contig_13488	pfam00141	peroxidase,	0,0	4,7	12,3	5,7
Contig_14577	pfam00141	peroxidase,	9,5	6,5	23,8	13,2
Contig_20987	pfam00141	peroxidase,	4,5	0,8	3,2	2,8
Contig_26080	pfam00141	peroxidase,	19,6	18,5	14,1	17,4
Contig_29621	pfam00141	peroxidase,	0,7	1,7	2,9	1,8
Contig_33622	pfam00141	peroxidase,	2,2	2,0	11,1	5,1
Contig_42069	pfam00141	peroxidase,	0,8	12,4	28,8	14,0
Contig_44867	pfam00141	peroxidase,	7,7	4,2	0,5	4,1
Contig_60883	pfam00141	peroxidase,	24,6	18,5	12,3	18,5
Contig_61386	pfam00141	peroxidase,	5,7	6,8	0,0	4,2
Contig_64634	pfam00141	peroxidase,	22,5	43,0	40,7	35,4
Contig_72850	pfam00141	peroxidase,	1,1	7,1	10,7	6,3
Contig_79549	pfam00141	peroxidase,	3,7	1,4	8,0	4,3
Contig_93428	pfam00141	peroxidase,	0,0	2,1	1,5	1,2
Contig_93809	pfam00141	peroxidase,	1,8	0,0	6,7	2,8
Contig_97906	pfam00141	peroxidase,	7,0	6,0	0,0	4,4
Contig_105895	pfam00141	peroxidase,	8,4	0,0	3,5	4,0
Contig_109408	pfam00141	peroxidase,	4,4	1,8	34,9	13,7
Contig_118682	pfam00141	peroxidase,	1,0	2,8	0,0	1,3
Contig_119731	pfam00141	peroxidase,	7,8	2,0	6,9	5,6
Contig_128475	pfam00141	peroxidase,	9,3	15,6	8,7	11,2
Contig_138547	pfam00141	peroxidase,	0,0	1,5	10,0	3,8
Contig_146711	pfam00141	peroxidase,	2,6	1,6	5,4	3,2
Contig_147223	pfam00141	peroxidase,	0,0	0,9	3,6	1,5
Contig_151184	pfam00141	peroxidase,	0,0	14,1	0,0	4,7
Contig_152535	pfam00141	peroxidase,	9,2	0,0	2,9	4,0
Contig_158075	pfam00141	peroxidase,	20,5	23,4	29,7	24,5
Contig_163559	pfam00141	peroxidase,	0,0	2,8	0,0	0,9
Contig_164621	pfam00141	peroxidase,	6,2	2,2	0,0	2,8
Contig_174541	pfam00141	peroxidase,	32,1	34,7	29,8	32,2
Contig_184988	pfam00141	peroxidase,	5,2	4,0	0,0	3,1
Contig_185968	pfam00141	peroxidase,	9,7	18,0	20,9	16,2
Contig_186211	pfam00141	peroxidase,	10,5	9,6	3,4	7,8
Contig_186883	pfam00141	peroxidase,	21,8	22,5	30,0	24,8
Contig_201313	pfam00141	peroxidase,	12,2	19,1	9,0	13,4
Contig_205001	pfam00141	peroxidase,	0,0	16,6	0,0	5,5
Contig_207660	pfam00141	peroxidase,	1,0	4,1	0,0	1,7
Contig_218391	pfam00141	peroxidase,	9,5	16,1	16,8	14,1
Contig_227512	pfam00141	peroxidase,	20,4	38,5	23,8	27,6
Contig_230799	pfam00141	peroxidase,	25,6	16,5	37,3	26,5
Contig_235565	pfam00141	peroxidase,	11,8	16,0	27,5	18,5
Contig_235988	pfam00141	peroxidase,	9,6	2,9	0,0	4,2
Contig_239030	pfam00141	peroxidase,	1,8	1,6	0,0	1,1
Contig_239483	pfam00141	peroxidase,	6,7	4,0	0,0	3,6
Contig_243750	pfam00141	peroxidase,	0,8	1,4	0,0	0,7
Contig_245747	pfam00141	peroxidase,	14,8	11,7	0,0	8,8
Contig_256159	pfam00141	peroxidase,	8,2	11,3	0,0	6,5
Contig_257810	pfam00141	peroxidase,	0,0	5,1	0,0	1,7
Contig_261752	pfam00141	peroxidase,	0,0	1,7	0,0	0,6
Contig_262862	pfam00141	peroxidase,	0,0	5,7	0,0	1,9
Contig_263572	pfam00141	peroxidase,	1,5	3,7	0,0	1,8
Contig_263944	pfam00141	peroxidase,	4,9	1,0	0,0	2,0
Contig_265295	pfam00141	peroxidase,	1,8	6,6	0,0	2,8
Contig_267928	pfam00141	peroxidase,	0,0	0,9	0,0	0,3
Contig_273710	pfam00141	peroxidase,	21,8	0,0	0,0	7,3
Contig_274818	pfam00141	peroxidase,	21,3	14,4	14,5	16,8
Contig_275923	pfam00141	peroxidase,	5,1	8,7	0,0	4,6
Contig_278615	pfam00141	peroxidase,	19,0	17,4	18,1	18,2
Contig_283059	pfam00141	peroxidase,	6,8	2,5	0,0	3,1
Contig_284813	pfam00141	peroxidase,	0,6	0,4	0,0	0,3
Contig_289721	pfam00141	peroxidase,	6,5	19,0	84,1	36,5
Contig_297939	pfam00141	peroxidase,	0,0	16,4	0,0	5,5
Contig_298968	pfam00141	peroxidase,	8,9	5,4	0,0	4,8
Contig_311202	pfam00141	peroxidase,	4,2	8,5	0,0	4,3

Total expressed genes	4784132
Sum of expressed* peroxidase genes	826
**% of peroxidase family**	**0,02**

* FPKM normalized

Contig_325725	pfam00141	peroxidase,	3,3	1,3	0,0	1,5
Contig_330947	pfam00141	peroxidase,	4,9	2,9	0,0	2,6
Contig_331078	pfam00141	peroxidase,	0,0	0,8	0,0	0,3
Contig_332924	pfam00141	peroxidase,	3,4	1,6	0,0	1,7
Contig_350571	pfam00141	peroxidase,	1,8	3,2	0,0	1,6
Contig_356383	pfam00141	peroxidase,	1,1	1,5	0,0	0,8
Contig_370775	pfam00141	peroxidase,	7,0	3,2	0,0	3,4
Contig_382940	pfam00141	peroxidase,	12,3	19,4	18,9	16,9
Contig_386515	pfam00141	peroxidase,	1,2	11,8	0,0	4,3
Contig_397893	pfam00141	peroxidase,	9,4	0,0	0,0	3,1
Contig_403740	pfam00141	peroxidase,	6,1	0,0	0,0	2,0
Contig_406444	pfam00141	peroxidase,	3,5	1,2	0,0	1,6
Contig_411824	pfam00141	peroxidase,	0,0	3,6	0,0	1,2
Contig_418965	pfam00141	peroxidase,	1,6	2,0	0,0	1,2
Contig_425715	pfam00141	peroxidase,	1,6	1,4	0,0	1,0
Contig_433174	pfam00141	peroxidase,	28,5	22,7	0,0	17,1
Contig_434226	pfam00141	peroxidase,	3,6	2,2	0,0	1,9
Contig_434923	pfam00141	peroxidase,	17,5	16,7	29,3	21,2
Contig_440165	pfam00141	peroxidase,	22,8	23,6	20,5	22,3
Contig_445704	pfam00141	peroxidase,	9,7	16,9	0,0	8,9
Contig_454302	pfam00141	peroxidase,	0,0	0,0	0,0	0,0
Contig_465816	pfam00141	peroxidase,	12,7	12,5	14,5	13,2
Contig_477672	pfam00141	peroxidase,	0,0	4,6	0,0	1,5
Contig_482202	pfam00141	peroxidase,	0,0	2,6	19,0	7,2
Contig_488667	pfam00141	peroxidase,	2,2	3,3	0,0	1,8
Contig_495279	pfam00141	peroxidase,	1,6	3,9	0,0	1,8
Contig_497578	pfam00141	peroxidase,	10,0	14,4	32,2	18,9
Contig_497838	pfam00141	peroxidase,	9,9	4,1	0,0	4,7
Contig_502845	pfam00141	peroxidase,	29,4	23,6	5,4	19,5
Contig_506914	pfam00141	peroxidase,	1,3	0,0	0,0	0,4
Contig_525107	pfam00141	peroxidase,	25,6	10,7	13,9	16,7
Contig_536230	pfam00141	peroxidase,	1,5	0,9	0,0	0,8
Contig_538029	pfam00141	peroxidase,	2,6	1,6	0,0	1,4
Contig_539766	pfam00141	peroxidase,	4,3	0,0	0,0	1,4
Contig_543715	pfam00141	peroxidase,	10,9	11,3	43,0	21,7
Contig_562119	pfam00141	peroxidase,	21,6	13,1	0,0	11,6
Contig_571855	pfam00141	peroxidase,	6,1	3,3	0,0	3,1
Contig_572789	pfam00141	peroxidase,	12,9	13,0	51,1	25,7
Contig_577675	pfam00141	peroxidase,	12,6	7,6	0,0	6,7
Contig_582527	pfam00141	peroxidase,	0,0	4,0	0,0	1,3
