# Supplementary material for: Hunters or farmers? Microbiome characteristics help elucidate the diet composition in an aquatic carnivorous plant
Source: Microbiome. 2018 Dec 17;6:225. doi: 10.1186/s40168-018-0600-7 (PMC6297986; doi:10.1186/s40168-018-0600-7)
Supplement: Supplementary file 5 — Table S8. Functional annotation of OTUs (only for those with genus assigment) genes. Annotation was based on RDP Fungene database ver. 8.3. (PDF 673 kb) [file 40168_2018_600_MOESM5_ESM.pdf]

Table S8: Functional annotation of OTUs (only for those with **genus** assigment) genes. Annotation was based on RDP Fungene database ver. 8.3

SampleID	5	12	4	13	9	6	11	10	14	taxonomy (ARB Silva v.119)					
	Distribution of OTUs (%) in *U. vulgaris* trap samples of different ages														
		medium			young			old							
OTU ID										Domian	Phylum	Class	Order	Family	Genus
4452721	14,4%	0,0%	0,0%	0,8%	0,0%	0,6%	0,7%	25,1%	0,0%	Bacteria	Proteobacteria	Alphaproteobacteria	Rhizobiales	Hyphomicrobiaceae	Devosia
838843	2,3%	2,2%	1,9%	3,6%	2,4%	1,9%	5,4%	1,0%	0,5%	Bacteria	Proteobacteria	Alphaproteobacteria	Rhodospirillales	Acetobacteraceae	Roseomonas
244336	2,3%	2,9%	2,1%	1,4%	1,9%	1,9%	3,4%	0,7%	1,5%	Bacteria	Firmicutes	Bacilli	Bacillales	Paenibacillaceae	Paenibacillus
4479940	1,9%	1,6%	3,8%	2,1%	1,9%	1,0%	1,5%	1,2%	0,8%	Bacteria	Proteobacteria	Gammaproteobacteria	Legionellales	Coxiellaceae	uncultured
509265	0,6%	1,5%	4,7%	3,1%	1,3%	0,9%	1,2%	1,7%	3,3%	Bacteria	Planctomycetes	Planctomycetia	Gemmatales	Gemmataceae	Gemmata
590690	0,4%	1,6%	3,0%	1,9%	2,5%	1,1%	1,1%	0,6%	0,7%	Bacteria	Planctomycetes	Planctomycetia	Gemmatales	Gemmataceae	Gemmata
248442	1,2%	0,6%	1,3%	2,2%	3,4%	0,4%	0,9%	1,3%	0,1%	Bacteria	Proteobacteria	Gammaproteobacteria	Alteromonadales	OM60	Halioglobus
824867	1,2%	1,2%	2,5%	1,6%	1,0%	1,3%	1,6%	0,9%	1,9%	Bacteria	Proteobacteria	Gammaproteobacteria	Legionellales	Coxiellaceae	Aquicella
149938	0,6%	4,0%	0,0%	0,8%	0,1%	0,3%	4,9%	0,6%	0,7%	Bacteria	Proteobacteria	Alphaproteobacteria	Sphingomonadales	Sphingomonadaceae	Sphingomonas
185100	0,4%	3,3%	0,1%	2,2%	0,6%	0,3%	4,1%	0,2%	0,1%	Bacteria	Proteobacteria	Deltaproteobacteria	Bdellovibrionales	Bacteriovoracaceae	Peredibacter
109053	5,7%	0,6%	0,1%	0,4%	0,3%	0,2%	0,7%	0,5%	0,3%	Bacteria	Verrucomicrobia	Opitutae	Opitutales	Opitutaceae	Opitutus
172410	0,5%	0,9%	2,9%	1,2%	1,0%	1,4%	0,5%	0,9%	1,7%	Bacteria	Proteobacteria	Gammaproteobacteria	Xanthomonadales	Xanthomonadaceae	Rhodanobacter
544561	0,2%	0,2%	0,5%	1,3%	4,3%	0,9%	0,3%	0,5%	0,0%	Bacteria	Proteobacteria	Alphaproteobacteria	Rhizobiales	Rhizobiaceae	uncultured
4310715	0,0%	1,5%	0,3%	1,7%	1,9%	3,0%	0,0%	1,0%	4,8%	Bacteria	Bacteroidetes	Cytophagia	Cytophagales	Cytophagaceae	Emticicia
4353982	0,7%	0,9%	1,7%	0,9%	0,7%	1,3%	0,9%	0,5%	0,7%	Bacteria	Verrucomicrobia	[Spartobacteria]	[Chthoniobacterales]	[Chthoniobacteraceae]	Chthoniobacter
4372421	0,2%	1,3%	0,9%	0,7%	2,2%	1,3%	0,2%	0,6%	0,5%	Bacteria	Proteobacteria	Betaproteobacteria	Burkholderiales	Oxalobacteraceae	Massilia
4455330	0,1%	1,0%	0,7%	1,1%	1,2%	2,8%	0,0%	0,6%	2,3%	Bacteria	Proteobacteria	Betaproteobacteria	Burkholderiales	Oxalobacteraceae	beta proteobacterium INB21
373070	0,1%	3,5%	0,0%	0,0%	0,0%	0,1%	1,4%	3,8%	0,0%	Bacteria	Acidobacteria	Holophagae	Holophagales	Holophagaceae	uncultured
4470549	0,5%	0,6%	1,3%	0,5%	0,9%	0,2%	0,9%	0,2%	0,4%	Bacteria	Proteobacteria	Betaproteobacteria	Burkholderiales	Comamonadaceae	Aquincola
4316533	0,2%	2,5%	0,0%	0,1%	0,2%	0,9%	2,1%	0,1%	0,0%	Bacteria	Proteobacteria	Gammaproteobacteria	Methylococcales	Methylococcaceae	Methylomonas
4459446	1,6%	1,5%	0,1%	0,3%	0,2%	0,9%	1,0%	0,2%	0,1%	Bacteria	Actinobacteria	Actinobacteria	Actinomycetales	Mycobacteriaceae	Nocardia
3564333	2,3%	0,2%	0,1%	0,3%	0,2%	0,3%	1,4%	0,0%	0,0%	Bacteria	Verrucomicrobia	Verrucomicrobiae	Verrucomicrobiales	Verrucomicrobiaceae	Luteolibacter
818509	0,4%	0,8%	0,6%	0,2%	1,9%	0,4%	0,2%	0,4%	0,4%	Bacteria	Proteobacteria	Betaproteobacteria	Burkholderiales	Comamonadaceae	uncultured
61192	0,4%	0,4%	0,4%	0,1%	2,0%	0,2%	0,6%	0,0%	0,3%	Bacteria	Proteobacteria	Betaproteobacteria	Burkholderiales	Oxalobacteraceae	Undibacterium
143021	0,4%	0,1%	0,4%	1,4%	1,7%	0,0%	0,4%	0,5%	0,3%	Bacteria	Bacteroidetes	Flavobacteriia	Flavobacteriales	Flavobacteriaceae	Flavobacterium
513803	0,5%	0,4%	1,3%	0,4%	1,2%	0,3%	0,0%	0,0%	0,1%	Bacteria	Proteobacteria	Alphaproteobacteria	Sphingomonadales	Sphingomonadaceae	Novosphingobium
834418	0,2%	1,0%	0,8%	0,6%	0,5%	0,6%	1,1%	0,2%	0,3%	Bacteria	Proteobacteria	Gammaproteobacteria	Xanthomonadales	Sinobacteraceae	uncultured bacterium
4339417	0,3%	1,2%	0,8%	0,6%	0,6%	0,4%	0,6%	0,2%	0,7%	Bacteria	Proteobacteria	Alphaproteobacteria	Rhizobiales	Hyphomicrobiaceae	uncultured
4343749	0,4%	0,5%	1,2%	0,7%	0,7%	0,4%	0,2%	0,6%	0,7%	Bacteria	Verrucomicrobia	[Pedosphaerae]	[Pedosphaerales]	R4-41B	uncultured bacterium
1134668	0,2%	0,6%	0,5%	1,3%	0,6%	1,2%	0,3%	0,4%	1,2%	Bacteria	Proteobacteria	Alphaproteobacteria	Rhodospirillales	Rhodospirillaceae	uncultured bacterium
1134664	3,0%	0,0%	0,0%	0,0%	0,0%	0,0%	0,0%	0,0%	0,0%	Bacteria	Proteobacteria	Betaproteobacteria	Burkholderiales	Comamonadaceae	uncultured
4396041	0,0%	0,1%	0,0%	0,1%	0,0%	0,2%	0,2%	8,3%	0,0%	Bacteria	Bacteroidetes	[Saprospirae]	[Saprospirales]		uncultured
4357677	0,5%	0,4%	0,9%	0,4%	0,2%	0,5%	0,4%	0,4%	0,7%	Bacteria	Bacteroidetes	Sphingobacteriia	Sphingobacteriales	Sphingobacteriaceae	Mucilaginibacter
4454531	0,0%	1,8%	0,1%	0,1%	0,3%	0,1%	1,2%	0,2%	0,0%	Bacteria	Proteobacteria	Gammaproteobacteria	Enterobacteriales	Enterobacteriaceae	Escherichia-Shigella
4393783	0,3%	0,2%	0,4%	0,9%	0,5%	1,9%	0,2%	0,3%	2,1%	Bacteria	Proteobacteria	Betaproteobacteria	Neisseriales	Neisseriaceae	Vogesella
4450676	0,0%	0,2%	1,3%	0,3%	0,5%	0,1%	0,3%	0,4%	0,5%	Bacteria	Proteobacteria	Alphaproteobacteria	Rhizobiales	Hyphomicrobiaceae	uncultured
2209903	0,3%	0,3%	0,7%	0,8%	0,5%	0,3%	0,3%	0,2%	0,3%	Bacteria	Proteobacteria	Betaproteobacteria	Burkholderiales	Oxalobacteraceae	Variovorax
807452	0,4%	0,1%	1,1%	0,1%	0,2%	0,2%	0,2%	0,9%	1,1%	Bacteria	Proteobacteria	Alphaproteobacteria	Rhodobacterales	Rhodobacteraceae	Rhodobacter
853114	0,3%	0,2%	0,3%	0,7%	0,2%	0,7%	0,6%	0,6%	1,3%	Bacteria	Bacteroidetes	[Saprospirae]	[Saprospirales]	Chitinophagaceae	uncultured
4441442	0,0%	0,2%	0,4%	0,3%	1,2%	0,0%	0,4%	0,1%	0,0%	Bacteria	Proteobacteria	Betaproteobacteria	Burkholderiales	Comamonadaceae	uncultured
4437283	0,1%	0,2%	0,1%	0,7%	1,0%	0,0%	0,5%	0,2%	0,0%	Bacteria	Proteobacteria	Deltaproteobacteria	Desulfuromonadales	Desulfuromonadaceae	Geothermobacter
3239359	1,9%	0,1%	0,1%	0,2%	0,0%	0,1%	0,1%	0,2%	0,4%	Bacteria	Proteobacteria	Gammaproteobacteria	Methylococcales	Crenotrichaceae	Methylovulum
832784	0,1%	0,2%	0,3%	0,1%	1,1%	0,2%	0,4%	0,1%	0,1%	Bacteria	Proteobacteria	Gammaproteobacteria	Aeromonadales	Aeromonadaceae	uncultured bacterium
818411	1,3%	0,1%	0,0%	0,1%	0,0%	0,0%	0,0%	2,9%	0,0%	Bacteria	Proteobacteria	Alphaproteobacteria	Rhodospirillales	Rhodospirillaceae	Dongia
172172	0,2%	0,2%	0,8%	0,1%	0,2%	0,3%	0,8%	0,1%	0,1%	Bacteria	Proteobacteria	Gammaproteobacteria	Legionellales	Coxiellaceae	Coxiella
822006	0,2%	0,1%	0,0%	0,1%	0,1%	0,0%	1,8%	0,0%	0,1%	Bacteria	Planctomycetes	Phycisphaerae	WD2101		uncultured bacterium
575993	0,1%	0,6%	0,5%	0,3%	0,6%	0,8%	0,2%	0,0%	0,0%	Bacteria	Proteobacteria	Deltaproteobacteria	Spirobacillales		uncultured bacterium
816128	0,1%	0,7%	0,4%	0,4%	0,8%	0,1%	0,0%	0,2%	0,3%	Bacteria	Proteobacteria	Betaproteobacteria	Methylophilales	Methylophilaceae	uncultured
4300692	1,0%	0,0%	0,0%	0,1%	0,1%	0,0%	1,0%	0,1%	0,1%	Bacteria	Verrucomicrobia	[Pedosphaerae]	[Pedosphaerales]	Ellin515	uncultured Verrucomicrobia bacterium
894047	0,1%	0,3%	0,3%	0,8%	0,4%	1,4%	0,1%	0,1%	0,0%	Bacteria	Actinobacteria	Acidimicrobiia	Acidimicrobiales	C111	CL500-29 marine group
811424	0,0%	0,5%	0,4%	0,2%	0,7%	0,1%	0,5%	0,0%	0,0%	Bacteria	Verrucomicrobia	Opitutae	Opitutales	Opitutaceae	Opitutus
688259	0,8%	0,2%	0,4%	0,2%	0,1%	0,6%	0,0%	1,1%	0,5%	Bacteria	Actinobacteria	Actinobacteria	Actinomycetales	Micromonosporaceae	Actinoplanes
227239	0,0%	1,4%	0,0%	0,7%	0,2%	0,0%	0,0%	2,0%	0,5%	Bacteria	Proteobacteria	Alphaproteobacteria	Rhodospirillales	Rhodospirillaceae	uncultured
830322	1,4%	0,1%	0,2%	0,0%	0,0%	0,0%	0,3%	0,1%	0,1%	Bacteria	Acidobacteria	Acidobacteriia	Acidobacteriales	Acidobacteriaceae	Acidicapsa
499887	0,1%	0,3%	0,1%	1,1%	0,4%	1,1%	0,0%	0,4%	2,4%	Bacteria	Proteobacteria	Alphaproteobacteria	Rhizobiales	Rhizobiaceae	Kaistia
540856	0,0%	0,1%	0,2%	0,6%	0,7%	0,0%	0,5%	0,2%	0,0%	Bacteria	Firmicutes	Clostridia	Clostridiales	Clostridiaceae	Clostridium sensu stricto 1
348570	0,7%	0,3%	0,1%	0,3%	0,1%	0,3%	0,6%	0,2%	0,3%	Bacteria	Proteobacteria	Alphaproteobacteria	Rhodospirillales	Acetobacteraceae	uncultured
101542	0,6%	0,1%	0,6%	0,2%	0,1%	0,1%	0,2%	0,2%	0,7%	Bacteria	Proteobacteria	Alphaproteobacteria	Rhizobiales		uncultured
4471434	0,2%	0,5%	0,4%	0,3%	0,2%	0,2%	0,4%	0,1%	0,3%	Bacteria	Verrucomicrobia	Verrucomicrobiae	Verrucomicrobiales	Verrucomicrobiaceae	uncultured
4302249	0,0%	0,5%	0,3%	0,6%	0,5%	0,2%	0,2%	0,5%	0,3%	Bacteria	Chloroflexi	Ktedonobacteria	Thermogemmatisporales	Thermogemmatisporaceae	uncultured Ktedonobacter sp.
3364423	0,1%	0,6%	0,2%	0,3%	0,4%	0,5%	0,3%	0,1%	0,1%	Bacteria	Proteobacteria	Betaproteobacteria	SBla14		Sideroxydans
632342	0,0%	0,0%	1,6%	0,0%	0,1%	0,0%	0,0%	0,0%	0,0%	Bacteria	Proteobacteria	Alphaproteobacteria	Rickettsiales		Candidatus Odyssella
2792167	0,0%	0,7%	0,2%	0,1%	0,3%	0,1%	0,6%	0,1%	0,1%	Bacteria	Bacteroidetes	[Saprospirae]	[Saprospirales]	Chitinophagaceae	Sediminibacterium
1062356	0,4%	0,4%	0,3%	0,3%	0,3%	0,1%	0,2%	0,2%	0,3%	Bacteria	Actinobacteria	Actinobacteria	Actinomycetales	Corynebacteriaceae	Corynebacterium
3271869	0,0%	0,4%	0,4%	0,4%	0,3%	0,0%	0,3%	0,1%	0,1%	Bacteria	Proteobacteria	Alphaproteobacteria	Rhodobacterales	Rhodobacteraceae	Amaricoccus
4343367	0,1%	0,4%	0,9%	0,0%	0,1%	0,1%	0,1%	0,0%	0,1%	Bacteria	Actinobacteria	Actinobacteria	Actinomycetales	ACK-M1	hgcI clade
4482563	0,1%	0,1%	0,5%	0,2%	0,4%	0,3%	0,1%	0,3%	0,4%	Bacteria	Planctomycetes	Planctomycetia	Gemmatales	Gemmataceae	uncultured
4343369	0,0%	0,1%	0,5%	0,4%	0,2%	0,3%	0,4%	0,2%	0,1%	Bacteria	Proteobacteria	Betaproteobacteria	Methylophilales	Methylophilaceae	OM43 clade
4424705	0,1%	0,3%	0,6%	0,2%	0,3%	0,5%	0,0%	0,3%	0,5%	Bacteria	Proteobacteria	Betaproteobacteria	Burkholderiales	Comamonadaceae	Curvibacter
4357886	0,1%	0,4%	0,6%	0,1%	0,3%	0,0%	0,1%	0,1%	0,0%	Bacteria	Proteobacteria	Alphaproteobacteria	Rhizobiales	Methylocystaceae	uncultured
4357887	0,2%	0,3%	0,1%	0,3%	0,3%	0,2%	0,4%	0,0%	0,8%	Bacteria	Proteobacteria	Alphaproteobacteria	Rhizobiales		alpha proteobacterium SK200a-2
4315164	0,0%	0,2%	1,0%	0,1%	0,1%	0,1%	0,1%	0,2%	0,1%	Bacteria	Proteobacteria	Betaproteobacteria	Burkholderiales	Comamonadaceae	uncultured
826904	0,0%	0,2%	0,6%	0,2%	0,2%	0,2%	0,1%	0,2%	0,4%	Bacteria	Proteobacteria	Alphaproteobacteria	Rhizobiales		Devosia
820575	0,6%	0,2%	0,0%	0,1%	0,3%	0,5%	0,3%	0,0%	0,0%	Bacteria	Actinobacteria	Actinobacteria	Actinomycetales	Williamsiaceae	Williamsia
152605	0,0%	0,1%	0,0%	0,6%	0,5%	0,2%	0,4%	0,3%	0,4%	Bacteria	Proteobacteria	Alphaproteobacteria	Rhodospirillales	Acetobacteraceae	Acidisoma
4325231	0,2%	0,0%	0,0%	0,4%	0,2%	0,3%	0,7%	0,2%	0,1%	Bacteria	Acidobacteria	Acidobacteriia	Acidobacteriales	Acidobacteriaceae	Telmatobacter
909097	0,2%	0,2%	0,4%	0,3%	0,3%	0,1%	0,1%	0,1%	0,1%	Bacteria	Proteobacteria	Alphaproteobacteria	Rhodospirillales	Acetobacteraceae	Roseomonas
4438548	0,4%	0,3%	0,3%	0,3%	0,1%	0,3%	0,2%	0,2%	0,1%	Bacteria	Bacteroidetes	Flavobacteriia	Flavobacteriales	Flavobacteriaceae	Flavobacterium
4325237	0,2%	0,3%	0,4%	0,2%	0,1%	0,1%	0,2%	0,2%	0,8%	Bacteria	Proteobacteria	Gammaproteobacteria	Xanthomonadales	Xanthomonadaceae	uncultured
4423750	0,4%	0,2%	0,1%	0,3%	0,2%	0,3%	0,2%	0,4%	0,5%	Bacteria	Bacteroidetes	[Saprospirae]	[Saprospirales]	Chitinophagaceae	Sediminibacterium
790527	0,1%	0,3%	0,3%	0,2%	0,3%	1,0%	0,0%	0,2%	0,5%	Bacteria	Armatimonadetes	Armatimonadia	FW68		uncultured bacterium
1119668	0,0%	0,3%	0,4%	0,2%	0,1%	0,2%	0,4%	0,1%	0,1%	Bacteria	Proteobacteria	Alphaproteobacteria	Rhizobiales	Hyphomicrobiaceae	Devosia
236725	0,1%	0,3%	0,1%	0,2%	0,3%	1,1%	0,3%	0,1%	0,1%	Bacteria	Proteobacteria	Gammaproteobacteria	Chromatiales		uncultured
543327	0,6%	0,0%	0,3%	0,2%	0,1%	0,2%	0,1%	0,1%	0,4%	Bacteria	Verrucomicrobia	[Pedosphaerae]	[Pedosphaerales]	auto67_4W	uncultured Verrucomicrobia bacterium
1133478	0,1%	0,4%	0,3%	0,1%	0,2%	0,2%	0,4%	0,1%	0,1%	Bacteria	Actinobacteria	Actinobacteria	Actinomycetales	Micromonosporaceae	Actinoplanes
821602	0,0%	0,1%	0,6%	0,2%	0,2%	0,1%	0,3%	0,3%	0,0%	Bacteria	Verrucomicrobia	Opitutae	[Cerasicoccales]	[Cerasicoccaceae]	uncultured bacterium
4434799	1,3%	0,0%	0,0%	0,0%	0,0%	0,0%	0,0%	0,0%	0,0%	Bacteria	Proteobacteria	Alphaproteobacteria	Rhizobiales	Methylobacteriaceae	Methylobacterium
517939	0,1%	0,3%	0,2%	0,2%	0,5%	0,3%	0,0%	0,2%	0,0%	Bacteria	Proteobacteria	Gammaproteobacteria	Xanthomonadales	Sinobacteraceae	uncultured bacterium
4302412	1,0%	0,1%	0,0%	0,1%	0,0%	0,3%	0,0%	0,2%	0,5%	Bacteria	Proteobacteria	Betaproteobacteria	SBla14		Sideroxydans
1640195	0,1%	0,3%	0,2%	0,1%	0,2%	0,5%	0,4%	0,1%	0,0%	Bacteria	Proteobacteria	Alphaproteobacteria	Rhodospirillales	Rhodospirillaceae	Reyranella
2400244	0,0%	0,3%	0,3%	0,3%	0,3%	0,2%	0,0%	0,6%	0,4%	Bacteria	Proteobacteria	Gammaproteobacteria	Methylococcales	Crenotrichaceae	Crenothrix
113404	0,0%	0,2%	0,4%	0,2%	0,2%	0,1%	0,4%	0,0%	0,0%	Bacteria	Acidobacteria	Acidobacteria-6	iii1-15		uncultured Acidobacteriales bacterium
4467678	0,0%	0,0%	0,1%	0,2%	0,6%	0,0%	0,3%	0,0%	0,0%	Bacteria	Proteobacteria	Alphaproteobacteria	Sphingomonadales	Sphingomonadaceae	Novosphingobium
4318356	1,1%	0,1%	0,0%	0,0%	0,0%	0,0%	0,0%	0,0%	0,0%	Bacteria	Verrucomicrobia	[Spartobacteria]	[Chthoniobacterales]	[Chthoniobacteraceae]	Chthoniobacter
112133	0,2%	0,4%	0,1%	0,3%	0,2%	0,7%	0,0%	0,3%	0,4%	Bacteria	Armatimonadetes	Armatimonadia	Armatimonadales	Armatimonadaceae	Armatimonas
841487	0,0%	0,1%	0,7%	0,2%	0,1%	0,5%	0,0%	0,0%	0,1%	Bacteria	Planctomycetes	Planctomycetia	Gemmatales	Gemmataceae	uncultured
2011775	0,0%	0,1%	0,2%	0,0%	0,0%	0,2%	0,8%	0,0%	0,0%	Bacteria	Planctomycetes	Planctomycetia	Gemmatales	Gemmataceae	uncultured
29360	0,0%	0,3%	0,2%	0,2%	0,3%	0,3%	0,2%	0,1%	0,0%	Bacteria	Proteobacteria	Alphaproteobacteria	Rhodospirillales	Acetobacteraceae	Rhodovastum
4389007	0,8%	0,1%	0,1%	0,1%	0,1%	0,1%	0,0%	0,1%	0,0%	Bacteria	Verrucomicrobia	Verrucomicrobiae	Verrucomicrobiales	Verrucomicrobiaceae	uncultured
611360	0,5%	0,1%	0,1%	0,0%	0,0%	1,6%	0,0%	0,0%	0,0%	Bacteria	Proteobacteria	Betaproteobacteria	Burkholderiales	Comamonadaceae	Acidovorax
844535	0,2%	0,1%	0,1%	0,2%	0,3%	0,3%	0,2%	0,2%	0,4%	Bacteria	Proteobacteria	Alphaproteobacteria	Caulobacterales	Caulobacteraceae	Asticcacaulis
4318801	0,3%	0,2%	0,2%	0,1%	0,1%	0,0%	0,2%	0,1%	0,0%	Bacteria	Proteobacteria	Alphaproteobacteria	Sphingomonadales	Sphingomonadaceae	Sphingomonas
4477127	0,3%	0,0%	0,1%	0,7%	0,2%	0,2%	0,0%	0,2%	0,1%	Bacteria	Proteobacteria	Deltaproteobacteria	Bdellovibrionales	Bdellovibrionaceae	Bdellovibrio
4431277	0,0%	0,4%	0,0%	0,1%	0,2%	0,2%	0,5%	0,1%	0,0%	Bacteria	Proteobacteria	Alphaproteobacteria	Caulobacterales	Caulobacteraceae	uncultured
810360	0,0%	0,1%	0,0%	0,0%	0,0%	0,0%	1,0%	0,0%	0,0%	Bacteria	Verrucomicrobia	[Pedosphaerae]	[Pedosphaerales]	auto67_4W	uncultured Verrucomicrobia bacterium
1624	0,2%	0,2%	0,2%	0,2%	0,2%	0,0%	0,3%	0,1%	0,1%	Bacteria	Verrucomicrobia	[Spartobacteria]	[Chthoniobacterales]	[Chthoniobacteraceae]	uncultured bacterium
571199	0,1%	0,0%	0,0%	0,0%	0,8%	0,2%	0,0%	0,0%	0,0%	Bacteria	Proteobacteria	Deltaproteobacteria	Myxococcales	OM27	OM27 clade
809256	0,0%	0,4%	0,3%	0,3%	0,3%	0,0%	0,1%	0,1%	0,0%	Bacteria	Verrucomicrobia	Opitutae	Opitutales	Opitutaceae	Opitutus
4425571	0,0%	0,0%	0,1%	0,3%	0,5%	0,0%	0,1%	0,1%	0,1%	Bacteria	Proteobacteria	Gammaproteobacteria	Enterobacteriales	Enterobacteriaceae	Escherichia-Shigella
331407	0,0%	0,0%	0,5%	0,8%	0,0%	0,2%	0,0%	0,0%	0,4%	Bacteria	Proteobacteria	Betaproteobacteria	Procabacteriales	Procabacteriaceae	uncultured
4445991	0,4%	0,3%	0,0%	0,2%	0,3%	0,2%	0,0%	0,0%	0,1%	Bacteria	Proteobacteria	Betaproteobacteria	Rhodocyclales	Rhodocyclaceae	Uliginosibacterium
4326874	0,0%	0,3%	0,1%	0,0%	0,3%	0,5%	0,2%	0,0%	0,0%	Bacteria	Proteobacteria	Betaproteobacteria	Burkholderiales	Comamonadaceae	Acidovorax
811148	0,1%	0,1%	0,2%	0,1%	0,3%	0,5%	0,2%	0,0%	0,0%	Bacteria	TM6	SJA-4			uncultured bacterium
524860	0,1%	0,1%	0,5%	0,2%	0,1%	0,1%	0,2%	0,1%	0,3%	Bacteria	Planctomycetes	Planctomycetia	Gemmatales	Gemmataceae	Gemmata
4442504	0,0%	0,2%	0,1%	0,3%	0,3%	0,1%	0,3%	0,1%	0,1%	Bacteria	Actinobacteria	Actinobacteria	Actinomycetales	Microbacteriaceae	Arthrobacter
575450	0,5%	0,2%	0,1%	0,0%	0,1%	0,6%	0,0%	0,0%	0,0%	Bacteria	Proteobacteria	Gammaproteobacteria	Legionellales		uncultured bacterium
4470542	0,0%	0,1%	0,3%	0,1%	0,3%	0,0%	0,3%	0,1%	0,0%	Bacteria	Proteobacteria	Alphaproteobacteria	Rhizobiales	Hyphomicrobiaceae	Hyphomicrobium
826124	0,1%	0,2%	0,1%	0,2%	0,2%	0,2%	0,3%	0,1%	0,1%	Bacteria	Proteobacteria	Alphaproteobacteria	Caulobacterales	Caulobacteraceae	Phenylobacterium
2651333	0,0%	0,2%	0,5%	0,2%	0,1%	0,2%	0,0%	0,3%	0,3%	Bacteria	Actinobacteria	Actinobacteria	Actinomycetales	Mycobacteriaceae	Nocardia
160071	0,7%	0,0%	0,0%	0,0%	0,0%	0,4%	0,0%	0,4%	0,0%	Bacteria	Proteobacteria	Alphaproteobacteria	Rhodospirillales	Rhodospirillaceae	uncultured Hyphomicrobiaceae bacterium
4321043	0,0%	0,0%	0,0%	0,0%	0,0%	2,7%	0,0%	0,0%	0,3%	Bacteria	Actinobacteria	Acidimicrobiia	Acidimicrobiales	C111	Ilumatobacter
3460758	0,3%	0,1%	0,0%	0,1%	0,1%	0,6%	0,2%	0,0%	0,1%	Bacteria	Acidobacteria	Acidobacteriia	Acidobacteriales	Acidobacteriaceae	Acidobacterium
4424553	0,3%	0,1%	0,1%	0,1%	0,1%	0,0%	0,3%	0,1%	0,0%	Bacteria	Proteobacteria	Alphaproteobacteria	Rhizobiales	Hyphomicrobiaceae	Rhodomicrobium
4469416	0,0%	0,4%	0,0%	0,1%	0,0%	1,1%	0,0%	0,0%	2,4%	Bacteria	Proteobacteria	Alphaproteobacteria	Rhodobacterales	Rhodobacteraceae	Rubellimicrobium
4479752	0,1%	0,1%	0,1%	0,3%	0,2%	0,7%	0,0%	0,0%	0,1%	Bacteria	Proteobacteria	Alphaproteobacteria	Rhizobiales	Hyphomicrobiaceae	Hyphomicrobium
37958	0,9%	0,0%	0,0%	0,0%	0,0%	0,0%	0,0%	0,0%	0,0%	Bacteria	Proteobacteria	Betaproteobacteria	Burkholderiales	Alcaligenaceae	Achromobacter
4413214	0,3%	0,0%	0,1%	0,1%	0,3%	0,1%	0,0%	0,3%	0,1%	Bacteria	Proteobacteria	Alphaproteobacteria	Sphingomonadales	Sphingomonadaceae	Novosphingobium
4401025	0,0%	0,1%	0,1%	0,1%	0,1%	0,2%	0,4%	0,0%	0,0%	Bacteria	Acidobacteria	Acidobacteriia	Acidobacteriales	Acidobacteriaceae	uncultured bacterium
136119	0,0%	0,3%	0,2%	0,1%	0,1%	0,0%	0,3%	0,0%	0,1%	Bacteria	Proteobacteria	Deltaproteobacteria	Myxococcales		uncultured bacterium
4017244	0,0%	0,1%	0,6%	0,0%	0,0%	0,0%	0,1%	0,1%	0,0%	Bacteria	Spirochaetes	[Leptospirae]	[Leptospirales]	Leptospiraceae	Turneriella
814647	0,6%	0,0%	0,1%	0,0%	0,0%	0,0%	0,0%	0,2%	0,7%	Bacteria	Bacteroidetes	Sphingobacteriia	Sphingobacteriales		uncultured bacterium
4428544	0,0%	0,1%	0,3%	0,2%	0,1%	0,1%	0,3%	0,0%	0,0%	Bacteria	Firmicutes	Clostridia	Clostridiales	Lachnospiraceae	uncultured
541789	0,0%	0,1%	0,0%	0,4%	0,3%	0,5%	0,0%	0,2%	0,3%	Bacteria	Verrucomicrobia	[Spartobacteria]	[Chthoniobacterales]	[Chthoniobacteraceae]	Chthoniobacter
726730	0,3%	0,0%	0,1%	0,1%	0,1%	0,0%	0,3%	0,1%	0,0%	Bacteria	Actinobacteria	Actinobacteria	Actinomycetales	Microbacteriaceae	Candidatus Aquiluna
4477307	0,0%	0,3%	0,3%	0,1%	0,1%	0,1%	0,1%	0,0%	0,1%	Bacteria	Verrucomicrobia	[Pedosphaerae]	[Pedosphaerales]	auto67_4W	uncultured bacterium
520587	0,0%	0,1%	0,1%	1,1%	0,1%	0,0%	0,0%	0,1%	0,4%	Bacteria	Acidobacteria	DA052	Ellin6513		uncultured Acidobacteria bacterium
944968	0,1%	0,2%	0,1%	0,2%	0,1%	0,0%	0,3%	0,0%	0,3%	Bacteria	Proteobacteria	Gammaproteobacteria	Methylococcales	Crenotrichaceae	uncultured
161616	0,3%	0,1%	0,1%	0,2%	0,1%	0,0%	0,2%	0,0%	0,0%	Bacteria	Proteobacteria	Betaproteobacteria	Burkholderiales	Comamonadaceae	Leptothrix
4377842	0,3%	0,1%	0,0%	0,1%	0,1%	0,0%	0,1%	0,2%	1,3%	Bacteria	Proteobacteria	Betaproteobacteria	Burkholderiales	Comamonadaceae	Variovorax
3078471	0,0%	0,1%	0,2%	0,2%	0,3%	0,0%	0,1%	0,0%	0,0%	Bacteria	Proteobacteria	Alphaproteobacteria	Rhizobiales	Hyphomicrobiaceae	Hyphomicrobium
332574	0,0%	0,3%	0,2%	0,2%	0,2%	0,0%	0,1%	0,2%	0,0%	Bacteria	Proteobacteria	Gammaproteobacteria	Xanthomonadales	Sinobacteraceae	uncultured bacterium
4323373	0,0%	0,1%	0,1%	0,2%	0,2%	0,4%	0,2%	0,2%	0,3%	Bacteria	Planctomycetes	Planctomycetia	Pirellulales	Pirellulaceae	uncultured
296086	0,5%	0,0%	0,0%	0,1%	0,1%	0,1%	0,1%	0,1%	0,0%	Bacteria	Actinobacteria	Thermoleophilia	Solirubrobacterales		uncultured bacterium
4381918	0,1%	0,2%	0,3%	0,2%	0,0%	0,1%	0,2%	0,1%	0,1%	Bacteria	Chloroflexi	Anaerolineae	envOPS12		uncultured
2281139	0,7%	0,0%	0,0%	0,0%	0,0%	0,0%	0,0%	0,2%	0,0%	Bacteria	Planctomycetes	Planctomycetia	Pirellulales	Pirellulaceae	Pirellula
4369502	0,1%	0,1%	0,1%	0,2%	0,2%	0,1%	0,3%	0,0%	0,1%	Bacteria	Verrucomicrobia	[Pedosphaerae]	[Pedosphaerales]	auto67_4W	uncultured bacterium
4421180	0,0%	0,2%	0,1%	0,2%	0,1%	0,0%	0,2%	0,1%	0,0%	Bacteria	Proteobacteria	Alphaproteobacteria	Rhizobiales	Aurantimonadaceae	Rhizobium
4369504	0,0%	0,3%	0,0%	0,2%	0,3%	0,0%	0,0%	0,3%	0,4%	Bacteria	Acidobacteria	Acidobacteriia	Acidobacteriales	Koribacteraceae	uncultured
1584969	0,0%	0,3%	0,1%	0,3%	0,2%	0,0%	0,1%	0,2%	0,1%	Bacteria	Proteobacteria	Alphaproteobacteria	Rhizobiales	Beijerinckiaceae	Methylorosula
1584967	0,0%	0,2%	0,2%	0,3%	0,2%	0,2%	0,1%	0,1%	0,1%	Bacteria	Proteobacteria	Gammaproteobacteria	Xanthomonadales	Xanthomonadaceae	Rhodanobacter
4409730	0,1%	0,4%	0,0%	0,0%	0,1%	0,1%	0,4%	0,0%	0,1%	Bacteria	Firmicutes	Clostridia	Clostridiales	Peptostreptococcaceae	Incertae Sedis
852722	0,4%	0,2%	0,1%	0,1%	0,1%	0,0%	0,0%	0,0%	0,0%	Bacteria	Proteobacteria	Deltaproteobacteria	Spirobacillales		uncultured bacterium
4349607	0,2%	0,1%	0,2%	0,2%	0,0%	0,0%	0,1%	0,1%	0,1%	Bacteria	Proteobacteria	Deltaproteobacteria	Myxococcales		uncultured soil bacterium
4452696	0,2%	0,4%	0,0%	0,0%	0,2%	0,0%	0,0%	0,1%	0,0%	Bacteria	Planctomycetes	Planctomycetia	Gemmatales	Gemmataceae	uncultured
1008207	0,2%	0,1%	0,0%	0,3%	0,1%	0,4%	0,1%	0,1%	0,0%	Bacteria	Proteobacteria	Deltaproteobacteria	Myxococcales		Sorangium
992035	0,0%	0,1%	0,2%	0,2%	0,2%	0,4%	0,1%	0,2%	0,0%	Bacteria	Proteobacteria	Betaproteobacteria	Burkholderiales	Oxalobacteraceae	Massilia
680509	0,0%	0,1%	0,1%	0,2%	0,1%	0,1%	0,2%	0,2%	0,7%	Bacteria	Proteobacteria	Betaproteobacteria	Rhodocyclales	Rhodocyclaceae	Uliginosibacterium
4243758	0,0%	0,0%	0,1%	0,2%	0,0%	0,1%	0,4%	0,2%	0,1%	Bacteria	Proteobacteria	Alphaproteobacteria	BD7-3		uncultured
4385637	0,4%	0,1%	0,1%	0,1%	0,0%	0,2%	0,0%	0,0%	0,7%	Bacteria	Proteobacteria	Alphaproteobacteria	Rhodospirillales	Rhodospirillaceae	uncultured
812105	0,0%	0,1%	0,2%	0,1%	0,1%	0,0%	0,3%	0,0%	0,1%	Bacteria	WS3	PRR-12	Sediment-1	PRR-10	uncultured bacterium
4310013	0,0%	0,0%	0,1%	0,0%	0,4%	0,0%	0,1%	0,1%	0,0%	Bacteria	Actinobacteria	Thermoleophilia	Gaiellales	Gaiellaceae	uncultured bacterium
1140475	0,1%	0,2%	0,0%	0,1%	0,1%	0,2%	0,1%	0,3%	0,4%	Bacteria	Proteobacteria	Alphaproteobacteria	Rhodospirillales	Rhodospirillaceae	uncultured bacterium

OTU ID	cellulolytic	nitarte reducers	nitarte reducers	nitrate assimilation	nitrite oxidizers	denirifiers	denirifiers	denirifiers	denirifiers	N2 fixators	N2 fixators	bactera capable of DNRA	urea degraders	sulphate reducers	sulphate reducers	sulfide oxidizers	ammonia oxidizers	chitin degraders	methanotrophs	methanotrophs	polyphenol degraders	polyphenol degraders	lignin degraders	lignin degraders	lignin degraders
4452721	-	napA	-	-	-	-	nirK	-	-	nifH	-	-	ureA	-	-	-	-	chb	-	-	laccase_Asco	laccase_Basidio	-	-	ppo
838843	-	napA	-	-	-	-	-	-	-	-	nifD	-	ureA	-	-	-	-	-	-	-	-	laccase_Basidio	-	-	-
244336	-	napA	narG	-	-	nirS	nirK	norB	nosZ	nifH	nifD	-	ureA	-	-	-	-	-	-	-	laccase_Asco	laccase_Basidio	-	-	ppo
4479940	-	-	-	-	-	-	-	-	-	-	-	-	-	-	-	-	-	-	-	-	-	-	-	lip	-
509265	-	-	-	-	-	-	-	-	-	-	-	-	-	-	-	-	-	-	-	-	-	-	-	-	-
590690	-	-	-	-	-	-	-	-	-	-	-	-	-	-	-	-	-	-	-	-	-	-	-	-	-
248442	-	-	-	-	-	-	-	-	-	-	-	-	-	-	-	-	-	-	-	-	-	-	-	-	-
824867	-	-	-	-	-	-	-	-	-	-	-	-	-	-	-	-	-	-	-	-	-	-	-	-	-
149938	-	napA	narG	nirA	-	-	nirK	norB	-	nifH	nifD	-	ureA	-	-	-	-	chb	mmoX	-	laccase_Asco	laccase_Basidio	-	-	ppo
185100	-	-	-	-	-	-	-	-	-	-	-	-	-	-	-	-	-	-	-	-	-	-	-	-	-
109053	-	napA	-	nirA	-	-	-	-	nosZ	-	-	nrfA	ureA	-	-	-	-	-	-	-	-	laccase_Basidio	-	-	-
172410	cellulolytic	napA	narG	-	-	nirS	nirK	norB	nosZ	-	-	-	-	-	-	-	-	-	mmoX	-	-	laccase_Basidio	-	-	ppo
544561	-	-	-	-	-	-	-	-	-	-	-	-	-	-	-	-	-	-	-	-	-	-	-	lip	-
4310715	cellulolytic	-	-	nirA	-	-	-	-	-	-	-	-	-	-	-	-	-	chb	-	-	laccase_Asco	laccase_Basidio	-	-	ppo
4353982	-	napA	-	-	-	-	nirK	-	-	-	-	nrfA	ureA	-	-	-	-	-	-	-	-	-	-	-	-
4372421	-	napA	narG	-	-	-	-	norB	nosZ	-	-	-	ureA	-	-	-	-	chb	mmoX	-	laccase_Asco	laccase_Basidio	-	-	ppo
4455330	-	-	-	-	-	-	-	-	-	-	-	-	-	-	-	-	-	-	-	-	-	-	-	-	-
373070	-	-	-	-	-	-	-	-	-	-	-	-	-	-	-	-	-	-	-	-	-	-	-	lip	-
4470549	-	-	-	-	-	-	-	-	-	-	-	-	-	-	-	-	-	-	-	-	-	-	-	-	-
4316533	-	napA	-	nirA	-	nirS	nirK	norB	-	nifH	nifD	-	ureA	-	-	-	amoA_AOB	-	mmoX	pmoA	laccase_Asco	laccase_Basidio	-	-	ppo
4459446	-	napA	narG	-	-	-	nirK	-	-	-	-	-	ureA	-	-	-	-	-	mmoX	-	laccase_Asco	laccase_Basidio	-	-	ppo
3564333	-	-	-	-	-	-	-	-	-	-	-	-	-	-	-	-	-	-	-	-	-	-	-	-	-
818509	-	-	-	-	-	-	-	-	-	-	-	-	-	-	-	-	-	-	-	-	-	-	-	lip	-
61192	-	-	-	-	-	-	-	-	-	-	-	-	-	-	-	-	-	-	-	-	-	-	-	-	-
143021	cellulolytic	napA	-	nirA	-	-	nirK	norB	nosZ	-	-	-	ureA	-	-	-	-	-	-	-	laccase_Asco	laccase_Basidio	-	-	ppo
513803	-	napA	-	-	-	-	-	norB	-	nifH	nifD	-	ureA	-	-	-	-	-	mmoX	-	laccase_Asco	laccase_Basidio	-	-	ppo
834418	-	-	-	-	-	-	-	-	-	-	-	-	-	-	-	-	-	-	-	-	-	-	-	-	-
4339417	-	-	-	-	-	-	-	-	-	-	-	-	-	-	-	-	-	-	-	-	-	-	-	lip	-
4343749	-	-	-	-	-	-	-	-	-	-	-	-	-	-	-	-	-	-	-	-	-	-	-	-	-
1134668	-	-	-	-	-	-	-	-	-	-	-	-	-	-	-	-	-	-	-	-	-	-	-	-	-
1134664	-	-	-	-	-	-	-	-	-	-	-	-	-	-	-	-	-	-	-	-	-	-	-	lip	-
4396041	-	-	-	-	-	-	-	-	-	-	-	-	-	-	-	-	-	-	-	-	-	-	-	lip	-
4357677	-	-	-	-	-	-	-	-	-	-	-	-	-	-	-	-	-	-	-	-	-	-	-	-	-
4454531	cellulolytic	-	-	-	-	-	-	-	-	-	-	-	-	-	-	-	-	-	-	-	-	-	-	-	-
4393783	-	napA	narG	-	-	nirS	-	-	-	-	-	-	-	-	-	-	-	-	-	-	laccase_Asco	laccase_Basidio	-	-	ppo
4450676	-	-	-	-	-	-	-	-	-	-	-	-	-	-	-	-	-	-	-	-	-	-	-	lip	-
2209903	-	napA	narG	nirA	-	-	-	norB	-	-	-	-	ureA	-	-	-	-	-	mmoX	-	-	-	-	-	-
807452	-	napA	-	nirA	-	nirS	nirK	norB	nosZ	nifH	nifD	-	ureA	-	-	SoxB	-	-	mmoX	-	laccase_Asco	laccase_Basidio	-	-	ppo
853114	-	-	-	-	-	-	-	-	-	-	-	-	-	-	-	-	-	-	-	-	-	-	-	lip	-
4441442	-	-	-	-	-	-	-	-	-	-	-	-	-	-	-	-	-	-	-	-	-	-	-	lip	-
4437283	-	-	-	-	-	-	-	-	-	-	nifD	-	-	-	-	-	-	-	-	-	-	-	-	-	-
3239359	-	-	-	-	-	-	-	-	-	nifH	-	-	-	-	-	-	-	-	mmoX	pmoA	-	-	-	-	-
832784	-	-	-	-	-	-	-	-	-	-	-	-	-	-	-	-	-	-	-	-	-	-	-	-	-
818411	-	-	-	-	-	-	-	-	-	-	-	-	-	-	-	-	-	-	-	-	-	-	-	-	-
172172	-	-	-	-	-	-	-	-	-	-	-	-	-	dsrA	dsrB	-	-	-	-	-	laccase_Asco	laccase_Basidio	-	-	ppo
822006	-	-	-	-	-	-	-	-	-	-	-	-	-	-	-	-	-	-	-	-	-	-	-	-	-
575993	-	-	-	-	-	-	-	-	-	-	-	-	-	-	-	-	-	-	-	-	-	-	-	-	-
816128	-	-	-	-	-	-	-	-	-	-	-	-	-	-	-	-	-	-	-	-	-	-	-	lip	-
4300692	-	-	-	-	-	-	-	-	-	-	-	-	-	-	-	-	-	-	-	-	-	-	-	-	-
894047	-	-	-	-	-	-	-	-	-	-	-	-	-	-	-	-	-	-	-	-	-	-	-	-	-
811424	-	napA	-	nirA	-	-	-	-	nosZ	-	-	nrfA	ureA	-	-	-	-	-	-	-	-	laccase_Basidio	-	-	-
688259	cellulolytic	napA	narG	-	-	-	nirK	-	-	-	-	-	ureA	-	-	-	-	-	-	-	laccase_Asco	laccase_Basidio	-	-	ppo
227239	-	-	-	-	-	-	-	-	-	-	-	-	-	-	-	-	-	-	-	-	-	-	-	lip	-
830322	cellulolytic	-	-	-	-	-	-	-	-	-	-	-	-	-	-	-	-	-	-	-	-	-	-	-	-
499887	-	-	-	-	-	-	-	-	-	-	-	-	-	-	-	-	-	-	-	-	-	-	-	-	-
540856	cellulolytic	-	-	-	-	-	-	-	-	-	-	-	-	-	-	-	-	-	-	-	-	-	-	-	-
348570	-	-	-	-	-	-	-	-	-	-	-	-	-	-	-	-	-	-	-	-	-	-	-	lip	-
101542	-	-	-	-	-	-	-	-	-	-	-	-	-	-	-	-	-	-	-	-	-	-	-	lip	-
4471434	-	-	-	-	-	-	-	-	-	-	-	-	-	-	-	-	-	-	-	-	-	-	-	lip	-
4302249	-	-	-	-	-	-	-	-	-	-	-	-	-	-	-	-	-	-	-	-	-	-	-	-	-
3364423	-	napA	-	-	-	nirS	-	norB	-	nifH	nifD	-	-	dsrA	dsrB	-	-	-	-	-	-	-	-	-	-
632342	-	-	-	-	-	-	-	-	-	-	-	-	-	-	-	-	-	-	-	-	-	-	-	-	-
2792167	-	-	-	-	-	-	-	-	-	-	-	-	-	-	-	-	-	-	-	-	-	-	-	-	-
1062356	-	napA	narG	-	-	-	nirK	norB	-	-	-	nrfA	ureA	-	-	-	-	-	-	-	laccase_Asco	laccase_Basidio	-	-	ppo
3271869	-	-	-	-	-	-	-	-	-	-	-	-	-	-	-	-	-	-	-	-	-	-	-	-	-
4343367	-	-	-	-	-	-	-	-	-	-	-	-	-	-	-	-	-	-	-	-	-	-	-	-	-
4482563	-	-	-	-	-	-	-	-	-	-	-	-	-	-	-	-	-	-	-	-	-	-	-	lip	-
4343369	-	-	-	-	-	-	-	-	-	-	-	-	-	-	-	-	-	-	-	-	-	-	-	-	-
4424705	-	napA	narG	nirA	-	nirS	-	norB	-	-	-	-	ureA	dsrA	dsrB	-	-	-	-	-	laccase_Asco	laccase_Basidio	-	-	ppo
4357886	-	-	-	-	-	-	-	-	-	-	-	-	-	-	-	-	-	-	-	-	-	-	-	lip	-
4357887	-	-	-	-	-	-	-	-	-	-	-	-	-	-	-	-	-	-	-	-	-	-	-	-	-
4315164	-	-	-	-	-	-	-	-	-	-	-	-	-	-	-	-	-	-	-	-	-	-	-	lip	-
826904	-	napA	-	-	-	-	nirK	-	-	nifH	-	-	ureA	-	-	-	-	chb	-	-	laccase_Asco	laccase_Basidio	-	-	ppo
820575	-	napA	narG	-	-	-	-	-	-	-	-	-	ureA	-	-	-	-	-	-	-	laccase_Asco	laccase_Basidio	-	-	ppo
152605	-	-	-	-	-	-	-	-	-	-	-	-	-	-	-	-	-	-	-	-	-	-	-	-	-
4325231	cellulolytic	-	-	-	-	-	-	-	-	-	-	-	-	-	-	-	-	-	-	-	-	-	-	-	-
909097	-	napA	-	-	-	-	-	-	-	-	nifD	-	ureA	-	-	-	-	-	-	-	-	laccase_Basidio	-	-	-
4438548	cellulolytic	napA	-	nirA	-	-	nirK	norB	nosZ	-	-	-	ureA	-	-	-	-	-	-	-	laccase_Asco	laccase_Basidio	-	-	ppo
4325237	cellulolytic	-	-	-	-	-	-	-	-	-	-	-	-	-	-	-	-	-	-	-	-	-	-	lip	-
4423750	-	-	-	-	-	-	-	-	-	-	-	-	-	-	-	-	-	-	-	-	-	-	-	-	-
790527	-	-	-	-	-	-	-	-	-	-	-	-	-	-	-	-	-	-	-	-	-	-	-	-	-
1119668	-	napA	-	-	-	-	nirK	-	-	nifH	-	-	ureA	-	-	-	-	chb	-	-	laccase_Asco	laccase_Basidio	-	-	ppo
236725	-	-	-	-	-	-	-	-	-	-	-	-	-	-	-	-	-	-	-	-	-	-	-	lip	-
543327	-	-	-	-	-	-	-	-	-	-	-	-	-	-	-	-	-	-	-	-	-	-	-	-	-
1133478	cellulolytic	napA	narG	-	-	-	nirK	-	-	-	-	-	ureA	-	-	-	-	-	-	-	laccase_Asco	laccase_Basidio	-	-	ppo
821602	-	-	-	-	-	-	-	-	-	-	-	-	-	-	-	-	-	-	-	-	-	-	-	-	-
4434799	-	napA	narG	-	-	-	nirK	-	nosZ	nifH	nifD	-	ureA	-	-	-	-	-	-	-	laccase_Asco	laccase_Basidio	-	-	ppo
517939	-	-	-	-	-	-	-	-	-	-	-	-	-	-	-	-	-	-	-	-	-	-	-	-	-
4302412	-	napA	-	-	-	nirS	-	norB	-	nifH	nifD	-	-	dsrA	dsrB	-	-	-	-	-	-	-	-	-	-
1640195	-	-	-	-	-	-	-	-	-	-	-	-	-	-	-	-	-	-	-	-	-	-	-	-	-
2400244	-	-	-	-	-	-	-	-	-	-	-	-	-	-	-	-	-	-	-	pmoA	-	-	-	-	-
113404	-	-	-	-	-	-	-	-	-	-	-	-	-	-	-	-	-	-	-	-	-	-	-	-	-
4467678	-	napA	-	-	-	-	-	norB	-	nifH	nifD	-	ureA	-	-	-	-	-	mmoX	-	laccase_Asco	laccase_Basidio	-	-	ppo
4318356	-	napA	-	-	-	-	nirK	-	-	-	-	nrfA	ureA	-	-	-	-	-	-	-	-	-	-	-	-
112133	-	-	-	-	-	-	-	-	-	-	-	-	-	-	-	-	-	-	-	-	-	-	-	-	-
841487	-	-	-	-	-	-	-	-	-	-	-	-	-	-	-	-	-	-	-	-	-	-	-	lip	-
2011775	-	-	-	-	-	-	-	-	-	-	-	-	-	-	-	-	-	-	-	-	-	-	-	lip	-
29360	-	-	-	-	-	-	-	-	-	-	-	-	-	-	-	-	-	-	-	-	-	-	-	-	-
4389007	-	-	-	-	-	-	-	-	-	-	-	-	-	-	-	-	-	-	-	-	-	-	-	lip	-
611360	-	napA	narG	nirA	-	nirS	nirK	norB	nosZ	-	-	-	ureA	-	-	-	-	-	mmoX	-	-	laccase_Basidio	-	-	ppo
844535	-	napA	narG	nirA	-	-	-	-	-	-	-	-	-	-	-	-	-	chb	-	-	laccase_Asco	laccase_Basidio	-	-	ppo
4318801	-	napA	narG	nirA	-	-	nirK	norB	-	nifH	nifD	-	ureA	-	-	-	-	chb	mmoX	-	laccase_Asco	laccase_Basidio	-	-	ppo
4477127	-	-	-	-	-	-	nirK	norB	-	-	-	nrfA	-	-	-	-	-	-	-	-	laccase_Asco	laccase_Basidio	-	-	ppo
4431277	-	-	-	-	-	-	-	-	-	-	-	-	-	-	-	-	-	-	-	-	-	-	-	lip	-
810360	-	-	-	-	-	-	-	-	-	-	-	-	-	-	-	-	-	-	-	-	-	-	-	-	-
1624	-	-	-	-	-	-	-	-	-	-	-	-	-	-	-	-	-	-	-	-	-	-	-	-	-
571199	-	-	-	-	-	-	-	-	-	-	-	-	-	-	-	-	-	-	-	-	-	-	-	-	-
809256	-	napA	-	nirA	-	-	-	-	nosZ	-	-	nrfA	ureA	-	-	-	-	-	-	-	-	laccase_Basidio	-	-	-
4425571	cellulolytic	-	-	-	-	-	-	-	-	-	-	-	-	-	-	-	-	-	-	-	-	-	-	-	-
331407	-	-	-	-	-	-	-	-	-	-	-	-	-	-	-	-	-	-	-	-	-	-	-	lip	-
4445991	-	-	-	-	-	-	-	-	-	-	-	-	-	-	-	-	-	-	-	-	-	-	-	-	-
4326874	-	napA	narG	nirA	-	nirS	nirK	norB	nosZ	-	-	-	ureA	-	-	-	-	-	mmoX	-	-	laccase_Basidio	-	-	ppo
811148	-	-	-	-	-	-	-	-	-	-	-	-	-	-	-	-	-	-	-	-	-	-	-	-	-
524860	-	-	-	-	-	-	-	-	-	-	-	-	-	-	-	-	-	-	-	-	-	-	-	-	-
4442504	cellulolytic	napA	narG	-	-	-	-	-	-	nifH	-	-	ureA	dsrA	-	-	-	-	mmoX	-	laccase_Asco	laccase_Basidio	-	-	ppo
575450	-	-	-	-	-	-	-	-	-	-	-	-	-	-	-	-	-	-	-	-	-	-	-	-	-
4470542	-	napA	narG	nirA	-	-	nirK	norB	nosZ	nifH	nifD	-	ureA	-	-	-	-	-	mmoX	-	laccase_Asco	laccase_Basidio	-	-	ppo
826124	-	napA	narG	-	-	-	nirK	norB	-	-	-	-	-	-	-	-	-	-	-	-	laccase_Asco	laccase_Basidio	-	-	ppo
2651333	-	napA	narG	-	-	-	nirK	-	-	-	-	-	ureA	-	-	-	-	-	mmoX	-	laccase_Asco	laccase_Basidio	-	-	ppo
160071	-	-	-	-	-	-	-	-	-	-	-	-	-	-	-	-	-	-	-	-	-	-	-	-	-
4321043	-	napA	-	-	-	-	-	-	-	-	-	-	ureA	-	-	-	-	-	-	-	-	-	-	-	-
3460758	cellulolytic	napA	-	-	-	-	-	-	-	-	-	-	-	-	-	-	-	-	-	-	-	laccase_Basidio	-	-	ppo
4424553	-	-	-	nirA	-	-	-	-	-	nifH	nifD	-	-	dsrA	dsrB	-	-	-	-	-	-	-	-	-	-
4469416	-	napA	narG	-	-	-	-	norB	-	-	-	-	ureA	-	-	-	-	-	-	-	laccase_Asco	laccase_Basidio	-	-	ppo
4479752	-	napA	narG	nirA	-	-	nirK	norB	nosZ	nifH	nifD	-	ureA	-	-	-	-	-	mmoX	-	laccase_Asco	laccase_Basidio	-	-	ppo
37958	cellulolytic	napA	narG	-	-	nirS	nirK	norB	nosZ	-	-	nrfA	ureA	-	-	-	-	-	mmoX	-	laccase_Asco	laccase_Basidio	-	-	ppo
4413214	-	napA	-	-	-	-	-	norB	-	nifH	nifD	-	ureA	-	-	-	-	-	mmoX	-	laccase_Asco	laccase_Basidio	-	-	ppo
4401025	cellulolytic	-	-	-	-	-	-	-	-	-	-	-	-	-	-	-	-	-	-	-	-	-	-	-	-
136119	-	-	-	-	-	-	-	-	-	-	-	-	-	-	-	-	-	-	-	-	-	-	-	-	-
4017244	-	-	-	-	-	-	nirK	norB	-	-	-	-	-	-	-	-	-	-	-	-	laccase_Asco	-	-	-	-
814647	-	-	-	-	-	-	-	-	-	-	-	-	-	-	-	-	-	-	-	-	-	-	-	-	-
4428544	cellulolytic	-	-	-	-	-	-	-	-	-	-	-	-	-	-	-	-	-	-	-	-	-	-	lip	-
541789	-	napA	-	-	-	-	nirK	-	-	-	-	nrfA	ureA	-	-	-	-	-	-	-	-	-	-	-	-
726730	cellulolytic	-	-	-	-	-	-	-	-	-	-	-	-	-	-	-	-	-	-	-	-	-	-	-	-
4477307	-	-	-	-	-	-	-	-	-	-	-	-	-	-	-	-	-	-	-	-	-	-	-	-	-
520587	-	-	-	-	-	-	-	-	-	-	-	-	-	-	-	-	-	-	-	-	-	-	-	-	-
944968	-	-	-	-	-	-	-	-	-	-	-	-	-	-	-	-	-	-	-	-	-	-	-	lip	-
161616	-	napA	-	-	-	nirS	-	norB	nosZ	nifH	nifD	-	ureA	-	-	-	-	-	mmoX	-	-	-	-	-	-
4377842	-	napA	narG	nirA	-	-	-	norB	-	-	-	-	ureA	-	-	-	-	-	mmoX	-	-	-	-	-	-
3078471	-	napA	narG	nirA	-	-	nirK	norB	nosZ	nifH	nifD	-	ureA	-	-	-	-	-	mmoX	-	laccase_Asco	laccase_Basidio	-	-	ppo
332574	-	-	-	-	-	-	-	-	-	-	-	-	-	-	-	-	-	-	-	-	-	-	-	-	-
4323373	-	-	-	-	-	-	-	-	-	-	-	-	-	-	-	-	-	-	-	-	-	-	-	lip	-
296086	-	-	-	-	-	-	-	-	-	-	-	-	-	-	-	-	-	-	-	-	-	-	-	-	-
4381918	-	-	-	-	-	-	-	-	-	-	-	-	-	-	-	-	-	-	-	-	-	-	-	lip	-
2281139	-	napA	-	-	-	-	-	norB	-	-	-	-	-	-	-	-	-	-	-	-	-	-	-	-	-
4369502	-	-	-	-	-	-	-	-	-	-	-	-	-	-	-	-	-	-	-	-	-	-	-	-	-
4421180	-	napA	narG	nirA	-	-	nirK	norB	nosZ	nifH	nifD	nrfA	ureA	-	-	-	-	chb	mmoX	-	laccase_Asco	laccase_Basidio	-	-	ppo
4369504	-	-	-	-	-	-	-	-	-	-	-	-	-	-	-	-	-	-	-	-	-	-	-	lip	-
1584969	-	-	-	-	-	-	-	-	-	-	-	-	-	-	-	-	-	-	-	-	-	-	-	-	-
1584967	cellulolytic	napA	narG	-	-	nirS	nirK	norB	nosZ	-	-	-	-	-	-	-	-	-	mmoX	-	-	laccase_Basidio	-	-	ppo
4409730	-	-	-	-	-	-	-	-	-	-	-	-	-	-	-	-	-	-	-	-	-	-	-	-	-
852722	-	-	-	-	-	-	-	-	-	-	-	-	-	-	-	-	-	-	-	-	-	-	-	-	-
4349607	-	-	-	-	-	-	-	-	-	-	-	-	-	-	-	-	-	-	-	-	-	-	-	-	-
4452696	-	-	-	-	-	-	-	-	-	-	-	-	-	-	-	-	-	-	-	-	-	-	-	lip	-
1008207	-	napA	-	-	-	-	nirK	norB	-	-	-	nrfA	ureA	-	-	-	-	chb	-	-	laccase_Asco	laccase_Basidio	-	-	ppo
992035	-	napA	narG	-	-	-	-	norB	nosZ	-	-	-	ureA	-	-	-	-	chb	mmoX	-	laccase_Asco	laccase_Basidio	-	-	ppo
680509	-	-	-	-	-	-	-	-	-	-	-	-	-	-	-	-	-	-	-	-	-	-	-	-	-
4243758	-	-	-	-	-	-	-	-	-	-	-	-	-	-	-	-	-	-	-	-	-	-	-	lip	-
4385637	-	-	-	-	-	-	-	-	-	-	-	-	-	-	-	-	-	-	-	-	-	-	-	lip	-
812105	-	-	-	-	-	-	-	-	-	-	-	-	-	-	-	-	-	-	-	-	-	-	-	-	-
4310013	-	-	-	-	-	-	-	-	-	-	-	-	-	-	-	-	-	-	-	-	-	-	-	-	-
1140475	-	-	-	-	-	-	-	-	-	-	-	-	-	-	-	-	-	-	-	-	-	-	-	-	-

2641600	0,2%	0,1%	0,1%	0,1%	0,1%	0,0%	0,2%	0,0%	0,1%	Bacteria	Proteobacteria	Gammaproteobacteria	Xanthomonadales	Xanthomonadaceae	Arenimonas	cellulolytic	-	-	-	-	nirS	-	norB	-	-	-	-	-	-	-	-	-	-	-	-	-	-	-	-	-
836331	0,0%	0,1%	0,1%	0,2%	0,1%	0,3%	0,2%	0,1%	0,0%	Bacteria	Proteobacteria	Alphaproteobacteria	Sphingomonadales	Sphingomonadaceae	Sphingomonas	-	napA	narG	nirA	-	-	nirK	norB	-	nifH	nifD	-	ureA	-	-	-	-	chb	mmoX	-	laccase_Asco	laccase_Basidio	-	-	ppo
4478426	0,0%	0,3%	0,0%	0,1%	0,0%	0,1%	0,4%	0,0%	0,0%	Bacteria	Bacteroidetes	Flavobacteriia	Flavobacteriales	[Weeksellaceae]	Chryseobacterium	-	napA	-	-	-	-	nirK	norB	nosZ	nifH	-	-	ureA	-	-	-	-	chb	-	-	laccase_Asco	laccase_Basidio	-	-	ppo
729697	0,1%	0,0%	0,3%	0,1%	0,0%	0,5%	0,0%	0,1%	0,3%	Bacteria	Actinobacteria	Thermoleophilia	Solirubrobacterales	Conexibacteraceae	Conexibacter	-	-	narG	-	-	-	-	-	-	-	-	-	ureA	-	-	-	-	-	-	-	-	laccase_Basidio	-	-	-
241204	0,0%	0,2%	0,2%	0,2%	0,3%	0,0%	0,0%	0,0%	0,0%	Bacteria	Proteobacteria	Alphaproteobacteria	Rhodospirillales	Rhodospirillaceae	Azospirillum	-	napA	-	nirA	-	nirS	nirK	norB	nosZ	nifH	nifD	-	ureA	-	-	-	-	-	mmoX	-	laccase_Asco	laccase_Basidio	-	-	ppo
212688	0,7%	0,0%	0,0%	0,0%	0,0%	0,0%	0,0%	0,0%	0,0%	Bacteria	Proteobacteria	Alphaproteobacteria	Caulobacterales	Caulobacteraceae	uncultured	-	-	-	-	-	-	-	-	-	-	-	-	-	-	-	-	-	-	-	-	-	-	-	lip	-
81438	0,0%	0,2%	0,2%	0,2%	0,2%	0,0%	0,0%	0,0%	0,1%	Bacteria	Proteobacteria	Deltaproteobacteria	Desulfuromonadales	Geobacteraceae	Geobacter	-	napA	narG	-	-	-	-	norB	-	nifH	nifD	nrfA	-	-	-	-	-	-	-	-	laccase_Asco	laccase_Basidio	-	-	ppo
873518	0,5%	0,0%	0,0%	0,0%	0,1%	0,0%	0,0%	0,0%	0,0%	Bacteria	Proteobacteria	Deltaproteobacteria	Myxococcales		uncultured bacterium	-	-	-	-	-	-	-	-	-	-	-	-	-	-	-	-	-	-	-	-	-	-	-	-	-
4339146	0,1%	0,2%	0,1%	0,1%	0,1%	0,4%	0,0%	0,2%	0,4%	Bacteria	Firmicutes	Bacilli	Bacillales	Paenibacillaceae	Paenibacillus	-	napA	narG	-	-	nirS	nirK	norB	nosZ	nifH	nifD	-	ureA	-	-	-	-	-	-	-	laccase_Asco	laccase_Basidio	-	-	ppo
279993	0,1%	0,1%	0,1%	0,2%	0,0%	0,0%	0,2%	0,1%	0,4%	Bacteria	Proteobacteria	Betaproteobacteria	SC-I-84		uncultured bacterium	-	-	-	-	-	-	-	-	-	-	-	-	-	-	-	-	-	-	-	-	-	-	-	-	-
1068936	0,0%	0,0%	0,0%	0,1%	0,2%	0,1%	0,3%	0,0%	0,0%	Bacteria	Actinobacteria	Acidimicrobiia	Acidimicrobiales		Candidatus Microthrix	-	-	-	-	-	-	-	-	-	-	-	-	-	-	-	-	-	-	-	-	-	-	-	-	-
542789	0,0%	0,2%	0,2%	0,1%	0,0%	0,0%	0,2%	0,0%	0,0%	Bacteria	Chlamydiae	Chlamydiia	Chlamydiales	Parachlamydiaceae	Candidatus Protochlamydia	-	-	-	-	-	-	-	-	-	-	-	-	-	-	-	-	-	-	-	-	-	-	-	-	-
1018359	0,2%	0,0%	0,0%	0,5%	0,1%	0,1%	0,0%	0,2%	0,0%	Bacteria	Bacteroidetes	Cytophagia	Cytophagales	Cytophagaceae	Dyadobacter	cellulolytic	napA	-	nirA	-	-	-	norB	nosZ	-	-	-	-	-	-	-	-	chb	-	-	-	-	-	-	-
760263	0,2%	0,1%	0,1%	0,1%	0,2%	0,3%	0,0%	0,0%	0,0%	Bacteria	Firmicutes	Clostridia	Clostridiales	Ruminococcaceae	uncultured	cellulolytic	-	-	-	-	-	-	-	-	-	-	-	-	-	-	-	-	-	-	-	-	-	-	lip	-
35840	0,1%	0,1%	0,1%	0,2%	0,1%	0,0%	0,3%	0,1%	0,0%	Bacteria	Proteobacteria	Betaproteobacteria	Neisseriales	Neisseriaceae	Iodobacter	-	-	-	-	-	-	-	-	-	-	-	-	-	-	-	-	-	-	-	-	-	-	-	-	-
4448785	0,0%	0,1%	0,2%	0,0%	0,2%	0,2%	0,1%	0,0%	0,0%	Bacteria	Firmicutes	Clostridia	Clostridiales	Veillonellaceae	Pelosinus	-	napA	narG	nirA	-	-	-	norB	-	nifH	nifD	nrfA	-	-	-	-	-	-	-	-	-	-	-	-	-
3487653	0,0%	0,0%	0,2%	0,2%	0,1%	0,5%	0,0%	0,0%	0,1%	Bacteria	Proteobacteria	Betaproteobacteria	Burkholderiales	Comamonadaceae	Aquabacterium	-	-	-	-	-	-	-	-	-	-	-	-	-	-	-	-	-	-	-	-	-	-	-	-	-
4341734	0,0%	0,1%	0,1%	0,0%	0,0%	0,0%	0,5%	0,0%	0,0%	Bacteria	Actinobacteria	Actinobacteria	Actinomycetales	Microbacteriaceae	Amnibacterium	cellulolytic	-	-	-	-	-	-	-	-	-	-	-	-	-	-	-	-	-	-	-	-	-	-	-	-
3581985	0,1%	0,0%	0,0%	0,1%	0,0%	0,0%	0,4%	0,0%	0,1%	Bacteria	Proteobacteria	Betaproteobacteria	Burkholderiales	Comamonadaceae	uncultured	-	-	-	-	-	-	-	-	-	-	-	-	-	-	-	-	-	-	-	-	-	-	-	lip	-
842453	0,0%	0,1%	0,1%	0,5%	0,1%	0,0%	0,1%	0,1%	0,0%	Bacteria	Acidobacteria	Acidobacteriia	Acidobacteriales	Acidobacteriaceae	Bryocella	cellulolytic	-	-	-	-	-	-	-	-	-	-	-	-	-	-	-	-	-	-	-	-	-	-	-	-
512172	0,0%	0,2%	0,1%	0,1%	0,2%	0,2%	0,1%	0,0%	0,1%	Bacteria	Verrucomicrobia	[Spartobacteria]	[Chthoniobacterales]	[Chthoniobacteraceae]	uncultured eubacterium WCHD3-88	-	-	-	-	-	-	-	-	-	-	-	-	-	-	-	-	-	-	-	-	-	-	-	-	-
4454257	0,1%	0,0%	0,1%	0,1%	0,1%	0,0%	0,2%	0,1%	0,0%	Bacteria	Proteobacteria	Gammaproteobacteria	Enterobacteriales	Enterobacteriaceae	Enterobacter	cellulolytic	napA	narG	-	-	-	-	-	-	nifH	nifD	nrfA	ureA	-	-	-	-	chb	-	-	laccase_Asco	laccase_Basidio	-	-	ppo
244496	0,0%	0,0%	0,0%	0,2%	0,1%	0,0%	0,4%	0,0%	0,0%	Bacteria	Verrucomicrobia	[Spartobacteria]	[Chthoniobacterales]	[Chthoniobacteraceae]	uncultured bacterium	-	-	-	-	-	-	-	-	-	-	-	-	-	-	-	-	-	-	-	-	-	-	-	-	-
3330580	0,0%	0,2%	0,1%	0,0%	0,1%	0,0%	0,3%	0,0%	0,0%	Bacteria	Proteobacteria	Gammaproteobacteria	Xanthomonadales	Xanthomonadaceae	Stenotrophomonas	cellulolytic	napA	narG	-	-	-	-	norB	-	nifH	-	-	-	-	-	-	-	-	-	-	laccase_Asco	laccase_Basidio	-	-	ppo
350772	0,0%	0,1%	0,1%	0,2%	0,1%	0,1%	0,2%	0,1%	0,0%	Bacteria	Verrucomicrobia	Opitutae	Opitutales	Opitutaceae	Opitutus	-	napA	-	nirA	-	-	-	-	nosZ	-	-	nrfA	ureA	-	-	-	-	-	-	-	-	laccase_Basidio	-	-	-
1857060	0,5%	0,0%	0,0%	0,0%	0,0%	0,0%	0,0%	0,1%	0,0%	Bacteria	Bacteroidetes	[Saprospirae]	[Saprospirales]	Chitinophagaceae	uncultured	-	-	-	-	-	-	-	-	-	-	-	-	-	-	-	-	-	-	-	-	-	-	-	lip	-
4393554	0,2%	0,1%	0,1%	0,1%	0,0%	0,0%	0,2%	0,1%	0,0%	Bacteria	Proteobacteria	Gammaproteobacteria	Legionellales	Legionellaceae	Legionella	-	-	-	-	-	nirS	-	norB	-	-	-	-	-	-	-	-	-	-	-	-	laccase_Asco	laccase_Basidio	-	-	ppo
254922	0,0%	0,1%	0,2%	0,0%	0,3%	0,0%	0,0%	0,0%	0,0%	Bacteria	Proteobacteria	Alphaproteobacteria	Rhodospirillales	Acetobacteraceae	Acidocella	-	napA	narG	nirA	-	-	-	-	-	-	-	-	ureA	-	-	-	-	-	-	-	laccase_Asco	laccase_Basidio	-	-	ppo
157514	0,0%	0,1%	0,2%	0,1%	0,1%	0,0%	0,2%	0,0%	0,3%	Bacteria	Proteobacteria	Alphaproteobacteria	Rhizobiales	Bradyrhizobiaceae	Rhizobiales bacterium KVD-1959-05	-	-	-	-	-	-	-	-	-	-	-	-	-	-	-	-	-	-	-	-	-	-	-	-	-
4483373	0,0%	0,1%	0,3%	0,1%	0,1%	0,1%	0,1%	0,0%	0,0%	Bacteria	Proteobacteria	Alphaproteobacteria	Caulobacterales	Caulobacteraceae	uncultured	-	-	-	-	-	-	-	-	-	-	-	-	-	-	-	-	-	-	-	-	-	-	-	lip	-
4393791	0,0%	0,0%	0,0%	0,0%	0,0%	0,0%	0,5%	0,0%	0,0%	Bacteria	Bacteroidetes	[Saprospirae]	[Saprospirales]	Chitinophagaceae	Ferruginibacter	-	-	-	-	-	-	-	-	-	-	-	-	-	-	-	-	-	-	-	-	-	-	-	-	-
1148212	0,2%	0,1%	0,1%	0,2%	0,1%	0,0%	0,1%	0,1%	0,1%	Bacteria	Planctomycetes	Planctomycetia	Gemmatales	Gemmataceae	Gemmata	-	-	-	-	-	-	-	-	-	-	-	-	-	-	-	-	-	-	-	-	-	-	-	-	-
3447868	0,0%	0,0%	0,2%	0,1%	0,2%	0,0%	0,1%	0,0%	0,0%	Bacteria	Proteobacteria	Alphaproteobacteria	Rhizobiales	Rhizobiaceae	Rhizobium	-	napA	narG	nirA	-	-	nirK	norB	nosZ	nifH	nifD	nrfA	ureA	-	-	-	-	chb	mmoX	-	laccase_Asco	laccase_Basidio	-	-	ppo
4483428	0,2%	0,1%	0,1%	0,0%	0,1%	0,1%	0,0%	0,1%	0,0%	Bacteria	Actinobacteria	Thermoleophilia	Gaiellales	Gaiellaceae	uncultured bacterium	-	-	-	-	-	-	-	-	-	-	-	-	-	-	-	-	-	-	-	-	-	-	-	-	-
810425	0,0%	0,2%	0,2%	0,1%	0,2%	0,1%	0,0%	0,1%	0,1%	Bacteria	Actinobacteria	Actinobacteria	Actinomycetales	Corynebacteriaceae	Corynebacterium	-	napA	narG	-	-	-	nirK	norB	-	-	-	nrfA	ureA	-	-	-	-	-	-	-	laccase_Asco	laccase_Basidio	-	-	ppo
254888	0,4%	0,0%	0,0%	0,1%	0,0%	0,0%	0,2%	0,1%	0,0%	Bacteria	Proteobacteria	Betaproteobacteria	Burkholderiales	Comamonadaceae	Pelomonas	-	napA	narG	nirA	-	nirS	-	norB	nosZ	nifH	-	-	ureA	-	-	-	-	-	-	-	-	-	-	-	-
3623039	0,0%	0,1%	0,1%	0,1%	0,1%	0,0%	0,3%	0,0%	0,0%	Bacteria	Proteobacteria	Deltaproteobacteria	Myxococcales		uncultured bacterium	-	-	-	-	-	-	-	-	-	-	-	-	-	-	-	-	-	-	-	-	-	-	-	-	-
4404743	0,1%	0,1%	0,1%	0,1%	0,1%	0,0%	0,3%	0,0%	0,0%	Bacteria	Proteobacteria	Gammaproteobacteria	Methylococcales	Crenotrichaceae	Crenothrix	-	-	-	-	-	-	-	-	-	-	-	-	-	-	-	-	-	-	-	pmoA	-	-	-	-	-
982819	0,0%	0,1%	0,0%	0,2%	0,1%	0,0%	0,2%	0,1%	0,3%	Bacteria	Proteobacteria	Deltaproteobacteria	MIZ46		uncultured bacterium	-	-	-	-	-	-	-	-	-	-	-	-	-	-	-	-	-	-	-	-	-	-	-	-	-
4477623	0,5%	0,0%	0,0%	0,0%	0,0%	0,0%	0,0%	0,0%	0,0%	Bacteria	Nitrospirae	Nitrospira	Nitrospirales	[Thermodesulfovibrionaceae]	uncultured	-	-	-	-	-	-	-	-	-	-	-	-	-	-	-	-	-	-	-	-	-	-	-	lip	-
19964	0,0%	0,2%	0,1%	0,1%	0,1%	0,0%	0,1%	0,1%	0,1%	Bacteria	Actinobacteria	Thermoleophilia	Solirubrobacterales		uncultured bacterium	-	-	-	-	-	-	-	-	-	-	-	-	-	-	-	-	-	-	-	-	-	-	-	-	-
222554	0,3%	0,2%	0,1%	0,1%	0,0%	0,0%	0,0%	0,0%	0,0%	Bacteria	Proteobacteria	Betaproteobacteria	Methylophilales	Methylophilaceae	Methylotenera	-	napA	-	nirA	-	-	nirK	norB	-	-	-	-	ureA	-	-	-	-	-	-	-	-	-	-	-	-
1142257	0,0%	0,0%	0,0%	0,1%	0,3%	0,1%	0,0%	0,1%	0,0%	Bacteria	Actinobacteria	Actinobacteria	Actinomycetales	Microbacteriaceae	Pseudoclavibacter	cellulolytic	-	-	-	-	-	-	-	-	-	-	-	-	-	-	-	-	-	-	-	-	-	-	-	-
150766	0,0%	0,1%	0,1%	0,0%	0,1%	0,3%	0,2%	0,0%	0,1%	Bacteria	Planctomycetes	Planctomycetia	Gemmatales	Isosphaeraceae	Singulisphaera	-	napA	-	-	-	-	-	-	-	-	-	-	ureA	-	-	-	-	-	-	-	-	-	-	-	-
581763	0,0%	0,0%	0,2%	0,0%	0,1%	0,1%	0,3%	0,0%	0,0%	Bacteria	Proteobacteria	Alphaproteobacteria	Sphingomonadales	Sphingomonadaceae	Novosphingobium	-	napA	-	-	-	-	-	norB	-	nifH	nifD	-	ureA	-	-	-	-	-	mmoX	-	laccase_Asco	laccase_Basidio	-	-	ppo
4225240	0,0%	0,0%	0,1%	0,0%	0,0%	0,1%	0,4%	0,0%	0,0%	Bacteria	Verrucomicrobia	Opitutae	Opitutales	Opitutaceae	Opitutus	-	napA	-	nirA	-	-	-	-	nosZ	-	-	nrfA	ureA	-	-	-	-	-	-	-	-	laccase_Basidio	-	-	-
832844	0,0%	0,0%	0,3%	0,1%	0,1%	0,2%	0,0%	0,1%	0,3%	Bacteria	Proteobacteria	Deltaproteobacteria	Myxococcales	Myxococcaceae	Anaeromyxobacter	-	napA	narG	nirA	-	nirS	-	norB	nosZ	nifH	nifD	nrfA	ureA	-	-	SoxB	-	-	-	-	-	laccase_Basidio	-	-	-
4396454	0,5%	0,0%	0,0%	0,0%	0,0%	0,0%	0,0%	0,0%	0,0%	Bacteria	Proteobacteria	Betaproteobacteria	Burkholderiales	Comamonadaceae	uncultured	-	-	-	-	-	-	-	-	-	-	-	-	-	-	-	-	-	-	-	-	-	-	-	lip	-
802011	0,1%	0,2%	0,2%	0,1%	0,1%	0,0%	0,0%	0,0%	0,1%	Bacteria	Proteobacteria	Betaproteobacteria	Methylophilales	Methylophilaceae	Methylotenera	-	napA	-	nirA	-	-	nirK	norB	-	-	-	-	ureA	-	-	-	-	-	-	-	-	-	-	-	-
533472	0,0%	0,1%	0,1%	0,1%	0,3%	0,0%	0,0%	0,1%	0,0%	Bacteria	Acidobacteria	Solibacteres	Solibacterales		Bryobacter	-	-	-	-	-	-	-	-	-	-	-	-	-	-	-	-	-	-	-	-	-	-	-	-	-
311929	0,0%	0,1%	0,2%	0,1%	0,1%	0,1%	0,1%	0,1%	0,1%	Bacteria	Proteobacteria	Deltaproteobacteria	NB1-j	JTB38	uncultured bacterium	-	-	-	-	-	-	-	-	-	-	-	-	-	-	-	-	-	-	-	-	-	-	-	-	-
4451003	0,0%	0,0%	0,1%	0,1%	0,0%	0,6%	0,0%	0,2%	0,5%	Bacteria	Proteobacteria	Betaproteobacteria	Burkholderiales	Oxalobacteraceae	Achromobacter	-	napA	narG	-	-	nirS	nirK	norB	nosZ	-	-	nrfA	ureA	-	-	-	-	-	mmoX	-	laccase_Asco	laccase_Basidio	-	-	ppo
4389637	0,0%	0,1%	0,1%	0,1%	0,1%	0,0%	0,1%	0,1%	0,0%	Bacteria	Proteobacteria	Deltaproteobacteria	Desulfuromonadales	Geobacteraceae	uncultured Geobacteraceae bacterium	-	-	-	-	-	-	-	-	-	-	-	-	-	-	-	-	-	-	-	-	-	-	-	-	-
4346675	0,5%	0,0%	0,0%	0,0%	0,0%	0,0%	0,0%	0,0%	0,0%	Bacteria	Firmicutes	Clostridia	Clostridiales	Ruminococcaceae	Subdoligranulum	cellulolytic	-	-	nirA	-	-	-	-	-	nifH	-	-	-	-	-	-	-	-	-	-	-	-	-	-	-
4321860	0,0%	0,0%	0,0%	0,0%	0,0%	0,0%	0,5%	0,0%	0,0%	Bacteria	Proteobacteria	Alphaproteobacteria	Rhodobacterales	Rhodobacteraceae	Defluviimonas	-	napA	narG	-	-	-	-	-	-	-	-	-	ureA	-	-	-	-	-	-	-	-	laccase_Basidio	-	-	ppo
4342972	0,4%	0,0%	0,0%	0,0%	0,0%	0,1%	0,0%	0,2%	0,1%	Bacteria	Verrucomicrobia	[Spartobacteria]	[Chthoniobacterales]	[Chthoniobacteraceae]	uncultured bacterium	-	-	-	-	-	-	-	-	-	-	-	-	-	-	-	-	-	-	-	-	-	-	-	-	-
251499	0,1%	0,1%	0,0%	0,1%	0,2%	0,1%	0,0%	0,0%	0,0%	Bacteria	Verrucomicrobia	[Spartobacteria]	[Chthoniobacterales]	[Chthoniobacteraceae]	Chthoniobacter	-	napA	-	-	-	-	nirK	-	-	-	-	nrfA	ureA	-	-	-	-	-	-	-	-	-	-	-	-
221334	0,3%	0,0%	0,0%	0,0%	0,1%	0,0%	0,0%	0,1%	0,0%	Bacteria	Verrucomicrobia	[Spartobacteria]	[Chthoniobacterales]	[Chthoniobacteraceae]	Chthoniobacter	-	napA	-	-	-	-	nirK	-	-	-	-	nrfA	ureA	-	-	-	-	-	-	-	-	-	-	-	-
4456673	0,0%	0,1%	0,1%	0,0%	0,0%	0,0%	0,3%	0,0%	0,0%	Bacteria	Verrucomicrobia	Verrucomicrobiae	Verrucomicrobiales	Verrucomicrobiaceae	Luteolibacter	-	-	-	-	-	-	-	-	-	-	-	-	-	-	-	-	-	-	-	-	-	-	-	-	-
865086	0,3%	0,0%	0,1%	0,0%	0,0%	0,0%	0,0%	0,0%	0,0%	Bacteria	Bacteroidetes	Cytophagia	Cytophagales	Cytophagaceae	Cytophaga	cellulolytic	napA	-	-	-	-	-	norB	-	-	nifD	-	ureA	-	-	-	-	-	-	-	-	-	-	-	-
61836	0,5%	0,0%	0,0%	0,0%	0,0%	0,0%	0,0%	0,0%	0,0%	Bacteria	Proteobacteria	Betaproteobacteria	Burkholderiales	Comamonadaceae	Leptothrix	-	napA	-	-	-	nirS	-	norB	nosZ	nifH	nifD	-	ureA	-	-	-	-	-	mmoX	-	-	-	-	-	-
4469924	0,0%	0,1%	0,0%	0,0%	0,0%	0,1%	0,3%	0,2%	0,0%	Bacteria	Firmicutes	Bacilli	Bacillales	Bacillaceae	Anoxybacillus	cellulolytic	napA	narG	-	-	-	-	-	nosZ	-	-	nrfA	ureA	-	-	-	-	-	-	-	laccase_Asco	laccase_Basidio	-	-	-
2360704	0,3%	0,0%	0,0%	0,0%	0,0%	0,0%	0,2%	0,0%	0,0%	Bacteria	Proteobacteria	Betaproteobacteria	Burkholderiales	Comamonadaceae	Variovorax	-	napA	narG	nirA	-	-	-	norB	-	-	-	-	ureA	-	-	-	-	-	mmoX	-	-	-	-	-	-
665877	0,2%	0,0%	0,1%	0,1%	0,1%	0,0%	0,0%	0,1%	0,0%	Bacteria	Actinobacteria	Actinobacteria	Actinomycetales		uncultured	-	-	-	-	-	-	-	-	-	-	-	-	-	-	-	-	-	-	-	-	-	-	-	lip	-
4449185	0,0%	0,0%	0,1%	0,2%	0,1%	0,1%	0,1%	0,1%	0,1%	Bacteria	Proteobacteria	Alphaproteobacteria	Rhizobiales	Phyllobacteriaceae	Mesorhizobium	-	napA	narG	nirA	-	-	nirK	norB	nosZ	nifH	nifD	-	ureA	-	-	-	-	-	mmoX	-	laccase_Asco	laccase_Basidio	-	-	ppo
724366	0,1%	0,2%	0,0%	0,1%	0,1%	0,0%	0,1%	0,0%	0,0%	Bacteria	Proteobacteria	Gammaproteobacteria	Methylococcales	Crenotrichaceae	Crenothrix	-	-	-	-	-	-	-	-	-	-	-	-	-	-	-	-	-	-	-	pmoA	-	-	-	-	-
4357500	0,0%	0,1%	0,1%	0,1%	0,2%	0,1%	0,0%	0,1%	0,1%	Bacteria	Proteobacteria	Betaproteobacteria	Burkholderiales	Comamonadaceae	Leptothrix	-	napA	-	-	-	nirS	-	norB	nosZ	nifH	nifD	-	ureA	-	-	-	-	-	mmoX	-	-	-	-	-	-
11622	0,1%	0,1%	0,0%	0,0%	0,0%	0,0%	0,3%	0,0%	0,4%	Bacteria	Actinobacteria	Actinobacteria	Actinomycetales	Frankiaceae	Jatrophihabitans	-	-	-	-	-	-	-	-	-	-	-	-	-	-	-	-	-	-	-	-	-	-	-	-	-
101445	0,2%	0,1%	0,0%	0,1%	0,1%	0,0%	0,0%	0,0%	0,3%	Bacteria	Proteobacteria	Betaproteobacteria	Methylophilales	Methylophilaceae	uncultured	-	-	-	-	-	-	-	-	-	-	-	-	-	-	-	-	-	-	-	-	-	-	-	lip	-
647790	0,0%	0,0%	0,2%	0,0%	0,0%	0,0%	0,2%	0,0%	0,0%	Bacteria	Proteobacteria	Alphaproteobacteria	Rhizobiales	Methylocystaceae	uncultured bacterium	-	-	-	-	-	-	-	-	-	-	-	-	-	-	-	-	-	-	-	-	-	-	-	-	-
253816	0,0%	0,1%	0,1%	0,1%	0,0%	0,9%	0,0%	0,0%	0,3%	Bacteria	Proteobacteria	Gammaproteobacteria	Methylococcales	Crenotrichaceae	Methyloglobulus	-	napA	-	-	-	-	-	-	-	nifH	nifD	-	ureA	-	-	-	-	-	-	pmoA	laccase_Asco	laccase_Basidio	-	-	ppo
809666	0,3%	0,1%	0,1%	0,1%	0,0%	0,0%	0,0%	0,0%	0,0%	Bacteria	Proteobacteria	TA18	PHOS-HD29		uncultured bacterium	-	-	-	-	-	-	-	-	-	-	-	-	-	-	-	-	-	-	-	-	-	-	-	-	-
627479	0,4%	0,0%	0,0%	0,0%	0,0%	0,0%	0,0%	0,0%	0,1%	Bacteria	Proteobacteria	Alphaproteobacteria	Sphingomonadales	Sphingomonadaceae	Sphingomonas	-	napA	narG	nirA	-	-	nirK	norB	-	nifH	nifD	-	ureA	-	-	-	-	chb	mmoX	-	laccase_Asco	laccase_Basidio	-	-	ppo
226240	0,0%	0,0%	0,0%	0,0%	0,0%	0,0%	0,4%	0,0%	0,0%	Bacteria	Verrucomicrobia	Opitutae	Opitutales	Opitutaceae	Opitutus	-	napA	-	nirA	-	-	-	-	nosZ	-	-	nrfA	ureA	-	-	-	-	-	-	-	-	laccase_Basidio	-	-	-
4411088	0,1%	0,0%	0,0%	0,0%	0,3%	0,0%	0,0%	0,0%	0,0%	Bacteria	Actinobacteria	Actinobacteria	Actinomycetales		Acidothermus	-	napA	narG	-	-	-	nirK	-	-	-	-	-	-	-	-	-	-	-	-	-	-	-	-	-	-
689939	0,0%	0,1%	0,1%	0,1%	0,1%	0,0%	0,0%	0,1%	0,1%	Bacteria	Acidobacteria	BPC102	B110		uncultured bacterium	-	-	-	-	-	-	-	-	-	-	-	-	-	-	-	-	-	-	-	-	-	-	-	-	-
1109749	0,2%	0,1%	0,1%	0,0%	0,0%	0,1%	0,0%	0,0%	0,0%	Bacteria	Proteobacteria	Alphaproteobacteria	Ellin329		Rhizomicrobium	-	-	-	-	-	-	-	-	-	-	-	-	-	-	-	-	-	-	-	-	-	-	-	-	-
4456129	0,0%	0,0%	0,0%	0,0%	0,0%	0,0%	0,4%	0,0%	0,0%	Bacteria	Proteobacteria	Gammaproteobacteria	Alteromonadales	Alteromonadaceae	Cellvibrio	-	napA	narG	-	-	-	nirK	norB	-	-	-	-	-	-	-	-	-	chb	-	-	-	laccase_Basidio	-	-	-
96941	0,3%	0,0%	0,0%	0,0%	0,0%	0,1%	0,0%	0,1%	0,0%	Bacteria	Proteobacteria	Betaproteobacteria	Methylophilales	Methylophilaceae	uncultured	-	-	-	-	-	-	-	-	-	-	-	-	-	-	-	-	-	-	-	-	-	-	-	lip	-
4396611	0,0%	0,2%	0,0%	0,0%	0,0%	0,0%	0,2%	0,0%	0,0%	Bacteria	Bacteroidetes	Sphingobacteriia	Sphingobacteriales	Sphingobacteriaceae	Solitalea	-	napA	-	nirA	-	-	nirK	norB	nosZ	-	-	nrfA	-	-	-	-	-	chb	-	-	-	-	-	-	-
4458460	0,0%	0,0%	0,1%	0,0%	0,1%	0,0%	0,1%	0,1%	0,0%	Bacteria	Actinobacteria	Acidimicrobiia	Acidimicrobiales	C111	CL500-29 marine group	-	-	-	-	-	-	-	-	-	-	-	-	-	-	-	-	-	-	-	-	-	-	-	-	-
510937	0,0%	0,2%	0,1%	0,1%	0,1%	0,1%	0,0%	0,0%	0,3%	Bacteria	Proteobacteria	Deltaproteobacteria	Myxococcales		Sorangium	-	napA	-	-	-	-	nirK	norB	-	-	-	nrfA	ureA	-	-	-	-	chb	-	-	laccase_Asco	laccase_Basidio	-	-	ppo
153239	0,0%	0,1%	0,1%	0,1%	0,2%	0,0%	0,0%	0,0%	0,0%	Bacteria	Verrucomicrobia	[Pedosphaerae]	[Pedosphaerales]		uncultured bacterium	-	-	-	-	-	-	-	-	-	-	-	-	-	-	-	-	-	-	-	-	-	-	-	-	-
4330365	0,0%	0,1%	0,1%	0,1%	0,1%	0,1%	0,1%	0,0%	0,0%	Bacteria	Firmicutes	Bacilli	Bacillales	Bacillaceae	Bacillus	cellulolytic	napA	narG	nirA	-	nirS	nirK	norB	nosZ	nifH	nifD	nrfA	ureA	-	-	-	-	-	mmoX	-	laccase_Asco	laccase_Basidio	-	-	ppo
3627262	0,0%	0,0%	0,0%	0,0%	0,1%	0,7%	0,0%	0,2%	0,1%	Bacteria	Acidobacteria	Acidobacteria-6	CCU21		uncultured Acidobacteria bacterium	-	-	-	-	-	-	-	-	-	-	-	-	-	-	-	-	-	-	-	-	-	-	-	-	-
4415366	0,3%	0,0%	0,0%	0,0%	0,0%	0,0%	0,0%	0,4%	0,7%	Bacteria	Planctomycetes	Phycisphaerae	WD2101		uncultured bacterium	-	-	-	-	-	-	-	-	-	-	-	-	-	-	-	-	-	-	-	-	-	-	-	-	-
4362555	0,0%	0,1%	0,0%	0,0%	0,1%	0,3%	0,2%	0,0%	0,0%	Bacteria	Verrucomicrobia	[Spartobacteria]	[Chthoniobacterales]	[Chthoniobacteraceae]	uncultured bacterium	-	-	-	-	-	-	-	-	-	-	-	-	-	-	-	-	-	-	-	-	-	-	-	-	-
1053194	0,4%	0,0%	0,0%	0,0%	0,0%	0,0%	0,0%	0,0%	0,0%	Bacteria	Proteobacteria	Gammaproteobacteria	Xanthomonadales	Sinobacteraceae	uncultured proteobacterium	-	-	-	-	-	-	-	-	-	-	-	-	-	-	-	-	-	-	-	-	-	-	-	-	-
4366665	0,2%	0,0%	0,1%	0,1%	0,1%	0,0%	0,0%	0,0%	0,0%	Bacteria	Bacteroidetes	Bacteroidia	Bacteroidales		uncultured bacterium	-	-	-	-	-	-	-	-	-	-	-	-	-	-	-	-	-	-	-	-	-	-	-	-	-
4443003	0,0%	0,3%	0,0%	0,0%	0,1%	0,0%	0,1%	0,0%	0,0%	Bacteria	Planctomycetes	Planctomycetia	Pirellulales	Pirellulaceae	Pirellula	-	napA	-	-	-	-	-	norB	-	-	-	-	-	-	-	-	-	-	-	-	-	-	-	-	-
4476648	0,0%	0,0%	0,0%	0,0%	0,0%	0,0%	0,4%	0,0%	0,0%	Bacteria	Proteobacteria	Betaproteobacteria	Burkholderiales	Comamonadaceae	Ramlibacter	-	napA	-	nirA	-	-	-	-	-	-	-	-	ureA	-	-	-	-	-	-	-	laccase_Asco	laccase_Basidio	-	-	ppo
4456892	0,4%	0,0%	0,0%	0,0%	0,0%	0,1%	0,0%	0,0%	0,0%	Bacteria	Proteobacteria	Gammaproteobacteria	Enterobacteriales	Enterobacteriaceae	Erwinia	cellulolytic	napA	narG	-	-	-	-	-	-	nifH	-	-	-	-	-	-	-	chb	-	-	laccase_Asco	laccase_Basidio	-	-	ppo
4456891	0,0%	0,1%	0,1%	0,0%	0,1%	0,0%	0,2%	0,0%	0,0%	Bacteria	Proteobacteria	Gammaproteobacteria	Pseudomonadales	Pseudomonadaceae	Pseudomonas	cellulolytic	napA	narG	nirA	-	nirS	nirK	norB	nosZ	nifH	nifD	-	ureA	-	dsrB	SoxB	-	-	mmoX	-	laccase_Asco	laccase_Basidio	-	-	ppo
4362775	0,0%	0,0%	0,0%	0,1%	0,2%	0,1%	0,0%	0,1%	0,1%	Bacteria	Proteobacteria	Alphaproteobacteria	Rhizobiales	Hyphomicrobiaceae	Pedomicrobium	-	-	-	-	-	-	nirK	-	-	-	-	-	-	-	-	-	-	-	-	-	-	-	-	-	-
1145091	0,0%	0,1%	0,0%	0,4%	0,1%	0,1%	0,0%	0,1%	0,8%	Bacteria	Proteobacteria	Alphaproteobacteria	Rhizobiales	Phyllobacteriaceae	uncultured bacterium	-	-	-	-	-	-	-	-	-	-	-	-	-	-	-	-	-	-	-	-	-	-	-	-	-
4433032	0,0%	0,1%	0,1%	0,1%	0,1%	0,0%	0,0%	0,0%	0,1%	Bacteria	Planctomycetes	Planctomycetia	Gemmatales	Gemmataceae	uncultured	-	-	-	-	-	-	-	-	-	-	-	-	-	-	-	-	-	-	-	-	-	-	-	lip	-
168212	0,0%	0,0%	0,1%	0,2%	0,2%	0,0%	0,0%	0,0%	0,0%	Bacteria	Proteobacteria	Alphaproteobacteria	Caulobacterales	Caulobacteraceae	uncultured	-	-	-	-	-	-	-	-	-	-	-	-	-	-	-	-	-	-	-	-	-	-	-	lip	-
83595	0,4%	0,0%	0,0%	0,0%	0,0%	0,0%	0,0%	0,0%	0,1%	Bacteria	Cyanobacteria	Synechococcophycideae	Pseudanabaenales	Pseudanabaenaceae	Phormidium	-	napA	-	nirA	-	-	-	norB	-	-	nifD	-	ureA	-	-	-	-	-	-	-	-	-	-	-	-
4348899	0,0%	0,0%	0,2%	0,2%	0,1%	0,0%	0,0%	0,0%	0,0%	Bacteria	Verrucomicrobia	Opitutae	Opitutales	Opitutaceae	Opitutus	-	napA	-	nirA	-	-	-	-	nosZ	-	-	nrfA	ureA	-	-	-	-	-	-	-	-	laccase_Basidio	-	-	-
1046774	0,2%	0,0%	0,1%	0,1%	0,0%	0,0%	0,0%	0,0%	0,1%	Bacteria	Bacteroidetes	Flavobacteriia	Flavobacteriales	Flavobacteriaceae	Flavobacterium	cellulolytic	napA	-	nirA	-	-	nirK	norB	nosZ	-	-	-	ureA	-	-	-	-	-	-	-	laccase_Asco	laccase_Basidio	-	-	ppo
4350665	0,0%	0,0%	0,1%	0,1%	0,0%	0,2%	0,1%	0,1%	0,1%	Bacteria	Proteobacteria	Betaproteobacteria	Burkholderiales	Oxalobacteraceae	uncultured bacterium	-	-	-	-	-	-	-	-	-	-	-	-	-	-	-	-	-	-	-	-	-	-	-	-	-
338371	0,0%	0,0%	0,0%	0,0%	0,0%	0,0%	0,4%	0,0%	0,0%	Bacteria	Proteobacteria	Deltaproteobacteria	Bdellovibrionales	Bdellovibrionaceae	Bdellovibrio	-	-	-	-	-	-	nirK	norB	-	-	-	nrfA	-	-	-	-	-	-	-	-	laccase_Asco	laccase_Basidio	-	-	ppo
587098	0,3%	0,0%	0,0%	0,0%	0,0%	0,0%	0,0%	0,0%	0,0%	Bacteria	Proteobacteria	Betaproteobacteria	Gallionellales	Gallionellaceae	uncultured	-	-	-	-	-	-	-	-	-	-	-	-	-	-	-	-	-	-	-	-	-	-	-	lip	-
619389	0,0%	0,0%	0,0%	0,0%	0,0%	0,0%	0,4%	0,0%	0,0%	Archaea	Crenarchaeota	Thaumarchaeota	Cenarchaeales	Cenarchaeaceae	uncultured archaeon	-	-	-	-	-	-	-	-	-	-	-	-	-	-	-	-	-	-	-	-	-	-	-	-	-
1127748	0,0%	0,0%	0,1%	0,0%	0,0%	0,0%	0,3%	0,0%	0,0%	Bacteria	Proteobacteria	Betaproteobacteria	Burkholderiales	Comamonadaceae	Variovorax	-	napA	narG	nirA	-	-	-	norB	-	-	-	-	ureA	-	-	-	-	-	mmoX	-	-	-	-	-	-
818162	0,3%	0,0%	0,0%	0,1%	0,1%	0,0%	0,0%	0,0%	0,0%	Bacteria	Actinobacteria	Actinobacteria	Actinomycetales	Intrasporangiaceae	Lapillicoccus	-	-	-	-	-	-	-	-	-	-	-	-	-	-	-	-	-	-	-	-	-	-	-	-	-
564026	0,3%	0,0%	0,0%	0,1%	0,0%	0,0%	0,0%	0,0%	0,0%	Bacteria	Firmicutes	Clostridia	Clostridiales	Ruminococcaceae	Incertae Sedis	cellulolytic	-	-	-	-	-	-	-	-	-	-	-	-	-	-	-	-	-	-	-	-	-	-	-	-
4466002	0,0%	0,1%	0,1%	0,0%	0,2%	0,0%	0,0%	0,0%	0,0%	Bacteria	Proteobacteria	Deltaproteobacteria	Myxococcales		uncultured bacterium	-	-	-	-	-	-	-	-	-	-	-	-	-	-	-	-	-	-	-	-	-	-	-	-	-
775178	0,0%	0,1%	0,1%	0,2%	0,0%	0,1%	0,0%	0,3%	0,1%	Bacteria	Proteobacteria	Alphaproteobacteria	Rhodobacterales	Hyphomonadaceae	Hirschia	-	napA	-	-	-	-	-	-	-	-	-	-	ureA	-	-	-	-	-	-	-	laccase_Asco	laccase_Basidio	-	-	ppo
217402	0,0%	0,1%	0,0%	0,2%	0,1%	0,0%	0,0%	0,0%	0,0%	Bacteria	Proteobacteria	Betaproteobacteria	Burkholderiales	Comamonadaceae	Rhizobacter	-	napA	narG	nirA	-	-	-	norB	-	-	-	-	ureA	-	-	-	-	-	-	-	-	-	-	-	-
254127	0,1%	0,0%	0,1%	0,0%	0,1%	0,0%	0,0%	0,0%	0,0%	Bacteria	Proteobacteria	Alphaproteobacteria	Rickettsiales	Rickettsiaceae	Rickettsia	-	-	-	-	-	-	-	-	-	-	-	-	-	-	-	-	-	-	-	-	-	-	-	-	-
235971	0,3%	0,0%	0,0%	0,0%	0,0%	0,0%	0,1%	0,0%	0,0%	Bacteria	Verrucomicrobia	[Spartobacteria]	[Chthoniobacterales]	[Chthoniobacteraceae]	Chthoniobacter	-	napA	-	-	-	-	nirK	-	-	-	-	nrfA	ureA	-	-	-	-	-	-	-	-	-	-	-	-
2474017	0,0%	0,1%	0,2%	0,0%	0,1%	0,0%	0,0%	0,0%	0,0%	Bacteria	Proteobacteria	Deltaproteobacteria	Myxococcales	OM27	OM27 clade	-	-	-	-	-	-	-	-	-	-	-	-	-	-	-	-	-	-	-	-	-	-	-	-	-
1897510	0,0%	0,0%	0,1%	0,0%	0,0%	0,0%	0,2%	0,0%	0,0%	Bacteria	Proteobacteria	Deltaproteobacteria	Myxococcales	Haliangiaceae	Haliangium	-	-	-	-	-	-	-	-	-	-	-	-	ureA	-	-	-	-	chb	-	-	-	-	-	-	-
539488	0,4%	0,0%	0,0%	0,0%	0,0%	0,0%	0,0%	0,0%	0,0%	Bacteria	Proteobacteria	Gammaproteobacteria	[Marinicellales]	[Marinicellaceae]	Marinicella	-	-	-	-	-	-	-	-	-	-	-	-	-	-	-	-	-	-	-	-	-	-	-	-	-
4388545	0,0%	0,0%	0,0%	0,1%	0,0%	0,2%	0,0%	0,2%	1,9%	Bacteria	Proteobacteria	Betaproteobacteria	Burkholderiales	Comamonadaceae	uncultured	-	-	-	-	-	-	-	-	-	-	-	-	-	-	-	-	-	-	-	-	-	-	-	lip	-
156870	0,0%	0,0%	0,0%	0,2%	0,0%	0,0%	0,2%	0,0%	0,0%	Bacteria	Proteobacteria	Deltaproteobacteria	Myxococcales		Sorangium	-	napA	-	-	-	-	nirK	norB	-	-	-	nrfA	ureA	-	-	-	-	chb	-	-	laccase_Asco	laccase_Basidio	-	-	ppo
1086980	0,0%	0,1%	0,0%	0,1%	0,1%	0,1%	0,1%	0,0%	0,0%	Bacteria	Proteobacteria	Deltaproteobacteria	Bdellovibrionales	Bdellovibrionaceae	Bdellovibrio	-	-	-	-	-	-	nirK	norB	-	-	-	nrfA	-	-	-	-	-	-	-	-	laccase_Asco	laccase_Basidio	-	-	ppo
4366206	0,0%	0,1%	0,0%	0,2%	0,0%	0,0%	0,0%	0,2%	0,4%	Bacteria	Proteobacteria	Alphaproteobacteria	Rhizobiales	Hyphomicrobiaceae	Pseudolabrys	-	napA	narG	-	-	-	-	-	nosZ	-	-	-	ureA	-	-	-	-	-	-	-	-	-	-	-	-
4317795	0,0%	0,0%	0,0%	0,0%	0,0%	0,4%	0,2%	0,0%	0,0%	Bacteria	Proteobacteria	Betaproteobacteria	Burkholderiales	Comamonadaceae	uncultured	-	-	-	-	-	-	-	-	-	-	-	-	-	-	-	-	-	-	-	-	-	-	-	lip	-
699114	0,0%	0,0%	0,1%	0,1%	0,0%	0,0%	0,2%	0,0%	0,0%	Bacteria	Proteobacteria	Deltaproteobacteria	Myxococcales		Sandaracinus	-	napA	-	-	-	-	-	-	-	-	-	-	-	-	-	-	-	-	-	-	-	laccase_Basidio	-	-	ppo
1068650	0,0%	0,0%	0,0%	0,0%	0,0%	0,0%	0,4%	0,0%	0,0%	Bacteria	Planctomycetes	Planctomycetia	Gemmatales	Gemmataceae	Gemmata	-	-	-	-	-	-	-	-	-	-	-	-	-	-	-	-	-	-	-	-	-	-	-	-	-
3059360	0,0%	0,1%	0,1%	0,1%	0,1%	0,0%	0,0%	0,2%	0,0%	Bacteria	Proteobacteria	Gammaproteobacteria	Legionellales	Coxiellaceae	uncultured bacterium	-	-	-	-	-	-	-	-	-	-	-	-	-	-	-	-	-	-	-	-	-	-	-	-	-
4457836	0,1%	0,0%	0,0%	0,0%	0,0%	0,0%	0,2%	0,0%	0,0%	Bacteria	Bacteroidetes	[Saprospirae]	[Saprospirales]	Chitinophagaceae	Hydrotalea	-	-	-	-	-	-	-	-	-	-	-	-	-	-	-	-	-	-	-	-	-	-	-	-	-
2068430	0,0%	0,0%	0,1%	0,1%	0,0%	0,0%	0,1%	0,0%	0,1%	Bacteria	Proteobacteria	Betaproteobacteria	Burkholderiales	Comamonadaceae	Chlorochromatium	-	-	-	-	-	-	-	-	-	-	-	-	-	-	-	-	-	-	-	-	-	-	-	-	-
1139646	0,0%	0,1%	0,1%	0,1%	0,0%	0,0%	0,1%	0,0%	0,0%	Bacteria	Bacteroidetes	Flavobacteriia	Flavobacteriales	Flavobacteriaceae	Flavobacterium	cellulolytic	napA	-	nirA	-	-	nirK	norB	nosZ	-	-	-	ureA	-	-	-	-	-	-	-	laccase_Asco	laccase_Basidio	-	-	ppo
4314035	0,0%	0,0%	0,0%	0,1%	0,2%	0,0%	0,0%	0,0%	0,0%	Bacteria	Proteobacteria	Betaproteobacteria	Burkholderiales	Comamonadaceae	Variovorax	-	napA	narG	nirA	-	-	-	norB	-	-	-	-	ureA	-	-	-	-	-	mmoX	-	-	-	-	-	-
728271	0,2%	0,0%	0,0%	0,1%	0,0%	0,1%	0,0%	0,0%	0,0%	Bacteria	Proteobacteria	Deltaproteobacteria			uncultured Desulfuromonadales bacterium	-	-	-	-	-	-	-	-	-	-	-	-	-	-	-	-	-	-	-	-	-	-	-	-	-
3875675	0,0%	0,1%	0,1%	0,2%	0,0%	0,1%	0,0%	0,0%	0,1%	Bacteria	Proteobacteria	Betaproteobacteria	Burkholderiales	Oxalobacteraceae	Massilia	-	napA	narG	-	-	-	-	norB	nosZ	-	-	-	ureA	-	-	-	-	chb	mmoX	-	laccase_Asco	laccase_Basidio	-	-	ppo
721619	0,0%	0,1%	0,1%	0,0%	0,0%	0,0%	0,1%	0,0%	0,0%	Bacteria	Bacteroidetes	Cytophagia	Cytophagales	Cytophagaceae	Cytophaga	cellulolytic	napA	-	-	-	-	-	norB	-	-	nifD	-	ureA	-	-	-	-	-	-	-	-	-	-	-	-
7061	0,0%	0,1%	0,0%	0,1%	0,1%	0,0%	0,1%	0,0%	0,0%	Bacteria	Proteobacteria	Betaproteobacteria	Burkholderiales	Comamonadaceae	Pelomonas	-	napA	narG	nirA	-	nirS	-	norB	nosZ	nifH	-	-	ureA	-	-	-	-	-	-	-	-	-	-	-	-
3692328	0,3%	0,0%	0,0%	0,0%	0,0%	0,0%	0,0%	0,0%	0,0%	Bacteria	Proteobacteria	Alphaproteobacteria	Rhizobiales	Methylocystaceae	uncultured organism	-	-	-	-	-	-	-	-	-	-	-	-	-	-	-	-	-	-	-	-	-	-	-	-	-
4416987	0,0%	0,0%	0,1%	0,2%	0,0%	0,1%	0,0%	0,1%	0,0%	Bacteria	Actinobacteria	Actinobacteria	Actinomycetales	Dermatophilaceae	Piscicoccus	-	-	-	-	-	-	-	-	-	-	-	-	-	-	-	-	-	-	-	-	-	-	-	-	-
3243803	0,0%	0,0%	0,2%	0,0%	0,0%	0,0%	0,0%	0,0%	0,0%	Bacteria	Acidobacteria	DA052	Ellin6513		uncultured bacterium	-	-	-	-	-	-	-	-	-	-	-	-	-	-	-	-	-	-	-	-	-	-	-	-	-
133044	0,0%	0,0%	0,2%	0,2%	0,0%	0,1%	0,0%	0,0%	0,1%	Bacteria	Proteobacteria	Alphaproteobacteria	Sphingomonadales	Sphingomonadaceae	Sphingopyxis	-	napA	-	-	-	-	-	norB	-	-	-	-	-	-	-	-	-	chb	-	-	laccase_Asco	laccase_Basidio	-	-	ppo
4305721	0,0%	0,0%	0,1%	0,1%	0,1%	0,0%	0,0%	0,0%	0,4%	Bacteria	Bacteroidetes	Cytophagia	Cytophagales	Cytophagaceae	uncultured	cellulolytic	-	-	-	-	-	-	-	-	-	-	-	-	-	-	-	-	-	-	-	-	-	-	lip	-
4452317	0,2%	0,0%	0,0%	0,0%	0,0%	0,1%	0,1%	0,0%	0,0%	Bacteria	Acidobacteria	Acidobacteriia	Acidobacteriales	Koribacteraceae	uncultured	-	-	-	-	-	-	-	-	-	-	-	-	-	-	-	-	-	-	-	-	-	-	-	lip	-
4445768	0,2%	0,1%	0,0%	0,0%	0,0%	0,0%	0,0%	0,1%	0,0%	Bacteria	Bacteroidetes	[Saprospirae]	[Saprospirales]	Chitinophagaceae	uncultured	-	-	-	-	-	-	-	-	-	-	-	-	-	-	-	-	-	-	-	-	-	-	-	lip	-
4484176	0,0%	0,2%	0,1%	0,2%	0,1%	0,0%	0,0%	0,0%	0,0%	Bacteria	Actinobacteria	Actinobacteria	Actinomycetales	Microbacteriaceae	Salinibacterium	cellulolytic	-	-	-	-	-	-	-	-	-	-	-	-	-	-	-	-	-	-	-	-	-	-	-	-
77966	0,0%	0,0%	0,1%	0,0%	0,1%	0,0%	0,1%	0,1%	0,0%	Archaea	Euryarchaeota	Methanobacteria	Methanobacteriales	Methanobacteriaceae	Methanobacterium	-	napA	-	-	-	-	-	-	-	nifH	nifD	-	-	-	-	-	-	-	-	-	-	-	-	-	-
591299	0,0%	0,1%	0,1%	0,1%	0,1%	0,0%	0,1%	0,0%	0,0%	Bacteria	Planctomycetes	Planctomycetia	Planctomycetales	Planctomycetaceae	Schlesneria	-	-	-	-	-	-	-	-	-	-	-	-	-	-	-	-	-	-	-	-	-	-	-	-	-
541424	0,0%	0,0%	0,0%	0,0%	0,0%	0,0%	0,2%	0,1%	0,4%	Bacteria	Proteobacteria	Betaproteobacteria	Methylophilales	Methylophilaceae	OM43 clade	-	-	-	-	-	-	-	-	-	-	-	-	-	-	-	-	-	-	-	-	-	-	-	-	-
369289	0,0%	0,0%	0,2%	0,1%	0,0%	0,0%	0,0%	0,0%	0,0%	Bacteria	Actinobacteria	Actinobacteria	Actinomycetales	Micromonosporaceae	Actinoplanes	cellulolytic	napA	narG	-	-	-	nirK	-	-	-	-	-	ureA	-	-	-	-	-	-	-	laccase_Asco	laccase_Basidio	-	-	ppo
846892	0,2%	0,0%	0,1%	0,0%	0,0%	0,0%	0,0%	0,0%	0,0%	Bacteria	Verrucomicrobia	[Pedosphaerae]	[Pedosphaerales]	R4-41B	uncultured Acidobacteria bacterium	-	-	-	-	-	-	-	-	-	-	-	-	-	-	-	-	-	-	-	-	-	-	-	-	-
332569	0,0%	0,0%	0,0%	0,1%	0,0%	0,6%	0,1%	0,0%	0,4%	Bacteria	Proteobacteria	Betaproteobacteria	Burkholderiales	Comamonadaceae	Massilia	-	napA	narG	-	-	-	-	norB	nosZ	-	-	-	ureA	-	-	-	-	chb	mmoX	-	laccase_Asco	laccase_Basidio	-	-	ppo
749696	0,0%	0,1%	0,0%	0,0%	0,0%	0,6%	0,0%	0,0%	0,1%	Bacteria	Proteobacteria	Alphaproteobacteria	Sphingomonadales	Sphingomonadaceae	Novosphingobium	-	napA	-	-	-	-	-	norB	-	nifH	nifD	-	ureA	-	-	-	-	-	mmoX	-	laccase_Asco	laccase_Basidio	-	-	ppo
4418570	0,0%	0,1%	0,1%	0,0%	0,0%	0,0%	0,0%	0,0%	0,0%	Bacteria	Bacteroidetes	[Saprospirae]	[Saprospirales]	Chitinophagaceae	uncultured	-	-	-	-	-	-	-	-	-	-	-	-	-	-	-	-	-	-	-	-	-	-	-	lip	-
3735930	0,1%	0,0%	0,1%	0,1%	0,0%	0,1%	0,0%	0,1%	0,0%	Bacteria	Proteobacteria	Gammaproteobacteria			Acidiferrobacter	-	-	-	-	-	-	-	-	-	nifH	-	-	-	-	-	-	-	-	-	-	-	-	-	-	-
1121753	0,0%	0,2%	0,1%	0,2%	0,0%	0,0%	0,0%	0,0%	0,0%	Bacteria	Firmicutes	Clostridia	Clostridiales	Peptostreptococcaceae	Incertae Sedis	-	-	-	-	-	-	-	-	-	-	-	-	-	-	-	-	-	-	-	-	-	-	-	-	-
212151	0,3%	0,0%	0,0%	0,0%	0,0%	0,0%	0,0%	0,0%	0,0%	Bacteria	Proteobacteria	Alphaproteobacteria	Ellin329		Rhizomicrobium	-	-	-	-	-	-	-	-	-	-	-	-	-	-	-	-	-	-	-	-	-	-	-	-	-
111824	0,0%	0,0%	0,0%	0,1%	0,0%	0,1%	0,2%	0,1%	0,1%	Bacteria	Proteobacteria	Betaproteobacteria	Burkholderiales	Burkholderiaceae	Massilia	-	napA	narG	-	-	-	-	norB	nosZ	-	-	-	ureA	-	-	-	-	chb	mmoX	-	laccase_Asco	laccase_Basidio	-	-	ppo
4091428	0,0%	0,1%	0,1%	0,1%	0,1%	0,0%	0,0%	0,1%	0,0%	Bacteria	Proteobacteria	Betaproteobacteria	Methylophilales		uncultured	-	-	-	-	-	-	-	-	-	-	-	-	-	-	-	-	-	-	-	-	-	-	-	lip	-
842848	0,2%	0,0%	0,0%	0,0%	0,0%	0,2%	0,0%	0,0%	0,0%	Bacteria	Armatimonadetes	Armatimonadia	FW68		uncultured bacterium	-	-	-	-	-	-	-	-	-	-	-	-	-	-	-	-	-	-	-	-	-	-	-	-	-
1126297	0,0%	0,1%	0,0%	0,0%	0,1%	0,0%	0,1%	0,0%	0,0%	Bacteria	Bacteroidetes	Sphingobacteriia	Sphingobacteriales	Sphingobacteriaceae	Pedobacter	-	napA	-	nirA	-	-	-	-	nosZ	-	-	-	ureA	-	-	-	-	chb	-	-	laccase_Asco	laccase_Basidio	-	-	ppo
4354562	0,0%	0,1%	0,0%	0,0%	0,0%	0,0%	0,1%	0,0%	0,0%	Bacteria	Proteobacteria	Alphaproteobacteria	Rhizobiales		Bosea	-	napA	narG	-	-	-	nirK	-	nosZ	nifH	-	-	ureA	-	-	-	-	-	-	-	-	-	-	-	ppo
633535	0,1%	0,1%	0,0%	0,1%	0,0%	0,1%	0,1%	0,0%	0,0%	Bacteria	Proteobacteria	Gammaproteobacteria	Legionellales	Coxiellaceae	Aquicella	-	-	-	-	-	-	-	-	-	-	-	-	-	-	-	-	-	-	-	-	-	-	-	-	-
527906	0,0%	0,1%	0,1%	0,0%	0,1%	0,1%	0,0%	0,0%	0,0%	Bacteria	Proteobacteria	Alphaproteobacteria	Rhizobiales	Rhizobiaceae	Kaistia	-	-	-	-	-	-	-	-	-	-	-	-	-	-	-	-	-	-	-	-	-	-	-	-	-
8028	0,0%	0,0%	0,1%	0,1%	0,1%	0,0%	0,1%	0,0%	0,0%	Bacteria	Proteobacteria	Gammaproteobacteria	Legionellales	Coxiellaceae	Diplorickettsia	-	-	-	-	-	-	-	-	-	-	-	-	-	-	-	-	-	-	-	-	-	-	-	-	-
3658133	0,0%	0,1%	0,1%	0,0%	0,1%	0,2%	0,0%	0,1%	0,0%	Bacteria	Planctomycetes	Planctomycetia	Gemmatales	Gemmataceae	Gemmata	-	-	-	-	-	-	-	-	-	-	-	-	-	-	-	-	-	-	-	-	-	-	-	-	-
1108343	0,0%	0,1%	0,1%	0,1%	0,1%	0,0%	0,0%	0,0%	0,3%	Bacteria	Firmicutes	Bacilli	Bacillales	[Exiguobacteraceae]	Exiguobacterium	-	napA	-	-	-	-	-	-	nosZ	-	-	-	-	-	-	-	-	-	-	-	laccase_Asco	laccase_Basidio	-	-	ppo
516569	0,0%	0,0%	0,0%	0,1%	0,1%	0,3%	0,0%	0,1%	0,1%	Bacteria	Verrucomicrobia	Verrucomicrobiae	Verrucomicrobiales	Verrucomicrobiaceae	Prosthecobacter	-	-	-	-	-	-	-	-	-	-	-	-	-	-	-	-	-	-	-	-	-	-	-	-	-
1137563	0,0%	0,1%	0,0%	0,0%	0,0%	0,1%	0,1%	0,0%	0,0%	Bacteria	Verrucomicrobia	Opitutae	Opitutales	Opitutaceae	Opitutus	-	napA	-	nirA	-	-	-	-	nosZ	-	-	nrfA	ureA	-	-	-	-	-	-	-	-	laccase_Basidio	-	-	-
4337253	0,0%	0,0%	0,1%	0,1%	0,1%	0,0%	0,0%	0,0%	0,1%	Bacteria	Proteobacteria	Gammaproteobacteria	Legionellales	Legionellaceae	Legionella	-	-	-	-	-	nirS	-	norB	-	-	-	-	-	-	-	-	-	-	-	-	laccase_Asco	laccase_Basidio	-	-	ppo
740317	0,0%	0,0%	0,0%	0,0%	0,0%	0,0%	0,3%	0,0%	0,0%	Bacteria	Proteobacteria	Alphaproteobacteria	Rhizobiales	Bradyrhizobiaceae	Bradyrhizobium	-	napA	narG	nirA	-	nirS	nirK	norB	nosZ	nifH	nifD	-	ureA	-	-	SoxB	-	-	mmoX	-	laccase_Asco	laccase_Basidio	-	-	ppo
625652	0,0%	0,0%	0,0%	0,5%	0,0%	0,0%	0,0%	0,0%	0,9%	Bacteria	Proteobacteria	Alphaproteobacteria	Rhizobiales		uncultured bacterium	-	-	-	-	-	-	-	-	-	-	-	-	-	-	-	-	-	-	-	-	-	-	-	-	-
702015	0,0%	0,0%	0,0%	0,0%	0,0%	0,0%	0,2%	0,0%	0,0%	Bacteria	Bacteroidetes	Sphingobacteriia	Sphingobacteriales		uncultured bacterium	-	-	-	-	-	-	-	-	-	-	-	-	-	-	-	-	-	-	-	-	-	-	-	-	-
547217	0,0%	0,0%	0,0%	0,2%	0,0%	0,0%	0,1%	0,0%	0,1%	Bacteria	Planctomycetes	Planctomycetia	Gemmatales	Isosphaeraceae	Isosphaera	-	napA	-	-	-	-	-	-	-	-	-	-	-	-	-	-	-	-	-	-	-	-	-	-	-

4467949	0,0%	0,0%	0,0%	0,2%	0,1%	0,0%	0,0%	0,0%	0,0%	Bacteria	Planctomycetes	Planctomycetia	Pirellulales	Pirellulaceae	uncultured	-	-	-	-	-	-	-	-	-	-	-	-	-	-	-	-	-	-	-	-	-	-	-	lip	-
4444965	0,0%	0,0%	0,0%	0,0%	0,0%	0,0%	0,2%	0,0%	0,0%	Bacteria	Proteobacteria	Deltaproteobacteria	Myxococcales		Sorangium	-	napA	-	-	-	-	nirK	norB	-	-	-	nrfA	ureA	-	-	-	-	chb	-	-	laccase_Asco	laccase_Basidio	-	-	ppo
279180	0,0%	0,0%	0,2%	0,1%	0,0%	0,0%	0,0%	0,0%	0,0%	Bacteria	Proteobacteria	Gammaproteobacteria	Legionellales	Coxiellaceae	uncultured bacterium	-	-	-	-	-	-	-	-	-	-	-	-	-	-	-	-	-	-	-	-	-	-	-	-	-
284168	0,0%	0,2%	0,0%	0,1%	0,0%	0,0%	0,0%	0,0%	0,4%	Bacteria	Verrucomicrobia	Opitutae	Opitutales	Opitutaceae	Opitutus	-	napA	-	nirA	-	-	-	-	nosZ	-	-	nrfA	ureA	-	-	-	-	-	-	-	-	laccase_Basidio	-	-	-
245588	0,0%	0,1%	0,0%	0,0%	0,0%	0,0%	0,2%	0,0%	0,0%	Bacteria	Proteobacteria	Gammaproteobacteria	Oceanospirillales	Oceanospirillaceae	Neptunomonas	-	-	-	-	-	-	-	-	-	-	-	-	-	-	-	-	-	-	-	-	-	-	-	-	-
206386	0,1%	0,0%	0,0%	0,0%	0,0%	0,0%	0,2%	0,0%	0,0%	Bacteria	Proteobacteria	Deltaproteobacteria	Myxococcales		uncultured proteobacterium	-	-	-	-	-	-	-	-	-	-	-	-	-	-	-	-	-	-	-	-	-	-	-	-	-
4354415	0,0%	0,0%	0,0%	0,0%	0,0%	0,0%	0,0%	0,8%	0,1%	Bacteria	Proteobacteria	Alphaproteobacteria	Rhodobacterales	Rhodobacteraceae	Tabrizicola	-	-	-	-	-	-	-	-	-	-	-	-	-	-	-	-	-	-	-	-	-	-	-	-	-
4417470	0,0%	0,1%	0,0%	0,0%	0,0%	0,0%	0,2%	0,0%	0,0%	Bacteria	Proteobacteria	Betaproteobacteria	Methylophilales		uncultured bacterium	-	-	-	-	-	-	-	-	-	-	-	-	-	-	-	-	-	-	-	-	-	-	-	-	-
946052	0,0%	0,1%	0,0%	0,1%	0,0%	0,1%	0,0%	0,2%	0,0%	Bacteria	Proteobacteria	Alphaproteobacteria	Rhodospirillales	Acetobacteraceae	Roseomonas	-	napA	-	-	-	-	-	-	-	-	nifD	-	ureA	-	-	-	-	-	-	-	-	laccase_Basidio	-	-	-
3526720	0,0%	0,1%	0,1%	0,1%	0,1%	0,0%	0,0%	0,0%	0,0%	Bacteria	Proteobacteria	Betaproteobacteria	Burkholderiales	Oxalobacteraceae	Herbaspirillum	-	napA	narG	-	-	nirS	nirK	norB	nosZ	nifH	nifD	-	ureA	-	-	-	-	-	-	-	-	laccase_Basidio	-	-	-
929281	0,1%	0,0%	0,1%	0,0%	0,0%	0,0%	0,0%	0,0%	0,1%	Bacteria	Bacteroidetes	Sphingobacteriia	Sphingobacteriales		uncultured bacterium	-	-	-	-	-	-	-	-	-	-	-	-	-	-	-	-	-	-	-	-	-	-	-	-	-
160433	0,0%	0,1%	0,0%	0,0%	0,0%	0,3%	0,0%	0,2%	0,1%	Bacteria	Chloroflexi	Ktedonobacteria	Thermogemmatisporales	Thermogemmatisporaceae	uncultured Chloroflexi bacterium	-	-	-	-	-	-	-	-	-	-	-	-	-	-	-	-	-	-	-	-	-	-	-	-	-
552725	0,0%	0,1%	0,1%	0,1%	0,0%	0,1%	0,0%	0,0%	0,1%	Bacteria	Proteobacteria	Alphaproteobacteria	Rhodospirillales	Rhodospirillaceae	uncultured alpha proteobacterium	-	-	-	-	-	-	-	-	-	-	-	-	-	-	-	-	-	-	-	-	-	-	-	-	-
1099772	0,0%	0,1%	0,1%	0,0%	0,1%	0,0%	0,0%	0,1%	0,0%	Bacteria	Proteobacteria	Alphaproteobacteria	Rhodospirillales	Acetobacteraceae	uncultured	-	-	-	-	-	-	-	-	-	-	-	-	-	-	-	-	-	-	-	-	-	-	-	lip	-
815293	0,0%	0,0%	0,0%	0,0%	0,0%	0,0%	0,3%	0,0%	0,0%	Bacteria	Proteobacteria	Alphaproteobacteria	Rhodobacterales	Rhodobacteraceae	Rhodobacter	-	napA	-	nirA	-	nirS	nirK	norB	nosZ	nifH	nifD	-	ureA	-	-	SoxB	-	-	mmoX	-	laccase_Asco	laccase_Basidio	-	-	ppo
825612	0,2%	0,0%	0,0%	0,0%	0,0%	0,0%	0,1%	0,0%	0,0%	Bacteria	Proteobacteria	Alphaproteobacteria	Rhizobiales	Methylocystaceae	uncultured Methylocystaceae bacterium	-	-	-	-	-	-	-	-	-	-	-	-	-	-	-	-	-	-	-	-	-	-	-	-	-
111553	0,0%	0,0%	0,0%	0,0%	0,0%	0,0%	0,2%	0,0%	0,0%	Bacteria	Proteobacteria	Alphaproteobacteria	Rhizobiales	Bradyrhizobiaceae	Bradyrhizobium	-	napA	narG	nirA	-	nirS	nirK	norB	nosZ	nifH	nifD	-	ureA	-	-	SoxB	-	-	mmoX	-	laccase_Asco	laccase_Basidio	-	-	ppo
809880	0,3%	0,0%	0,0%	0,0%	0,0%	0,0%	0,0%	0,0%	0,0%	Bacteria	Acidobacteria	Acidobacteriia	Acidobacteriales	Acidobacteriaceae	Granulicella	cellulolytic	napA	-	-	-	-	-	-	-	-	-	-	-	-	-	-	-	-	-	-	laccase_Asco	laccase_Basidio	-	-	ppo
109275	0,3%	0,0%	0,0%	0,0%	0,0%	0,0%	0,0%	0,0%	0,0%	Bacteria	Proteobacteria	Deltaproteobacteria	Desulfobacterales	Desulfobulbaceae	Desulfobulbus	-	napA	-	-	-	-	-	-	-	nifH	nifD	nrfA	-	dsrA	dsrB	-	-	-	-	-	-	-	-	-	-
511320	0,0%	0,0%	0,0%	0,0%	0,0%	0,2%	0,2%	0,0%	0,0%	Bacteria	Bacteroidetes	[Saprospirae]	[Saprospirales]	Chitinophagaceae	uncultured	-	-	-	-	-	-	-	-	-	-	-	-	-	-	-	-	-	-	-	-	-	-	-	lip	-
452324	0,0%	0,1%	0,1%	0,0%	0,1%	0,0%	0,0%	0,0%	0,1%	Bacteria	Verrucomicrobia	[Spartobacteria]	[Chthoniobacterales]	[Chthoniobacteraceae]	Chthoniobacter	-	napA	-	-	-	-	nirK	-	-	-	-	nrfA	ureA	-	-	-	-	-	-	-	-	-	-	-	-
89151	0,0%	0,0%	0,1%	0,0%	0,0%	0,1%	0,0%	0,0%	0,1%	Bacteria	Cyanobacteria	4C0d-2	MLE1-12		uncultured bacterium	-	-	-	-	-	-	-	-	-	-	-	-	-	-	-	-	-	-	-	-	-	-	-	-	-
1105528	0,2%	0,0%	0,0%	0,0%	0,0%	0,0%	0,0%	0,0%	0,0%	Bacteria	Proteobacteria	Deltaproteobacteria	Bdellovibrionales	Bdellovibrionaceae	Bdellovibrio	-	-	-	-	-	-	nirK	norB	-	-	-	nrfA	-	-	-	-	-	-	-	-	laccase_Asco	laccase_Basidio	-	-	ppo
819038	0,0%	0,0%	0,0%	0,0%	0,2%	0,0%	0,0%	0,0%	0,0%	Bacteria	Proteobacteria	Deltaproteobacteria	Myxococcales	Haliangiaceae	Haliangium	-	-	-	-	-	-	-	-	-	-	-	-	ureA	-	-	-	-	chb	-	-	-	-	-	-	-
221410	0,0%	0,3%	0,0%	0,0%	0,0%	0,0%	0,1%	0,0%	0,1%	Bacteria	Proteobacteria	Deltaproteobacteria	Myxococcales		uncultured bacterium	-	-	-	-	-	-	-	-	-	-	-	-	-	-	-	-	-	-	-	-	-	-	-	-	-
221411	0,0%	0,1%	0,1%	0,0%	0,0%	0,0%	0,0%	0,0%	0,0%	Bacteria	Armatimonadetes	Armatimonadia	Armatimonadales	Armatimonadaceae	uncultured bacterium	-	-	-	-	-	-	-	-	-	-	-	-	-	-	-	-	-	-	-	-	-	-	-	-	-
915240	0,0%	0,1%	0,0%	0,0%	0,0%	0,0%	0,1%	0,0%	0,0%	Bacteria	Actinobacteria	Actinobacteria	Actinomycetales	Kineosporiaceae	Kineosporia	-	-	-	-	-	-	-	-	-	-	-	-	-	-	-	-	-	-	-	-	-	-	-	-	-
358745	0,2%	0,0%	0,0%	0,1%	0,0%	0,0%	0,0%	0,1%	0,0%	Bacteria	Proteobacteria	Alphaproteobacteria	Sphingomonadales	Sphingomonadaceae	uncultured bacterium	-	-	-	-	-	-	-	-	-	-	-	-	-	-	-	-	-	-	-	-	-	-	-	-	-
807782	0,1%	0,0%	0,0%	0,1%	0,0%	0,0%	0,0%	0,0%	0,0%	Bacteria	Proteobacteria	Alphaproteobacteria	Rhodospirillales	Acetobacteraceae	Acidocella	-	napA	narG	nirA	-	-	-	-	-	-	-	-	ureA	-	-	-	-	-	-	-	laccase_Asco	laccase_Basidio	-	-	ppo
592584	0,2%	0,1%	0,0%	0,0%	0,0%	0,0%	0,0%	0,0%	0,1%	Bacteria	Proteobacteria	Deltaproteobacteria	NKB15		uncultured	-	-	-	-	-	-	-	-	-	-	-	-	-	-	-	-	-	-	-	-	-	-	-	lip	-
736099	0,0%	0,0%	0,0%	0,0%	0,0%	0,0%	0,2%	0,0%	0,0%	Bacteria	Bacteroidetes	Sphingobacteriia	Sphingobacteriales	Sphingobacteriaceae	Mucilaginibacter	-	-	-	-	-	-	-	-	-	-	-	-	-	-	-	-	-	-	-	-	-	-	-	-	-
832652	0,0%	0,0%	0,0%	0,0%	0,0%	0,0%	0,2%	0,0%	0,0%	Bacteria	Actinobacteria	Actinobacteria	Actinomycetales	Intrasporangiaceae	Terracoccus	-	-	-	-	-	-	-	-	-	-	-	-	-	-	-	-	-	-	-	-	-	-	-	-	-
535922	0,3%	0,0%	0,0%	0,0%	0,0%	0,0%	0,0%	0,0%	0,0%	Bacteria	Proteobacteria	Deltaproteobacteria	Myxococcales		Haliangium	-	-	-	-	-	-	-	-	-	-	-	-	ureA	-	-	-	-	chb	-	-	-	-	-	-	-
150283	0,0%	0,0%	0,0%	0,0%	0,1%	0,2%	0,0%	0,1%	0,0%	Bacteria	Proteobacteria	Alphaproteobacteria			uncultured bacterium	-	-	-	-	-	-	-	-	-	-	-	-	-	-	-	-	-	-	-	-	-	-	-	-	-
4424785	0,0%	0,0%	0,0%	0,0%	0,0%	0,0%	0,2%	0,0%	0,0%	Bacteria	Acidobacteria	Acidobacteria-6	iii1-15		uncultured bacterium	-	-	-	-	-	-	-	-	-	-	-	-	-	-	-	-	-	-	-	-	-	-	-	-	-
4424782	0,2%	0,0%	0,0%	0,0%	0,0%	0,0%	0,0%	0,0%	0,0%	Bacteria	Acidobacteria	Acidobacteriia	Acidobacteriales	Koribacteraceae	uncultured	-	-	-	-	-	-	-	-	-	-	-	-	-	-	-	-	-	-	-	-	-	-	-	lip	-
1133107	0,0%	0,0%	0,0%	0,1%	0,1%	0,0%	0,1%	0,0%	0,0%	Bacteria	Firmicutes	Clostridia	Clostridiales	Ruminococcaceae	Incertae Sedis	cellulolytic	-	-	-	-	-	-	-	-	-	-	-	-	-	-	-	-	-	-	-	-	-	-	-	-
4322804	0,0%	0,0%	0,0%	0,0%	0,0%	0,0%	0,2%	0,0%	0,0%	Bacteria	Bacteroidetes	Cytophagia	Cytophagales	Cytophagaceae	uncultured	cellulolytic	-	-	-	-	-	-	-	-	-	-	-	-	-	-	-	-	-	-	-	-	-	-	lip	-
558848	0,0%	0,0%	0,1%	0,0%	0,1%	0,0%	0,1%	0,0%	0,0%	Bacteria	Proteobacteria	Betaproteobacteria	Neisseriales	Neisseriaceae	Chitinibacter	-	-	-	-	-	-	-	-	-	-	-	-	-	-	-	-	-	-	-	-	-	-	-	-	-
2006401	0,1%	0,0%	0,1%	0,0%	0,0%	0,0%	0,0%	0,0%	0,0%	Bacteria	Planctomycetes	Planctomycetia	Pirellulales	Pirellulaceae	Pirellula	-	napA	-	-	-	-	-	norB	-	-	-	-	-	-	-	-	-	-	-	-	-	-	-	-	-
4466150	0,2%	0,0%	0,0%	0,0%	0,0%	0,0%	0,0%	0,0%	0,0%	Bacteria	Proteobacteria	Gammaproteobacteria	Pasteurellales	Pasteurellaceae	Aggregatibacter	-	napA	-	-	-	-	-	norB	-	-	-	nrfA	-	-	-	-	-	-	-	-	-	-	-	-	-
4455864	0,0%	0,0%	0,0%	0,0%	0,0%	0,0%	0,2%	0,0%	0,0%	Bacteria	Proteobacteria	Gammaproteobacteria			Beggiatoa	-	napA	narG	-	nxrB	nirS	-	norB	-	-	nifD	-	ureA	-	-	-	-	-	mmoX	-	-	-	-	-	ppo
4465431	0,0%	0,0%	0,0%	0,0%	0,0%	0,0%	0,1%	0,1%	0,0%	Bacteria	Proteobacteria	Alphaproteobacteria	Rhizobiales		uncultured	-	-	-	-	-	-	-	-	-	-	-	-	-	-	-	-	-	-	-	-	-	-	-	lip	-
1082607	0,0%	0,0%	0,0%	0,0%	0,0%	0,0%	0,2%	0,0%	0,0%	Bacteria	Actinobacteria	Actinobacteria	Actinomycetales	Corynebacteriaceae	uncultured	-	-	-	-	-	-	-	-	-	-	-	-	-	-	-	-	-	-	-	-	-	-	-	lip	-
165338	0,0%	0,1%	0,1%	0,0%	0,0%	0,0%	0,1%	0,0%	0,0%	Bacteria	Proteobacteria	Gammaproteobacteria	Legionellales	Legionellaceae	Legionella	-	-	-	-	-	nirS	-	norB	-	-	-	-	-	-	-	-	-	-	-	-	laccase_Asco	laccase_Basidio	-	-	ppo
4479101	0,0%	0,0%	0,0%	0,0%	0,0%	0,3%	0,1%	0,0%	0,0%	Bacteria	Proteobacteria	Alphaproteobacteria	Sphingomonadales	Sphingomonadaceae	Novosphingobium	-	napA	-	-	-	-	-	norB	-	nifH	nifD	-	ureA	-	-	-	-	-	mmoX	-	laccase_Asco	laccase_Basidio	-	-	ppo
4475688	0,0%	0,0%	0,0%	0,0%	0,2%	0,0%	0,0%	0,0%	0,1%	Bacteria	Proteobacteria	Betaproteobacteria	Rhodocyclales	Rhodocyclaceae	uncultured beta proteobacterium	-	-	-	-	-	-	-	-	-	-	-	-	-	-	-	-	-	-	-	-	-	-	-	-	-
624830	0,0%	0,0%	0,2%	0,0%	0,0%	0,0%	0,0%	0,0%	0,0%	Bacteria	Proteobacteria	Alphaproteobacteria	Caulobacterales	Caulobacteraceae	uncultured	-	-	-	-	-	-	-	-	-	-	-	-	-	-	-	-	-	-	-	-	-	-	-	lip	-
4382580	0,1%	0,0%	0,0%	0,1%	0,1%	0,0%	0,0%	0,0%	0,0%	Bacteria	Bacteroidetes	Sphingobacteriia	Sphingobacteriales	Sphingobacteriaceae	Mucilaginibacter	-	-	-	-	-	-	-	-	-	-	-	-	-	-	-	-	-	-	-	-	-	-	-	-	-
3240295	0,0%	0,0%	0,1%	0,1%	0,0%	0,0%	0,0%	0,0%	0,0%	Bacteria	Chloroflexi	Ktedonobacteria	Thermogemmatisporales	Thermogemmatisporaceae	uncultured Ktedonobacter sp.	-	-	-	-	-	-	-	-	-	-	-	-	-	-	-	-	-	-	-	-	-	-	-	-	-
4383662	0,1%	0,0%	0,0%	0,0%	0,0%	0,0%	0,0%	0,1%	0,0%	Bacteria	Planctomycetes	Planctomycetia	Gemmatales	Gemmataceae	uncultured	-	-	-	-	-	-	-	-	-	-	-	-	-	-	-	-	-	-	-	-	-	-	-	lip	-
1136974	0,0%	0,0%	0,1%	0,0%	0,0%	0,0%	0,1%	0,0%	0,1%	Bacteria	Proteobacteria	Alphaproteobacteria	Sphingomonadales	Sphingomonadaceae	Novosphingobium	-	napA	-	-	-	-	-	norB	-	nifH	nifD	-	ureA	-	-	-	-	-	mmoX	-	laccase_Asco	laccase_Basidio	-	-	ppo
854368	0,0%	0,0%	0,0%	0,0%	0,1%	0,0%	0,0%	0,0%	0,0%	Bacteria	Actinobacteria	Acidimicrobiia	Acidimicrobiales	EB1017	uncultured bacterium	-	-	-	-	-	-	-	-	-	-	-	-	-	-	-	-	-	-	-	-	-	-	-	-	-
870470	0,0%	0,0%	0,0%	0,0%	0,0%	0,0%	0,1%	0,3%	0,0%	Bacteria	Proteobacteria	Alphaproteobacteria	Rhizobiales		uncultured	-	-	-	-	-	-	-	-	-	-	-	-	-	-	-	-	-	-	-	-	-	-	-	lip	-
4423331	0,1%	0,0%	0,0%	0,1%	0,0%	0,1%	0,0%	0,1%	0,0%	Bacteria	Proteobacteria	Betaproteobacteria	Burkholderiales	Oxalobacteraceae	uncultured bacterium	-	-	-	-	-	-	-	-	-	-	-	-	-	-	-	-	-	-	-	-	-	-	-	-	-
2753316	0,0%	0,0%	0,0%	0,0%	0,0%	0,0%	0,2%	0,0%	0,0%	Bacteria	Actinobacteria	Acidimicrobiia	Acidimicrobiales		uncultured bacterium	-	-	-	-	-	-	-	-	-	-	-	-	-	-	-	-	-	-	-	-	-	-	-	-	-
141216	0,0%	0,0%	0,0%	0,0%	0,0%	0,7%	0,0%	0,0%	0,3%	Bacteria	Actinobacteria	Actinobacteria	Actinomycetales		Acidothermus	-	napA	narG	-	-	-	nirK	-	-	-	-	-	-	-	-	-	-	-	-	-	-	-	-	-	-
4466827	0,0%	0,1%	0,1%	0,0%	0,0%	0,0%	0,0%	0,0%	0,0%	Bacteria	Proteobacteria	Betaproteobacteria	Burkholderiales	Oxalobacteraceae	Achromobacter	-	napA	narG	-	-	nirS	nirK	norB	nosZ	-	-	nrfA	ureA	-	-	-	-	-	mmoX	-	laccase_Asco	laccase_Basidio	-	-	ppo
588684	0,0%	0,0%	0,0%	0,2%	0,1%	0,0%	0,0%	0,0%	0,3%	Bacteria	Planctomycetes	Planctomycetia	Gemmatales	Gemmataceae	Gemmata	-	-	-	-	-	-	-	-	-	-	-	-	-	-	-	-	-	-	-	-	-	-	-	-	-
205391	0,2%	0,0%	0,0%	0,0%	0,0%	0,1%	0,0%	0,0%	0,1%	Bacteria	Acidobacteria	Solibacteres	Solibacterales		Bryobacter	-	-	-	-	-	-	-	-	-	-	-	-	-	-	-	-	-	-	-	-	-	-	-	-	-
36221	0,0%	0,0%	0,0%	0,1%	0,1%	0,0%	0,0%	0,0%	0,0%	Bacteria	Proteobacteria	Gammaproteobacteria	Legionellales	Coxiellaceae	Aquicella	-	-	-	-	-	-	-	-	-	-	-	-	-	-	-	-	-	-	-	-	-	-	-	-	-
53994	0,0%	0,0%	0,1%	0,0%	0,0%	0,0%	0,0%	0,0%	0,0%	Bacteria	Proteobacteria	Alphaproteobacteria	Rhodospirillales	Acetobacteraceae	Acidocella	-	napA	narG	nirA	-	-	-	-	-	-	-	-	ureA	-	-	-	-	-	-	-	laccase_Asco	laccase_Basidio	-	-	ppo
141219	0,0%	0,1%	0,1%	0,0%	0,0%	0,1%	0,0%	0,0%	0,0%	Bacteria	Spirochaetes	Spirochaetes	Spirochaetales	Spirochaetaceae	Spirochaeta	-	-	-	nirA	-	-	-	-	-	nifH	nifD	-	-	-	-	-	-	-	-	-	-	-	-	-	-
4333971	0,0%	0,0%	0,1%	0,1%	0,0%	0,0%	0,0%	0,0%	0,0%	Bacteria	Planctomycetes	Planctomycetia	Gemmatales	Gemmataceae	Gemmata	-	-	-	-	-	-	-	-	-	-	-	-	-	-	-	-	-	-	-	-	-	-	-	-	-
1134737	0,0%	0,4%	0,0%	0,0%	0,0%	0,0%	0,0%	0,0%	0,0%	Bacteria	Proteobacteria	Betaproteobacteria	Neisseriales	Neisseriaceae	Deefgea	-	-	-	-	-	-	-	-	-	-	-	-	-	-	-	-	-	-	-	-	-	-	-	-	-
341698	0,0%	0,0%	0,1%	0,0%	0,0%	0,0%	0,0%	0,0%	0,1%	Bacteria	Verrucomicrobia	Verrucomicrobiae	Verrucomicrobiales	Verrucomicrobiaceae	uncultured	-	-	-	-	-	-	-	-	-	-	-	-	-	-	-	-	-	-	-	-	-	-	-	lip	-
325796	0,0%	0,0%	0,2%	0,0%	0,0%	0,0%	0,0%	0,0%	0,1%	Bacteria	Chloroflexi	TK10	B07_WMSP1	FFCH4570	uncultured Chloroflexi bacterium	-	-	-	-	-	-	-	-	-	-	-	-	-	-	-	-	-	-	-	-	-	-	-	-	-
754628	0,0%	0,0%	0,1%	0,1%	0,0%	0,2%	0,0%	0,0%	0,0%	Bacteria	Proteobacteria	Alphaproteobacteria	Rhizobiales	Methylocystaceae	uncultured type II methanotroph	-	-	-	-	-	-	-	-	-	-	-	-	-	-	-	-	-	-	-	-	-	-	-	-	-
578951	0,0%	0,1%	0,0%	0,1%	0,1%	0,0%	0,0%	0,0%	0,0%	Bacteria	Gemmatimonadetes	Gemmatimonadetes	Gemmatimonadales	Gemmatimonadaceae	Gemmatimonas	-	napA	-	-	-	-	nirK	norB	nosZ	-	-	nrfA	-	dsrA	dsrB	-	-	chb	-	-	-	-	-	-	-
1137001	0,0%	0,0%	0,1%	0,2%	0,0%	0,0%	0,0%	0,1%	0,1%	Bacteria	Proteobacteria	Betaproteobacteria	Rhodocyclales	Rhodocyclaceae	Denitratisoma	-	-	-	-	-	-	-	-	-	-	-	-	-	-	-	-	-	-	-	-	-	-	-	-	-
43037	0,0%	0,0%	0,0%	0,0%	0,0%	0,0%	0,2%	0,0%	0,0%	Bacteria	Proteobacteria	Alphaproteobacteria	Rhodospirillales	Acetobacteraceae	Rhodopila	-	-	-	-	-	-	-	-	-	-	-	-	-	-	-	-	-	-	-	-	-	-	-	-	-
4312075	0,0%	0,1%	0,0%	0,0%	0,0%	0,0%	0,1%	0,0%	0,0%	Bacteria	Bacteroidetes	[Saprospirae]	[Saprospirales]	Saprospiraceae	uncultured	-	-	-	-	-	-	-	-	-	-	-	-	-	-	-	-	-	-	-	-	-	-	-	lip	-
4451011	0,2%	0,0%	0,0%	0,0%	0,0%	0,0%	0,0%	0,0%	0,0%	Bacteria	Proteobacteria	Gammaproteobacteria	Pseudomonadales	Pseudomonadaceae	Pseudomonas	cellulolytic	napA	narG	nirA	-	nirS	nirK	norB	nosZ	nifH	nifD	-	ureA	-	dsrB	SoxB	-	-	mmoX	-	laccase_Asco	laccase_Basidio	-	-	ppo
4326875	0,0%	0,1%	0,1%	0,1%	0,0%	0,0%	0,0%	0,0%	0,0%	Bacteria	Chlamydiae	Chlamydiia	Chlamydiales		uncultured bacterium	-	-	-	-	-	-	-	-	-	-	-	-	-	-	-	-	-	-	-	-	-	-	-	-	-
4460976	0,0%	0,0%	0,0%	0,1%	0,0%	0,3%	0,0%	0,0%	0,0%	Bacteria	Proteobacteria	Betaproteobacteria	Thiobacterales		uncultured	-	-	-	-	-	-	-	-	-	-	-	-	-	-	-	-	-	-	-	-	-	-	-	lip	-
4451018	0,0%	0,0%	0,1%	0,1%	0,0%	0,1%	0,0%	0,0%	0,0%	Bacteria	Proteobacteria	Betaproteobacteria	Burkholderiales	Comamonadaceae	Variovorax	-	napA	narG	nirA	-	-	-	norB	-	-	-	-	ureA	-	-	-	-	-	mmoX	-	-	-	-	-	-
4216839	0,0%	0,0%	0,0%	0,0%	0,1%	0,1%	0,0%	0,1%	0,0%	Bacteria	Proteobacteria	Gammaproteobacteria	Legionellales	Legionellaceae	Legionella	-	-	-	-	-	nirS	-	norB	-	-	-	-	-	-	-	-	-	-	-	-	laccase_Asco	laccase_Basidio	-	-	ppo
744839	0,0%	0,0%	0,0%	0,0%	0,0%	0,0%	0,2%	0,0%	0,0%	Bacteria	Bacteroidetes	[Saprospirae]	[Saprospirales]	Chitinophagaceae	Chitinophaga	-	napA	-	-	-	-	-	norB	-	-	-	-	ureA	-	-	-	-	chb	-	-	laccase_Asco	laccase_Basidio	-	-	ppo
4329003	0,0%	0,1%	0,1%	0,0%	0,1%	0,0%	0,0%	0,0%	0,1%	Bacteria	Proteobacteria	Deltaproteobacteria	MIZ46		uncultured bacterium	-	-	-	-	-	-	-	-	-	-	-	-	-	-	-	-	-	-	-	-	-	-	-	-	-
4472017	0,0%	0,0%	0,1%	0,1%	0,0%	0,1%	0,0%	0,0%	0,0%	Bacteria	Proteobacteria	Betaproteobacteria	Burkholderiales	Comamonadaceae	Limnohabitans	-	napA	narG	-	-	-	-	-	-	-	-	-	ureA	-	-	-	-	-	-	-	-	-	-	-	-
4462710	0,0%	0,1%	0,0%	0,0%	0,0%	0,0%	0,1%	0,1%	0,0%	Bacteria	Proteobacteria	Alphaproteobacteria	Rhizobiales	Hyphomicrobiaceae	Devosia	-	napA	-	-	-	-	nirK	-	-	nifH	-	-	ureA	-	-	-	-	chb	-	-	laccase_Asco	laccase_Basidio	-	-	ppo
2595164	0,0%	0,0%	0,0%	0,0%	0,2%	0,0%	0,0%	0,0%	0,0%	Bacteria	Actinobacteria	Actinobacteria	Actinomycetales	Pseudonocardiaceae	Pseudonocardia	cellulolytic	napA	narG	-	-	-	-	-	-	-	-	-	ureA	-	-	-	-	-	mmoX	-	-	laccase_Basidio	-	-	-
659078	0,0%	0,0%	0,0%	0,0%	0,0%	0,2%	0,1%	0,0%	0,0%	Bacteria	Bacteroidetes	Cytophagia	Cytophagales	Cytophagaceae	Arcicella	cellulolytic	-	-	-	-	-	-	-	-	-	-	-	-	-	-	-	-	-	-	-	-	-	-	-	-
239066	0,1%	0,0%	0,1%	0,0%	0,0%	0,0%	0,0%	0,0%	0,1%	Bacteria	Proteobacteria	Deltaproteobacteria	Bdellovibrionales	Bacteriovoracaceae	Peredibacter	-	-	-	-	-	-	-	-	-	-	-	-	-	-	-	-	-	-	-	-	-	-	-	-	-
665869	0,0%	0,0%	0,0%	0,1%	0,0%	0,1%	0,1%	0,0%	0,0%	Bacteria	Bacteroidetes	[Saprospirae]	[Saprospirales]	Chitinophagaceae	Ferruginibacter	-	-	-	-	-	-	-	-	-	-	-	-	-	-	-	-	-	-	-	-	-	-	-	-	-
265028	0,0%	0,0%	0,0%	0,0%	0,0%	0,1%	0,1%	0,0%	0,1%	Bacteria	Proteobacteria	Alphaproteobacteria	Kordiimonadales	Kordiimonadaceae	Kordiimonas	-	-	-	-	-	-	-	-	-	-	-	-	-	-	-	-	-	-	-	-	-	-	-	-	-
669896	0,0%	0,1%	0,1%	0,0%	0,0%	0,0%	0,0%	0,1%	0,0%	Bacteria	Proteobacteria	Betaproteobacteria	Rhodocyclales	Rhodocyclaceae	Uliginosibacterium	-	-	-	-	-	-	-	-	-	-	-	-	-	-	-	-	-	-	-	-	-	-	-	-	-
70005	0,0%	0,0%	0,0%	0,0%	0,0%	0,0%	0,2%	0,0%	0,0%	Bacteria	Proteobacteria	Alphaproteobacteria	Caulobacterales	Caulobacteraceae	Asticcacaulis	-	napA	narG	nirA	-	-	-	-	-	-	-	-	-	-	-	-	-	chb	-	-	laccase_Asco	laccase_Basidio	-	-	ppo
1009482	0,0%	0,2%	0,0%	0,0%	0,1%	0,0%	0,0%	0,0%	0,0%	Bacteria	Proteobacteria	Gammaproteobacteria	Legionellales	Legionellaceae	Legionella	-	-	-	-	-	nirS	-	norB	-	-	-	-	-	-	-	-	-	-	-	-	laccase_Asco	laccase_Basidio	-	-	ppo
125736	0,0%	0,0%	0,0%	0,0%	0,0%	0,1%	0,1%	0,0%	0,0%	Bacteria	Verrucomicrobia	[Pedosphaerae]	[Pedosphaerales]	auto67_4W	uncultured Verrucomicrobia bacterium	-	-	-	-	-	-	-	-	-	-	-	-	-	-	-	-	-	-	-	-	-	-	-	-	-
202686	0,0%	0,1%	0,1%	0,0%	0,0%	0,0%	0,0%	0,0%	0,0%	Bacteria	Planctomycetes	Planctomycetia	Gemmatales	Gemmataceae	uncultured	-	-	-	-	-	-	-	-	-	-	-	-	-	-	-	-	-	-	-	-	-	-	-	lip	-
833876	0,2%	0,0%	0,0%	0,0%	0,0%	0,0%	0,0%	0,0%	0,0%	Bacteria	Proteobacteria	Gammaproteobacteria	Aeromonadales	Aeromonadaceae	uncultured bacterium	-	-	-	-	-	-	-	-	-	-	-	-	-	-	-	-	-	-	-	-	-	-	-	-	-
4414775	0,1%	0,0%	0,0%	0,0%	0,0%	0,1%	0,0%	0,0%	0,1%	Bacteria	Proteobacteria	Alphaproteobacteria	Rhizobiales	Hyphomicrobiaceae	Rhodoplanes	-	-	-	-	-	-	-	-	-	-	-	-	-	-	-	-	-	-	-	-	-	-	-	-	-
4451942	0,0%	0,0%	0,0%	0,0%	0,0%	0,0%	0,1%	0,0%	0,0%	Bacteria	Acidobacteria	Acidobacteriia	Acidobacteriales	Koribacteraceae	uncultured	-	-	-	-	-	-	-	-	-	-	-	-	-	-	-	-	-	-	-	-	-	-	-	lip	-
320742	0,1%	0,0%	0,0%	0,1%	0,0%	0,0%	0,0%	0,0%	0,0%	Bacteria	Bacteroidetes	[Saprospirae]	[Saprospirales]		uncultured	-	-	-	-	-	-	-	-	-	-	-	-	-	-	-	-	-	-	-	-	-	-	-	lip	-
1123984	0,0%	0,0%	0,0%	0,1%	0,0%	0,0%	0,1%	0,0%	0,1%	Bacteria	Acidobacteria	DA052	Ellin6513		uncultured bacterium	-	-	-	-	-	-	-	-	-	-	-	-	-	-	-	-	-	-	-	-	-	-	-	-	-
4344472	0,0%	0,1%	0,0%	0,1%	0,0%	0,1%	0,0%	0,1%	0,0%	Bacteria	Acidobacteria	Holophagae	Holophagales	Holophagaceae	Holophaga	-	napA	-	-	-	-	-	-	-	nifH	nifD	nrfA	-	-	-	-	-	-	-	-	-	-	-	-	-
107461	0,0%	0,0%	0,1%	0,1%	0,0%	0,1%	0,0%	0,1%	0,1%	Bacteria	Proteobacteria	Alphaproteobacteria	Rhizobiales		uncultured	-	-	-	-	-	-	-	-	-	-	-	-	-	-	-	-	-	-	-	-	-	-	-	lip	-
62406	0,0%	0,1%	0,0%	0,0%	0,0%	0,0%	0,1%	0,0%	0,0%	Bacteria	Proteobacteria	Alphaproteobacteria	Rhizobiales	Bradyrhizobiaceae	Bradyrhizobium	-	napA	narG	nirA	-	nirS	nirK	norB	nosZ	nifH	nifD	-	ureA	-	-	SoxB	-	-	mmoX	-	laccase_Asco	laccase_Basidio	-	-	ppo
564301	0,0%	0,1%	0,0%	0,0%	0,0%	0,0%	0,0%	0,0%	0,0%	Bacteria	Proteobacteria	Gammaproteobacteria	Xanthomonadales	Sinobacteraceae	uncultured bacterium	-	-	-	-	-	-	-	-	-	-	-	-	-	-	-	-	-	-	-	-	-	-	-	-	-
4395995	0,0%	0,0%	0,1%	0,0%	0,0%	0,0%	0,1%	0,1%	0,0%	Bacteria	Proteobacteria	Betaproteobacteria	Burkholderiales	Comamonadaceae	Paucibacter	-	-	-	-	-	-	-	-	-	-	-	-	-	-	-	-	-	-	-	-	-	-	-	-	-
3902438	0,2%	0,0%	0,0%	0,0%	0,0%	0,0%	0,0%	0,0%	0,1%	Bacteria	Actinobacteria	Actinobacteria	Actinomycetales	Microbacteriaceae	Pseudoclavibacter	cellulolytic	-	-	-	-	-	-	-	-	-	-	-	-	-	-	-	-	-	-	-	-	-	-	-	-
208090	0,0%	0,0%	0,0%	0,1%	0,1%	0,0%	0,0%	0,1%	0,0%	Bacteria	Proteobacteria	Gammaproteobacteria	Xanthomonadales	Sinobacteraceae	uncultured bacterium	-	-	-	-	-	-	-	-	-	-	-	-	-	-	-	-	-	-	-	-	-	-	-	-	-
1129210	0,0%	0,0%	0,1%	0,0%	0,0%	0,1%	0,0%	0,0%	0,0%	Bacteria	Acidobacteria	Acidobacteriia	Acidobacteriales	Koribacteraceae	uncultured	-	-	-	-	-	-	-	-	-	-	-	-	-	-	-	-	-	-	-	-	-	-	-	lip	-
1114968	0,0%	0,0%	0,1%	0,0%	0,0%	0,0%	0,0%	0,0%	0,0%	Bacteria	Proteobacteria	Betaproteobacteria	Rhodocyclales	Rhodocyclaceae	uncultured	-	-	-	-	-	-	-	-	-	-	-	-	-	-	-	-	-	-	-	-	-	-	-	lip	-
4462155	0,0%	0,0%	0,1%	0,1%	0,0%	0,3%	0,0%	0,0%	0,0%	Bacteria	Proteobacteria	Betaproteobacteria	Burkholderiales	Comamonadaceae	Xylophilus	-	napA	-	nirA	-	-	-	-	-	-	-	-	ureA	-	-	-	-	-	-	-	-	-	-	-	-
149057	0,1%	0,1%	0,0%	0,0%	0,0%	0,1%	0,0%	0,0%	0,1%	Bacteria	Proteobacteria	Gammaproteobacteria	[Marinicellales]	[Marinicellaceae]	Marinicella	-	-	-	-	-	-	-	-	-	-	-	-	-	-	-	-	-	-	-	-	-	-	-	-	-
221896	0,0%	0,0%	0,0%	0,0%	0,0%	0,0%	0,2%	0,0%	0,0%	Bacteria	Verrucomicrobia	[Spartobacteria]	[Chthoniobacterales]	[Chthoniobacteraceae]	Candidatus Xiphinematobacter	-	-	-	-	-	-	-	-	-	-	-	-	-	-	-	-	-	-	-	-	-	-	-	-	-
4434025	0,0%	0,1%	0,0%	0,1%	0,1%	0,1%	0,0%	0,0%	0,0%	Bacteria	Proteobacteria	Betaproteobacteria			uncultured	-	-	-	-	-	-	-	-	-	-	-	-	-	-	-	-	-	-	-	-	-	-	-	lip	-
4456889	0,0%	0,0%	0,1%	0,0%	0,0%	0,0%	0,0%	0,1%	0,0%	Bacteria	Proteobacteria	Gammaproteobacteria	Pseudomonadales	Pseudomonadaceae	Pseudomonas	cellulolytic	napA	narG	nirA	-	nirS	nirK	norB	nosZ	nifH	nifD	-	ureA	-	dsrB	SoxB	-	-	mmoX	-	laccase_Asco	laccase_Basidio	-	-	ppo
4337815	0,1%	0,0%	0,0%	0,0%	0,0%	0,1%	0,0%	0,1%	0,0%	Bacteria	Firmicutes	Clostridia	Clostridiales	Clostridiaceae	Clostridium sensu stricto 1	cellulolytic	-	-	-	-	-	-	-	-	-	-	-	-	-	-	-	-	-	-	-	-	-	-	-	-
1004666	0,2%	0,0%	0,0%	0,0%	0,0%	0,0%	0,0%	0,0%	0,0%	Bacteria	Proteobacteria	Deltaproteobacteria	Bdellovibrionales	Bdellovibrionaceae	Bdellovibrio	-	-	-	-	-	-	nirK	norB	-	-	-	nrfA	-	-	-	-	-	-	-	-	laccase_Asco	laccase_Basidio	-	-	ppo
236994	0,0%	0,0%	0,0%	0,0%	0,0%	0,0%	0,2%	0,0%	0,0%	Bacteria	Proteobacteria	Deltaproteobacteria	Myxococcales		uncultured bacterium	-	-	-	-	-	-	-	-	-	-	-	-	-	-	-	-	-	-	-	-	-	-	-	-	-
827343	0,0%	0,0%	0,0%	0,0%	0,0%	0,1%	0,1%	0,0%	0,0%	Bacteria	Proteobacteria	Alphaproteobacteria	Rhodospirillales	Acetobacteraceae	uncultured	-	-	-	-	-	-	-	-	-	-	-	-	-	-	-	-	-	-	-	-	-	-	-	lip	-
2815573	0,0%	0,0%	0,0%	0,0%	0,1%	0,2%	0,0%	0,0%	0,0%	Bacteria	Bacteroidetes	Sphingobacteriia	Sphingobacteriales		uncultured bacterium	-	-	-	-	-	-	-	-	-	-	-	-	-	-	-	-	-	-	-	-	-	-	-	-	-
739867	0,2%	0,0%	0,0%	0,0%	0,0%	0,0%	0,0%	0,0%	0,0%	Bacteria	Actinobacteria	Actinobacteria	Actinomycetales	Microbacteriaceae	Lysinimonas	cellulolytic	-	-	-	-	-	-	-	-	-	-	-	-	-	-	-	-	-	-	-	-	-	-	-	-
1136510	0,1%	0,0%	0,0%	0,1%	0,0%	0,0%	0,1%	0,0%	0,0%	Bacteria	Proteobacteria	Betaproteobacteria	Burkholderiales	Comamonadaceae	uncultured	-	-	-	-	-	-	-	-	-	-	-	-	-	-	-	-	-	-	-	-	-	-	-	lip	-
2087663	0,2%	0,0%	0,0%	0,0%	0,0%	0,0%	0,0%	0,0%	0,0%	Bacteria	Proteobacteria	Betaproteobacteria	Neisseriales	Neisseriaceae	uncultured	-	-	-	-	-	-	-	-	-	-	-	-	-	-	-	-	-	-	-	-	-	-	-	lip	-
196652	0,0%	0,0%	0,0%	0,1%	0,0%	0,0%	0,2%	0,0%	0,0%	Bacteria	Proteobacteria	Betaproteobacteria	Burkholderiales	Burkholderiaceae	Burkholderia	-	napA	narG	nirA	-	nirS	nirK	norB	nosZ	nifH	nifD	-	ureA	-	-	-	-	chb	mmoX	-	laccase_Asco	laccase_Basidio	-	-	ppo
18546	0,0%	0,0%	0,2%	0,0%	0,0%	0,0%	0,0%	0,0%	0,0%	Bacteria	Proteobacteria	Alphaproteobacteria	Rhodospirillales	Rhodospirillaceae	Inquilinus	-	-	-	-	-	-	-	-	-	-	-	-	-	-	-	-	-	-	-	-	-	-	-	-	-
760601	0,0%	0,0%	0,0%	0,0%	0,0%	0,3%	0,0%	0,2%	0,0%	Bacteria	Proteobacteria	Alphaproteobacteria	Rhodospirillales	Acetobacteraceae	uncultured	-	-	-	-	-	-	-	-	-	-	-	-	-	-	-	-	-	-	-	-	-	-	-	lip	-
1028297	0,0%	0,0%	0,1%	0,0%	0,0%	0,0%	0,0%	0,0%	0,0%	Bacteria	Verrucomicrobia	[Spartobacteria]	[Chthoniobacterales]	[Chthoniobacteraceae]	Chthoniobacter	-	napA	-	-	-	-	nirK	-	-	-	-	nrfA	ureA	-	-	-	-	-	-	-	-	-	-	-	-
2963709	0,0%	0,0%	0,0%	0,0%	0,0%	0,0%	0,0%	0,6%	0,0%	Bacteria	Proteobacteria	Deltaproteobacteria	Myxococcales		uncultured bacterium	-	-	-	-	-	-	-	-	-	-	-	-	-	-	-	-	-	-	-	-	-	-	-	-	-
1088618	0,0%	0,1%	0,0%	0,0%	0,1%	0,0%	0,1%	0,1%	0,0%	Bacteria	Proteobacteria	Alphaproteobacteria	Caulobacterales	Caulobacteraceae	Phenylobacterium	-	napA	narG	-	-	-	nirK	norB	-	-	-	-	-	-	-	-	-	-	-	-	laccase_Asco	laccase_Basidio	-	-	ppo
805571	0,0%	0,0%	0,0%	0,0%	0,1%	0,0%	0,1%	0,0%	0,0%	Bacteria	Proteobacteria	Betaproteobacteria	Rhodocyclales	Rhodocyclaceae	Denitratisoma	-	-	-	-	-	-	-	-	-	-	-	-	-	-	-	-	-	-	-	-	-	-	-	-	-
1124268	0,0%	0,1%	0,0%	0,0%	0,0%	0,0%	0,0%	0,0%	0,0%	Bacteria	Proteobacteria	Betaproteobacteria	Burkholderiales	Comamonadaceae	Acidovorax	-	napA	narG	nirA	-	nirS	nirK	norB	nosZ	-	-	-	ureA	-	-	-	-	-	mmoX	-	-	laccase_Basidio	-	-	ppo
611757	0,0%	0,0%	0,1%	0,1%	0,0%	0,0%	0,0%	0,0%	0,0%	Bacteria	Proteobacteria	Alphaproteobacteria	Rhodobacterales	Rhodobacteraceae	Sulfitobacter	-	napA	narG	-	-	-	-	-	nosZ	nifH	-	-	ureA	-	-	-	-	-	-	-	laccase_Asco	laccase_Basidio	-	-	ppo
4311072	0,0%	0,1%	0,1%	0,0%	0,1%	0,0%	0,0%	0,0%	0,0%	Bacteria	Actinobacteria	Thermoleophilia	Solirubrobacterales	Patulibacteraceae	Patulibacter	-	napA	narG	-	-	-	-	-	-	-	-	-	ureA	-	-	-	-	-	mmoX	-	-	-	-	-	-
1087462	0,0%	0,3%	0,0%	0,0%	0,0%	0,0%	0,0%	0,0%	0,1%	Bacteria	Bacteroidetes	Cytophagia	Cytophagales	Cytophagaceae	Cytophaga	cellulolytic	napA	-	-	-	-	-	norB	-	-	nifD	-	ureA	-	-	-	-	-	-	-	-	-	-	-	-
4415229	0,0%	0,0%	0,0%	0,0%	0,1%	0,3%	0,0%	0,0%	0,0%	Bacteria	Bacteroidetes	[Saprospirae]	[Saprospirales]	Chitinophagaceae	uncultured	-	-	-	-	-	-	-	-	-	-	-	-	-	-	-	-	-	-	-	-	-	-	-	lip	-
4389974	0,1%	0,0%	0,0%	0,0%	0,0%	0,0%	0,0%	0,0%	0,0%	Bacteria	Acidobacteria	Acidobacteria-6	iii1-15	RB40	uncultured Acidobacteria bacterium	-	-	-	-	-	-	-	-	-	-	-	-	-	-	-	-	-	-	-	-	-	-	-	-	-
113331	0,0%	0,0%	0,1%	0,0%	0,1%	0,0%	0,0%	0,0%	0,0%	Bacteria	Acidobacteria	DA052	Ellin6513		uncultured forest soil bacterium	-	-	-	-	-	-	-	-	-	-	-	-	-	-	-	-	-	-	-	-	-	-	-	-	-
1062936	0,0%	0,1%	0,0%	0,0%	0,1%	0,0%	0,0%	0,0%	0,0%	Bacteria	Actinobacteria	Acidimicrobiia	Acidimicrobiales		uncultured	-	-	-	-	-	-	-	-	-	-	-	-	-	-	-	-	-	-	-	-	-	-	-	lip	-
612937	0,1%	0,0%	0,0%	0,0%	0,0%	0,0%	0,0%	0,0%	0,1%	Bacteria	Proteobacteria	Alphaproteobacteria	BD7-3		uncultured bacterium	-	-	-	-	-	-	-	-	-	-	-	-	-	-	-	-	-	-	-	-	-	-	-	-	-
202619	0,0%	0,0%	0,0%	0,0%	0,0%	0,0%	0,2%	0,0%	0,0%	Bacteria	Verrucomicrobia	Verrucomicrobiae	Verrucomicrobiales	Verrucomicrobiaceae	uncultured	-	-	-	-	-	-	-	-	-	-	-	-	-	-	-	-	-	-	-	-	-	-	-	lip	-
3946733	0,0%	0,0%	0,0%	0,0%	0,1%	0,0%	0,0%	0,0%	0,0%	Bacteria	Bacteroidetes	Cytophagia	Cytophagales	Cytophagaceae	Spirosoma	cellulolytic	napA	-	nirA	-	-	-	-	-	-	-	-	-	-	-	-	-	chb	-	-	laccase_Asco	laccase_Basidio	-	-	ppo
76374	0,0%	0,0%	0,0%	0,1%	0,0%	0,4%	0,0%	0,0%	0,1%	Bacteria	Gemmatimonadetes	Gemmatimonadetes	KD8-87		uncultured	-	-	-	-	-	-	-	-	-	-	-	-	-	-	-	-	-	-	-	-	-	-	-	lip	-
996068	0,1%	0,0%	0,0%	0,0%	0,0%	0,1%	0,0%	0,0%	0,0%	Bacteria	Cyanobacteria	4C0d-2	YS2		uncultured bacterium	-	-	-	-	-	-	-	-	-	-	-	-	-	-	-	-	-	-	-	-	-	-	-	-	-
837424	0,0%	0,0%	0,0%	0,0%	0,0%	0,0%	0,0%	0,1%	0,0%	Bacteria	Proteobacteria	Alphaproteobacteria	Rhodobacterales	Hyphomonadaceae	Hirschia	-	napA	-	-	-	-	-	-	-	-	-	-	ureA	-	-	-	-	-	-	-	laccase_Asco	laccase_Basidio	-	-	ppo
538346	0,2%	0,0%	0,0%	0,0%	0,0%	0,0%	0,0%	0,0%	0,0%	Bacteria	Bacteroidetes	[Saprospirae]	[Saprospirales]	Saprospiraceae	uncultured	-	-	-	-	-	-	-	-	-	-	-	-	-	-	-	-	-	-	-	-	-	-	-	lip	-
813980	0,0%	0,0%	0,0%	0,0%	0,0%	0,0%	0,2%	0,0%	0,0%	Bacteria	Proteobacteria	Alphaproteobacteria	Rhizobiales		Nitratireductor	-	napA	-	nirA	-	-	nirK	-	nosZ	nifH	-	-	ureA	-	-	-	-	-	-	-	laccase_Asco	laccase_Basidio	-	-	ppo
360253	0,0%	0,1%	0,0%	0,0%	0,1%	0,0%	0,0%	0,1%	0,3%	Bacteria	Proteobacteria	Alphaproteobacteria	Sphingomonadales	Sphingomonadaceae	Novosphingobium	-	napA	-	-	-	-	-	norB	-	nifH	nifD	-	ureA	-	-	-	-	-	mmoX	-	laccase_Asco	laccase_Basidio	-	-	ppo
733893	0,0%	0,0%	0,0%	0,1%	0,0%	0,2%	0,0%	0,1%	0,0%	Bacteria	Proteobacteria	Alphaproteobacteria	Rhizobiales	Rhizobiaceae	Rhizobium	-	napA	narG	nirA	-	-	nirK	norB	nosZ	nifH	nifD	nrfA	ureA	-	-	-	-	chb	mmoX	-	laccase_Asco	laccase_Basidio	-	-	ppo
54353	0,0%	0,0%	0,0%	0,0%	0,0%	0,0%	0,2%	0,0%	0,0%	Bacteria	Proteobacteria	Alphaproteobacteria	Caulobacterales	Caulobacteraceae	uncultured	-	-	-	-	-	-	-	-	-	-	-	-	-	-	-	-	-	-	-	-	-	-	-	lip	-
150347	0,0%	0,0%	0,1%	0,1%	0,0%	0,0%	0,0%	0,1%	0,0%	Bacteria	Proteobacteria	Alphaproteobacteria	Caulobacterales	Caulobacteraceae	Phenylobacterium	-	napA	narG	-	-	-	nirK	norB	-	-	-	-	-	-	-	-	-	-	-	-	laccase_Asco	laccase_Basidio	-	-	ppo
4472794	0,0%	0,1%	0,0%	0,0%	0,1%	0,0%	0,0%	0,0%	0,1%	Bacteria	Proteobacteria	Betaproteobacteria	Neisseriales	Neisseriaceae	uncultured	-	-	-	-	-	-	-	-	-	-	-	-	-	-	-	-	-	-	-	-	-	-	-	lip	-
4452146	0,0%	0,0%	0,0%	0,1%	0,0%	0,3%	0,0%	0,0%	0,0%	Bacteria	Firmicutes	Clostridia	Clostridiales	Clostridiaceae	Clostridium sensu stricto 14	cellulolytic	-	-	-	-	-	-	-	-	-	-	-	-	-	-	-	-	-	-	-	-	-	-	-	-
4320961	0,0%	0,0%	0,1%	0,0%	0,1%	0,0%	0,0%	0,0%	0,0%	Bacteria	Chloroflexi	Ktedonobacteria	Thermogemmatisporales	Thermogemmatisporaceae	uncultured Thermosporotrichaceae bacterium	-	-	-	-	-	-	-	-	-	-	-	-	-	-	-	-	-	-	-	-	-	-	-	-	-
4338687	0,0%	0,0%	0,1%	0,0%	0,0%	0,0%	0,0%	0,0%	0,0%	Bacteria	Proteobacteria	Alphaproteobacteria	Rickettsiales	Pelagibacteraceae	uncultured bacterium	-	-	-	-	-	-	-	-	-	-	-	-	-	-	-	-	-	-	-	-	-	-	-	-	-
137669	0,0%	0,1%	0,0%	0,1%	0,1%	0,0%	0,0%	0,0%	0,0%	Bacteria	Proteobacteria	Alphaproteobacteria	Rhizobiales	Methylocystaceae	uncultured organism	-	-	-	-	-	-	-	-	-	-	-	-	-	-	-	-	-	-	-	-	-	-	-	-	-
788779	0,0%	0,0%	0,1%	0,1%	0,0%	0,0%	0,0%	0,0%	0,0%	Bacteria	Actinobacteria	Acidimicrobiia	Acidimicrobiales	Microthrixaceae	Candidatus Microthrix	-	-	-	-	-	-	-	-	-	-	-	-	-	-	-	-	-	-	-	-	-	-	-	-	-
965853	0,0%	0,1%	0,0%	0,1%	0,0%	0,0%	0,1%	0,0%	0,0%	Bacteria	Actinobacteria	Thermoleophilia	Solirubrobacterales		uncultured actinobacterium	-	-	-	-	-	-	-	-	-	-	-	-	-	-	-	-	-	-	-	-	-	-	-	-	-
4416997	0,0%	0,0%	0,0%	0,1%	0,1%	0,0%	0,0%	0,0%	0,0%	Bacteria	Planctomycetes	Phycisphaerae	Phycisphaerales		SM1A02	-	-	-	-	-	-	-	-	-	-	-	-	-	-	-	-	-	-	-	-	-	-	-	-	-
68621	0,0%	0,0%	0,0%	0,1%	0,0%	0,0%	0,0%	0,3%	0,4%	Bacteria	Proteobacteria	Betaproteobacteria	Burkholderiales	Comamonadaceae	Rhizobacter	-	napA	narG	nirA	-	-	-	norB	-	-	-	-	ureA	-	-	-	-	-	-	-	-	-	-	-	-
769365	0,1%	0,0%	0,0%	0,0%	0,0%	0,0%	0,0%	0,0%	0,1%	Bacteria	Proteobacteria	Alphaproteobacteria	Rhodospirillales	Acetobacteraceae	Acidocella	-	napA	narG	nirA	-	-	-	-	-	-	-	-	ureA	-	-	-	-	-	-	-	laccase_Asco	laccase_Basidio	-	-	ppo
849642	0,0%	0,0%	0,0%	0,0%	0,0%	0,0%	0,0%	0,0%	0,4%	Bacteria	Proteobacteria	Betaproteobacteria	Burkholderiales	Comamonadaceae	Aquabacterium	-	-	-	-	-	-	-	-	-	-	-	-	-	-	-	-	-	-	-	-	-	-	-	-	-
4469214	0,0%	0,0%	0,0%	0,0%	0,0%	0,1%	0,0%	0,0%	0,0%	Bacteria	Firmicutes	Bacilli	Bacillales	Bacillaceae	Anoxybacillus	cellulolytic	napA	narG	-	-	-	-	-	nosZ	-	-	nrfA	ureA	-	-	-	-	-	-	-	laccase_Asco	laccase_Basidio	-	-	-
1123475	0,0%	0,0%	0,0%	0,0%	0,0%	0,0%	0,1%	0,0%	0,1%	Bacteria	Acidobacteria	Holophagae	Holophagales	Holophagaceae	uncultured	-	-	-	-	-	-	-	-	-	-	-	-	-	-	-	-	-	-	-	-	-	-	-	lip	-
4417061	0,0%	0,1%	0,0%	0,0%	0,0%	0,0%	0,1%	0,0%	0,0%	Bacteria	Proteobacteria	Alphaproteobacteria	Rhizobiales	Xanthobacteraceae	Azorhizobium	-	napA	narG	nirA	-	-	-	-	-	nifH	nifD	-	ureA	-	-	-	-	-	-	-	laccase_Asco	laccase_Basidio	-	-	ppo
231447	0,0%	0,1%	0,0%	0,1%	0,1%	0,0%	0,0%	0,1%	0,0%	Bacteria	Verrucomicrobia	Opitutae	Opitutales	Opitutaceae	Opitutus	-	napA	-	nirA	-	-	-	-	nosZ	-	-	nrfA	ureA	-	-	-	-	-	-	-	-	laccase_Basidio	-	-	-
778059	0,0%	0,0%	0,1%	0,1%	0,0%	0,0%	0,0%	0,0%	0,0%	Bacteria	Proteobacteria	Gammaproteobacteria	Aeromonadales	Aeromonadaceae	uncultured bacterium	-	-	-	-	-	-	-	-	-	-	-	-	-	-	-	-	-	-	-	-	-	-	-	-	-
934158	0,0%	0,0%	0,0%	0,0%	0,0%	0,0%	0,0%	0,0%	0,0%	Bacteria	Acidobacteria	Acidobacteriia	Acidobacteriales	Acidobacteriaceae	uncultured bacterium	cellulolytic	-	-	-	-	-	-	-	-	-	-	-	-	-	-	-	-	-	-	-	-	-	-	-	-
1118070	0,0%	0,0%	0,1%	0,0%	0,0%	0,0%	0,0%	0,0%	0,0%	Bacteria	Proteobacteria	Gammaproteobacteria	Methylococcales	Crenotrichaceae	Methylovulum	-	-	-	-	-	-	-	-	-	nifH	-	-	-	-	-	-	-	-	mmoX	pmoA	-	-	-	-	-
701112	0,0%	0,0%	0,0%	0,0%	0,1%	0,0%	0,0%	0,0%	0,0%	Bacteria	Proteobacteria	Alphaproteobacteria	Rhizobiales	Beijerinckiaceae	uncultured	-	-	-	-	-	-	-	-	-	-	-	-	-	-	-	-	-	-	-	-	-	-	-	lip	-
512091	0,0%	0,0%	0,0%	0,0%	0,0%	0,0%	0,1%	0,0%	0,0%	Bacteria	Proteobacteria	Deltaproteobacteria	Myxococcales		Sorangium	-	napA	-	-	-	-	nirK	norB	-	-	-	nrfA	ureA	-	-	-	-	chb	-	-	laccase_Asco	laccase_Basidio	-	-	ppo
4319473	0,0%	0,0%	0,0%	0,0%	0,1%	0,0%	0,0%	0,1%	0,1%	Bacteria	Firmicutes	Bacilli	Lactobacillales	Streptococcaceae	Lactococcus	-	-	-	-	-	-	-	-	-	-	-	-	-	-	-	-	-	-	-	-	laccase_Asco	laccase_Basidio	-	-	ppo
4413494	0,0%	0,0%	0,1%	0,0%	0,0%	0,1%	0,0%	0,0%	0,0%	Bacteria	Actinobacteria	Actinobacteria	Actinomycetales	Mycobacteriaceae	Mycobacterium	-	napA	narG	nirA	-	-	-	norB	-	-	-	-	ureA	-	-	-	amoA_AOB	-	mmoX	pmoA	laccase_Asco	laccase_Basidio	-	-	ppo

17991	0,0%	0,0%	0,1%	0,0%	0,0%	0,0%	0,0%	0,0%	0,0%	Bacteria	Actinobacteria	Actinobacteria	Actinomycetales	Micromonosporaceae	Actinoplanes	cellulolytic	napA	narG	-	-	-	nirK	-	-	-	-	-	ureA	-	-	-	-	-	-	-	laccase_Asco	laccase_Basidio	-	-	ppo
222209	0,0%	0,0%	0,1%	0,0%	0,0%	0,0%	0,0%	0,1%	0,0%	Bacteria	Proteobacteria	Alphaproteobacteria	Rhizobiales	Bradyrhizobiaceae	Bradyrhizobium	-	napA	narG	nirA	-	nirS	nirK	norB	nosZ	nifH	nifD	-	ureA	-	-	SoxB	-	-	mmoX	-	laccase_Asco	laccase_Basidio	-	-	ppo
1559	0,0%	0,0%	0,1%	0,0%	0,0%	0,1%	0,0%	0,0%	0,0%	Bacteria	Verrucomicrobia	Opitutae	Opitutales	Opitutaceae	Opitutus	-	napA	-	nirA	-	-	-	-	nosZ	-	-	nrfA	ureA	-	-	-	-	-	-	-	-	laccase_Basidio	-	-	-
4439509	0,0%	0,0%	0,0%	0,0%	0,0%	0,1%	0,0%	0,2%	0,0%	Bacteria	Proteobacteria	Betaproteobacteria	Burkholderiales	Burkholderiaceae	Burkholderia	-	napA	narG	nirA	-	nirS	nirK	norB	nosZ	nifH	nifD	-	ureA	-	-	-	-	chb	mmoX	-	laccase_Asco	laccase_Basidio	-	-	ppo
533261	0,2%	0,0%	0,0%	0,0%	0,0%	0,0%	0,0%	0,0%	0,0%	Bacteria	Verrucomicrobia	Verrucomicrobiae	Verrucomicrobiales	Verrucomicrobiaceae	Roseibacillus	-	-	-	-	-	-	-	-	-	-	-	-	-	-	-	-	-	-	-	-	-	-	-	-	-
4459929	0,1%	0,0%	0,0%	0,0%	0,0%	0,0%	0,0%	0,0%	0,0%	Bacteria	Proteobacteria	Betaproteobacteria	Burkholderiales	Comamonadaceae	Paucibacter	-	-	-	-	-	-	-	-	-	-	-	-	-	-	-	-	-	-	-	-	-	-	-	-	-
4091432	0,0%	0,1%	0,0%	0,0%	0,1%	0,0%	0,0%	0,0%	0,0%	Bacteria	Planctomycetes	Planctomycetia	Gemmatales	Isosphaeraceae	uncultured	-	-	-	-	-	-	-	-	-	-	-	-	-	-	-	-	-	-	-	-	-	-	-	lip	-
78648	0,0%	0,0%	0,1%	0,0%	0,0%	0,1%	0,0%	0,0%	0,0%	Bacteria	Acidobacteria	DA052	Ellin6513		uncultured bacterium	-	-	-	-	-	-	-	-	-	-	-	-	-	-	-	-	-	-	-	-	-	-	-	-	-
222768	0,0%	0,0%	0,0%	0,0%	0,0%	0,0%	0,1%	0,0%	0,0%	Bacteria	Proteobacteria	Alphaproteobacteria	Ellin329		Rhizomicrobium	-	-	-	-	-	-	-	-	-	-	-	-	-	-	-	-	-	-	-	-	-	-	-	-	-
1138828	0,0%	0,0%	0,0%	0,0%	0,0%	0,0%	0,0%	0,1%	0,1%	Bacteria	Proteobacteria	Betaproteobacteria	Burkholderiales	Comamonadaceae	Variovorax	-	napA	narG	nirA	-	-	-	norB	-	-	-	-	ureA	-	-	-	-	-	mmoX	-	-	-	-	-	-
4296290	0,0%	0,0%	0,1%	0,0%	0,1%	0,0%	0,0%	0,0%	0,0%	Bacteria	Proteobacteria	Betaproteobacteria	Burkholderiales	Comamonadaceae	uncultured	-	-	-	-	-	-	-	-	-	-	-	-	-	-	-	-	-	-	-	-	-	-	-	lip	-
4477696	0,0%	0,0%	0,1%	0,0%	0,1%	0,0%	0,0%	0,0%	0,0%	Bacteria	Proteobacteria	Gammaproteobacteria	Pasteurellales	Pasteurellaceae	Haemophilus	-	napA	-	-	-	-	nirK	norB	-	-	-	nrfA	ureA	-	-	-	-	-	-	-	laccase_Asco	laccase_Basidio	-	-	ppo
4478194	0,0%	0,0%	0,0%	0,0%	0,0%	0,1%	0,1%	0,0%	0,3%	Bacteria	Verrucomicrobia	[Spartobacteria]	[Chthoniobacterales]	[Chthoniobacteraceae]	Candidatus Xiphinematobacter	-	-	-	-	-	-	-	-	-	-	-	-	-	-	-	-	-	-	-	-	-	-	-	-	-
4438545	0,1%	0,0%	0,0%	0,0%	0,0%	0,0%	0,0%	0,0%	0,0%	Bacteria	Proteobacteria	Alphaproteobacteria	Sphingomonadales	Sphingomonadaceae	Novosphingobium	-	napA	-	-	-	-	-	norB	-	nifH	nifD	-	ureA	-	-	-	-	-	mmoX	-	laccase_Asco	laccase_Basidio	-	-	ppo
585228	0,0%	0,0%	0,0%	0,0%	0,0%	0,0%	0,2%	0,0%	0,0%	Bacteria	Proteobacteria	Alphaproteobacteria	Rhodospirillales	Rhodospirillaceae	uncultured bacterium	-	-	-	-	-	-	-	-	-	-	-	-	-	-	-	-	-	-	-	-	-	-	-	-	-
4482839	0,1%	0,0%	0,0%	0,0%	0,0%	0,0%	0,0%	0,0%	0,1%	Bacteria	Proteobacteria	Betaproteobacteria	Burkholderiales	Comamonadaceae	uncultured	-	-	-	-	-	-	-	-	-	-	-	-	-	-	-	-	-	-	-	-	-	-	-	lip	-
1123057	0,0%	0,1%	0,1%	0,0%	0,0%	0,0%	0,0%	0,1%	0,0%	Bacteria	Actinobacteria	Acidimicrobiia	Acidimicrobiales	EB1017	uncultured bacterium	-	-	-	-	-	-	-	-	-	-	-	-	-	-	-	-	-	-	-	-	-	-	-	-	-
4481482	0,0%	0,0%	0,0%	0,1%	0,0%	0,0%	0,0%	0,0%	0,0%	Bacteria	Actinobacteria	Actinobacteria	Actinomycetales	Microbacteriaceae	MWH-Ta3	cellulolytic	-	-	-	-	-	-	-	-	-	-	-	-	-	-	-	-	-	-	-	-	-	-	-	-
4444550	0,0%	0,0%	0,0%	0,0%	0,1%	0,2%	0,0%	0,0%	0,0%	Bacteria	Proteobacteria	Deltaproteobacteria	Desulfuromonadales	Geobacteraceae	uncultured bacterium	-	-	-	-	-	-	-	-	-	-	-	-	-	-	-	-	-	-	-	-	-	-	-	-	-
4375292	0,0%	0,0%	0,1%	0,0%	0,1%	0,0%	0,0%	0,0%	0,1%	Bacteria	Proteobacteria	Deltaproteobacteria	MIZ46		uncultured Bacteriovorax sp.	-	-	-	-	-	-	-	-	-	-	-	-	-	-	-	-	-	-	-	-	-	-	-	-	-
82683	0,1%	0,0%	0,0%	0,0%	0,0%	0,0%	0,0%	0,1%	0,0%	Bacteria	Actinobacteria	Actinobacteria	Actinomycetales	Microbacteriaceae	Arthrobacter	cellulolytic	napA	narG	-	-	-	-	-	-	nifH	-	-	ureA	dsrA	-	-	-	-	mmoX	-	laccase_Asco	laccase_Basidio	-	-	ppo
267851	0,0%	0,0%	0,0%	0,0%	0,0%	0,2%	0,0%	0,1%	0,3%	Bacteria	Actinobacteria	Thermoleophilia	Solirubrobacterales	Patulibacteraceae	Patulibacter	-	napA	narG	-	-	-	-	-	-	-	-	-	ureA	-	-	-	-	-	mmoX	-	-	-	-	-	-
4439479	0,0%	0,0%	0,0%	0,0%	0,0%	0,1%	0,0%	0,1%	0,1%	Bacteria	Proteobacteria	Deltaproteobacteria	Myxococcales	Haliangiaceae	Haliangium	-	-	-	-	-	-	-	-	-	-	-	-	ureA	-	-	-	-	chb	-	-	-	-	-	-	-
4454018	0,0%	0,0%	0,1%	0,0%	0,0%	0,0%	0,0%	0,1%	0,0%	Bacteria	Acidobacteria	DA052	Ellin6513		uncultured forest soil bacterium	-	-	-	-	-	-	-	-	-	-	-	-	-	-	-	-	-	-	-	-	-	-	-	-	-
4325081	0,0%	0,0%	0,0%	0,0%	0,0%	0,5%	0,0%	0,0%	0,1%	Bacteria	Actinobacteria	Actinobacteria	Actinomycetales		Kineosporia	-	-	-	-	-	-	-	-	-	-	-	-	-	-	-	-	-	-	-	-	-	-	-	-	-
821712	0,1%	0,0%	0,0%	0,0%	0,0%	0,0%	0,0%	0,1%	0,1%	Bacteria	Proteobacteria	Betaproteobacteria	Methylophilales	Methylophilaceae	Methylotenera	-	napA	-	nirA	-	-	nirK	norB	-	-	-	-	ureA	-	-	-	-	-	-	-	-	-	-	-	-
3276016	0,0%	0,0%	0,0%	0,0%	0,0%	0,1%	0,1%	0,0%	0,0%	Bacteria	Planctomycetes	Planctomycetia	Gemmatales	Gemmataceae	uncultured	-	-	-	-	-	-	-	-	-	-	-	-	-	-	-	-	-	-	-	-	-	-	-	lip	-
4377314	0,0%	0,1%	0,0%	0,0%	0,1%	0,0%	0,0%	0,1%	0,0%	Bacteria	Actinobacteria	Actinobacteria	Actinomycetales	Nocardiaceae	Nocardia	-	napA	narG	-	-	-	nirK	-	-	-	-	-	ureA	-	-	-	-	-	mmoX	-	laccase_Asco	laccase_Basidio	-	-	ppo
663822	0,1%	0,0%	0,0%	0,0%	0,0%	0,0%	0,0%	0,0%	0,0%	Bacteria	Proteobacteria	Gammaproteobacteria	Legionellales	Coxiellaceae	Aquicella	-	-	-	-	-	-	-	-	-	-	-	-	-	-	-	-	-	-	-	-	-	-	-	-	-
1138936	0,0%	0,0%	0,0%	0,0%	0,0%	0,0%	0,1%	0,0%	0,1%	Bacteria	Proteobacteria	Deltaproteobacteria	Myxococcales	Polyangiaceae	Byssovorax	-	-	-	-	-	-	-	-	-	-	-	-	-	-	-	-	-	-	-	-	-	-	-	-	-
4323733	0,0%	0,0%	0,0%	0,0%	0,1%	0,0%	0,0%	0,0%	0,0%	Bacteria	Actinobacteria	Actinobacteria	Actinomycetales	Mycobacteriaceae	Mycobacterium	-	napA	narG	nirA	-	-	-	norB	-	-	-	-	ureA	-	-	-	amoA_AOB	-	mmoX	pmoA	laccase_Asco	laccase_Basidio	-	-	ppo
332410	0,0%	0,0%	0,1%	0,0%	0,0%	0,0%	0,0%	0,0%	0,0%	Bacteria	Proteobacteria	Alphaproteobacteria	Rhizobiales	Methylocystaceae	uncultured bacterium	-	-	-	-	-	-	-	-	-	-	-	-	-	-	-	-	-	-	-	-	-	-	-	-	-
1116547	0,0%	0,0%	0,0%	0,0%	0,0%	0,0%	0,2%	0,0%	0,0%	Bacteria	Actinobacteria	Acidimicrobiia	Acidimicrobiales		uncultured bacterium	-	-	-	-	-	-	-	-	-	-	-	-	-	-	-	-	-	-	-	-	-	-	-	-	-
593096	0,0%	0,1%	0,0%	0,0%	0,1%	0,0%	0,0%	0,0%	0,0%	Bacteria	Proteobacteria	Alphaproteobacteria	Rhodospirillales	Acetobacteraceae	Acidocella	-	napA	narG	nirA	-	-	-	-	-	-	-	-	ureA	-	-	-	-	-	-	-	laccase_Asco	laccase_Basidio	-	-	ppo
11358	0,0%	0,0%	0,0%	0,0%	0,0%	0,0%	0,0%	0,0%	1,1%	Bacteria	Actinobacteria	Thermoleophilia	Solirubrobacterales	Conexibacteraceae	Conexibacter	-	-	narG	-	-	-	-	-	-	-	-	-	ureA	-	-	-	-	-	-	-	-	laccase_Basidio	-	-	-
343557	0,0%	0,0%	0,0%	0,1%	0,1%	0,0%	0,0%	0,0%	0,0%	Bacteria	Proteobacteria	Betaproteobacteria	Burkholderiales	Comamonadaceae	Roseateles	-	-	-	-	-	-	-	-	-	nifH	-	-	-	-	-	-	-	-	-	-	-	-	-	-	-
614651	0,0%	0,0%	0,0%	0,0%	0,0%	0,0%	0,1%	0,0%	0,0%	Bacteria	Proteobacteria	Alphaproteobacteria	Rhizobiales	Rhizobiaceae	Rhizobium	-	napA	narG	nirA	-	-	nirK	norB	nosZ	nifH	nifD	nrfA	ureA	-	-	-	-	chb	mmoX	-	laccase_Asco	laccase_Basidio	-	-	ppo
259155	0,1%	0,0%	0,0%	0,0%	0,0%	0,1%	0,0%	0,0%	0,0%	Bacteria	WS3	PRR-12	Sediment-1	PRR-10	uncultured bacterium	-	-	-	-	-	-	-	-	-	-	-	-	-	-	-	-	-	-	-	-	-	-	-	-	-
159560	0,0%	0,0%	0,1%	0,0%	0,0%	0,0%	0,0%	0,0%	0,0%	Bacteria	Bacteroidetes	Sphingobacteriia	Sphingobacteriales		uncultured bacterium	-	-	-	-	-	-	-	-	-	-	-	-	-	-	-	-	-	-	-	-	-	-	-	-	-
131787	0,0%	0,0%	0,0%	0,1%	0,0%	0,2%	0,0%	0,0%	0,0%	Bacteria	Proteobacteria	Alphaproteobacteria	Rhizobiales		Rhizobium	-	napA	narG	nirA	-	-	nirK	norB	nosZ	nifH	nifD	nrfA	ureA	-	-	-	-	chb	mmoX	-	laccase_Asco	laccase_Basidio	-	-	ppo
4297239	0,0%	0,0%	0,0%	0,1%	0,1%	0,0%	0,0%	0,1%	0,0%	Bacteria	Acidobacteria	DA052	Ellin6513		uncultured eubacterium WD264	-	-	-	-	-	-	-	-	-	-	-	-	-	-	-	-	-	-	-	-	-	-	-	-	-
2232179	0,0%	0,1%	0,0%	0,1%	0,0%	0,0%	0,0%	0,0%	0,0%	Bacteria	Acidobacteria	Solibacteres	Solibacterales		Bryobacter	-	-	-	-	-	-	-	-	-	-	-	-	-	-	-	-	-	-	-	-	-	-	-	-	-
4422848	0,0%	0,0%	0,0%	0,0%	0,0%	0,0%	0,0%	0,1%	0,4%	Bacteria	Proteobacteria	Betaproteobacteria			Denitratisoma	-	-	-	-	-	-	-	-	-	-	-	-	-	-	-	-	-	-	-	-	-	-	-	-	-
22253	0,1%	0,0%	0,0%	0,0%	0,0%	0,0%	0,0%	0,0%	0,1%	Bacteria	Proteobacteria	Gammaproteobacteria	Legionellales	Coxiellaceae	Aquicella	-	-	-	-	-	-	-	-	-	-	-	-	-	-	-	-	-	-	-	-	-	-	-	-	-
4378776	0,0%	0,0%	0,0%	0,1%	0,1%	0,0%	0,0%	0,0%	0,0%	Bacteria	Proteobacteria	Alphaproteobacteria	Rhizobiales	Hyphomicrobiaceae	Devosia	-	napA	-	-	-	-	nirK	-	-	nifH	-	-	ureA	-	-	-	-	chb	-	-	laccase_Asco	laccase_Basidio	-	-	ppo
807799	0,0%	0,0%	0,0%	0,0%	0,0%	0,5%	0,0%	0,0%	0,0%	Bacteria	Proteobacteria	Alphaproteobacteria			uncultured	-	-	-	-	-	-	-	-	-	-	-	-	-	-	-	-	-	-	-	-	-	-	-	lip	-
4445550	0,0%	0,0%	0,0%	0,0%	0,0%	0,0%	0,0%	0,0%	0,0%	Bacteria	Actinobacteria	Actinobacteria	Actinomycetales	Microbacteriaceae	Frigoribacterium	cellulolytic	napA	-	-	-	-	-	-	-	-	-	-	-	-	-	-	-	-	-	-	-	-	-	-	-
4361133	0,0%	0,0%	0,0%	0,0%	0,0%	0,1%	0,0%	0,0%	0,3%	Bacteria	Acidobacteria	Acidobacteria-6	iii1-15		uncultured bacterium	-	-	-	-	-	-	-	-	-	-	-	-	-	-	-	-	-	-	-	-	-	-	-	-	-
750263	0,1%	0,0%	0,0%	0,0%	0,0%	0,0%	0,0%	0,0%	0,0%	Bacteria	Proteobacteria	Alphaproteobacteria	Rhizobiales		uncultured bacterium	-	-	-	-	-	-	-	-	-	-	-	-	-	-	-	-	-	-	-	-	-	-	-	-	-
866187	0,0%	0,0%	0,0%	0,1%	0,0%	0,1%	0,0%	0,0%	0,0%	Bacteria	Proteobacteria	Deltaproteobacteria	Spirobacillales		uncultured bacterium	-	-	-	-	-	-	-	-	-	-	-	-	-	-	-	-	-	-	-	-	-	-	-	-	-
4463853	0,0%	0,0%	0,0%	0,0%	0,0%	0,3%	0,0%	0,0%	0,0%	Bacteria	Proteobacteria	Deltaproteobacteria	Desulfuromonadales	Geobacteraceae	uncultured bacterium	-	-	-	-	-	-	-	-	-	-	-	-	-	-	-	-	-	-	-	-	-	-	-	-	-
100737	0,0%	0,0%	0,0%	0,0%	0,0%	0,0%	0,1%	0,0%	0,0%	Bacteria	Verrucomicrobia	Opitutae	Opitutales	Opitutaceae	Opitutus	-	napA	-	nirA	-	-	-	-	nosZ	-	-	nrfA	ureA	-	-	-	-	-	-	-	-	laccase_Basidio	-	-	-
545460	0,0%	0,0%	0,0%	0,0%	0,1%	0,0%	0,0%	0,0%	0,0%	Bacteria	Actinobacteria	Actinobacteria	Actinomycetales	ACK-M1	hgcI clade	-	-	-	-	-	-	-	-	-	-	-	-	-	-	-	-	-	-	-	-	-	-	-	-	-
4339171	0,0%	0,0%	0,0%	0,0%	0,1%	0,0%	0,0%	0,0%	0,0%	Bacteria	Bacteroidetes	Cytophagia	Cytophagales	Cytophagaceae	Flectobacillus	cellulolytic	-	-	-	-	-	-	-	-	-	-	-	-	-	-	-	-	-	-	-	-	-	-	-	-
249034	0,0%	0,0%	0,0%	0,1%	0,1%	0,0%	0,0%	0,0%	0,1%	Bacteria	Proteobacteria	Alphaproteobacteria			uncultured bacterium	-	-	-	-	-	-	-	-	-	-	-	-	-	-	-	-	-	-	-	-	-	-	-	-	-
136417	0,0%	0,0%	0,0%	0,0%	0,1%	0,0%	0,0%	0,1%	0,1%	Bacteria	Actinobacteria	Actinobacteria	Actinomycetales		Acidothermus	-	napA	narG	-	-	-	nirK	-	-	-	-	-	-	-	-	-	-	-	-	-	-	-	-	-	-
3116300	0,0%	0,0%	0,0%	0,1%	0,1%	0,0%	0,0%	0,0%	0,0%	Bacteria	Proteobacteria	Betaproteobacteria	Methylophilales	Methylophilaceae	uncultured	-	-	-	-	-	-	-	-	-	-	-	-	-	-	-	-	-	-	-	-	-	-	-	lip	-
683530	0,0%	0,0%	0,1%	0,0%	0,1%	0,0%	0,0%	0,0%	0,0%	Bacteria	Bacteroidetes	Cytophagia	Cytophagales	Cytophagaceae	uncultured	cellulolytic	-	-	-	-	-	-	-	-	-	-	-	-	-	-	-	-	-	-	-	-	-	-	lip	-
4406139	0,0%	0,0%	0,0%	0,0%	0,1%	0,0%	0,0%	0,1%	0,3%	Bacteria	Proteobacteria	Betaproteobacteria			uncultured	-	-	-	-	-	-	-	-	-	-	-	-	-	-	-	-	-	-	-	-	-	-	-	lip	-
896425	0,0%	0,0%	0,0%	0,1%	0,1%	0,1%	0,0%	0,0%	0,0%	Bacteria	Actinobacteria	Actinobacteria	Actinomycetales	Actinospicaceae	Actinospica	-	napA	-	-	-	-	-	-	-	-	-	-	-	-	-	-	-	-	-	-	-	-	-	-	-
4420495	0,0%	0,0%	0,1%	0,1%	0,0%	0,0%	0,0%	0,0%	0,0%	Bacteria	Proteobacteria	Alphaproteobacteria			uncultured bacterium	-	-	-	-	-	-	-	-	-	-	-	-	-	-	-	-	-	-	-	-	-	-	-	-	-
4475690	0,0%	0,0%	0,0%	0,0%	0,0%	0,3%	0,0%	0,0%	0,0%	Bacteria	Proteobacteria	Betaproteobacteria	Burkholderiales	Oxalobacteraceae	Herbaspirillum	-	napA	narG	-	-	nirS	nirK	norB	nosZ	nifH	nifD	-	ureA	-	-	-	-	-	-	-	-	laccase_Basidio	-	-	-
1783754	0,0%	0,0%	0,0%	0,0%	0,0%	0,2%	0,0%	0,1%	0,0%	Bacteria	Proteobacteria	Gammaproteobacteria	[Marinicellales]	[Marinicellaceae]	Marinicella	-	-	-	-	-	-	-	-	-	-	-	-	-	-	-	-	-	-	-	-	-	-	-	-	-
4450317	0,0%	0,0%	0,1%	0,0%	0,0%	0,0%	0,0%	0,1%	0,0%	Bacteria	Proteobacteria	Betaproteobacteria	Burkholderiales	Comamonadaceae	uncultured	-	-	-	-	-	-	-	-	-	-	-	-	-	-	-	-	-	-	-	-	-	-	-	lip	-
4345879	0,0%	0,0%	0,0%	0,0%	0,0%	0,0%	0,1%	0,0%	0,0%	Bacteria	Acidobacteria	Solibacteres	Solibacterales	Solibacteraceae	Candidatus Solibacter	-	-	-	-	-	-	-	-	-	-	-	-	-	-	-	-	-	-	-	-	-	-	-	-	-
407742	0,0%	0,0%	0,0%	0,0%	0,0%	0,0%	0,0%	0,0%	0,0%	Bacteria	Proteobacteria	Alphaproteobacteria	Rhodospirillales	Acetobacteraceae	Rhodovastum	-	-	-	-	-	-	-	-	-	-	-	-	-	-	-	-	-	-	-	-	-	-	-	-	-
110215	0,0%	0,0%	0,1%	0,0%	0,0%	0,0%	0,0%	0,0%	0,0%	Bacteria	Proteobacteria	Betaproteobacteria	Burkholderiales	Comamonadaceae	uncultured	-	-	-	-	-	-	-	-	-	-	-	-	-	-	-	-	-	-	-	-	-	-	-	lip	-
4254478	0,0%	0,0%	0,0%	0,0%	0,0%	0,0%	0,0%	0,0%	0,0%	Bacteria	Proteobacteria	Betaproteobacteria	Burkholderiales	Comamonadaceae	uncultured	-	-	-	-	-	-	-	-	-	-	-	-	-	-	-	-	-	-	-	-	-	-	-	lip	-
4479485	0,0%	0,0%	0,1%	0,0%	0,0%	0,0%	0,0%	0,0%	0,0%	Bacteria	Proteobacteria	Alphaproteobacteria	Rhodospirillales	Rhodospirillaceae	Inquilinus	-	-	-	-	-	-	-	-	-	-	-	-	-	-	-	-	-	-	-	-	-	-	-	-	-
4479484	0,1%	0,0%	0,0%	0,0%	0,0%	0,0%	0,0%	0,0%	0,0%	Bacteria	Proteobacteria	Alphaproteobacteria	Sphingomonadales	Sphingomonadaceae	Sphingomonas	-	napA	narG	nirA	-	-	nirK	norB	-	nifH	nifD	-	ureA	-	-	-	-	chb	mmoX	-	laccase_Asco	laccase_Basidio	-	-	ppo
4479483	0,0%	0,0%	0,0%	0,0%	0,0%	0,1%	0,0%	0,0%	0,0%	Bacteria	Chloroflexi	Ktedonobacteria	Ktedonobacterales	Ktedonobacteraceae	Ktedonobacter	-	-	-	-	-	-	-	-	-	-	-	-	ureA	-	-	-	-	-	-	-	-	laccase_Basidio	-	-	-
617099	0,0%	0,0%	0,1%	0,0%	0,1%	0,1%	0,0%	0,0%	0,0%	Bacteria	Proteobacteria	Betaproteobacteria	Burkholderiales	Oxalobacteraceae	Herbaspirillum	-	napA	narG	-	-	nirS	nirK	norB	nosZ	nifH	nifD	-	ureA	-	-	-	-	-	-	-	-	laccase_Basidio	-	-	-
509038	0,0%	0,0%	0,0%	0,1%	0,0%	0,0%	0,0%	0,0%	0,0%	Bacteria	Proteobacteria	Betaproteobacteria	Burkholderiales	Alcaligenaceae	uncultured	cellulolytic	-	-	-	-	-	-	-	-	-	-	-	-	-	-	-	-	-	-	-	-	-	-	lip	-
536133	0,0%	0,1%	0,0%	0,1%	0,0%	0,0%	0,0%	0,0%	0,1%	Bacteria	Verrucomicrobia	[Methylacidiphilae]	Methylacidiphilales		Candidatus Methylacidiphilum	-	-	-	-	-	-	-	-	-	-	-	-	-	-	-	-	-	-	-	-	-	-	-	-	-
4422872	0,1%	0,0%	0,0%	0,0%	0,0%	0,0%	0,0%	0,0%	0,0%	Bacteria	Bacteroidetes	[Saprospirae]	[Saprospirales]	Chitinophagaceae	Hydrotalea	-	-	-	-	-	-	-	-	-	-	-	-	-	-	-	-	-	-	-	-	-	-	-	-	-
921911	0,0%	0,1%	0,0%	0,1%	0,0%	0,0%	0,0%	0,0%	0,1%	Bacteria	Proteobacteria	Deltaproteobacteria	MIZ46		uncultured Syntrophobacteraceae bacterium	-	-	-	-	-	-	-	-	-	-	-	-	-	-	-	-	-	-	-	-	-	-	-	-	-
583945	0,0%	0,0%	0,0%	0,1%	0,0%	0,2%	0,0%	0,0%	0,0%	Bacteria	Verrucomicrobia	[Pedosphaerae]	[Pedosphaerales]	[Pedosphaeraceae]	Pedosphaera	-	napA	-	-	-	-	nirK	-	-	-	-	-	-	-	-	-	-	-	-	-	-	-	-	-	-
4298129	0,0%	0,0%	0,0%	0,0%	0,0%	0,0%	0,0%	0,0%	0,1%	Bacteria	Proteobacteria	Alphaproteobacteria	Rhodospirillales	Rhodospirillaceae	uncultured bacterium	-	-	-	-	-	-	-	-	-	-	-	-	-	-	-	-	-	-	-	-	-	-	-	-	-
150982	0,0%	0,0%	0,1%	0,0%	0,0%	0,0%	0,0%	0,0%	0,0%	Bacteria	Proteobacteria	Gammaproteobacteria	Xanthomonadales	Sinobacteraceae	uncultured gamma proteobacterium	-	-	-	-	-	-	-	-	-	-	-	-	-	-	-	-	-	-	-	-	-	-	-	-	-
800909	0,0%	0,0%	0,1%	0,0%	0,0%	0,0%	0,0%	0,0%	0,0%	Bacteria	Planctomycetes	Planctomycetia	Gemmatales	Isosphaeraceae	uncultured bacterium	-	-	-	-	-	-	-	-	-	-	-	-	-	-	-	-	-	-	-	-	-	-	-	-	-
801258	0,0%	0,0%	0,0%	0,0%	0,1%	0,2%	0,0%	0,0%	0,1%	Bacteria	Verrucomicrobia	[Methylacidiphilae]	Methylacidiphilales		Candidatus Methylacidiphilum	-	-	-	-	-	-	-	-	-	-	-	-	-	-	-	-	-	-	-	-	-	-	-	-	-
1059762	0,0%	0,0%	0,0%	0,0%	0,1%	0,0%	0,0%	0,0%	0,0%	Bacteria	Proteobacteria	Gammaproteobacteria	Xanthomonadales	Sinobacteraceae	uncultured bacterium	-	-	-	-	-	-	-	-	-	-	-	-	-	-	-	-	-	-	-	-	-	-	-	-	-
277727	0,0%	0,1%	0,0%	0,1%	0,0%	0,0%	0,0%	0,0%	0,0%	Bacteria	Bacteroidetes	Sphingobacteriia	Sphingobacteriales		uncultured bacterium	-	-	-	-	-	-	-	-	-	-	-	-	-	-	-	-	-	-	-	-	-	-	-	-	-
828474	0,0%	0,0%	0,0%	0,0%	0,1%	0,0%	0,0%	0,0%	0,0%	Bacteria	Proteobacteria	Gammaproteobacteria	Aeromonadales	Aeromonadaceae	uncultured bacterium	-	-	-	-	-	-	-	-	-	-	-	-	-	-	-	-	-	-	-	-	-	-	-	-	-
569628	0,0%	0,0%	0,0%	0,0%	0,0%	0,4%	0,0%	0,0%	0,0%	Bacteria	Verrucomicrobia	[Pedosphaerae]	[Pedosphaerales]	auto67_4W	uncultured bacterium	-	-	-	-	-	-	-	-	-	-	-	-	-	-	-	-	-	-	-	-	-	-	-	-	-
1120966	0,0%	0,0%	0,1%	0,0%	0,0%	0,0%	0,0%	0,1%	0,0%	Bacteria	Proteobacteria	Betaproteobacteria	Burkholderiales	Comamonadaceae	Variovorax	-	napA	narG	nirA	-	-	-	norB	-	-	-	-	ureA	-	-	-	-	-	mmoX	-	-	-	-	-	-
579129	0,0%	0,0%	0,1%	0,0%	0,0%	0,0%	0,0%	0,0%	0,0%	Bacteria	Planctomycetes	Planctomycetia	Pirellulales	Pirellulaceae	uncultured	-	-	-	-	-	-	-	-	-	-	-	-	-	-	-	-	-	-	-	-	-	-	-	lip	-
753767	0,1%	0,0%	0,0%	0,0%	0,0%	0,0%	0,0%	0,0%	0,1%	Bacteria	Proteobacteria	Alphaproteobacteria	Rhodospirillales	Rhodospirillaceae	Azospirillum	-	napA	-	nirA	-	nirS	nirK	norB	nosZ	nifH	nifD	-	ureA	-	-	-	-	-	mmoX	-	laccase_Asco	laccase_Basidio	-	-	ppo
4314949	0,1%	0,0%	0,0%	0,0%	0,0%	0,0%	0,0%	0,0%	0,0%	Bacteria	Proteobacteria	Deltaproteobacteria	MIZ46		uncultured bacterium	-	-	-	-	-	-	-	-	-	-	-	-	-	-	-	-	-	-	-	-	-	-	-	-	-
340714	0,1%	0,0%	0,0%	0,0%	0,0%	0,0%	0,0%	0,0%	0,0%	Bacteria	Proteobacteria	Alphaproteobacteria	Rhizobiales	Aurantimonadaceae	uncultured	-	-	-	-	-	-	-	-	-	-	-	-	-	-	-	-	-	-	-	-	-	-	-	lip	-
2775220	0,1%	0,0%	0,0%	0,0%	0,0%	0,2%	0,0%	0,0%	0,0%	Bacteria	Bacteroidetes	Sphingobacteriia	Sphingobacteriales		uncultured bacterium	-	-	-	-	-	-	-	-	-	-	-	-	-	-	-	-	-	-	-	-	-	-	-	-	-
660211	0,0%	0,0%	0,1%	0,0%	0,0%	0,0%	0,1%	0,0%	0,0%	Bacteria	Proteobacteria	Gammaproteobacteria	Methylococcales	Crenotrichaceae	Methylovulum	-	-	-	-	-	-	-	-	-	nifH	-	-	-	-	-	-	-	-	mmoX	pmoA	-	-	-	-	-
4451152	0,0%	0,0%	0,1%	0,0%	0,0%	0,0%	0,0%	0,0%	0,3%	Bacteria	Bacteroidetes	Bacteroidia	Bacteroidales	Bacteroidaceae	Bacteroides	cellulolytic	-	narG	-	-	-	-	-	-	-	nifD	nrfA	-	-	dsrB	-	-	chb	-	-	-	-	-	-	-
831179	0,0%	0,1%	0,0%	0,0%	0,0%	0,1%	0,0%	0,0%	0,0%	Bacteria	Proteobacteria	Betaproteobacteria	Burkholderiales	Comamonadaceae	uncultured	-	-	-	-	-	-	-	-	-	-	-	-	-	-	-	-	-	-	-	-	-	-	-	lip	-
4364412	0,0%	0,1%	0,0%	0,0%	0,0%	0,1%	0,0%	0,0%	0,0%	Bacteria	Proteobacteria	Alphaproteobacteria	Rhodospirillales	Rhodospirillaceae	Reyranella	-	-	-	-	-	-	-	-	-	-	-	-	-	-	-	-	-	-	-	-	-	-	-	-	-
671445	0,0%	0,0%	0,0%	0,0%	0,1%	0,0%	0,0%	0,0%	0,4%	Bacteria	Proteobacteria	Alphaproteobacteria			uncultured bacterium	-	-	-	-	-	-	-	-	-	-	-	-	-	-	-	-	-	-	-	-	-	-	-	-	-
4388819	0,0%	0,1%	0,0%	0,0%	0,0%	0,0%	0,0%	0,1%	0,5%	Bacteria	Proteobacteria	Alphaproteobacteria	Rhizobiales	Bradyrhizobiaceae	Afipia	-	napA	narG	-	-	-	nirK	-	nosZ	nifH	-	-	ureA	-	-	-	-	-	-	-	laccase_Asco	laccase_Basidio	-	-	ppo
755581	0,0%	0,0%	0,1%	0,0%	0,0%	0,2%	0,0%	0,0%	0,0%	Bacteria	Proteobacteria	Deltaproteobacteria	MIZ46		uncultured bacterium	-	-	-	-	-	-	-	-	-	-	-	-	-	-	-	-	-	-	-	-	-	-	-	-	-
4467399	0,0%	0,0%	0,0%	0,1%	0,0%	0,0%	0,0%	0,0%	0,0%	Bacteria	Proteobacteria	Deltaproteobacteria	Desulfuromonadales	Geobacteraceae	Geobacter	-	napA	narG	-	-	-	-	norB	-	nifH	nifD	nrfA	-	-	-	-	-	-	-	-	laccase_Asco	laccase_Basidio	-	-	ppo
4464847	0,0%	0,0%	0,0%	0,0%	0,0%	0,0%	0,1%	0,0%	0,0%	Bacteria	Proteobacteria	Deltaproteobacteria	Syntrophobacterales	Syntrophobacteraceae	uncultured bacterium	-	-	-	-	-	-	-	-	-	-	-	-	-	-	-	-	-	-	-	-	-	-	-	-	-
648004	0,0%	0,0%	0,0%	0,0%	0,1%	0,1%	0,0%	0,0%	0,0%	Bacteria	Proteobacteria	Alphaproteobacteria	Rhodospirillales	Acetobacteraceae	Roseomonas	-	napA	-	-	-	-	-	-	-	-	nifD	-	ureA	-	-	-	-	-	-	-	-	laccase_Basidio	-	-	-
358386	0,0%	0,0%	0,0%	0,0%	0,0%	0,0%	0,0%	0,0%	0,0%	Bacteria	Proteobacteria	Alphaproteobacteria	Rhodospirillales	Acetobacteraceae	uncultured	-	-	-	-	-	-	-	-	-	-	-	-	-	-	-	-	-	-	-	-	-	-	-	lip	-
57713	0,0%	0,0%	0,1%	0,0%	0,0%	0,0%	0,0%	0,0%	0,0%	Bacteria	Armatimonadetes	[Fimbriimonadia]	[Fimbriimonadales]	[Fimbriimonadaceae]	uncultured bacterium	-	-	-	-	-	-	-	-	-	-	-	-	-	-	-	-	-	-	-	-	-	-	-	-	-
307622	0,0%	0,0%	0,1%	0,0%	0,0%	0,0%	0,0%	0,0%	0,0%	Bacteria	Proteobacteria	Deltaproteobacteria	Myxococcales		uncultured delta proteobacterium	-	-	-	-	-	-	-	-	-	-	-	-	-	-	-	-	-	-	-	-	-	-	-	-	-
1135155	0,0%	0,0%	0,0%	0,0%	0,0%	0,1%	0,0%	0,0%	0,0%	Bacteria	Bacteroidetes	[Saprospirae]	[Saprospirales]	Chitinophagaceae	Ferruginibacter	-	-	-	-	-	-	-	-	-	-	-	-	-	-	-	-	-	-	-	-	-	-	-	-	-
509629	0,0%	0,0%	0,0%	0,0%	0,0%	0,0%	0,1%	0,0%	0,0%	Bacteria	Verrucomicrobia	[Pedosphaerae]	[Pedosphaerales]		uncultured Verrucomicrobia bacterium	-	-	-	-	-	-	-	-	-	-	-	-	-	-	-	-	-	-	-	-	-	-	-	-	-
131389	0,0%	0,0%	0,0%	0,0%	0,0%	0,0%	0,0%	0,0%	0,0%	Bacteria	Proteobacteria	Alphaproteobacteria	Rhizobiales	Hyphomicrobiaceae	Hyphomicrobium	-	napA	narG	nirA	-	-	nirK	norB	nosZ	nifH	nifD	-	ureA	-	-	-	-	-	mmoX	-	laccase_Asco	laccase_Basidio	-	-	ppo
808919	0,0%	0,0%	0,0%	0,0%	0,0%	0,1%	0,0%	0,0%	0,0%	Bacteria	Proteobacteria	Deltaproteobacteria	Myxococcales	Polyangiaceae	Byssovorax	-	-	-	-	-	-	-	-	-	-	-	-	-	-	-	-	-	-	-	-	-	-	-	-	-
222753	0,1%	0,0%	0,0%	0,0%	0,0%	0,0%	0,0%	0,0%	0,1%	Bacteria	Proteobacteria	Gammaproteobacteria	Alteromonadales	Alteromonadaceae	Cellvibrio	-	napA	narG	-	-	-	nirK	norB	-	-	-	-	-	-	-	-	-	chb	-	-	-	laccase_Basidio	-	-	-
4418892	0,0%	0,0%	0,0%	0,0%	0,1%	0,0%	0,0%	0,0%	0,0%	Bacteria	Proteobacteria	Alphaproteobacteria	Rhodospirillales	Acetobacteraceae	Acidicaldus	-	-	-	-	-	-	-	-	-	-	-	-	-	-	-	-	-	-	-	-	-	-	-	-	-
4459144	0,0%	0,0%	0,1%	0,0%	0,0%	0,0%	0,0%	0,1%	0,0%	Bacteria	Proteobacteria	Alphaproteobacteria	Rhodospirillales	Rhodospirillaceae	uncultured bacterium	-	-	-	-	-	-	-	-	-	-	-	-	-	-	-	-	-	-	-	-	-	-	-	-	-
575014	0,0%	0,0%	0,0%	0,0%	0,1%	0,0%	0,0%	0,0%	0,0%	Bacteria	Bacteroidetes	Cytophagia	Cytophagales	Cytophagaceae	uncultured	cellulolytic	-	-	-	-	-	-	-	-	-	-	-	-	-	-	-	-	-	-	-	-	-	-	lip	-
4309997	0,0%	0,0%	0,0%	0,1%	0,0%	0,1%	0,0%	0,0%	0,3%	Bacteria	Bacteroidetes	Flavobacteriia	Flavobacteriales	Cryomorphaceae	Fluviicola	-	-	-	nirA	-	-	-	norB	-	-	-	-	-	-	-	-	-	-	-	-	laccase_Asco	laccase_Basidio	-	-	ppo
758197	0,0%	0,0%	0,1%	0,0%	0,0%	0,0%	0,0%	0,0%	0,0%	Bacteria	Proteobacteria	Deltaproteobacteria	MIZ46		uncultured bacterium	-	-	-	-	-	-	-	-	-	-	-	-	-	-	-	-	-	-	-	-	-	-	-	-	-
41200	0,0%	0,1%	0,0%	0,1%	0,0%	0,0%	0,0%	0,0%	0,0%	Bacteria	Proteobacteria	Betaproteobacteria	Ellin6067		uncultured	-	-	-	-	-	-	-	-	-	-	-	-	-	-	-	-	-	-	-	-	-	-	-	lip	-
1746395	0,0%	0,0%	0,0%	0,0%	0,0%	0,0%	0,0%	0,0%	0,0%	Bacteria	Proteobacteria	Alphaproteobacteria	Rhodobacterales	Rhodobacteraceae	Rhodobacter	-	napA	-	nirA	-	nirS	nirK	norB	nosZ	nifH	nifD	-	ureA	-	-	SoxB	-	-	mmoX	-	laccase_Asco	laccase_Basidio	-	-	ppo
2708428	0,1%	0,0%	0,0%	0,0%	0,0%	0,0%	0,0%	0,0%	0,0%	Bacteria	Acidobacteria	Solibacteres	Solibacterales	Solibacteraceae	Candidatus Solibacter	-	-	-	-	-	-	-	-	-	-	-	-	-	-	-	-	-	-	-	-	-	-	-	-	-
1129599	0,1%	0,0%	0,0%	0,0%	0,0%	0,0%	0,0%	0,0%	0,0%	Bacteria	Proteobacteria	Alphaproteobacteria	Sphingomonadales	Sphingomonadaceae	Novosphingobium	-	napA	-	-	-	-	-	norB	-	nifH	nifD	-	ureA	-	-	-	-	-	mmoX	-	laccase_Asco	laccase_Basidio	-	-	ppo
352392	0,0%	0,0%	0,1%	0,0%	0,0%	0,0%	0,0%	0,0%	0,0%	Bacteria	Proteobacteria	Gammaproteobacteria	Pseudomonadales	Moraxellaceae	Perlucidibaca	-	-	-	-	-	-	-	-	-	-	-	-	-	-	-	-	-	-	-	-	-	-	-	-	-
1100082	0,0%	0,0%	0,1%	0,0%	0,0%	0,0%	0,0%	0,0%	0,0%	Bacteria	Verrucomicrobia	[Spartobacteria]	[Chthoniobacterales]	[Chthoniobacteraceae]	Chthoniobacter	-	napA	-	-	-	-	nirK	-	-	-	-	nrfA	ureA	-	-	-	-	-	-	-	-	-	-	-	-
1128978	0,0%	0,0%	0,1%	0,0%	0,0%	0,0%	0,0%	0,0%	0,0%	Bacteria	Proteobacteria	Deltaproteobacteria	Myxococcales		Sandaracinus	-	napA	-	-	-	-	-	-	-	-	-	-	-	-	-	-	-	-	-	-	-	laccase_Basidio	-	-	ppo
4434564	0,0%	0,0%	0,0%	0,0%	0,0%	0,3%	0,0%	0,0%	0,0%	Bacteria	Proteobacteria	Betaproteobacteria	Burkholderiales	Comamonadaceae	Comamonas	-	napA	-	nirA	-	nirS	nirK	norB	nosZ	-	-	-	ureA	-	-	SoxB	-	-	mmoX	-	-	laccase_Basidio	-	-	-
208293	0,0%	0,0%	0,0%	0,0%	0,0%	0,0%	0,0%	0,0%	0,1%	Bacteria	Proteobacteria	Alphaproteobacteria	Rhizobiales	Hyphomicrobiaceae	Pseudolabrys	-	napA	narG	-	-	-	-	-	nosZ	-	-	-	ureA	-	-	-	-	-	-	-	-	-	-	-	-
137056	0,0%	0,0%	0,0%	0,0%	0,0%	0,0%	0,0%	0,0%	0,1%	Bacteria	Firmicutes	Bacilli	Bacillales	Planococcaceae	Staphylococcus	-	napA	narG	-	-	-	nirK	norB	-	-	-	-	ureA	-	-	-	-	-	-	-	laccase_Asco	laccase_Basidio	-	-	-
3990161	0,0%	0,0%	0,0%	0,0%	0,0%	0,0%	0,1%	0,0%	0,0%	Bacteria	Acidobacteria	Acidobacteriia	Acidobacteriales	Acidobacteriaceae	Granulicella	cellulolytic	napA	-	-	-	-	-	-	-	-	-	-	-	-	-	-	-	-	-	-	laccase_Asco	laccase_Basidio	-	-	ppo
99144	0,1%	0,0%	0,0%	0,0%	0,0%	0,0%	0,0%	0,0%	0,0%	Bacteria	Verrucomicrobia	[Pedosphaerae]	[Pedosphaerales]	R4-41B	uncultured Verrucomicrobia bacterium	-	-	-	-	-	-	-	-	-	-	-	-	-	-	-	-	-	-	-	-	-	-	-	-	-
567259	0,0%	0,0%	0,1%	0,0%	0,0%	0,0%	0,0%	0,0%	0,0%	Bacteria	Bacteroidetes	Cytophagia	Cytophagales	Cytophagaceae	Arcicella	cellulolytic	-	-	-	-	-	-	-	-	-	-	-	-	-	-	-	-	-	-	-	-	-	-	-	-
810198	0,0%	0,0%	0,0%	0,0%	0,0%	0,0%	0,1%	0,0%	0,0%	Bacteria	Acidobacteria	Acidobacteriia	Acidobacteriales	Acidobacteriaceae	Acidobacterium	cellulolytic	napA	-	-	-	-	-	-	-	-	-	-	-	-	-	-	-	-	-	-	-	laccase_Basidio	-	-	ppo
4347970	0,0%	0,0%	0,0%	0,0%	0,0%	0,0%	0,1%	0,1%	0,0%	Bacteria	Proteobacteria	Deltaproteobacteria	Myxococcales	Haliangiaceae	Haliangium	-	-	-	-	-	-	-	-	-	-	-	-	ureA	-	-	-	-	chb	-	-	-	-	-	-	-
4461581	0,0%	0,0%	0,1%	0,1%	0,0%	0,1%	0,0%	0,0%	0,0%	Bacteria	Planctomycetes	Planctomycetia	Gemmatales	Gemmataceae	uncultured	-	-	-	-	-	-	-	-	-	-	-	-	-	-	-	-	-	-	-	-	-	-	-	lip	-
4473756	0,0%	0,0%	0,1%	0,0%	0,0%	0,0%	0,0%	0,0%	0,0%	Bacteria	Actinobacteria	Actinobacteria	Actinomycetales	Microbacteriaceae	Pseudoclavibacter	cellulolytic	-	-	-	-	-	-	-	-	-	-	-	-	-	-	-	-	-	-	-	-	-	-	-	-
1087471	0,0%	0,0%	0,0%	0,0%	0,0%	0,0%	0,1%	0,0%	0,0%	Bacteria	Bacteroidetes	Sphingobacteriia	Sphingobacteriales		uncultured bacterium	-	-	-	-	-	-	-	-	-	-	-	-	-	-	-	-	-	-	-	-	-	-	-	-	-
4458848	0,0%	0,0%	0,0%	0,0%	0,0%	0,1%	0,1%	0,0%	0,0%	Bacteria	Proteobacteria	Deltaproteobacteria	Myxococcales	0319-6G20	uncultured bacterium	-	-	-	-	-	-	-	-	-	-	-	-	-	-	-	-	-	-	-	-	-	-	-	-	-
1124273	0,0%	0,0%	0,0%	0,0%	0,0%	0,0%	0,1%	0,0%	0,0%	Bacteria	Bacteroidetes	Flavobacteriia	Flavobacteriales	Flavobacteriaceae	Flavobacterium	cellulolytic	napA	-	nirA	-	-	nirK	norB	nosZ	-	-	-	ureA	-	-	-	-	-	-	-	laccase_Asco	laccase_Basidio	-	-	ppo
799069	0,0%	0,0%	0,0%	0,0%	0,0%	0,0%	0,0%	0,1%	0,0%	Bacteria	Actinobacteria	Actinobacteria	Actinomycetales		Acidothermus	-	napA	narG	-	-	-	nirK	-	-	-	-	-	-	-	-	-	-	-	-	-	-	-	-	-	-
217201	0,0%	0,0%	0,1%	0,0%	0,0%	0,0%	0,0%	0,0%	0,0%	Bacteria	Proteobacteria	Alphaproteobacteria	Rhodospirillales	Rhodospirillaceae	Reyranella	-	-	-	-	-	-	-	-	-	-	-	-	-	-	-	-	-	-	-	-	-	-	-	-	-
3394215	0,0%	0,0%	0,0%	0,0%	0,0%	0,1%	0,0%	0,1%	0,0%	Bacteria	Verrucomicrobia	[Spartobacteria]	[Chthoniobacterales]	[Chthoniobacteraceae]	uncultured bacterium	-	-	-	-	-	-	-	-	-	-	-	-	-	-	-	-	-	-	-	-	-	-	-	-	-
2440017	0,1%	0,0%	0,0%	0,0%	0,0%	0,0%	0,0%	0,0%	0,0%	Bacteria	Bacteroidetes	Sphingobacteriia	Sphingobacteriales		uncultured bacterium	-	-	-	-	-	-	-	-	-	-	-	-	-	-	-	-	-	-	-	-	-	-	-	-	-
246691	0,0%	0,0%	0,1%	0,0%	0,0%	0,0%	0,0%	0,0%	0,0%	Bacteria	Proteobacteria	Betaproteobacteria	Methylophilales	Methylophilaceae	uncultured	-	-	-	-	-	-	-	-	-	-	-	-	-	-	-	-	-	-	-	-	-	-	-	lip	-
85393	0,0%	0,0%	0,0%	0,0%	0,0%	0,0%	0,0%	0,0%	0,0%	Bacteria	Bacteroidetes	Sphingobacteriia	Sphingobacteriales		uncultured bacterium	-	-	-	-	-	-	-	-	-	-	-	-	-	-	-	-	-	-	-	-	-	-	-	-	-
4467002	0,0%	0,1%	0,0%	0,1%	0,0%	0,0%	0,0%	0,0%	0,0%	Bacteria	Proteobacteria	Alphaproteobacteria	Sphingomonadales	Erythrobacteraceae	Porphyrobacter	-	napA	-	-	-	-	-	-	-	-	-	-	-	-	-	-	-	-	-	-	-	-	-	-	ppo
871009	0,0%	0,0%	0,0%	0,0%	0,0%	0,0%	0,0%	0,0%	0,0%	Bacteria	Proteobacteria	Deltaproteobacteria	FAC87		uncultured bacterium	-	-	-	-	-	-	-	-	-	-	-	-	-	-	-	-	-	-	-	-	-	-	-	-	-
4334436	0,1%	0,0%	0,0%	0,0%	0,0%	0,0%	0,0%	0,0%	0,0%	Bacteria	Proteobacteria	Epsilonproteobacteria	Campylobacterales	Helicobacteraceae	Sulfuricurvum	-	napA	-	nirA	-	-	nirK	norB	-	nifH	nifD	-	-	-	-	SoxB	-	-	-	-	-	-	-	-	-
1107976	0,0%	0,0%	0,0%	0,0%	0,0%	0,1%	0,0%	0,1%	0,0%	Bacteria	Acidobacteria	Sva0725	Sva0725		uncultured bacterium	-	-	-	-	-	-	-	-	-	-	-	-	-	-	-	-	-	-	-	-	-	-	-	-	-
278140	0,0%	0,1%	0,0%	0,0%	0,1%	0,0%	0,0%	0,0%	0,0%	Bacteria	Planctomycetes	Planctomycetia	Gemmatales	Gemmataceae	Gemmata	-	-	-	-	-	-	-	-	-	-	-	-	-	-	-	-	-	-	-	-	-	-	-	-	-
331322	0,0%	0,1%	0,0%	0,1%	0,0%	0,0%	0,0%	0,0%	0,0%	Bacteria	Proteobacteria	Gammaproteobacteria	Legionellales	Legionellaceae	Legionella	-	-	-	-	-	nirS	-	norB	-	-	-	-	-	-	-	-	-	-	-	-	laccase_Asco	laccase_Basidio	-	-	ppo
119231	0,0%	0,0%	0,0%	0,0%	0,1%	0,0%	0,0%	0,0%	0,0%	Bacteria	Verrucomicrobia	[Spartobacteria]	[Chthoniobacterales]	[Chthoniobacteraceae]	uncultured Verrucomicrobia bacterium	-	-	-	-	-	-	-	-	-	-	-	-	-	-	-	-	-	-	-	-	-	-	-	-	-
4431178	0,0%	0,0%	0,0%	0,0%	0,0%	0,0%	0,0%	0,0%	0,0%	Bacteria	Proteobacteria	Alphaproteobacteria	Rhizobiales	Phyllobacteriaceae	Ensifer	-	napA	-	-	-	-	nirK	-	nosZ	nifH	-	-	ureA	-	-	-	-	-	-	-	laccase_Asco	laccase_Basidio	-	-	ppo
4035617	0,0%	0,0%	0,0%	0,0%	0,1%	0,1%	0,0%	0,0%	0,0%	Bacteria	Proteobacteria	Betaproteobacteria	Burkholderiales	Comamonadaceae	Aquincola	-	-	-	-	-	-	-	-	-	-	-	-	-	-	-	-	-	-	-	-	-	-	-	-	-
398721	0,0%	0,0%	0,0%	0,0%	0,1%	0,0%	0,0%	0,0%	0,0%	Bacteria	Proteobacteria	Gammaproteobacteria	Xanthomonadales	Sinobacteraceae	uncultured bacterium	-	-	-	-	-	-	-	-	-	-	-	-	-	-	-	-	-	-	-	-	-	-	-	-	-
937813	0,0%	0,1%	0,0%	0,0%	0,0%	0,0%	0,0%	0,1%	0,0%	Bacteria	Firmicutes	Clostridia	Clostridiales	[Tissierellaceae]	Anaerococcus	-	napA	-	-	-	-	-	-	-	-	-	nrfA	ureA	-	-	-	-	-	-	-	-	-	-	-	-
5266	0,0%	0,1%	0,0%	0,0%	0,0%	0,0%	0,0%	0,1%	0,0%	Bacteria	Proteobacteria	Alphaproteobacteria	Rhizobiales	Rhizobiaceae	Rhizobium	-	napA	narG	nirA	-	-	nirK	norB	nosZ	nifH	nifD	nrfA	ureA	-	-	-	-	chb	mmoX	-	laccase_Asco	laccase_Basidio	-	-	ppo
4311663	0,0%	0,0%	0,0%	0,0%	0,0%	0,3%	0,0%	0,0%	0,0%	Bacteria	Proteobacteria	Betaproteobacteria	Methylophilales	Methylophilaceae	uncultured	-	-	-	-	-	-	-	-	-	-	-	-	-	-	-	-	-	-	-	-	-	-	-	lip	-
818395	0,0%	0,0%	0,0%	0,0%	0,0%	0,0%	0,1%	0,0%	0,0%	Bacteria	OP3	PBS-25			uncultured bacterium	-	-	-	-	-	-	-	-	-	-	-	-	-	-	-	-	-	-	-	-	-	-	-	-	-
156353	0,0%	0,0%	0,0%	0,0%	0,0%	0,0%	0,1%	0,0%	0,0%	Bacteria	Verrucomicrobia	[Pedosphaerae]	[Pedosphaerales]		uncultured bacterium	-	-	-	-	-	-	-	-	-	-	-	-	-	-	-	-	-	-	-	-	-	-	-	-	-
760704	0,1%	0,0%	0,0%	0,0%	0,0%	0,0%	0,0%	0,0%	0,0%	Bacteria	Proteobacteria	Gammaproteobacteria	Legionellales	Legionellaceae	Legionella	-	-	-	-	-	nirS	-	norB	-	-	-	-	-	-	-	-	-	-	-	-	laccase_Asco	laccase_Basidio	-	-	ppo
588961	0,0%	0,0%	0,0%	0,0%	0,0%	0,0%	0,0%	0,1%	0,0%	Bacteria	Verrucomicrobia	[Spartobacteria]	[Chthoniobacterales]	[Chthoniobacteraceae]	Chthoniobacter	-	napA	-	-	-	-	nirK	-	-	-	-	nrfA	ureA	-	-	-	-	-	-	-	-	-	-	-	-
4469172	0,0%	0,0%	0,0%	0,0%	0,0%	0,0%	0,1%	0,0%	0,0%	Bacteria	Proteobacteria	Alphaproteobacteria	Rickettsiales	Rickettsiaceae	Rickettsia	-	-	-	-	-	-	-	-	-	-	-	-	-	-	-	-	-	-	-	-	-	-	-	-	-
4366310	0,0%	0,0%	0,0%	0,1%	0,0%	0,0%	0,0%	0,0%	0,5%	Bacteria	Proteobacteria	Deltaproteobacteria	Myxococcales		Sorangium	-	napA	-	-	-	-	nirK	norB	-	-	-	nrfA	ureA	-	-	-	-	chb	-	-	laccase_Asco	laccase_Basidio	-	-	ppo
587788	0,0%	0,0%	0,1%	0,0%	0,0%	0,1%	0,0%	0,0%	0,0%	Bacteria	Armatimonadetes	[Fimbriimonadia]	[Fimbriimonadales]	[Fimbriimonadaceae]	uncultured Armatimonadetes bacterium	-	-	-	-	-	-	-	-	-	-	-	-	-	-	-	-	-	-	-	-	-	-	-	-	-
814055	0,0%	0,0%	0,1%	0,0%	0,0%	0,0%	0,0%	0,0%	0,0%	Bacteria	Proteobacteria	Alphaproteobacteria	Rhizobiales		Devosia	-	napA	-	-	-	-	nirK	-	-	nifH	-	-	ureA	-	-	-	-	chb	-	-	laccase_Asco	laccase_Basidio	-	-	ppo
40205	0,0%	0,0%	0,1%	0,1%	0,0%	0,0%	0,0%	0,0%	0,0%	Bacteria	Proteobacteria	Betaproteobacteria	Burkholderiales	Comamonadaceae	Aquincola	-	-	-	-	-	-	-	-	-	-	-	-	-	-	-	-	-	-	-	-	-	-	-	-	-
970359	0,0%	0,0%	0,0%	0,0%	0,0%	0,0%	0,0%	0,0%	0,0%	Bacteria	Proteobacteria	Alphaproteobacteria	Rhizobiales	Hyphomicrobiaceae	Rhodomicrobium	-	-	-	nirA	-	-	-	-	-	nifH	nifD	-	-	dsrA	dsrB	-	-	-	-	-	-	-	-	-	-
30991	0,0%	0,0%	0,0%	0,0%	0,0%	0,3%	0,0%	0,0%	0,0%	Bacteria	Proteobacteria	Deltaproteobacteria	Myxococcales	Haliangiaceae	Haliangium	-	-	-	-	-	-	-	-	-	-	-	-	ureA	-	-	-	-	chb	-	-	-	-	-	-	-
4434709	0,0%	0,0%	0,0%	0,0%	0,0%	0,0%	0,0%	0,0%	0,0%	Bacteria	Planctomycetes	Planctomycetia	Planctomycetales	Planctomycetaceae	Planctomyces	-	napA	-	-	-	-	-	norB	-	-	-	nrfA	-	-	-	-	-	-	-	-	-	-	-	-	-
4421203	0,1%	0,0%	0,0%	0,0%	0,0%	0,0%	0,0%	0,0%	0,0%	Bacteria	Firmicutes	Clostridia	Clostridiales	Ruminococcaceae	Incertae Sedis	cellulolytic	-	-	-	-	-	-	-	-	-	-	-	-	-	-	-	-	-	-	-	-	-	-	-	-
4434837	0,1%	0,0%	0,0%	0,0%	0,0%	0,0%	0,0%	0,0%	0,0%	Bacteria	Verrucomicrobia	Verrucomicrobiae	Verrucomicrobiales	Verrucomicrobiaceae	Luteolibacter	-	-	-	-	-	-	-	-	-	-	-	-	-	-	-	-	-	-	-	-	-	-	-	-	-

547327	0,0%	0,0%	0,0%	0,0%	0,0%	0,1%	0,0%	0,0%	0,0%	Bacteria	Proteobacteria	Betaproteobacteria	Burkholderiales	Comamonadaceae	uncultured	-	-	-	-	-	-	-	-	-	-	-	-	-	-	-	-	-	-	-	-	-	-	-	lip	-
719008	0,0%	0,0%	0,0%	0,0%	0,1%	0,0%	0,0%	0,0%	0,0%	Bacteria	Bacteroidetes	Sphingobacteriia	Sphingobacteriales	Sphingobacteriaceae	Mucilaginibacter	-	-	-	-	-	-	-	-	-	-	-	-	-	-	-	-	-	-	-	-	-	-	-	-	-
4443429	0,0%	0,0%	0,0%	0,0%	0,0%	0,0%	0,0%	0,1%	0,8%	Bacteria	Proteobacteria	Gammaproteobacteria	Pseudomonadales	Moraxellaceae	Perlucidibaca	-	-	-	-	-	-	-	-	-	-	-	-	-	-	-	-	-	-	-	-	-	-	-	-	-
611144	0,0%	0,0%	0,0%	0,0%	0,0%	0,3%	0,0%	0,0%	0,0%	Bacteria	Bacteroidetes	Sphingobacteriia	Sphingobacteriales		uncultured bacterium	-	-	-	-	-	-	-	-	-	-	-	-	-	-	-	-	-	-	-	-	-	-	-	-	-
814183	0,0%	0,0%	0,0%	0,0%	0,0%	0,0%	0,0%	0,0%	0,0%	Bacteria	Proteobacteria	Betaproteobacteria	Ellin6067		uncultured	-	-	-	-	-	-	-	-	-	-	-	-	-	-	-	-	-	-	-	-	-	-	-	lip	-
4354769	0,0%	0,0%	0,1%	0,0%	0,0%	0,0%	0,0%	0,0%	0,0%	Bacteria	Bacteroidetes	Flavobacteriia	Flavobacteriales	Flavobacteriaceae	Flavobacterium	cellulolytic	napA	-	nirA	-	-	nirK	norB	nosZ	-	-	-	ureA	-	-	-	-	-	-	-	laccase_Asco	laccase_Basidio	-	-	ppo
4429771	0,0%	0,0%	0,0%	0,1%	0,0%	0,1%	0,0%	0,0%	0,0%	Bacteria	Proteobacteria	Betaproteobacteria	Burkholderiales	Comamonadaceae	Variovorax	-	napA	narG	nirA	-	-	-	norB	-	-	-	-	ureA	-	-	-	-	-	mmoX	-	-	-	-	-	-
4435098	0,0%	0,0%	0,0%	0,0%	0,0%	0,3%	0,0%	0,0%	0,0%	Bacteria	Proteobacteria	Alphaproteobacteria	Rhizobiales	Hyphomicrobiaceae	Pedomicrobium	-	-	-	-	-	-	nirK	-	-	-	-	-	-	-	-	-	-	-	-	-	-	-	-	-	-
207125	0,0%	0,0%	0,0%	0,0%	0,0%	0,1%	0,1%	0,0%	0,1%	Bacteria	Verrucomicrobia	[Spartobacteria]	[Chthoniobacterales]	[Chthoniobacteraceae]	Chthoniobacter	-	napA	-	-	-	-	nirK	-	-	-	-	nrfA	ureA	-	-	-	-	-	-	-	-	-	-	-	-
325906	0,0%	0,0%	0,0%	0,0%	0,0%	0,0%	0,0%	0,0%	0,1%	Bacteria	Proteobacteria	Betaproteobacteria			OM43 clade	-	-	-	-	-	-	-	-	-	-	-	-	-	-	-	-	-	-	-	-	-	-	-	-	-
4402133	0,1%	0,0%	0,0%	0,0%	0,0%	0,0%	0,0%	0,0%	0,0%	Bacteria	TM6	SJA-4			uncultured bacterium	-	-	-	-	-	-	-	-	-	-	-	-	-	-	-	-	-	-	-	-	-	-	-	-	-
4340165	0,0%	0,0%	0,1%	0,0%	0,0%	0,0%	0,0%	0,0%	0,0%	Bacteria	Proteobacteria	Betaproteobacteria	Burkholderiales	Comamonadaceae	uncultured	-	-	-	-	-	-	-	-	-	-	-	-	-	-	-	-	-	-	-	-	-	-	-	lip	-
645742	0,0%	0,0%	0,0%	0,0%	0,1%	0,0%	0,0%	0,0%	0,0%	Bacteria	Proteobacteria	Alphaproteobacteria	Rhizobiales	Rhizobiaceae	Rhizobium	-	napA	narG	nirA	-	-	nirK	norB	nosZ	nifH	nifD	nrfA	ureA	-	-	-	-	chb	mmoX	-	laccase_Asco	laccase_Basidio	-	-	ppo
4466603	0,0%	0,0%	0,0%	0,0%	0,0%	0,0%	0,1%	0,0%	0,0%	Bacteria	Proteobacteria	Deltaproteobacteria	Syntrophobacterales	Syntrophobacteraceae	uncultured proteobacterium	-	-	-	-	-	-	-	-	-	-	-	-	-	-	-	-	-	-	-	-	-	-	-	-	-
654531	0,0%	0,0%	0,0%	0,1%	0,0%	0,1%	0,0%	0,0%	0,0%	Bacteria	Acidobacteria	Acidobacteriia	Acidobacteriales	Koribacteraceae	uncultured	-	-	-	-	-	-	-	-	-	-	-	-	-	-	-	-	-	-	-	-	-	-	-	lip	-
4380522	0,0%	0,0%	0,0%	0,0%	0,0%	0,0%	0,1%	0,0%	0,0%	Bacteria	Proteobacteria	Alphaproteobacteria	Rhizobiales	Hyphomicrobiaceae	Rhodobium	-	-	-	-	-	-	-	-	-	-	-	-	-	-	-	-	-	-	-	-	-	-	-	-	-
4301762	0,1%	0,0%	0,0%	0,0%	0,0%	0,0%	0,0%	0,0%	0,0%	Bacteria	Proteobacteria	Gammaproteobacteria	Thiotrichales	Piscirickettsiaceae	uncultured gamma proteobacterium	-	-	-	-	-	-	-	-	-	-	-	-	-	-	-	-	-	-	-	-	-	-	-	-	-
287984	0,0%	0,0%	0,0%	0,0%	0,0%	0,0%	0,0%	0,0%	0,0%	Bacteria	Proteobacteria	Betaproteobacteria	SBla14		Azospira	-	-	narG	-	-	-	nirK	-	-	nifH	-	-	-	-	-	-	-	-	-	-	-	-	-	-	-
214306	0,0%	0,0%	0,0%	0,0%	0,0%	0,0%	0,1%	0,0%	0,0%	Bacteria	Verrucomicrobia	[Spartobacteria]	[Chthoniobacterales]	[Chthoniobacteraceae]	Chthoniobacter	-	napA	-	-	-	-	nirK	-	-	-	-	nrfA	ureA	-	-	-	-	-	-	-	-	-	-	-	-
843663	0,0%	0,0%	0,0%	0,1%	0,1%	0,0%	0,0%	0,0%	0,0%	Bacteria	Proteobacteria	Alphaproteobacteria	Rhizobiales	Methylocystaceae	uncultured Bradyrhizobium sp.	-	-	-	-	-	-	-	-	-	-	-	-	-	-	-	-	-	-	-	-	-	-	-	-	-
1068363	0,0%	0,0%	0,0%	0,0%	0,0%	0,0%	0,0%	0,0%	0,1%	Bacteria	Armatimonadetes	[Fimbriimonadia]	[Fimbriimonadales]	[Fimbriimonadaceae]	wastewater metagenome	-	-	-	-	-	-	-	-	-	-	-	-	-	-	-	-	-	-	-	-	-	-	-	-	-
532928	0,0%	0,0%	0,1%	0,0%	0,0%	0,0%	0,0%	0,0%	0,0%	Bacteria	Proteobacteria	Betaproteobacteria	Methylophilales	Methylophilaceae	Methylotenera	-	napA	-	nirA	-	-	nirK	norB	-	-	-	-	ureA	-	-	-	-	-	-	-	-	-	-	-	-
589946	0,0%	0,0%	0,0%	0,0%	0,0%	0,0%	0,0%	0,0%	0,0%	Bacteria	Proteobacteria	Alphaproteobacteria	Rhodobacterales	Hyphomonadaceae	Hirschia	-	napA	-	-	-	-	-	-	-	-	-	-	ureA	-	-	-	-	-	-	-	laccase_Asco	laccase_Basidio	-	-	ppo
825452	0,0%	0,0%	0,1%	0,0%	0,0%	0,0%	0,0%	0,0%	0,0%	Bacteria	Proteobacteria	Alphaproteobacteria	Rhodospirillales		Candidatus Alysiosphaera	-	-	-	-	-	-	-	-	-	-	-	-	-	-	-	-	-	-	-	-	-	-	-	-	-
3198474	0,0%	0,0%	0,0%	0,0%	0,0%	0,1%	0,0%	0,0%	0,0%	Bacteria	Proteobacteria	Gammaproteobacteria	Methylococcales	Methylococcaceae	Methylomonas	-	napA	-	nirA	-	nirS	nirK	norB	-	nifH	nifD	-	ureA	-	-	-	amoA_AOB	-	mmoX	pmoA	laccase_Asco	laccase_Basidio	-	-	ppo
1123510	0,0%	0,0%	0,0%	0,1%	0,1%	0,0%	0,0%	0,0%	0,0%	Bacteria	TM6	SJA-4			uncultured candidate division TM6 bacterium	-	-	-	-	-	-	-	-	-	-	-	-	-	-	-	-	-	-	-	-	-	-	-	-	-
50466	0,1%	0,0%	0,0%	0,0%	0,0%	0,0%	0,0%	0,0%	0,0%	Bacteria	Proteobacteria	Alphaproteobacteria			uncultured gamma proteobacterium	-	-	-	-	-	-	-	-	-	-	-	-	-	-	-	-	-	-	-	-	-	-	-	-	-
4477999	0,0%	0,0%	0,0%	0,1%	0,0%	0,0%	0,0%	0,0%	0,3%	Bacteria	Firmicutes	Clostridia	Clostridiales	Clostridiaceae	Clostridium sensu stricto 13	cellulolytic	-	-	-	-	-	-	-	-	-	-	-	-	-	-	-	-	-	-	-	-	-	-	-	-
4330939	0,0%	0,1%	0,0%	0,0%	0,0%	0,0%	0,0%	0,0%	0,0%	Bacteria	Proteobacteria	Gammaproteobacteria	Xanthomonadales	Xanthomonadaceae	uncultured	cellulolytic	-	-	-	-	-	-	-	-	-	-	-	-	-	-	-	-	-	-	-	-	-	-	lip	-
151176	0,0%	0,0%	0,0%	0,0%	0,0%	0,0%	0,0%	0,0%	0,0%	Bacteria	Proteobacteria	Alphaproteobacteria	Rhizobiales	Methylobacteriaceae	Methylobacterium	-	napA	narG	-	-	-	nirK	-	nosZ	nifH	nifD	-	ureA	-	-	-	-	-	-	-	laccase_Asco	laccase_Basidio	-	-	ppo
662682	0,0%	0,0%	0,1%	0,0%	0,0%	0,0%	0,0%	0,0%	0,0%	Bacteria	Planctomycetes	Planctomycetia	Planctomycetales	Planctomycetaceae	Planctomyces	-	napA	-	-	-	-	-	norB	-	-	-	nrfA	-	-	-	-	-	-	-	-	-	-	-	-	-
4309682	0,0%	0,0%	0,0%	0,0%	0,0%	0,0%	0,0%	0,0%	0,1%	Bacteria	WPS-2				uncultured bacterium	-	-	-	-	-	-	-	-	-	-	-	-	-	-	-	-	-	-	-	-	-	-	-	-	-
2788507	0,0%	0,0%	0,0%	0,0%	0,0%	0,0%	0,0%	0,0%	0,0%	Bacteria	Planctomycetes	Planctomycetia	Planctomycetales	Planctomycetaceae	Planctomyces	-	napA	-	-	-	-	-	norB	-	-	-	nrfA	-	-	-	-	-	-	-	-	-	-	-	-	-
862250	0,0%	0,0%	0,0%	0,1%	0,0%	0,0%	0,0%	0,0%	0,1%	Bacteria	Planctomycetes	Phycisphaerae	WD2101		uncultured bacterium	-	-	-	-	-	-	-	-	-	-	-	-	-	-	-	-	-	-	-	-	-	-	-	-	-
111797	0,0%	0,1%	0,0%	0,0%	0,1%	0,0%	0,0%	0,0%	0,0%	Bacteria	Proteobacteria	Alphaproteobacteria	Rhizobiales	Rhizobiaceae	uncultured	-	-	-	-	-	-	-	-	-	-	-	-	-	-	-	-	-	-	-	-	-	-	-	lip	-
4401954	0,0%	0,0%	0,0%	0,0%	0,0%	0,0%	0,1%	0,0%	0,1%	Bacteria	Proteobacteria	Deltaproteobacteria	Myxococcales		Sorangium	-	napA	-	-	-	-	nirK	norB	-	-	-	nrfA	ureA	-	-	-	-	chb	-	-	laccase_Asco	laccase_Basidio	-	-	ppo
1517344	0,0%	0,0%	0,0%	0,0%	0,0%	0,0%	0,0%	0,0%	0,0%	Bacteria	Proteobacteria	Betaproteobacteria	Burkholderiales	Comamonadaceae	Acidovorax	-	napA	narG	nirA	-	nirS	nirK	norB	nosZ	-	-	-	ureA	-	-	-	-	-	mmoX	-	-	laccase_Basidio	-	-	ppo
4453060	0,0%	0,0%	0,0%	0,0%	0,0%	0,0%	0,0%	0,2%	0,0%	Bacteria	Firmicutes	Bacilli	Lactobacillales	Enterococcaceae	Enterococcus	-	napA	narG	-	-	-	nirK	-	-	-	-	-	ureA	-	-	-	-	-	-	-	laccase_Asco	laccase_Basidio	-	-	ppo
55494	0,0%	0,0%	0,0%	0,0%	0,0%	0,3%	0,0%	0,0%	0,0%	Bacteria	Bacteroidetes	Bacteroidia	Bacteroidales	Porphyromonadaceae	Paludibacter	-	-	-	-	-	-	-	-	-	nifH	nifD	-	-	-	-	-	-	-	-	-	-	-	-	-	-
1139325	0,0%	0,0%	0,1%	0,0%	0,0%	0,0%	0,0%	0,0%	0,0%	Bacteria	Proteobacteria	Alphaproteobacteria	BD7-3		uncultured alpha proteobacterium	-	-	-	-	-	-	-	-	-	-	-	-	-	-	-	-	-	-	-	-	-	-	-	-	-
641481	0,0%	0,0%	0,0%	0,0%	0,0%	0,0%	0,0%	0,2%	0,0%	Bacteria	Proteobacteria	Deltaproteobacteria	Myxococcales		Haliangium	-	-	-	-	-	-	-	-	-	-	-	-	ureA	-	-	-	-	chb	-	-	-	-	-	-	-
825639	0,0%	0,0%	0,0%	0,0%	0,0%	0,0%	0,0%	0,0%	0,0%	Bacteria	Acidobacteria	Solibacteres	Solibacterales		Bryobacter	-	-	-	-	-	-	-	-	-	-	-	-	-	-	-	-	-	-	-	-	-	-	-	-	-
882028	0,0%	0,0%	0,0%	0,0%	0,0%	0,0%	0,1%	0,0%	0,0%	Bacteria	Proteobacteria	Alphaproteobacteria	Rhizobiales	Methylocystaceae	uncultured bacterium	-	-	-	-	-	-	-	-	-	-	-	-	-	-	-	-	-	-	-	-	-	-	-	-	-
1023267	0,0%	0,0%	0,0%	0,0%	0,0%	0,0%	0,0%	0,0%	0,0%	Bacteria	Proteobacteria	Deltaproteobacteria	Myxococcales	Nannocystaceae	Nannocystis	-	-	-	-	-	-	-	-	-	-	-	-	-	-	-	-	-	-	-	-	-	-	-	-	-
812595	0,0%	0,0%	0,0%	0,0%	0,0%	0,0%	0,1%	0,0%	0,0%	Bacteria	Proteobacteria	Deltaproteobacteria	Myxococcales		Phaselicystis	-	-	-	-	-	-	-	-	-	-	-	-	-	-	-	-	-	-	-	-	-	-	-	-	-
672587	0,0%	0,0%	0,0%	0,1%	0,0%	0,1%	0,0%	0,0%	0,0%	Bacteria	Proteobacteria	Alphaproteobacteria	Rhizobiales		Pleomorphomonas	-	-	-	-	-	-	-	-	-	nifH	-	-	-	-	-	-	-	-	-	-	-	-	-	-	-
729349	0,0%	0,0%	0,1%	0,0%	0,0%	0,0%	0,0%	0,0%	0,0%	Bacteria	Proteobacteria	Alphaproteobacteria	Ellin329		Rhizomicrobium	-	-	-	-	-	-	-	-	-	-	-	-	-	-	-	-	-	-	-	-	-	-	-	-	-
1701846	0,1%	0,0%	0,0%	0,0%	0,0%	0,0%	0,0%	0,0%	0,0%	Bacteria	Spirochaetes	Spirochaetes	Spirochaetales	Spirochaetaceae	Spirochaeta	-	-	-	nirA	-	-	-	-	-	nifH	nifD	-	-	-	-	-	-	-	-	-	-	-	-	-	-
3700608	0,0%	0,0%	0,0%	0,0%	0,0%	0,0%	0,0%	0,0%	0,0%	Bacteria	Proteobacteria	Betaproteobacteria	Burkholderiales		uncultured Oxalobacteraceae bacterium	-	-	-	-	-	-	-	-	-	-	-	-	-	-	-	-	-	-	-	-	-	-	-	-	-
4420559	0,0%	0,0%	0,0%	0,0%	0,0%	0,0%	0,1%	0,0%	0,0%	Bacteria	Actinobacteria	Thermoleophilia	Gaiellales	Gaiellaceae	Gaiella	-	-	-	-	-	-	-	-	-	-	-	-	-	-	-	-	-	-	-	-	-	-	-	-	-
4464602	0,0%	0,0%	0,1%	0,0%	0,0%	0,0%	0,0%	0,0%	0,0%	Bacteria	Actinobacteria	Actinobacteria	Actinomycetales	Nocardioidaceae	Aeromicrobium	-	napA	-	-	-	-	-	-	-	nifH	-	-	-	-	-	-	-	-	-	-	-	-	-	-	-
656342	0,0%	0,1%	0,0%	0,0%	0,0%	0,0%	0,0%	0,0%	0,0%	Bacteria	Firmicutes	Bacilli	Lactobacillales	Aerococcaceae	Aerococcus	-	-	-	-	-	-	-	-	-	-	-	-	-	-	-	-	-	-	-	-	-	-	-	-	-
27005	0,0%	0,0%	0,0%	0,0%	0,0%	0,0%	0,0%	0,0%	0,0%	Bacteria	Bacteroidetes	[Saprospirae]	[Saprospirales]	Chitinophagaceae	uncultured	-	-	-	-	-	-	-	-	-	-	-	-	-	-	-	-	-	-	-	-	-	-	-	lip	-
103580	0,0%	0,0%	0,0%	0,0%	0,0%	0,0%	0,0%	0,0%	0,0%	Bacteria	Acidobacteria	Solibacteres	Solibacterales		Bryobacter	-	-	-	-	-	-	-	-	-	-	-	-	-	-	-	-	-	-	-	-	-	-	-	-	-
4353238	0,0%	0,0%	0,0%	0,0%	0,0%	0,2%	0,0%	0,0%	0,0%	Bacteria	Planctomycetes	Planctomycetia	Pirellulales	Pirellulaceae	uncultured	-	-	-	-	-	-	-	-	-	-	-	-	-	-	-	-	-	-	-	-	-	-	-	lip	-
7559	0,0%	0,0%	0,0%	0,0%	0,0%	0,0%	0,0%	0,0%	0,0%	Bacteria	Proteobacteria	Gammaproteobacteria	Chromatiales	Chromatiaceae	Thiodictyon	-	-	-	-	-	-	-	-	-	-	-	-	-	-	-	-	-	-	-	-	-	-	-	-	-
4465584	0,0%	0,0%	0,0%	0,0%	0,0%	0,0%	0,1%	0,0%	0,0%	Bacteria	Proteobacteria	Deltaproteobacteria	Myxococcales		uncultured delta proteobacterium	-	-	-	-	-	-	-	-	-	-	-	-	-	-	-	-	-	-	-	-	-	-	-	-	-
833431	0,0%	0,0%	0,1%	0,0%	0,0%	0,0%	0,0%	0,0%	0,0%	Bacteria	Firmicutes	Clostridia	Clostridiales	Clostridiaceae	Clostridium sensu stricto 9	cellulolytic	-	-	-	-	-	-	-	-	-	-	-	-	-	-	-	-	-	-	-	-	-	-	-	-
4334834	0,1%	0,0%	0,0%	0,0%	0,0%	0,0%	0,0%	0,1%	0,0%	Bacteria	Actinobacteria	Acidimicrobiia	Acidimicrobiales		uncultured bacterium	-	-	-	-	-	-	-	-	-	-	-	-	-	-	-	-	-	-	-	-	-	-	-	-	-
519588	0,0%	0,0%	0,0%	0,0%	0,0%	0,0%	0,1%	0,0%	0,0%	Bacteria	Planctomycetes	Planctomycetia	Gemmatales	Gemmataceae	uncultured	-	-	-	-	-	-	-	-	-	-	-	-	-	-	-	-	-	-	-	-	-	-	-	lip	-
131883	0,0%	0,0%	0,1%	0,0%	0,0%	0,0%	0,0%	0,0%	0,0%	Bacteria	[Thermi]	Deinococci	Deinococcales	Deinococcaceae	Deinococcus	-	napA	-	-	-	-	nirK	-	nosZ	-	-	-	ureA	-	-	-	-	-	-	-	laccase_Asco	laccase_Basidio	-	-	ppo
4426208	0,0%	0,0%	0,0%	0,0%	0,0%	0,0%	0,0%	0,0%	0,0%	Bacteria	FCPU426				uncultured bacterium	-	-	-	-	-	-	-	-	-	-	-	-	-	-	-	-	-	-	-	-	-	-	-	-	-
241491	0,0%	0,0%	0,0%	0,0%	0,0%	0,0%	0,1%	0,0%	0,0%	Bacteria	Proteobacteria	Alphaproteobacteria	Rhodospirillales	Rhodospirillaceae	Azospirillum	-	napA	-	nirA	-	nirS	nirK	norB	nosZ	nifH	nifD	-	ureA	-	-	-	-	-	mmoX	-	laccase_Asco	laccase_Basidio	-	-	ppo
4383166	0,0%	0,0%	0,0%	0,0%	0,0%	0,1%	0,0%	0,0%	0,0%	Bacteria	Proteobacteria	Betaproteobacteria	Burkholderiales	Comamonadaceae	uncultured	-	-	-	-	-	-	-	-	-	-	-	-	-	-	-	-	-	-	-	-	-	-	-	lip	-
11359	0,0%	0,1%	0,0%	0,0%	0,0%	0,0%	0,0%	0,0%	0,0%	Bacteria	Actinobacteria	Thermoleophilia	Solirubrobacterales		uncultured Actinomycetales bacterium	-	-	-	-	-	-	-	-	-	-	-	-	-	-	-	-	-	-	-	-	-	-	-	-	-
242645	0,0%	0,0%	0,0%	0,0%	0,0%	0,0%	0,0%	0,0%	0,0%	Bacteria	Bacteroidetes	[Saprospirae]	[Saprospirales]	Chitinophagaceae	Lacibacter	-	-	-	-	-	-	-	-	-	-	-	-	-	-	-	-	-	-	-	-	-	-	-	-	-
792045	0,0%	0,0%	0,0%	0,0%	0,0%	0,2%	0,0%	0,0%	0,0%	Bacteria	Proteobacteria	Gammaproteobacteria	Legionellales	Coxiellaceae	Aquicella	-	-	-	-	-	-	-	-	-	-	-	-	-	-	-	-	-	-	-	-	-	-	-	-	-
4343704	0,0%	0,0%	0,0%	0,0%	0,0%	0,0%	0,0%	0,0%	0,0%	Bacteria	Bacteroidetes	Cytophagia	Cytophagales	Cytophagaceae	uncultured	cellulolytic	-	-	-	-	-	-	-	-	-	-	-	-	-	-	-	-	-	-	-	-	-	-	lip	-
32581	0,1%	0,0%	0,0%	0,0%	0,0%	0,0%	0,0%	0,0%	0,0%	Bacteria	Bacteroidetes	[Saprospirae]	[Saprospirales]	Chitinophagaceae	Flavisolibacter	-	-	-	-	-	-	-	-	-	-	-	-	-	-	-	-	-	-	-	-	-	-	-	-	-
4402862	0,0%	0,0%	0,0%	0,0%	0,0%	0,0%	0,0%	0,0%	0,1%	Bacteria	Planctomycetes	Planctomycetia	Gemmatales	Gemmataceae	Gemmata	-	-	-	-	-	-	-	-	-	-	-	-	-	-	-	-	-	-	-	-	-	-	-	-	-
4302627	0,0%	0,0%	0,1%	0,0%	0,0%	0,1%	0,0%	0,0%	0,0%	Bacteria	Bacteroidetes	Sphingobacteriia	Sphingobacteriales	Sphingobacteriaceae	Mucilaginibacter	-	-	-	-	-	-	-	-	-	-	-	-	-	-	-	-	-	-	-	-	-	-	-	-	-
1126251	0,0%	0,0%	0,0%	0,0%	0,0%	0,0%	0,1%	0,0%	0,0%	Bacteria	Actinobacteria	Acidimicrobiia	Acidimicrobiales		uncultured bacterium	-	-	-	-	-	-	-	-	-	-	-	-	-	-	-	-	-	-	-	-	-	-	-	-	-
589233	0,0%	0,0%	0,0%	0,0%	0,0%	0,0%	0,0%	0,0%	0,1%	Bacteria	Proteobacteria	Deltaproteobacteria	Myxococcales		Sorangium	-	napA	-	-	-	-	nirK	norB	-	-	-	nrfA	ureA	-	-	-	-	chb	-	-	laccase_Asco	laccase_Basidio	-	-	ppo
2869502	0,0%	0,0%	0,0%	0,0%	0,1%	0,0%	0,0%	0,0%	0,0%	Bacteria	Proteobacteria	Deltaproteobacteria			uncultured bacterium	-	-	-	-	-	-	-	-	-	-	-	-	-	-	-	-	-	-	-	-	-	-	-	-	-
4422405	0,0%	0,0%	0,0%	0,0%	0,0%	0,0%	0,0%	0,0%	0,0%	Bacteria	Actinobacteria	Actinobacteria	Actinomycetales	Micrococcaceae	Micrococcus	-	-	-	-	-	-	-	-	-	nifH	-	-	ureA	-	-	-	-	-	-	-	laccase_Asco	laccase_Basidio	-	-	ppo
748462	0,0%	0,0%	0,0%	0,0%	0,0%	0,2%	0,0%	0,0%	0,0%	Bacteria	Bacteroidetes	[Saprospirae]	[Saprospirales]	Chitinophagaceae	Hydrotalea	-	-	-	-	-	-	-	-	-	-	-	-	-	-	-	-	-	-	-	-	-	-	-	-	-
4353654	0,0%	0,0%	0,0%	0,0%	0,0%	0,1%	0,0%	0,0%	0,0%	Bacteria	Proteobacteria	Alphaproteobacteria	Rhizobiales	Rhizobiaceae	Rhizobium	-	napA	narG	nirA	-	-	nirK	norB	nosZ	nifH	nifD	nrfA	ureA	-	-	-	-	chb	mmoX	-	laccase_Asco	laccase_Basidio	-	-	ppo
159038	0,0%	0,0%	0,0%	0,0%	0,0%	0,0%	0,1%	0,0%	0,0%	Bacteria	Proteobacteria	Deltaproteobacteria	Bdellovibrionales	Bacteriovoracaceae	Bacteriovorax	-	-	-	-	-	-	-	norB	-	-	-	-	-	-	-	-	-	-	-	-	-	-	-	-	-
4393740	0,0%	0,0%	0,0%	0,1%	0,0%	0,0%	0,0%	0,0%	0,0%	Bacteria	Proteobacteria	Gammaproteobacteria	[Marinicellales]	[Marinicellaceae]	Marinicella	-	-	-	-	-	-	-	-	-	-	-	-	-	-	-	-	-	-	-	-	-	-	-	-	-
155216	0,0%	0,0%	0,0%	0,0%	0,0%	0,0%	0,0%	0,0%	0,0%	Bacteria	Acidobacteria	DA052	Ellin6513		uncultured bacterium	-	-	-	-	-	-	-	-	-	-	-	-	-	-	-	-	-	-	-	-	-	-	-	-	-
220676	0,0%	0,0%	0,0%	0,0%	0,0%	0,2%	0,0%	0,0%	0,3%	Bacteria	Planctomycetes	Planctomycetia	Gemmatales	Isosphaeraceae	uncultured bacterium	-	-	-	-	-	-	-	-	-	-	-	-	-	-	-	-	-	-	-	-	-	-	-	-	-
19930	0,0%	0,0%	0,0%	0,1%	0,0%	0,0%	0,0%	0,0%	0,0%	Bacteria	Verrucomicrobia	Verrucomicrobiae	Verrucomicrobiales	Verrucomicrobiaceae	Prosthecobacter	-	-	-	-	-	-	-	-	-	-	-	-	-	-	-	-	-	-	-	-	-	-	-	-	-
741531	0,0%	0,0%	0,0%	0,0%	0,0%	0,1%	0,0%	0,1%	0,0%	Bacteria	Proteobacteria	Alphaproteobacteria	Rhodospirillales	Rhodospirillaceae	Azospirillum	-	napA	-	nirA	-	nirS	nirK	norB	nosZ	nifH	nifD	-	ureA	-	-	-	-	-	mmoX	-	laccase_Asco	laccase_Basidio	-	-	ppo
593384	0,1%	0,0%	0,0%	0,0%	0,0%	0,0%	0,0%	0,0%	0,0%	Bacteria	Proteobacteria	Betaproteobacteria	Burkholderiales	Oxalobacteraceae	Undibacterium	-	-	-	-	-	-	-	-	-	-	-	-	-	-	-	-	-	-	-	-	-	-	-	-	-
4396717	0,0%	0,0%	0,1%	0,0%	0,0%	0,0%	0,0%	0,0%	0,0%	Bacteria	Proteobacteria	Alphaproteobacteria	Rhizobiales	Methylobacteriaceae	Methylobacterium	-	napA	narG	-	-	-	nirK	-	nosZ	nifH	nifD	-	ureA	-	-	-	-	-	-	-	laccase_Asco	laccase_Basidio	-	-	ppo
4383099	0,0%	0,0%	0,0%	0,0%	0,0%	0,0%	0,1%	0,0%	0,0%	Bacteria	Proteobacteria	Deltaproteobacteria	Myxococcales	OM27	OM27 clade	-	-	-	-	-	-	-	-	-	-	-	-	-	-	-	-	-	-	-	-	-	-	-	-	-
3819514	0,0%	0,0%	0,0%	0,0%	0,0%	0,0%	0,0%	0,0%	0,3%	Bacteria	Proteobacteria	Deltaproteobacteria	Myxococcales		uncultured bacterium	-	-	-	-	-	-	-	-	-	-	-	-	-	-	-	-	-	-	-	-	-	-	-	-	-
4614	0,0%	0,0%	0,1%	0,0%	0,0%	0,0%	0,0%	0,0%	0,0%	Bacteria	Proteobacteria	Alphaproteobacteria	Rhizobiales	Hyphomicrobiaceae	Pedomicrobium	-	-	-	-	-	-	nirK	-	-	-	-	-	-	-	-	-	-	-	-	-	-	-	-	-	-
835586	0,0%	0,0%	0,0%	0,1%	0,0%	0,0%	0,0%	0,0%	0,0%	Bacteria	Proteobacteria	Gammaproteobacteria	Aeromonadales	Aeromonadaceae	uncultured bacterium	-	-	-	-	-	-	-	-	-	-	-	-	-	-	-	-	-	-	-	-	-	-	-	-	-
4483034	0,0%	0,0%	0,1%	0,0%	0,0%	0,0%	0,0%	0,0%	0,0%	Bacteria	Proteobacteria	Betaproteobacteria	Burkholderiales		Undibacterium	-	-	-	-	-	-	-	-	-	-	-	-	-	-	-	-	-	-	-	-	-	-	-	-	-
1131874	0,0%	0,0%	0,0%	0,0%	0,0%	0,0%	0,1%	0,0%	0,0%	Bacteria	Proteobacteria	Alphaproteobacteria	Rhizobiales		uncultured bacterium	-	-	-	-	-	-	-	-	-	-	-	-	-	-	-	-	-	-	-	-	-	-	-	-	-
215343	0,0%	0,0%	0,1%	0,0%	0,0%	0,0%	0,0%	0,0%	0,0%	Bacteria	Proteobacteria	Alphaproteobacteria	Ellin329		Rhizomicrobium	-	-	-	-	-	-	-	-	-	-	-	-	-	-	-	-	-	-	-	-	-	-	-	-	-
4420774	0,0%	0,0%	0,0%	0,0%	0,0%	0,0%	0,0%	0,0%	0,0%	Bacteria	Proteobacteria	Betaproteobacteria	Burkholderiales	Comamonadaceae	Aquincola	-	-	-	-	-	-	-	-	-	-	-	-	-	-	-	-	-	-	-	-	-	-	-	-	-
1121839	0,0%	0,0%	0,0%	0,0%	0,1%	0,0%	0,0%	0,0%	0,0%	Bacteria	Proteobacteria	Alphaproteobacteria	Rhizobiales	Methylocystaceae	Methylosinus	-	napA	-	nirA	-	-	-	-	-	nifH	nifD	-	ureA	-	-	-	-	-	mmoX	pmoA	-	-	-	-	-
3655114	0,0%	0,0%	0,0%	0,0%	0,0%	0,0%	0,0%	0,0%	0,0%	Bacteria	Acidobacteria	Acidobacteria-6	iii1-15		uncultured bacterium	-	-	-	-	-	-	-	-	-	-	-	-	-	-	-	-	-	-	-	-	-	-	-	-	-
836919	0,0%	0,1%	0,0%	0,0%	0,0%	0,0%	0,0%	0,0%	0,0%	Bacteria	Proteobacteria	Alphaproteobacteria	Rickettsiales	Rickettsiaceae	Wolbachia	-	-	-	-	-	-	-	-	-	-	-	-	-	-	-	-	-	-	-	-	-	-	-	-	-
153442	0,0%	0,0%	0,0%	0,0%	0,0%	0,0%	0,0%	0,0%	0,0%	Bacteria	Verrucomicrobia	Opitutae	Opitutales	Opitutaceae	Opitutus	-	napA	-	nirA	-	-	-	-	nosZ	-	-	nrfA	ureA	-	-	-	-	-	-	-	-	laccase_Basidio	-	-	-
1932541	0,0%	0,0%	0,0%	0,0%	0,0%	0,0%	0,0%	0,0%	0,1%	Bacteria	Proteobacteria	Betaproteobacteria	Rhodocyclales	Rhodocyclaceae	uncultured	-	-	-	-	-	-	-	-	-	-	-	-	-	-	-	-	-	-	-	-	-	-	-	lip	-
489457	0,0%	0,0%	0,0%	0,0%	0,1%	0,0%	0,0%	0,0%	0,1%	Bacteria	Proteobacteria	Alphaproteobacteria	Sphingomonadales	Sphingomonadaceae	Sphingomonas	-	napA	narG	nirA	-	-	nirK	norB	-	nifH	nifD	-	ureA	-	-	-	-	chb	mmoX	-	laccase_Asco	laccase_Basidio	-	-	ppo
1932546	0,0%	0,0%	0,1%	0,0%	0,0%	0,0%	0,0%	0,0%	0,0%	Bacteria	Proteobacteria	Betaproteobacteria	Burkholderiales	Comamonadaceae	uncultured	-	-	-	-	-	-	-	-	-	-	-	-	-	-	-	-	-	-	-	-	-	-	-	lip	-
253113	0,1%	0,0%	0,0%	0,0%	0,0%	0,0%	0,0%	0,0%	0,0%	Bacteria	Proteobacteria	Deltaproteobacteria	NB1-j	NB1-i	uncultured bacterium	-	-	-	-	-	-	-	-	-	-	-	-	-	-	-	-	-	-	-	-	-	-	-	-	-
4018589	0,0%	0,0%	0,0%	0,0%	0,0%	0,0%	0,0%	0,1%	0,1%	Bacteria	Verrucomicrobia	Opitutae	Opitutales	Opitutaceae	Opitutus	-	napA	-	nirA	-	-	-	-	nosZ	-	-	nrfA	ureA	-	-	-	-	-	-	-	-	laccase_Basidio	-	-	-
4437318	0,0%	0,0%	0,0%	0,0%	0,0%	0,0%	0,0%	0,0%	0,0%	Bacteria	Actinobacteria	Actinobacteria	Actinomycetales	Nocardioidaceae	Nocardioides	-	napA	narG	-	-	-	-	-	-	nifH	-	-	ureA	-	-	-	-	-	mmoX	pmoA	laccase_Asco	laccase_Basidio	-	-	ppo
805334	0,0%	0,0%	0,0%	0,0%	0,0%	0,0%	0,0%	0,0%	0,0%	Bacteria	Proteobacteria	Betaproteobacteria			uncultured bacterium	-	-	-	-	-	-	-	-	-	-	-	-	-	-	-	-	-	-	-	-	-	-	-	-	-
7543	0,0%	0,0%	0,0%	0,1%	0,0%	0,0%	0,0%	0,1%	0,0%	Bacteria	Proteobacteria	Gammaproteobacteria	Chromatiales	Chromatiaceae	Lamprocystis	-	-	-	-	-	-	-	-	-	-	-	-	-	-	-	-	-	-	-	-	-	-	-	-	-
4471583	0,0%	0,0%	0,0%	0,0%	0,0%	0,0%	0,0%	0,0%	0,0%	Bacteria	Verrucomicrobia	[Spartobacteria]	[Chthoniobacterales]	[Chthoniobacteraceae]	uncultured bacterium	-	-	-	-	-	-	-	-	-	-	-	-	-	-	-	-	-	-	-	-	-	-	-	-	-
236458	0,0%	0,0%	0,0%	0,0%	0,0%	0,0%	0,0%	0,0%	0,0%	Bacteria	Verrucomicrobia	[Spartobacteria]	[Chthoniobacterales]	[Chthoniobacteraceae]	Chthoniobacter	-	napA	-	-	-	-	nirK	-	-	-	-	nrfA	ureA	-	-	-	-	-	-	-	-	-	-	-	-
669183	0,0%	0,0%	0,0%	0,1%	0,0%	0,0%	0,0%	0,0%	0,0%	Bacteria	Proteobacteria	Gammaproteobacteria	[Marinicellales]	[Marinicellaceae]	Marinicella	-	-	-	-	-	-	-	-	-	-	-	-	-	-	-	-	-	-	-	-	-	-	-	-	-
4460355	0,0%	0,0%	0,0%	0,0%	0,0%	0,0%	0,0%	0,1%	0,1%	Bacteria	Proteobacteria	Betaproteobacteria	Rhodocyclales	Rhodocyclaceae	Massilia	-	napA	narG	-	-	-	-	norB	nosZ	-	-	-	ureA	-	-	-	-	chb	mmoX	-	laccase_Asco	laccase_Basidio	-	-	ppo
4460354	0,0%	0,0%	0,0%	0,0%	0,0%	0,0%	0,0%	0,0%	0,0%	Bacteria	Proteobacteria	Betaproteobacteria	Rhodocyclales	Rhodocyclaceae	Ferribacterium	-	-	-	-	-	-	-	-	-	-	-	-	-	-	-	-	-	-	-	-	-	-	-	-	-
1059681	0,0%	0,1%	0,0%	0,0%	0,0%	0,0%	0,0%	0,0%	0,0%	Bacteria	Planctomycetes	Planctomycetia	Gemmatales	Gemmataceae	uncultured	-	-	-	-	-	-	-	-	-	-	-	-	-	-	-	-	-	-	-	-	-	-	-	lip	-
4346000	0,0%	0,0%	0,1%	0,0%	0,0%	0,0%	0,0%	0,0%	0,1%	Bacteria	Proteobacteria	Alphaproteobacteria	Rhodobacterales	Rhodobacteraceae	Loktanella	-	napA	narG	nirA	-	-	-	norB	-	nifH	-	-	ureA	-	-	-	-	-	-	-	laccase_Asco	laccase_Basidio	-	-	ppo
649722	0,0%	0,0%	0,0%	0,0%	0,0%	0,0%	0,0%	0,0%	0,1%	Bacteria	Proteobacteria	Deltaproteobacteria	Myxococcales	Haliangiaceae	Haliangium	-	-	-	-	-	-	-	-	-	-	-	-	ureA	-	-	-	-	chb	-	-	-	-	-	-	-
90465	0,0%	0,0%	0,0%	0,0%	0,1%	0,0%	0,0%	0,0%	0,0%	Bacteria	Spirochaetes	Spirochaetes	Spirochaetales	Spirochaetaceae	Spirochaeta	-	-	-	nirA	-	-	-	-	-	nifH	nifD	-	-	-	-	-	-	-	-	-	-	-	-	-	-
141905	0,0%	0,0%	0,0%	0,0%	0,0%	0,0%	0,0%	0,0%	0,0%	Bacteria	Proteobacteria	Deltaproteobacteria	Myxococcales		Sandaracinus	-	napA	-	-	-	-	-	-	-	-	-	-	-	-	-	-	-	-	-	-	-	laccase_Basidio	-	-	ppo
805980	0,0%	0,0%	0,0%	0,0%	0,0%	0,0%	0,0%	0,1%	0,0%	Bacteria	Actinobacteria	Actinobacteria	Actinomycetales	Streptosporangiaceae	Microbispora	cellulolytic	napA	-	-	-	-	-	-	-	-	-	-	ureA	-	-	-	-	-	-	-	laccase_Asco	laccase_Basidio	-	-	-
4303858	0,0%	0,0%	0,0%	0,0%	0,0%	0,0%	0,0%	0,0%	0,0%	Bacteria	Proteobacteria	Betaproteobacteria	Burkholderiales	Comamonadaceae	uncultured	-	-	-	-	-	-	-	-	-	-	-	-	-	-	-	-	-	-	-	-	-	-	-	lip	-
700402	0,0%	0,0%	0,0%	0,0%	0,0%	0,0%	0,0%	0,0%	0,0%	Bacteria	Bacteroidetes	Flavobacteriia	Flavobacteriales	Flavobacteriaceae	Flavobacterium	cellulolytic	napA	-	nirA	-	-	nirK	norB	nosZ	-	-	-	ureA	-	-	-	-	-	-	-	laccase_Asco	laccase_Basidio	-	-	ppo
4464858	0,0%	0,0%	0,0%	0,0%	0,0%	0,0%	0,0%	0,0%	0,0%	Bacteria	Proteobacteria	Betaproteobacteria	Burkholderiales	Comamonadaceae	Comamonas	-	napA	-	nirA	-	nirS	nirK	norB	nosZ	-	-	-	ureA	-	-	SoxB	-	-	mmoX	-	-	laccase_Basidio	-	-	-
4337902	0,0%	0,0%	0,0%	0,0%	0,0%	0,1%	0,0%	0,0%	0,0%	Bacteria	Actinobacteria	Actinobacteria	Actinomycetales	Microbacteriaceae	Agromyces	cellulolytic	napA	narG	-	-	-	-	-	-	nifH	-	-	ureA	-	-	-	-	-	-	-	-	-	-	-	ppo
4170464	0,0%	0,0%	0,0%	0,1%	0,0%	0,0%	0,0%	0,0%	0,0%	Bacteria	Proteobacteria	Betaproteobacteria	Burkholderiales	Comamonadaceae	uncultured	-	-	-	-	-	-	-	-	-	-	-	-	-	-	-	-	-	-	-	-	-	-	-	lip	-
143360	0,0%	0,0%	0,1%	0,0%	0,0%	0,0%	0,0%	0,0%	0,0%	Bacteria	Proteobacteria	Epsilonproteobacteria	Campylobacterales	Helicobacteraceae	uncultured	-	-	-	-	-	-	-	-	-	-	-	-	-	-	-	-	-	-	-	-	-	-	-	lip	-
655879	0,0%	0,0%	0,0%	0,0%	0,0%	0,0%	0,0%	0,0%	0,0%	Bacteria	Armatimonadetes	[Fimbriimonadia]	[Fimbriimonadales]	[Fimbriimonadaceae]	uncultured bacterium	-	-	-	-	-	-	-	-	-	-	-	-	-	-	-	-	-	-	-	-	-	-	-	-	-
592038	0,0%	0,0%	0,1%	0,0%	0,0%	0,0%	0,0%	0,0%	0,0%	Bacteria	Verrucomicrobia	[Spartobacteria]	[Chthoniobacterales]	[Chthoniobacteraceae]	Chthoniobacter	-	napA	-	-	-	-	nirK	-	-	-	-	nrfA	ureA	-	-	-	-	-	-	-	-	-	-	-	-
49637	0,0%	0,0%	0,0%	0,0%	0,0%	0,0%	0,0%	0,0%	0,0%	Bacteria	Proteobacteria	Alphaproteobacteria	Rhizobiales	Xanthobacteraceae	Labrys	-	-	-	-	-	-	-	-	-	-	-	-	-	-	-	-	-	-	-	-	-	-	-	-	-
162144	0,0%	0,0%	0,0%	0,0%	0,0%	0,0%	0,0%	0,0%	0,0%	Bacteria	Proteobacteria	Alphaproteobacteria	Sphingomonadales	Sphingomonadaceae	Altererythrobacter	-	napA	narG	-	-	-	-	-	-	-	-	-	-	-	-	-	-	-	-	-	laccase_Asco	laccase_Basidio	-	-	ppo
668105	0,0%	0,0%	0,0%	0,0%	0,0%	0,0%	0,1%	0,0%	0,0%	Bacteria	Proteobacteria	Alphaproteobacteria	Sphingomonadales	Sphingomonadaceae	Novosphingobium	-	napA	-	-	-	-	-	norB	-	nifH	nifD	-	ureA	-	-	-	-	-	mmoX	-	laccase_Asco	laccase_Basidio	-	-	ppo
718326	0,0%	0,0%	0,0%	0,1%	0,0%	0,0%	0,0%	0,0%	0,0%	Bacteria	Proteobacteria	Alphaproteobacteria	Rhizobiales		Devosia	-	napA	-	-	-	-	nirK	-	-	nifH	-	-	ureA	-	-	-	-	chb	-	-	laccase_Asco	laccase_Basidio	-	-	ppo
4336414	0,0%	0,0%	0,0%	0,0%	0,0%	0,0%	0,0%	0,0%	0,0%	Bacteria	Proteobacteria	Deltaproteobacteria	Desulfobacterales	Desulfobulbaceae	Desulfobulbus	-	napA	-	-	-	-	-	-	-	nifH	nifD	nrfA	-	dsrA	dsrB	-	-	-	-	-	-	-	-	-	-
270402	0,0%	0,0%	0,0%	0,0%	0,0%	0,0%	0,0%	0,0%	0,0%	Bacteria	Proteobacteria	Betaproteobacteria	Burkholderiales	Comamonadaceae	uncultured	-	-	-	-	-	-	-	-	-	-	-	-	-	-	-	-	-	-	-	-	-	-	-	lip	-
4314531	0,0%	0,0%	0,0%	0,1%	0,0%	0,0%	0,0%	0,0%	0,0%	Bacteria	Proteobacteria	Alphaproteobacteria	Rhodobacterales	Rhodobacteraceae	Rhodobacter	-	napA	-	nirA	-	nirS	nirK	norB	nosZ	nifH	nifD	-	ureA	-	-	SoxB	-	-	mmoX	-	laccase_Asco	laccase_Basidio	-	-	ppo
4343457	0,0%	0,0%	0,0%	0,0%	0,0%	0,0%	0,0%	0,1%	0,0%	Bacteria	Proteobacteria	Gammaproteobacteria	Xanthomonadales	Xanthomonadaceae	Rhodanobacter	cellulolytic	napA	narG	-	-	nirS	nirK	norB	nosZ	-	-	-	-	-	-	-	-	-	mmoX	-	-	laccase_Basidio	-	-	ppo
4303696	0,0%	0,0%	0,1%	0,0%	0,0%	0,0%	0,0%	0,0%	0,0%	Bacteria	Proteobacteria	Alphaproteobacteria	Caulobacterales	Caulobacteraceae	Caulobacter	-	napA	narG	nirA	-	-	nirK	norB	-	-	-	-	-	-	-	-	-	chb	-	-	laccase_Asco	laccase_Basidio	-	-	ppo
141872	0,0%	0,0%	0,0%	0,0%	0,1%	0,0%	0,0%	0,0%	0,0%	Bacteria	Proteobacteria	Gammaproteobacteria	Methylococcales		Crenothrix	-	-	-	-	-	-	-	-	-	-	-	-	-	-	-	-	-	-	-	pmoA	-	-	-	-	-
826836	0,0%	0,0%	0,0%	0,0%	0,0%	0,2%	0,0%	0,0%	0,0%	Bacteria	Proteobacteria	Deltaproteobacteria	Desulfobacterales	Desulfobulbaceae	Desulfobulbus	-	napA	-	-	-	-	-	-	-	nifH	nifD	nrfA	-	dsrA	dsrB	-	-	-	-	-	-	-	-	-	-
4343458	0,0%	0,0%	0,0%	0,0%	0,1%	0,0%	0,0%	0,0%	0,0%	Bacteria	Proteobacteria	Gammaproteobacteria	Xanthomonadales	Sinobacteraceae	uncultured bacterium	-	-	-	-	-	-	-	-	-	-	-	-	-	-	-	-	-	-	-	-	-	-	-	-	-
4408129	0,0%	0,0%	0,0%	0,0%	0,0%	0,1%	0,0%	0,0%	0,1%	Bacteria	Proteobacteria	Gammaproteobacteria	Enterobacteriales	Enterobacteriaceae	Erwinia	cellulolytic	napA	narG	-	-	-	-	-	-	nifH	-	-	-	-	-	-	-	chb	-	-	laccase_Asco	laccase_Basidio	-	-	ppo
4299269	0,0%	0,0%	0,0%	0,0%	0,0%	0,3%	0,0%	0,0%	0,0%	Bacteria	Proteobacteria	Deltaproteobacteria	Myxococcales		Phaselicystis	-	-	-	-	-	-	-	-	-	-	-	-	-	-	-	-	-	-	-	-	-	-	-	-	-
4412141	0,0%	0,0%	0,0%	0,0%	0,0%	0,3%	0,0%	0,0%	0,0%	Bacteria	Proteobacteria	Gammaproteobacteria	Xanthomonadales	Xanthomonadaceae	Rhodanobacter	cellulolytic	napA	narG	-	-	nirS	nirK	norB	nosZ	-	-	-	-	-	-	-	-	-	mmoX	-	-	laccase_Basidio	-	-	ppo
155989	0,0%	0,0%	0,0%	0,0%	0,0%	0,0%	0,0%	0,0%	0,0%	Bacteria	Proteobacteria	Gammaproteobacteria	Xanthomonadales	Sinobacteraceae	uncultured bacterium	-	-	-	-	-	-	-	-	-	-	-	-	-	-	-	-	-	-	-	-	-	-	-	-	-
843469	0,1%	0,0%	0,0%	0,0%	0,0%	0,0%	0,0%	0,0%	0,0%	Bacteria	Proteobacteria	Betaproteobacteria	Burkholderiales	Comamonadaceae	uncultured	-	-	-	-	-	-	-	-	-	-	-	-	-	-	-	-	-	-	-	-	-	-	-	lip	-
4478794	0,0%	0,0%	0,0%	0,0%	0,0%	0,0%	0,0%	0,0%	0,1%	Bacteria	Acidobacteria	Acidobacteriia	Acidobacteriales	Koribacteraceae	uncultured	-	-	-	-	-	-	-	-	-	-	-	-	-	-	-	-	-	-	-	-	-	-	-	lip	-
4456518	0,0%	0,0%	0,0%	0,0%	0,0%	0,3%	0,0%	0,0%	0,0%	Bacteria	Verrucomicrobia	[Pedosphaerae]	[Pedosphaerales]	Ellin515	uncultured bacterium	-	-	-	-	-	-	-	-	-	-	-	-	-	-	-	-	-	-	-	-	-	-	-	-	-
4384016	0,0%	0,0%	0,0%	0,0%	0,0%	0,0%	0,0%	0,1%	0,0%	Bacteria	Proteobacteria	Betaproteobacteria	Burkholderiales	Comamonadaceae	uncultured	-	-	-	-	-	-	-	-	-	-	-	-	-	-	-	-	-	-	-	-	-	-	-	lip	-
1645783	0,0%	0,0%	0,0%	0,1%	0,0%	0,0%	0,0%	0,0%	0,0%	Bacteria	Planctomycetes	Phycisphaerae	Phycisphaerales		CL500-3	-	-	-	-	-	-	-	-	-	-	-	-	-	-	-	-	-	-	-	-	-	-	-	-	-
4305857	0,0%	0,0%	0,0%	0,0%	0,0%	0,0%	0,0%	0,0%	0,0%	Bacteria	Proteobacteria	Alphaproteobacteria	Rhodospirillales	Acetobacteraceae	Roseococcus	-	-	-	-	-	-	-	-	-	-	-	-	-	-	-	-	-	-	-	-	-	-	-	-	-
4467035	0,0%	0,1%	0,0%	0,0%	0,0%	0,0%	0,0%	0,0%	0,0%	Bacteria	Proteobacteria	Gammaproteobacteria	Aeromonadales	Aeromonadaceae	Tolumonas	-	napA	-	-	-	-	-	-	-	nifH	nifD	-	ureA	-	-	-	-	-	-	-	laccase_Asco	laccase_Basidio	-	-	ppo
4388776	0,0%	0,0%	0,1%	0,0%	0,0%	0,0%	0,0%	0,0%	0,0%	Bacteria	Proteobacteria	Alphaproteobacteria	Rhodospirillales	Acetobacteraceae	Acidocella	-	napA	narG	nirA	-	-	-	-	-	-	-	-	ureA	-	-	-	-	-	-	-	laccase_Asco	laccase_Basidio	-	-	ppo
1125844	0,0%	0,0%	0,0%	0,0%	0,1%	0,0%	0,0%	0,0%	0,0%	Bacteria	Proteobacteria	Alphaproteobacteria	Rhizobiales	Beijerinckiaceae	Methylocella	-	napA	narG	-	-	-	nirK	norB	-	nifH	nifD	-	ureA	-	-	-	-	-	mmoX	-	laccase_Asco	laccase_Basidio	-	-	ppo
4430327	0,0%	0,0%	0,0%	0,0%	0,0%	0,2%	0,0%	0,0%	0,0%	Bacteria	Planctomycetes	Planctomycetia	Planctomycetales	Planctomycetaceae	Planctomyces	-	napA	-	-	-	-	-	norB	-	-	-	nrfA	-	-	-	-	-	-	-	-	-	-	-	-	-
88754	0,0%	0,0%	0,0%	0,0%	0,0%	0,0%	0,1%	0,0%	0,0%	Bacteria	Proteobacteria	Alphaproteobacteria	Rhodospirillales	Acetobacteraceae	uncultured	-	-	-	-	-	-	-	-	-	-	-	-	-	-	-	-	-	-	-	-	-	-	-	lip	-
1114448	0,0%	0,0%	0,0%	0,0%	0,0%	0,0%	0,0%	0,0%	0,0%	Bacteria	Bacteroidetes	Flavobacteriia	Flavobacteriales	Cryomorphaceae	Fluviicola	-	-	-	nirA	-	-	-	norB	-	-	-	-	-	-	-	-	-	-	-	-	laccase_Asco	laccase_Basidio	-	-	ppo
1124243	0,0%	0,0%	0,0%	0,1%	0,0%	0,0%	0,0%	0,0%	0,0%	Bacteria	Proteobacteria	Gammaproteobacteria	Methylococcales	Methylococcaceae	Methylocaldum	-	-	-	-	-	-	-	-	-	nifH	-	-	-	-	-	-	-	-	mmoX	pmoA	-	-	-	-	-
1106411	0,0%	0,0%	0,0%	0,0%	0,0%	0,0%	0,0%	0,0%	0,0%	Bacteria	Armatimonadetes	Armatimonadia	FW68		uncultured bacterium	-	-	-	-	-	-	-	-	-	-	-	-	-	-	-	-	-	-	-	-	-	-	-	-	-
4465540	0,0%	0,0%	0,0%	0,0%	0,0%	0,0%	0,0%	0,2%	0,1%	Bacteria	Actinobacteria	Actinobacteria	Actinomycetales	Intrasporangiaceae	Terracoccus	-	-	-	-	-	-	-	-	-	-	-	-	-	-	-	-	-	-	-	-	-	-	-	-	-
216023	0,0%	0,0%	0,0%	0,0%	0,0%	0,0%	0,0%	0,0%	0,0%	Bacteria	Proteobacteria	Alphaproteobacteria	Rhodospirillales	Acetobacteraceae	uncultured	-	-	-	-	-	-	-	-	-	-	-	-	-	-	-	-	-	-	-	-	-	-	-	lip	-
820713	0,0%	0,0%	0,0%	0,0%	0,0%	0,0%	0,0%	0,0%	0,0%	Bacteria	Proteobacteria	Alphaproteobacteria	Sphingomonadales	Sphingomonadaceae	Novosphingobium	-	napA	-	-	-	-	-	norB	-	nifH	nifD	-	ureA	-	-	-	-	-	mmoX	-	laccase_Asco	laccase_Basidio	-	-	ppo
146122	0,0%	0,0%	0,0%	0,1%	0,0%	0,0%	0,0%	0,0%	0,0%	Bacteria	Actinobacteria	Actinobacteria	Actinomycetales		uncultured	-	-	-	-	-	-	-	-	-	-	-	-	-	-	-	-	-	-	-	-	-	-	-	lip	-
809293	0,0%	0,0%	0,0%	0,0%	0,0%	0,0%	0,0%	0,0%	0,0%	Bacteria	Proteobacteria	Deltaproteobacteria	Myxococcales		Sorangium	-	napA	-	-	-	-	nirK	norB	-	-	-	nrfA	ureA	-	-	-	-	chb	-	-	laccase_Asco	laccase_Basidio	-	-	ppo
1145194	0,0%	0,0%	0,0%	0,0%	0,0%	0,1%	0,0%	0,0%	0,0%	Bacteria	Proteobacteria	Gammaproteobacteria	Methylococcales	Crenotrichaceae	Methylosoma	-	-	-	-	-	-	-	-	-	nifH	-	-	-	-	-	-	-	-	-	pmoA	-	-	-	-	-
748865	0,0%	0,0%	0,0%	0,1%	0,0%	0,1%	0,0%	0,0%	0,0%	Bacteria	Proteobacteria	Alphaproteobacteria			uncultured bacterium	-	-	-	-	-	-	-	-	-	-	-	-	-	-	-	-	-	-	-	-	-	-	-	-	-
4397450	0,0%	0,0%	0,0%	0,0%	0,0%	0,2%	0,0%	0,0%	0,0%	Bacteria	Proteobacteria	Gammaproteobacteria	Methylococcales		Crenothrix	-	-	-	-	-	-	-	-	-	-	-	-	-	-	-	-	-	-	-	pmoA	-	-	-	-	-
4442656	0,0%	0,0%	0,1%	0,0%	0,0%	0,0%	0,0%	0,0%	0,0%	Bacteria	Proteobacteria	Alphaproteobacteria	Rhodospirillales	Acetobacteraceae	uncultured	-	-	-	-	-	-	-	-	-	-	-	-	-	-	-	-	-	-	-	-	-	-	-	lip	-
275472	0,0%	0,0%	0,0%	0,0%	0,0%	0,2%	0,0%	0,0%	0,0%	Bacteria	Firmicutes	Clostridia	Clostridiales	Clostridiaceae	Clostridium sensu stricto 5	cellulolytic	-	-	-	-	-	-	-	-	-	-	-	-	-	-	-	-	-	-	-	-	-	-	-	-
4434317	0,0%	0,0%	0,0%	0,0%	0,0%	0,0%	0,0%	0,0%	0,0%	Bacteria	Proteobacteria	Gammaproteobacteria	Oceanospirillales	Oceanospirillaceae	Marinomonas	-	napA	-	nirA	-	-	-	-	-	-	-	-	ureA	-	-	-	-	-	-	-	laccase_Asco	laccase_Basidio	-	-	ppo
4389358	0,0%	0,0%	0,1%	0,0%	0,0%	0,0%	0,0%	0,0%	0,0%	Bacteria	Planctomycetes	Planctomycetia	Gemmatales	Gemmataceae	uncultured	-	-	-	-	-	-	-	-	-	-	-	-	-	-	-	-	-	-	-	-	-	-	-	lip	-
4479989	0,0%	0,0%	0,0%	0,0%	0,0%	0,0%	0,0%	0,0%	0,0%	Bacteria	Firmicutes	Bacilli	Lactobacillales	Streptococcaceae	Streptococcus	-	napA	narG	-	-	nirS	nirK	norB	nosZ	-	-	-	ureA	-	-	-	-	-	-	-	laccase_Asco	laccase_Basidio	-	-	ppo

4425792	0,0%	0,0%	0,0%	0,1%	0,0%	0,1%	0,0%	0,0%	0,0%	Bacteria	Actinobacteria	Actinobacteria	Actinomycetales	Streptomycetaceae	Streptomyces	cellulolytic	napA	narG	nirA	-	-	-	norB	-	nifH	-	-	ureA	-	-	-	-	chb	mmoX	-	laccase_Asco	laccase_Basidio	-	-	ppo
143882	0,0%	0,0%	0,0%	0,0%	0,0%	0,2%	0,0%	0,0%	0,0%	Bacteria	Proteobacteria	Deltaproteobacteria	Myxococcales	OM27	OM27 clade	-	-	-	-	-	-	-	-	-	-	-	-	-	-	-	-	-	-	-	-	-	-	-	-	-
4423471	0,0%	0,0%	0,0%	0,1%	0,0%	0,0%	0,0%	0,0%	0,0%	Bacteria	Proteobacteria	Betaproteobacteria	Rhodocyclales	Rhodocyclaceae	Dechloromonas	-	napA	narG	-	-	nirS	-	norB	nosZ	nifH	nifD	-	ureA	-	-	SoxB	-	-	mmoX	-	-	-	-	-	-
156611	0,0%	0,0%	0,0%	0,0%	0,0%	0,0%	0,0%	0,0%	0,0%	Bacteria	Acidobacteria	Acidobacteriia	Acidobacteriales	Acidobacteriaceae	uncultured	cellulolytic	-	-	-	-	-	-	-	-	-	-	-	-	-	-	-	-	-	-	-	-	-	-	lip	-
4325011	0,0%	0,0%	0,1%	0,0%	0,0%	0,0%	0,0%	0,0%	0,0%	Bacteria	Proteobacteria	Betaproteobacteria	Burkholderiales	Comamonadaceae	uncultured	-	-	-	-	-	-	-	-	-	-	-	-	-	-	-	-	-	-	-	-	-	-	-	lip	-
4367782	0,0%	0,0%	0,0%	0,0%	0,0%	0,0%	0,1%	0,0%	0,0%	Bacteria	Acidobacteria	Acidobacteriia	Acidobacteriales	Acidobacteriaceae	Bryocella	cellulolytic	-	-	-	-	-	-	-	-	-	-	-	-	-	-	-	-	-	-	-	-	-	-	-	-
1128417	0,0%	0,0%	0,0%	0,0%	0,0%	0,0%	0,0%	0,0%	0,1%	Bacteria	Actinobacteria	Acidimicrobiia	Acidimicrobiales		uncultured bacterium	-	-	-	-	-	-	-	-	-	-	-	-	-	-	-	-	-	-	-	-	-	-	-	-	-
555696	0,0%	0,0%	0,0%	0,0%	0,0%	0,0%	0,0%	0,0%	0,0%	Bacteria	Cyanobacteria	Oscillatoriophycideae	Oscillatoriales	Phormidiaceae	uncultured cyanobacterium	-	-	-	-	-	-	-	-	-	-	-	-	-	-	-	-	-	-	-	-	-	-	-	-	-
156986	0,0%	0,0%	0,0%	0,0%	0,0%	0,2%	0,0%	0,0%	0,0%	Bacteria	Proteobacteria	Gammaproteobacteria	Xanthomonadales	Sinobacteraceae	uncultured	-	-	-	-	-	-	-	-	-	-	-	-	-	-	-	-	-	-	-	-	-	-	-	lip	-
111162	0,1%	0,0%	0,0%	0,0%	0,0%	0,0%	0,0%	0,0%	0,0%	Bacteria	Planctomycetes	Phycisphaerae	WD2101		uncultured bacterium	-	-	-	-	-	-	-	-	-	-	-	-	-	-	-	-	-	-	-	-	-	-	-	-	-
105993	0,0%	0,0%	0,0%	0,0%	0,0%	0,0%	0,0%	0,0%	0,0%	Bacteria	Proteobacteria	Alphaproteobacteria	Rhizobiales	Hyphomicrobiaceae	Devosia	-	napA	-	-	-	-	nirK	-	-	nifH	-	-	ureA	-	-	-	-	chb	-	-	laccase_Asco	laccase_Basidio	-	-	ppo
285591	0,0%	0,0%	0,0%	0,0%	0,0%	0,0%	0,0%	0,0%	0,0%	Bacteria	Actinobacteria	Thermoleophilia	Solirubrobacterales		uncultured bacterium	-	-	-	-	-	-	-	-	-	-	-	-	-	-	-	-	-	-	-	-	-	-	-	-	-
568926	0,0%	0,0%	0,0%	0,0%	0,0%	0,0%	0,0%	0,0%	0,0%	Bacteria	Bacteroidetes	Cytophagia	Cytophagales	Cytophagaceae	Hymenobacter	cellulolytic	-	-	-	-	-	-	-	nosZ	-	-	-	ureA	-	-	-	-	-	-	-	-	laccase_Basidio	-	-	ppo
841979	0,0%	0,0%	0,0%	0,0%	0,0%	0,0%	0,1%	0,0%	0,0%	Bacteria	Proteobacteria	Alphaproteobacteria	Rhodospirillales	Acetobacteraceae	uncultured	-	-	-	-	-	-	-	-	-	-	-	-	-	-	-	-	-	-	-	-	-	-	-	lip	-
353499	0,0%	0,0%	0,0%	0,0%	0,0%	0,0%	0,0%	0,0%	0,0%	Bacteria	Proteobacteria	Deltaproteobacteria	Bdellovibrionales	Bdellovibrionaceae	Bdellovibrio	-	-	-	-	-	-	nirK	norB	-	-	-	nrfA	-	-	-	-	-	-	-	-	laccase_Asco	laccase_Basidio	-	-	ppo
4387187	0,0%	0,0%	0,0%	0,0%	0,0%	0,0%	0,0%	0,0%	0,0%	Bacteria	Bacteroidetes	[Saprospirae]	[Saprospirales]	Chitinophagaceae	Sediminibacterium	-	-	-	-	-	-	-	-	-	-	-	-	-	-	-	-	-	-	-	-	-	-	-	-	-
4483895	0,0%	0,0%	0,0%	0,0%	0,0%	0,0%	0,0%	0,2%	0,0%	Bacteria	Proteobacteria	Gammaproteobacteria	Aeromonadales	Aeromonadaceae	uncultured bacterium	-	-	-	-	-	-	-	-	-	-	-	-	-	-	-	-	-	-	-	-	-	-	-	-	-
1132285	0,0%	0,0%	0,0%	0,0%	0,0%	0,0%	0,1%	0,0%	0,0%	Bacteria	Planctomycetes	Planctomycetia	Gemmatales	Gemmataceae	Gemmata	-	-	-	-	-	-	-	-	-	-	-	-	-	-	-	-	-	-	-	-	-	-	-	-	-
137956	0,0%	0,0%	0,0%	0,0%	0,0%	0,0%	0,1%	0,0%	0,0%	Bacteria	Actinobacteria	Actinobacteria	Actinomycetales	Streptomycetaceae	Streptomyces	cellulolytic	napA	narG	nirA	-	-	-	norB	-	nifH	-	-	ureA	-	-	-	-	chb	mmoX	-	laccase_Asco	laccase_Basidio	-	-	ppo
158804	0,0%	0,1%	0,0%	0,0%	0,0%	0,0%	0,0%	0,0%	0,0%	Bacteria	Actinobacteria	Actinobacteria	Actinomycetales	Nocardioidaceae	Marmoricola	-	napA	-	-	-	-	-	-	-	-	-	-	-	-	-	-	-	-	-	-	-	-	-	-	-
1503863	0,0%	0,0%	0,0%	0,0%	0,0%	0,0%	0,0%	0,1%	0,0%	Bacteria	Proteobacteria	Gammaproteobacteria	Xanthomonadales	Sinobacteraceae	Nevskia	-	-	-	-	-	-	-	-	-	-	-	-	-	-	-	-	-	-	-	-	-	-	-	-	-
194242	0,0%	0,0%	0,0%	0,0%	0,0%	0,1%	0,0%	0,0%	0,0%	Bacteria	TM7	TM7-3	EW055		uncultured bacterium	-	-	-	-	-	-	-	-	-	-	-	-	-	-	-	-	-	-	-	-	-	-	-	-	-
4467810	0,0%	0,0%	0,0%	0,0%	0,1%	0,0%	0,0%	0,0%	0,0%	Bacteria	Firmicutes	Bacilli	Bacillales	Bacillaceae	Geobacillus	cellulolytic	napA	narG	-	-	-	nirK	norB	nosZ	-	-	-	ureA	-	-	-	-	-	-	-	laccase_Asco	laccase_Basidio	-	-	ppo
4480120	0,0%	0,0%	0,0%	0,0%	0,0%	0,0%	0,0%	0,1%	0,0%	Bacteria	Proteobacteria	Deltaproteobacteria	Desulfuromonadales	Geobacteraceae	Geobacter	-	napA	narG	-	-	-	-	norB	-	nifH	nifD	nrfA	-	-	-	-	-	-	-	-	laccase_Asco	laccase_Basidio	-	-	ppo
4480121	0,0%	0,0%	0,0%	0,0%	0,0%	0,0%	0,0%	0,0%	0,0%	Bacteria	Proteobacteria	Deltaproteobacteria	Myxococcales		Phaselicystis	-	-	-	-	-	-	-	-	-	-	-	-	-	-	-	-	-	-	-	-	-	-	-	-	-
4468125	0,0%	0,0%	0,0%	0,0%	0,0%	0,0%	0,1%	0,0%	0,0%	Bacteria	Actinobacteria	Actinobacteria	Actinomycetales	Nocardiaceae	Rhodococcus	-	napA	narG	nirA	-	-	nirK	norB	-	-	-	-	ureA	-	-	-	-	-	mmoX	-	laccase_Asco	laccase_Basidio	-	-	ppo
4432796	0,0%	0,0%	0,1%	0,0%	0,0%	0,0%	0,0%	0,0%	0,0%	Bacteria	Proteobacteria	Gammaproteobacteria	Pseudomonadales	Pseudomonadaceae	Pseudomonas	cellulolytic	napA	narG	nirA	-	nirS	nirK	norB	nosZ	nifH	nifD	-	ureA	-	dsrB	SoxB	-	-	mmoX	-	laccase_Asco	laccase_Basidio	-	-	ppo
1918591	0,0%	0,0%	0,0%	0,0%	0,0%	0,0%	0,0%	0,0%	0,0%	Bacteria	Planctomycetes	Planctomycetia	Gemmatales	Gemmataceae	Gemmata	-	-	-	-	-	-	-	-	-	-	-	-	-	-	-	-	-	-	-	-	-	-	-	-	-
4418395	0,0%	0,0%	0,0%	0,0%	0,0%	0,2%	0,0%	0,0%	0,0%	Bacteria	Proteobacteria	Alphaproteobacteria	Rhizobiales	Methylobacteriaceae	Methylobacterium	-	napA	narG	-	-	-	nirK	-	nosZ	nifH	nifD	-	ureA	-	-	-	-	-	-	-	laccase_Asco	laccase_Basidio	-	-	ppo
4045633	0,0%	0,0%	0,0%	0,1%	0,0%	0,0%	0,0%	0,0%	0,0%	Bacteria	Proteobacteria	Alphaproteobacteria	Rhizobiales	Rhizobiaceae	Rhizobium	-	napA	narG	nirA	-	-	nirK	norB	nosZ	nifH	nifD	nrfA	ureA	-	-	-	-	chb	mmoX	-	laccase_Asco	laccase_Basidio	-	-	ppo
338140	0,0%	0,0%	0,0%	0,0%	0,0%	0,0%	0,0%	0,0%	0,0%	Bacteria	Proteobacteria	Gammaproteobacteria	Pseudomonadales	Pseudomonadaceae	Pseudomonas	cellulolytic	napA	narG	nirA	-	nirS	nirK	norB	nosZ	nifH	nifD	-	ureA	-	dsrB	SoxB	-	-	mmoX	-	laccase_Asco	laccase_Basidio	-	-	ppo
541345	0,0%	0,0%	0,1%	0,0%	0,0%	0,0%	0,0%	0,0%	0,0%	Bacteria	Verrucomicrobia	[Pedosphaerae]	[Pedosphaerales]	auto67_4W	uncultured bacterium	-	-	-	-	-	-	-	-	-	-	-	-	-	-	-	-	-	-	-	-	-	-	-	-	-
730204	0,0%	0,0%	0,0%	0,0%	0,0%	0,0%	0,0%	0,0%	0,0%	Bacteria	Proteobacteria	Alphaproteobacteria	Rhodospirillales	Rhodospirillaceae	Azospirillum	-	napA	-	nirA	-	nirS	nirK	norB	nosZ	nifH	nifD	-	ureA	-	-	-	-	-	mmoX	-	laccase_Asco	laccase_Basidio	-	-	ppo
1127233	0,0%	0,0%	0,0%	0,0%	0,0%	0,0%	0,0%	0,1%	0,0%	Bacteria	Gemmatimonadetes	Gemm-1			uncultured	-	-	-	-	-	-	-	-	-	-	-	-	-	-	-	-	-	-	-	-	-	-	-	lip	-
4442386	0,0%	0,0%	0,0%	0,0%	0,0%	0,1%	0,0%	0,0%	0,0%	Bacteria	Firmicutes	Bacilli	Bacillales	Bacillaceae	Bacillus	cellulolytic	napA	narG	nirA	-	nirS	nirK	norB	nosZ	nifH	nifD	nrfA	ureA	-	-	-	-	-	mmoX	-	laccase_Asco	laccase_Basidio	-	-	ppo
525171	0,0%	0,0%	0,0%	0,0%	0,0%	0,0%	0,0%	0,0%	0,0%	Bacteria	Proteobacteria	Deltaproteobacteria	Bdellovibrionales	Bdellovibrionaceae	Bdellovibrio	-	-	-	-	-	-	nirK	norB	-	-	-	nrfA	-	-	-	-	-	-	-	-	laccase_Asco	laccase_Basidio	-	-	ppo
4380157	0,0%	0,0%	0,0%	0,0%	0,0%	0,0%	0,0%	0,0%	0,0%	Bacteria	Proteobacteria	Betaproteobacteria	Methylophilales	Methylophilaceae	Methylotenera	-	napA	-	nirA	-	-	nirK	norB	-	-	-	-	ureA	-	-	-	-	-	-	-	-	-	-	-	-
4373617	0,0%	0,0%	0,1%	0,0%	0,0%	0,0%	0,0%	0,0%	0,0%	Bacteria	Proteobacteria	Gammaproteobacteria	Xanthomonadales	Sinobacteraceae	uncultured bacterium	-	-	-	-	-	-	-	-	-	-	-	-	-	-	-	-	-	-	-	-	-	-	-	-	-
4386631	0,0%	0,0%	0,0%	0,0%	0,0%	0,0%	0,1%	0,0%	0,0%	Bacteria	Proteobacteria	Betaproteobacteria	Burkholderiales	Comamonadaceae	uncultured	-	-	-	-	-	-	-	-	-	-	-	-	-	-	-	-	-	-	-	-	-	-	-	lip	-
925585	0,0%	0,0%	0,0%	0,0%	0,0%	0,0%	0,0%	0,0%	0,0%	Bacteria	Planctomycetes	Planctomycetia	Planctomycetales	Planctomycetaceae	Planctomyces	-	napA	-	-	-	-	-	norB	-	-	-	nrfA	-	-	-	-	-	-	-	-	-	-	-	-	-
154822	0,0%	0,0%	0,0%	0,0%	0,0%	0,0%	0,0%	0,0%	0,0%	Bacteria	Acidobacteria	DA052	Ellin6513		uncultured bacterium	-	-	-	-	-	-	-	-	-	-	-	-	-	-	-	-	-	-	-	-	-	-	-	-	-
88989	0,0%	0,0%	0,0%	0,0%	0,0%	0,0%	0,0%	0,0%	0,0%	Bacteria	Cyanobacteria	4C0d-2	MLE1-12		uncultured bacterium	-	-	-	-	-	-	-	-	-	-	-	-	-	-	-	-	-	-	-	-	-	-	-	-	-
751207	0,0%	0,0%	0,0%	0,0%	0,0%	0,0%	0,1%	0,0%	0,0%	Bacteria	Proteobacteria	Betaproteobacteria	Burkholderiales	Comamonadaceae	Pelomonas	-	napA	narG	nirA	-	nirS	-	norB	nosZ	nifH	-	-	ureA	-	-	-	-	-	-	-	-	-	-	-	-
4332073	0,0%	0,1%	0,0%	0,0%	0,0%	0,0%	0,0%	0,0%	0,0%	Bacteria	Actinobacteria	OPB41			uncultured	-	-	-	-	-	-	-	-	-	-	-	-	-	-	-	-	-	-	-	-	-	-	-	lip	-
615729	0,0%	0,0%	0,0%	0,0%	0,0%	0,0%	0,1%	0,0%	0,0%	Bacteria	Actinobacteria	Acidimicrobiia	Acidimicrobiales		uncultured actinobacterium	-	-	-	-	-	-	-	-	-	-	-	-	-	-	-	-	-	-	-	-	-	-	-	-	-
711776	0,0%	0,0%	0,0%	0,0%	0,0%	0,1%	0,0%	0,0%	0,1%	Bacteria	Proteobacteria	Alphaproteobacteria	Rhizobiales	Beijerinckiaceae	Methylocella	-	napA	narG	-	-	-	nirK	norB	-	nifH	nifD	-	ureA	-	-	-	-	-	mmoX	-	laccase_Asco	laccase_Basidio	-	-	ppo
4450823	0,0%	0,0%	0,1%	0,0%	0,0%	0,0%	0,0%	0,0%	0,0%	Bacteria	Acidobacteria	Acidobacteriia	Acidobacteriales	Koribacteraceae	uncultured	-	-	-	-	-	-	-	-	-	-	-	-	-	-	-	-	-	-	-	-	-	-	-	lip	-
4456113	0,0%	0,0%	0,0%	0,0%	0,0%	0,1%	0,0%	0,0%	0,0%	Bacteria	Proteobacteria	Betaproteobacteria	Burkholderiales	Comamonadaceae	uncultured	-	-	-	-	-	-	-	-	-	-	-	-	-	-	-	-	-	-	-	-	-	-	-	lip	-
1136141	0,0%	0,0%	0,0%	0,0%	0,1%	0,0%	0,0%	0,0%	0,0%	Bacteria	Proteobacteria	Alphaproteobacteria	Rhodospirillales	Acetobacteraceae	uncultured bacterium	-	-	-	-	-	-	-	-	-	-	-	-	-	-	-	-	-	-	-	-	-	-	-	-	-
4479241	0,0%	0,0%	0,0%	0,0%	0,0%	0,0%	0,1%	0,0%	0,0%	Bacteria	Proteobacteria	Alphaproteobacteria	Rhodospirillales	Rhodospirillaceae	Telmatospirillum	-	-	-	-	-	-	-	-	-	nifH	-	-	-	-	-	-	-	-	-	-	-	-	-	-	-
533055	0,0%	0,0%	0,0%	0,0%	0,0%	0,2%	0,0%	0,0%	0,0%	Bacteria	Verrucomicrobia	[Pedosphaerae]	[Pedosphaerales]	auto67_4W	uncultured bacterium	-	-	-	-	-	-	-	-	-	-	-	-	-	-	-	-	-	-	-	-	-	-	-	-	-
225760	0,0%	0,0%	0,0%	0,1%	0,0%	0,0%	0,0%	0,0%	0,1%	Bacteria	Cyanobacteria	Oscillatoriophycideae	Oscillatoriales	Phormidiaceae	Microcoleus	-	napA	-	nirA	-	-	-	-	-	nifH	nifD	-	ureA	-	-	-	-	-	-	-	-	-	-	-	-
4421830	0,0%	0,0%	0,0%	0,0%	0,1%	0,0%	0,0%	0,0%	0,0%	Bacteria	Proteobacteria	Betaproteobacteria	Burkholderiales	Comamonadaceae	uncultured	-	-	-	-	-	-	-	-	-	-	-	-	-	-	-	-	-	-	-	-	-	-	-	lip	-
4470402	0,0%	0,0%	0,0%	0,0%	0,0%	0,0%	0,0%	0,0%	0,0%	Bacteria	Proteobacteria	Deltaproteobacteria	Bdellovibrionales	Bacteriovoracaceae	Peredibacter	-	-	-	-	-	-	-	-	-	-	-	-	-	-	-	-	-	-	-	-	-	-	-	-	-
2194873	0,0%	0,0%	0,0%	0,0%	0,0%	0,0%	0,0%	0,0%	0,0%	Bacteria	Proteobacteria	Betaproteobacteria	Burkholderiales	Comamonadaceae	uncultured	-	-	-	-	-	-	-	-	-	-	-	-	-	-	-	-	-	-	-	-	-	-	-	lip	-
819061	0,0%	0,0%	0,0%	0,0%	0,0%	0,0%	0,0%	0,0%	0,0%	Bacteria	Verrucomicrobia	[Pedosphaerae]	[Pedosphaerales]	auto67_4W	uncultured bacterium	-	-	-	-	-	-	-	-	-	-	-	-	-	-	-	-	-	-	-	-	-	-	-	-	-
206514	0,0%	0,0%	0,0%	0,0%	0,0%	0,0%	0,0%	0,0%	0,0%	Bacteria	Proteobacteria	Alphaproteobacteria	Ellin329		uncultured Gemmatimonadetes bacterium	-	-	-	-	-	-	-	-	-	-	-	-	-	-	-	-	-	-	-	-	-	-	-	-	-
2194877	0,0%	0,0%	0,0%	0,1%	0,0%	0,0%	0,0%	0,0%	0,0%	Bacteria	Proteobacteria	Betaproteobacteria	Burkholderiales	Comamonadaceae	Variovorax	-	napA	narG	nirA	-	-	-	norB	-	-	-	-	ureA	-	-	-	-	-	mmoX	-	-	-	-	-	-
1106090	0,0%	0,0%	0,0%	0,0%	0,0%	0,0%	0,0%	0,0%	0,0%	Bacteria	Proteobacteria	Alphaproteobacteria	Ellin329		Rhizomicrobium	-	-	-	-	-	-	-	-	-	-	-	-	-	-	-	-	-	-	-	-	-	-	-	-	-
282360	0,0%	0,0%	0,0%	0,0%	0,0%	0,2%	0,0%	0,0%	0,0%	Bacteria	Actinobacteria	Actinobacteria	Actinomycetales	Corynebacteriaceae	Corynebacterium	-	napA	narG	-	-	-	nirK	norB	-	-	-	nrfA	ureA	-	-	-	-	-	-	-	laccase_Asco	laccase_Basidio	-	-	ppo
4401307	0,0%	0,0%	0,0%	0,0%	0,0%	0,0%	0,0%	0,0%	0,0%	Bacteria	Proteobacteria	Alphaproteobacteria	Rhizobiales	Hyphomicrobiaceae	uncultured	-	-	-	-	-	-	-	-	-	-	-	-	-	-	-	-	-	-	-	-	-	-	-	lip	-
4455982	0,0%	0,0%	0,0%	0,0%	0,0%	0,1%	0,0%	0,0%	0,1%	Bacteria	Actinobacteria	Actinobacteria	Actinomycetales	Microbacteriaceae	Pseudoclavibacter	cellulolytic	-	-	-	-	-	-	-	-	-	-	-	-	-	-	-	-	-	-	-	-	-	-	-	-
546919	0,0%	0,0%	0,0%	0,0%	0,0%	0,0%	0,0%	0,0%	0,0%	Bacteria	Proteobacteria	Gammaproteobacteria	Xanthomonadales	Sinobacteraceae	Nevskia	-	-	-	-	-	-	-	-	-	-	-	-	-	-	-	-	-	-	-	-	-	-	-	-	-
74685	0,0%	0,0%	0,0%	0,0%	0,0%	0,0%	0,1%	0,0%	0,0%	Bacteria	Proteobacteria	Betaproteobacteria	Burkholderiales	Comamonadaceae	Acidovorax	-	napA	narG	nirA	-	nirS	nirK	norB	nosZ	-	-	-	ureA	-	-	-	-	-	mmoX	-	-	laccase_Basidio	-	-	ppo
4365307	0,0%	0,0%	0,0%	0,0%	0,0%	0,0%	0,0%	0,0%	0,0%	Bacteria	Proteobacteria	Alphaproteobacteria			Skermanella	-	napA	-	-	-	-	-	-	-	nifH	nifD	-	ureA	-	-	-	-	-	mmoX	-	laccase_Asco	laccase_Basidio	-	-	ppo
4361862	0,0%	0,0%	0,0%	0,1%	0,0%	0,0%	0,0%	0,0%	0,0%	Bacteria	Actinobacteria	Acidimicrobiia	Acidimicrobiales		uncultured Actinomycetales bacterium	-	-	-	-	-	-	-	-	-	-	-	-	-	-	-	-	-	-	-	-	-	-	-	-	-
4478861	0,0%	0,0%	0,1%	0,0%	0,0%	0,0%	0,0%	0,0%	0,0%	Bacteria	Proteobacteria	Gammaproteobacteria	Pseudomonadales	Pseudomonadaceae	Pseudomonas	cellulolytic	napA	narG	nirA	-	nirS	nirK	norB	nosZ	nifH	nifD	-	ureA	-	dsrB	SoxB	-	-	mmoX	-	laccase_Asco	laccase_Basidio	-	-	ppo
89212	0,0%	0,0%	0,0%	0,0%	0,0%	0,0%	0,0%	0,0%	0,1%	Bacteria	Proteobacteria	Alphaproteobacteria	Sphingomonadales	Sphingomonadaceae	Novosphingobium	-	napA	-	-	-	-	-	norB	-	nifH	nifD	-	ureA	-	-	-	-	-	mmoX	-	laccase_Asco	laccase_Basidio	-	-	ppo
1018733	0,0%	0,0%	0,0%	0,0%	0,0%	0,0%	0,0%	0,0%	0,0%	Bacteria	Bacteroidetes	[Saprospirae]	[Saprospirales]	Saprospiraceae	uncultured	-	-	-	-	-	-	-	-	-	-	-	-	-	-	-	-	-	-	-	-	-	-	-	lip	-
1011380	0,0%	0,0%	0,0%	0,0%	0,0%	0,0%	0,0%	0,0%	0,0%	Bacteria	Actinobacteria	Thermoleophilia	Solirubrobacterales	Conexibacteraceae	uncultured bacterium	-	-	-	-	-	-	-	-	-	-	-	-	-	-	-	-	-	-	-	-	-	-	-	-	-
4339358	0,0%	0,0%	0,0%	0,0%	0,0%	0,0%	0,0%	0,0%	0,1%	Bacteria	Proteobacteria	Alphaproteobacteria	Caulobacterales	Caulobacteraceae	Caulobacter	-	napA	narG	nirA	-	-	nirK	norB	-	-	-	-	-	-	-	-	-	chb	-	-	laccase_Asco	laccase_Basidio	-	-	ppo
570864	0,0%	0,0%	0,0%	0,0%	0,0%	0,0%	0,0%	0,0%	0,0%	Bacteria	Verrucomicrobia	[Spartobacteria]	[Chthoniobacterales]	[Chthoniobacteraceae]	Candidatus Xiphinematobacter	-	-	-	-	-	-	-	-	-	-	-	-	-	-	-	-	-	-	-	-	-	-	-	-	-
5808	0,0%	0,0%	0,0%	0,0%	0,0%	0,0%	0,0%	0,0%	0,1%	Bacteria	Proteobacteria	Alphaproteobacteria	Rhodobacterales	Rhodobacteraceae	Amaricoccus	-	-	-	-	-	-	-	-	-	-	-	-	-	-	-	-	-	-	-	-	-	-	-	-	-
352211	0,0%	0,0%	0,0%	0,0%	0,0%	0,0%	0,0%	0,0%	0,0%	Bacteria	Proteobacteria	Alphaproteobacteria	Sphingomonadales	Sphingomonadaceae	uncultured bacterium	-	-	-	-	-	-	-	-	-	-	-	-	-	-	-	-	-	-	-	-	-	-	-	-	-
643362	0,0%	0,0%	0,0%	0,0%	0,0%	0,0%	0,0%	0,1%	0,0%	Bacteria	Firmicutes	Clostridia	Clostridiales	Clostridiaceae	Clostridium sensu stricto 1	cellulolytic	-	-	-	-	-	-	-	-	-	-	-	-	-	-	-	-	-	-	-	-	-	-	-	-
819663	0,0%	0,0%	0,0%	0,0%	0,0%	0,0%	0,0%	0,0%	0,0%	Bacteria	Proteobacteria	Alphaproteobacteria	Rhizobiales	Hyphomicrobiaceae	uncultured	-	-	-	-	-	-	-	-	-	-	-	-	-	-	-	-	-	-	-	-	-	-	-	lip	-
890930	0,0%	0,0%	0,0%	0,1%	0,0%	0,0%	0,0%	0,0%	0,0%	Bacteria	Proteobacteria	Alphaproteobacteria	Rhodospirillales	Acetobacteraceae	uncultured	-	-	-	-	-	-	-	-	-	-	-	-	-	-	-	-	-	-	-	-	-	-	-	lip	-
897662	0,0%	0,0%	0,0%	0,0%	0,0%	0,0%	0,0%	0,0%	0,0%	Bacteria	Verrucomicrobia	[Spartobacteria]	[Chthoniobacterales]	[Chthoniobacteraceae]	Candidatus Xiphinematobacter	-	-	-	-	-	-	-	-	-	-	-	-	-	-	-	-	-	-	-	-	-	-	-	-	-
247980	0,0%	0,0%	0,0%	0,0%	0,0%	0,1%	0,0%	0,0%	0,0%	Bacteria	Proteobacteria	Gammaproteobacteria	Xanthomonadales	Sinobacteraceae	uncultured bacterium	-	-	-	-	-	-	-	-	-	-	-	-	-	-	-	-	-	-	-	-	-	-	-	-	-
2429852	0,0%	0,0%	0,0%	0,0%	0,0%	0,0%	0,1%	0,0%	0,0%	Bacteria	Proteobacteria	Alphaproteobacteria	Caulobacterales	Caulobacteraceae	Caulobacter	-	napA	narG	nirA	-	-	nirK	norB	-	-	-	-	-	-	-	-	-	chb	-	-	laccase_Asco	laccase_Basidio	-	-	ppo
4358275	0,0%	0,0%	0,1%	0,0%	0,0%	0,0%	0,0%	0,0%	0,0%	Bacteria	Verrucomicrobia	[Spartobacteria]	[Chthoniobacterales]	[Chthoniobacteraceae]	Candidatus Xiphinematobacter	-	-	-	-	-	-	-	-	-	-	-	-	-	-	-	-	-	-	-	-	-	-	-	-	-
4019171	0,0%	0,0%	0,0%	0,0%	0,0%	0,0%	0,0%	0,0%	0,0%	Bacteria	Acidobacteria	Acidobacteria-5			uncultured Holophaga sp.	-	-	-	-	-	-	-	-	-	-	-	-	-	-	-	-	-	-	-	-	-	-	-	-	-
305406	0,0%	0,1%	0,0%	0,0%	0,0%	0,0%	0,0%	0,0%	0,0%	Bacteria	Proteobacteria	Betaproteobacteria	Burkholderiales	Comamonadaceae	uncultured	-	-	-	-	-	-	-	-	-	-	-	-	-	-	-	-	-	-	-	-	-	-	-	lip	-
209577	0,0%	0,1%	0,0%	0,0%	0,0%	0,0%	0,0%	0,0%	0,0%	Bacteria	Proteobacteria	Alphaproteobacteria	Ellin329		Candidatus Liberibacter	-	-	-	-	-	-	-	-	-	-	-	-	-	-	-	-	-	-	-	-	-	-	-	-	-
4331511	0,0%	0,0%	0,0%	0,0%	0,0%	0,1%	0,0%	0,0%	0,0%	Bacteria	Proteobacteria	Alphaproteobacteria	Rhizobiales	Hyphomicrobiaceae	Hyphomicrobium	-	napA	narG	nirA	-	-	nirK	norB	nosZ	nifH	nifD	-	ureA	-	-	-	-	-	mmoX	-	laccase_Asco	laccase_Basidio	-	-	ppo
209573	0,0%	0,0%	0,0%	0,0%	0,0%	0,0%	0,0%	0,0%	0,0%	Bacteria	Bacteroidetes	[Saprospirae]	[Saprospirales]		uncultured	-	-	-	-	-	-	-	-	-	-	-	-	-	-	-	-	-	-	-	-	-	-	-	lip	-
1939004	0,0%	0,0%	0,0%	0,0%	0,0%	0,0%	0,0%	0,0%	0,0%	Bacteria	Proteobacteria	Betaproteobacteria	Burkholderiales	Comamonadaceae	uncultured	-	-	-	-	-	-	-	-	-	-	-	-	-	-	-	-	-	-	-	-	-	-	-	lip	-
4369229	0,0%	0,0%	0,0%	0,1%	0,0%	0,0%	0,0%	0,0%	0,0%	Bacteria	Proteobacteria	Betaproteobacteria	Burkholderiales	Oxalobacteraceae	Cupriavidus	-	napA	narG	nirA	-	nirS	nirK	norB	nosZ	nifH	nifD	-	ureA	-	-	SoxB	-	-	mmoX	-	laccase_Asco	laccase_Basidio	-	-	-
4047524	0,0%	0,0%	0,0%	0,0%	0,0%	0,2%	0,0%	0,0%	0,0%	Bacteria	Planctomycetes	Phycisphaerae	WD2101		uncultured planctomycete	-	-	-	-	-	-	-	-	-	-	-	-	-	-	-	-	-	-	-	-	-	-	-	-	-
223003	0,0%	0,0%	0,0%	0,0%	0,0%	0,0%	0,0%	0,0%	0,0%	Bacteria	Proteobacteria	Deltaproteobacteria	Myxococcales		Sorangium	-	napA	-	-	-	-	nirK	norB	-	-	-	nrfA	ureA	-	-	-	-	chb	-	-	laccase_Asco	laccase_Basidio	-	-	ppo
4416974	0,0%	0,0%	0,0%	0,0%	0,0%	0,0%	0,0%	0,0%	0,0%	Bacteria	Proteobacteria	Deltaproteobacteria	Syntrophobacterales	Syntrophobacteraceae	uncultured bacterium	-	-	-	-	-	-	-	-	-	-	-	-	-	-	-	-	-	-	-	-	-	-	-	-	-
499851	0,0%	0,0%	0,0%	0,0%	0,0%	0,0%	0,0%	0,0%	0,0%	Bacteria	Proteobacteria	Gammaproteobacteria	Legionellales	Legionellaceae	Legionella	-	-	-	-	-	nirS	-	norB	-	-	-	-	-	-	-	-	-	-	-	-	laccase_Asco	laccase_Basidio	-	-	ppo
128429	0,0%	0,0%	0,0%	0,0%	0,0%	0,0%	0,0%	0,0%	0,0%	Bacteria	Proteobacteria	Gammaproteobacteria	Methylococcales	Methylococcaceae	Methylococcus	-	napA	-	nirA	-	-	-	norB	-	nifH	nifD	-	-	-	-	-	-	-	mmoX	pmoA	laccase_Asco	laccase_Basidio	-	-	ppo
518302	0,0%	0,0%	0,0%	0,0%	0,0%	0,2%	0,0%	0,0%	0,0%	Bacteria	Proteobacteria	Alphaproteobacteria	Rhodobacterales	Rhodobacteraceae	uncultured	-	-	-	-	-	-	-	-	-	-	-	-	-	-	-	-	-	-	-	-	-	-	-	lip	-
157059	0,0%	0,0%	0,0%	0,0%	0,0%	0,0%	0,0%	0,0%	0,0%	Bacteria	Proteobacteria	Alphaproteobacteria	Sphingomonadales	Sphingomonadaceae	Sandarakinorhabdus	-	-	-	-	-	-	-	-	-	-	-	-	-	-	-	-	-	-	-	-	-	-	-	-	-
4371107	0,0%	0,0%	0,0%	0,0%	0,0%	0,0%	0,0%	0,0%	0,1%	Bacteria	Actinobacteria	Thermoleophilia	Solirubrobacterales	Conexibacteraceae	Conexibacter	-	-	narG	-	-	-	-	-	-	-	-	-	ureA	-	-	-	-	-	-	-	-	laccase_Basidio	-	-	-
129337	0,1%	0,0%	0,0%	0,0%	0,0%	0,0%	0,0%	0,0%	0,0%	Bacteria	Proteobacteria	Alphaproteobacteria	Rhodospirillales	Rhodospirillaceae	Telmatospirillum	-	-	-	-	-	-	-	-	-	nifH	-	-	-	-	-	-	-	-	-	-	-	-	-	-	-
4032624	0,0%	0,0%	0,0%	0,0%	0,0%	0,0%	0,1%	0,0%	0,0%	Bacteria	Verrucomicrobia	[Pedosphaerae]	[Pedosphaerales]	auto67_4W	uncultured bacterium	-	-	-	-	-	-	-	-	-	-	-	-	-	-	-	-	-	-	-	-	-	-	-	-	-
883548	0,0%	0,0%	0,0%	0,0%	0,0%	0,0%	0,0%	0,2%	0,0%	Bacteria	Proteobacteria	Gammaproteobacteria	Xanthomonadales	Sinobacteraceae	uncultured	-	-	-	-	-	-	-	-	-	-	-	-	-	-	-	-	-	-	-	-	-	-	-	lip	-
4471067	0,0%	0,0%	0,0%	0,0%	0,0%	0,1%	0,0%	0,0%	0,0%	Bacteria	Proteobacteria	Alphaproteobacteria	Rhizobiales	Rhizobiaceae	Mesorhizobium	-	napA	narG	nirA	-	-	nirK	norB	nosZ	nifH	nifD	-	ureA	-	-	-	-	-	mmoX	-	laccase_Asco	laccase_Basidio	-	-	ppo
1855596	0,0%	0,0%	0,0%	0,0%	0,0%	0,0%	0,1%	0,0%	0,0%	Bacteria	Proteobacteria	Betaproteobacteria	Burkholderiales	Comamonadaceae	Acidovorax	-	napA	narG	nirA	-	nirS	nirK	norB	nosZ	-	-	-	ureA	-	-	-	-	-	mmoX	-	-	laccase_Basidio	-	-	ppo
4441345	0,0%	0,0%	0,0%	0,0%	0,0%	0,0%	0,0%	0,0%	0,0%	Bacteria	Proteobacteria	Betaproteobacteria	Burkholderiales	Comamonadaceae	Limnohabitans	-	napA	narG	-	-	-	-	-	-	-	-	-	ureA	-	-	-	-	-	-	-	-	-	-	-	-
154314	0,0%	0,0%	0,0%	0,0%	0,0%	0,0%	0,1%	0,0%	0,0%	Bacteria	Proteobacteria	Alphaproteobacteria	Rhodospirillales	Acetobacteraceae	uncultured	-	-	-	-	-	-	-	-	-	-	-	-	-	-	-	-	-	-	-	-	-	-	-	lip	-
3951713	0,0%	0,0%	0,0%	0,0%	0,0%	0,0%	0,0%	0,0%	0,0%	Bacteria	Proteobacteria	Betaproteobacteria	Burkholderiales	Comamonadaceae	uncultured	-	-	-	-	-	-	-	-	-	-	-	-	-	-	-	-	-	-	-	-	-	-	-	lip	-
807260	0,0%	0,0%	0,0%	0,0%	0,0%	0,0%	0,0%	0,0%	0,0%	Bacteria	Planctomycetes	Planctomycetia	Pirellulales	Pirellulaceae	Pirellula	-	napA	-	-	-	-	-	norB	-	-	-	-	-	-	-	-	-	-	-	-	-	-	-	-	-
4481131	0,0%	0,0%	0,0%	0,0%	0,0%	0,0%	0,0%	0,0%	0,1%	Bacteria	Firmicutes	Clostridia	Clostridiales	Ruminococcaceae	Faecalibacterium	cellulolytic	-	-	-	-	-	-	-	-	-	-	-	-	-	-	-	-	chb	-	-	-	-	-	-	-
4423410	0,0%	0,0%	0,0%	0,0%	0,0%	0,2%	0,0%	0,0%	0,0%	Bacteria	Proteobacteria	Alphaproteobacteria	Sphingomonadales	Sphingomonadaceae	Sphingomonas	-	napA	narG	nirA	-	-	nirK	norB	-	nifH	nifD	-	ureA	-	-	-	-	chb	mmoX	-	laccase_Asco	laccase_Basidio	-	-	ppo
1112650	0,0%	0,0%	0,1%	0,0%	0,0%	0,0%	0,0%	0,0%	0,0%	Bacteria	Proteobacteria	Alphaproteobacteria	Rhodospirillales	Rhodospirillaceae	uncultured bacterium	-	-	-	-	-	-	-	-	-	-	-	-	-	-	-	-	-	-	-	-	-	-	-	-	-
228936	0,0%	0,0%	0,1%	0,0%	0,0%	0,0%	0,0%	0,0%	0,0%	Bacteria	Proteobacteria	Alphaproteobacteria	Rhizobiales	Methylocystaceae	uncultured bacterium	-	-	-	-	-	-	-	-	-	-	-	-	-	-	-	-	-	-	-	-	-	-	-	-	-
740940	0,0%	0,0%	0,0%	0,0%	0,0%	0,1%	0,0%	0,0%	0,0%	Bacteria	Proteobacteria	Betaproteobacteria	Burkholderiales	Oxalobacteraceae	Herminiimonas	-	napA	narG	-	-	-	nirK	norB	-	-	-	-	-	-	-	SoxB	-	-	-	-	laccase_Asco	laccase_Basidio	-	-	ppo
164822	0,0%	0,0%	0,0%	0,0%	0,0%	0,0%	0,0%	0,0%	0,0%	Bacteria	Proteobacteria	Gammaproteobacteria	Aeromonadales	Aeromonadaceae	uncultured bacterium	-	-	-	-	-	-	-	-	-	-	-	-	-	-	-	-	-	-	-	-	-	-	-	-	-
512701	0,0%	0,0%	0,0%	0,0%	0,0%	0,2%	0,0%	0,0%	0,0%	Bacteria	Planctomycetes	Planctomycetia	Planctomycetales	Planctomycetaceae	Planctomyces	-	napA	-	-	-	-	-	norB	-	-	-	nrfA	-	-	-	-	-	-	-	-	-	-	-	-	-
32348	0,0%	0,0%	0,0%	0,0%	0,0%	0,0%	0,0%	0,0%	0,0%	Bacteria	Proteobacteria	Alphaproteobacteria	Rhodospirillales	Acetobacteraceae	Acidiphilium	-	napA	narG	-	-	-	-	-	-	nifH	-	-	-	-	-	SoxB	-	-	mmoX	-	laccase_Asco	laccase_Basidio	-	-	ppo
1104864	0,0%	0,0%	0,0%	0,0%	0,0%	0,0%	0,0%	0,0%	0,0%	Bacteria	Armatimonadetes	[Fimbriimonadia]	[Fimbriimonadales]	[Fimbriimonadaceae]	uncultured Armatimonadetes bacterium	-	-	-	-	-	-	-	-	-	-	-	-	-	-	-	-	-	-	-	-	-	-	-	-	-
232282	0,0%	0,0%	0,0%	0,0%	0,0%	0,0%	0,1%	0,0%	0,0%	Bacteria	Proteobacteria	Alphaproteobacteria	Rhizobiales	Rhodobiaceae	uncultured	-	-	-	-	-	-	-	-	-	-	-	-	-	-	-	-	-	-	-	-	-	-	-	lip	-
4370747	0,0%	0,0%	0,0%	0,1%	0,0%	0,0%	0,0%	0,0%	0,0%	Bacteria	Proteobacteria	Gammaproteobacteria	Pseudomonadales	Pseudomonadaceae	Pseudomonas	cellulolytic	napA	narG	nirA	-	nirS	nirK	norB	nosZ	nifH	nifD	-	ureA	-	dsrB	SoxB	-	-	mmoX	-	laccase_Asco	laccase_Basidio	-	-	ppo
4362306	0,0%	0,0%	0,0%	0,0%	0,0%	0,0%	0,1%	0,0%	0,0%	Bacteria	Proteobacteria	Alphaproteobacteria	Rhizobiales		Rhizobium	-	napA	narG	nirA	-	-	nirK	norB	nosZ	nifH	nifD	nrfA	ureA	-	-	-	-	chb	mmoX	-	laccase_Asco	laccase_Basidio	-	-	ppo
128171	0,0%	0,0%	0,1%	0,0%	0,0%	0,0%	0,0%	0,0%	0,0%	Bacteria	Proteobacteria	Betaproteobacteria	Burkholderiales	Comamonadaceae	Aquincola	-	-	-	-	-	-	-	-	-	-	-	-	-	-	-	-	-	-	-	-	-	-	-	-	-
1131789	0,0%	0,0%	0,0%	0,0%	0,0%	0,0%	0,0%	0,0%	0,1%	Bacteria	Proteobacteria	Betaproteobacteria	Burkholderiales	Comamonadaceae	Variovorax	-	napA	narG	nirA	-	-	-	norB	-	-	-	-	ureA	-	-	-	-	-	mmoX	-	-	-	-	-	-
369261	0,0%	0,0%	0,0%	0,0%	0,0%	0,0%	0,0%	0,0%	0,0%	Bacteria	Proteobacteria	Deltaproteobacteria	Desulfuromonadales	Desulfuromonadaceae	uncultured delta proteobacterium	-	-	-	-	-	-	-	-	-	-	-	-	-	-	-	-	-	-	-	-	-	-	-	-	-
831393	0,0%	0,0%	0,0%	0,0%	0,0%	0,0%	0,0%	0,0%	0,3%	Bacteria	Actinobacteria	Actinobacteria	Actinomycetales	Dermacoccaceae	Flexivirga	-	-	-	-	-	-	-	-	-	-	-	-	-	-	-	-	-	-	-	-	-	-	-	-	-
250238	0,0%	0,0%	0,0%	0,0%	0,0%	0,0%	0,0%	0,0%	0,0%	Bacteria	Cyanobacteria	Oscillatoriophycideae	Oscillatoriales	Phormidiaceae	uncultured cyanobacterium	-	-	-	-	-	-	-	-	-	-	-	-	-	-	-	-	-	-	-	-	-	-	-	-	-
821725	0,0%	0,0%	0,0%	0,0%	0,0%	0,0%	0,0%	0,0%	0,0%	Bacteria	Proteobacteria	Gammaproteobacteria	Legionellales	Coxiellaceae	Aquicella	-	-	-	-	-	-	-	-	-	-	-	-	-	-	-	-	-	-	-	-	-	-	-	-	-
773575	0,0%	0,0%	0,0%	0,0%	0,0%	0,0%	0,0%	0,0%	0,0%	Bacteria	Bacteroidetes	[Saprospirae]	[Saprospirales]	Saprospiraceae	uncultured	-	-	-	-	-	-	-	-	-	-	-	-	-	-	-	-	-	-	-	-	-	-	-	lip	-
4426207	0,0%	0,0%	0,0%	0,0%	0,0%	0,0%	0,0%	0,0%	0,0%	Bacteria	Proteobacteria	Alphaproteobacteria	Caulobacterales	Caulobacteraceae	Asticcacaulis	-	napA	narG	nirA	-	-	-	-	-	-	-	-	-	-	-	-	-	chb	-	-	laccase_Asco	laccase_Basidio	-	-	ppo
1058761	0,0%	0,0%	0,0%	0,0%	0,0%	0,0%	0,0%	0,0%	0,0%	Bacteria	Proteobacteria	Deltaproteobacteria	MIZ46		uncultured bacterium	-	-	-	-	-	-	-	-	-	-	-	-	-	-	-	-	-	-	-	-	-	-	-	-	-
4312335	0,0%	0,0%	0,0%	0,0%	0,0%	0,0%	0,0%	0,0%	0,0%	Bacteria	Proteobacteria	Betaproteobacteria	Burkholderiales	Comamonadaceae	uncultured	-	-	-	-	-	-	-	-	-	-	-	-	-	-	-	-	-	-	-	-	-	-	-	lip	-
1110065	0,0%	0,0%	0,0%	0,0%	0,0%	0,0%	0,0%	0,0%	0,1%	Bacteria	Proteobacteria	Betaproteobacteria	Burkholderiales	Comamonadaceae	uncultured	-	-	-	-	-	-	-	-	-	-	-	-	-	-	-	-	-	-	-	-	-	-	-	lip	-
4344099	0,0%	0,0%	0,0%	0,0%	0,0%	0,0%	0,0%	0,0%	0,0%	Bacteria	Planctomycetes	Planctomycetia	Gemmatales	Gemmataceae	Gemmata	-	-	-	-	-	-	-	-	-	-	-	-	-	-	-	-	-	-	-	-	-	-	-	-	-
619454	0,0%	0,0%	0,0%	0,0%	0,0%	0,0%	0,0%	0,0%	0,0%	Bacteria	Chloroflexi	Anaerolineae	envOPS12		uncultured	-	-	-	-	-	-	-	-	-	-	-	-	-	-	-	-	-	-	-	-	-	-	-	lip	-
577299	0,0%	0,0%	0,0%	0,0%	0,0%	0,0%	0,0%	0,0%	0,0%	Bacteria	Acidobacteria	DA052	Ellin6513		uncultured bacterium	-	-	-	-	-	-	-	-	-	-	-	-	-	-	-	-	-	-	-	-	-	-	-	-	-
4349834	0,0%	0,0%	0,1%	0,0%	0,0%	0,0%	0,0%	0,0%	0,0%	Bacteria	Verrucomicrobia	[Spartobacteria]	[Chthoniobacterales]	[Chthoniobacteraceae]	Candidatus Xiphinematobacter	-	-	-	-	-	-	-	-	-	-	-	-	-	-	-	-	-	-	-	-	-	-	-	-	-
99384	0,0%	0,0%	0,0%	0,0%	0,0%	0,0%	0,0%	0,0%	0,0%	Bacteria	Proteobacteria	Betaproteobacteria	Rhodocyclales	Rhodocyclaceae	Ferribacterium	-	-	-	-	-	-	-	-	-	-	-	-	-	-	-	-	-	-	-	-	-	-	-	-	-
219243	0,0%	0,0%	0,0%	0,0%	0,0%	0,0%	0,0%	0,0%	0,0%	Bacteria	Bacteroidetes	Sphingobacteriia	Sphingobacteriales		uncultured bacterium	-	-	-	-	-	-	-	-	-	-	-	-	-	-	-	-	-	-	-	-	-	-	-	-	-
1145745	0,0%	0,0%	0,0%	0,0%	0,0%	0,0%	0,0%	0,0%	0,0%	Bacteria	Acidobacteria	Acidobacteriia	Acidobacteriales	Acidobacteriaceae	Telmatobacter	cellulolytic	-	-	-	-	-	-	-	-	-	-	-	-	-	-	-	-	-	-	-	-	-	-	-	-
2685346	0,0%	0,0%	0,0%	0,0%	0,0%	0,2%	0,0%	0,0%	0,0%	Bacteria	Bacteroidetes	Sphingobacteriia	Sphingobacteriales		uncultured bacterium	-	-	-	-	-	-	-	-	-	-	-	-	-	-	-	-	-	-	-	-	-	-	-	-	-
849836	0,0%	0,0%	0,0%	0,0%	0,0%	0,0%	0,0%	0,0%	0,0%	Bacteria	Proteobacteria	Alphaproteobacteria	Rhizobiales	Hyphomicrobiaceae	Pseudolabrys	-	napA	narG	-	-	-	-	-	nosZ	-	-	-	ureA	-	-	-	-	-	-	-	-	-	-	-	-
3480259	0,0%	0,0%	0,0%	0,0%	0,0%	0,2%	0,0%	0,0%	0,0%	Bacteria	Bacteroidetes	Cytophagia	Cytophagales	Cytophagaceae	Emticicia	cellulolytic	-	-	nirA	-	-	-	-	-	-	-	-	-	-	-	-	-	chb	-	-	laccase_Asco	laccase_Basidio	-	-	ppo
3802893	0,0%	0,0%	0,0%	0,0%	0,0%	0,0%	0,0%	0,1%	0,0%	Bacteria	Proteobacteria	Betaproteobacteria	MND1		uncultured	-	-	-	-	-	-	-	-	-	-	-	-	-	-	-	-	-	-	-	-	-	-	-	lip	-
4398173	0,0%	0,0%	0,0%	0,0%	0,0%	0,1%	0,0%	0,0%	0,0%	Bacteria	Proteobacteria	Deltaproteobacteria	Myxococcales	0319-6G20	uncultured bacterium	-	-	-	-	-	-	-	-	-	-	-	-	-	-	-	-	-	-	-	-	-	-	-	-	-
4472112	0,0%	0,0%	0,0%	0,0%	0,0%	0,0%	0,0%	0,0%	0,0%	Bacteria	Proteobacteria	Betaproteobacteria	Burkholderiales	Comamonadaceae	uncultured	-	-	-	-	-	-	-	-	-	-	-	-	-	-	-	-	-	-	-	-	-	-	-	lip	-
4449013	0,0%	0,0%	0,0%	0,0%	0,0%	0,0%	0,0%	0,0%	0,0%	Bacteria	Proteobacteria	Gammaproteobacteria	Aeromonadales	Aeromonadaceae	Aeromonas	-	napA	narG	-	-	-	-	-	nosZ	nifH	-	nrfA	ureA	-	dsrB	-	-	chb	-	-	laccase_Asco	laccase_Basidio	-	-	ppo
220025	0,0%	0,0%	0,0%	0,0%	0,0%	0,1%	0,0%	0,0%	0,0%	Bacteria	Proteobacteria	Deltaproteobacteria	Myxococcales	Myxococcaceae	Anaeromyxobacter	-	napA	narG	nirA	-	nirS	-	norB	nosZ	nifH	nifD	nrfA	ureA	-	-	SoxB	-	-	-	-	-	laccase_Basidio	-	-	-
250050	0,0%	0,0%	0,1%	0,0%	0,0%	0,0%	0,0%	0,0%	0,0%	Bacteria	Proteobacteria	Deltaproteobacteria	Myxococcales		Haliangium	-	-	-	-	-	-	-	-	-	-	-	-	ureA	-	-	-	-	chb	-	-	-	-	-	-	-
574297	0,0%	0,0%	0,0%	0,0%	0,0%	0,2%	0,0%	0,0%	0,0%	Bacteria	Proteobacteria	Alphaproteobacteria	Rhodospirillales	Rhodospirillaceae	Ferrovibrio	-	-	-	-	-	-	-	-	-	-	-	-	-	-	-	-	-	-	-	-	-	-	-	-	-
891150	0,0%	0,0%	0,0%	0,0%	0,0%	0,0%	0,0%	0,0%	0,0%	Bacteria	Cyanobacteria	Oscillatoriophycideae	Chroococcales		uncultured	-	-	-	-	-	-	-	-	-	-	-	-	-	-	-	-	-	-	-	-	-	-	-	lip	-
4444046	0,0%	0,0%	0,0%	0,0%	0,0%	0,1%	0,0%	0,0%	0,0%	Bacteria	Actinobacteria	Acidimicrobiia	Acidimicrobiales		uncultured bacterium	-	-	-	-	-	-	-	-	-	-	-	-	-	-	-	-	-	-	-	-	-	-	-	-	-
1006099	0,0%	0,0%	0,0%	0,0%	0,0%	0,0%	0,0%	0,0%	0,1%	Bacteria	Bacteroidetes	Sphingobacteriia	Sphingobacteriales		uncultured bacterium	-	-	-	-	-	-	-	-	-	-	-	-	-	-	-	-	-	-	-	-	-	-	-	-	-
4311005	0,0%	0,0%	0,0%	0,0%	0,0%	0,0%	0,0%	0,0%	0,0%	Bacteria	Proteobacteria	Betaproteobacteria	Burkholderiales	Burkholderiaceae	Massilia	-	napA	narG	-	-	-	-	norB	nosZ	-	-	-	ureA	-	-	-	-	chb	mmoX	-	laccase_Asco	laccase_Basidio	-	-	ppo
270180	0,0%	0,0%	0,0%	0,1%	0,0%	0,0%	0,0%	0,0%	0,0%	Bacteria	Verrucomicrobia	[Spartobacteria]	[Chthoniobacterales]	[Chthoniobacteraceae]	Chthoniobacter	-	napA	-	-	-	-	nirK	-	-	-	-	nrfA	ureA	-	-	-	-	-	-	-	-	-	-	-	-
927367	0,0%	0,0%	0,0%	0,0%	0,0%	0,0%	0,0%	0,0%	0,0%	Bacteria	Actinobacteria	Thermoleophilia	Solirubrobacterales		Patulibacter	-	napA	narG	-	-	-	-	-	-	-	-	-	ureA	-	-	-	-	-	mmoX	-	-	-	-	-	-
160337	0,0%	0,0%	0,0%	0,0%	0,0%	0,0%	0,1%	0,0%	0,0%	Bacteria	Actinobacteria	Actinobacteria	Actinomycetales	Frankiaceae	Jatrophihabitans	-	-	-	-	-	-	-	-	-	-	-	-	-	-	-	-	-	-	-	-	-	-	-	-	-
4245392	0,0%	0,0%	0,0%	0,0%	0,0%	0,0%	0,0%	0,0%	0,0%	Bacteria	Proteobacteria	Alphaproteobacteria	Rhodobacterales	Rhodobacteraceae	Paracoccus	-	napA	narG	nirA	-	nirS	nirK	norB	nosZ	nifH	-	-	ureA	-	-	SoxB	-	-	mmoX	-	laccase_Asco	laccase_Basidio	-	-	ppo
513640	0,0%	0,0%	0,0%	0,0%	0,0%	0,0%	0,0%	0,0%	0,1%	Bacteria	Proteobacteria	Alphaproteobacteria	Rhodospirillales	Acetobacteraceae	Roseomonas	-	napA	-	-	-	-	-	-	-	-	nifD	-	ureA	-	-	-	-	-	-	-	-	laccase_Basidio	-	-	-
99774	0,0%	0,0%	0,0%	0,0%	0,0%	0,0%	0,0%	0,0%	0,0%	Bacteria	Bacteroidetes	Flavobacteriia	Flavobacteriales	Flavobacteriaceae	Flavobacterium	cellulolytic	napA	-	nirA	-	-	nirK	norB	nosZ	-	-	-	ureA	-	-	-	-	-	-	-	laccase_Asco	laccase_Basidio	-	-	ppo
4323609	0,0%	0,0%	0,1%	0,0%	0,0%	0,0%	0,0%	0,0%	0,0%	Bacteria	Proteobacteria	Betaproteobacteria	Rhodocyclales	Rhodocyclaceae	Dechloromonas	-	napA	narG	-	-	nirS	-	norB	nosZ	nifH	nifD	-	ureA	-	-	SoxB	-	-	mmoX	-	-	-	-	-	-
347524	0,0%	0,0%	0,0%	0,0%	0,0%	0,0%	0,0%	0,0%	0,0%	Bacteria	Proteobacteria	Deltaproteobacteria	Myxococcales	Polyangiaceae	Byssovorax	-	-	-	-	-	-	-	-	-	-	-	-	-	-	-	-	-	-	-	-	-	-	-	-	-
357011	0,0%	0,0%	0,0%	0,0%	0,0%	0,0%	0,0%	0,1%	0,0%	Bacteria	Proteobacteria	Alphaproteobacteria	Rhodospirillales	Rhodospirillaceae	Reyranella	-	-	-	-	-	-	-	-	-	-	-	-	-	-	-	-	-	-	-	-	-	-	-	-	-
529635	0,0%	0,0%	0,0%	0,0%	0,0%	0,0%	0,0%	0,0%	0,0%	Bacteria	Proteobacteria	Gammaproteobacteria	Methylococcales	Crenotrichaceae	Crenothrix	-	-	-	-	-	-	-	-	-	-	-	-	-	-	-	-	-	-	-	pmoA	-	-	-	-	-
780476	0,0%	0,0%	0,0%	0,0%	0,0%	0,0%	0,0%	0,0%	0,0%	Bacteria	Bacteroidetes	[Saprospirae]	[Saprospirales]	Saprospiraceae	uncultured	-	-	-	-	-	-	-	-	-	-	-	-	-	-	-	-	-	-	-	-	-	-	-	lip	-
549969	0,0%	0,0%	0,0%	0,0%	0,0%	0,0%	0,0%	0,0%	0,0%	Bacteria	Proteobacteria	Alphaproteobacteria	Rhodospirillales	Rhodospirillaceae	uncultured bacterium	-	-	-	-	-	-	-	-	-	-	-	-	-	-	-	-	-	-	-	-	-	-	-	-	-
4415093	0,0%	0,0%	0,0%	0,0%	0,0%	0,0%	0,0%	0,0%	0,1%	Bacteria	Actinobacteria	Acidimicrobiia	Acidimicrobiales	Microthrixaceae	Candidatus Microthrix	-	-	-	-	-	-	-	-	-	-	-	-	-	-	-	-	-	-	-	-	-	-	-	-	-
4422320	0,0%	0,0%	0,0%	0,0%	0,0%	0,0%	0,0%	0,0%	0,0%	Bacteria	TM6	SJA-4			uncultured bacterium	-	-	-	-	-	-	-	-	-	-	-	-	-	-	-	-	-	-	-	-	-	-	-	-	-
4422321	0,0%	0,0%	0,0%	0,0%	0,0%	0,0%	0,0%	0,0%	0,0%	Bacteria	Proteobacteria	Betaproteobacteria	Burkholderiales	Comamonadaceae	Aquincola	-	-	-	-	-	-	-	-	-	-	-	-	-	-	-	-	-	-	-	-	-	-	-	-	-
534316	0,0%	0,0%	0,0%	0,0%	0,0%	0,0%	0,0%	0,0%	0,0%	Bacteria	Proteobacteria	Alphaproteobacteria	Sphingomonadales	Sphingomonadaceae	Sphingobium	-	napA	-	nirA	-	-	-	norB	-	-	-	-	ureA	-	-	-	-	-	mmoX	-	laccase_Asco	laccase_Basidio	-	-	ppo
1026261	0,0%	0,0%	0,0%	0,0%	0,0%	0,0%	0,0%	0,0%	0,0%	Bacteria	Proteobacteria	Deltaproteobacteria	Myxococcales		Haliangium	-	-	-	-	-	-	-	-	-	-	-	-	ureA	-	-	-	-	chb	-	-	-	-	-	-	-
2881877	0,0%	0,0%	0,0%	0,0%	0,0%	0,0%	0,0%	0,0%	0,0%	Bacteria	Proteobacteria	Betaproteobacteria	Burkholderiales	Comamonadaceae	uncultured	-	-	-	-	-	-	-	-	-	-	-	-	-	-	-	-	-	-	-	-	-	-	-	lip	-

565425	0,0%	0,0%	0,0%	0,1%	0,0%	0,0%	0,0%	0,0%	0,0%	Bacteria	Bacteroidetes	Flavobacteriia	Flavobacteriales	Flavobacteriaceae	Flavobacterium	cellulolytic	napA	-	nirA	-	-	nirK	norB	nosZ	-	-	-	ureA	-	-	-	-	-	-	-	laccase_Asco	laccase_Basidio	-	-	ppo
986752	0,0%	0,0%	0,0%	0,0%	0,0%	0,0%	0,0%	0,0%	0,1%	Bacteria	Proteobacteria	Alphaproteobacteria	Rhodospirillales	Rhodospirillaceae	uncultured	-	-	-	-	-	-	-	-	-	-	-	-	-	-	-	-	-	-	-	-	-	-	-	lip	-
1139057	0,0%	0,0%	0,0%	0,0%	0,0%	0,0%	0,0%	0,0%	0,0%	Bacteria	Chloroflexi	Chloroflexi	AKIW781		uncultured bacterium	-	-	-	-	-	-	-	-	-	-	-	-	-	-	-	-	-	-	-	-	-	-	-	-	-
542933	0,0%	0,0%	0,0%	0,0%	0,0%	0,0%	0,0%	0,0%	0,0%	Bacteria	Actinobacteria	Acidimicrobiia	Acidimicrobiales	EB1017	uncultured bacterium	-	-	-	-	-	-	-	-	-	-	-	-	-	-	-	-	-	-	-	-	-	-	-	-	-
4446565	0,0%	0,0%	0,0%	0,0%	0,0%	0,2%	0,0%	0,0%	0,0%	Bacteria	Bacteroidetes	[Saprospirae]	[Saprospirales]	Chitinophagaceae	Ferruginibacter	-	-	-	-	-	-	-	-	-	-	-	-	-	-	-	-	-	-	-	-	-	-	-	-	-
586907	0,0%	0,0%	0,0%	0,0%	0,0%	0,0%	0,0%	0,0%	0,0%	Bacteria	Cyanobacteria	Oscillatoriophycideae	Oscillatoriales	Phormidiaceae	Planktothrix	-	-	-	-	-	-	-	-	-	-	-	-	-	-	-	-	-	-	-	-	-	-	-	-	-
1565317	0,0%	0,0%	0,0%	0,1%	0,0%	0,0%	0,0%	0,0%	0,0%	Bacteria	Firmicutes	Bacilli	Bacillales	Bacillaceae	Geobacillus	cellulolytic	napA	narG	-	-	-	nirK	norB	nosZ	-	-	-	ureA	-	-	-	-	-	-	-	laccase_Asco	laccase_Basidio	-	-	ppo
58374	0,0%	0,0%	0,0%	0,0%	0,0%	0,0%	0,0%	0,0%	0,0%	Bacteria	Bacteroidetes	[Saprospirae]	[Saprospirales]	Chitinophagaceae	Hydrotalea	-	-	-	-	-	-	-	-	-	-	-	-	-	-	-	-	-	-	-	-	-	-	-	-	-
1076668	0,0%	0,0%	0,0%	0,0%	0,0%	0,0%	0,0%	0,0%	0,0%	Bacteria	Firmicutes	Bacilli	Lactobacillales	Aerococcaceae	Granulicatella	-	-	-	-	-	-	-	-	-	-	-	-	-	-	-	-	-	-	-	-	-	laccase_Basidio	-	-	-
4431110	0,0%	0,0%	0,0%	0,0%	0,0%	0,0%	0,0%	0,0%	0,0%	Bacteria	Proteobacteria	Alphaproteobacteria	Rhodospirillales	Acetobacteraceae	Rhodovastum	-	-	-	-	-	-	-	-	-	-	-	-	-	-	-	-	-	-	-	-	-	-	-	-	-
4473645	0,0%	0,0%	0,0%	0,0%	0,0%	0,0%	0,0%	0,0%	0,0%	Bacteria	Proteobacteria	Betaproteobacteria	Burkholderiales	Oxalobacteraceae	Massilia	-	napA	narG	-	-	-	-	norB	nosZ	-	-	-	ureA	-	-	-	-	chb	mmoX	-	laccase_Asco	laccase_Basidio	-	-	ppo
4041568	0,1%	0,0%	0,0%	0,0%	0,0%	0,0%	0,0%	0,0%	0,0%	Bacteria	Actinobacteria	Actinobacteria	Actinomycetales	Micromonosporaceae	Actinoplanes	cellulolytic	napA	narG	-	-	-	nirK	-	-	-	-	-	ureA	-	-	-	-	-	-	-	laccase_Asco	laccase_Basidio	-	-	ppo
539570	0,0%	0,0%	0,0%	0,0%	0,0%	0,0%	0,0%	0,0%	0,0%	Bacteria	Acidobacteria	Acidobacteria-6	iii1-15	mb2424	uncultured bacterium	-	-	-	-	-	-	-	-	-	-	-	-	-	-	-	-	-	-	-	-	-	-	-	-	-
4426972	0,0%	0,0%	0,0%	0,0%	0,0%	0,0%	0,0%	0,0%	0,0%	Bacteria	Bacteroidetes	Sphingobacteriia	Sphingobacteriales	Sphingobacteriaceae	Pedobacter	-	napA	-	nirA	-	-	-	-	nosZ	-	-	-	ureA	-	-	-	-	chb	-	-	laccase_Asco	laccase_Basidio	-	-	ppo
828284	0,0%	0,0%	0,0%	0,0%	0,0%	0,0%	0,0%	0,0%	0,0%	Bacteria	Proteobacteria	Gammaproteobacteria	Aeromonadales	Aeromonadaceae	uncultured bacterium	-	-	-	-	-	-	-	-	-	-	-	-	-	-	-	-	-	-	-	-	-	-	-	-	-
4466647	0,0%	0,0%	0,0%	0,0%	0,0%	0,0%	0,0%	0,0%	0,3%	Bacteria	Proteobacteria	Betaproteobacteria	Burkholderiales	Oxalobacteraceae	Herminiimonas	-	napA	narG	-	-	-	nirK	norB	-	-	-	-	-	-	-	SoxB	-	-	-	-	laccase_Asco	laccase_Basidio	-	-	ppo
4469355	0,0%	0,0%	0,0%	0,0%	0,0%	0,0%	0,0%	0,0%	0,0%	Bacteria	Proteobacteria	Betaproteobacteria	Burkholderiales	Comamonadaceae	Ramlibacter	-	napA	-	nirA	-	-	-	-	-	-	-	-	ureA	-	-	-	-	-	-	-	laccase_Asco	laccase_Basidio	-	-	ppo
4458959	0,0%	0,0%	0,0%	0,0%	0,0%	0,0%	0,0%	0,0%	0,0%	Bacteria	Firmicutes	Clostridia	Clostridiales	Veillonellaceae	Veillonella	-	napA	narG	-	-	-	-	norB	-	-	nifD	-	-	-	-	-	-	-	-	-	laccase_Asco	laccase_Basidio	-	-	ppo
4367778	0,0%	0,0%	0,0%	0,0%	0,0%	0,0%	0,0%	0,0%	0,0%	Bacteria	Proteobacteria	Alphaproteobacteria	Rhodospirillales	Rhodospirillaceae	uncultured	-	-	-	-	-	-	-	-	-	-	-	-	-	-	-	-	-	-	-	-	-	-	-	lip	-
775023	0,0%	0,0%	0,0%	0,0%	0,0%	0,0%	0,0%	0,0%	0,0%	Bacteria	Gemmatimonadetes	Gemmatimonadetes	Gemmatimonadales	Gemmatimonadaceae	Gemmatimonas	-	napA	-	-	-	-	nirK	norB	nosZ	-	-	nrfA	-	dsrA	dsrB	-	-	chb	-	-	-	-	-	-	-
824076	0,0%	0,0%	0,0%	0,0%	0,0%	0,0%	0,0%	0,1%	0,0%	Bacteria	Proteobacteria	Gammaproteobacteria	Methylococcales	Methylococcaceae	Methylocaldum	-	-	-	-	-	-	-	-	-	nifH	-	-	-	-	-	-	-	-	mmoX	pmoA	-	-	-	-	-
4468331	0,0%	0,0%	0,0%	0,0%	0,0%	0,0%	0,0%	0,0%	0,0%	Bacteria	Verrucomicrobia	Verrucomicrobiae	Verrucomicrobiales	Verrucomicrobiaceae	Prosthecobacter	-	-	-	-	-	-	-	-	-	-	-	-	-	-	-	-	-	-	-	-	-	-	-	-	-
3931106	0,0%	0,0%	0,0%	0,0%	0,0%	0,0%	0,0%	0,0%	0,0%	Bacteria	Proteobacteria	Gammaproteobacteria	[Marinicellales]	[Marinicellaceae]	Marinicella	-	-	-	-	-	-	-	-	-	-	-	-	-	-	-	-	-	-	-	-	-	-	-	-	-
4391549	0,0%	0,0%	0,1%	0,0%	0,0%	0,0%	0,0%	0,0%	0,0%	Bacteria	Bacteroidetes	Sphingobacteriia	Sphingobacteriales	Sphingobacteriaceae	Mucilaginibacter	-	-	-	-	-	-	-	-	-	-	-	-	-	-	-	-	-	-	-	-	-	-	-	-	-
2193695	0,0%	0,0%	0,0%	0,0%	0,0%	0,0%	0,0%	0,1%	0,0%	Bacteria	Planctomycetes	Planctomycetia	Pirellulales	Pirellulaceae	uncultured	-	-	-	-	-	-	-	-	-	-	-	-	-	-	-	-	-	-	-	-	-	-	-	lip	-
2193694	0,0%	0,0%	0,0%	0,0%	0,0%	0,2%	0,0%	0,0%	0,0%	Bacteria	Proteobacteria	Betaproteobacteria	Methylophilales	Methylophilaceae	uncultured	-	-	-	-	-	-	-	-	-	-	-	-	-	-	-	-	-	-	-	-	-	-	-	lip	-
533232	0,0%	0,0%	0,0%	0,0%	0,0%	0,0%	0,0%	0,0%	0,0%	Bacteria	Proteobacteria	Betaproteobacteria	Rhodocyclales	Rhodocyclaceae	Denitratisoma	-	-	-	-	-	-	-	-	-	-	-	-	-	-	-	-	-	-	-	-	-	-	-	-	-
841943	0,0%	0,0%	0,0%	0,0%	0,0%	0,0%	0,0%	0,0%	0,0%	Bacteria	Bacteroidetes	Bacteroidia	Bacteroidales	Porphyromonadaceae	Paludibacter	-	-	-	-	-	-	-	-	-	nifH	nifD	-	-	-	-	-	-	-	-	-	-	-	-	-	-
3430519	0,0%	0,0%	0,0%	0,0%	0,0%	0,0%	0,0%	0,0%	0,0%	Bacteria	Proteobacteria	Deltaproteobacteria	Myxococcales	Myxococcaceae	Anaeromyxobacter	-	napA	narG	nirA	-	nirS	-	norB	nosZ	nifH	nifD	nrfA	ureA	-	-	SoxB	-	-	-	-	-	laccase_Basidio	-	-	-
3833747	0,0%	0,0%	0,0%	0,0%	0,0%	0,1%	0,0%	0,0%	0,0%	Bacteria	Bacteroidetes	Sphingobacteriia	Sphingobacteriales	Sphingobacteriaceae	Mucilaginibacter	-	-	-	-	-	-	-	-	-	-	-	-	-	-	-	-	-	-	-	-	-	-	-	-	-
1127531	0,0%	0,0%	0,0%	0,0%	0,0%	0,0%	0,0%	0,0%	0,0%	Bacteria	Proteobacteria	Gammaproteobacteria	Aeromonadales	Aeromonadaceae	uncultured bacterium	-	-	-	-	-	-	-	-	-	-	-	-	-	-	-	-	-	-	-	-	-	-	-	-	-
4313865	0,0%	0,0%	0,0%	0,0%	0,0%	0,0%	0,0%	0,0%	0,0%	Bacteria	Proteobacteria	Betaproteobacteria	Burkholderiales	Comamonadaceae	uncultured	-	-	-	-	-	-	-	-	-	-	-	-	-	-	-	-	-	-	-	-	-	-	-	lip	-
4377341	0,0%	0,0%	0,0%	0,0%	0,0%	0,0%	0,0%	0,0%	0,0%	Bacteria	Proteobacteria	Alphaproteobacteria	Rhodospirillales	Rhodospirillaceae	Skermanella	-	napA	-	-	-	-	-	-	-	nifH	nifD	-	ureA	-	-	-	-	-	mmoX	-	laccase_Asco	laccase_Basidio	-	-	ppo
4310019	0,0%	0,0%	0,0%	0,0%	0,0%	0,0%	0,0%	0,0%	0,0%	Bacteria	Proteobacteria	Alphaproteobacteria	Rhizobiales	Hyphomicrobiaceae	uncultured	-	-	-	-	-	-	-	-	-	-	-	-	-	-	-	-	-	-	-	-	-	-	-	lip	-
4388687	0,0%	0,0%	0,0%	0,0%	0,0%	0,0%	0,0%	0,0%	0,0%	Bacteria	Proteobacteria	Alphaproteobacteria	Rhizobiales		uncultured	-	-	-	-	-	-	-	-	-	-	-	-	-	-	-	-	-	-	-	-	-	-	-	lip	-
1118393	0,0%	0,0%	0,0%	0,0%	0,0%	0,0%	0,0%	0,0%	0,0%	Bacteria	Gemmatimonadetes	Gemm-1			uncultured	-	-	-	-	-	-	-	-	-	-	-	-	-	-	-	-	-	-	-	-	-	-	-	lip	-
546434	0,0%	0,0%	0,0%	0,0%	0,0%	0,0%	0,1%	0,0%	0,0%	Bacteria	Proteobacteria	Betaproteobacteria	Burkholderiales	Burkholderiaceae	Lautropia	-	-	narG	-	-	-	nirK	norB	nosZ	-	-	-	ureA	-	-	-	-	-	-	-	-	-	-	-	-
827300	0,0%	0,0%	0,0%	0,0%	0,0%	0,0%	0,0%	0,0%	0,0%	Bacteria	Proteobacteria	Deltaproteobacteria	Myxococcales	0319-6G20	uncultured bacterium	-	-	-	-	-	-	-	-	-	-	-	-	-	-	-	-	-	-	-	-	-	-	-	-	-
796865	0,0%	0,0%	0,0%	0,0%	0,0%	0,0%	0,0%	0,0%	0,0%	Bacteria	Proteobacteria	Betaproteobacteria	Burkholderiales	Comamonadaceae	Rhizobacter	-	napA	narG	nirA	-	-	-	norB	-	-	-	-	ureA	-	-	-	-	-	-	-	-	-	-	-	-
4366579	0,0%	0,0%	0,0%	0,0%	0,0%	0,0%	0,0%	0,0%	0,0%	Bacteria	Proteobacteria	Deltaproteobacteria	Myxococcales		uncultured bacterium	-	-	-	-	-	-	-	-	-	-	-	-	-	-	-	-	-	-	-	-	-	-	-	-	-
4294964	0,0%	0,0%	0,0%	0,0%	0,0%	0,0%	0,0%	0,0%	0,0%	Bacteria	Proteobacteria	Betaproteobacteria	Burkholderiales	Comamonadaceae	Aquincola	-	-	-	-	-	-	-	-	-	-	-	-	-	-	-	-	-	-	-	-	-	-	-	-	-
1107170	0,0%	0,0%	0,0%	0,0%	0,0%	0,0%	0,0%	0,0%	0,0%	Bacteria	Cyanobacteria	Oscillatoriophycideae	Oscillatoriales	Phormidiaceae	Planktothrix	-	-	-	-	-	-	-	-	-	-	-	-	-	-	-	-	-	-	-	-	-	-	-	-	-
2867533	0,0%	0,0%	0,0%	0,0%	0,0%	0,0%	0,0%	0,0%	0,1%	Bacteria	Verrucomicrobia	[Spartobacteria]	[Chthoniobacterales]	[Chthoniobacteraceae]	uncultured Verrucomicrobia bacterium	-	-	-	-	-	-	-	-	-	-	-	-	-	-	-	-	-	-	-	-	-	-	-	-	-
814145	0,0%	0,0%	0,0%	0,0%	0,0%	0,0%	0,0%	0,0%	0,0%	Bacteria	Proteobacteria	Alphaproteobacteria	Rhizobiales	Methylocystaceae	uncultured bacterium	-	-	-	-	-	-	-	-	-	-	-	-	-	-	-	-	-	-	-	-	-	-	-	-	-
815498	0,0%	0,0%	0,0%	0,0%	0,0%	0,0%	0,0%	0,0%	0,0%	Bacteria	Actinobacteria	Actinobacteria	Actinomycetales	Mycobacteriaceae	uncultured Mycobacteriaceae bacterium	-	-	-	-	-	-	-	-	-	-	-	-	-	-	-	-	-	-	-	-	-	-	-	-	-
1108658	0,0%	0,0%	0,0%	0,0%	0,0%	0,0%	0,0%	0,0%	0,0%	Bacteria	Actinobacteria	Actinobacteria	Actinomycetales	Frankiaceae	Jatrophihabitans	-	-	-	-	-	-	-	-	-	-	-	-	-	-	-	-	-	-	-	-	-	-	-	-	-
359891	0,0%	0,0%	0,0%	0,0%	0,0%	0,0%	0,0%	0,0%	0,0%	Bacteria	Proteobacteria	Alphaproteobacteria	Rhodospirillales	Acetobacteraceae	Roseomonas	-	napA	-	-	-	-	-	-	-	-	nifD	-	ureA	-	-	-	-	-	-	-	-	laccase_Basidio	-	-	-
4418161	0,0%	0,0%	0,0%	0,0%	0,0%	0,0%	0,0%	0,0%	0,0%	Bacteria	Proteobacteria	Deltaproteobacteria	Desulfobacterales	Desulfobacteraceae	Desulfococcus	-	napA	narG	-	-	-	-	norB	-	-	-	-	-	dsrA	dsrB	-	-	-	-	-	laccase_Asco	laccase_Basidio	-	-	ppo
1135983	0,0%	0,0%	0,0%	0,0%	0,0%	0,0%	0,0%	0,0%	0,0%	Bacteria	Acidobacteria	Acidobacteriia	Acidobacteriales	Acidobacteriaceae	Granulicella	cellulolytic	napA	-	-	-	-	-	-	-	-	-	-	-	-	-	-	-	-	-	-	laccase_Asco	laccase_Basidio	-	-	ppo
224790	0,0%	0,0%	0,0%	0,0%	0,0%	0,0%	0,0%	0,0%	0,0%	Bacteria	Planctomycetes	Planctomycetia	Pirellulales	Pirellulaceae	uncultured	-	-	-	-	-	-	-	-	-	-	-	-	-	-	-	-	-	-	-	-	-	-	-	lip	-
343608	0,0%	0,0%	0,0%	0,0%	0,0%	0,0%	0,0%	0,0%	0,0%	Bacteria	Proteobacteria	Betaproteobacteria	Burkholderiales	Comamonadaceae	uncultured	-	-	-	-	-	-	-	-	-	-	-	-	-	-	-	-	-	-	-	-	-	-	-	lip	-
92490	0,0%	0,0%	0,0%	0,0%	0,0%	0,0%	0,0%	0,0%	0,0%	Bacteria	Proteobacteria	Alphaproteobacteria	Sphingomonadales	Sphingomonadaceae	Novosphingobium	-	napA	-	-	-	-	-	norB	-	nifH	nifD	-	ureA	-	-	-	-	-	mmoX	-	laccase_Asco	laccase_Basidio	-	-	ppo
156886	0,0%	0,0%	0,0%	0,0%	0,0%	0,0%	0,0%	0,0%	0,1%	Bacteria	Proteobacteria	Alphaproteobacteria	Ellin329		Rhizomicrobium	-	-	-	-	-	-	-	-	-	-	-	-	-	-	-	-	-	-	-	-	-	-	-	-	-
4461996	0,0%	0,0%	0,0%	0,0%	0,0%	0,0%	0,0%	0,0%	0,0%	Bacteria	Proteobacteria	Alphaproteobacteria	Rhodospirillales	Rhodospirillaceae	uncultured bacterium	-	-	-	-	-	-	-	-	-	-	-	-	-	-	-	-	-	-	-	-	-	-	-	-	-
4442910	0,0%	0,0%	0,0%	0,0%	0,0%	0,0%	0,0%	0,0%	0,0%	Bacteria	Actinobacteria	Actinobacteria	Actinomycetales	Microbacteriaceae	Leucobacter	cellulolytic	-	-	-	-	-	-	-	-	-	-	-	-	-	-	-	-	-	-	-	laccase_Asco	laccase_Basidio	-	-	ppo
1100261	0,0%	0,0%	0,0%	0,0%	0,0%	0,0%	0,0%	0,0%	0,0%	Bacteria	Bacteroidetes	[Rhodothermi]	[Rhodothermales]	[Balneolaceae]	Fodinibius	-	-	-	-	-	-	-	-	-	-	-	-	-	-	-	-	-	-	-	-	-	-	-	-	-
606431	0,0%	0,0%	0,0%	0,0%	0,0%	0,0%	0,0%	0,0%	0,0%	Bacteria	Elusimicrobia	Elusimicrobia	Elusimicrobiales		uncultured bacterium	-	-	-	-	-	-	-	-	-	-	-	-	-	-	-	-	-	-	-	-	-	-	-	-	-
151682	0,0%	0,0%	0,0%	0,0%	0,0%	0,0%	0,0%	0,0%	0,0%	Bacteria	Verrucomicrobia	[Pedosphaerae]	[Pedosphaerales]	[Pedosphaeraceae]	uncultured bacterium	-	-	-	-	-	-	-	-	-	-	-	-	-	-	-	-	-	-	-	-	-	-	-	-	-
304212	0,0%	0,0%	0,0%	0,0%	0,0%	0,0%	0,0%	0,0%	0,0%	Bacteria	Proteobacteria	Alphaproteobacteria	Rhizobiales		Methylorosula	-	-	-	-	-	-	-	-	-	-	-	-	-	-	-	-	-	-	-	-	-	-	-	-	-
156401	0,0%	0,0%	0,0%	0,0%	0,0%	0,0%	0,0%	0,0%	0,0%	Bacteria	Bacteroidetes	Sphingobacteriia	Sphingobacteriales	Sphingobacteriaceae	Mucilaginibacter	-	-	-	-	-	-	-	-	-	-	-	-	-	-	-	-	-	-	-	-	-	-	-	-	-
77498	0,0%	0,0%	0,0%	0,0%	0,0%	0,0%	0,0%	0,0%	0,0%	Bacteria	Bacteroidetes	Cytophagia	Cytophagales	Cytophagaceae	uncultured	cellulolytic	-	-	-	-	-	-	-	-	-	-	-	-	-	-	-	-	-	-	-	-	-	-	lip	-
815925	0,0%	0,0%	0,0%	0,0%	0,0%	0,0%	0,0%	0,0%	0,0%	Bacteria	Proteobacteria	Gammaproteobacteria	Methylococcales	Methylococcaceae	Methylomonas	-	napA	-	nirA	-	nirS	nirK	norB	-	nifH	nifD	-	ureA	-	-	-	amoA_AOB	-	mmoX	pmoA	laccase_Asco	laccase_Basidio	-	-	ppo
219175	0,0%	0,0%	0,0%	0,0%	0,0%	0,0%	0,0%	0,0%	0,0%	Bacteria	Proteobacteria	Alphaproteobacteria	Sphingomonadales	Sphingomonadaceae	Sphingobium	-	napA	-	nirA	-	-	-	norB	-	-	-	-	ureA	-	-	-	-	-	mmoX	-	laccase_Asco	laccase_Basidio	-	-	ppo
78839	0,0%	0,0%	0,0%	0,0%	0,0%	0,0%	0,0%	0,0%	0,0%	Bacteria	Acidobacteria	DA052	Ellin6513		uncultured bacterium	-	-	-	-	-	-	-	-	-	-	-	-	-	-	-	-	-	-	-	-	-	-	-	-	-
697714	0,0%	0,0%	0,0%	0,0%	0,0%	0,0%	0,0%	0,0%	0,0%	Bacteria	Verrucomicrobia	Opitutae	Opitutales	Opitutaceae	Opitutus	-	napA	-	nirA	-	-	-	-	nosZ	-	-	nrfA	ureA	-	-	-	-	-	-	-	-	laccase_Basidio	-	-	-
1111636	0,0%	0,0%	0,0%	0,0%	0,0%	0,0%	0,0%	0,0%	0,0%	Bacteria	Proteobacteria	Betaproteobacteria	Burkholderiales	Comamonadaceae	Acidovorax	-	napA	narG	nirA	-	nirS	nirK	norB	nosZ	-	-	-	ureA	-	-	-	-	-	mmoX	-	-	laccase_Basidio	-	-	ppo
4199936	0,0%	0,0%	0,0%	0,0%	0,0%	0,0%	0,0%	0,0%	0,0%	Bacteria	Proteobacteria	Betaproteobacteria			uncultured	-	-	-	-	-	-	-	-	-	-	-	-	-	-	-	-	-	-	-	-	-	-	-	lip	-
1108275	0,0%	0,0%	0,0%	0,0%	0,0%	0,0%	0,0%	0,0%	0,0%	Bacteria	Proteobacteria	Betaproteobacteria	Burkholderiales	Comamonadaceae	Leptothrix	-	napA	-	-	-	nirS	-	norB	nosZ	nifH	nifD	-	ureA	-	-	-	-	-	mmoX	-	-	-	-	-	-
1119609	0,0%	0,0%	0,0%	0,0%	0,0%	0,0%	0,0%	0,0%	0,1%	Bacteria	Verrucomicrobia	[Spartobacteria]	[Chthoniobacterales]	[Chthoniobacteraceae]	uncultured eubacterium WCHD3-88	-	-	-	-	-	-	-	-	-	-	-	-	-	-	-	-	-	-	-	-	-	-	-	-	-
612405	0,0%	0,0%	0,0%	0,0%	0,0%	0,0%	0,0%	0,0%	0,0%	Bacteria	Acidobacteria	Acidobacteriia	Acidobacteriales	Acidobacteriaceae	Telmatobacter	cellulolytic	-	-	-	-	-	-	-	-	-	-	-	-	-	-	-	-	-	-	-	-	-	-	-	-
199919	0,0%	0,0%	0,0%	0,0%	0,0%	0,0%	0,0%	0,0%	0,0%	Bacteria	Actinobacteria	Thermoleophilia	Solirubrobacterales		uncultured Actinomycetales bacterium	-	-	-	-	-	-	-	-	-	-	-	-	-	-	-	-	-	-	-	-	-	-	-	-	-
4235729	0,0%	0,0%	0,0%	0,0%	0,0%	0,0%	0,0%	0,0%	0,0%	Bacteria	Planctomycetes	Planctomycetia	Gemmatales	Gemmataceae	uncultured	-	-	-	-	-	-	-	-	-	-	-	-	-	-	-	-	-	-	-	-	-	-	-	lip	-
558943	0,0%	0,0%	0,0%	0,0%	0,0%	0,0%	0,0%	0,0%	0,0%	Bacteria	Proteobacteria	Deltaproteobacteria	Syntrophobacterales	Syntrophaceae	Desulfomonile	-	napA	narG	-	-	-	-	norB	nosZ	nifH	nifD	-	-	dsrA	dsrB	-	-	-	-	-	-	-	-	-	-
353491	0,0%	0,0%	0,0%	0,0%	0,0%	0,0%	0,0%	0,0%	0,0%	Bacteria	Proteobacteria	Betaproteobacteria	Burkholderiales	Comamonadaceae	Aquincola	-	-	-	-	-	-	-	-	-	-	-	-	-	-	-	-	-	-	-	-	-	-	-	-	-
95883	0,0%	0,0%	0,0%	0,0%	0,0%	0,0%	0,0%	0,0%	0,0%	Bacteria	Proteobacteria	Betaproteobacteria	Burkholderiales	Alcaligenaceae	LD28 freshwater group	cellulolytic	-	-	-	-	-	-	-	-	-	-	-	-	-	-	-	-	-	-	-	-	-	-	-	-
3841245	0,0%	0,0%	0,0%	0,0%	0,0%	0,1%	0,0%	0,0%	0,1%	Bacteria	Actinobacteria	Actinobacteria	Actinomycetales	Corynebacteriaceae	Corynebacterium	-	napA	narG	-	-	-	nirK	norB	-	-	-	nrfA	ureA	-	-	-	-	-	-	-	laccase_Asco	laccase_Basidio	-	-	ppo
4385991	0,0%	0,0%	0,0%	0,0%	0,0%	0,0%	0,0%	0,0%	0,0%	Bacteria	Proteobacteria	Alphaproteobacteria	Rhizobiales		uncultured	-	-	-	-	-	-	-	-	-	-	-	-	-	-	-	-	-	-	-	-	-	-	-	lip	-
1134377	0,0%	0,0%	0,0%	0,0%	0,0%	0,1%	0,0%	0,0%	0,0%	Bacteria	Proteobacteria	Gammaproteobacteria	Enterobacteriales	Enterobacteriaceae	Erwinia	cellulolytic	napA	narG	-	-	-	-	-	-	nifH	-	-	-	-	-	-	-	chb	-	-	laccase_Asco	laccase_Basidio	-	-	ppo
4453466	0,0%	0,0%	0,0%	0,0%	0,0%	0,0%	0,0%	0,0%	0,0%	Bacteria	Proteobacteria	Alphaproteobacteria	Sphingomonadales	Sphingomonadaceae	Sphingomonas	-	napA	narG	nirA	-	-	nirK	norB	-	nifH	nifD	-	ureA	-	-	-	-	chb	mmoX	-	laccase_Asco	laccase_Basidio	-	-	ppo
977755	0,0%	0,0%	0,0%	0,0%	0,0%	0,0%	0,0%	0,1%	0,0%	Bacteria	Proteobacteria	Betaproteobacteria	Thiobacterales		uncultured	-	-	-	-	-	-	-	-	-	-	-	-	-	-	-	-	-	-	-	-	-	-	-	lip	-
616402	0,0%	0,0%	0,0%	0,0%	0,0%	0,0%	0,0%	0,1%	0,0%	Bacteria	Proteobacteria	Alphaproteobacteria	Rhizobiales	Rhizobiaceae	Rhizobium	-	napA	narG	nirA	-	-	nirK	norB	nosZ	nifH	nifD	nrfA	ureA	-	-	-	-	chb	mmoX	-	laccase_Asco	laccase_Basidio	-	-	ppo
4453710	0,0%	0,0%	0,0%	0,0%	0,0%	0,0%	0,0%	0,0%	0,0%	Bacteria	Proteobacteria	Betaproteobacteria	Burkholderiales	Comamonadaceae	Leptothrix	-	napA	-	-	-	nirS	-	norB	nosZ	nifH	nifD	-	ureA	-	-	-	-	-	mmoX	-	-	-	-	-	-
4479818	0,0%	0,0%	0,0%	0,0%	0,0%	0,0%	0,1%	0,0%	0,0%	Bacteria	Actinobacteria	Actinobacteria	Actinomycetales	Streptomycetaceae	Streptomyces	cellulolytic	napA	narG	nirA	-	-	-	norB	-	nifH	-	-	ureA	-	-	-	-	chb	mmoX	-	laccase_Asco	laccase_Basidio	-	-	ppo
593017	0,0%	0,0%	0,0%	0,0%	0,0%	0,0%	0,0%	0,0%	0,0%	Bacteria	Bacteroidetes	Sphingobacteriia	Sphingobacteriales		uncultured bacterium	-	-	-	-	-	-	-	-	-	-	-	-	-	-	-	-	-	-	-	-	-	-	-	-	-
114410	0,0%	0,0%	0,0%	0,0%	0,0%	0,0%	0,0%	0,0%	0,0%	Bacteria	Proteobacteria	Betaproteobacteria	Burkholderiales	Alcaligenaceae	uncultured	cellulolytic	-	-	-	-	-	-	-	-	-	-	-	-	-	-	-	-	-	-	-	-	-	-	lip	-
692711	0,0%	0,0%	0,0%	0,0%	0,0%	0,0%	0,0%	0,0%	0,0%	Bacteria	Proteobacteria	Deltaproteobacteria	Myxococcales		Haliangium	-	-	-	-	-	-	-	-	-	-	-	-	ureA	-	-	-	-	chb	-	-	-	-	-	-	-
1143021	0,0%	0,0%	0,0%	0,0%	0,0%	0,0%	0,0%	0,0%	0,0%	Bacteria	Proteobacteria	Betaproteobacteria	Burkholderiales	Comamonadaceae	Comamonas	-	napA	-	nirA	-	nirS	nirK	norB	nosZ	-	-	-	ureA	-	-	SoxB	-	-	mmoX	-	-	laccase_Basidio	-	-	-
4413869	0,0%	0,0%	0,0%	0,0%	0,0%	0,0%	0,0%	0,0%	0,0%	Bacteria	Proteobacteria	Betaproteobacteria	Methylophilales	Methylophilaceae	Methylotenera	-	napA	-	nirA	-	-	nirK	norB	-	-	-	-	ureA	-	-	-	-	-	-	-	-	-	-	-	-
845780	0,0%	0,0%	0,0%	0,1%	0,0%	0,0%	0,0%	0,0%	0,0%	Bacteria	Bacteroidetes	Sphingobacteriia	Sphingobacteriales	Sphingobacteriaceae	Mucilaginibacter	-	-	-	-	-	-	-	-	-	-	-	-	-	-	-	-	-	-	-	-	-	-	-	-	-
2617885	0,0%	0,0%	0,0%	0,0%	0,0%	0,0%	0,0%	0,0%	0,0%	Bacteria	Planctomycetes	Planctomycetia	Gemmatales	Gemmataceae	Gemmata	-	-	-	-	-	-	-	-	-	-	-	-	-	-	-	-	-	-	-	-	-	-	-	-	-
929489	0,0%	0,0%	0,0%	0,0%	0,0%	0,0%	0,0%	0,0%	0,0%	Bacteria	Proteobacteria	Alphaproteobacteria			uncultured bacterium	-	-	-	-	-	-	-	-	-	-	-	-	-	-	-	-	-	-	-	-	-	-	-	-	-
4471106	0,0%	0,0%	0,0%	0,0%	0,0%	0,0%	0,0%	0,0%	0,0%	Bacteria	Firmicutes	Bacilli	Bacillales	Bacillaceae	Anoxybacillus	cellulolytic	napA	narG	-	-	-	-	-	nosZ	-	-	nrfA	ureA	-	-	-	-	-	-	-	laccase_Asco	laccase_Basidio	-	-	-
5839	0,0%	0,0%	0,0%	0,0%	0,0%	0,1%	0,0%	0,0%	0,0%	Bacteria	Proteobacteria	Alphaproteobacteria	Rhodospirillales	Rhodospirillaceae	Defluviicoccus	-	-	-	-	-	-	-	-	-	-	-	-	-	-	-	-	-	-	-	-	-	-	-	-	-
4436954	0,0%	0,0%	0,0%	0,0%	0,0%	0,0%	0,0%	0,0%	0,0%	Bacteria	Proteobacteria	Alphaproteobacteria	Rhizobiales	Hyphomicrobiaceae	Devosia	-	napA	-	-	-	-	nirK	-	-	nifH	-	-	ureA	-	-	-	-	chb	-	-	laccase_Asco	laccase_Basidio	-	-	ppo
2242061	0,0%	0,0%	0,0%	0,0%	0,0%	0,0%	0,0%	0,0%	0,0%	Bacteria	Bacteroidetes	Flavobacteriia	Flavobacteriales	Flavobacteriaceae	Flavobacterium	cellulolytic	napA	-	nirA	-	-	nirK	norB	nosZ	-	-	-	ureA	-	-	-	-	-	-	-	laccase_Asco	laccase_Basidio	-	-	ppo
616088	0,0%	0,0%	0,0%	0,0%	0,0%	0,0%	0,0%	0,0%	0,0%	Bacteria	Verrucomicrobia	[Spartobacteria]	[Chthoniobacterales]	[Chthoniobacteraceae]	uncultured eubacterium WCHD3-88	-	-	-	-	-	-	-	-	-	-	-	-	-	-	-	-	-	-	-	-	-	-	-	-	-
209883	0,0%	0,0%	0,0%	0,0%	0,0%	0,0%	0,0%	0,0%	0,0%	Bacteria	Proteobacteria	Betaproteobacteria	Burkholderiales	Comamonadaceae	uncultured	-	-	-	-	-	-	-	-	-	-	-	-	-	-	-	-	-	-	-	-	-	-	-	lip	-
953084	0,0%	0,0%	0,0%	0,0%	0,0%	0,0%	0,0%	0,0%	0,0%	Bacteria	Proteobacteria	Gammaproteobacteria	Pseudomonadales	Moraxellaceae	Alkanindiges	-	-	-	-	-	-	-	-	-	-	-	-	-	-	-	-	-	-	-	-	-	-	-	-	-
500937	0,0%	0,0%	0,0%	0,0%	0,0%	0,1%	0,0%	0,0%	0,0%	Bacteria	Bacteroidetes	[Saprospirae]	[Saprospirales]	Chitinophagaceae	Ferruginibacter	-	-	-	-	-	-	-	-	-	-	-	-	-	-	-	-	-	-	-	-	-	-	-	-	-
73954	0,0%	0,0%	0,0%	0,1%	0,0%	0,0%	0,0%	0,0%	0,0%	Bacteria	Proteobacteria	Deltaproteobacteria	Desulfovibrionales	Desulfovibrionaceae	Desulfovibrio	-	napA	narG	-	-	-	nirK	norB	-	nifH	nifD	nrfA	ureA	dsrA	dsrB	-	-	-	-	-	-	-	-	-	-
4375286	0,0%	0,0%	0,0%	0,0%	0,0%	0,0%	0,0%	0,0%	0,0%	Bacteria	Proteobacteria	Gammaproteobacteria	Xanthomonadales	Sinobacteraceae	uncultured bacterium	-	-	-	-	-	-	-	-	-	-	-	-	-	-	-	-	-	-	-	-	-	-	-	-	-
4449458	0,0%	0,0%	0,0%	0,0%	0,0%	0,0%	0,0%	0,0%	0,0%	Bacteria	Proteobacteria	Gammaproteobacteria	Pseudomonadales	Moraxellaceae	Acinetobacter	-	napA	narG	nirA	-	-	-	-	-	nifH	-	-	ureA	-	-	-	-	-	mmoX	-	laccase_Asco	laccase_Basidio	-	-	ppo
1108502	0,0%	0,0%	0,0%	0,0%	0,0%	0,0%	0,0%	0,0%	0,0%	Bacteria	Proteobacteria	Betaproteobacteria	Gallionellales	Gallionellaceae	Gallionella	-	-	-	nirA	-	-	-	norB	-	-	-	-	-	-	-	-	-	-	-	-	-	-	-	-	-
240501	0,0%	0,0%	0,0%	0,0%	0,0%	0,0%	0,0%	0,0%	0,0%	Bacteria	Firmicutes	Bacilli	Bacillales	Paenibacillaceae	Paenibacillus	-	napA	narG	-	-	nirS	nirK	norB	nosZ	nifH	nifD	-	ureA	-	-	-	-	-	-	-	laccase_Asco	laccase_Basidio	-	-	ppo
177153	0,0%	0,0%	0,0%	0,0%	0,0%	0,0%	0,0%	0,0%	0,0%	Bacteria	Firmicutes	Clostridia	Clostridiales		uncultured	-	-	-	-	-	-	-	-	-	-	-	-	-	-	-	-	-	-	-	-	-	-	-	lip	-
4298047	0,0%	0,0%	0,0%	0,1%	0,0%	0,0%	0,0%	0,0%	0,0%	Bacteria	Proteobacteria	Betaproteobacteria	Burkholderiales	Oxalobacteraceae	Massilia	-	napA	narG	-	-	-	-	norB	nosZ	-	-	-	ureA	-	-	-	-	chb	mmoX	-	laccase_Asco	laccase_Basidio	-	-	ppo
239206	0,0%	0,1%	0,0%	0,0%	0,0%	0,0%	0,0%	0,0%	0,0%	Bacteria	Proteobacteria	TA18	PHOS-HD29		uncultured bacterium	-	-	-	-	-	-	-	-	-	-	-	-	-	-	-	-	-	-	-	-	-	-	-	-	-
3579424	0,0%	0,0%	0,0%	0,0%	0,0%	0,0%	0,0%	0,0%	0,0%	Bacteria	Proteobacteria	Betaproteobacteria	Burkholderiales	Comamonadaceae	uncultured	-	-	-	-	-	-	-	-	-	-	-	-	-	-	-	-	-	-	-	-	-	-	-	lip	-
12074	0,0%	0,0%	0,0%	0,0%	0,0%	0,0%	0,0%	0,0%	0,0%	Bacteria	Actinobacteria	Actinobacteria	Actinomycetales	Streptosporangiaceae	Streptosporangium	cellulolytic	napA	narG	-	-	-	-	-	-	-	-	-	ureA	-	-	-	-	-	-	-	laccase_Asco	laccase_Basidio	-	-	-
809894	0,0%	0,0%	0,0%	0,0%	0,0%	0,0%	0,0%	0,0%	0,0%	Bacteria	Bacteroidetes	Sphingobacteriia	Sphingobacteriales	Sphingobacteriaceae	Mucilaginibacter	-	-	-	-	-	-	-	-	-	-	-	-	-	-	-	-	-	-	-	-	-	-	-	-	-
1128135	0,0%	0,0%	0,0%	0,0%	0,0%	0,0%	0,0%	0,0%	0,0%	Bacteria	Proteobacteria	Betaproteobacteria	Neisseriales	Neisseriaceae	uncultured	-	-	-	-	-	-	-	-	-	-	-	-	-	-	-	-	-	-	-	-	-	-	-	lip	-
562190	0,0%	0,0%	0,0%	0,0%	0,0%	0,0%	0,0%	0,0%	0,0%	Bacteria	Acidobacteria	Solibacteres	Solibacterales	Solibacteraceae	Candidatus Solibacter	-	-	-	-	-	-	-	-	-	-	-	-	-	-	-	-	-	-	-	-	-	-	-	-	-
220256	0,0%	0,0%	0,0%	0,0%	0,0%	0,0%	0,0%	0,0%	0,0%	Bacteria	Proteobacteria	Deltaproteobacteria	Myxococcales		uncultured bacterium	-	-	-	-	-	-	-	-	-	-	-	-	-	-	-	-	-	-	-	-	-	-	-	-	-
4479347	0,0%	0,0%	0,0%	0,0%	0,0%	0,0%	0,0%	0,0%	0,0%	Bacteria	Proteobacteria	Betaproteobacteria	Burkholderiales	Comamonadaceae	Rhizobacter	-	napA	narG	nirA	-	-	-	norB	-	-	-	-	ureA	-	-	-	-	-	-	-	-	-	-	-	-
4460024	0,0%	0,0%	0,0%	0,0%	0,0%	0,0%	0,0%	0,0%	0,1%	Bacteria	Proteobacteria	Gammaproteobacteria	Pseudomonadales	Pseudomonadaceae	Pseudomonas	cellulolytic	napA	narG	nirA	-	nirS	nirK	norB	nosZ	nifH	nifD	-	ureA	-	dsrB	SoxB	-	-	mmoX	-	laccase_Asco	laccase_Basidio	-	-	ppo
4483015	0,0%	0,0%	0,0%	0,0%	0,0%	0,0%	0,0%	0,0%	0,0%	Bacteria	Firmicutes	Bacilli	Lactobacillales	Carnobacteriaceae	Granulicatella	-	-	-	-	-	-	-	-	-	-	-	-	-	-	-	-	-	-	-	-	-	laccase_Basidio	-	-	-
2591420	0,0%	0,0%	0,0%	0,0%	0,0%	0,0%	0,0%	0,0%	0,0%	Bacteria	Proteobacteria	Alphaproteobacteria	Caulobacterales	Caulobacteraceae	uncultured	-	-	-	-	-	-	-	-	-	-	-	-	-	-	-	-	-	-	-	-	-	-	-	lip	-
1141722	0,0%	0,0%	0,0%	0,0%	0,0%	0,0%	0,0%	0,0%	0,0%	Bacteria	Cyanobacteria	Synechococcophycideae	Synechococcales	Synechococcaceae	Prochlorococcus	-	napA	-	nirA	-	-	-	-	-	nifH	nifD	-	ureA	-	-	-	-	-	-	-	-	-	-	-	-
51778	0,0%	0,0%	0,0%	0,0%	0,0%	0,0%	0,0%	0,0%	0,0%	Bacteria	Planctomycetes	Planctomycetia	Gemmatales	Isosphaeraceae	Singulisphaera	-	napA	-	-	-	-	-	-	-	-	-	-	ureA	-	-	-	-	-	-	-	-	-	-	-	-
3739088	0,0%	0,0%	0,0%	0,0%	0,0%	0,0%	0,0%	0,0%	0,0%	Bacteria	Proteobacteria	Gammaproteobacteria	Legionellales	Legionellaceae	Legionella	-	-	-	-	-	nirS	-	norB	-	-	-	-	-	-	-	-	-	-	-	-	laccase_Asco	laccase_Basidio	-	-	ppo
4294646	0,0%	0,0%	0,0%	0,0%	0,0%	0,0%	0,0%	0,0%	0,0%	Bacteria	Proteobacteria	Alphaproteobacteria	Rhizobiales	Rhizobiaceae	Rhizobium	-	napA	narG	nirA	-	-	nirK	norB	nosZ	nifH	nifD	nrfA	ureA	-	-	-	-	chb	mmoX	-	laccase_Asco	laccase_Basidio	-	-	ppo
4319936	0,0%	0,0%	0,0%	0,0%	0,0%	0,0%	0,0%	0,0%	0,0%	Bacteria	Proteobacteria	Deltaproteobacteria	Myxococcales		Sorangium	-	napA	-	-	-	-	nirK	norB	-	-	-	nrfA	ureA	-	-	-	-	chb	-	-	laccase_Asco	laccase_Basidio	-	-	ppo
824417	0,0%	0,0%	0,0%	0,0%	0,0%	0,0%	0,0%	0,0%	0,1%	Bacteria	Cyanobacteria	Nostocophycideae	Nostocales	Nostocaceae	Tolypothrix sp. CAWBG76	-	-	-	-	-	-	-	-	-	-	-	-	-	-	-	-	-	-	-	-	-	-	-	-	-
186867	0,0%	0,0%	0,0%	0,0%	0,0%	0,0%	0,0%	0,0%	0,0%	Bacteria	Proteobacteria	Alphaproteobacteria	Rickettsiales	Rickettsiaceae	Rickettsia	-	-	-	-	-	-	-	-	-	-	-	-	-	-	-	-	-	-	-	-	-	-	-	-	-
4352076	0,0%	0,0%	0,0%	0,0%	0,0%	0,0%	0,0%	0,0%	0,0%	Bacteria	Cyanobacteria	Nostocophycideae	Nostocales	Nostocaceae	Nostoc	-	napA	-	nirA	-	-	-	-	-	nifH	nifD	-	ureA	-	dsrB	-	-	-	-	-	-	laccase_Basidio	-	-	ppo
4405546	0,0%	0,0%	0,0%	0,0%	0,0%	0,0%	0,0%	0,0%	0,0%	Bacteria	Proteobacteria	Betaproteobacteria	Neisseriales	Neisseriaceae	uncultured	-	-	-	-	-	-	-	-	-	-	-	-	-	-	-	-	-	-	-	-	-	-	-	lip	-
1094433	0,0%	0,0%	0,0%	0,0%	0,0%	0,0%	0,0%	0,0%	0,0%	Bacteria	Actinobacteria	Thermoleophilia	Gaiellales	Gaiellaceae	uncultured	-	-	-	-	-	-	-	-	-	-	-	-	-	-	-	-	-	-	-	-	-	-	-	lip	-
3694822	0,0%	0,0%	0,0%	0,0%	0,0%	0,0%	0,0%	0,0%	0,0%	Bacteria	Actinobacteria	Actinobacteria	Actinomycetales	Mycobacteriaceae	Mycobacterium	-	napA	narG	nirA	-	-	-	norB	-	-	-	-	ureA	-	-	-	amoA_AOB	-	mmoX	pmoA	laccase_Asco	laccase_Basidio	-	-	ppo
2554	0,0%	0,0%	0,0%	0,0%	0,0%	0,0%	0,0%	0,0%	0,0%	Bacteria	Bacteroidetes	[Saprospirae]	[Saprospirales]		uncultured	-	-	-	-	-	-	-	-	-	-	-	-	-	-	-	-	-	-	-	-	-	-	-	lip	-
586002	0,0%	0,0%	0,0%	0,0%	0,0%	0,0%	0,0%	0,0%	0,0%	Bacteria	Verrucomicrobia	[Pedosphaerae]	[Pedosphaerales]	auto67_4W	uncultured Verrucomicrobia bacterium	-	-	-	-	-	-	-	-	-	-	-	-	-	-	-	-	-	-	-	-	-	-	-	-	-
1123994	0,0%	0,0%	0,0%	0,0%	0,0%	0,0%	0,0%	0,0%	0,0%	Bacteria	Proteobacteria	Gammaproteobacteria	Chromatiales	Chromatiaceae	Thiodictyon	-	-	-	-	-	-	-	-	-	-	-	-	-	-	-	-	-	-	-	-	-	-	-	-	-
666883	0,0%	0,0%	0,0%	0,0%	0,0%	0,0%	0,0%	0,0%	0,0%	Bacteria	Proteobacteria	Deltaproteobacteria	Bdellovibrionales	Bdellovibrionaceae	Bdellovibrio	-	-	-	-	-	-	nirK	norB	-	-	-	nrfA	-	-	-	-	-	-	-	-	laccase_Asco	laccase_Basidio	-	-	ppo
4451980	0,0%	0,0%	0,0%	0,0%	0,0%	0,0%	0,0%	0,0%	0,0%	Bacteria	Proteobacteria	Alphaproteobacteria	Rhodobacterales	Rhodobacteraceae	Rhodobacter	-	napA	-	nirA	-	nirS	nirK	norB	nosZ	nifH	nifD	-	ureA	-	-	SoxB	-	-	mmoX	-	laccase_Asco	laccase_Basidio	-	-	ppo
4369121	0,0%	0,0%	0,0%	0,0%	0,0%	0,0%	0,0%	0,0%	0,0%	Bacteria	Verrucomicrobia	Verrucomicrobiae	Verrucomicrobiales	Verrucomicrobiaceae	uncultured	-	-	-	-	-	-	-	-	-	-	-	-	-	-	-	-	-	-	-	-	-	-	-	lip	-
4369122	0,0%	0,0%	0,0%	0,0%	0,0%	0,0%	0,0%	0,0%	0,0%	Bacteria	Verrucomicrobia	[Spartobacteria]	[Chthoniobacterales]	[Chthoniobacteraceae]	uncultured eubacterium WCHD3-88	-	-	-	-	-	-	-	-	-	-	-	-	-	-	-	-	-	-	-	-	-	-	-	-	-
592723	0,0%	0,0%	0,0%	0,0%	0,0%	0,0%	0,0%	0,0%	0,0%	Bacteria	Cyanobacteria	4C0d-2	MLE1-12		uncultured bacterium	-	-	-	-	-	-	-	-	-	-	-	-	-	-	-	-	-	-	-	-	-	-	-	-	-
579026	0,0%	0,0%	0,0%	0,0%	0,0%	0,0%	0,0%	0,0%	0,0%	Bacteria	Proteobacteria	Alphaproteobacteria	Rickettsiales		Candidatus Captivus	-	-	-	-	-	-	-	-	-	-	-	-	-	-	-	-	-	-	-	-	-	-	-	-	-
1522003	0,0%	0,0%	0,0%	0,0%	0,0%	0,0%	0,0%	0,0%	0,0%	Bacteria	Bacteroidetes	Sphingobacteriia	Sphingobacteriales	Sphingobacteriaceae	Mucilaginibacter	-	-	-	-	-	-	-	-	-	-	-	-	-	-	-	-	-	-	-	-	-	-	-	-	-
3259009	0,0%	0,0%	0,0%	0,0%	0,0%	0,0%	0,0%	0,0%	0,0%	Bacteria	Proteobacteria	Alphaproteobacteria	Sphingomonadales	Sphingomonadaceae	Novosphingobium	-	napA	-	-	-	-	-	norB	-	nifH	nifD	-	ureA	-	-	-	-	-	mmoX	-	laccase_Asco	laccase_Basidio	-	-	ppo
790486	0,0%	0,0%	0,0%	0,0%	0,0%	0,0%	0,0%	0,0%	0,0%	Bacteria	Proteobacteria	Alphaproteobacteria	Rhodobacterales	Rhodobacteraceae	Thalassobacter	-	napA	-	-	-	nirS	-	-	-	-	-	-	ureA	-	-	-	-	-	-	-	laccase_Asco	laccase_Basidio	-	-	ppo
4426211	0,0%	0,0%	0,0%	0,0%	0,0%	0,1%	0,0%	0,0%	0,0%	Bacteria	Proteobacteria	Gammaproteobacteria	Xanthomonadales	Sinobacteraceae	Alkanibacter	-	-	-	-	-	-	-	-	-	-	-	-	-	-	-	-	-	-	-	-	-	-	-	-	-
578882	0,0%	0,0%	0,0%	0,0%	0,0%	0,0%	0,0%	0,1%	0,0%	Bacteria	Proteobacteria	Betaproteobacteria	Burkholderiales	Comamonadaceae	Aquincola	-	-	-	-	-	-	-	-	-	-	-	-	-	-	-	-	-	-	-	-	-	-	-	-	-
128347	0,0%	0,0%	0,0%	0,0%	0,0%	0,0%	0,0%	0,0%	0,0%	Bacteria	Proteobacteria	Betaproteobacteria	Burkholderiales	Comamonadaceae	uncultured	-	-	-	-	-	-	-	-	-	-	-	-	-	-	-	-	-	-	-	-	-	-	-	lip	-
801192	0,0%	0,1%	0,0%	0,0%	0,0%	0,0%	0,0%	0,0%	0,0%	Bacteria	Proteobacteria	Betaproteobacteria	Burkholderiales	Comamonadaceae	uncultured	-	-	-	-	-	-	-	-	-	-	-	-	-	-	-	-	-	-	-	-	-	-	-	lip	-
278955	0,0%	0,0%	0,0%	0,1%	0,0%	0,0%	0,0%	0,0%	0,0%	Bacteria	Planctomycetes	Planctomycetia	Gemmatales	Gemmataceae	Gemmata	-	-	-	-	-	-	-	-	-	-	-	-	-	-	-	-	-	-	-	-	-	-	-	-	-
679997	0,0%	0,0%	0,0%	0,0%	0,0%	0,0%	0,0%	0,0%	0,0%	Bacteria	Proteobacteria	Alphaproteobacteria	Rhodospirillales	Rhodospirillaceae	uncultured alpha proteobacterium	-	-	-	-	-	-	-	-	-	-	-	-	-	-	-	-	-	-	-	-	-	-	-	-	-
550142	0,0%	0,0%	0,0%	0,0%	0,0%	0,0%	0,0%	0,0%	0,0%	Bacteria	Proteobacteria	Alphaproteobacteria	Rickettsiales		Candidatus Odyssella	-	-	-	-	-	-	-	-	-	-	-	-	-	-	-	-	-	-	-	-	-	-	-	-	-
7971	0,0%	0,0%	0,0%	0,0%	0,0%	0,0%	0,0%	0,0%	0,0%	Bacteria	Proteobacteria	Gammaproteobacteria	Legionellales	Legionellaceae	Legionella	-	-	-	-	-	nirS	-	norB	-	-	-	-	-	-	-	-	-	-	-	-	laccase_Asco	laccase_Basidio	-	-	ppo
1106545	0,0%	0,0%	0,0%	0,0%	0,0%	0,1%	0,0%	0,0%	0,1%	Bacteria	Proteobacteria	Alphaproteobacteria			uncultured gamma proteobacterium	-	-	-	-	-	-	-	-	-	-	-	-	-	-	-	-	-	-	-	-	-	-	-	-	-
345135	0,0%	0,0%	0,0%	0,0%	0,0%	0,0%	0,0%	0,0%	0,0%	Bacteria	Proteobacteria	Betaproteobacteria	Methylophilales	Methylophilaceae	Methylotenera	-	napA	-	nirA	-	-	nirK	norB	-	-	-	-	ureA	-	-	-	-	-	-	-	-	-	-	-	-
4422913	0,0%	0,0%	0,0%	0,1%	0,0%	0,0%	0,0%	0,0%	0,0%	Bacteria	Proteobacteria	Alphaproteobacteria	Rhodobacterales	Rhodobacteraceae	Paracoccus	-	napA	narG	nirA	-	nirS	nirK	norB	nosZ	nifH	-	-	ureA	-	-	SoxB	-	-	mmoX	-	laccase_Asco	laccase_Basidio	-	-	ppo
4349934	0,0%	0,0%	0,0%	0,0%	0,0%	0,0%	0,0%	0,0%	0,1%	Bacteria	Firmicutes	Bacilli	Bacillales	Staphylococcaceae	Staphylococcus	-	napA	narG	-	-	-	nirK	norB	-	-	-	-	ureA	-	-	-	-	-	-	-	laccase_Asco	laccase_Basidio	-	-	-
350938	0,0%	0,0%	0,0%	0,0%	0,0%	0,0%	0,0%	0,0%	0,0%	Bacteria	Proteobacteria	Alphaproteobacteria	Caulobacterales	Caulobacteraceae	Brevundimonas	-	-	-	nirA	-	-	-	norB	-	nifH	-	-	-	-	-	-	-	-	mmoX	-	laccase_Asco	laccase_Basidio	-	-	ppo
4482278	0,0%	0,0%	0,0%	0,0%	0,0%	0,0%	0,0%	0,0%	0,0%	Bacteria	Proteobacteria	Alphaproteobacteria	Rhodobacterales	Rhodobacteraceae	uncultured	-	-	-	-	-	-	-	-	-	-	-	-	-	-	-	-	-	-	-	-	-	-	-	lip	-
141815	0,0%	0,0%	0,0%	0,0%	0,0%	0,0%	0,0%	0,0%	0,0%	Bacteria	Armatimonadetes	Armatimonadia	Armatimonadales	Armatimonadaceae	Armatimonas	-	-	-	-	-	-	-	-	-	-	-	-	-	-	-	-	-	-	-	-	-	-	-	-	-
4336436	0,0%	0,0%	0,0%	0,0%	0,0%	0,0%	0,0%	0,0%	0,1%	Bacteria	Proteobacteria	Betaproteobacteria	Burkholderiales	Comamonadaceae	Leptothrix	-	napA	-	-	-	nirS	-	norB	nosZ	nifH	nifD	-	ureA	-	-	-	-	-	mmoX	-	-	-	-	-	-
841077	0,0%	0,0%	0,0%	0,0%	0,0%	0,1%	0,0%	0,0%	0,0%	Bacteria	Proteobacteria	Deltaproteobacteria	MIZ46		uncultured bacterium	-	-	-	-	-	-	-	-	-	-	-	-	-	-	-	-	-	-	-	-	-	-	-	-	-
574991	0,0%	0,0%	0,0%	0,0%	0,0%	0,0%	0,0%	0,1%	0,0%	Bacteria	Proteobacteria	Betaproteobacteria	Burkholderiales	Comamonadaceae	uncultured	-	-	-	-	-	-	-	-	-	-	-	-	-	-	-	-	-	-	-	-	-	-	-	lip	-
1104963	0,0%	0,0%	0,0%	0,0%	0,0%	0,0%	0,0%	0,0%	0,1%	Bacteria	Firmicutes	Clostridia	Clostridiales	Clostridiaceae	Clostridium sensu stricto 5	cellulolytic	-	-	-	-	-	-	-	-	-	-	-	-	-	-	-	-	-	-	-	-	-	-	-	-
974797	0,0%	0,0%	0,0%	0,0%	0,0%	0,0%	0,0%	0,0%	0,0%	Bacteria	Proteobacteria	Betaproteobacteria	Burkholderiales	Comamonadaceae	Aquincola	-	-	-	-	-	-	-	-	-	-	-	-	-	-	-	-	-	-	-	-	-	-	-	-	-
317329	0,0%	0,0%	0,0%	0,0%	0,0%	0,0%	0,0%	0,0%	0,0%	Bacteria	Proteobacteria	Alphaproteobacteria	BD7-3		uncultured bacterium	-	-	-	-	-	-	-	-	-	-	-	-	-	-	-	-	-	-	-	-	-	-	-	-	-
4090187	0,0%	0,0%	0,0%	0,0%	0,0%	0,1%	0,0%	0,0%	0,1%	Bacteria	Proteobacteria	Betaproteobacteria	Burkholderiales	Comamonadaceae	uncultured	-	-	-	-	-	-	-	-	-	-	-	-	-	-	-	-	-	-	-	-	-	-	-	lip	-
1111283	0,0%	0,0%	0,0%	0,0%	0,0%	0,1%	0,0%	0,0%	0,0%	Bacteria	Proteobacteria	Alphaproteobacteria			alpha proteobacterium A0839	-	-	-	-	-	-	-	-	-	-	-	-	-	-	-	-	-	-	-	-	-	-	-	-	-
177555	0,0%	0,0%	0,0%	0,0%	0,0%	0,0%	0,0%	0,0%	0,0%	Bacteria	Proteobacteria	Gammaproteobacteria	Xanthomonadales	Xanthomonadaceae	Luteibacter	cellulolytic	napA	-	-	-	-	-	-	-	-	-	-	-	-	-	-	-	-	-	-	laccase_Asco	laccase_Basidio	-	-	ppo
4431746	0,0%	0,0%	0,0%	0,0%	0,0%	0,0%	0,0%	0,0%	0,0%	Bacteria	Proteobacteria	Alphaproteobacteria	Rhizobiales	Hyphomicrobiaceae	Rhodoplanes	-	-	-	-	-	-	-	-	-	-	-	-	-	-	-	-	-	-	-	-	-	-	-	-	-
1121651	0,0%	0,0%	0,0%	0,0%	0,0%	0,1%	0,0%	0,0%	0,0%	Bacteria	Proteobacteria	Gammaproteobacteria	Aeromonadales	Aeromonadaceae	Aeromonas	-	napA	narG	-	-	-	-	-	nosZ	nifH	-	nrfA	ureA	-	dsrB	-	-	chb	-	-	laccase_Asco	laccase_Basidio	-	-	ppo
1139598	0,0%	0,0%	0,0%	0,0%	0,0%	0,0%	0,0%	0,0%	0,0%	Bacteria	Proteobacteria	Alphaproteobacteria	Sphingomonadales	Sphingomonadaceae	Sphingomonas	-	napA	narG	nirA	-	-	nirK	norB	-	nifH	nifD	-	ureA	-	-	-	-	chb	mmoX	-	laccase_Asco	laccase_Basidio	-	-	ppo

1125869	0,0%	0,0%	0,0%	0,0%	0,0%	0,0%	0,0%	0,1%	0,0%	Bacteria	Proteobacteria	Betaproteobacteria	Burkholderiales	Comamonadaceae	Leptothrix	-	napA	-	-	-	nirS	-	norB	nosZ	nifH	nifD	-	ureA	-	-	-	-	-	mmoX	-	-	-	-	-	-
60733	0,0%	0,0%	0,0%	0,0%	0,0%	0,0%	0,0%	0,0%	0,0%	Bacteria	Bacteroidetes	Flavobacteriia	Flavobacteriales	Flavobacteriaceae	Flavobacterium	cellulolytic	napA	-	nirA	-	-	nirK	norB	nosZ	-	-	-	ureA	-	-	-	-	-	-	-	laccase_Asco	laccase_Basidio	-	-	ppo
4304054	0,0%	0,0%	0,0%	0,0%	0,0%	0,0%	0,0%	0,0%	0,0%	Bacteria	Proteobacteria	Alphaproteobacteria	Rickettsiales		Candidatus Captivus	-	-	-	-	-	-	-	-	-	-	-	-	-	-	-	-	-	-	-	-	-	-	-	-	-
1129898	0,0%	0,0%	0,0%	0,0%	0,0%	0,0%	0,0%	0,0%	0,0%	Bacteria	Proteobacteria	Alphaproteobacteria	Rickettsiales	Rickettsiaceae	Wolbachia	-	-	-	-	-	-	-	-	-	-	-	-	-	-	-	-	-	-	-	-	-	-	-	-	-
4320262	0,0%	0,0%	0,0%	0,0%	0,0%	0,0%	0,0%	0,0%	0,0%	Bacteria	Proteobacteria	Deltaproteobacteria	Desulfuromonadales	Geobacteraceae	uncultured bacterium	-	-	-	-	-	-	-	-	-	-	-	-	-	-	-	-	-	-	-	-	-	-	-	-	-
832215	0,0%	0,0%	0,0%	0,0%	0,0%	0,0%	0,0%	0,0%	0,0%	Bacteria	Proteobacteria	Deltaproteobacteria	Myxococcales		Sorangium	-	napA	-	-	-	-	nirK	norB	-	-	-	nrfA	ureA	-	-	-	-	chb	-	-	laccase_Asco	laccase_Basidio	-	-	ppo
768729	0,0%	0,0%	0,0%	0,0%	0,0%	0,0%	0,0%	0,0%	0,0%	Bacteria	Verrucomicrobia	[Spartobacteria]	[Chthoniobacterales]	[Chthoniobacteraceae]	Chthoniobacter	-	napA	-	-	-	-	nirK	-	-	-	-	nrfA	ureA	-	-	-	-	-	-	-	-	-	-	-	-
105552	0,0%	0,0%	0,0%	0,0%	0,0%	0,0%	0,0%	0,0%	0,0%	Bacteria	Bacteroidetes	[Saprospirae]	[Saprospirales]	Chitinophagaceae	uncultured	-	-	-	-	-	-	-	-	-	-	-	-	-	-	-	-	-	-	-	-	-	-	-	lip	-
4463042	0,0%	0,0%	0,0%	0,0%	0,0%	0,0%	0,0%	0,0%	0,0%	Bacteria	WPS-2				uncultured bacterium	-	-	-	-	-	-	-	-	-	-	-	-	-	-	-	-	-	-	-	-	-	-	-	-	-
236303	0,0%	0,0%	0,0%	0,0%	0,0%	0,0%	0,0%	0,0%	0,0%	Bacteria	Actinobacteria	Actinobacteria	Actinomycetales		Acidothermus	-	napA	narG	-	-	-	nirK	-	-	-	-	-	-	-	-	-	-	-	-	-	-	-	-	-	-
4463047	0,0%	0,0%	0,0%	0,0%	0,0%	0,0%	0,0%	0,0%	0,0%	Bacteria	Proteobacteria	Alphaproteobacteria	Sphingomonadales	Sphingomonadaceae	Sphingomonas	-	napA	narG	nirA	-	-	nirK	norB	-	nifH	nifD	-	ureA	-	-	-	-	chb	mmoX	-	laccase_Asco	laccase_Basidio	-	-	ppo
4340696	0,0%	0,0%	0,0%	0,0%	0,0%	0,0%	0,0%	0,0%	0,0%	Bacteria	Proteobacteria	Alphaproteobacteria	Rhodospirillales	Rhodospirillaceae	uncultured	-	-	-	-	-	-	-	-	-	-	-	-	-	-	-	-	-	-	-	-	-	-	-	lip	-
564123	0,0%	0,0%	0,0%	0,0%	0,0%	0,1%	0,0%	0,0%	0,0%	Bacteria	Cyanobacteria	Synechococcophycideae	Pseudanabaenales	Pseudanabaenaceae	Pseudanabaena	-	napA	-	nirA	-	-	-	-	-	-	nifD	-	ureA	-	-	-	-	-	-	-	-	laccase_Basidio	-	-	ppo
198250	0,0%	0,0%	0,0%	0,0%	0,0%	0,1%	0,0%	0,0%	0,0%	Bacteria	Proteobacteria	Alphaproteobacteria			uncultured alpha proteobacterium	-	-	-	-	-	-	-	-	-	-	-	-	-	-	-	-	-	-	-	-	-	-	-	-	-
346204	0,0%	0,0%	0,0%	0,0%	0,0%	0,1%	0,0%	0,0%	0,0%	Bacteria	Proteobacteria	Alphaproteobacteria	Rhodospirillales	Acetobacteraceae	Roseomonas	-	napA	-	-	-	-	-	-	-	-	nifD	-	ureA	-	-	-	-	-	-	-	-	laccase_Basidio	-	-	-
707653	0,0%	0,0%	0,0%	0,0%	0,0%	0,0%	0,0%	0,0%	0,0%	Bacteria	Firmicutes	Clostridia	Clostridiales	Lachnospiraceae	Incertae Sedis	cellulolytic	-	-	-	-	-	-	-	-	-	-	-	-	-	-	-	-	-	-	-	-	-	-	-	-
4387434	0,0%	0,0%	0,0%	0,0%	0,0%	0,0%	0,0%	0,0%	0,0%	Bacteria	Proteobacteria	Alphaproteobacteria	Rhizobiales	Hyphomicrobiaceae	Pseudolabrys	-	napA	narG	-	-	-	-	-	nosZ	-	-	-	ureA	-	-	-	-	-	-	-	-	-	-	-	-
905903	0,0%	0,0%	0,0%	0,0%	0,0%	0,0%	0,0%	0,0%	0,0%	Bacteria	Proteobacteria	Betaproteobacteria	Neisseriales	Neisseriaceae	Chitinimonas	-	-	-	-	-	-	-	-	-	-	-	-	-	-	-	-	-	-	-	-	-	-	-	-	-
4396861	0,0%	0,0%	0,0%	0,0%	0,0%	0,0%	0,0%	0,0%	0,0%	Bacteria	Proteobacteria	Alphaproteobacteria	Rickettsiales		Candidatus Gortzia	-	-	-	-	-	-	-	-	-	-	-	-	-	-	-	-	-	-	-	-	-	-	-	-	-
659163	0,0%	0,0%	0,0%	0,0%	0,0%	0,0%	0,0%	0,0%	0,0%	Bacteria	Proteobacteria	Betaproteobacteria	Methylophilales	Methylophilaceae	Methylotenera	-	napA	-	nirA	-	-	nirK	norB	-	-	-	-	ureA	-	-	-	-	-	-	-	-	-	-	-	-
4465276	0,0%	0,0%	0,0%	0,0%	0,0%	0,0%	0,0%	0,0%	0,0%	Bacteria	Proteobacteria	Alphaproteobacteria	Caulobacterales	Caulobacteraceae	Asticcacaulis	-	napA	narG	nirA	-	-	-	-	-	-	-	-	-	-	-	-	-	chb	-	-	laccase_Asco	laccase_Basidio	-	-	ppo
771023	0,0%	0,0%	0,0%	0,0%	0,0%	0,0%	0,0%	0,0%	0,0%	Bacteria	Proteobacteria	Alphaproteobacteria	Rhizobiales		uncultured bacterium	-	-	-	-	-	-	-	-	-	-	-	-	-	-	-	-	-	-	-	-	-	-	-	-	-
4355314	0,0%	0,0%	0,0%	0,0%	0,0%	0,0%	0,0%	0,0%	0,0%	Bacteria	Proteobacteria	Betaproteobacteria	Burkholderiales	Comamonadaceae	uncultured	-	-	-	-	-	-	-	-	-	-	-	-	-	-	-	-	-	-	-	-	-	-	-	lip	-
1127642	0,0%	0,0%	0,0%	0,0%	0,0%	0,0%	0,0%	0,0%	0,0%	Bacteria	Acidobacteria	Solibacteres	Solibacterales	Solibacteraceae	Bryobacter	-	-	-	-	-	-	-	-	-	-	-	-	-	-	-	-	-	-	-	-	-	-	-	-	-
4390387	0,0%	0,0%	0,0%	0,0%	0,0%	0,0%	0,0%	0,0%	0,0%	Bacteria	Proteobacteria	Betaproteobacteria			uncultured	-	-	-	-	-	-	-	-	-	-	-	-	-	-	-	-	-	-	-	-	-	-	-	lip	-
4178734	0,0%	0,0%	0,0%	0,0%	0,0%	0,0%	0,0%	0,0%	0,0%	Bacteria	Proteobacteria	Alphaproteobacteria	Rhizobiales	Methylocystaceae	Pleomorphomonas	-	-	-	-	-	-	-	-	-	nifH	-	-	-	-	-	-	-	-	-	-	-	-	-	-	-
143712	0,0%	0,0%	0,0%	0,0%	0,0%	0,0%	0,0%	0,0%	0,0%	Bacteria	Proteobacteria	Betaproteobacteria	Rhodocyclales	Rhodocyclaceae	uncultured	-	-	-	-	-	-	-	-	-	-	-	-	-	-	-	-	-	-	-	-	-	-	-	lip	-
591387	0,0%	0,0%	0,0%	0,0%	0,0%	0,0%	0,0%	0,0%	0,0%	Bacteria	Armatimonadetes	[Fimbriimonadia]	[Fimbriimonadales]	[Fimbriimonadaceae]	uncultured bacterium	-	-	-	-	-	-	-	-	-	-	-	-	-	-	-	-	-	-	-	-	-	-	-	-	-
1141056	0,0%	0,0%	0,0%	0,0%	0,0%	0,0%	0,0%	0,0%	0,0%	Archaea	[Parvarchaeota]	[Parvarchaea]	YLA114		uncultured euryarchaeote	-	-	-	-	-	-	-	-	-	-	-	-	-	-	-	-	-	-	-	-	-	-	-	-	-
13047	0,0%	0,0%	0,0%	0,0%	0,0%	0,0%	0,0%	0,0%	0,0%	Bacteria	Actinobacteria	Actinobacteria	Actinomycetales	Mycobacteriaceae	Mycobacterium	-	napA	narG	nirA	-	-	-	norB	-	-	-	-	ureA	-	-	-	amoA_AOB	-	mmoX	pmoA	laccase_Asco	laccase_Basidio	-	-	ppo
4469253	0,0%	0,0%	0,0%	0,0%	0,0%	0,0%	0,0%	0,0%	0,0%	Bacteria	Proteobacteria	Alphaproteobacteria	Rhizobiales	Rhizobiaceae	Rhizobium	-	napA	narG	nirA	-	-	nirK	norB	nosZ	nifH	nifD	nrfA	ureA	-	-	-	-	chb	mmoX	-	laccase_Asco	laccase_Basidio	-	-	ppo
4468329	0,0%	0,0%	0,0%	0,0%	0,0%	0,0%	0,0%	0,0%	0,0%	Bacteria	Proteobacteria	Alphaproteobacteria	Rhodobacterales	Hyphomonadaceae	uncultured bacterium	-	-	-	-	-	-	-	-	-	-	-	-	-	-	-	-	-	-	-	-	-	-	-	-	-
4452613	0,0%	0,0%	0,0%	0,0%	0,0%	0,0%	0,0%	0,0%	0,0%	Bacteria	Proteobacteria	Gammaproteobacteria	Enterobacteriales	Enterobacteriaceae	Enterobacter	cellulolytic	napA	narG	-	-	-	-	-	-	nifH	nifD	nrfA	ureA	-	-	-	-	chb	-	-	laccase_Asco	laccase_Basidio	-	-	ppo
583421	0,0%	0,0%	0,0%	0,0%	0,0%	0,0%	0,0%	0,0%	0,0%	Bacteria	Verrucomicrobia	[Pedosphaerae]	[Pedosphaerales]	Ellin517	uncultured soil bacterium	-	-	-	-	-	-	-	-	-	-	-	-	-	-	-	-	-	-	-	-	-	-	-	-	-
4483490	0,0%	0,0%	0,0%	0,0%	0,0%	0,0%	0,0%	0,0%	0,0%	Bacteria	Proteobacteria	Betaproteobacteria	Burkholderiales	Comamonadaceae	Aquincola	-	-	-	-	-	-	-	-	-	-	-	-	-	-	-	-	-	-	-	-	-	-	-	-	-
156963	0,0%	0,0%	0,0%	0,0%	0,0%	0,1%	0,0%	0,0%	0,0%	Bacteria	Bacteroidetes	Cytophagia	Cytophagales	Cytophagaceae	uncultured	cellulolytic	-	-	-	-	-	-	-	-	-	-	-	-	-	-	-	-	-	-	-	-	-	-	lip	-
672144	0,0%	0,0%	0,0%	0,0%	0,0%	0,0%	0,0%	0,1%	0,3%	Bacteria	Proteobacteria	Betaproteobacteria	Burkholderiales	Comamonadaceae	Rhizobacter	-	napA	narG	nirA	-	-	-	norB	-	-	-	-	ureA	-	-	-	-	-	-	-	-	-	-	-	-
1060474	0,0%	0,0%	0,0%	0,0%	0,0%	0,0%	0,0%	0,1%	0,0%	Bacteria	Actinobacteria	Actinobacteria	Actinomycetales	Kineosporiaceae	Kineosporia	-	-	-	-	-	-	-	-	-	-	-	-	-	-	-	-	-	-	-	-	-	-	-	-	-
4298844	0,0%	0,0%	0,0%	0,0%	0,0%	0,0%	0,0%	0,0%	0,0%	Bacteria	Proteobacteria	Betaproteobacteria	Procabacteriales	Procabacteriaceae	uncultured	-	-	-	-	-	-	-	-	-	-	-	-	-	-	-	-	-	-	-	-	-	-	-	lip	-
4368521	0,0%	0,0%	0,0%	0,0%	0,0%	0,0%	0,0%	0,0%	0,0%	Bacteria	Proteobacteria	Gammaproteobacteria	Chromatiales		Acidiferrobacter	-	-	-	-	-	-	-	-	-	nifH	-	-	-	-	-	-	-	-	-	-	-	-	-	-	-
1142110	0,0%	0,0%	0,0%	0,0%	0,0%	0,0%	0,0%	0,0%	0,0%	Bacteria	Firmicutes	Clostridia	Clostridiales	Peptostreptococcaceae	Incertae Sedis	-	-	-	-	-	-	-	-	-	-	-	-	-	-	-	-	-	-	-	-	-	-	-	-	-
4397941	0,0%	0,0%	0,0%	0,0%	0,0%	0,0%	0,0%	0,0%	0,0%	Bacteria	Acidobacteria	Solibacteres	Solibacterales	Solibacteraceae	Candidatus Solibacter	-	-	-	-	-	-	-	-	-	-	-	-	-	-	-	-	-	-	-	-	-	-	-	-	-
1889810	0,0%	0,0%	0,0%	0,0%	0,0%	0,0%	0,0%	0,0%	0,0%	Bacteria	Bacteroidetes	Flavobacteriia	Flavobacteriales	Flavobacteriaceae	Flavobacterium	cellulolytic	napA	-	nirA	-	-	nirK	norB	nosZ	-	-	-	ureA	-	-	-	-	-	-	-	laccase_Asco	laccase_Basidio	-	-	ppo
4468805	0,0%	0,0%	0,0%	0,0%	0,0%	0,0%	0,0%	0,0%	0,0%	Bacteria	Firmicutes	Bacilli	Lactobacillales	Streptococcaceae	Lactococcus	-	-	-	-	-	-	-	-	-	-	-	-	-	-	-	-	-	-	-	-	laccase_Asco	laccase_Basidio	-	-	ppo
1115291	0,0%	0,0%	0,0%	0,0%	0,0%	0,0%	0,0%	0,0%	0,0%	Bacteria	Gemmatimonadetes	Gemmatimonadetes	KD8-87		uncultured	-	-	-	-	-	-	-	-	-	-	-	-	-	-	-	-	-	-	-	-	-	-	-	lip	-
147541	0,0%	0,0%	0,0%	0,0%	0,0%	0,0%	0,0%	0,0%	0,0%	Bacteria	Proteobacteria	Alphaproteobacteria	Rhizobiales	Bradyrhizobiaceae	Afipia	-	napA	narG	-	-	-	nirK	-	nosZ	nifH	-	-	ureA	-	-	-	-	-	-	-	laccase_Asco	laccase_Basidio	-	-	ppo
333731	0,0%	0,0%	0,0%	0,0%	0,0%	0,0%	0,0%	0,0%	0,0%	Bacteria	Actinobacteria	Thermoleophilia	Solirubrobacterales		uncultured bacterium	-	-	-	-	-	-	-	-	-	-	-	-	-	-	-	-	-	-	-	-	-	-	-	-	-
823091	0,0%	0,0%	0,0%	0,0%	0,0%	0,0%	0,0%	0,0%	0,0%	Bacteria	Gemmatimonadetes	Gemm-1			uncultured	-	-	-	-	-	-	-	-	-	-	-	-	-	-	-	-	-	-	-	-	-	-	-	lip	-
4299406	0,0%	0,0%	0,0%	0,0%	0,0%	0,0%	0,0%	0,0%	0,0%	Bacteria	Proteobacteria	Betaproteobacteria	Burkholderiales	Oxalobacteraceae	Undibacterium	-	-	-	-	-	-	-	-	-	-	-	-	-	-	-	-	-	-	-	-	-	-	-	-	-
8079	0,0%	0,0%	0,0%	0,0%	0,0%	0,0%	0,0%	0,0%	0,0%	Bacteria	Proteobacteria	Gammaproteobacteria	Methylococcales	Methylococcaceae	Methylomonas	-	napA	-	nirA	-	nirS	nirK	norB	-	nifH	nifD	-	ureA	-	-	-	amoA_AOB	-	mmoX	pmoA	laccase_Asco	laccase_Basidio	-	-	ppo
217392	0,0%	0,0%	0,0%	0,0%	0,0%	0,0%	0,0%	0,0%	0,0%	Bacteria	Actinobacteria	Thermoleophilia	Solirubrobacterales	Conexibacteraceae	uncultured bacterium	-	-	-	-	-	-	-	-	-	-	-	-	-	-	-	-	-	-	-	-	-	-	-	-	-
4306763	0,0%	0,0%	0,0%	0,0%	0,0%	0,0%	0,0%	0,0%	0,0%	Bacteria	Proteobacteria	Betaproteobacteria	Burkholderiales	Comamonadaceae	Aquincola	-	-	-	-	-	-	-	-	-	-	-	-	-	-	-	-	-	-	-	-	-	-	-	-	-
1122011	0,0%	0,0%	0,0%	0,0%	0,0%	0,0%	0,0%	0,0%	0,0%	Bacteria	Proteobacteria	Betaproteobacteria	Burkholderiales	Oxalobacteraceae	uncultured	-	-	-	-	-	-	-	-	-	-	-	-	-	-	-	-	-	-	-	-	-	-	-	lip	-
7912	0,0%	0,0%	0,0%	0,0%	0,0%	0,0%	0,0%	0,0%	0,0%	Bacteria	Proteobacteria	Gammaproteobacteria	Legionellales	Legionellaceae	Legionella	-	-	-	-	-	nirS	-	norB	-	-	-	-	-	-	-	-	-	-	-	-	laccase_Asco	laccase_Basidio	-	-	ppo
782646	0,0%	0,0%	0,0%	0,0%	0,0%	0,1%	0,0%	0,0%	0,0%	Bacteria	Proteobacteria	Betaproteobacteria	Rhodocyclales	Rhodocyclaceae	uncultured	-	-	-	-	-	-	-	-	-	-	-	-	-	-	-	-	-	-	-	-	-	-	-	lip	-
4483172	0,0%	0,0%	0,0%	0,0%	0,0%	0,0%	0,0%	0,0%	0,0%	Bacteria	Proteobacteria	Deltaproteobacteria	Desulfuromonadales	Geobacteraceae	Pseudomonas	-	napA	narG	nirA	-	nirS	nirK	norB	nosZ	nifH	nifD	-	ureA	-	dsrB	SoxB	-	-	mmoX	-	laccase_Asco	laccase_Basidio	-	-	ppo
4319587	0,0%	0,0%	0,0%	0,0%	0,0%	0,0%	0,0%	0,0%	0,0%	Bacteria	Proteobacteria	Alphaproteobacteria	Sphingomonadales		Novosphingobium	-	napA	-	-	-	-	-	norB	-	nifH	nifD	-	ureA	-	-	-	-	-	mmoX	-	laccase_Asco	laccase_Basidio	-	-	ppo
2251909	0,0%	0,0%	0,0%	0,0%	0,0%	0,0%	0,0%	0,0%	0,0%	Bacteria	Firmicutes	Clostridia	Clostridiales	Peptostreptococcaceae	Incertae Sedis	-	-	-	-	-	-	-	-	-	-	-	-	-	-	-	-	-	-	-	-	-	-	-	-	-
136998	0,0%	0,0%	0,0%	0,0%	0,0%	0,0%	0,0%	0,0%	0,0%	Bacteria	Verrucomicrobia	Opitutae	Opitutales	Opitutaceae	Opitutus	-	napA	-	nirA	-	-	-	-	nosZ	-	-	nrfA	ureA	-	-	-	-	-	-	-	-	laccase_Basidio	-	-	-
2296710	0,0%	0,0%	0,0%	0,0%	0,0%	0,0%	0,0%	0,0%	0,0%	Bacteria	Proteobacteria	Alphaproteobacteria	Rhizobiales	Hyphomicrobiaceae	uncultured	-	-	-	-	-	-	-	-	-	-	-	-	-	-	-	-	-	-	-	-	-	-	-	lip	-
518990	0,0%	0,0%	0,0%	0,0%	0,0%	0,0%	0,0%	0,0%	0,0%	Bacteria	TM6	SJA-4			uncultured bacterium	-	-	-	-	-	-	-	-	-	-	-	-	-	-	-	-	-	-	-	-	-	-	-	-	-
1052235	0,0%	0,0%	0,0%	0,0%	0,0%	0,0%	0,0%	0,0%	0,0%	Bacteria	Proteobacteria	Deltaproteobacteria	Myxococcales	Haliangiaceae	Haliangium	-	-	-	-	-	-	-	-	-	-	-	-	ureA	-	-	-	-	chb	-	-	-	-	-	-	-
267819	0,0%	0,0%	0,0%	0,0%	0,0%	0,0%	0,0%	0,0%	0,0%	Bacteria	Planctomycetes	Planctomycetia	Planctomycetales	Planctomycetaceae	Planctomyces	-	napA	-	-	-	-	-	norB	-	-	-	nrfA	-	-	-	-	-	-	-	-	-	-	-	-	-
78776	0,0%	0,0%	0,0%	0,0%	0,0%	0,0%	0,0%	0,0%	0,0%	Bacteria	Proteobacteria	Alphaproteobacteria			uncultured alpha proteobacterium	-	-	-	-	-	-	-	-	-	-	-	-	-	-	-	-	-	-	-	-	-	-	-	-	-
529264	0,0%	0,0%	0,0%	0,1%	0,0%	0,0%	0,0%	0,0%	0,0%	Bacteria	Planctomycetes	Planctomycetia	Pirellulales	Pirellulaceae	Pirellula	-	napA	-	-	-	-	-	norB	-	-	-	-	-	-	-	-	-	-	-	-	-	-	-	-	-
591575	0,0%	0,0%	0,0%	0,0%	0,0%	0,0%	0,0%	0,1%	0,0%	Bacteria	FCPU426				uncultured bacterium	-	-	-	-	-	-	-	-	-	-	-	-	-	-	-	-	-	-	-	-	-	-	-	-	-
993307	0,0%	0,0%	0,0%	0,0%	0,0%	0,0%	0,0%	0,1%	0,0%	Bacteria	Planctomycetes	Planctomycetia	Pirellulales	Pirellulaceae	Pirellula	-	napA	-	-	-	-	-	norB	-	-	-	-	-	-	-	-	-	-	-	-	-	-	-	-	-
681051	0,0%	0,0%	0,0%	0,0%	0,0%	0,0%	0,0%	0,0%	0,0%	Bacteria	Proteobacteria	Alphaproteobacteria	Rhizobiales	Hyphomicrobiaceae	Hyphomicrobium	-	napA	narG	nirA	-	-	nirK	norB	nosZ	nifH	nifD	-	ureA	-	-	-	-	-	mmoX	-	laccase_Asco	laccase_Basidio	-	-	ppo
1116348	0,0%	0,0%	0,0%	0,0%	0,0%	0,0%	0,0%	0,0%	0,0%	Bacteria	Proteobacteria	Betaproteobacteria	Burkholderiales	Comamonadaceae	Chlorochromatium	-	-	-	-	-	-	-	-	-	-	-	-	-	-	-	-	-	-	-	-	-	-	-	-	-
1147699	0,0%	0,0%	0,0%	0,1%	0,0%	0,0%	0,0%	0,0%	0,0%	Bacteria	Acidobacteria	DA052	Ellin6513		uncultured bacterium	-	-	-	-	-	-	-	-	-	-	-	-	-	-	-	-	-	-	-	-	-	-	-	-	-
4357615	0,0%	0,0%	0,0%	0,0%	0,0%	0,0%	0,0%	0,0%	0,0%	Bacteria	Bacteroidetes	Sphingobacteriia	Sphingobacteriales	Sphingobacteriaceae	Mucilaginibacter	-	-	-	-	-	-	-	-	-	-	-	-	-	-	-	-	-	-	-	-	-	-	-	-	-
1016118	0,0%	0,0%	0,0%	0,0%	0,0%	0,0%	0,0%	0,0%	0,0%	Bacteria	Proteobacteria	Deltaproteobacteria	Myxococcales		Haliangium	-	-	-	-	-	-	-	-	-	-	-	-	ureA	-	-	-	-	chb	-	-	-	-	-	-	-
74063	0,0%	0,0%	0,0%	0,0%	0,0%	0,0%	0,0%	0,0%	0,0%	Bacteria	Proteobacteria	Alphaproteobacteria	Rhodospirillales	Rhodospirillaceae	uncultured	-	-	-	-	-	-	-	-	-	-	-	-	-	-	-	-	-	-	-	-	-	-	-	lip	-
1125638	0,0%	0,0%	0,0%	0,0%	0,0%	0,0%	0,0%	0,0%	0,0%	Bacteria	Proteobacteria	Gammaproteobacteria	Xanthomonadales	Sinobacteraceae	uncultured bacterium	-	-	-	-	-	-	-	-	-	-	-	-	-	-	-	-	-	-	-	-	-	-	-	-	-
533038	0,0%	0,0%	0,0%	0,0%	0,0%	0,0%	0,0%	0,0%	0,0%	Bacteria	Proteobacteria	Betaproteobacteria	Methylophilales	Methylophilaceae	LD28 freshwater group	-	-	-	-	-	-	-	-	-	-	-	-	-	-	-	-	-	-	-	-	-	-	-	-	-
4413347	0,0%	0,0%	0,0%	0,0%	0,0%	0,0%	0,0%	0,1%	0,0%	Bacteria	Actinobacteria	Actinobacteria	Bifidobacteriales	Bifidobacteriaceae	Bifidobacterium	-	-	-	-	-	-	-	-	-	-	-	-	ureA	-	-	-	-	-	-	-	-	-	-	-	-
311991	0,0%	0,0%	0,0%	0,0%	0,0%	0,0%	0,0%	0,0%	0,0%	Bacteria	Chloroflexi	Ktedonobacteria	Thermogemmatisporales	Thermogemmatisporaceae	uncultured bacterium	-	-	-	-	-	-	-	-	-	-	-	-	-	-	-	-	-	-	-	-	-	-	-	-	-
1029858	0,0%	0,0%	0,0%	0,0%	0,0%	0,0%	0,0%	0,0%	0,0%	Bacteria	Planctomycetes	Planctomycetia	Gemmatales	Gemmataceae	Gemmata	-	-	-	-	-	-	-	-	-	-	-	-	-	-	-	-	-	-	-	-	-	-	-	-	-
257129	0,0%	0,0%	0,0%	0,0%	0,0%	0,0%	0,0%	0,0%	0,0%	Bacteria	Proteobacteria	Gammaproteobacteria	Legionellales		Beggiatoa	-	napA	narG	-	nxrB	nirS	-	norB	-	-	nifD	-	ureA	-	-	-	-	-	mmoX	-	-	-	-	-	ppo
4369692	0,0%	0,0%	0,0%	0,0%	0,0%	0,0%	0,0%	0,0%	0,0%	Bacteria	Bacteroidetes	Sphingobacteriia	Sphingobacteriales	Sphingobacteriaceae	Mucilaginibacter	-	-	-	-	-	-	-	-	-	-	-	-	-	-	-	-	-	-	-	-	-	-	-	-	-
586558	0,0%	0,0%	0,0%	0,0%	0,0%	0,0%	0,0%	0,0%	0,0%	Bacteria	Actinobacteria	Actinobacteria	Actinomycetales		uncultured bacterium	-	-	-	-	-	-	-	-	-	-	-	-	-	-	-	-	-	-	-	-	-	-	-	-	-
219534	0,0%	0,0%	0,0%	0,0%	0,0%	0,0%	0,0%	0,0%	0,0%	Bacteria	Bacteroidetes	[Saprospirae]	[Saprospirales]	Chitinophagaceae	uncultured	-	-	-	-	-	-	-	-	-	-	-	-	-	-	-	-	-	-	-	-	-	-	-	lip	-
4407152	0,0%	0,0%	0,0%	0,0%	0,0%	0,0%	0,0%	0,0%	0,0%	Bacteria	Planctomycetes	Planctomycetia	Planctomycetales	Planctomycetaceae	Planctomyces	-	napA	-	-	-	-	-	norB	-	-	-	nrfA	-	-	-	-	-	-	-	-	-	-	-	-	-
833489	0,0%	0,0%	0,0%	0,0%	0,0%	0,0%	0,0%	0,0%	0,0%	Bacteria	Planctomycetes	Planctomycetia	Gemmatales	Gemmataceae	Gemmata	-	-	-	-	-	-	-	-	-	-	-	-	-	-	-	-	-	-	-	-	-	-	-	-	-
276586	0,0%	0,0%	0,0%	0,0%	0,0%	0,0%	0,0%	0,0%	0,1%	Bacteria	Elusimicrobia	Elusimicrobia	IIb		uncultured soil bacterium	-	-	-	-	-	-	-	-	-	-	-	-	-	-	-	-	-	-	-	-	-	-	-	-	-
2457622	0,0%	0,0%	0,0%	0,0%	0,0%	0,0%	0,0%	0,0%	0,0%	Bacteria	Proteobacteria	Betaproteobacteria	Rhodocyclales	Rhodocyclaceae	Azospira	-	-	narG	-	-	-	nirK	-	-	nifH	-	-	-	-	-	-	-	-	-	-	-	-	-	-	-
4479288	0,0%	0,0%	0,0%	0,0%	0,0%	0,0%	0,0%	0,0%	0,0%	Bacteria	Proteobacteria	Alphaproteobacteria	Rhizobiales	Rhizobiaceae	Rhizobium	-	napA	narG	nirA	-	-	nirK	norB	nosZ	nifH	nifD	nrfA	ureA	-	-	-	-	chb	mmoX	-	laccase_Asco	laccase_Basidio	-	-	ppo
361234	0,0%	0,0%	0,0%	0,0%	0,0%	0,0%	0,0%	0,0%	0,0%	Bacteria	Acidobacteria	Holophagae	Holophagales	Holophagaceae	Geothrix	-	-	-	-	-	-	-	-	-	-	-	-	-	-	-	-	-	-	-	-	-	-	-	-	-
812652	0,0%	0,0%	0,0%	0,1%	0,0%	0,0%	0,0%	0,0%	0,0%	Bacteria	Proteobacteria	Alphaproteobacteria	Rhodospirillales	Acetobacteraceae	Acidiphilium	-	napA	narG	-	-	-	-	-	-	nifH	-	-	-	-	-	SoxB	-	-	mmoX	-	laccase_Asco	laccase_Basidio	-	-	ppo
566996	0,0%	0,0%	0,0%	0,0%	0,0%	0,0%	0,0%	0,0%	0,0%	Bacteria	Acidobacteria	Acidobacteriia	Acidobacteriales	Acidobacteriaceae	Granulicella	cellulolytic	napA	-	-	-	-	-	-	-	-	-	-	-	-	-	-	-	-	-	-	laccase_Asco	laccase_Basidio	-	-	ppo
227534	0,0%	0,0%	0,0%	0,0%	0,0%	0,0%	0,0%	0,0%	0,0%	Bacteria	Planctomycetes	Planctomycetia	Gemmatales	Isosphaeraceae	Singulisphaera	-	napA	-	-	-	-	-	-	-	-	-	-	ureA	-	-	-	-	-	-	-	-	-	-	-	-
289730	0,0%	0,0%	0,0%	0,0%	0,0%	0,0%	0,0%	0,0%	0,0%	Bacteria	Firmicutes	Clostridia	Clostridiales	Peptostreptococcaceae	Incertae Sedis	-	-	-	-	-	-	-	-	-	-	-	-	-	-	-	-	-	-	-	-	-	-	-	-	-
8097	0,0%	0,0%	0,0%	0,0%	0,0%	0,0%	0,0%	0,0%	0,0%	Bacteria	Proteobacteria	Gammaproteobacteria	Methylococcales	Crenotrichaceae	Crenothrix	-	-	-	-	-	-	-	-	-	-	-	-	-	-	-	-	-	-	-	pmoA	-	-	-	-	-
511068	0,0%	0,0%	0,0%	0,0%	0,0%	0,0%	0,0%	0,0%	0,0%	Bacteria	Proteobacteria	Betaproteobacteria	SBla14		Sideroxydans	-	napA	-	-	-	nirS	-	norB	-	nifH	nifD	-	-	dsrA	dsrB	-	-	-	-	-	-	-	-	-	-
132722	0,0%	0,0%	0,0%	0,0%	0,0%	0,0%	0,0%	0,0%	0,0%	Bacteria	Verrucomicrobia	[Pedosphaerae]	[Pedosphaerales]		uncultured bacterium	-	-	-	-	-	-	-	-	-	-	-	-	-	-	-	-	-	-	-	-	-	-	-	-	-
4145	0,0%	0,0%	0,0%	0,0%	0,0%	0,0%	0,0%	0,0%	0,0%	Bacteria	Spirochaetes	Spirochaetes	Spirochaetales	Spirochaetaceae	Spirochaeta	-	-	-	nirA	-	-	-	-	-	nifH	nifD	-	-	-	-	-	-	-	-	-	-	-	-	-	-
561727	0,0%	0,0%	0,0%	0,0%	0,0%	0,0%	0,0%	0,0%	0,0%	Bacteria	Planctomycetes	Planctomycetia	Gemmatales	Isosphaeraceae	Isosphaera	-	napA	-	-	-	-	-	-	-	-	-	-	-	-	-	-	-	-	-	-	-	-	-	-	-
564004	0,0%	0,0%	0,0%	0,0%	0,0%	0,0%	0,0%	0,0%	0,0%	Bacteria	Proteobacteria	Alphaproteobacteria	Rhodospirillales	Rhodospirillaceae	Reyranella	-	-	-	-	-	-	-	-	-	-	-	-	-	-	-	-	-	-	-	-	-	-	-	-	-
974238	0,0%	0,0%	0,0%	0,0%	0,0%	0,0%	0,0%	0,0%	0,0%	Bacteria	Proteobacteria	Deltaproteobacteria	Myxococcales	Haliangiaceae	Haliangium	-	-	-	-	-	-	-	-	-	-	-	-	ureA	-	-	-	-	chb	-	-	-	-	-	-	-
279355	0,0%	0,0%	0,0%	0,0%	0,0%	0,0%	0,0%	0,0%	0,0%	Bacteria	Planctomycetes	Planctomycetia	Gemmatales	Gemmataceae	uncultured	-	-	-	-	-	-	-	-	-	-	-	-	-	-	-	-	-	-	-	-	-	-	-	lip	-
224548	0,0%	0,0%	0,0%	0,1%	0,0%	0,0%	0,0%	0,0%	0,0%	Bacteria	Proteobacteria	Betaproteobacteria	SC-I-84		uncultured bacterium	-	-	-	-	-	-	-	-	-	-	-	-	-	-	-	-	-	-	-	-	-	-	-	-	-
206404	0,0%	0,0%	0,0%	0,0%	0,0%	0,0%	0,0%	0,1%	0,0%	Bacteria	Proteobacteria	Alphaproteobacteria			Rhizomicrobium	-	-	-	-	-	-	-	-	-	-	-	-	-	-	-	-	-	-	-	-	-	-	-	-	-
142193	0,0%	0,0%	0,0%	0,0%	0,0%	0,0%	0,0%	0,0%	0,0%	Bacteria	Chloroflexi	Anaerolineae	envOPS12		uncultured	-	-	-	-	-	-	-	-	-	-	-	-	-	-	-	-	-	-	-	-	-	-	-	lip	-
4401333	0,0%	0,0%	0,0%	0,0%	0,0%	0,0%	0,0%	0,0%	0,0%	Bacteria	Proteobacteria	Deltaproteobacteria	Myxococcales	Haliangiaceae	Haliangium	-	-	-	-	-	-	-	-	-	-	-	-	ureA	-	-	-	-	chb	-	-	-	-	-	-	-
1954099	0,0%	0,0%	0,0%	0,0%	0,0%	0,0%	0,0%	0,0%	0,0%	Bacteria	Proteobacteria	Gammaproteobacteria	Xanthomonadales	Sinobacteraceae	Steroidobacter	-	-	-	-	-	-	-	-	-	-	-	-	-	-	-	-	-	-	-	-	-	-	-	-	-
220629	0,0%	0,0%	0,0%	0,0%	0,0%	0,0%	0,0%	0,0%	0,0%	Bacteria	Verrucomicrobia	[Spartobacteria]	[Chthoniobacterales]	[Chthoniobacteraceae]	Chthoniobacter	-	napA	-	-	-	-	nirK	-	-	-	-	nrfA	ureA	-	-	-	-	-	-	-	-	-	-	-	-
579691	0,0%	0,0%	0,0%	0,1%	0,0%	0,0%	0,0%	0,0%	0,0%	Bacteria	Proteobacteria	Gammaproteobacteria	Legionellales	Legionellaceae	Legionella	-	-	-	-	-	nirS	-	norB	-	-	-	-	-	-	-	-	-	-	-	-	laccase_Asco	laccase_Basidio	-	-	ppo
3984866	0,0%	0,0%	0,0%	0,0%	0,0%	0,0%	0,0%	0,0%	0,0%	Bacteria	Proteobacteria	Alphaproteobacteria	Rhizobiales	Hyphomicrobiaceae	uncultured	-	-	-	-	-	-	-	-	-	-	-	-	-	-	-	-	-	-	-	-	-	-	-	lip	-
222534	0,0%	0,0%	0,0%	0,0%	0,0%	0,0%	0,0%	0,0%	0,0%	Bacteria	Proteobacteria	Gammaproteobacteria	Xanthomonadales	Xanthomonadaceae	Dyella	cellulolytic	napA	-	-	-	-	-	norB	nosZ	-	-	-	-	-	-	-	-	-	-	-	laccase_Asco	laccase_Basidio	-	-	ppo
4203522	0,0%	0,0%	0,0%	0,0%	0,0%	0,0%	0,0%	0,0%	0,1%	Bacteria	Acidobacteria	Solibacteres	Solibacterales		Bryobacter	-	-	-	-	-	-	-	-	-	-	-	-	-	-	-	-	-	-	-	-	-	-	-	-	-
1114786	0,0%	0,0%	0,0%	0,0%	0,0%	0,0%	0,0%	0,0%	0,0%	Bacteria	Verrucomicrobia	[Spartobacteria]	[Chthoniobacterales]	[Chthoniobacteraceae]	uncultured bacterium	-	-	-	-	-	-	-	-	-	-	-	-	-	-	-	-	-	-	-	-	-	-	-	-	-
4483006	0,0%	0,0%	0,0%	0,0%	0,0%	0,1%	0,0%	0,0%	0,0%	Bacteria	Firmicutes	Bacilli	Bacillales	Bacillaceae	Bacillus	cellulolytic	napA	narG	nirA	-	nirS	nirK	norB	nosZ	nifH	nifD	nrfA	ureA	-	-	-	-	-	mmoX	-	laccase_Asco	laccase_Basidio	-	-	ppo
100307	0,0%	0,0%	0,0%	0,0%	0,0%	0,0%	0,0%	0,0%	0,0%	Bacteria	Proteobacteria	Gammaproteobacteria	Xanthomonadales	Sinobacteraceae	uncultured bacterium	-	-	-	-	-	-	-	-	-	-	-	-	-	-	-	-	-	-	-	-	-	-	-	-	-
817152	0,0%	0,0%	0,0%	0,0%	0,0%	0,0%	0,0%	0,0%	0,0%	Bacteria	Actinobacteria	Acidimicrobiia	Acidimicrobiales	C111	uncultured	-	-	-	-	-	-	-	-	-	-	-	-	-	-	-	-	-	-	-	-	-	-	-	lip	-
340065	0,0%	0,0%	0,0%	0,1%	0,0%	0,0%	0,0%	0,0%	0,0%	Bacteria	Proteobacteria	Alphaproteobacteria	Rhodospirillales		Candidatus Alysiosphaera	-	-	-	-	-	-	-	-	-	-	-	-	-	-	-	-	-	-	-	-	-	-	-	-	-
1719556	0,0%	0,0%	0,0%	0,0%	0,0%	0,0%	0,0%	0,0%	0,0%	Bacteria	Planctomycetes	Planctomycetia	Gemmatales	Gemmataceae	Gemmata	-	-	-	-	-	-	-	-	-	-	-	-	-	-	-	-	-	-	-	-	-	-	-	-	-
161203	0,0%	0,0%	0,0%	0,0%	0,0%	0,0%	0,0%	0,0%	0,0%	Bacteria	Verrucomicrobia	[Methylacidiphilae]	S-BQ2-57		uncultured bacterium	-	-	-	-	-	-	-	-	-	-	-	-	-	-	-	-	-	-	-	-	-	-	-	-	-
223286	0,0%	0,0%	0,0%	0,0%	0,0%	0,0%	0,0%	0,0%	0,0%	Bacteria	Proteobacteria	Alphaproteobacteria	Rhodospirillales	Acetobacteraceae	uncultured	-	-	-	-	-	-	-	-	-	-	-	-	-	-	-	-	-	-	-	-	-	-	-	lip	-
574416	0,0%	0,0%	0,0%	0,0%	0,0%	0,0%	0,0%	0,0%	0,0%	Bacteria	Proteobacteria	Alphaproteobacteria	Caulobacterales	Caulobacteraceae	Phenylobacterium	-	napA	narG	-	-	-	nirK	norB	-	-	-	-	-	-	-	-	-	-	-	-	laccase_Asco	laccase_Basidio	-	-	ppo
573320	0,0%	0,0%	0,0%	0,0%	0,0%	0,0%	0,0%	0,0%	0,0%	Bacteria	Verrucomicrobia	[Pedosphaerae]	[Pedosphaerales]	auto67_4W	uncultured bacterium	-	-	-	-	-	-	-	-	-	-	-	-	-	-	-	-	-	-	-	-	-	-	-	-	-
942894	0,0%	0,0%	0,0%	0,0%	0,0%	0,0%	0,0%	0,0%	0,0%	Bacteria	Bacteroidetes	Flavobacteriia	Flavobacteriales	Cryomorphaceae	Fluviicola	-	-	-	nirA	-	-	-	norB	-	-	-	-	-	-	-	-	-	-	-	-	laccase_Asco	laccase_Basidio	-	-	ppo
818067	0,0%	0,0%	0,0%	0,0%	0,0%	0,0%	0,0%	0,0%	0,0%	Bacteria	TM6	SJA-4			uncultured bacterium	-	-	-	-	-	-	-	-	-	-	-	-	-	-	-	-	-	-	-	-	-	-	-	-	-
220524	0,0%	0,0%	0,0%	0,0%	0,0%	0,0%	0,0%	0,0%	0,0%	Bacteria	Actinobacteria	Actinobacteria	Actinomycetales	Nakamurellaceae	Nakamurella	-	napA	narG	-	-	-	-	-	-	-	-	-	ureA	-	-	-	-	-	-	-	-	laccase_Basidio	-	-	-
336493	0,0%	0,0%	0,0%	0,0%	0,0%	0,0%	0,0%	0,0%	0,0%	Bacteria	Proteobacteria	Deltaproteobacteria	PB19		uncultured bacterium	-	-	-	-	-	-	-	-	-	-	-	-	-	-	-	-	-	-	-	-	-	-	-	-	-
539206	0,0%	0,0%	0,0%	0,0%	0,0%	0,0%	0,0%	0,0%	0,0%	Bacteria	Bacteroidetes	Cytophagia	Cytophagales	Cytophagaceae	uncultured	cellulolytic	-	-	-	-	-	-	-	-	-	-	-	-	-	-	-	-	-	-	-	-	-	-	lip	-
4303249	0,0%	0,0%	0,0%	0,0%	0,0%	0,0%	0,0%	0,0%	0,0%	Bacteria	Proteobacteria	Alphaproteobacteria	Rhizobiales	Methylobacteriaceae	Methylobacterium	-	napA	narG	-	-	-	nirK	-	nosZ	nifH	nifD	-	ureA	-	-	-	-	-	-	-	laccase_Asco	laccase_Basidio	-	-	ppo
558853	0,0%	0,0%	0,0%	0,0%	0,0%	0,0%	0,0%	0,0%	0,0%	Bacteria	Proteobacteria	Deltaproteobacteria	Syntrophobacterales	Syntrophaceae	Desulfobacca	-	napA	-	-	-	-	-	-	-	nifH	nifD	-	-	dsrA	dsrB	-	-	-	-	-	-	-	-	-	-
771804	0,0%	0,1%	0,0%	0,0%	0,0%	0,0%	0,0%	0,0%	0,0%	Bacteria	Planctomycetes	Planctomycetia	Pirellulales	Pirellulaceae	Pirellula	-	napA	-	-	-	-	-	norB	-	-	-	-	-	-	-	-	-	-	-	-	-	-	-	-	-
1120920	0,0%	0,0%	0,0%	0,0%	0,0%	0,0%	0,0%	0,0%	0,0%	Bacteria	TM6	SJA-4			uncultured bacterium	-	-	-	-	-	-	-	-	-	-	-	-	-	-	-	-	-	-	-	-	-	-	-	-	-
915764	0,0%	0,0%	0,0%	0,0%	0,0%	0,0%	0,0%	0,0%	0,0%	Bacteria	Proteobacteria	Deltaproteobacteria	Myxococcales		uncultured delta proteobacterium	-	-	-	-	-	-	-	-	-	-	-	-	-	-	-	-	-	-	-	-	-	-	-	-	-
838940	0,0%	0,0%	0,0%	0,0%	0,0%	0,0%	0,0%	0,0%	0,0%	Bacteria	Actinobacteria	Actinobacteria	Actinomycetales	Microbacteriaceae	MWH-Ta3	cellulolytic	-	-	-	-	-	-	-	-	-	-	-	-	-	-	-	-	-	-	-	-	-	-	-	-
4311989	0,0%	0,0%	0,0%	0,0%	0,0%	0,0%	0,0%	0,0%	0,0%	Bacteria	Proteobacteria	Gammaproteobacteria	Methylococcales	Methylococcaceae	Methylomonas	-	napA	-	nirA	-	nirS	nirK	norB	-	nifH	nifD	-	ureA	-	-	-	amoA_AOB	-	mmoX	pmoA	laccase_Asco	laccase_Basidio	-	-	ppo
1656940	0,0%	0,0%	0,0%	0,0%	0,0%	0,0%	0,0%	0,0%	0,0%	Bacteria	Proteobacteria	Betaproteobacteria	Burkholderiales		Lautropia	-	-	narG	-	-	-	nirK	norB	nosZ	-	-	-	ureA	-	-	-	-	-	-	-	-	-	-	-	-
4459295	0,0%	0,0%	0,0%	0,0%	0,0%	0,0%	0,0%	0,0%	0,0%	Bacteria	Proteobacteria	Deltaproteobacteria	Desulfuromonadales	Geobacteraceae	uncultured bacterium	-	-	-	-	-	-	-	-	-	-	-	-	-	-	-	-	-	-	-	-	-	-	-	-	-
613333	0,0%	0,0%	0,0%	0,0%	0,0%	0,0%	0,0%	0,0%	0,0%	Bacteria	Planctomycetes	Planctomycetia	Pirellulales	Pirellulaceae	uncultured	-	-	-	-	-	-	-	-	-	-	-	-	-	-	-	-	-	-	-	-	-	-	-	lip	-
4414706	0,0%	0,0%	0,0%	0,0%	0,0%	0,0%	0,0%	0,0%	0,0%	Bacteria	Acidobacteria	Acidobacteriia	Acidobacteriales	Koribacteraceae	Candidatus Koribacter	-	-	-	-	-	-	-	-	-	-	-	-	-	-	-	-	-	-	-	-	-	-	-	-	-
2718809	0,0%	0,0%	0,0%	0,0%	0,0%	0,0%	0,0%	0,0%	0,0%	Bacteria	Planctomycetes	Planctomycetia	Gemmatales	Gemmataceae	uncultured	-	-	-	-	-	-	-	-	-	-	-	-	-	-	-	-	-	-	-	-	-	-	-	lip	-
4463100	0,0%	0,0%	0,0%	0,0%	0,0%	0,0%	0,0%	0,0%	0,0%	Bacteria	Bacteroidetes	[Saprospirae]	[Saprospirales]	Saprospiraceae	uncultured	-	-	-	-	-	-	-	-	-	-	-	-	-	-	-	-	-	-	-	-	-	-	-	lip	-
4375703	0,0%	0,0%	0,0%	0,0%	0,0%	0,0%	0,0%	0,0%	0,0%	Bacteria	Proteobacteria	Betaproteobacteria	Methylophilales	Methylophilaceae	LD28 freshwater group	-	-	-	-	-	-	-	-	-	-	-	-	-	-	-	-	-	-	-	-	-	-	-	-	-
661838	0,0%	0,0%	0,0%	0,0%	0,0%	0,0%	0,0%	0,0%	0,0%	Bacteria	Proteobacteria	Betaproteobacteria	Burkholderiales	Oxalobacteraceae	Janthinobacterium	-	napA	narG	nirA	-	nirS	-	norB	nosZ	-	-	-	ureA	-	-	SoxB	-	chb	-	-	-	laccase_Basidio	-	-	-
2139822	0,0%	0,0%	0,0%	0,0%	0,0%	0,0%	0,0%	0,0%	0,0%	Bacteria	Proteobacteria	Alphaproteobacteria	Caulobacterales	Caulobacteraceae	Caulobacter	-	napA	narG	nirA	-	-	nirK	norB	-	-	-	-	-	-	-	-	-	chb	-	-	laccase_Asco	laccase_Basidio	-	-	ppo
4391571	0,0%	0,0%	0,0%	0,0%	0,0%	0,0%	0,0%	0,0%	0,0%	Bacteria	Cyanobacteria	4C0d-2	MLE1-12		uncultured bacterium	-	-	-	-	-	-	-	-	-	-	-	-	-	-	-	-	-	-	-	-	-	-	-	-	-
1110000	0,0%	0,0%	0,0%	0,0%	0,0%	0,0%	0,0%	0,0%	0,0%	Bacteria	Bacteroidetes	Sphingobacteriia	Sphingobacteriales	Sphingobacteriaceae	Mucilaginibacter	-	-	-	-	-	-	-	-	-	-	-	-	-	-	-	-	-	-	-	-	-	-	-	-	-
153255	0,0%	0,0%	0,0%	0,0%	0,0%	0,0%	0,0%	0,0%	0,0%	Bacteria	Proteobacteria	Betaproteobacteria	Rhodocyclales	Rhodocyclaceae	Dechloromonas	-	napA	narG	-	-	nirS	-	norB	nosZ	nifH	nifD	-	ureA	-	-	SoxB	-	-	mmoX	-	-	-	-	-	-
529704	0,0%	0,0%	0,0%	0,0%	0,0%	0,0%	0,0%	0,0%	0,0%	Bacteria	Planctomycetes	Planctomycetia	Planctomycetales	Planctomycetaceae	Planctomyces	-	napA	-	-	-	-	-	norB	-	-	-	nrfA	-	-	-	-	-	-	-	-	-	-	-	-	-
556657	0,0%	0,0%	0,0%	0,0%	0,0%	0,0%	0,0%	0,0%	0,0%	Bacteria	Proteobacteria	Betaproteobacteria	Burkholderiales	Comamonadaceae	Acidovorax	-	napA	narG	nirA	-	nirS	nirK	norB	nosZ	-	-	-	ureA	-	-	-	-	-	mmoX	-	-	laccase_Basidio	-	-	ppo
661442	0,0%	0,0%	0,0%	0,0%	0,0%	0,0%	0,0%	0,0%	0,0%	Bacteria	Proteobacteria	Betaproteobacteria	Burkholderiales	Comamonadaceae	Acidovorax	-	napA	narG	nirA	-	nirS	nirK	norB	nosZ	-	-	-	ureA	-	-	-	-	-	mmoX	-	-	laccase_Basidio	-	-	ppo
342100	0,0%	0,0%	0,0%	0,0%	0,0%	0,0%	0,0%	0,0%	0,0%	Bacteria	Proteobacteria	Alphaproteobacteria	Rhodospirillales		Candidatus Alysiosphaera	-	-	-	-	-	-	-	-	-	-	-	-	-	-	-	-	-	-	-	-	-	-	-	-	-
878375	0,0%	0,0%	0,0%	0,0%	0,0%	0,0%	0,0%	0,0%	0,0%	Bacteria	Proteobacteria	Alphaproteobacteria	Rhizobiales	Methylobacteriaceae	Methylobacterium	-	napA	narG	-	-	-	nirK	-	nosZ	nifH	nifD	-	ureA	-	-	-	-	-	-	-	laccase_Asco	laccase_Basidio	-	-	ppo
2228269	0,0%	0,0%	0,0%	0,0%	0,0%	0,1%	0,0%	0,0%	0,0%	Bacteria	Proteobacteria	Betaproteobacteria	Neisseriales	Neisseriaceae	uncultured	-	-	-	-	-	-	-	-	-	-	-	-	-	-	-	-	-	-	-	-	-	-	-	lip	-
560704	0,0%	0,0%	0,0%	0,0%	0,0%	0,0%	0,0%	0,0%	0,0%	Bacteria	Bacteroidetes	Cytophagia	Cytophagales	Cytophagaceae	uncultured	cellulolytic	-	-	-	-	-	-	-	-	-	-	-	-	-	-	-	-	-	-	-	-	-	-	lip	-
7908	0,0%	0,0%	0,0%	0,0%	0,0%	0,0%	0,0%	0,0%	0,0%	Bacteria	Proteobacteria	Gammaproteobacteria	Legionellales	Legionellaceae	Legionella	-	-	-	-	-	nirS	-	norB	-	-	-	-	-	-	-	-	-	-	-	-	laccase_Asco	laccase_Basidio	-	-	ppo
4327601	0,0%	0,0%	0,0%	0,1%	0,0%	0,0%	0,0%	0,0%	0,0%	Bacteria	Proteobacteria	Betaproteobacteria	Methylophilales	Methylophilaceae	Methylotenera	-	napA	-	nirA	-	-	nirK	norB	-	-	-	-	ureA	-	-	-	-	-	-	-	-	-	-	-	-
205306	0,0%	0,0%	0,0%	0,0%	0,0%	0,0%	0,0%	0,0%	0,0%	Bacteria	Proteobacteria	Betaproteobacteria	SC-I-84		uncultured	-	-	-	-	-	-	-	-	-	-	-	-	-	-	-	-	-	-	-	-	-	-	-	lip	-
4393043	0,0%	0,0%	0,0%	0,0%	0,0%	0,0%	0,0%	0,0%	0,0%	Bacteria	Proteobacteria	Deltaproteobacteria	MIZ46		uncultured bacterium	-	-	-	-	-	-	-	-	-	-	-	-	-	-	-	-	-	-	-	-	-	-	-	-	-
4348329	0,0%	0,0%	0,0%	0,0%	0,0%	0,0%	0,0%	0,0%	0,1%	Bacteria	Actinobacteria	Acidimicrobiia	Acidimicrobiales	koll13	uncultured Gram-positive bacterium	-	-	-	-	-	-	-	-	-	-	-	-	-	-	-	-	-	-	-	-	-	-	-	-	-
329035	0,0%	0,0%	0,0%	0,0%	0,0%	0,0%	0,0%	0,0%	0,0%	Bacteria	Proteobacteria	Deltaproteobacteria	Myxococcales	Polyangiaceae	Byssovorax	-	-	-	-	-	-	-	-	-	-	-	-	-	-	-	-	-	-	-	-	-	-	-	-	-
4300016	0,0%	0,0%	0,0%	0,0%	0,0%	0,0%	0,0%	0,0%	0,0%	Bacteria	Proteobacteria	Gammaproteobacteria	Thiotrichales	Piscirickettsiaceae	uncultured bacterium	-	-	-	-	-	-	-	-	-	-	-	-	-	-	-	-	-	-	-	-	-	-	-	-	-
947930	0,0%	0,0%	0,0%	0,0%	0,0%	0,0%	0,0%	0,0%	0,0%	Bacteria	Firmicutes	Bacilli	Bacillales	Paenibacillaceae	Paenibacillus	-	napA	narG	-	-	nirS	nirK	norB	nosZ	nifH	nifD	-	ureA	-	-	-	-	-	-	-	laccase_Asco	laccase_Basidio	-	-	ppo
4418260	0,0%	0,0%	0,0%	0,0%	0,0%	0,0%	0,0%	0,0%	0,0%	Bacteria	Proteobacteria	Betaproteobacteria	Burkholderiales	Comamonadaceae	Rhizobacter	-	napA	narG	nirA	-	-	-	norB	-	-	-	-	ureA	-	-	-	-	-	-	-	-	-	-	-	-
1142898	0,0%	0,0%	0,0%	0,0%	0,0%	0,0%	0,0%	0,0%	0,0%	Bacteria	Proteobacteria	Alphaproteobacteria	Rhizobiales	Methylocystaceae	Rhodoblastus	-	-	-	-	-	-	-	-	-	nifH	-	-	-	-	-	-	-	-	-	-	-	-	-	-	-
64222	0,0%	0,0%	0,0%	0,0%	0,0%	0,0%	0,0%	0,0%	0,0%	Bacteria	Proteobacteria	Alphaproteobacteria	Rhizobiales	Methylocystaceae	uncultured	-	-	-	-	-	-	-	-	-	-	-	-	-	-	-	-	-	-	-	-	-	-	-	lip	-
218479	0,0%	0,0%	0,0%	0,0%	0,0%	0,0%	0,0%	0,0%	0,0%	Bacteria	Proteobacteria	Gammaproteobacteria	Xanthomonadales	Xanthomonadaceae	uncultured	cellulolytic	-	-	-	-	-	-	-	-	-	-	-	-	-	-	-	-	-	-	-	-	-	-	lip	-
4111963	0,0%	0,0%	0,0%	0,0%	0,0%	0,0%	0,0%	0,0%	0,0%	Bacteria	Bacteroidetes	[Saprospirae]	[Saprospirales]	Chitinophagaceae	Sediminibacterium	-	-	-	-	-	-	-	-	-	-	-	-	-	-	-	-	-	-	-	-	-	-	-	-	-
1145162	0,0%	0,0%	0,0%	0,0%	0,0%	0,0%	0,0%	0,0%	0,0%	Bacteria	Proteobacteria	Betaproteobacteria	Burkholderiales	Comamonadaceae	uncultured	-	-	-	-	-	-	-	-	-	-	-	-	-	-	-	-	-	-	-	-	-	-	-	lip	-
4425872	0,0%	0,0%	0,0%	0,0%	0,0%	0,0%	0,0%	0,0%	0,0%	Bacteria	Bacteroidetes	[Saprospirae]	[Saprospirales]	Chitinophagaceae	Ferruginibacter	-	-	-	-	-	-	-	-	-	-	-	-	-	-	-	-	-	-	-	-	-	-	-	-	-
4335789	0,0%	0,0%	0,0%	0,0%	0,0%	0,0%	0,0%	0,0%	0,1%	Bacteria	Proteobacteria	Gammaproteobacteria	Legionellales	Legionellaceae	Legionella	-	-	-	-	-	nirS	-	norB	-	-	-	-	-	-	-	-	-	-	-	-	laccase_Asco	laccase_Basidio	-	-	ppo

850137	0,0%	0,0%	0,0%	0,0%	0,0%	0,0%	0,0%	0,0%	0,0%	Bacteria	Proteobacteria	Alphaproteobacteria	Caulobacterales	Caulobacteraceae	Asticcacaulis	-	napA	narG	nirA	-	-	-	-	-	-	-	-	-	-	-	-	-	chb	-	-	laccase_Asco	laccase_Basidio	-	-	ppo
265527	0,0%	0,0%	0,0%	0,0%	0,0%	0,0%	0,0%	0,0%	0,0%	Bacteria	Proteobacteria	Gammaproteobacteria	Methylococcales	Methylococcaceae	Crenothrix	-	-	-	-	-	-	-	-	-	-	-	-	-	-	-	-	-	-	-	pmoA	-	-	-	-	-
821362	0,0%	0,0%	0,0%	0,0%	0,0%	0,0%	0,0%	0,0%	0,1%	Bacteria	Bacteroidetes	Cytophagia	Cytophagales	Cytophagaceae	uncultured	cellulolytic	-	-	-	-	-	-	-	-	-	-	-	-	-	-	-	-	-	-	-	-	-	-	lip	-
2211108	0,0%	0,0%	0,0%	0,0%	0,0%	0,0%	0,0%	0,0%	0,0%	Bacteria	Proteobacteria	Alphaproteobacteria	Caulobacterales	Caulobacteraceae	Brevundimonas	-	-	-	nirA	-	-	-	norB	-	nifH	-	-	-	-	-	-	-	-	mmoX	-	laccase_Asco	laccase_Basidio	-	-	ppo
509881	0,0%	0,0%	0,0%	0,0%	0,0%	0,0%	0,0%	0,0%	0,0%	Bacteria	Actinobacteria	Thermoleophilia	Solirubrobacterales		uncultured bacterium	-	-	-	-	-	-	-	-	-	-	-	-	-	-	-	-	-	-	-	-	-	-	-	-	-
575223	0,0%	0,0%	0,0%	0,0%	0,0%	0,0%	0,0%	0,0%	0,0%	Bacteria	Verrucomicrobia	[Pedosphaerae]	[Pedosphaerales]	auto67_4W	uncultured bacterium	-	-	-	-	-	-	-	-	-	-	-	-	-	-	-	-	-	-	-	-	-	-	-	-	-
2753150	0,0%	0,0%	0,0%	0,0%	0,0%	0,0%	0,0%	0,0%	0,0%	Bacteria	Actinobacteria	Acidimicrobiia	Acidimicrobiales		uncultured	-	-	-	-	-	-	-	-	-	-	-	-	-	-	-	-	-	-	-	-	-	-	-	lip	-
1114591	0,0%	0,0%	0,0%	0,0%	0,0%	0,0%	0,0%	0,0%	0,0%	Bacteria	Bacteroidetes	Sphingobacteriia	Sphingobacteriales		uncultured bacterium	-	-	-	-	-	-	-	-	-	-	-	-	-	-	-	-	-	-	-	-	-	-	-	-	-
4484204	0,0%	0,0%	0,0%	0,0%	0,0%	0,0%	0,0%	0,0%	0,0%	Bacteria	Bacteroidetes	Bacteroidia	Bacteroidales		uncultured bacterium	-	-	-	-	-	-	-	-	-	-	-	-	-	-	-	-	-	-	-	-	-	-	-	-	-
1071596	0,0%	0,0%	0,0%	0,0%	0,0%	0,0%	0,0%	0,0%	0,0%	Bacteria	Proteobacteria	Alphaproteobacteria	Rhizobiales	Hyphomicrobiaceae	uncultured	-	-	-	-	-	-	-	-	-	-	-	-	-	-	-	-	-	-	-	-	-	-	-	lip	-
4351336	0,0%	0,0%	0,0%	0,0%	0,0%	0,0%	0,0%	0,0%	0,0%	Bacteria	Proteobacteria	Betaproteobacteria	Burkholderiales	Comamonadaceae	Variovorax	-	napA	narG	nirA	-	-	-	norB	-	-	-	-	ureA	-	-	-	-	-	mmoX	-	-	-	-	-	-
2033299	0,0%	0,0%	0,0%	0,0%	0,0%	0,0%	0,0%	0,0%	0,0%	Bacteria	Proteobacteria	Gammaproteobacteria	Alteromonadales	[Chromatiaceae]	Rheinheimera	-	-	narG	-	-	-	-	norB	-	-	-	-	-	-	-	-	-	chb	-	-	-	laccase_Basidio	-	-	-
1114023	0,0%	0,0%	0,0%	0,0%	0,0%	0,0%	0,0%	0,0%	0,0%	Bacteria	Bacteroidetes	Flavobacteriia	Flavobacteriales	Flavobacteriaceae	Flavobacterium	cellulolytic	napA	-	nirA	-	-	nirK	norB	nosZ	-	-	-	ureA	-	-	-	-	-	-	-	laccase_Asco	laccase_Basidio	-	-	ppo
4445104	0,0%	0,0%	0,0%	0,0%	0,0%	0,0%	0,0%	0,0%	0,0%	Bacteria	Proteobacteria	Betaproteobacteria	Burkholderiales	Oxalobacteraceae	Massilia	-	napA	narG	-	-	-	-	norB	nosZ	-	-	-	ureA	-	-	-	-	chb	mmoX	-	laccase_Asco	laccase_Basidio	-	-	ppo
1115511	0,0%	0,0%	0,0%	0,0%	0,0%	0,1%	0,0%	0,0%	0,0%	Bacteria	Proteobacteria	Betaproteobacteria	Methylophilales	Methylophilaceae	Methylotenera	-	napA	-	nirA	-	-	nirK	norB	-	-	-	-	ureA	-	-	-	-	-	-	-	-	-	-	-	-
4326776	0,0%	0,0%	0,0%	0,0%	0,0%	0,0%	0,0%	0,0%	0,0%	Bacteria	Proteobacteria	Gammaproteobacteria	Legionellales	Coxiellaceae	Aquicella	-	-	-	-	-	-	-	-	-	-	-	-	-	-	-	-	-	-	-	-	-	-	-	-	-
3270631	0,0%	0,0%	0,0%	0,0%	0,0%	0,0%	0,0%	0,0%	0,0%	Bacteria	Acidobacteria	Acidobacteriia	Acidobacteriales	Koribacteraceae	uncultured	-	-	-	-	-	-	-	-	-	-	-	-	-	-	-	-	-	-	-	-	-	-	-	lip	-
37092	0,0%	0,0%	0,0%	0,0%	0,0%	0,0%	0,0%	0,0%	0,0%	Bacteria	Proteobacteria	Alphaproteobacteria	Sphingomonadales	Sphingomonadaceae	uncultured bacterium	-	-	-	-	-	-	-	-	-	-	-	-	-	-	-	-	-	-	-	-	-	-	-	-	-
1129425	0,0%	0,0%	0,0%	0,0%	0,0%	0,0%	0,0%	0,0%	0,0%	Bacteria	Actinobacteria	Thermoleophilia	Solirubrobacterales	Solirubrobacteraceae	Solirubrobacter	-	-	-	-	-	-	-	-	-	-	-	-	-	-	-	-	-	-	-	-	-	-	-	-	-
380204	0,0%	0,0%	0,0%	0,0%	0,0%	0,0%	0,0%	0,0%	0,1%	Bacteria	Proteobacteria	Gammaproteobacteria	Methylococcales	Methylococcaceae	Methylocaldum	-	-	-	-	-	-	-	-	-	nifH	-	-	-	-	-	-	-	-	mmoX	pmoA	-	-	-	-	-
4374946	0,0%	0,0%	0,0%	0,0%	0,0%	0,0%	0,0%	0,0%	0,0%	Bacteria	Proteobacteria	Betaproteobacteria	Burkholderiales		Azospira	-	-	narG	-	-	-	nirK	-	-	nifH	-	-	-	-	-	-	-	-	-	-	-	-	-	-	-
1054903	0,0%	0,0%	0,0%	0,0%	0,0%	0,0%	0,0%	0,0%	0,1%	Bacteria	Proteobacteria	Gammaproteobacteria	Legionellales	Coxiellaceae	Aquicella	-	-	-	-	-	-	-	-	-	-	-	-	-	-	-	-	-	-	-	-	-	-	-	-	-
4379895	0,0%	0,0%	0,0%	0,0%	0,0%	0,0%	0,0%	0,0%	0,0%	Bacteria	Proteobacteria	Alphaproteobacteria	Caulobacterales	Caulobacteraceae	Brevundimonas	-	-	-	nirA	-	-	-	norB	-	nifH	-	-	-	-	-	-	-	-	mmoX	-	laccase_Asco	laccase_Basidio	-	-	ppo
4322256	0,0%	0,0%	0,0%	0,0%	0,0%	0,0%	0,0%	0,0%	0,0%	Bacteria	Proteobacteria	Alphaproteobacteria	Rhodospirillales	Acetobacteraceae	uncultured eubacterium WD248	-	-	-	-	-	-	-	-	-	-	-	-	-	-	-	-	-	-	-	-	-	-	-	-	-
136767	0,0%	0,0%	0,0%	0,0%	0,0%	0,0%	0,0%	0,0%	0,0%	Bacteria	Proteobacteria	Gammaproteobacteria	Chromatiales	Chromatiaceae	Thiocystis	-	napA	-	-	-	-	-	norB	-	nifH	nifD	-	ureA	dsrA	dsrB	-	-	-	-	-	-	-	-	-	-
142471	0,0%	0,0%	0,0%	0,0%	0,0%	0,0%	0,0%	0,0%	0,0%	Bacteria	Verrucomicrobia	Verrucomicrobiae	Verrucomicrobiales	Verrucomicrobiaceae	uncultured	-	-	-	-	-	-	-	-	-	-	-	-	-	-	-	-	-	-	-	-	-	-	-	lip	-
99309	0,0%	0,0%	0,0%	0,0%	0,0%	0,0%	0,0%	0,0%	0,0%	Bacteria	Proteobacteria	Betaproteobacteria	Burkholderiales	Comamonadaceae	Comamonas	-	napA	-	nirA	-	nirS	nirK	norB	nosZ	-	-	-	ureA	-	-	SoxB	-	-	mmoX	-	-	laccase_Basidio	-	-	-
899404	0,0%	0,0%	0,0%	0,0%	0,0%	0,0%	0,0%	0,0%	0,0%	Bacteria	Actinobacteria	Actinobacteria	Actinomycetales		Acidothermus	-	napA	narG	-	-	-	nirK	-	-	-	-	-	-	-	-	-	-	-	-	-	-	-	-	-	-
552338	0,0%	0,0%	0,0%	0,0%	0,0%	0,0%	0,0%	0,0%	0,1%	Bacteria	Proteobacteria	Deltaproteobacteria	Myxococcales		uncultured bacterium	-	-	-	-	-	-	-	-	-	-	-	-	-	-	-	-	-	-	-	-	-	-	-	-	-
851113	0,0%	0,0%	0,0%	0,0%	0,0%	0,0%	0,0%	0,0%	0,0%	Bacteria	Bacteroidetes	Sphingobacteriia	Sphingobacteriales	Sphingobacteriaceae	Mucilaginibacter	-	-	-	-	-	-	-	-	-	-	-	-	-	-	-	-	-	-	-	-	-	-	-	-	-
4391730	0,0%	0,0%	0,0%	0,0%	0,0%	0,0%	0,0%	0,0%	0,0%	Bacteria	Planctomycetes	Planctomycetia	Gemmatales	Gemmataceae	uncultured	-	-	-	-	-	-	-	-	-	-	-	-	-	-	-	-	-	-	-	-	-	-	-	lip	-
156227	0,0%	0,0%	0,0%	0,0%	0,0%	0,0%	0,0%	0,0%	0,0%	Bacteria	Proteobacteria	Alphaproteobacteria	Rhizobiales	Hyphomicrobiaceae	Rhodomicrobium	-	-	-	nirA	-	-	-	-	-	nifH	nifD	-	-	dsrA	dsrB	-	-	-	-	-	-	-	-	-	-
4407305	0,0%	0,0%	0,0%	0,0%	0,0%	0,0%	0,0%	0,0%	0,0%	Bacteria	Firmicutes	Clostridia	Clostridiales	Clostridiaceae	Clostridium sensu stricto 1	cellulolytic	-	-	-	-	-	-	-	-	-	-	-	-	-	-	-	-	-	-	-	-	-	-	-	-
971170	0,0%	0,0%	0,0%	0,0%	0,0%	0,0%	0,0%	0,1%	0,0%	Bacteria	Verrucomicrobia	[Spartobacteria]	[Chthoniobacterales]	[Chthoniobacteraceae]	Chthoniobacter	-	napA	-	-	-	-	nirK	-	-	-	-	nrfA	ureA	-	-	-	-	-	-	-	-	-	-	-	-
364780	0,0%	0,0%	0,0%	0,0%	0,0%	0,0%	0,0%	0,0%	0,0%	Bacteria	Proteobacteria	Alphaproteobacteria	Rhizobiales		Methylorosula	-	-	-	-	-	-	-	-	-	-	-	-	-	-	-	-	-	-	-	-	-	-	-	-	-
142277	0,0%	0,0%	0,0%	0,0%	0,0%	0,0%	0,0%	0,0%	0,0%	Bacteria	Firmicutes	Clostridia	Clostridiales	Ruminococcaceae	Incertae Sedis	cellulolytic	-	-	-	-	-	-	-	-	-	-	-	-	-	-	-	-	-	-	-	-	-	-	-	-
804828	0,0%	0,0%	0,0%	0,0%	0,0%	0,0%	0,0%	0,0%	0,0%	Bacteria	Planctomycetes	Planctomycetia	Gemmatales	Isosphaeraceae	Singulisphaera	-	napA	-	-	-	-	-	-	-	-	-	-	ureA	-	-	-	-	-	-	-	-	-	-	-	-
119236	0,0%	0,0%	0,0%	0,0%	0,0%	0,0%	0,0%	0,0%	0,0%	Bacteria	Proteobacteria	Deltaproteobacteria	MIZ46		uncultured bacterium	-	-	-	-	-	-	-	-	-	-	-	-	-	-	-	-	-	-	-	-	-	-	-	-	-
4416105	0,0%	0,0%	0,0%	0,0%	0,0%	0,1%	0,0%	0,0%	0,0%	Bacteria	Actinobacteria	Actinobacteria	Actinomycetales	Microbacteriaceae	Alpinimonas	cellulolytic	-	-	-	-	-	-	-	-	-	-	-	-	-	-	-	-	-	-	-	-	-	-	-	-
843029	0,0%	0,0%	0,0%	0,0%	0,0%	0,0%	0,0%	0,0%	0,0%	Bacteria	Planctomycetes	Planctomycetia	Planctomycetales	Planctomycetaceae	Planctomyces	-	napA	-	-	-	-	-	norB	-	-	-	nrfA	-	-	-	-	-	-	-	-	-	-	-	-	-
4360561	0,0%	0,0%	0,0%	0,0%	0,0%	0,0%	0,0%	0,0%	0,0%	Bacteria	Proteobacteria	Deltaproteobacteria			uncultured marine bacterium	-	-	-	-	-	-	-	-	-	-	-	-	-	-	-	-	-	-	-	-	-	-	-	-	-
729766	0,0%	0,0%	0,0%	0,0%	0,0%	0,0%	0,0%	0,0%	0,0%	Bacteria	Bacteroidetes	Sphingobacteriia	Sphingobacteriales		bacterium enrichment culture clone auto79_4W	-	-	-	-	-	-	-	-	-	-	-	-	-	-	-	-	-	-	-	-	-	-	-	-	-
4428468	0,0%	0,0%	0,0%	0,0%	0,0%	0,0%	0,0%	0,0%	0,0%	Bacteria	Proteobacteria	Gammaproteobacteria	Pseudomonadales	Moraxellaceae	Perlucidibaca	-	-	-	-	-	-	-	-	-	-	-	-	-	-	-	-	-	-	-	-	-	-	-	-	-
40351	0,0%	0,0%	0,0%	0,0%	0,0%	0,0%	0,0%	0,0%	0,0%	Bacteria	Bacteroidetes	Sphingobacteriia	Sphingobacteriales		uncultured bacterium	-	-	-	-	-	-	-	-	-	-	-	-	-	-	-	-	-	-	-	-	-	-	-	-	-
4412914	0,0%	0,0%	0,0%	0,0%	0,0%	0,0%	0,0%	0,0%	0,0%	Bacteria	Planctomycetes	Planctomycetia	Gemmatales	Gemmataceae	uncultured	-	-	-	-	-	-	-	-	-	-	-	-	-	-	-	-	-	-	-	-	-	-	-	lip	-
4446019	0,0%	0,0%	0,0%	0,0%	0,0%	0,0%	0,0%	0,0%	0,0%	Bacteria	Acidobacteria	Acidobacteria-6	iii1-15		uncultured Acidobacteria bacterium	-	-	-	-	-	-	-	-	-	-	-	-	-	-	-	-	-	-	-	-	-	-	-	-	-
245601	0,0%	0,0%	0,0%	0,0%	0,0%	0,0%	0,0%	0,0%	0,0%	Bacteria	Proteobacteria	Alphaproteobacteria	Rhizobiales	Bradyrhizobiaceae	Bradyrhizobium	-	napA	narG	nirA	-	nirS	nirK	norB	nosZ	nifH	nifD	-	ureA	-	-	SoxB	-	-	mmoX	-	laccase_Asco	laccase_Basidio	-	-	ppo
194304	0,0%	0,0%	0,0%	0,0%	0,0%	0,0%	0,0%	0,0%	0,0%	Bacteria	Cyanobacteria	Oscillatoriophycideae	Chroococcales	Xenococcaceae	Pleurocapsa	-	napA	-	-	-	-	-	norB	-	-	nifD	-	ureA	-	-	-	-	-	-	-	-	laccase_Basidio	-	-	ppo
614357	0,0%	0,0%	0,0%	0,0%	0,0%	0,0%	0,0%	0,0%	0,0%	Bacteria	Proteobacteria	Alphaproteobacteria	Rhizobiales	Methylocystaceae	uncultured alpha proteobacterium	-	-	-	-	-	-	-	-	-	-	-	-	-	-	-	-	-	-	-	-	-	-	-	-	-
4442930	0,0%	0,0%	0,0%	0,0%	0,0%	0,1%	0,0%	0,0%	0,0%	Bacteria	Proteobacteria	Betaproteobacteria	Burkholderiales	Comamonadaceae	uncultured	-	-	-	-	-	-	-	-	-	-	-	-	-	-	-	-	-	-	-	-	-	-	-	lip	-
766989	0,0%	0,0%	0,0%	0,0%	0,0%	0,0%	0,0%	0,0%	0,0%	Bacteria	Bacteroidetes	[Saprospirae]	[Saprospirales]	Chitinophagaceae	Ferruginibacter	-	-	-	-	-	-	-	-	-	-	-	-	-	-	-	-	-	-	-	-	-	-	-	-	-
769643	0,0%	0,0%	0,0%	0,0%	0,0%	0,0%	0,0%	0,0%	0,0%	Bacteria	Proteobacteria	Gammaproteobacteria	Pseudomonadales	Pseudomonadaceae	Pseudomonas	cellulolytic	napA	narG	nirA	-	nirS	nirK	norB	nosZ	nifH	nifD	-	ureA	-	dsrB	SoxB	-	-	mmoX	-	laccase_Asco	laccase_Basidio	-	-	ppo
136162	0,0%	0,0%	0,0%	0,1%	0,0%	0,0%	0,0%	0,0%	0,0%	Bacteria	Proteobacteria	Alphaproteobacteria	Rhizobiales	Hyphomicrobiaceae	uncultured	-	-	-	-	-	-	-	-	-	-	-	-	-	-	-	-	-	-	-	-	-	-	-	lip	-
815546	0,0%	0,0%	0,0%	0,0%	0,0%	0,0%	0,0%	0,0%	0,0%	Bacteria	Acidobacteria	DA052	Ellin6513		uncultured Acidobacteria bacterium	-	-	-	-	-	-	-	-	-	-	-	-	-	-	-	-	-	-	-	-	-	-	-	-	-
4461200	0,0%	0,0%	0,0%	0,0%	0,0%	0,0%	0,0%	0,0%	0,0%	Bacteria	Proteobacteria	Gammaproteobacteria	Oceanospirillales	Halomonadaceae	Halomonas	-	napA	narG	nirA	-	nirS	-	norB	nosZ	nifH	-	nrfA	ureA	-	-	-	-	chb	-	-	laccase_Asco	laccase_Basidio	-	-	ppo
151539	0,0%	0,0%	0,0%	0,0%	0,0%	0,0%	0,0%	0,0%	0,0%	Bacteria	Acidobacteria	Acidobacteriia	Acidobacteriales	Acidobacteriaceae	uncultured	cellulolytic	-	-	-	-	-	-	-	-	-	-	-	-	-	-	-	-	-	-	-	-	-	-	lip	-
1133992	0,0%	0,0%	0,0%	0,0%	0,0%	0,0%	0,0%	0,0%	0,1%	Bacteria	Proteobacteria	Alphaproteobacteria	Rickettsiales		Caedibacter caryophilus	-	-	-	-	-	-	-	-	-	-	-	-	-	-	-	-	-	-	-	-	-	-	-	-	-
1069437	0,0%	0,0%	0,0%	0,0%	0,0%	0,0%	0,0%	0,0%	0,0%	Bacteria	Actinobacteria	Thermoleophilia	Solirubrobacterales		uncultured actinobacterium	-	-	-	-	-	-	-	-	-	-	-	-	-	-	-	-	-	-	-	-	-	-	-	-	-
4445692	0,0%	0,0%	0,0%	0,0%	0,0%	0,0%	0,0%	0,0%	0,0%	Bacteria	Proteobacteria	Gammaproteobacteria	Aeromonadales	Aeromonadaceae	uncultured bacterium	-	-	-	-	-	-	-	-	-	-	-	-	-	-	-	-	-	-	-	-	-	-	-	-	-
1118818	0,0%	0,0%	0,0%	0,0%	0,0%	0,1%	0,0%	0,0%	0,0%	Bacteria	TM6	SJA-4			uncultured bacterium	-	-	-	-	-	-	-	-	-	-	-	-	-	-	-	-	-	-	-	-	-	-	-	-	-
3292593	0,0%	0,0%	0,0%	0,0%	0,0%	0,0%	0,0%	0,0%	0,0%	Bacteria	Acidobacteria	Solibacteres	Solibacterales	Solibacteraceae	Candidatus Solibacter	-	-	-	-	-	-	-	-	-	-	-	-	-	-	-	-	-	-	-	-	-	-	-	-	-
156210	0,0%	0,0%	0,0%	0,0%	0,0%	0,0%	0,0%	0,0%	0,0%	Bacteria	Verrucomicrobia	Opitutae	Opitutales	Opitutaceae	Opitutus	-	napA	-	nirA	-	-	-	-	nosZ	-	-	nrfA	ureA	-	-	-	-	-	-	-	-	laccase_Basidio	-	-	-
4319686	0,0%	0,0%	0,0%	0,0%	0,0%	0,0%	0,0%	0,0%	0,0%	Bacteria	Proteobacteria	Gammaproteobacteria	Xanthomonadales	Xanthomonadaceae	Stenotrophomonas	cellulolytic	napA	narG	-	-	-	-	norB	-	nifH	-	-	-	-	-	-	-	-	-	-	laccase_Asco	laccase_Basidio	-	-	ppo
4460868	0,0%	0,0%	0,0%	0,0%	0,0%	0,0%	0,0%	0,0%	0,0%	Bacteria	Proteobacteria	Betaproteobacteria	Burkholderiales	Comamonadaceae	Acidovorax	-	napA	narG	nirA	-	nirS	nirK	norB	nosZ	-	-	-	ureA	-	-	-	-	-	mmoX	-	-	laccase_Basidio	-	-	ppo
3232050	0,0%	0,0%	0,0%	0,0%	0,0%	0,0%	0,0%	0,0%	0,0%	Bacteria	Verrucomicrobia	[Pedosphaerae]	[Pedosphaerales]	Ellin515	uncultured bacterium	-	-	-	-	-	-	-	-	-	-	-	-	-	-	-	-	-	-	-	-	-	-	-	-	-
533024	0,0%	0,0%	0,0%	0,0%	0,0%	0,1%	0,0%	0,0%	0,0%	Bacteria	Bacteroidetes	Sphingobacteriia	Sphingobacteriales		uncultured bacterium	-	-	-	-	-	-	-	-	-	-	-	-	-	-	-	-	-	-	-	-	-	-	-	-	-
913399	0,0%	0,0%	0,0%	0,0%	0,0%	0,0%	0,0%	0,0%	0,0%	Bacteria	Bacteroidetes	BME43			uncultured	-	-	-	-	-	-	-	-	-	-	-	-	-	-	-	-	-	-	-	-	-	-	-	lip	-
541736	0,0%	0,0%	0,0%	0,0%	0,0%	0,0%	0,0%	0,0%	0,0%	Bacteria	Proteobacteria	Alphaproteobacteria	Rhizobiales	Methylocystaceae	Methylocystis	-	napA	-	nirA	-	-	nirK	norB	nosZ	nifH	nifD	-	-	-	-	-	-	-	mmoX	pmoA	laccase_Asco	laccase_Basidio	-	-	ppo
4385224	0,0%	0,0%	0,0%	0,0%	0,0%	0,0%	0,0%	0,0%	0,0%	Bacteria	Actinobacteria	Actinobacteria	Actinomycetales	Microbacteriaceae	Microbacterium	cellulolytic	napA	narG	-	-	-	-	-	-	nifH	-	-	ureA	-	-	-	amoA_AOB	-	-	pmoA	laccase_Asco	laccase_Basidio	-	-	ppo
4353904	0,0%	0,0%	0,0%	0,0%	0,0%	0,1%	0,0%	0,0%	0,0%	Bacteria	Proteobacteria	Deltaproteobacteria	Myxococcales		Haliangium	-	-	-	-	-	-	-	-	-	-	-	-	ureA	-	-	-	-	chb	-	-	-	-	-	-	-
3341679	0,0%	0,0%	0,0%	0,0%	0,0%	0,0%	0,0%	0,1%	0,0%	Bacteria	Gemmatimonadetes	Gemmatimonadetes	Gemmatimonadales	Gemmatimonadaceae	Gemmatimonas	-	napA	-	-	-	-	nirK	norB	nosZ	-	-	nrfA	-	dsrA	dsrB	-	-	chb	-	-	-	-	-	-	-
4397087	0,0%	0,0%	0,0%	0,0%	0,0%	0,0%	0,0%	0,0%	0,1%	Bacteria	Bacteroidetes	Sphingobacteriia	Sphingobacteriales	Sphingobacteriaceae	Mucilaginibacter	-	-	-	-	-	-	-	-	-	-	-	-	-	-	-	-	-	-	-	-	-	-	-	-	-
151797	0,0%	0,0%	0,0%	0,0%	0,0%	0,0%	0,0%	0,0%	0,0%	Bacteria	Proteobacteria	Betaproteobacteria	Neisseriales	Neisseriaceae	Chitinibacter	-	-	-	-	-	-	-	-	-	-	-	-	-	-	-	-	-	-	-	-	-	-	-	-	-
959929	0,0%	0,0%	0,0%	0,0%	0,0%	0,0%	0,0%	0,0%	0,1%	Bacteria	Proteobacteria	Deltaproteobacteria	MIZ46		uncultured bacterium	-	-	-	-	-	-	-	-	-	-	-	-	-	-	-	-	-	-	-	-	-	-	-	-	-
574086	0,0%	0,0%	0,0%	0,0%	0,0%	0,1%	0,0%	0,0%	0,0%	Bacteria	Proteobacteria	Alphaproteobacteria	Rhizobiales	Hyphomicrobiaceae	Pedomicrobium	-	-	-	-	-	-	nirK	-	-	-	-	-	-	-	-	-	-	-	-	-	-	-	-	-	-
4421805	0,0%	0,0%	0,0%	0,0%	0,0%	0,0%	0,0%	0,0%	0,0%	Bacteria	Actinobacteria	Actinobacteria	Actinomycetales	Microbacteriaceae	Subtercola	cellulolytic	-	-	-	-	-	-	-	-	-	-	-	-	-	-	-	-	-	-	-	-	-	-	-	-
143746	0,0%	0,0%	0,0%	0,0%	0,0%	0,0%	0,0%	0,0%	0,0%	Bacteria	Proteobacteria	Alphaproteobacteria	Rickettsiales		Candidatus Captivus	-	-	-	-	-	-	-	-	-	-	-	-	-	-	-	-	-	-	-	-	-	-	-	-	-
1065350	0,0%	0,0%	0,0%	0,0%	0,0%	0,0%	0,0%	0,0%	0,0%	Bacteria	Bacteroidetes	Bacteroidia	Bacteroidales		uncultured bacterium	-	-	-	-	-	-	-	-	-	-	-	-	-	-	-	-	-	-	-	-	-	-	-	-	-
217769	0,0%	0,0%	0,0%	0,0%	0,0%	0,1%	0,0%	0,0%	0,0%	Bacteria	Bacteroidetes	Sphingobacteriia	Sphingobacteriales	Sphingobacteriaceae	Mucilaginibacter	-	-	-	-	-	-	-	-	-	-	-	-	-	-	-	-	-	-	-	-	-	-	-	-	-
143744	0,0%	0,0%	0,0%	0,0%	0,0%	0,0%	0,0%	0,0%	0,0%	Bacteria	Bacteroidetes	Bacteroidia	Bacteroidales		Paludibacter	-	-	-	-	-	-	-	-	-	nifH	nifD	-	-	-	-	-	-	-	-	-	-	-	-	-	-
350105	0,0%	0,0%	0,0%	0,0%	0,0%	0,0%	0,0%	0,0%	0,0%	Bacteria	Proteobacteria	Gammaproteobacteria	Pseudomonadales	Pseudomonadaceae	Pseudomonas	cellulolytic	napA	narG	nirA	-	nirS	nirK	norB	nosZ	nifH	nifD	-	ureA	-	dsrB	SoxB	-	-	mmoX	-	laccase_Asco	laccase_Basidio	-	-	ppo
11278	0,0%	0,0%	0,0%	0,0%	0,0%	0,0%	0,0%	0,0%	0,0%	Bacteria	Gemmatimonadetes	Gemm-2			uncultured bacterium	-	-	-	-	-	-	-	-	-	-	-	-	-	-	-	-	-	-	-	-	-	-	-	-	-
6713	0,0%	0,0%	0,0%	0,0%	0,0%	0,1%	0,0%	0,0%	0,0%	Bacteria	Proteobacteria	Betaproteobacteria	Rhodocyclales	Rhodocyclaceae	uncultured	-	-	-	-	-	-	-	-	-	-	-	-	-	-	-	-	-	-	-	-	-	-	-	lip	-
4385959	0,0%	0,0%	0,0%	0,0%	0,0%	0,0%	0,0%	0,1%	0,0%	Bacteria	Proteobacteria	Deltaproteobacteria	Myxococcales		uncultured bacterium	-	-	-	-	-	-	-	-	-	-	-	-	-	-	-	-	-	-	-	-	-	-	-	-	-
4324483	0,0%	0,0%	0,0%	0,0%	0,0%	0,0%	0,0%	0,0%	0,0%	Bacteria	Firmicutes	Bacilli	Bacillales	Paenibacillaceae	Paenibacillus	-	napA	narG	-	-	nirS	nirK	norB	nosZ	nifH	nifD	-	ureA	-	-	-	-	-	-	-	laccase_Asco	laccase_Basidio	-	-	ppo
4430569	0,0%	0,0%	0,0%	0,0%	0,0%	0,0%	0,0%	0,0%	0,1%	Bacteria	Proteobacteria	Alphaproteobacteria	Rhizobiales	Hyphomicrobiaceae	Hyphomicrobium	-	napA	narG	nirA	-	-	nirK	norB	nosZ	nifH	nifD	-	ureA	-	-	-	-	-	mmoX	-	laccase_Asco	laccase_Basidio	-	-	ppo
4371286	0,0%	0,0%	0,0%	0,0%	0,0%	0,0%	0,0%	0,0%	0,0%	Bacteria	Chlorobi	BSV26	VC38		uncultured bacterium	-	-	-	-	-	-	-	-	-	-	-	-	-	-	-	-	-	-	-	-	-	-	-	-	-
228223	0,0%	0,0%	0,0%	0,0%	0,0%	0,0%	0,0%	0,0%	0,0%	Bacteria	Proteobacteria	Alphaproteobacteria	Rhodospirillales	Rhodospirillaceae	uncultured	-	-	-	-	-	-	-	-	-	-	-	-	-	-	-	-	-	-	-	-	-	-	-	lip	-
4406766	0,0%	0,0%	0,0%	0,0%	0,0%	0,0%	0,0%	0,0%	0,0%	Bacteria	Proteobacteria	Betaproteobacteria	Burkholderiales	Comamonadaceae	uncultured	-	-	-	-	-	-	-	-	-	-	-	-	-	-	-	-	-	-	-	-	-	-	-	lip	-
4043609	0,0%	0,0%	0,0%	0,0%	0,0%	0,0%	0,0%	0,0%	0,0%	Bacteria	Bacteroidetes	Flavobacteriia	Flavobacteriales	Cryomorphaceae	Fluviicola	-	-	-	nirA	-	-	-	norB	-	-	-	-	-	-	-	-	-	-	-	-	laccase_Asco	laccase_Basidio	-	-	ppo
4454737	0,0%	0,0%	0,0%	0,0%	0,0%	0,0%	0,0%	0,0%	0,0%	Bacteria	Firmicutes	Bacilli	Bacillales	Staphylococcaceae	Staphylococcus	-	napA	narG	-	-	-	nirK	norB	-	-	-	-	ureA	-	-	-	-	-	-	-	laccase_Asco	laccase_Basidio	-	-	-
4468850	0,0%	0,0%	0,0%	0,0%	0,0%	0,0%	0,0%	0,1%	0,0%	Bacteria	Actinobacteria	Actinobacteria	Actinomycetales	Microbacteriaceae	Rathayibacter	cellulolytic	napA	-	-	-	-	-	-	-	-	-	-	ureA	-	-	-	-	-	-	-	-	-	-	-	-
4372534	0,0%	0,0%	0,0%	0,0%	0,0%	0,0%	0,0%	0,0%	0,0%	Bacteria	Proteobacteria	Betaproteobacteria			uncultured bacterium	-	-	-	-	-	-	-	-	-	-	-	-	-	-	-	-	-	-	-	-	-	-	-	-	-
4327995	0,0%	0,0%	0,0%	0,0%	0,0%	0,0%	0,0%	0,0%	0,0%	Bacteria	Bacteroidetes	[Saprospirae]	[Saprospirales]	Chitinophagaceae	Ferruginibacter	-	-	-	-	-	-	-	-	-	-	-	-	-	-	-	-	-	-	-	-	-	-	-	-	-
817141	0,0%	0,0%	0,0%	0,0%	0,0%	0,0%	0,0%	0,0%	0,0%	Bacteria	Proteobacteria	Deltaproteobacteria	Bdellovibrionales	Bdellovibrionaceae	Bdellovibrio	-	-	-	-	-	-	nirK	norB	-	-	-	nrfA	-	-	-	-	-	-	-	-	laccase_Asco	laccase_Basidio	-	-	ppo
2399265	0,0%	0,0%	0,0%	0,0%	0,0%	0,0%	0,0%	0,0%	0,0%	Bacteria	Bacteroidetes	Cytophagia	Cytophagales	[Amoebophilaceae]	Candidatus Paenicardinium	-	-	-	-	-	-	-	-	-	-	-	-	-	-	-	-	-	-	-	-	-	-	-	-	-
1001333	0,0%	0,0%	0,0%	0,0%	0,0%	0,0%	0,0%	0,0%	0,0%	Bacteria	Acidobacteria	Holophagae	Holophagales	Holophagaceae	uncultured bacterium	-	-	-	-	-	-	-	-	-	-	-	-	-	-	-	-	-	-	-	-	-	-	-	-	-
4472813	0,0%	0,0%	0,0%	0,0%	0,0%	0,0%	0,0%	0,0%	0,0%	Bacteria	Proteobacteria	Betaproteobacteria	Burkholderiales	Comamonadaceae	Pelomonas	-	napA	narG	nirA	-	nirS	-	norB	nosZ	nifH	-	-	ureA	-	-	-	-	-	-	-	-	-	-	-	-
1120868	0,0%	0,0%	0,0%	0,0%	0,0%	0,0%	0,0%	0,0%	0,0%	Bacteria	Proteobacteria	Gammaproteobacteria	Methylococcales	Methylococcaceae	Methylomonas	-	napA	-	nirA	-	nirS	nirK	norB	-	nifH	nifD	-	ureA	-	-	-	amoA_AOB	-	mmoX	pmoA	laccase_Asco	laccase_Basidio	-	-	ppo
189407	0,0%	0,0%	0,0%	0,0%	0,0%	0,1%	0,0%	0,0%	0,0%	Bacteria	Firmicutes	Clostridia	Clostridiales	Peptostreptococcaceae	Incertae Sedis	-	-	-	-	-	-	-	-	-	-	-	-	-	-	-	-	-	-	-	-	-	-	-	-	-
1123812	0,0%	0,0%	0,0%	0,0%	0,0%	0,0%	0,0%	0,0%	0,0%	Bacteria	Planctomycetes	Planctomycetia	Gemmatales	Gemmataceae	Zavarzinella	-	-	-	-	-	-	-	-	-	-	-	-	-	-	-	-	-	-	-	-	-	-	-	-	-
1136827	0,0%	0,0%	0,0%	0,0%	0,0%	0,0%	0,0%	0,0%	0,0%	Bacteria	Planctomycetes	Planctomycetia	Planctomycetales	Planctomycetaceae	Schlesneria	-	-	-	-	-	-	-	-	-	-	-	-	-	-	-	-	-	-	-	-	-	-	-	-	-
515379	0,0%	0,0%	0,0%	0,0%	0,0%	0,1%	0,0%	0,0%	0,0%	Bacteria	Proteobacteria	Alphaproteobacteria	Rhodospirillales	Acetobacteraceae	Roseomonas	-	napA	-	-	-	-	-	-	-	-	nifD	-	ureA	-	-	-	-	-	-	-	-	laccase_Basidio	-	-	-
173557	0,0%	0,0%	0,0%	0,0%	0,0%	0,0%	0,0%	0,0%	0,0%	Bacteria	Proteobacteria	Alphaproteobacteria	Rickettsiales		uncultured	-	-	-	-	-	-	-	-	-	-	-	-	-	-	-	-	-	-	-	-	-	-	-	lip	-
355361	0,0%	0,0%	0,0%	0,0%	0,0%	0,0%	0,0%	0,0%	0,0%	Bacteria	Actinobacteria	Actinobacteria	Actinomycetales	Streptomycetaceae	Streptomyces	cellulolytic	napA	narG	nirA	-	-	-	norB	-	nifH	-	-	ureA	-	-	-	-	chb	mmoX	-	laccase_Asco	laccase_Basidio	-	-	ppo
4378898	0,0%	0,0%	0,0%	0,0%	0,0%	0,0%	0,0%	0,0%	0,0%	Bacteria	Proteobacteria	Alphaproteobacteria	Rhizobiales	Methylocystaceae	Methylosinus	-	napA	-	nirA	-	-	-	-	-	nifH	nifD	-	ureA	-	-	-	-	-	mmoX	pmoA	-	-	-	-	-
4475022	0,0%	0,0%	0,0%	0,0%	0,0%	0,0%	0,0%	0,0%	0,1%	Bacteria	Proteobacteria	Deltaproteobacteria	Myxococcales		uncultured bacterium	-	-	-	-	-	-	-	-	-	-	-	-	-	-	-	-	-	-	-	-	-	-	-	-	-
82334	0,0%	0,0%	0,0%	0,0%	0,0%	0,0%	0,0%	0,0%	0,0%	Bacteria	Proteobacteria	Deltaproteobacteria	Myxococcales		uncultured bacterium	-	-	-	-	-	-	-	-	-	-	-	-	-	-	-	-	-	-	-	-	-	-	-	-	-
4253236	0,0%	0,0%	0,0%	0,0%	0,0%	0,0%	0,0%	0,0%	0,0%	Bacteria	Cyanobacteria	Synechococcophycideae	Synechococcales	Synechococcaceae	Prochlorococcus	-	napA	-	nirA	-	-	-	-	-	nifH	nifD	-	ureA	-	-	-	-	-	-	-	-	-	-	-	-
4253237	0,0%	0,0%	0,0%	0,0%	0,0%	0,0%	0,0%	0,0%	0,0%	Bacteria	Verrucomicrobia	[Spartobacteria]	[Chthoniobacterales]	[Chthoniobacteraceae]	uncultured Verrucomicrobia bacterium	-	-	-	-	-	-	-	-	-	-	-	-	-	-	-	-	-	-	-	-	-	-	-	-	-
579465	0,0%	0,0%	0,0%	0,1%	0,0%	0,0%	0,0%	0,0%	0,0%	Bacteria	Proteobacteria	Alphaproteobacteria	Rhodobacterales	Rhodobacteraceae	Rhodobacter	-	napA	-	nirA	-	nirS	nirK	norB	nosZ	nifH	nifD	-	ureA	-	-	SoxB	-	-	mmoX	-	laccase_Asco	laccase_Basidio	-	-	ppo
2896033	0,0%	0,0%	0,0%	0,0%	0,0%	0,0%	0,0%	0,0%	0,0%	Bacteria	Proteobacteria	Alphaproteobacteria	Rhizobiales	Hyphomicrobiaceae	Hyphomicrobium	-	napA	narG	nirA	-	-	nirK	norB	nosZ	nifH	nifD	-	ureA	-	-	-	-	-	mmoX	-	laccase_Asco	laccase_Basidio	-	-	ppo
4379413	0,0%	0,0%	0,0%	0,0%	0,0%	0,0%	0,0%	0,0%	0,0%	Bacteria	Proteobacteria	Betaproteobacteria	Burkholderiales	Comamonadaceae	Aquincola	-	-	-	-	-	-	-	-	-	-	-	-	-	-	-	-	-	-	-	-	-	-	-	-	-
4425152	0,0%	0,0%	0,0%	0,0%	0,0%	0,0%	0,0%	0,0%	0,0%	Bacteria	Proteobacteria	Betaproteobacteria	Burkholderiales	Comamonadaceae	Ottowia	-	-	-	nirA	-	-	-	norB	nosZ	-	-	-	-	-	-	-	-	-	-	-	-	-	-	-	-
704060	0,0%	0,0%	0,0%	0,0%	0,0%	0,0%	0,0%	0,0%	0,0%	Bacteria	Bacteroidetes	Flavobacteriia	Flavobacteriales	Cryomorphaceae	uncultured bacterium	-	-	-	-	-	-	-	-	-	-	-	-	-	-	-	-	-	-	-	-	-	-	-	-	-
4369970	0,0%	0,0%	0,0%	0,0%	0,0%	0,0%	0,0%	0,0%	0,0%	Bacteria	Proteobacteria	Betaproteobacteria	Burkholderiales	Oxalobacteraceae	Undibacterium	-	-	-	-	-	-	-	-	-	-	-	-	-	-	-	-	-	-	-	-	-	-	-	-	-
679178	0,0%	0,0%	0,0%	0,0%	0,0%	0,0%	0,0%	0,0%	0,0%	Bacteria	Proteobacteria	Deltaproteobacteria	Myxococcales		uncultured bacterium	-	-	-	-	-	-	-	-	-	-	-	-	-	-	-	-	-	-	-	-	-	-	-	-	-
4441684	0,0%	0,0%	0,0%	0,0%	0,0%	0,0%	0,0%	0,0%	0,0%	Bacteria	Verrucomicrobia	Opitutae	Opitutales	Opitutaceae	Opitutus	-	napA	-	nirA	-	-	-	-	nosZ	-	-	nrfA	ureA	-	-	-	-	-	-	-	-	laccase_Basidio	-	-	-
4325519	0,0%	0,0%	0,0%	0,0%	0,0%	0,0%	0,0%	0,0%	0,0%	Bacteria	Actinobacteria	Actinobacteria	Actinomycetales	Geodermatophilaceae	Arthrobacter	-	napA	narG	-	-	-	-	-	-	nifH	-	-	ureA	dsrA	-	-	-	-	mmoX	-	laccase_Asco	laccase_Basidio	-	-	ppo
741284	0,0%	0,0%	0,0%	0,0%	0,0%	0,0%	0,0%	0,0%	0,0%	Bacteria	Proteobacteria	Betaproteobacteria	Rhodocyclales	Rhodocyclaceae	Zoogloea	-	-	-	-	-	-	-	-	-	nifH	-	-	-	-	-	-	-	-	-	-	-	-	-	-	-
130396	0,0%	0,0%	0,0%	0,0%	0,0%	0,0%	0,0%	0,0%	0,0%	Bacteria	Proteobacteria	Gammaproteobacteria	Methylococcales	Methylococcaceae	uncultured bacterium	-	-	-	-	-	-	-	-	-	-	-	-	-	-	-	-	-	-	-	-	-	-	-	-	-
250206	0,0%	0,0%	0,0%	0,0%	0,0%	0,0%	0,0%	0,0%	0,0%	Bacteria	Proteobacteria	Alphaproteobacteria	Rhizobiales	Rhizobiaceae	Rhizobium	-	napA	narG	nirA	-	-	nirK	norB	nosZ	nifH	nifD	nrfA	ureA	-	-	-	-	chb	mmoX	-	laccase_Asco	laccase_Basidio	-	-	ppo
4426764	0,0%	0,0%	0,0%	0,0%	0,0%	0,0%	0,0%	0,0%	0,0%	Bacteria	Bacteroidetes	Flavobacteriia	Flavobacteriales	Flavobacteriaceae	Flavobacterium	cellulolytic	napA	-	nirA	-	-	nirK	norB	nosZ	-	-	-	ureA	-	-	-	-	-	-	-	laccase_Asco	laccase_Basidio	-	-	ppo
4426763	0,0%	0,0%	0,0%	0,0%	0,0%	0,0%	0,0%	0,0%	0,0%	Bacteria	Proteobacteria	Betaproteobacteria	Burkholderiales	Comamonadaceae	uncultured	-	-	-	-	-	-	-	-	-	-	-	-	-	-	-	-	-	-	-	-	-	-	-	lip	-
104155	0,0%	0,0%	0,0%	0,0%	0,0%	0,0%	0,0%	0,0%	0,0%	Bacteria	Proteobacteria	Gammaproteobacteria	Xanthomonadales	Sinobacteraceae	Nevskia	-	-	-	-	-	-	-	-	-	-	-	-	-	-	-	-	-	-	-	-	-	-	-	-	-
141636	0,0%	0,0%	0,0%	0,0%	0,0%	0,0%	0,0%	0,0%	0,0%	Bacteria	Proteobacteria	Deltaproteobacteria	Bdellovibrionales	Bacteriovoracaceae	Bacteriovorax	-	-	-	-	-	-	-	norB	-	-	-	-	-	-	-	-	-	-	-	-	-	-	-	-	-
878161	0,0%	0,0%	0,0%	0,0%	0,0%	0,0%	0,0%	0,0%	0,0%	Bacteria	Acidobacteria	Acidobacteriia	Acidobacteriales	Acidobacteriaceae	Terriglobus	cellulolytic	napA	-	-	-	-	nirK	-	-	-	-	-	-	-	-	-	-	-	-	-	laccase_Asco	laccase_Basidio	-	-	ppo
4364714	0,0%	0,0%	0,0%	0,0%	0,0%	0,0%	0,0%	0,0%	0,3%	Bacteria	Proteobacteria	Betaproteobacteria	Ellin6067		uncultured	-	-	-	-	-	-	-	-	-	-	-	-	-	-	-	-	-	-	-	-	-	-	-	lip	-
4337631	0,0%	0,0%	0,0%	0,0%	0,0%	0,1%	0,0%	0,0%	0,0%	Bacteria	Proteobacteria	Alphaproteobacteria	Rhizobiales	Hyphomicrobiaceae	uncultured	-	-	-	-	-	-	-	-	-	-	-	-	-	-	-	-	-	-	-	-	-	-	-	lip	-
4471705	0,0%	0,0%	0,0%	0,0%	0,0%	0,0%	0,0%	0,0%	0,1%	Bacteria	Proteobacteria	Betaproteobacteria	Thiobacterales		uncultured	-	-	-	-	-	-	-	-	-	-	-	-	-	-	-	-	-	-	-	-	-	-	-	lip	-
4471706	0,0%	0,0%	0,0%	0,0%	0,0%	0,0%	0,0%	0,0%	0,0%	Bacteria	Proteobacteria	Betaproteobacteria	Burkholderiales	Comamonadaceae	uncultured	-	-	-	-	-	-	-	-	-	-	-	-	-	-	-	-	-	-	-	-	-	-	-	lip	-
551999	0,0%	0,0%	0,0%	0,0%	0,0%	0,0%	0,0%	0,0%	0,0%	Bacteria	Bacteroidetes	VC2_1_Bac22			uncultured bacterium	-	-	-	-	-	-	-	-	-	-	-	-	-	-	-	-	-	-	-	-	-	-	-	-	-
1127804	0,0%	0,0%	0,0%	0,0%	0,0%	0,0%	0,0%	0,0%	0,0%	Bacteria	Proteobacteria	Betaproteobacteria	Rhodocyclales	Rhodocyclaceae	uncultured	-	-	-	-	-	-	-	-	-	-	-	-	-	-	-	-	-	-	-	-	-	-	-	lip	-
843251	0,0%	0,0%	0,0%	0,0%	0,0%	0,0%	0,0%	0,0%	0,0%	Bacteria	Verrucomicrobia	[Pedosphaerae]	[Pedosphaerales]	R4-41B	uncultured bacterium	-	-	-	-	-	-	-	-	-	-	-	-	-	-	-	-	-	-	-	-	-	-	-	-	-
4391254	0,0%	0,0%	0,0%	0,0%	0,0%	0,0%	0,0%	0,0%	0,0%	Bacteria	Proteobacteria	Alphaproteobacteria	Rhizobiales		Bauldia	-	-	-	-	-	-	-	-	-	-	-	-	-	-	-	-	-	-	-	-	-	-	-	-	-
749563	0,0%	0,0%	0,0%	0,0%	0,0%	0,0%	0,0%	0,1%	0,0%	Bacteria	Actinobacteria	Actinobacteria	Actinomycetales	Microbacteriaceae	Pseudoclavibacter	cellulolytic	-	-	-	-	-	-	-	-	-	-	-	-	-	-	-	-	-	-	-	-	-	-	-	-
4430568	0,0%	0,0%	0,0%	0,0%	0,0%	0,0%	0,0%	0,0%	0,0%	Bacteria	Acidobacteria	Acidobacteriia	Acidobacteriales	Acidobacteriaceae	uncultured	cellulolytic	-	-	-	-	-	-	-	-	-	-	-	-	-	-	-	-	-	-	-	-	-	-	lip	-
222706	0,0%	0,0%	0,0%	0,0%	0,0%	0,0%	0,0%	0,0%	0,0%	Bacteria	Proteobacteria	Gammaproteobacteria	Enterobacteriales	Enterobacteriaceae	uncultured bacterium	cellulolytic	-	-	-	-	-	-	-	-	-	-	-	-	-	-	-	-	-	-	-	-	-	-	-	-
4444335	0,0%	0,0%	0,0%	0,0%	0,0%	0,0%	0,0%	0,0%	0,0%	Bacteria	Verrucomicrobia	Verrucomicrobiae	Verrucomicrobiales	Verrucomicrobiaceae	Prosthecobacter	-	-	-	-	-	-	-	-	-	-	-	-	-	-	-	-	-	-	-	-	-	-	-	-	-
1125242	0,0%	0,0%	0,0%	0,0%	0,0%	0,0%	0,0%	0,0%	0,0%	Bacteria	Proteobacteria	Betaproteobacteria	Burkholderiales	Oxalobacteraceae	Janthinobacterium	-	napA	narG	nirA	-	nirS	-	norB	nosZ	-	-	-	ureA	-	-	SoxB	-	chb	-	-	-	laccase_Basidio	-	-	-
217481	0,0%	0,0%	0,0%	0,0%	0,0%	0,0%	0,0%	0,0%	0,0%	Bacteria	Proteobacteria	Alphaproteobacteria	Rhodospirillales	Rhodospirillaceae	uncultured	-	-	-	-	-	-	-	-	-	-	-	-	-	-	-	-	-	-	-	-	-	-	-	lip	-
215053	0,0%	0,0%	0,0%	0,0%	0,0%	0,0%	0,0%	0,1%	0,0%	Bacteria	Proteobacteria	Alphaproteobacteria	Rhodospirillales	Acetobacteraceae	uncultured	-	-	-	-	-	-	-	-	-	-	-	-	-	-	-	-	-	-	-	-	-	-	-	lip	-
240252	0,0%	0,0%	0,0%	0,0%	0,0%	0,0%	0,0%	0,0%	0,0%	Bacteria	Proteobacteria	Alphaproteobacteria	Rhodospirillales	Acetobacteraceae	Acidocella	-	napA	narG	nirA	-	-	-	-	-	-	-	-	ureA	-	-	-	-	-	-	-	laccase_Asco	laccase_Basidio	-	-	ppo
305616	0,0%	0,0%	0,0%	0,0%	0,0%	0,0%	0,0%	0,0%	0,0%	Bacteria	Proteobacteria	Deltaproteobacteria	Myxococcales	Haliangiaceae	Haliangium	-	-	-	-	-	-	-	-	-	-	-	-	ureA	-	-	-	-	chb	-	-	-	-	-	-	-
816630	0,0%	0,0%	0,0%	0,0%	0,0%	0,0%	0,0%	0,0%	0,0%	Bacteria	Proteobacteria	Betaproteobacteria	Gallionellales	Gallionellaceae	Gallionella	-	-	-	nirA	-	-	-	norB	-	-	-	-	-	-	-	-	-	-	-	-	-	-	-	-	-
672252	0,0%	0,0%	0,0%	0,0%	0,0%	0,0%	0,0%	0,0%	0,0%	Bacteria	Acidobacteria	Acidobacteriia	Acidobacteriales	Acidobacteriaceae	Granulicella	cellulolytic	napA	-	-	-	-	-	-	-	-	-	-	-	-	-	-	-	-	-	-	laccase_Asco	laccase_Basidio	-	-	ppo
1107631	0,0%	0,0%	0,0%	0,0%	0,0%	0,0%	0,0%	0,0%	0,0%	Bacteria	Proteobacteria	Gammaproteobacteria	Xanthomonadales	Sinobacteraceae	uncultured bacterium	-	-	-	-	-	-	-	-	-	-	-	-	-	-	-	-	-	-	-	-	-	-	-	-	-
2058264	0,0%	0,0%	0,0%	0,0%	0,0%	0,0%	0,0%	0,0%	0,0%	Bacteria	Proteobacteria	Deltaproteobacteria	Myxococcales	OM27	OM27 clade	-	-	-	-	-	-	-	-	-	-	-	-	-	-	-	-	-	-	-	-	-	-	-	-	-
512811	0,0%	0,0%	0,0%	0,0%	0,0%	0,0%	0,0%	0,0%	0,0%	Bacteria	Acidobacteria	Acidobacteriia	Acidobacteriales	Acidobacteriaceae	Frigoribacterium	cellulolytic	napA	-	-	-	-	-	-	-	-	-	-	-	-	-	-	-	-	-	-	-	-	-	-	-
4440449	0,0%	0,0%	0,0%	0,0%	0,0%	0,0%	0,0%	0,0%	0,0%	Bacteria	Proteobacteria	Alphaproteobacteria	Rhizobiales	Rhizobiaceae	Rhizobium	-	napA	narG	nirA	-	-	nirK	norB	nosZ	nifH	nifD	nrfA	ureA	-	-	-	-	chb	mmoX	-	laccase_Asco	laccase_Basidio	-	-	ppo
104795	0,0%	0,0%	0,0%	0,0%	0,0%	0,0%	0,0%	0,0%	0,0%	Bacteria	Verrucomicrobia	Verrucomicrobiae	Verrucomicrobiales	Verrucomicrobiaceae	Luteolibacter	-	-	-	-	-	-	-	-	-	-	-	-	-	-	-	-	-	-	-	-	-	-	-	-	-
184685	0,0%	0,0%	0,0%	0,0%	0,0%	0,0%	0,0%	0,0%	0,0%	Bacteria	Bacteroidetes	[Saprospirae]	[Saprospirales]	Saprospiraceae	Haliscomenobacter	-	napA	-	nirA	-	-	-	norB	nosZ	-	-	-	-	-	-	-	-	-	-	-	laccase_Asco	laccase_Basidio	-	-	ppo
806708	0,0%	0,0%	0,0%	0,0%	0,0%	0,0%	0,0%	0,0%	0,0%	Bacteria	Proteobacteria	Alphaproteobacteria	Rhodobacterales	Rhodobacteraceae	Roseobacter	-	napA	narG	-	-	nirS	nirK	norB	nosZ	nifH	-	-	ureA	-	-	SoxB	-	-	-	-	laccase_Asco	laccase_Basidio	-	-	ppo
125742	0,0%	0,0%	0,0%	0,0%	0,0%	0,0%	0,0%	0,0%	0,0%	Bacteria	Proteobacteria	Betaproteobacteria	Methylophilales	Methylophilaceae	uncultured	-	-	-	-	-	-	-	-	-	-	-	-	-	-	-	-	-	-	-	-	-	-	-	lip	-
4457829	0,0%	0,0%	0,0%	0,0%	0,0%	0,0%	0,0%	0,0%	0,0%	Bacteria	Proteobacteria	Betaproteobacteria	Burkholderiales	Oxalobacteraceae	Massilia	-	napA	narG	-	-	-	-	norB	nosZ	-	-	-	ureA	-	-	-	-	chb	mmoX	-	laccase_Asco	laccase_Basidio	-	-	ppo
99135	0,0%	0,0%	0,0%	0,0%	0,0%	0,0%	0,0%	0,0%	0,0%	Bacteria	Proteobacteria	Alphaproteobacteria	Rhizobiales	Rhizobiaceae	Rhizobium	-	napA	narG	nirA	-	-	nirK	norB	nosZ	nifH	nifD	nrfA	ureA	-	-	-	-	chb	mmoX	-	laccase_Asco	laccase_Basidio	-	-	ppo
128033	0,0%	0,0%	0,0%	0,0%	0,0%	0,0%	0,0%	0,0%	0,0%	Bacteria	Proteobacteria	Alphaproteobacteria	Rhizobiales	Rhizobiaceae	Ochrobactrum	-	napA	narG	nirA	-	-	nirK	norB	nosZ	nifH	-	-	ureA	-	-	-	-	-	-	-	laccase_Asco	laccase_Basidio	-	-	ppo
2848150	0,0%	0,0%	0,0%	0,0%	0,0%	0,0%	0,0%	0,0%	0,0%	Bacteria	Proteobacteria	Betaproteobacteria	Burkholderiales	Comamonadaceae	uncultured	-	-	-	-	-	-	-	-	-	-	-	-	-	-	-	-	-	-	-	-	-	-	-	lip	-
4395040	0,0%	0,0%	0,0%	0,0%	0,0%	0,0%	0,0%	0,0%	0,0%	Bacteria	Acidobacteria	Solibacteres	Solibacterales	[Bryobacteraceae]	uncultured Acidobacteria bacterium	-	-	-	-	-	-	-	-	-	-	-	-	-	-	-	-	-	-	-	-	-	-	-	-	-
4482432	0,0%	0,0%	0,0%	0,0%	0,0%	0,0%	0,0%	0,0%	0,0%	Bacteria	Proteobacteria	Alphaproteobacteria	Rhodobacterales	Rhodobacteraceae	uncultured	-	-	-	-	-	-	-	-	-	-	-	-	-	-	-	-	-	-	-	-	-	-	-	lip	-
2969888	0,0%	0,0%	0,0%	0,0%	0,0%	0,0%	0,0%	0,0%	0,0%	Bacteria	Proteobacteria	Betaproteobacteria	Burkholderiales	Alcaligenaceae	uncultured	cellulolytic	-	-	-	-	-	-	-	-	-	-	-	-	-	-	-	-	-	-	-	-	-	-	lip	-
764906	0,0%	0,0%	0,0%	0,1%	0,0%	0,0%	0,0%	0,0%	0,0%	Bacteria	Proteobacteria	Betaproteobacteria	Burkholderiales	Comamonadaceae	uncultured	-	-	-	-	-	-	-	-	-	-	-	-	-	-	-	-	-	-	-	-	-	-	-	lip	-
4476547	0,0%	0,0%	0,0%	0,0%	0,0%	0,1%	0,0%	0,0%	0,0%	Bacteria	Proteobacteria	Betaproteobacteria	Burkholderiales	Oxalobacteraceae	Undibacterium	-	-	-	-	-	-	-	-	-	-	-	-	-	-	-	-	-	-	-	-	-	-	-	-	-
810672	0,0%	0,0%	0,0%	0,0%	0,0%	0,0%	0,0%	0,0%	0,0%	Bacteria	Actinobacteria	Actinobacteria	Actinomycetales	Microbacteriaceae	Candidatus Rhodoluna	cellulolytic	-	-	-	-	-	-	-	-	-	-	-	-	-	-	-	-	-	-	-	-	-	-	-	-
1111937	0,0%	0,0%	0,0%	0,0%	0,0%	0,0%	0,0%	0,0%	0,0%	Bacteria	Proteobacteria	Alphaproteobacteria	Rhizobiales		uncultured Phyllobacteriaceae bacterium	-	-	-	-	-	-	-	-	-	-	-	-	-	-	-	-	-	-	-	-	-	-	-	-	-
4470872	0,0%	0,0%	0,0%	0,0%	0,0%	0,1%	0,0%	0,0%	0,0%	Bacteria	Proteobacteria	Betaproteobacteria	Burkholderiales	Comamonadaceae	Variovorax	-	napA	narG	nirA	-	-	-	norB	-	-	-	-	ureA	-	-	-	-	-	mmoX	-	-	-	-	-	-
4457555	0,0%	0,0%	0,0%	0,0%	0,0%	0,0%	0,0%	0,1%	0,0%	Bacteria	Proteobacteria	Alphaproteobacteria	Sphingomonadales	Sphingomonadaceae	Altererythrobacter	-	napA	narG	-	-	-	-	-	-	-	-	-	-	-	-	-	-	-	-	-	laccase_Asco	laccase_Basidio	-	-	ppo

4470874	0,0%	0,0%	0,0%	0,0%	0,0%	0,0%	0,0%	0,1%	0,0%	Bacteria	Proteobacteria	Betaproteobacteria	Rhodocyclales	Rhodocyclaceae	Zoogloea	-	-	-	-	-	-	-	-	-	nifH	-	-	-	-	-	-	-	-	-	-	-	-	-	-	-
794581	0,0%	0,0%	0,0%	0,0%	0,0%	0,0%	0,0%	0,0%	0,0%	Bacteria	Proteobacteria	Gammaproteobacteria	Legionellales	Coxiellaceae	Aquicella	-	-	-	-	-	-	-	-	-	-	-	-	-	-	-	-	-	-	-	-	-	-	-	-	-
4399692	0,0%	0,0%	0,0%	0,0%	0,0%	0,0%	0,0%	0,0%	0,0%	Bacteria	Proteobacteria	Gammaproteobacteria	Legionellales	Legionellaceae	Legionella	-	-	-	-	-	nirS	-	norB	-	-	-	-	-	-	-	-	-	-	-	-	laccase_Asco	laccase_Basidio	-	-	ppo
4371434	0,0%	0,0%	0,0%	0,0%	0,0%	0,0%	0,0%	0,0%	0,1%	Bacteria	Actinobacteria	Actinobacteria	Actinomycetales	Streptomycetaceae	Streptomyces	cellulolytic	napA	narG	nirA	-	-	-	norB	-	nifH	-	-	ureA	-	-	-	-	chb	mmoX	-	laccase_Asco	laccase_Basidio	-	-	ppo
1107985	0,0%	0,0%	0,0%	0,0%	0,0%	0,0%	0,0%	0,0%	0,0%	Bacteria	Proteobacteria	Deltaproteobacteria	Myxococcales		uncultured delta proteobacterium	-	-	-	-	-	-	-	-	-	-	-	-	-	-	-	-	-	-	-	-	-	-	-	-	-
894244	0,0%	0,0%	0,0%	0,0%	0,0%	0,0%	0,0%	0,0%	0,0%	Bacteria	Proteobacteria	Deltaproteobacteria	MIZ46		uncultured bacterium	-	-	-	-	-	-	-	-	-	-	-	-	-	-	-	-	-	-	-	-	-	-	-	-	-
4310042	0,0%	0,0%	0,0%	0,0%	0,0%	0,0%	0,0%	0,0%	0,0%	Bacteria	Planctomycetes	Planctomycetia	Gemmatales	Gemmataceae	Gemmata	-	-	-	-	-	-	-	-	-	-	-	-	-	-	-	-	-	-	-	-	-	-	-	-	-
1137888	0,0%	0,0%	0,0%	0,0%	0,0%	0,0%	0,0%	0,0%	0,0%	Bacteria	Bacteroidetes	Cytophagia	Cytophagales	Cytophagaceae	uncultured	cellulolytic	-	-	-	-	-	-	-	-	-	-	-	-	-	-	-	-	-	-	-	-	-	-	lip	-
247981	0,0%	0,0%	0,0%	0,0%	0,0%	0,0%	0,0%	0,0%	0,0%	Bacteria	Proteobacteria	Deltaproteobacteria	Bdellovibrionales	Bdellovibrionaceae	Bdellovibrio	-	-	-	-	-	-	nirK	norB	-	-	-	nrfA	-	-	-	-	-	-	-	-	laccase_Asco	laccase_Basidio	-	-	ppo
4426381	0,0%	0,0%	0,0%	0,0%	0,0%	0,0%	0,0%	0,0%	0,0%	Bacteria	Proteobacteria	Alphaproteobacteria	Rhodobacterales	Rhodobacteraceae	Leisingera	-	napA	-	-	-	-	-	norB	nosZ	-	-	-	ureA	-	-	-	-	-	-	-	-	-	-	-	ppo
4327906	0,0%	0,0%	0,0%	0,0%	0,0%	0,0%	0,0%	0,0%	0,0%	Bacteria	Bacteroidetes	Sphingobacteriia	Sphingobacteriales	Sphingobacteriaceae	Mucilaginibacter	-	-	-	-	-	-	-	-	-	-	-	-	-	-	-	-	-	-	-	-	-	-	-	-	-
169755	0,0%	0,0%	0,0%	0,0%	0,0%	0,0%	0,0%	0,0%	0,0%	Bacteria	Proteobacteria	Alphaproteobacteria	Rhodospirillales	Acetobacteraceae	Roseococcus	-	-	-	-	-	-	-	-	-	-	-	-	-	-	-	-	-	-	-	-	-	-	-	-	-
4363537	0,0%	0,0%	0,0%	0,0%	0,0%	0,0%	0,0%	0,0%	0,0%	Bacteria	Proteobacteria	Betaproteobacteria	Burkholderiales	Oxalobacteraceae	Herbaspirillum	-	napA	narG	-	-	nirS	nirK	norB	nosZ	nifH	nifD	-	ureA	-	-	-	-	-	-	-	-	laccase_Basidio	-	-	-
190663	0,0%	0,0%	0,0%	0,0%	0,0%	0,0%	0,0%	0,0%	0,0%	Bacteria	Actinobacteria	Actinobacteria	Actinomycetales	Kineosporiaceae	Kineosporia	-	-	-	-	-	-	-	-	-	-	-	-	-	-	-	-	-	-	-	-	-	-	-	-	-
781550	0,0%	0,0%	0,0%	0,0%	0,0%	0,0%	0,0%	0,0%	0,0%	Bacteria	Proteobacteria	Alphaproteobacteria	Sphingomonadales	Sphingomonadaceae	Sphingosinicella sp. YJ22	-	-	-	-	-	-	-	-	-	-	-	-	-	-	-	-	-	-	-	-	-	-	-	-	-
1144864	0,0%	0,0%	0,0%	0,0%	0,0%	0,0%	0,0%	0,0%	0,0%	Bacteria	Proteobacteria	Gammaproteobacteria	Alteromonadales		BD1-7 clade	-	-	-	-	-	-	-	-	-	-	-	-	-	-	-	-	-	-	-	-	-	-	-	-	-
1110219	0,0%	0,0%	0,0%	0,0%	0,0%	0,0%	0,0%	0,0%	0,0%	Bacteria	Chloroflexi	Chloroflexi	AKIW781		uncultured bacterium	-	-	-	-	-	-	-	-	-	-	-	-	-	-	-	-	-	-	-	-	-	-	-	-	-
4315319	0,0%	0,0%	0,0%	0,0%	0,0%	0,0%	0,0%	0,0%	0,0%	Bacteria	Proteobacteria	Betaproteobacteria	Neisseriales	Neisseriaceae	Deefgea	-	-	-	-	-	-	-	-	-	-	-	-	-	-	-	-	-	-	-	-	-	-	-	-	-
575999	0,0%	0,0%	0,0%	0,0%	0,0%	0,0%	0,0%	0,0%	0,0%	Bacteria	Acidobacteria	Solibacteres	Solibacterales		uncultured prokaryote	-	-	-	-	-	-	-	-	-	-	-	-	-	-	-	-	-	-	-	-	-	-	-	-	-
689696	0,0%	0,0%	0,0%	0,0%	0,0%	0,0%	0,0%	0,0%	0,0%	Bacteria	Proteobacteria	Betaproteobacteria	Burkholderiales	Comamonadaceae	uncultured	-	-	-	-	-	-	-	-	-	-	-	-	-	-	-	-	-	-	-	-	-	-	-	lip	-
3025055	0,0%	0,0%	0,0%	0,0%	0,0%	0,0%	0,0%	0,0%	0,0%	Bacteria	Bacteroidetes	[Saprospirae]	[Saprospirales]		uncultured	-	-	-	-	-	-	-	-	-	-	-	-	-	-	-	-	-	-	-	-	-	-	-	lip	-
1128107	0,0%	0,0%	0,0%	0,0%	0,0%	0,0%	0,0%	0,0%	0,0%	Bacteria	WS3	PRR-12	Sediment-1	PRR-10	uncultured Latescibacteria bacterium	-	-	-	-	-	-	-	-	-	-	-	-	-	-	-	-	-	-	-	-	-	-	-	-	-
202787	0,0%	0,0%	0,0%	0,0%	0,0%	0,0%	0,0%	0,0%	0,0%	Bacteria	Planctomycetes	Planctomycetia	Gemmatales	Gemmataceae	uncultured	-	-	-	-	-	-	-	-	-	-	-	-	-	-	-	-	-	-	-	-	-	-	-	lip	-
4419795	0,0%	0,0%	0,0%	0,0%	0,0%	0,0%	0,0%	0,0%	0,0%	Bacteria	Proteobacteria	Deltaproteobacteria			uncultured bacterium	-	-	-	-	-	-	-	-	-	-	-	-	-	-	-	-	-	-	-	-	-	-	-	-	-
4469492	0,0%	0,0%	0,0%	0,0%	0,0%	0,0%	0,0%	0,0%	0,0%	Bacteria	Proteobacteria	Betaproteobacteria	Burkholderiales	Comamonadaceae	Pelomonas	-	napA	narG	nirA	-	nirS	-	norB	nosZ	nifH	-	-	ureA	-	-	-	-	-	-	-	-	-	-	-	-
2565173	0,0%	0,0%	0,0%	0,0%	0,0%	0,0%	0,0%	0,0%	0,0%	Bacteria	Verrucomicrobia	Opitutae	Opitutales	Opitutaceae	Opitutus	-	napA	-	nirA	-	-	-	-	nosZ	-	-	nrfA	ureA	-	-	-	-	-	-	-	-	laccase_Basidio	-	-	-
204372	0,0%	0,0%	0,0%	0,0%	0,0%	0,0%	0,0%	0,1%	0,0%	Bacteria	Proteobacteria	Deltaproteobacteria	Myxococcales		Haliangium	-	-	-	-	-	-	-	-	-	-	-	-	ureA	-	-	-	-	chb	-	-	-	-	-	-	-
1042160	0,0%	0,0%	0,0%	0,0%	0,0%	0,0%	0,0%	0,0%	0,0%	Bacteria	Actinobacteria	Thermoleophilia	Solirubrobacterales	Patulibacteraceae	Erythrobacter	-	napA	narG	nirA	-	-	-	-	-	nifH	-	-	-	-	-	-	-	-	-	-	laccase_Asco	laccase_Basidio	-	-	ppo
534216	0,0%	0,0%	0,0%	0,0%	0,0%	0,0%	0,0%	0,0%	0,0%	Bacteria	Proteobacteria	Betaproteobacteria	Burkholderiales	Comamonadaceae	uncultured	-	-	-	-	-	-	-	-	-	-	-	-	-	-	-	-	-	-	-	-	-	-	-	lip	-
33768	0,0%	0,0%	0,0%	0,0%	0,0%	0,0%	0,0%	0,0%	0,0%	Bacteria	Planctomycetes	Planctomycetia	Gemmatales	Isosphaeraceae	uncultured bacterium	-	-	-	-	-	-	-	-	-	-	-	-	-	-	-	-	-	-	-	-	-	-	-	-	-
564587	0,0%	0,0%	0,0%	0,0%	0,0%	0,0%	0,0%	0,0%	0,0%	Bacteria	Proteobacteria	Alphaproteobacteria	Rhodospirillales	Acetobacteraceae	Roseomonas	-	napA	-	-	-	-	-	-	-	-	nifD	-	ureA	-	-	-	-	-	-	-	-	laccase_Basidio	-	-	-
137007	0,0%	0,0%	0,0%	0,0%	0,0%	0,0%	0,0%	0,0%	0,1%	Bacteria	Proteobacteria	Betaproteobacteria	Burkholderiales	Comamonadaceae	Limnohabitans	-	napA	narG	-	-	-	-	-	-	-	-	-	ureA	-	-	-	-	-	-	-	-	-	-	-	-
4448407	0,0%	0,0%	0,0%	0,0%	0,0%	0,0%	0,0%	0,0%	0,0%	Bacteria	Proteobacteria	Betaproteobacteria	Burkholderiales	Burkholderiaceae	Burkholderia	-	napA	narG	nirA	-	nirS	nirK	norB	nosZ	nifH	nifD	-	ureA	-	-	-	-	chb	mmoX	-	laccase_Asco	laccase_Basidio	-	-	ppo
113244	0,0%	0,0%	0,0%	0,0%	0,0%	0,0%	0,0%	0,0%	0,0%	Bacteria	Proteobacteria	Deltaproteobacteria	Myxococcales		uncultured bacterium	-	-	-	-	-	-	-	-	-	-	-	-	-	-	-	-	-	-	-	-	-	-	-	-	-
1017063	0,0%	0,0%	0,0%	0,0%	0,0%	0,0%	0,0%	0,0%	0,0%	Bacteria	Proteobacteria	Deltaproteobacteria	Myxococcales	Polyangiaceae	Byssovorax	-	-	-	-	-	-	-	-	-	-	-	-	-	-	-	-	-	-	-	-	-	-	-	-	-
847644	0,0%	0,0%	0,0%	0,0%	0,0%	0,0%	0,0%	0,0%	0,0%	Bacteria	Planctomycetes	Planctomycetia	Gemmatales	Isosphaeraceae	uncultured	-	-	-	-	-	-	-	-	-	-	-	-	-	-	-	-	-	-	-	-	-	-	-	lip	-
4434444	0,0%	0,0%	0,0%	0,0%	0,0%	0,0%	0,0%	0,0%	0,0%	Bacteria	Proteobacteria	Alphaproteobacteria	Rhizobiales	Bradyrhizobiaceae	Bradyrhizobium	-	napA	narG	nirA	-	nirS	nirK	norB	nosZ	nifH	nifD	-	ureA	-	-	SoxB	-	-	mmoX	-	laccase_Asco	laccase_Basidio	-	-	ppo
4479555	0,0%	0,0%	0,0%	0,0%	0,0%	0,0%	0,0%	0,0%	0,0%	Bacteria	Proteobacteria	Alphaproteobacteria	Rhodospirillales	Acetobacteraceae	Rhodovarius	-	-	-	-	-	-	-	-	-	-	-	-	-	-	-	-	-	-	-	-	-	-	-	-	-
4389260	0,0%	0,0%	0,0%	0,0%	0,0%	0,0%	0,0%	0,0%	0,0%	Bacteria	Planctomycetes	Planctomycetia	Gemmatales	Gemmataceae	Gemmata	-	-	-	-	-	-	-	-	-	-	-	-	-	-	-	-	-	-	-	-	-	-	-	-	-
364646	0,0%	0,0%	0,0%	0,0%	0,0%	0,0%	0,0%	0,0%	0,0%	Bacteria	Proteobacteria	Deltaproteobacteria	Myxococcales		uncultured bacterium	-	-	-	-	-	-	-	-	-	-	-	-	-	-	-	-	-	-	-	-	-	-	-	-	-
4417996	0,0%	0,0%	0,0%	0,0%	0,0%	0,0%	0,0%	0,0%	0,0%	Bacteria	Proteobacteria	Betaproteobacteria	Burkholderiales	Comamonadaceae	Leptothrix	-	napA	-	-	-	nirS	-	norB	nosZ	nifH	nifD	-	ureA	-	-	-	-	-	mmoX	-	-	-	-	-	-
827054	0,0%	0,0%	0,0%	0,0%	0,0%	0,0%	0,0%	0,0%	0,0%	Bacteria	Elusimicrobia	Elusimicrobia	Elusimicrobiales		uncultured bacterium	-	-	-	-	-	-	-	-	-	-	-	-	-	-	-	-	-	-	-	-	-	-	-	-	-
1088405	0,0%	0,0%	0,0%	0,0%	0,0%	0,0%	0,0%	0,0%	0,1%	Bacteria	Proteobacteria	Gammaproteobacteria	Alteromonadales		BD1-7 clade	-	-	-	-	-	-	-	-	-	-	-	-	-	-	-	-	-	-	-	-	-	-	-	-	-
805496	0,0%	0,0%	0,0%	0,0%	0,0%	0,0%	0,0%	0,0%	0,0%	Bacteria	Cyanobacteria	Oscillatoriophycideae	Oscillatoriales	Phormidiaceae	Microcoleus	-	napA	-	nirA	-	-	-	-	-	nifH	nifD	-	ureA	-	-	-	-	-	-	-	-	-	-	-	-
718367	0,0%	0,0%	0,0%	0,0%	0,0%	0,0%	0,0%	0,0%	0,0%	Bacteria	Bacteroidetes	Sphingobacteriia	Sphingobacteriales	Sphingobacteriaceae	Mucilaginibacter	-	-	-	-	-	-	-	-	-	-	-	-	-	-	-	-	-	-	-	-	-	-	-	-	-
226704	0,0%	0,0%	0,0%	0,0%	0,0%	0,0%	0,0%	0,0%	0,0%	Bacteria	Proteobacteria	Deltaproteobacteria	Myxococcales		Sorangium	-	napA	-	-	-	-	nirK	norB	-	-	-	nrfA	ureA	-	-	-	-	chb	-	-	laccase_Asco	laccase_Basidio	-	-	ppo
529444	0,0%	0,0%	0,0%	0,0%	0,0%	0,0%	0,0%	0,0%	0,0%	Bacteria	Actinobacteria	Actinobacteria	Actinomycetales	Microbacteriaceae	Salinibacterium	cellulolytic	-	-	-	-	-	-	-	-	-	-	-	-	-	-	-	-	-	-	-	-	-	-	-	-
3701020	0,0%	0,0%	0,0%	0,0%	0,0%	0,0%	0,0%	0,0%	0,0%	Bacteria	Verrucomicrobia	[Pedosphaerae]	[Pedosphaerales]		uncultured Verrucomicrobia bacterium	-	-	-	-	-	-	-	-	-	-	-	-	-	-	-	-	-	-	-	-	-	-	-	-	-
575419	0,0%	0,0%	0,0%	0,1%	0,0%	0,0%	0,0%	0,0%	0,0%	Bacteria	Proteobacteria	Alphaproteobacteria	Rhizobiales	Hyphomicrobiaceae	Devosia	-	napA	-	-	-	-	nirK	-	-	nifH	-	-	ureA	-	-	-	-	chb	-	-	laccase_Asco	laccase_Basidio	-	-	ppo
756819	0,0%	0,0%	0,0%	0,0%	0,0%	0,0%	0,0%	0,0%	0,0%	Bacteria	Proteobacteria	Gammaproteobacteria	Aeromonadales	Aeromonadaceae	uncultured bacterium	-	-	-	-	-	-	-	-	-	-	-	-	-	-	-	-	-	-	-	-	-	-	-	-	-
636827	0,0%	0,0%	0,0%	0,0%	0,0%	0,0%	0,0%	0,0%	0,0%	Bacteria	Firmicutes	Bacilli	Bacillales	Paenibacillaceae	Paenibacillus	-	napA	narG	-	-	nirS	nirK	norB	nosZ	nifH	nifD	-	ureA	-	-	-	-	-	-	-	laccase_Asco	laccase_Basidio	-	-	ppo
40073	0,0%	0,0%	0,0%	0,0%	0,0%	0,0%	0,0%	0,0%	0,0%	Bacteria	Bacteroidetes	[Saprospirae]	[Saprospirales]	Chitinophagaceae	Sediminibacterium	-	-	-	-	-	-	-	-	-	-	-	-	-	-	-	-	-	-	-	-	-	-	-	-	-
4422628	0,0%	0,0%	0,0%	0,0%	0,0%	0,0%	0,0%	0,0%	0,0%	Bacteria	Proteobacteria	Alphaproteobacteria	Rhizobiales		uncultured	-	-	-	-	-	-	-	-	-	-	-	-	-	-	-	-	-	-	-	-	-	-	-	lip	-
2116632	0,0%	0,0%	0,0%	0,0%	0,0%	0,0%	0,0%	0,0%	0,0%	Bacteria	Acidobacteria	Holophagae	Holophagales	Holophagaceae	uncultured	-	-	-	-	-	-	-	-	-	-	-	-	-	-	-	-	-	-	-	-	-	-	-	lip	-
834065	0,0%	0,0%	0,0%	0,0%	0,0%	0,0%	0,0%	0,0%	0,0%	Bacteria	Proteobacteria	Gammaproteobacteria	Aeromonadales	Aeromonadaceae	uncultured bacterium	-	-	-	-	-	-	-	-	-	-	-	-	-	-	-	-	-	-	-	-	-	-	-	-	-
4304056	0,0%	0,0%	0,0%	0,0%	0,0%	0,0%	0,0%	0,0%	0,0%	Bacteria	Proteobacteria	Alphaproteobacteria	Rhizobiales	Rhizobiaceae	Rhizobium	-	napA	narG	nirA	-	-	nirK	norB	nosZ	nifH	nifD	nrfA	ureA	-	-	-	-	chb	mmoX	-	laccase_Asco	laccase_Basidio	-	-	ppo
3222178	0,0%	0,0%	0,0%	0,0%	0,0%	0,0%	0,0%	0,0%	0,0%	Bacteria	Proteobacteria	Deltaproteobacteria	MIZ46		uncultured bacterium	-	-	-	-	-	-	-	-	-	-	-	-	-	-	-	-	-	-	-	-	-	-	-	-	-
4466432	0,0%	0,0%	0,0%	0,0%	0,0%	0,0%	0,0%	0,0%	0,0%	Bacteria	Proteobacteria	Alphaproteobacteria	Rhizobiales	Rhizobiaceae	Rhizobium	-	napA	narG	nirA	-	-	nirK	norB	nosZ	nifH	nifD	nrfA	ureA	-	-	-	-	chb	mmoX	-	laccase_Asco	laccase_Basidio	-	-	ppo
4416113	0,0%	0,0%	0,0%	0,0%	0,0%	0,0%	0,0%	0,0%	0,1%	Bacteria	Proteobacteria	Gammaproteobacteria	Enterobacteriales	Enterobacteriaceae	Escherichia-Shigella	cellulolytic	-	-	-	-	-	-	-	-	-	-	-	-	-	-	-	-	-	-	-	-	-	-	-	-
1124975	0,0%	0,0%	0,0%	0,0%	0,0%	0,0%	0,0%	0,0%	0,0%	Bacteria	Proteobacteria	Deltaproteobacteria	Myxococcales		Haliangium	-	-	-	-	-	-	-	-	-	-	-	-	ureA	-	-	-	-	chb	-	-	-	-	-	-	-
4452700	0,0%	0,0%	0,0%	0,0%	0,0%	0,0%	0,0%	0,0%	0,0%	Bacteria	Firmicutes	Clostridia	Clostridiales	Clostridiaceae	Clostridium sensu stricto 5	cellulolytic	-	-	-	-	-	-	-	-	-	-	-	-	-	-	-	-	-	-	-	-	-	-	-	-
2471299	0,0%	0,0%	0,0%	0,0%	0,0%	0,0%	0,0%	0,0%	0,0%	Bacteria	Proteobacteria	Alphaproteobacteria	Rhodospirillales	Rhodospirillaceae	uncultured	-	-	-	-	-	-	-	-	-	-	-	-	-	-	-	-	-	-	-	-	-	-	-	lip	-
584177	0,0%	0,0%	0,0%	0,0%	0,0%	0,0%	0,0%	0,0%	0,0%	Bacteria	Proteobacteria	Betaproteobacteria	Burkholderiales	Comamonadaceae	uncultured	-	-	-	-	-	-	-	-	-	-	-	-	-	-	-	-	-	-	-	-	-	-	-	lip	-
4457268	0,0%	0,0%	0,0%	0,0%	0,0%	0,0%	0,0%	0,0%	0,0%	Bacteria	Proteobacteria	Gammaproteobacteria	Enterobacteriales	Enterobacteriaceae	Cronobacter	cellulolytic	napA	narG	-	-	-	-	-	-	-	-	-	-	-	-	-	-	chb	-	-	laccase_Asco	laccase_Basidio	-	-	ppo
4418684	0,0%	0,0%	0,0%	0,0%	0,0%	0,0%	0,0%	0,0%	0,0%	Bacteria	Proteobacteria	Alphaproteobacteria	Rhizobiales	Methylocystaceae	uncultured organism	-	-	-	-	-	-	-	-	-	-	-	-	-	-	-	-	-	-	-	-	-	-	-	-	-
2471295	0,0%	0,0%	0,0%	0,0%	0,0%	0,0%	0,0%	0,0%	0,0%	Bacteria	Proteobacteria	Alphaproteobacteria			uncultured	-	-	-	-	-	-	-	-	-	-	-	-	-	-	-	-	-	-	-	-	-	-	-	lip	-
4472969	0,0%	0,0%	0,0%	0,0%	0,0%	0,0%	0,0%	0,0%	0,0%	Bacteria	Proteobacteria	Deltaproteobacteria	Myxococcales		Sorangium	-	napA	-	-	-	-	nirK	norB	-	-	-	nrfA	ureA	-	-	-	-	chb	-	-	laccase_Asco	laccase_Basidio	-	-	ppo
676387	0,0%	0,0%	0,0%	0,0%	0,0%	0,0%	0,0%	0,0%	0,0%	Bacteria	Proteobacteria	Alphaproteobacteria	Rhizobiales	Methylocystaceae	uncultured	-	-	-	-	-	-	-	-	-	-	-	-	-	-	-	-	-	-	-	-	-	-	-	lip	-
309755	0,0%	0,0%	0,0%	0,0%	0,0%	0,1%	0,0%	0,0%	0,0%	Bacteria	Proteobacteria	Alphaproteobacteria	Rhodospirillales	Acetobacteraceae	Roseomonas	-	napA	-	-	-	-	-	-	-	-	nifD	-	ureA	-	-	-	-	-	-	-	-	laccase_Basidio	-	-	-
3310211	0,0%	0,0%	0,0%	0,0%	0,0%	0,0%	0,0%	0,1%	0,0%	Bacteria	Bacteroidetes	[Saprospirae]	[Saprospirales]	Chitinophagaceae	Sediminibacterium	-	-	-	-	-	-	-	-	-	-	-	-	-	-	-	-	-	-	-	-	-	-	-	-	-
4477199	0,0%	0,0%	0,0%	0,0%	0,0%	0,1%	0,0%	0,0%	0,0%	Bacteria	Proteobacteria	Betaproteobacteria	Burkholderiales	Comamonadaceae	uncultured	-	-	-	-	-	-	-	-	-	-	-	-	-	-	-	-	-	-	-	-	-	-	-	lip	-
4457554	0,0%	0,0%	0,0%	0,0%	0,0%	0,0%	0,0%	0,0%	0,0%	Bacteria	Proteobacteria	Alphaproteobacteria	Caulobacterales	Caulobacteraceae	Brevundimonas	-	-	-	nirA	-	-	-	norB	-	nifH	-	-	-	-	-	-	-	-	mmoX	-	laccase_Asco	laccase_Basidio	-	-	ppo
4380971	0,0%	0,0%	0,0%	0,0%	0,0%	0,0%	0,0%	0,0%	0,0%	Bacteria	Firmicutes	Clostridia	Clostridiales	Clostridiaceae	Clostridium sensu stricto 1	cellulolytic	-	-	-	-	-	-	-	-	-	-	-	-	-	-	-	-	-	-	-	-	-	-	-	-
4438989	0,0%	0,0%	0,0%	0,0%	0,0%	0,0%	0,0%	0,0%	0,0%	Bacteria	Proteobacteria	Alphaproteobacteria	Rhizobiales	Hyphomicrobiaceae	Hyphomicrobium	-	napA	narG	nirA	-	-	nirK	norB	nosZ	nifH	nifD	-	ureA	-	-	-	-	-	mmoX	-	laccase_Asco	laccase_Basidio	-	-	ppo
525648	0,0%	0,0%	0,0%	0,0%	0,0%	0,0%	0,0%	0,0%	0,0%	Bacteria	Proteobacteria	Alphaproteobacteria	Rhizobiales		Devosia	-	napA	-	-	-	-	nirK	-	-	nifH	-	-	ureA	-	-	-	-	chb	-	-	laccase_Asco	laccase_Basidio	-	-	ppo
84980	0,0%	0,0%	0,0%	0,0%	0,0%	0,0%	0,0%	0,0%	0,0%	Bacteria	Proteobacteria	Betaproteobacteria	Burkholderiales	Comamonadaceae	uncultured	-	-	-	-	-	-	-	-	-	-	-	-	-	-	-	-	-	-	-	-	-	-	-	lip	-
815975	0,0%	0,0%	0,0%	0,0%	0,0%	0,0%	0,0%	0,0%	0,0%	Bacteria	Proteobacteria	Alphaproteobacteria	Rhodobacterales	Hyphomonadaceae	Hirschia	-	napA	-	-	-	-	-	-	-	-	-	-	ureA	-	-	-	-	-	-	-	laccase_Asco	laccase_Basidio	-	-	ppo
228517	0,0%	0,0%	0,0%	0,0%	0,0%	0,0%	0,0%	0,0%	0,0%	Bacteria	Bacteroidetes	[Saprospirae]	[Saprospirales]	Saprospiraceae	Haliscomenobacter	-	napA	-	nirA	-	-	-	norB	nosZ	-	-	-	-	-	-	-	-	-	-	-	laccase_Asco	laccase_Basidio	-	-	ppo
4472157	0,0%	0,0%	0,0%	0,0%	0,0%	0,0%	0,0%	0,0%	0,0%	Bacteria	Proteobacteria	Alphaproteobacteria	Rhizobiales	Methylocystaceae	uncultured bacterium	-	-	-	-	-	-	-	-	-	-	-	-	-	-	-	-	-	-	-	-	-	-	-	-	-
692510	0,0%	0,0%	0,0%	0,0%	0,0%	0,0%	0,0%	0,0%	0,0%	Bacteria	Proteobacteria	Deltaproteobacteria	Bdellovibrionales	Bdellovibrionaceae	Bdellovibrio	-	-	-	-	-	-	nirK	norB	-	-	-	nrfA	-	-	-	-	-	-	-	-	laccase_Asco	laccase_Basidio	-	-	ppo
731678	0,0%	0,0%	0,0%	0,0%	0,0%	0,0%	0,0%	0,0%	0,0%	Bacteria	Bacteroidetes	Sphingobacteriia	Sphingobacteriales	Sphingobacteriaceae	Mucilaginibacter	-	-	-	-	-	-	-	-	-	-	-	-	-	-	-	-	-	-	-	-	-	-	-	-	-
666374	0,0%	0,0%	0,0%	0,0%	0,0%	0,0%	0,0%	0,0%	0,0%	Bacteria	Proteobacteria	Deltaproteobacteria	Desulfobacterales	Desulfobulbaceae	Desulfobulbus	-	napA	-	-	-	-	-	-	-	nifH	nifD	nrfA	-	dsrA	dsrB	-	-	-	-	-	-	-	-	-	-
1669782	0,0%	0,0%	0,0%	0,0%	0,0%	0,0%	0,0%	0,0%	0,0%	Bacteria	Actinobacteria	Thermoleophilia	Gaiellales	AK1AB1_02E	uncultured bacterium	-	-	-	-	-	-	-	-	-	-	-	-	-	-	-	-	-	-	-	-	-	-	-	-	-
356568	0,0%	0,0%	0,0%	0,0%	0,0%	0,0%	0,0%	0,0%	0,0%	Bacteria	Proteobacteria	Betaproteobacteria	Methylophilales	Methylophilaceae	Methylotenera	-	napA	-	nirA	-	-	nirK	norB	-	-	-	-	ureA	-	-	-	-	-	-	-	-	-	-	-	-
4464495	0,0%	0,0%	0,0%	0,0%	0,0%	0,0%	0,0%	0,0%	0,0%	Bacteria	Proteobacteria	Betaproteobacteria	Rhodocyclales	Rhodocyclaceae	Denitratisoma	-	-	-	-	-	-	-	-	-	-	-	-	-	-	-	-	-	-	-	-	-	-	-	-	-
4410974	0,0%	0,0%	0,0%	0,0%	0,0%	0,0%	0,0%	0,0%	0,0%	Bacteria	Proteobacteria	Betaproteobacteria	Burkholderiales	Comamonadaceae	Massilia	-	napA	narG	-	-	-	-	norB	nosZ	-	-	-	ureA	-	-	-	-	chb	mmoX	-	laccase_Asco	laccase_Basidio	-	-	ppo
311640	0,0%	0,0%	0,0%	0,0%	0,0%	0,0%	0,0%	0,0%	0,0%	Bacteria	Proteobacteria	Alphaproteobacteria	Rhizobiales		uncultured bacterium	-	-	-	-	-	-	-	-	-	-	-	-	-	-	-	-	-	-	-	-	-	-	-	-	-
4417972	0,0%	0,0%	0,0%	0,0%	0,0%	0,0%	0,0%	0,0%	0,0%	Bacteria	Planctomycetes	Planctomycetia	Pirellulales	Pirellulaceae	uncultured	-	-	-	-	-	-	-	-	-	-	-	-	-	-	-	-	-	-	-	-	-	-	-	lip	-
4330693	0,0%	0,0%	0,0%	0,0%	0,0%	0,0%	0,0%	0,0%	0,0%	Bacteria	Proteobacteria	Alphaproteobacteria	Rhizobiales		Defluviimonas	-	napA	narG	-	-	-	-	-	-	-	-	-	ureA	-	-	-	-	-	-	-	-	laccase_Basidio	-	-	ppo
4293003	0,0%	0,0%	0,0%	0,0%	0,0%	0,0%	0,0%	0,0%	0,0%	Bacteria	Proteobacteria	Alphaproteobacteria	Rhizobiales	Hyphomicrobiaceae	Hyphomicrobium	-	napA	narG	nirA	-	-	nirK	norB	nosZ	nifH	nifD	-	ureA	-	-	-	-	-	mmoX	-	laccase_Asco	laccase_Basidio	-	-	ppo
625087	0,0%	0,0%	0,0%	0,0%	0,0%	0,0%	0,0%	0,0%	0,0%	Bacteria	Proteobacteria	Gammaproteobacteria	Alteromonadales	OM60	OM60(NOR5) clade	-	-	-	-	-	-	-	-	-	-	-	-	-	-	-	-	-	-	-	-	-	-	-	-	-
4359608	0,0%	0,0%	0,0%	0,0%	0,0%	0,0%	0,0%	0,0%	0,0%	Bacteria	Proteobacteria	Alphaproteobacteria	Rhizobiales	Hyphomicrobiaceae	Pedomicrobium	-	-	-	-	-	-	nirK	-	-	-	-	-	-	-	-	-	-	-	-	-	-	-	-	-	-
4361153	0,0%	0,0%	0,0%	0,0%	0,0%	0,0%	0,0%	0,0%	0,0%	Bacteria	Bacteroidetes	Sphingobacteriia	Sphingobacteriales	Sphingobacteriaceae	Mucilaginibacter	-	-	-	-	-	-	-	-	-	-	-	-	-	-	-	-	-	-	-	-	-	-	-	-	-
142010	0,0%	0,0%	0,0%	0,0%	0,0%	0,0%	0,0%	0,0%	0,0%	Bacteria	Verrucomicrobia	Opitutae	Opitutales	Opitutaceae	Opitutus	-	napA	-	nirA	-	-	-	-	nosZ	-	-	nrfA	ureA	-	-	-	-	-	-	-	-	laccase_Basidio	-	-	-
4453723	0,0%	0,0%	0,0%	0,0%	0,0%	0,0%	0,0%	0,0%	0,0%	Bacteria	Verrucomicrobia	[Spartobacteria]	[Chthoniobacterales]	[Chthoniobacteraceae]	Chthoniobacter	-	napA	-	-	-	-	nirK	-	-	-	-	nrfA	ureA	-	-	-	-	-	-	-	-	-	-	-	-
100791	0,0%	0,0%	0,0%	0,0%	0,0%	0,0%	0,0%	0,0%	0,0%	Bacteria	Proteobacteria	Betaproteobacteria	Burkholderiales	Comamonadaceae	Comamonas	-	napA	-	nirA	-	nirS	nirK	norB	nosZ	-	-	-	ureA	-	-	SoxB	-	-	mmoX	-	-	laccase_Basidio	-	-	-
803166	0,0%	0,0%	0,0%	0,0%	0,0%	0,0%	0,0%	0,0%	0,0%	Bacteria	Proteobacteria	Deltaproteobacteria	Myxococcales		uncultured bacterium	-	-	-	-	-	-	-	-	-	-	-	-	-	-	-	-	-	-	-	-	-	-	-	-	-
663932	0,0%	0,0%	0,0%	0,0%	0,0%	0,0%	0,0%	0,0%	0,0%	Bacteria	Planctomycetes	Planctomycetia	Gemmatales	Gemmataceae	uncultured	-	-	-	-	-	-	-	-	-	-	-	-	-	-	-	-	-	-	-	-	-	-	-	lip	-
16939	0,0%	0,0%	0,0%	0,0%	0,0%	0,0%	0,0%	0,0%	0,0%	Bacteria	Proteobacteria	Deltaproteobacteria	Bdellovibrionales	Bdellovibrionaceae	Bdellovibrio	-	-	-	-	-	-	nirK	norB	-	-	-	nrfA	-	-	-	-	-	-	-	-	laccase_Asco	laccase_Basidio	-	-	ppo
4410004	0,0%	0,0%	0,0%	0,0%	0,0%	0,0%	0,0%	0,0%	0,0%	Bacteria	Bacteroidetes	Sphingobacteriia	Sphingobacteriales		uncultured bacterium	-	-	-	-	-	-	-	-	-	-	-	-	-	-	-	-	-	-	-	-	-	-	-	-	-
3478700	0,0%	0,0%	0,0%	0,0%	0,0%	0,0%	0,0%	0,0%	0,0%	Bacteria	Planctomycetes	Planctomycetia	Pirellulales	Pirellulaceae	Pirellula	-	napA	-	-	-	-	-	norB	-	-	-	-	-	-	-	-	-	-	-	-	-	-	-	-	-
4409759	0,0%	0,0%	0,0%	0,0%	0,0%	0,0%	0,0%	0,0%	0,0%	Bacteria	Proteobacteria	Alphaproteobacteria	Rhizobiales	Rhizobiaceae	Rhizobium	-	napA	narG	nirA	-	-	nirK	norB	nosZ	nifH	nifD	nrfA	ureA	-	-	-	-	chb	mmoX	-	laccase_Asco	laccase_Basidio	-	-	ppo
1138847	0,0%	0,0%	0,0%	0,0%	0,0%	0,0%	0,0%	0,0%	0,0%	Bacteria	Proteobacteria	Alphaproteobacteria	Rhodospirillales	Acetobacteraceae	Acidiphilium	-	napA	narG	-	-	-	-	-	-	nifH	-	-	-	-	-	SoxB	-	-	mmoX	-	laccase_Asco	laccase_Basidio	-	-	ppo
207957	0,0%	0,0%	0,0%	0,0%	0,0%	0,0%	0,0%	0,0%	0,0%	Bacteria	Gemmatimonadetes	Gemmatimonadetes	Gemmatimonadales	Gemmatimonadaceae	Gemmatimonas	-	napA	-	-	-	-	nirK	norB	nosZ	-	-	nrfA	-	dsrA	dsrB	-	-	chb	-	-	-	-	-	-	-
4470260	0,0%	0,0%	0,0%	0,0%	0,0%	0,0%	0,0%	0,0%	0,0%	Bacteria	Firmicutes	Bacilli	Bacillales		Sporosarcina	-	napA	narG	-	-	nirS	-	-	nosZ	-	-	-	ureA	-	-	-	-	-	-	-	-	laccase_Basidio	-	-	ppo
818488	0,0%	0,0%	0,0%	0,0%	0,0%	0,0%	0,0%	0,0%	0,0%	Bacteria	Proteobacteria	Betaproteobacteria	Gallionellales	Gallionellaceae	uncultured	-	-	-	-	-	-	-	-	-	-	-	-	-	-	-	-	-	-	-	-	-	-	-	lip	-
4454565	0,0%	0,0%	0,0%	0,0%	0,0%	0,0%	0,0%	0,0%	0,0%	Bacteria	Firmicutes	Bacilli	Bacillales		Bacillus	-	napA	narG	nirA	-	nirS	nirK	norB	nosZ	nifH	nifD	nrfA	ureA	-	-	-	-	-	mmoX	-	laccase_Asco	laccase_Basidio	-	-	ppo
215447	0,0%	0,0%	0,0%	0,0%	0,0%	0,0%	0,0%	0,0%	0,0%	Bacteria	Proteobacteria	Alphaproteobacteria	Rhizobiales	Hyphomicrobiaceae	Devosia	-	napA	-	-	-	-	nirK	-	-	nifH	-	-	ureA	-	-	-	-	chb	-	-	laccase_Asco	laccase_Basidio	-	-	ppo
4408378	0,0%	0,0%	0,0%	0,0%	0,0%	0,0%	0,0%	0,0%	0,0%	Bacteria	Proteobacteria	Betaproteobacteria	Burkholderiales	Comamonadaceae	Comamonas	-	napA	-	nirA	-	nirS	nirK	norB	nosZ	-	-	-	ureA	-	-	SoxB	-	-	mmoX	-	-	laccase_Basidio	-	-	-
4417400	0,0%	0,0%	0,0%	0,0%	0,0%	0,0%	0,0%	0,0%	0,0%	Bacteria	Proteobacteria	Betaproteobacteria	MND1		uncultured	-	-	-	-	-	-	-	-	-	-	-	-	-	-	-	-	-	-	-	-	-	-	-	lip	-
4379396	0,0%	0,0%	0,0%	0,0%	0,0%	0,0%	0,0%	0,0%	0,0%	Bacteria	Proteobacteria	Alphaproteobacteria	Rhizobiales	Hyphomicrobiaceae	Hyphomicrobium	-	napA	narG	nirA	-	-	nirK	norB	nosZ	nifH	nifD	-	ureA	-	-	-	-	-	mmoX	-	laccase_Asco	laccase_Basidio	-	-	ppo
4382194	0,0%	0,0%	0,0%	0,0%	0,0%	0,0%	0,0%	0,0%	0,0%	Bacteria	Bacteroidetes	[Saprospirae]	[Saprospirales]	Chitinophagaceae	Ferruginibacter	-	-	-	-	-	-	-	-	-	-	-	-	-	-	-	-	-	-	-	-	-	-	-	-	-
134470	0,0%	0,0%	0,0%	0,0%	0,0%	0,0%	0,0%	0,0%	0,0%	Bacteria	Firmicutes	Bacilli	Bacillales	Alicyclobacillaceae	Tumebacillus	cellulolytic	napA	-	-	-	-	-	-	-	-	-	-	-	-	-	-	-	-	-	-	-	-	-	-	-
737260	0,0%	0,0%	0,0%	0,0%	0,0%	0,0%	0,0%	0,0%	0,0%	Bacteria	Bacteroidetes	Cytophagia	Cytophagales	Cytophagaceae	Pseudarcicella	cellulolytic	-	-	-	-	-	-	-	-	-	-	-	-	-	-	-	-	-	-	-	-	-	-	-	-
527642	0,0%	0,0%	0,0%	0,0%	0,0%	0,0%	0,0%	0,1%	0,0%	Bacteria	Proteobacteria	Deltaproteobacteria	MIZ46		uncultured bacterium	-	-	-	-	-	-	-	-	-	-	-	-	-	-	-	-	-	-	-	-	-	-	-	-	-
173071	0,0%	0,0%	0,0%	0,0%	0,0%	0,0%	0,0%	0,0%	0,0%	Bacteria	Proteobacteria	Alphaproteobacteria	Rhizobiales		Ancalomicrobium	-	-	-	-	-	-	-	-	-	-	-	-	-	-	-	-	-	-	-	-	-	-	-	-	-
154174	0,0%	0,0%	0,0%	0,0%	0,0%	0,1%	0,0%	0,0%	0,0%	Bacteria	Proteobacteria	Alphaproteobacteria	Caulobacterales	Caulobacteraceae	Caulobacter	-	napA	narG	nirA	-	-	nirK	norB	-	-	-	-	-	-	-	-	-	chb	-	-	laccase_Asco	laccase_Basidio	-	-	ppo
89329	0,0%	0,0%	0,0%	0,0%	0,0%	0,0%	0,0%	0,0%	0,0%	Bacteria	Spirochaetes	Spirochaetes	Spirochaetales	Spirochaetaceae	Spirochaeta	-	-	-	nirA	-	-	-	-	-	nifH	nifD	-	-	-	-	-	-	-	-	-	-	-	-	-	-
562144	0,0%	0,0%	0,0%	0,0%	0,0%	0,0%	0,0%	0,0%	0,0%	Bacteria	Bacteroidetes	Cytophagia	Cytophagales	Cytophagaceae	uncultured	cellulolytic	-	-	-	-	-	-	-	-	-	-	-	-	-	-	-	-	-	-	-	-	-	-	lip	-
304089	0,0%	0,0%	0,0%	0,0%	0,0%	0,0%	0,0%	0,0%	0,0%	Bacteria	Actinobacteria	Thermoleophilia	Solirubrobacterales	Patulibacteraceae	Erythrobacter	-	napA	narG	nirA	-	-	-	-	-	nifH	-	-	-	-	-	-	-	-	-	-	laccase_Asco	laccase_Basidio	-	-	ppo
4475526	0,0%	0,0%	0,0%	0,0%	0,0%	0,0%	0,0%	0,0%	0,0%	Bacteria	Proteobacteria	Alphaproteobacteria	Rhodobacterales	Hyphomonadaceae	uncultured	-	-	-	-	-	-	-	-	-	-	-	-	-	-	-	-	-	-	-	-	-	-	-	lip	-
1119422	0,0%	0,0%	0,0%	0,0%	0,0%	0,0%	0,0%	0,0%	0,0%	Bacteria	Planctomycetes	Planctomycetia	Pirellulales	Pirellulaceae	Pirellula	-	napA	-	-	-	-	-	norB	-	-	-	-	-	-	-	-	-	-	-	-	-	-	-	-	-
3478456	0,0%	0,0%	0,0%	0,0%	0,0%	0,0%	0,0%	0,0%	0,0%	Bacteria	Proteobacteria	Alphaproteobacteria	Rhizobiales	Hyphomicrobiaceae	Hyphomicrobium	-	napA	narG	nirA	-	-	nirK	norB	nosZ	nifH	nifD	-	ureA	-	-	-	-	-	mmoX	-	laccase_Asco	laccase_Basidio	-	-	ppo
419180	0,0%	0,0%	0,0%	0,0%	0,0%	0,0%	0,0%	0,0%	0,0%	Bacteria	Proteobacteria	Gammaproteobacteria	Legionellales	Coxiellaceae	Aquicella	-	-	-	-	-	-	-	-	-	-	-	-	-	-	-	-	-	-	-	-	-	-	-	-	-
3473409	0,0%	0,0%	0,0%	0,0%	0,0%	0,0%	0,0%	0,0%	0,0%	Bacteria	Proteobacteria	Betaproteobacteria	Burkholderiales	Comamonadaceae	uncultured	-	-	-	-	-	-	-	-	-	-	-	-	-	-	-	-	-	-	-	-	-	-	-	lip	-
1059509	0,0%	0,0%	0,0%	0,0%	0,0%	0,0%	0,0%	0,0%	0,0%	Bacteria	Bacteroidetes	Flavobacteriia	Flavobacteriales	Flavobacteriaceae	Flavobacterium	cellulolytic	napA	-	nirA	-	-	nirK	norB	nosZ	-	-	-	ureA	-	-	-	-	-	-	-	laccase_Asco	laccase_Basidio	-	-	ppo
4393663	0,0%	0,0%	0,0%	0,0%	0,0%	0,0%	0,0%	0,0%	0,0%	Bacteria	Acidobacteria	Acidobacteriia	Acidobacteriales	Koribacteraceae	Candidatus Koribacter	-	-	-	-	-	-	-	-	-	-	-	-	-	-	-	-	-	-	-	-	-	-	-	-	-
4357324	0,0%	0,0%	0,0%	0,0%	0,0%	0,0%	0,0%	0,0%	0,0%	Bacteria	Proteobacteria	Alphaproteobacteria	Caulobacterales	Caulobacteraceae	Asticcacaulis	-	napA	narG	nirA	-	-	-	-	-	-	-	-	-	-	-	-	-	chb	-	-	laccase_Asco	laccase_Basidio	-	-	ppo
4393666	0,0%	0,0%	0,0%	0,0%	0,0%	0,0%	0,0%	0,0%	0,0%	Bacteria	Acidobacteria	Acidobacteriia	Acidobacteriales	Acidobacteriaceae	uncultured	cellulolytic	-	-	-	-	-	-	-	-	-	-	-	-	-	-	-	-	-	-	-	-	-	-	lip	-
806467	0,0%	0,0%	0,0%	0,0%	0,0%	0,0%	0,0%	0,0%	0,0%	Bacteria	Proteobacteria	Deltaproteobacteria	Spirobacillales		bacterium GLA1	-	-	-	-	-	-	-	-	-	-	-	-	-	-	-	-	-	-	-	-	-	-	-	-	-
250274	0,0%	0,0%	0,0%	0,0%	0,0%	0,0%	0,0%	0,0%	0,0%	Bacteria	Proteobacteria	Betaproteobacteria	Burkholderiales	Comamonadaceae	uncultured	-	-	-	-	-	-	-	-	-	-	-	-	-	-	-	-	-	-	-	-	-	-	-	lip	-
566265	0,0%	0,0%	0,0%	0,0%	0,0%	0,0%	0,0%	0,0%	0,0%	Bacteria	Proteobacteria	Alphaproteobacteria	BD7-3		uncultured bacterium	-	-	-	-	-	-	-	-	-	-	-	-	-	-	-	-	-	-	-	-	-	-	-	-	-
1118783	0,0%	0,0%	0,0%	0,0%	0,0%	0,0%	0,0%	0,0%	0,0%	Bacteria	Proteobacteria	Betaproteobacteria			uncultured	-	-	-	-	-	-	-	-	-	-	-	-	-	-	-	-	-	-	-	-	-	-	-	lip	-
969266	0,0%	0,0%	0,0%	0,0%	0,0%	0,0%	0,0%	0,0%	0,0%	Bacteria	Proteobacteria	Deltaproteobacteria	Sva0853	SAR324	uncultured delta proteobacterium	-	-	-	-	-	-	-	-	-	-	-	-	-	-	-	-	-	-	-	-	-	-	-	-	-
581019	0,0%	0,0%	0,0%	0,0%	0,0%	0,0%	0,0%	0,0%	0,0%	Bacteria	Proteobacteria	Alphaproteobacteria	Sphingomonadales	Sphingomonadaceae	Novosphingobium	-	napA	-	-	-	-	-	norB	-	nifH	nifD	-	ureA	-	-	-	-	-	mmoX	-	laccase_Asco	laccase_Basidio	-	-	ppo
43917	0,0%	0,0%	0,0%	0,0%	0,0%	0,0%	0,0%	0,0%	0,0%	Bacteria	Proteobacteria	Betaproteobacteria	Burkholderiales	Comamonadaceae	uncultured	-	-	-	-	-	-	-	-	-	-	-	-	-	-	-	-	-	-	-	-	-	-	-	lip	-
4337622	0,0%	0,0%	0,0%	0,0%	0,0%	0,0%	0,0%	0,0%	0,0%	Bacteria	Proteobacteria	Alphaproteobacteria	Rhizobiales	Rhizobiaceae	Ochrobactrum	-	napA	narG	nirA	-	-	nirK	norB	nosZ	nifH	-	-	ureA	-	-	-	-	-	-	-	laccase_Asco	laccase_Basidio	-	-	ppo
821052	0,0%	0,0%	0,0%	0,0%	0,0%	0,0%	0,0%	0,0%	0,0%	Bacteria	Acidobacteria	Acidobacteriia	Acidobacteriales	Koribacteraceae	uncultured	-	-	-	-	-	-	-	-	-	-	-	-	-	-	-	-	-	-	-	-	-	-	-	lip	-
52053	0,0%	0,0%	0,0%	0,0%	0,0%	0,0%	0,0%	0,0%	0,0%	Bacteria	Proteobacteria	Alphaproteobacteria	Rhizobiales	Hyphomicrobiaceae	uncultured	-	-	-	-	-	-	-	-	-	-	-	-	-	-	-	-	-	-	-	-	-	-	-	lip	-
4396925	0,0%	0,0%	0,0%	0,0%	0,0%	0,0%	0,0%	0,0%	0,0%	Bacteria	Acidobacteria	Acidobacteriia	Acidobacteriales	Koribacteraceae	uncultured	-	-	-	-	-	-	-	-	-	-	-	-	-	-	-	-	-	-	-	-	-	-	-	lip	-
4460871	0,0%	0,0%	0,0%	0,0%	0,0%	0,0%	0,0%	0,0%	0,0%	Bacteria	Proteobacteria	Betaproteobacteria	Methylophilales	Methylophilaceae	uncultured	-	-	-	-	-	-	-	-	-	-	-	-	-	-	-	-	-	-	-	-	-	-	-	lip	-
3196019	0,0%	0,0%	0,0%	0,0%	0,0%	0,0%	0,0%	0,0%	0,1%	Bacteria	Proteobacteria	Deltaproteobacteria	Myxococcales	Haliangiaceae	Haliangium	-	-	-	-	-	-	-	-	-	-	-	-	ureA	-	-	-	-	chb	-	-	-	-	-	-	-
4388385	0,0%	0,0%	0,0%	0,0%	0,0%	0,0%	0,0%	0,0%	0,0%	Bacteria	Proteobacteria	Alphaproteobacteria	Rhizobiales		Ochrobactrum	-	napA	narG	nirA	-	-	nirK	norB	nosZ	nifH	-	-	ureA	-	-	-	-	-	-	-	laccase_Asco	laccase_Basidio	-	-	ppo
1109902	0,0%	0,0%	0,0%	0,0%	0,0%	0,0%	0,0%	0,0%	0,0%	Bacteria	Acidobacteria	DA052	Ellin6513		uncultured bacterium	-	-	-	-	-	-	-	-	-	-	-	-	-	-	-	-	-	-	-	-	-	-	-	-	-
2313705	0,0%	0,0%	0,0%	0,0%	0,0%	0,0%	0,0%	0,0%	0,0%	Bacteria	Proteobacteria	Alphaproteobacteria	Sphingomonadales	Sphingomonadaceae	Novosphingobium	-	napA	-	-	-	-	-	norB	-	nifH	nifD	-	ureA	-	-	-	-	-	mmoX	-	laccase_Asco	laccase_Basidio	-	-	ppo
4427396	0,0%	0,0%	0,0%	0,0%	0,0%	0,0%	0,0%	0,0%	0,0%	Bacteria	Verrucomicrobia	Opitutae	Opitutales	Opitutaceae	Opitutus	-	napA	-	nirA	-	-	-	-	nosZ	-	-	nrfA	ureA	-	-	-	-	-	-	-	-	laccase_Basidio	-	-	-
4333186	0,0%	0,0%	0,0%	0,0%	0,0%	0,0%	0,0%	0,0%	0,0%	Bacteria	Proteobacteria	Betaproteobacteria	Rhodocyclales	Rhodocyclaceae	Ferribacterium	-	-	-	-	-	-	-	-	-	-	-	-	-	-	-	-	-	-	-	-	-	-	-	-	-
4484193	0,0%	0,0%	0,0%	0,0%	0,0%	0,0%	0,0%	0,0%	0,0%	Bacteria	Proteobacteria	Gammaproteobacteria	Xanthomonadales	Xanthomonadaceae	Dyella	cellulolytic	napA	-	-	-	-	-	norB	nosZ	-	-	-	-	-	-	-	-	-	-	-	laccase_Asco	laccase_Basidio	-	-	ppo
3780777	0,0%	0,0%	0,0%	0,0%	0,0%	0,0%	0,0%	0,0%	0,0%	Bacteria	Proteobacteria	Betaproteobacteria			uncultured	-	-	-	-	-	-	-	-	-	-	-	-	-	-	-	-	-	-	-	-	-	-	-	lip	-
4344428	0,0%	0,0%	0,0%	0,0%	0,0%	0,0%	0,0%	0,0%	0,0%	Bacteria	Proteobacteria	Alphaproteobacteria	Rhodospirillales	Rhodospirillaceae	Telmatospirillum	-	-	-	-	-	-	-	-	-	nifH	-	-	-	-	-	-	-	-	-	-	-	-	-	-	-
582110	0,0%	0,0%	0,0%	0,0%	0,0%	0,1%	0,0%	0,0%	0,0%	Bacteria	Proteobacteria	Deltaproteobacteria	Myxococcales	Myxococcaceae	Anaeromyxobacter	-	napA	narG	nirA	-	nirS	-	norB	nosZ	nifH	nifD	nrfA	ureA	-	-	SoxB	-	-	-	-	-	laccase_Basidio	-	-	-
4411138	0,0%	0,0%	0,0%	0,0%	0,0%	0,0%	0,0%	0,0%	0,0%	Bacteria	Actinobacteria	Actinobacteria	Actinomycetales	Micrococcaceae	Rothia	-	-	narG	-	-	-	-	norB	-	-	-	-	-	-	-	-	-	-	-	-	-	laccase_Basidio	-	-	-
894654	0,0%	0,0%	0,0%	0,0%	0,0%	0,0%	0,0%	0,0%	0,0%	Bacteria	Proteobacteria	Gammaproteobacteria	Legionellales	Legionellaceae	Legionella	-	-	-	-	-	nirS	-	norB	-	-	-	-	-	-	-	-	-	-	-	-	laccase_Asco	laccase_Basidio	-	-	ppo
204148	0,0%	0,0%	0,0%	0,0%	0,0%	0,0%	0,0%	0,0%	0,0%	Bacteria	Proteobacteria	Alphaproteobacteria	Ellin329		Rhizomicrobium	-	-	-	-	-	-	-	-	-	-	-	-	-	-	-	-	-	-	-	-	-	-	-	-	-
535429	0,0%	0,0%	0,0%	0,0%	0,0%	0,0%	0,0%	0,0%	0,0%	Bacteria	Proteobacteria	Alphaproteobacteria	Rhizobiales		Methylosinus	-	napA	-	nirA	-	-	-	-	-	nifH	nifD	-	ureA	-	-	-	-	-	mmoX	pmoA	-	-	-	-	-
210657	0,0%	0,0%	0,0%	0,0%	0,0%	0,0%	0,0%	0,0%	0,0%	Bacteria	Proteobacteria	Alphaproteobacteria	Rhodospirillales	Rhodospirillaceae	uncultured Hyphomicrobiaceae bacterium	-	-	-	-	-	-	-	-	-	-	-	-	-	-	-	-	-	-	-	-	-	-	-	-	-
3091248	0,0%	0,0%	0,0%	0,0%	0,0%	0,0%	0,0%	0,0%	0,0%	Bacteria	Proteobacteria	Betaproteobacteria	Methylophilales	Methylophilaceae	uncultured	-	-	-	-	-	-	-	-	-	-	-	-	-	-	-	-	-	-	-	-	-	-	-	lip	-
204785	0,0%	0,0%	0,0%	0,0%	0,0%	0,0%	0,0%	0,0%	0,0%	Bacteria	Bacteroidetes	[Saprospirae]	[Saprospirales]	Chitinophagaceae	Segetibacter	-	-	-	-	-	-	-	-	-	-	-	-	-	-	-	-	-	-	-	-	-	-	-	-	-
4315250	0,0%	0,0%	0,0%	0,0%	0,0%	0,0%	0,0%	0,0%	0,0%	Bacteria	Proteobacteria	Betaproteobacteria	Burkholderiales	Comamonadaceae	uncultured	-	-	-	-	-	-	-	-	-	-	-	-	-	-	-	-	-	-	-	-	-	-	-	lip	-
1008869	0,0%	0,0%	0,0%	0,0%	0,0%	0,0%	0,0%	0,0%	0,0%	Bacteria	Proteobacteria	Gammaproteobacteria	Methylococcales	Crenotrichaceae	Crenothrix	-	-	-	-	-	-	-	-	-	-	-	-	-	-	-	-	-	-	-	pmoA	-	-	-	-	-
4315258	0,0%	0,0%	0,0%	0,0%	0,0%	0,0%	0,0%	0,0%	0,0%	Bacteria	Bacteroidetes	Bacteroidia	Bacteroidales	Porphyromonadaceae	Paludibacter	-	-	-	-	-	-	-	-	-	nifH	nifD	-	-	-	-	-	-	-	-	-	-	-	-	-	-
4336275	0,0%	0,0%	0,0%	0,0%	0,0%	0,0%	0,0%	0,0%	0,0%	Bacteria	Proteobacteria	Alphaproteobacteria	Sphingomonadales	Sphingomonadaceae	uncultured bacterium	-	-	-	-	-	-	-	-	-	-	-	-	-	-	-	-	-	-	-	-	-	-	-	-	-
1079393	0,0%	0,0%	0,0%	0,0%	0,0%	0,0%	0,0%	0,0%	0,0%	Bacteria	Actinobacteria	Thermoleophilia	Solirubrobacterales		uncultured bacterium	-	-	-	-	-	-	-	-	-	-	-	-	-	-	-	-	-	-	-	-	-	-	-	-	-
633243	0,0%	0,0%	0,0%	0,0%	0,0%	0,0%	0,0%	0,0%	0,0%	Bacteria	Actinobacteria	Actinobacteria	Actinomycetales	Microbacteriaceae	Candidatus Rhodoluna	cellulolytic	-	-	-	-	-	-	-	-	-	-	-	-	-	-	-	-	-	-	-	-	-	-	-	-
537648	0,0%	0,0%	0,0%	0,0%	0,0%	0,0%	0,0%	0,0%	0,0%	Bacteria	Proteobacteria	Deltaproteobacteria	Myxococcales	OM27	OM27 clade	-	-	-	-	-	-	-	-	-	-	-	-	-	-	-	-	-	-	-	-	-	-	-	-	-
4366287	0,0%	0,0%	0,0%	0,0%	0,0%	0,0%	0,0%	0,0%	0,0%	Bacteria	Proteobacteria	Alphaproteobacteria	Rickettsiales	Pelagibacteraceae	uncultured bacterium	-	-	-	-	-	-	-	-	-	-	-	-	-	-	-	-	-	-	-	-	-	-	-	-	-
4392826	0,0%	0,0%	0,0%	0,0%	0,0%	0,0%	0,0%	0,0%	0,0%	Bacteria	Bacteroidetes	Sphingobacteriia	Sphingobacteriales		uncultured bacterium	-	-	-	-	-	-	-	-	-	-	-	-	-	-	-	-	-	-	-	-	-	-	-	-	-

4296910	0,0%	0,0%	0,0%	0,0%	0,0%	0,1%	0,0%	0,0%	0,0%	Bacteria	Proteobacteria	Alphaproteobacteria	Rhizobiales		uncultured bacterium	-	-	-	-	-	-	-	-	-	-	-	-	-	-	-	-	-	-	-	-	-	-	-	-	-
4075741	0,0%	0,0%	0,0%	0,0%	0,0%	0,0%	0,0%	0,0%	0,0%	Bacteria	Proteobacteria	Alphaproteobacteria	Rhizobiales	Hyphomicrobiaceae	Hyphomicrobium	-	napA	narG	nirA	-	-	nirK	norB	nosZ	nifH	nifD	-	ureA	-	-	-	-	-	mmoX	-	laccase_Asco	laccase_Basidio	-	-	ppo
1120835	0,0%	0,0%	0,0%	0,0%	0,0%	0,0%	0,0%	0,0%	0,0%	Bacteria	Proteobacteria	Alphaproteobacteria	Rhizobiales	Methylocystaceae	Methylosinus	-	napA	-	nirA	-	-	-	-	-	nifH	nifD	-	ureA	-	-	-	-	-	mmoX	pmoA	-	-	-	-	-
4476089	0,0%	0,0%	0,0%	0,0%	0,0%	0,0%	0,0%	0,0%	0,0%	Bacteria	Proteobacteria	Alphaproteobacteria	Caulobacterales	Caulobacteraceae	uncultured	-	-	-	-	-	-	-	-	-	-	-	-	-	-	-	-	-	-	-	-	-	-	-	lip	-
2720386	0,0%	0,0%	0,0%	0,0%	0,0%	0,0%	0,0%	0,0%	0,0%	Bacteria	Proteobacteria	Alphaproteobacteria	Rhodospirillales	Rhodospirillaceae	uncultured	-	-	-	-	-	-	-	-	-	-	-	-	-	-	-	-	-	-	-	-	-	-	-	lip	-
265055	0,0%	0,0%	0,0%	0,0%	0,0%	0,0%	0,0%	0,0%	0,0%	Bacteria	Proteobacteria	Deltaproteobacteria	Bdellovibrionales	Bdellovibrionaceae	Bdellovibrio	-	-	-	-	-	-	nirK	norB	-	-	-	nrfA	-	-	-	-	-	-	-	-	laccase_Asco	laccase_Basidio	-	-	ppo
2139819	0,0%	0,0%	0,0%	0,0%	0,0%	0,0%	0,0%	0,0%	0,0%	Bacteria	Proteobacteria	Gammaproteobacteria	Legionellales	Legionellaceae	Legionella	-	-	-	-	-	nirS	-	norB	-	-	-	-	-	-	-	-	-	-	-	-	laccase_Asco	laccase_Basidio	-	-	ppo
242145	0,0%	0,0%	0,0%	0,0%	0,0%	0,0%	0,0%	0,0%	0,0%	Bacteria	Planctomycetes	Planctomycetia	Gemmatales	Gemmataceae	Gemmata	-	-	-	-	-	-	-	-	-	-	-	-	-	-	-	-	-	-	-	-	-	-	-	-	-
1136697	0,0%	0,0%	0,0%	0,0%	0,0%	0,0%	0,0%	0,0%	0,0%	Bacteria	Proteobacteria	Alphaproteobacteria	Rhodospirillales	Rhodospirillaceae	Defluviicoccus	-	-	-	-	-	-	-	-	-	-	-	-	-	-	-	-	-	-	-	-	-	-	-	-	-
982282	0,0%	0,0%	0,0%	0,0%	0,0%	0,0%	0,0%	0,0%	0,0%	Bacteria	Planctomycetes	Phycisphaerae	WD2101		uncultured bacterium	-	-	-	-	-	-	-	-	-	-	-	-	-	-	-	-	-	-	-	-	-	-	-	-	-
264673	0,0%	0,0%	0,0%	0,0%	0,0%	0,0%	0,0%	0,0%	0,0%	Bacteria	Proteobacteria	Gammaproteobacteria	Pseudomonadales	Pseudomonadaceae	Pseudomonas	cellulolytic	napA	narG	nirA	-	nirS	nirK	norB	nosZ	nifH	nifD	-	ureA	-	dsrB	SoxB	-	-	mmoX	-	laccase_Asco	laccase_Basidio	-	-	ppo
4475561	0,0%	0,0%	0,0%	0,0%	0,0%	0,0%	0,0%	0,0%	0,0%	Bacteria	Proteobacteria	Alphaproteobacteria	Rhizobiales	Bradyrhizobiaceae	Afipia	-	napA	narG	-	-	-	nirK	-	nosZ	nifH	-	-	ureA	-	-	-	-	-	-	-	laccase_Asco	laccase_Basidio	-	-	ppo
4463892	0,0%	0,0%	0,0%	0,0%	0,0%	0,0%	0,0%	0,0%	0,0%	Bacteria	Bacteroidetes	Bacteroidia	Bacteroidales	Bacteroidaceae	Bacteroides	cellulolytic	-	narG	-	-	-	-	-	-	-	nifD	nrfA	-	-	dsrB	-	-	chb	-	-	-	-	-	-	-
1109053	0,0%	0,0%	0,0%	0,0%	0,0%	0,0%	0,0%	0,0%	0,0%	Bacteria	Spirochaetes	Spirochaetes	Spirochaetales	Spirochaetaceae	Spirochaeta	-	-	-	nirA	-	-	-	-	-	nifH	nifD	-	-	-	-	-	-	-	-	-	-	-	-	-	-
868555	0,0%	0,0%	0,0%	0,0%	0,0%	0,0%	0,0%	0,0%	0,1%	Bacteria	Planctomycetes	Planctomycetia	Gemmatales	Isosphaeraceae	uncultured bacterium	-	-	-	-	-	-	-	-	-	-	-	-	-	-	-	-	-	-	-	-	-	-	-	-	-
736813	0,0%	0,0%	0,0%	0,0%	0,0%	0,0%	0,0%	0,0%	0,0%	Bacteria	Actinobacteria	Actinobacteria	Actinomycetales	Mycobacteriaceae	Mycobacterium	-	napA	narG	nirA	-	-	-	norB	-	-	-	-	ureA	-	-	-	amoA_AOB	-	mmoX	pmoA	laccase_Asco	laccase_Basidio	-	-	ppo
1046664	0,0%	0,0%	0,0%	0,0%	0,0%	0,0%	0,0%	0,0%	0,0%	Bacteria	Actinobacteria	Acidimicrobiia	Acidimicrobiales		uncultured	-	-	-	-	-	-	-	-	-	-	-	-	-	-	-	-	-	-	-	-	-	-	-	lip	-
138368	0,0%	0,0%	0,0%	0,0%	0,0%	0,0%	0,0%	0,0%	0,0%	Bacteria	Firmicutes	Clostridia	Clostridiales	Peptostreptococcaceae	Incertae Sedis	-	-	-	-	-	-	-	-	-	-	-	-	-	-	-	-	-	-	-	-	-	-	-	-	-
4455618	0,0%	0,0%	0,0%	0,0%	0,0%	0,0%	0,0%	0,0%	0,0%	Bacteria	Bacteroidetes	Flavobacteriia	Flavobacteriales	[Weeksellaceae]	Chryseobacterium	-	napA	-	-	-	-	nirK	norB	nosZ	nifH	-	-	ureA	-	-	-	-	chb	-	-	laccase_Asco	laccase_Basidio	-	-	ppo
527532	0,0%	0,0%	0,0%	0,0%	0,0%	0,0%	0,0%	0,0%	0,0%	Bacteria	Planctomycetes	Planctomycetia	Pirellulales	Pirellulaceae	uncultured	-	-	-	-	-	-	-	-	-	-	-	-	-	-	-	-	-	-	-	-	-	-	-	lip	-
757795	0,0%	0,0%	0,0%	0,0%	0,0%	0,0%	0,0%	0,0%	0,0%	Bacteria	Actinobacteria	Thermoleophilia	Solirubrobacterales		uncultured bacterium	-	-	-	-	-	-	-	-	-	-	-	-	-	-	-	-	-	-	-	-	-	-	-	-	-
742886	0,0%	0,0%	0,0%	0,0%	0,0%	0,0%	0,0%	0,0%	0,0%	Bacteria	Proteobacteria	Gammaproteobacteria	Thiotrichales	Piscirickettsiaceae	uncultured bacterium	-	-	-	-	-	-	-	-	-	-	-	-	-	-	-	-	-	-	-	-	-	-	-	-	-
210832	0,0%	0,0%	0,0%	0,0%	0,0%	0,0%	0,0%	0,0%	0,1%	Bacteria	Planctomycetes	Planctomycetia	Gemmatales	Gemmataceae	uncultured	-	-	-	-	-	-	-	-	-	-	-	-	-	-	-	-	-	-	-	-	-	-	-	lip	-
1124436	0,0%	0,0%	0,0%	0,0%	0,0%	0,0%	0,0%	0,0%	0,0%	Bacteria	Planctomycetes	Planctomycetia	Pirellulales	Pirellulaceae	Pirellula	-	napA	-	-	-	-	-	norB	-	-	-	-	-	-	-	-	-	-	-	-	-	-	-	-	-
819537	0,0%	0,0%	0,0%	0,0%	0,0%	0,0%	0,0%	0,0%	0,0%	Bacteria	Verrucomicrobia	Opitutae	[Cerasicoccales]	[Cerasicoccaceae]	uncultured Verrucomicrobium sp.	-	-	-	-	-	-	-	-	-	-	-	-	-	-	-	-	-	-	-	-	-	-	-	-	-
4434051	0,0%	0,0%	0,0%	0,0%	0,0%	0,0%	0,0%	0,0%	0,0%	Bacteria	Proteobacteria	Gammaproteobacteria	Methylococcales	Methylococcaceae	Methylomonas	-	napA	-	nirA	-	nirS	nirK	norB	-	nifH	nifD	-	ureA	-	-	-	amoA_AOB	-	mmoX	pmoA	laccase_Asco	laccase_Basidio	-	-	ppo
626238	0,0%	0,0%	0,0%	0,0%	0,0%	0,0%	0,0%	0,0%	0,0%	Bacteria	Acidobacteria	Acidobacteriia	Acidobacteriales	Acidobacteriaceae	Telmatobacter	cellulolytic	-	-	-	-	-	-	-	-	-	-	-	-	-	-	-	-	-	-	-	-	-	-	-	-
810076	0,0%	0,0%	0,0%	0,0%	0,0%	0,0%	0,0%	0,0%	0,0%	Bacteria	Proteobacteria	Deltaproteobacteria	Myxococcales	Haliangiaceae	Haliangium	-	-	-	-	-	-	-	-	-	-	-	-	ureA	-	-	-	-	chb	-	-	-	-	-	-	-
219727	0,0%	0,0%	0,0%	0,0%	0,0%	0,0%	0,0%	0,0%	0,0%	Bacteria	Bacteroidetes	Flavobacteriia	Flavobacteriales	Flavobacteriaceae	Flavobacterium	cellulolytic	napA	-	nirA	-	-	nirK	norB	nosZ	-	-	-	ureA	-	-	-	-	-	-	-	laccase_Asco	laccase_Basidio	-	-	ppo
512552	0,0%	0,0%	0,0%	0,0%	0,0%	0,0%	0,0%	0,0%	0,0%	Bacteria	Planctomycetes	Planctomycetia	Gemmatales	Gemmataceae	uncultured	-	-	-	-	-	-	-	-	-	-	-	-	-	-	-	-	-	-	-	-	-	-	-	lip	-
26459	0,0%	0,0%	0,0%	0,0%	0,0%	0,0%	0,0%	0,0%	0,0%	Bacteria	Bacteroidetes	[Saprospirae]	[Saprospirales]	Chitinophagaceae	uncultured	-	-	-	-	-	-	-	-	-	-	-	-	-	-	-	-	-	-	-	-	-	-	-	lip	-
4362942	0,0%	0,0%	0,0%	0,0%	0,0%	0,0%	0,0%	0,0%	0,0%	Bacteria	Firmicutes	Bacilli	Lactobacillales	Enterococcaceae	uncultured bacterium	-	-	-	-	-	-	-	-	-	-	-	-	-	-	-	-	-	-	-	-	-	-	-	-	-
1109067	0,0%	0,0%	0,0%	0,0%	0,0%	0,0%	0,0%	0,0%	0,0%	Bacteria	Proteobacteria	Alphaproteobacteria	Rhizobiales	Methylobacteriaceae	Methylobacterium	-	napA	narG	-	-	-	nirK	-	nosZ	nifH	nifD	-	ureA	-	-	-	-	-	-	-	laccase_Asco	laccase_Basidio	-	-	ppo
4333746	0,0%	0,0%	0,0%	0,0%	0,0%	0,0%	0,0%	0,0%	0,0%	Bacteria	Proteobacteria	Alphaproteobacteria	Rhodobacterales	Rhodobacteraceae	Rhodobacter	-	napA	-	nirA	-	nirS	nirK	norB	nosZ	nifH	nifD	-	ureA	-	-	SoxB	-	-	mmoX	-	laccase_Asco	laccase_Basidio	-	-	ppo
4473114	0,0%	0,0%	0,0%	0,0%	0,0%	0,1%	0,0%	0,0%	0,0%	Bacteria	Proteobacteria	Betaproteobacteria	Burkholderiales	Comamonadaceae	Achromobacter	-	napA	narG	-	-	nirS	nirK	norB	nosZ	-	-	nrfA	ureA	-	-	-	-	-	mmoX	-	laccase_Asco	laccase_Basidio	-	-	ppo
1115406	0,0%	0,0%	0,0%	0,0%	0,0%	0,0%	0,0%	0,0%	0,0%	Bacteria	Verrucomicrobia	[Spartobacteria]	[Chthoniobacterales]	[Chthoniobacteraceae]	Chthoniobacter	-	napA	-	-	-	-	nirK	-	-	-	-	nrfA	ureA	-	-	-	-	-	-	-	-	-	-	-	-
247781	0,0%	0,0%	0,0%	0,0%	0,0%	0,0%	0,0%	0,0%	0,0%	Bacteria	Proteobacteria	Alphaproteobacteria			Defluviicoccus	-	-	-	-	-	-	-	-	-	-	-	-	-	-	-	-	-	-	-	-	-	-	-	-	-
1072854	0,0%	0,0%	0,0%	0,0%	0,0%	0,0%	0,0%	0,0%	0,0%	Bacteria	Actinobacteria	Actinobacteria	Actinomycetales		Arthrobacter	-	napA	narG	-	-	-	-	-	-	nifH	-	-	ureA	dsrA	-	-	-	-	mmoX	-	laccase_Asco	laccase_Basidio	-	-	ppo
4413642	0,0%	0,0%	0,0%	0,0%	0,0%	0,0%	0,0%	0,0%	0,0%	Bacteria	Proteobacteria	Alphaproteobacteria	Rhodospirillales	Rhodospirillaceae	Elstera	-	-	-	-	-	-	-	-	-	-	-	-	-	-	-	-	-	-	-	-	-	-	-	-	-
828311	0,0%	0,0%	0,0%	0,0%	0,0%	0,0%	0,0%	0,0%	0,0%	Bacteria	Proteobacteria	Alphaproteobacteria	Rhizobiales		Meganema	-	-	-	-	-	-	-	-	-	-	-	-	-	-	-	-	-	-	-	-	-	-	-	-	-
223897	0,0%	0,0%	0,0%	0,0%	0,0%	0,0%	0,0%	0,0%	0,0%	Bacteria	Proteobacteria	Gammaproteobacteria	Pseudomonadales	Moraxellaceae	Alkanindiges	-	-	-	-	-	-	-	-	-	-	-	-	-	-	-	-	-	-	-	-	-	-	-	-	-
683436	0,0%	0,0%	0,0%	0,0%	0,0%	0,0%	0,0%	0,0%	0,0%	Bacteria	Proteobacteria	Deltaproteobacteria	Myxococcales		Sorangium	-	napA	-	-	-	-	nirK	norB	-	-	-	nrfA	ureA	-	-	-	-	chb	-	-	laccase_Asco	laccase_Basidio	-	-	ppo
216368	0,0%	0,0%	0,0%	0,0%	0,0%	0,0%	0,0%	0,0%	0,0%	Bacteria	Planctomycetes	BD7-11			uncultured bacterium	-	-	-	-	-	-	-	-	-	-	-	-	-	-	-	-	-	-	-	-	-	-	-	-	-
1141156	0,0%	0,0%	0,0%	0,0%	0,0%	0,0%	0,0%	0,0%	0,0%	Bacteria	Acidobacteria	Solibacteres	Solibacterales	Solibacteraceae	Candidatus Solibacter	-	-	-	-	-	-	-	-	-	-	-	-	-	-	-	-	-	-	-	-	-	-	-	-	-
1050747	0,0%	0,0%	0,0%	0,0%	0,0%	0,1%	0,0%	0,0%	0,0%	Bacteria	Verrucomicrobia	[Spartobacteria]	[Chthoniobacterales]	[Chthoniobacteraceae]	uncultured bacterium	-	-	-	-	-	-	-	-	-	-	-	-	-	-	-	-	-	-	-	-	-	-	-	-	-
585708	0,0%	0,0%	0,0%	0,0%	0,0%	0,0%	0,0%	0,0%	0,0%	Bacteria	Proteobacteria	Alphaproteobacteria	Sphingomonadales	Sphingomonadaceae	Sphingobium	-	napA	-	nirA	-	-	-	norB	-	-	-	-	ureA	-	-	-	-	-	mmoX	-	laccase_Asco	laccase_Basidio	-	-	ppo
1597279	0,0%	0,0%	0,0%	0,0%	0,0%	0,0%	0,0%	0,0%	0,0%	Bacteria	Bacteroidetes	Sphingobacteriia	Sphingobacteriales		uncultured bacterium	-	-	-	-	-	-	-	-	-	-	-	-	-	-	-	-	-	-	-	-	-	-	-	-	-
242829	0,0%	0,0%	0,0%	0,0%	0,0%	0,0%	0,0%	0,0%	0,0%	Bacteria	Proteobacteria	Alphaproteobacteria	Caulobacterales	Caulobacteraceae	uncultured	-	-	-	-	-	-	-	-	-	-	-	-	-	-	-	-	-	-	-	-	-	-	-	lip	-
216180	0,0%	0,0%	0,0%	0,0%	0,0%	0,1%	0,0%	0,0%	0,0%	Bacteria	Bacteroidetes	[Saprospirae]	[Saprospirales]	Chitinophagaceae	uncultured	-	-	-	-	-	-	-	-	-	-	-	-	-	-	-	-	-	-	-	-	-	-	-	lip	-
843003	0,0%	0,0%	0,0%	0,0%	0,0%	0,0%	0,0%	0,0%	0,0%	Bacteria	Proteobacteria	Betaproteobacteria	Burkholderiales	Comamonadaceae	Aquincola	-	-	-	-	-	-	-	-	-	-	-	-	-	-	-	-	-	-	-	-	-	-	-	-	-
2565174	0,0%	0,0%	0,0%	0,0%	0,0%	0,0%	0,0%	0,0%	0,0%	Bacteria	Planctomycetes	Planctomycetia	Gemmatales	Gemmataceae	Gemmata	-	-	-	-	-	-	-	-	-	-	-	-	-	-	-	-	-	-	-	-	-	-	-	-	-
4397998	0,0%	0,0%	0,0%	0,0%	0,0%	0,0%	0,0%	0,0%	0,0%	Bacteria	Proteobacteria	Alphaproteobacteria	Sphingomonadales	Sphingomonadaceae	Sphingopyxis	-	napA	-	-	-	-	-	norB	-	-	-	-	-	-	-	-	-	chb	-	-	laccase_Asco	laccase_Basidio	-	-	ppo
153998	0,0%	0,0%	0,0%	0,0%	0,0%	0,1%	0,0%	0,0%	0,0%	Bacteria	Actinobacteria	Acidimicrobiia	Acidimicrobiales		Iamia	-	-	-	-	-	-	-	-	-	-	-	-	-	-	-	-	-	-	-	-	-	-	-	-	-
511452	0,0%	0,0%	0,0%	0,0%	0,0%	0,0%	0,0%	0,0%	0,0%	Bacteria	Proteobacteria	Gammaproteobacteria	Legionellales	Coxiellaceae	uncultured bacterium	-	-	-	-	-	-	-	-	-	-	-	-	-	-	-	-	-	-	-	-	-	-	-	-	-
565604	0,0%	0,0%	0,0%	0,0%	0,0%	0,0%	0,0%	0,0%	0,0%	Bacteria	Proteobacteria	Deltaproteobacteria	Myxococcales		uncultured bacterium	-	-	-	-	-	-	-	-	-	-	-	-	-	-	-	-	-	-	-	-	-	-	-	-	-
152964	0,0%	0,0%	0,0%	0,0%	0,0%	0,0%	0,0%	0,0%	0,0%	Bacteria	Proteobacteria	Deltaproteobacteria	Syntrophobacterales	Syntrophobacteraceae	Rhodococcus	-	napA	narG	nirA	-	-	nirK	norB	-	-	-	-	ureA	-	-	-	-	-	mmoX	-	laccase_Asco	laccase_Basidio	-	-	ppo
151126	0,0%	0,0%	0,0%	0,0%	0,0%	0,0%	0,0%	0,0%	0,0%	Bacteria	Cyanobacteria	4C0d-2	MLE1-12		uncultured bacterium	-	-	-	-	-	-	-	-	-	-	-	-	-	-	-	-	-	-	-	-	-	-	-	-	-
4318238	0,0%	0,0%	0,0%	0,0%	0,0%	0,0%	0,0%	0,0%	0,0%	Bacteria	Proteobacteria	Alphaproteobacteria	Caulobacterales	Caulobacteraceae	uncultured	-	-	-	-	-	-	-	-	-	-	-	-	-	-	-	-	-	-	-	-	-	-	-	lip	-
852179	0,0%	0,0%	0,0%	0,0%	0,0%	0,0%	0,0%	0,0%	0,0%	Bacteria	Proteobacteria	Betaproteobacteria	Rhodocyclales	Rhodocyclaceae	Candidatus Accumulibacter	-	-	-	-	-	-	-	-	-	-	-	-	-	-	-	-	-	-	-	-	-	-	-	-	-
136741	0,0%	0,0%	0,0%	0,0%	0,0%	0,0%	0,0%	0,0%	0,0%	Bacteria	Proteobacteria	Gammaproteobacteria	Legionellales		uncultured soil bacterium	-	-	-	-	-	-	-	-	-	-	-	-	-	-	-	-	-	-	-	-	-	-	-	-	-
585360	0,0%	0,0%	0,0%	0,0%	0,0%	0,0%	0,0%	0,0%	0,0%	Bacteria	Verrucomicrobia	Verrucomicrobiae	Verrucomicrobiales	Verrucomicrobiaceae	uncultured	-	-	-	-	-	-	-	-	-	-	-	-	-	-	-	-	-	-	-	-	-	-	-	lip	-
4480022	0,0%	0,0%	0,0%	0,0%	0,0%	0,0%	0,0%	0,0%	0,0%	Bacteria	Proteobacteria	Alphaproteobacteria	Sphingomonadales	Sphingomonadaceae	Novosphingobium	-	napA	-	-	-	-	-	norB	-	nifH	nifD	-	ureA	-	-	-	-	-	mmoX	-	laccase_Asco	laccase_Basidio	-	-	ppo
4294965	0,0%	0,0%	0,0%	0,0%	0,0%	0,0%	0,0%	0,0%	0,0%	Bacteria	Proteobacteria	Betaproteobacteria	Burkholderiales		Derxia	-	-	-	-	-	-	-	-	-	nifH	-	-	-	-	-	-	-	-	-	-	-	-	-	-	-
4373482	0,0%	0,0%	0,0%	0,0%	0,0%	0,0%	0,0%	0,0%	0,0%	Bacteria	Proteobacteria	Alphaproteobacteria	Caulobacterales	Caulobacteraceae	Brevundimonas	-	-	-	nirA	-	-	-	norB	-	nifH	-	-	-	-	-	-	-	-	mmoX	-	laccase_Asco	laccase_Basidio	-	-	ppo
286652	0,0%	0,0%	0,0%	0,0%	0,0%	0,0%	0,0%	0,0%	0,0%	Bacteria	Planctomycetes	Phycisphaerae	WD2101		uncultured bacterium	-	-	-	-	-	-	-	-	-	-	-	-	-	-	-	-	-	-	-	-	-	-	-	-	-
1638795	0,0%	0,0%	0,0%	0,0%	0,0%	0,0%	0,0%	0,0%	0,0%	Bacteria	Proteobacteria	Deltaproteobacteria	Myxococcales	Haliangiaceae	Haliangium	-	-	-	-	-	-	-	-	-	-	-	-	ureA	-	-	-	-	chb	-	-	-	-	-	-	-
699085	0,0%	0,0%	0,0%	0,0%	0,0%	0,0%	0,0%	0,0%	0,0%	Bacteria	Acidobacteria	Acidobacteriia	Acidobacteriales	Acidobacteriaceae	uncultured	cellulolytic	-	-	-	-	-	-	-	-	-	-	-	-	-	-	-	-	-	-	-	-	-	-	lip	-
680824	0,0%	0,0%	0,0%	0,0%	0,0%	0,0%	0,0%	0,0%	0,0%	Bacteria	Proteobacteria	Deltaproteobacteria			uncultured bacterium	-	-	-	-	-	-	-	-	-	-	-	-	-	-	-	-	-	-	-	-	-	-	-	-	-
4409615	0,0%	0,0%	0,0%	0,0%	0,0%	0,0%	0,0%	0,0%	0,0%	Bacteria	Proteobacteria	Deltaproteobacteria	Myxococcales	Haliangiaceae	Haliangium	-	-	-	-	-	-	-	-	-	-	-	-	ureA	-	-	-	-	chb	-	-	-	-	-	-	-
1116539	0,0%	0,0%	0,0%	0,0%	0,0%	0,0%	0,0%	0,0%	0,0%	Bacteria	Acidobacteria	Sva0725	Sva0725		Acidobacteria bacterium WY67	-	-	-	-	-	-	-	-	-	-	-	-	-	-	-	-	-	-	-	-	-	-	-	-	-
1136507	0,0%	0,0%	0,0%	0,0%	0,0%	0,0%	0,0%	0,0%	0,0%	Bacteria	Proteobacteria	Alphaproteobacteria	Sphingomonadales	Sphingomonadaceae	Novosphingobium	-	napA	-	-	-	-	-	norB	-	nifH	nifD	-	ureA	-	-	-	-	-	mmoX	-	laccase_Asco	laccase_Basidio	-	-	ppo
1083723	0,0%	0,0%	0,0%	0,0%	0,0%	0,0%	0,0%	0,0%	0,0%	Bacteria	Planctomycetes	Planctomycetia	Gemmatales	Gemmataceae	uncultured	-	-	-	-	-	-	-	-	-	-	-	-	-	-	-	-	-	-	-	-	-	-	-	lip	-
4386579	0,0%	0,0%	0,0%	0,0%	0,0%	0,0%	0,0%	0,0%	0,0%	Bacteria	Proteobacteria	Gammaproteobacteria	Legionellales	Legionellaceae	Legionella	-	-	-	-	-	nirS	-	norB	-	-	-	-	-	-	-	-	-	-	-	-	laccase_Asco	laccase_Basidio	-	-	ppo
1120485	0,0%	0,0%	0,0%	0,0%	0,0%	0,0%	0,0%	0,0%	0,0%	Bacteria	Proteobacteria	Gammaproteobacteria	Legionellales	Legionellaceae	Legionella	-	-	-	-	-	nirS	-	norB	-	-	-	-	-	-	-	-	-	-	-	-	laccase_Asco	laccase_Basidio	-	-	ppo
228186	0,0%	0,0%	0,0%	0,0%	0,0%	0,0%	0,0%	0,0%	0,0%	Bacteria	Proteobacteria	Alphaproteobacteria	Rhizobiales		Methylorosula	-	-	-	-	-	-	-	-	-	-	-	-	-	-	-	-	-	-	-	-	-	-	-	-	-
325983	0,0%	0,0%	0,0%	0,0%	0,0%	0,0%	0,0%	0,0%	0,0%	Bacteria	Planctomycetes	Phycisphaerae	Phycisphaerales		Urania-1B-19 marine sediment group	-	-	-	-	-	-	-	-	-	-	-	-	-	-	-	-	-	-	-	-	-	-	-	-	-
1012768	0,0%	0,0%	0,0%	0,0%	0,0%	0,0%	0,0%	0,0%	0,0%	Bacteria	Proteobacteria	Deltaproteobacteria	MIZ46		uncultured bacterium	-	-	-	-	-	-	-	-	-	-	-	-	-	-	-	-	-	-	-	-	-	-	-	-	-
4402421	0,0%	0,0%	0,0%	0,0%	0,0%	0,0%	0,0%	0,0%	0,0%	Bacteria	Acidobacteria	Acidobacteriia	Acidobacteriales	Koribacteraceae	uncultured	-	-	-	-	-	-	-	-	-	-	-	-	-	-	-	-	-	-	-	-	-	-	-	lip	-
607810	0,0%	0,0%	0,0%	0,0%	0,0%	0,0%	0,0%	0,0%	0,0%	Bacteria	Fusobacteria	Fusobacteriia	Fusobacteriales		uncultured	-	-	-	-	-	-	-	-	-	-	-	-	-	-	-	-	-	-	-	-	-	-	-	lip	-
4380638	0,0%	0,0%	0,0%	0,0%	0,0%	0,0%	0,0%	0,0%	0,0%	Bacteria	Proteobacteria	Alphaproteobacteria	Rickettsiales		Candidatus Captivus	-	-	-	-	-	-	-	-	-	-	-	-	-	-	-	-	-	-	-	-	-	-	-	-	-
4474485	0,0%	0,0%	0,0%	0,0%	0,0%	0,0%	0,0%	0,0%	0,0%	Bacteria	Firmicutes	Bacilli	Bacillales	Bacillaceae	Bacillus	cellulolytic	napA	narG	nirA	-	nirS	nirK	norB	nosZ	nifH	nifD	nrfA	ureA	-	-	-	-	-	mmoX	-	laccase_Asco	laccase_Basidio	-	-	ppo
4361147	0,0%	0,0%	0,0%	0,0%	0,0%	0,0%	0,0%	0,0%	0,0%	Bacteria	Proteobacteria	Alphaproteobacteria	Sphingomonadales	Sphingomonadaceae	Sphingomonas	-	napA	narG	nirA	-	-	nirK	norB	-	nifH	nifD	-	ureA	-	-	-	-	chb	mmoX	-	laccase_Asco	laccase_Basidio	-	-	ppo
529067	0,0%	0,0%	0,0%	0,0%	0,0%	0,0%	0,0%	0,0%	0,0%	Bacteria	Planctomycetes	Planctomycetia	Pirellulales	Pirellulaceae	Pir4 lineage	-	-	-	-	-	-	-	-	-	-	-	-	-	-	-	-	-	-	-	-	-	-	-	-	-
4421534	0,0%	0,0%	0,0%	0,0%	0,0%	0,1%	0,0%	0,0%	0,0%	Bacteria	Proteobacteria	Betaproteobacteria	Burkholderiales	Comamonadaceae	Leptothrix	-	napA	-	-	-	nirS	-	norB	nosZ	nifH	nifD	-	ureA	-	-	-	-	-	mmoX	-	-	-	-	-	-
776570	0,0%	0,0%	0,0%	0,0%	0,0%	0,0%	0,0%	0,0%	0,0%	Bacteria	Verrucomicrobia	Opitutae	Opitutales	Opitutaceae	Opitutus	-	napA	-	nirA	-	-	-	-	nosZ	-	-	nrfA	ureA	-	-	-	-	-	-	-	-	laccase_Basidio	-	-	-
4340146	0,0%	0,0%	0,0%	0,0%	0,0%	0,0%	0,0%	0,1%	0,0%	Bacteria	Proteobacteria	Betaproteobacteria	Burkholderiales	Comamonadaceae	Variovorax	-	napA	narG	nirA	-	-	-	norB	-	-	-	-	ureA	-	-	-	-	-	mmoX	-	-	-	-	-	-
344331	0,0%	0,0%	0,0%	0,0%	0,0%	0,0%	0,0%	0,0%	0,0%	Bacteria	Planctomycetes	Planctomycetia	Gemmatales	Isosphaeraceae	Isosphaera	-	napA	-	-	-	-	-	-	-	-	-	-	-	-	-	-	-	-	-	-	-	-	-	-	-
1119551	0,0%	0,0%	0,0%	0,0%	0,0%	0,0%	0,0%	0,0%	0,0%	Bacteria	Proteobacteria	Betaproteobacteria	Burkholderiales	Comamonadaceae	Rhizobacter	-	napA	narG	nirA	-	-	-	norB	-	-	-	-	ureA	-	-	-	-	-	-	-	-	-	-	-	-
1102974	0,0%	0,0%	0,0%	0,0%	0,0%	0,0%	0,0%	0,0%	0,0%	Bacteria	Armatimonadetes	Chthonomonadetes	Chthonomonadales	Chthonomonadaceae	Chthonomonas	-	-	-	-	-	-	-	-	-	-	-	-	-	-	-	-	-	-	-	-	laccase_Asco	laccase_Basidio	-	-	-
3608294	0,0%	0,0%	0,0%	0,0%	0,0%	0,0%	0,0%	0,0%	0,0%	Bacteria	Proteobacteria	Deltaproteobacteria	Myxococcales		uncultured bacterium	-	-	-	-	-	-	-	-	-	-	-	-	-	-	-	-	-	-	-	-	-	-	-	-	-
4445096	0,0%	0,0%	0,0%	0,0%	0,0%	0,0%	0,0%	0,0%	0,0%	Bacteria	Proteobacteria	Betaproteobacteria	Burkholderiales	Oxalobacteraceae	Massilia	-	napA	narG	-	-	-	-	norB	nosZ	-	-	-	ureA	-	-	-	-	chb	mmoX	-	laccase_Asco	laccase_Basidio	-	-	ppo
863942	0,0%	0,0%	0,0%	0,0%	0,0%	0,0%	0,0%	0,1%	0,0%	Bacteria	Proteobacteria	Alphaproteobacteria	Rhizobiales		uncultured bacterium	-	-	-	-	-	-	-	-	-	-	-	-	-	-	-	-	-	-	-	-	-	-	-	-	-
4364519	0,0%	0,0%	0,0%	0,0%	0,0%	0,0%	0,0%	0,0%	0,0%	Bacteria	Proteobacteria	Deltaproteobacteria	MIZ46		uncultured bacterium	-	-	-	-	-	-	-	-	-	-	-	-	-	-	-	-	-	-	-	-	-	-	-	-	-
568461	0,0%	0,0%	0,0%	0,0%	0,0%	0,0%	0,0%	0,0%	0,0%	Bacteria	Actinobacteria	Thermoleophilia	Gaiellales	Gaiellaceae	uncultured Conexibacter sp.	-	-	-	-	-	-	-	-	-	-	-	-	-	-	-	-	-	-	-	-	-	-	-	-	-
109391	0,0%	0,0%	0,0%	0,0%	0,0%	0,0%	0,0%	0,0%	0,0%	Bacteria	Proteobacteria	Betaproteobacteria	Burkholderiales	Comamonadaceae	Undibacterium	-	-	-	-	-	-	-	-	-	-	-	-	-	-	-	-	-	-	-	-	-	-	-	-	-
625186	0,0%	0,0%	0,0%	0,0%	0,0%	0,0%	0,0%	0,0%	0,0%	Bacteria	Proteobacteria	Gammaproteobacteria	Chromatiales	Chromatiaceae	Thiocystis	-	napA	-	-	-	-	-	norB	-	nifH	nifD	-	ureA	dsrA	dsrB	-	-	-	-	-	-	-	-	-	-
1135798	0,0%	0,0%	0,0%	0,0%	0,0%	0,0%	0,0%	0,0%	0,0%	Bacteria	Proteobacteria	Alphaproteobacteria	Sphingomonadales	Sphingomonadaceae	Novosphingobium	-	napA	-	-	-	-	-	norB	-	nifH	nifD	-	ureA	-	-	-	-	-	mmoX	-	laccase_Asco	laccase_Basidio	-	-	ppo
340360	0,0%	0,0%	0,0%	0,0%	0,0%	0,0%	0,0%	0,0%	0,0%	Bacteria	Proteobacteria	Betaproteobacteria	Methylophilales	Methylophilaceae	uncultured	-	-	-	-	-	-	-	-	-	-	-	-	-	-	-	-	-	-	-	-	-	-	-	lip	-
4424415	0,0%	0,0%	0,0%	0,0%	0,0%	0,0%	0,0%	0,0%	0,0%	Bacteria	Proteobacteria	Betaproteobacteria	Rhodocyclales	Rhodocyclaceae	Sulfuritalea	-	napA	-	-	-	nirS	-	-	nosZ	-	-	-	-	dsrA	dsrB	-	-	-	-	-	-	-	-	-	-
560957	0,0%	0,0%	0,0%	0,0%	0,0%	0,0%	0,0%	0,0%	0,0%	Bacteria	Planctomycetes	Planctomycetia	Pirellulales	Pirellulaceae	uncultured	-	-	-	-	-	-	-	-	-	-	-	-	-	-	-	-	-	-	-	-	-	-	-	lip	-
4399333	0,0%	0,0%	0,0%	0,0%	0,0%	0,0%	0,0%	0,0%	0,0%	Bacteria	Proteobacteria	Betaproteobacteria	Burkholderiales		Limnobacter	-	napA	-	-	-	-	-	-	-	-	-	-	-	-	-	-	-	-	mmoX	-	-	laccase_Basidio	-	-	-
4302012	0,0%	0,0%	0,0%	0,0%	0,0%	0,0%	0,0%	0,0%	0,0%	Bacteria	Proteobacteria	Alphaproteobacteria	Rhodobacterales	Rhodobacteraceae	Amaricoccus	-	-	-	-	-	-	-	-	-	-	-	-	-	-	-	-	-	-	-	-	-	-	-	-	-
4343659	0,0%	0,0%	0,0%	0,0%	0,0%	0,0%	0,0%	0,0%	0,0%	Bacteria	Proteobacteria	Betaproteobacteria	Burkholderiales	Comamonadaceae	uncultured	-	-	-	-	-	-	-	-	-	-	-	-	-	-	-	-	-	-	-	-	-	-	-	lip	-
557255	0,0%	0,0%	0,0%	0,0%	0,0%	0,0%	0,0%	0,0%	0,0%	Bacteria	Proteobacteria	Betaproteobacteria			uncultured	-	-	-	-	-	-	-	-	-	-	-	-	-	-	-	-	-	-	-	-	-	-	-	lip	-
4358255	0,0%	0,0%	0,0%	0,0%	0,0%	0,0%	0,0%	0,0%	0,0%	Bacteria	Proteobacteria	Deltaproteobacteria	Myxococcales		uncultured delta proteobacterium	-	-	-	-	-	-	-	-	-	-	-	-	-	-	-	-	-	-	-	-	-	-	-	-	-
669019	0,0%	0,0%	0,0%	0,0%	0,0%	0,0%	0,0%	0,0%	0,0%	Bacteria	Planctomycetes	Planctomycetia	Gemmatales	Gemmataceae	Gemmata	-	-	-	-	-	-	-	-	-	-	-	-	-	-	-	-	-	-	-	-	-	-	-	-	-
513398	0,0%	0,0%	0,0%	0,0%	0,0%	0,0%	0,0%	0,0%	0,0%	Bacteria	Bacteroidetes	[Saprospirae]	[Saprospirales]	Chitinophagaceae	Segetibacter	-	-	-	-	-	-	-	-	-	-	-	-	-	-	-	-	-	-	-	-	-	-	-	-	-
3474521	0,0%	0,0%	0,0%	0,0%	0,0%	0,0%	0,0%	0,0%	0,0%	Bacteria	Proteobacteria	Deltaproteobacteria	Bdellovibrionales	Bdellovibrionaceae	Bdellovibrio	-	-	-	-	-	-	nirK	norB	-	-	-	nrfA	-	-	-	-	-	-	-	-	laccase_Asco	laccase_Basidio	-	-	ppo
830077	0,0%	0,0%	0,0%	0,0%	0,0%	0,0%	0,0%	0,0%	0,0%	Bacteria	Proteobacteria	Alphaproteobacteria	Rhizobiales	Beijerinckiaceae	Meganema	-	-	-	-	-	-	-	-	-	-	-	-	-	-	-	-	-	-	-	-	-	-	-	-	-
826298	0,0%	0,0%	0,0%	0,0%	0,0%	0,0%	0,0%	0,0%	0,0%	Bacteria	Proteobacteria	Gammaproteobacteria	Aeromonadales	Aeromonadaceae	uncultured bacterium	-	-	-	-	-	-	-	-	-	-	-	-	-	-	-	-	-	-	-	-	-	-	-	-	-
684785	0,0%	0,0%	0,0%	0,0%	0,0%	0,0%	0,0%	0,0%	0,0%	Bacteria	Proteobacteria	Alphaproteobacteria	Rhizobiales		Devosia	-	napA	-	-	-	-	nirK	-	-	nifH	-	-	ureA	-	-	-	-	chb	-	-	laccase_Asco	laccase_Basidio	-	-	ppo
4427546	0,0%	0,0%	0,0%	0,0%	0,0%	0,0%	0,0%	0,0%	0,0%	Bacteria	Proteobacteria	Alphaproteobacteria	Sphingomonadales	Sphingomonadaceae	Novosphingobium	-	napA	-	-	-	-	-	norB	-	nifH	nifD	-	ureA	-	-	-	-	-	mmoX	-	laccase_Asco	laccase_Basidio	-	-	ppo
234041	0,0%	0,0%	0,0%	0,0%	0,0%	0,0%	0,0%	0,0%	0,0%	Bacteria	Gemmatimonadetes	Gemmatimonadetes	Gemmatimonadales	Gemmatimonadaceae	Gemmatimonas	-	napA	-	-	-	-	nirK	norB	nosZ	-	-	nrfA	-	dsrA	dsrB	-	-	chb	-	-	-	-	-	-	-
368986	0,0%	0,0%	0,0%	0,0%	0,0%	0,0%	0,0%	0,0%	0,0%	Bacteria	Proteobacteria	Alphaproteobacteria	Rhodospirillales	Acetobacteraceae	uncultured	-	-	-	-	-	-	-	-	-	-	-	-	-	-	-	-	-	-	-	-	-	-	-	lip	-
5784	0,0%	0,0%	0,0%	0,0%	0,0%	0,0%	0,0%	0,0%	0,0%	Bacteria	Proteobacteria	Alphaproteobacteria	Rhodobacterales	Rhodobacteraceae	Rhodobacter	-	napA	-	nirA	-	nirS	nirK	norB	nosZ	nifH	nifD	-	ureA	-	-	SoxB	-	-	mmoX	-	laccase_Asco	laccase_Basidio	-	-	ppo
266536	0,0%	0,0%	0,0%	0,0%	0,0%	0,0%	0,0%	0,0%	0,0%	Bacteria	Proteobacteria	Alphaproteobacteria	Rhizobiales		uncultured alpha proteobacterium	-	-	-	-	-	-	-	-	-	-	-	-	-	-	-	-	-	-	-	-	-	-	-	-	-
4454153	0,0%	0,0%	0,0%	0,0%	0,0%	0,0%	0,0%	0,0%	0,0%	Bacteria	Actinobacteria	Actinobacteria	Actinomycetales	Microbacteriaceae	Pseudoclavibacter	cellulolytic	-	-	-	-	-	-	-	-	-	-	-	-	-	-	-	-	-	-	-	-	-	-	-	-
978841	0,0%	0,0%	0,0%	0,0%	0,0%	0,0%	0,0%	0,0%	0,0%	Bacteria	Actinobacteria	Actinobacteria	Actinomycetales		Nakamurella	-	napA	narG	-	-	-	-	-	-	-	-	-	ureA	-	-	-	-	-	-	-	-	laccase_Basidio	-	-	-
819400	0,0%	0,0%	0,0%	0,0%	0,0%	0,0%	0,0%	0,0%	0,0%	Bacteria	Proteobacteria	Betaproteobacteria	Burkholderiales	Comamonadaceae	uncultured	-	-	-	-	-	-	-	-	-	-	-	-	-	-	-	-	-	-	-	-	-	-	-	lip	-
4454408	0,0%	0,0%	0,0%	0,0%	0,0%	0,0%	0,0%	0,0%	0,0%	Bacteria	Bacteroidetes	Bacteroidia	Bacteroidales		uncultured bacterium	-	-	-	-	-	-	-	-	-	-	-	-	-	-	-	-	-	-	-	-	-	-	-	-	-
832166	0,0%	0,0%	0,0%	0,0%	0,0%	0,0%	0,0%	0,0%	0,0%	Bacteria	Proteobacteria	Betaproteobacteria	Methylophilales	Methylophilaceae	uncultured	-	-	-	-	-	-	-	-	-	-	-	-	-	-	-	-	-	-	-	-	-	-	-	lip	-
3571344	0,0%	0,0%	0,0%	0,0%	0,0%	0,0%	0,0%	0,0%	0,0%	Bacteria	Gemmatimonadetes	Gemm-2			uncultured bacterium	-	-	-	-	-	-	-	-	-	-	-	-	-	-	-	-	-	-	-	-	-	-	-	-	-
4396488	0,0%	0,0%	0,0%	0,0%	0,0%	0,0%	0,0%	0,0%	0,0%	Bacteria	TM6	SJA-4			uncultured bacterium	-	-	-	-	-	-	-	-	-	-	-	-	-	-	-	-	-	-	-	-	-	-	-	-	-
286499	0,0%	0,0%	0,0%	0,0%	0,0%	0,0%	0,0%	0,0%	0,0%	Archaea	Euryarchaeota	Thermoplasmata	E2	Marine group III	uncultured marine group III euryarchaeote	-	-	-	-	-	-	-	-	-	-	-	-	-	-	-	-	-	-	-	-	-	-	-	-	-
141189	0,0%	0,0%	0,0%	0,0%	0,0%	0,1%	0,0%	0,0%	0,0%	Bacteria	Spirochaetes	Spirochaetes	Spirochaetales	Spirochaetaceae	Spirochaeta	-	-	-	nirA	-	-	-	-	-	nifH	nifD	-	-	-	-	-	-	-	-	-	-	-	-	-	-
551109	0,0%	0,0%	0,0%	0,0%	0,0%	0,0%	0,0%	0,0%	0,0%	Bacteria	Proteobacteria	Alphaproteobacteria	Caulobacterales	Caulobacteraceae	Asticcacaulis	-	napA	narG	nirA	-	-	-	-	-	-	-	-	-	-	-	-	-	chb	-	-	laccase_Asco	laccase_Basidio	-	-	ppo
218032	0,0%	0,0%	0,0%	0,0%	0,0%	0,0%	0,0%	0,1%	0,0%	Bacteria	Verrucomicrobia	[Pedosphaerae]	[Pedosphaerales]		uncultured bacterium	-	-	-	-	-	-	-	-	-	-	-	-	-	-	-	-	-	-	-	-	-	-	-	-	-
4430763	0,0%	0,0%	0,0%	0,0%	0,0%	0,0%	0,0%	0,0%	0,0%	Bacteria	Proteobacteria	Betaproteobacteria	Methylophilales	Methylophilaceae	Methylotenera	-	napA	-	nirA	-	-	nirK	norB	-	-	-	-	ureA	-	-	-	-	-	-	-	-	-	-	-	-
994191	0,0%	0,0%	0,0%	0,0%	0,0%	0,0%	0,0%	0,0%	0,1%	Bacteria	Verrucomicrobia	[Spartobacteria]	[Chthoniobacterales]	[Chthoniobacteraceae]	Chthoniobacter	-	napA	-	-	-	-	nirK	-	-	-	-	nrfA	ureA	-	-	-	-	-	-	-	-	-	-	-	-
6118	0,0%	0,0%	0,0%	0,0%	0,0%	0,0%	0,0%	0,0%	0,0%	Bacteria	Proteobacteria	Alphaproteobacteria	Rickettsiales	Rickettsiaceae	Wolbachia	-	-	-	-	-	-	-	-	-	-	-	-	-	-	-	-	-	-	-	-	-	-	-	-	-
4470101	0,0%	0,0%	0,0%	0,0%	0,0%	0,0%	0,0%	0,0%	0,0%	Bacteria	Proteobacteria	Betaproteobacteria			uncultured	-	-	-	-	-	-	-	-	-	-	-	-	-	-	-	-	-	-	-	-	-	-	-	lip	-
536765	0,0%	0,0%	0,0%	0,0%	0,0%	0,0%	0,0%	0,0%	0,1%	Bacteria	Bacteroidetes	[Saprospirae]	[Saprospirales]	Chitinophagaceae	Taibaiella	-	-	-	-	-	-	-	-	-	-	-	-	-	-	-	-	-	-	-	-	-	-	-	-	-
4393057	0,0%	0,0%	0,0%	0,0%	0,0%	0,0%	0,0%	0,0%	0,0%	Bacteria	Proteobacteria	Alphaproteobacteria	Sphingomonadales	Sphingomonadaceae	Novosphingobium	-	napA	-	-	-	-	-	norB	-	nifH	nifD	-	ureA	-	-	-	-	-	mmoX	-	laccase_Asco	laccase_Basidio	-	-	ppo
4419214	0,0%	0,0%	0,0%	0,0%	0,0%	0,0%	0,0%	0,0%	0,0%	Bacteria	Proteobacteria	Deltaproteobacteria	Myxococcales		uncultured bacterium	-	-	-	-	-	-	-	-	-	-	-	-	-	-	-	-	-	-	-	-	-	-	-	-	-
11431	0,0%	0,0%	0,0%	0,0%	0,0%	0,0%	0,0%	0,0%	0,0%	Bacteria	Actinobacteria	Actinobacteria	Actinomycetales		uncultured bacterium	-	-	-	-	-	-	-	-	-	-	-	-	-	-	-	-	-	-	-	-	-	-	-	-	-
101692	0,0%	0,0%	0,0%	0,0%	0,0%	0,0%	0,0%	0,0%	0,0%	Bacteria	Proteobacteria	Alphaproteobacteria	Rickettsiales		Candidatus Captivus	-	-	-	-	-	-	-	-	-	-	-	-	-	-	-	-	-	-	-	-	-	-	-	-	-
4475864	0,0%	0,0%	0,0%	0,0%	0,0%	0,0%	0,0%	0,0%	0,0%	Bacteria	Proteobacteria	Betaproteobacteria	Burkholderiales	Comamonadaceae	uncultured	-	-	-	-	-	-	-	-	-	-	-	-	-	-	-	-	-	-	-	-	-	-	-	lip	-
1144575	0,0%	0,0%	0,0%	0,0%	0,0%	0,0%	0,0%	0,0%	0,0%	Bacteria	Proteobacteria	Deltaproteobacteria	Myxococcales		uncultured bacterium	-	-	-	-	-	-	-	-	-	-	-	-	-	-	-	-	-	-	-	-	-	-	-	-	-
909520	0,0%	0,0%	0,0%	0,0%	0,0%	0,0%	0,0%	0,0%	0,0%	Bacteria	Proteobacteria	Deltaproteobacteria	Myxococcales	Myxococcaceae	Anaeromyxobacter	-	napA	narG	nirA	-	nirS	-	norB	nosZ	nifH	nifD	nrfA	ureA	-	-	SoxB	-	-	-	-	-	laccase_Basidio	-	-	-
1109260	0,0%	0,0%	0,0%	0,0%	0,0%	0,0%	0,0%	0,0%	0,0%	Bacteria	Bacteroidetes	Sphingobacteriia	Sphingobacteriales	Sphingobacteriaceae	Mucilaginibacter	-	-	-	-	-	-	-	-	-	-	-	-	-	-	-	-	-	-	-	-	-	-	-	-	-
153512	0,0%	0,0%	0,0%	0,0%	0,0%	0,0%	0,0%	0,0%	0,0%	Bacteria	Proteobacteria	Betaproteobacteria	Burkholderiales	Alcaligenaceae	uncultured	cellulolytic	-	-	-	-	-	-	-	-	-	-	-	-	-	-	-	-	-	-	-	-	-	-	lip	-
1089636	0,0%	0,0%	0,0%	0,0%	0,0%	0,0%	0,0%	0,0%	0,0%	Bacteria	Firmicutes	Clostridia	Clostridiales	Ruminococcaceae	Incertae Sedis	cellulolytic	-	-	-	-	-	-	-	-	-	-	-	-	-	-	-	-	-	-	-	-	-	-	-	-
526316	0,0%	0,0%	0,0%	0,0%	0,0%	0,0%	0,0%	0,0%	0,0%	Bacteria	Planctomycetes	Planctomycetia	Planctomycetales	Planctomycetaceae	Planctomyces	-	napA	-	-	-	-	-	norB	-	-	-	nrfA	-	-	-	-	-	-	-	-	-	-	-	-	-
4383656	0,0%	0,0%	0,0%	0,0%	0,0%	0,0%	0,0%	0,0%	0,0%	Bacteria	Verrucomicrobia	[Pedosphaerae]	[Pedosphaerales]	Ellin515	uncultured bacterium	-	-	-	-	-	-	-	-	-	-	-	-	-	-	-	-	-	-	-	-	-	-	-	-	-
1013213	0,0%	0,0%	0,0%	0,0%	0,0%	0,0%	0,0%	0,0%	0,0%	Bacteria	Proteobacteria	Betaproteobacteria	Burkholderiales	Comamonadaceae	Variovorax	-	napA	narG	nirA	-	-	-	norB	-	-	-	-	ureA	-	-	-	-	-	mmoX	-	-	-	-	-	-
578443	0,0%	0,0%	0,0%	0,0%	0,0%	0,0%	0,0%	0,0%	0,0%	Bacteria	Acidobacteria	Solibacteres	Solibacterales		Bryobacter	-	-	-	-	-	-	-	-	-	-	-	-	-	-	-	-	-	-	-	-	-	-	-	-	-
967505	0,0%	0,0%	0,0%	0,0%	0,0%	0,1%	0,0%	0,0%	0,0%	Bacteria	Proteobacteria	Deltaproteobacteria	Bdellovibrionales	Bdellovibrionaceae	Bdellovibrio	-	-	-	-	-	-	nirK	norB	-	-	-	nrfA	-	-	-	-	-	-	-	-	laccase_Asco	laccase_Basidio	-	-	ppo
4343193	0,0%	0,0%	0,0%	0,0%	0,0%	0,0%	0,0%	0,0%	0,0%	Bacteria	Proteobacteria	Alphaproteobacteria	Rhodospirillales	Acetobacteraceae	Roseomonas	-	napA	-	-	-	-	-	-	-	-	nifD	-	ureA	-	-	-	-	-	-	-	-	laccase_Basidio	-	-	-
7976	0,0%	0,0%	0,0%	0,0%	0,0%	0,0%	0,0%	0,0%	0,0%	Bacteria	Proteobacteria	Gammaproteobacteria	Legionellales	Legionellaceae	Legionella	-	-	-	-	-	nirS	-	norB	-	-	-	-	-	-	-	-	-	-	-	-	laccase_Asco	laccase_Basidio	-	-	ppo
45556	0,0%	0,0%	0,0%	0,0%	0,0%	0,0%	0,0%	0,0%	0,0%	Bacteria	Proteobacteria	Deltaproteobacteria	Myxococcales		uncultured bacterium	-	-	-	-	-	-	-	-	-	-	-	-	-	-	-	-	-	-	-	-	-	-	-	-	-
154435	0,0%	0,0%	0,0%	0,0%	0,0%	0,0%	0,0%	0,0%	0,0%	Bacteria	Proteobacteria	Gammaproteobacteria	Xanthomonadales	Sinobacteraceae	uncultured proteobacterium	-	-	-	-	-	-	-	-	-	-	-	-	-	-	-	-	-	-	-	-	-	-	-	-	-
4395692	0,0%	0,0%	0,0%	0,0%	0,0%	0,0%	0,0%	0,0%	0,0%	Bacteria	Proteobacteria	Alphaproteobacteria	Rhizobiales	Hyphomicrobiaceae	uncultured	-	-	-	-	-	-	-	-	-	-	-	-	-	-	-	-	-	-	-	-	-	-	-	lip	-
52421	0,0%	0,0%	0,0%	0,0%	0,0%	0,0%	0,0%	0,0%	0,1%	Bacteria	Verrucomicrobia	Verrucomicrobiae	Verrucomicrobiales	Verrucomicrobiaceae	uncultured	-	-	-	-	-	-	-	-	-	-	-	-	-	-	-	-	-	-	-	-	-	-	-	lip	-
1109867	0,0%	0,0%	0,0%	0,0%	0,0%	0,0%	0,0%	0,0%	0,0%	Bacteria	Verrucomicrobia	[Pedosphaerae]	[Pedosphaerales]	auto67_4W	uncultured bacterium	-	-	-	-	-	-	-	-	-	-	-	-	-	-	-	-	-	-	-	-	-	-	-	-	-
2792170	0,0%	0,0%	0,0%	0,0%	0,0%	0,0%	0,0%	0,0%	0,0%	Bacteria	BHI80-139				uncultured Geobacter sp.	-	-	-	-	-	-	-	-	-	-	-	-	-	-	-	-	-	-	-	-	-	-	-	-	-
741139	0,0%	0,0%	0,0%	0,0%	0,0%	0,0%	0,0%	0,0%	0,0%	Bacteria	Firmicutes	Clostridia	Clostridiales	Clostridiaceae	Clostridium sensu stricto 2	cellulolytic	-	-	-	-	-	-	-	-	-	-	-	-	-	-	-	-	-	-	-	-	-	-	-	-
582668	0,0%	0,0%	0,0%	0,0%	0,0%	0,0%	0,0%	0,0%	0,0%	Bacteria	Cyanobacteria	4C0d-2	MLE1-12		uncultured bacterium	-	-	-	-	-	-	-	-	-	-	-	-	-	-	-	-	-	-	-	-	-	-	-	-	-
4390723	0,0%	0,0%	0,0%	0,0%	0,0%	0,0%	0,0%	0,0%	0,0%	Bacteria	Actinobacteria	Actinobacteria	Actinomycetales	Mycobacteriaceae	Mycobacterium	-	napA	narG	nirA	-	-	-	norB	-	-	-	-	ureA	-	-	-	amoA_AOB	-	mmoX	pmoA	laccase_Asco	laccase_Basidio	-	-	ppo
944401	0,0%	0,0%	0,0%	0,0%	0,0%	0,0%	0,0%	0,0%	0,0%	Bacteria	Bacteroidetes	Sphingobacteriia	Sphingobacteriales	Sphingobacteriaceae	Pedobacter	-	napA	-	nirA	-	-	-	-	nosZ	-	-	-	ureA	-	-	-	-	chb	-	-	laccase_Asco	laccase_Basidio	-	-	ppo
5162	0,0%	0,0%	0,0%	0,0%	0,0%	0,0%	0,0%	0,0%	0,0%	Bacteria	Proteobacteria	Alphaproteobacteria	Rhizobiales	Rhizobiaceae	Rhizobium	-	napA	narG	nirA	-	-	nirK	norB	nosZ	nifH	nifD	nrfA	ureA	-	-	-	-	chb	mmoX	-	laccase_Asco	laccase_Basidio	-	-	ppo
742110	0,0%	0,0%	0,0%	0,0%	0,0%	0,0%	0,0%	0,0%	0,1%	Bacteria	Acidobacteria	Acidobacteriia	Acidobacteriales	Acidobacteriaceae	Granulicella	cellulolytic	napA	-	-	-	-	-	-	-	-	-	-	-	-	-	-	-	-	-	-	laccase_Asco	laccase_Basidio	-	-	ppo
4453998	0,0%	0,0%	0,0%	0,0%	0,0%	0,0%	0,0%	0,0%	0,0%	Bacteria	Proteobacteria	Betaproteobacteria	Burkholderiales	Comamonadaceae	Pelomonas	-	napA	narG	nirA	-	nirS	-	norB	nosZ	nifH	-	-	ureA	-	-	-	-	-	-	-	-	-	-	-	-
1110169	0,0%	0,0%	0,0%	0,0%	0,0%	0,0%	0,0%	0,0%	0,0%	Bacteria	Actinobacteria	Actinobacteria	Actinomycetales		Kineosporia	-	-	-	-	-	-	-	-	-	-	-	-	-	-	-	-	-	-	-	-	-	-	-	-	-
827453	0,0%	0,0%	0,0%	0,0%	0,0%	0,0%	0,0%	0,0%	0,0%	Bacteria	Acidobacteria	Acidobacteriia	Acidobacteriales	Acidobacteriaceae	uncultured bacterium	cellulolytic	-	-	-	-	-	-	-	-	-	-	-	-	-	-	-	-	-	-	-	-	-	-	-	-
21368	0,0%	0,0%	0,0%	0,0%	0,0%	0,0%	0,0%	0,0%	0,0%	Bacteria	Proteobacteria	Betaproteobacteria	SBla14		uncultured	-	-	-	-	-	-	-	-	-	-	-	-	-	-	-	-	-	-	-	-	-	-	-	lip	-
246446	0,0%	0,0%	0,0%	0,0%	0,0%	0,0%	0,0%	0,0%	0,0%	Bacteria	Cyanobacteria	4C0d-2	MLE1-12		uncultured bacterium	-	-	-	-	-	-	-	-	-	-	-	-	-	-	-	-	-	-	-	-	-	-	-	-	-
278511	0,0%	0,0%	0,0%	0,0%	0,0%	0,0%	0,0%	0,0%	0,0%	Bacteria	Acidobacteria	DA052	Ellin6513		uncultured bacterium	-	-	-	-	-	-	-	-	-	-	-	-	-	-	-	-	-	-	-	-	-	-	-	-	-

4479548	0,0%	0,0%	0,0%	0,0%	0,0%	0,0%	0,0%	0,0%	0,0%	Bacteria	Proteobacteria	Betaproteobacteria	Neisseriales	Neisseriaceae	Chromobacterium	-	napA	narG	-	-	nirS	nirK	norB	-	-	-	-	-	-	-	-	-	chb	-	-	laccase_Asco	laccase_Basidio	-	-	ppo
1145649	0,0%	0,0%	0,0%	0,0%	0,0%	0,0%	0,0%	0,0%	0,1%	Bacteria	Acidobacteria	[Chloracidobacteria]	RB41	Ellin6075	Blastocatella	-	-	-	-	-	-	-	-	-	-	-	-	-	-	-	-	-	-	-	-	-	-	-	-	-
202662	0,0%	0,0%	0,0%	0,0%	0,0%	0,0%	0,0%	0,0%	0,0%	Bacteria	Proteobacteria	Betaproteobacteria	Burkholderiales	Comamonadaceae	Rhizobacter	-	napA	narG	nirA	-	-	-	norB	-	-	-	-	ureA	-	-	-	-	-	-	-	-	-	-	-	-
135498	0,0%	0,0%	0,0%	0,0%	0,0%	0,0%	0,0%	0,0%	0,1%	Bacteria	Proteobacteria	Alphaproteobacteria	Rhizobiales		Defluviimonas	-	napA	narG	-	-	-	-	-	-	-	-	-	ureA	-	-	-	-	-	-	-	-	laccase_Basidio	-	-	ppo
1117255	0,0%	0,0%	0,0%	0,0%	0,0%	0,0%	0,0%	0,0%	0,0%	Bacteria	Proteobacteria	Betaproteobacteria	Burkholderiales	Comamonadaceae	uncultured	-	-	-	-	-	-	-	-	-	-	-	-	-	-	-	-	-	-	-	-	-	-	-	lip	-
4314933	0,0%	0,0%	0,0%	0,0%	0,0%	0,0%	0,0%	0,0%	0,0%	Bacteria	Acidobacteria	Acidobacteriia	Acidobacteriales	Koribacteraceae	uncultured	-	-	-	-	-	-	-	-	-	-	-	-	-	-	-	-	-	-	-	-	-	-	-	lip	-
362561	0,0%	0,0%	0,0%	0,0%	0,0%	0,0%	0,0%	0,0%	0,0%	Bacteria	Acidobacteria				uncultured Acidobacteria bacterium	-	-	-	-	-	-	-	-	-	-	-	-	-	-	-	-	-	-	-	-	-	-	-	-	-
4328978	0,0%	0,0%	0,0%	0,0%	0,0%	0,0%	0,0%	0,0%	0,0%	Bacteria	Proteobacteria	Alphaproteobacteria	Caulobacterales	Caulobacteraceae	Caulobacter	-	napA	narG	nirA	-	-	nirK	norB	-	-	-	-	-	-	-	-	-	chb	-	-	laccase_Asco	laccase_Basidio	-	-	ppo
167950	0,0%	0,0%	0,0%	0,0%	0,0%	0,0%	0,0%	0,0%	0,0%	Bacteria	Proteobacteria	Gammaproteobacteria	Enterobacteriales	Enterobacteriaceae	Enterobacter	cellulolytic	napA	narG	-	-	-	-	-	-	nifH	nifD	nrfA	ureA	-	-	-	-	chb	-	-	laccase_Asco	laccase_Basidio	-	-	ppo
3219861	0,0%	0,0%	0,0%	0,0%	0,0%	0,0%	0,0%	0,0%	0,0%	Bacteria	Proteobacteria	Gammaproteobacteria	Methylococcales	Crenotrichaceae	Crenothrix	-	-	-	-	-	-	-	-	-	-	-	-	-	-	-	-	-	-	-	pmoA	-	-	-	-	-
252012	0,0%	0,0%	0,0%	0,0%	0,0%	0,0%	0,0%	0,0%	0,0%	Bacteria	Proteobacteria	Gammaproteobacteria	Xanthomonadales	Sinobacteraceae	uncultured proteobacterium	-	-	-	-	-	-	-	-	-	-	-	-	-	-	-	-	-	-	-	-	-	-	-	-	-
143820	0,0%	0,0%	0,0%	0,0%	0,0%	0,0%	0,0%	0,0%	0,0%	Bacteria	Proteobacteria	Alphaproteobacteria	Rickettsiales		Candidatus Captivus	-	-	-	-	-	-	-	-	-	-	-	-	-	-	-	-	-	-	-	-	-	-	-	-	-
4457992	0,0%	0,0%	0,0%	0,0%	0,0%	0,0%	0,0%	0,0%	0,0%	Bacteria	Proteobacteria				uncultured alpha proteobacterium	-	-	-	-	-	-	-	-	-	-	-	-	-	-	-	-	-	-	-	-	-	-	-	-	-
823916	0,0%	0,0%	0,0%	0,0%	0,0%	0,0%	0,0%	0,0%	0,0%	Bacteria	Proteobacteria	Gammaproteobacteria	Pseudomonadales	Moraxellaceae	Enhydrobacter	-	napA	narG	nirA	-	-	-	-	-	-	-	-	ureA	-	-	-	-	-	-	-	-	laccase_Basidio	-	-	-
4399780	0,0%	0,0%	0,0%	0,0%	0,0%	0,0%	0,0%	0,0%	0,1%	Bacteria	Proteobacteria	Alphaproteobacteria	Rhodospirillales	Rhodospirillaceae	Azospirillum	-	napA	-	nirA	-	nirS	nirK	norB	nosZ	nifH	nifD	-	ureA	-	-	-	-	-	mmoX	-	laccase_Asco	laccase_Basidio	-	-	ppo
775724	0,0%	0,0%	0,0%	0,0%	0,0%	0,0%	0,0%	0,0%	0,0%	Bacteria	Proteobacteria	Gammaproteobacteria	Methylococcales	Crenotrichaceae	Methylomonas	-	napA	-	nirA	-	nirS	nirK	norB	-	nifH	nifD	-	ureA	-	-	-	amoA_AOB	-	mmoX	pmoA	laccase_Asco	laccase_Basidio	-	-	ppo
4385099	0,0%	0,0%	0,0%	0,0%	0,0%	0,0%	0,0%	0,0%	0,0%	Bacteria	Verrucomicrobia	[Pedosphaerae]	[Pedosphaerales]		uncultured bacterium	-	-	-	-	-	-	-	-	-	-	-	-	-	-	-	-	-	-	-	-	-	-	-	-	-
4425446	0,0%	0,0%	0,0%	0,0%	0,0%	0,0%	0,0%	0,0%	0,0%	Bacteria	Proteobacteria	Alphaproteobacteria	Sphingomonadales	Sphingomonadaceae	Blastomonas	-	napA	-	-	-	-	-	-	-	-	-	-	-	-	-	-	-	-	-	-	-	laccase_Basidio	-	-	ppo
4415338	0,0%	0,0%	0,0%	0,0%	0,0%	0,0%	0,0%	0,0%	0,0%	Bacteria	Proteobacteria	Betaproteobacteria	Burkholderiales	Comamonadaceae	uncultured	-	-	-	-	-	-	-	-	-	-	-	-	-	-	-	-	-	-	-	-	-	-	-	lip	-
211514	0,0%	0,0%	0,0%	0,0%	0,0%	0,0%	0,0%	0,0%	0,0%	Bacteria	Proteobacteria	Alphaproteobacteria	Rhodospirillales	Rhodospirillaceae	Defluviicoccus	-	-	-	-	-	-	-	-	-	-	-	-	-	-	-	-	-	-	-	-	-	-	-	-	-
1091363	0,0%	0,0%	0,0%	0,0%	0,0%	0,0%	0,0%	0,0%	0,0%	Bacteria	Proteobacteria	Betaproteobacteria	Methylophilales	Methylophilaceae	Methylotenera	-	napA	-	nirA	-	-	nirK	norB	-	-	-	-	ureA	-	-	-	-	-	-	-	-	-	-	-	-
133568	0,0%	0,0%	0,0%	0,0%	0,0%	0,0%	0,0%	0,0%	0,0%	Bacteria	Proteobacteria	Betaproteobacteria	Burkholderiales	Oxalobacteraceae	Undibacterium	-	-	-	-	-	-	-	-	-	-	-	-	-	-	-	-	-	-	-	-	-	-	-	-	-
134359	0,0%	0,0%	0,0%	0,0%	0,0%	0,0%	0,0%	0,0%	0,0%	Bacteria	Proteobacteria	Alphaproteobacteria	Rhizobiales	Rhizobiaceae	Rhizobium	-	napA	narG	nirA	-	-	nirK	norB	nosZ	nifH	nifD	nrfA	ureA	-	-	-	-	chb	mmoX	-	laccase_Asco	laccase_Basidio	-	-	ppo
4330456	0,0%	0,0%	0,0%	0,0%	0,0%	0,0%	0,0%	0,0%	0,0%	Bacteria	Proteobacteria	Gammaproteobacteria	Aeromonadales	Aeromonadaceae	uncultured bacterium	-	-	-	-	-	-	-	-	-	-	-	-	-	-	-	-	-	-	-	-	-	-	-	-	-
4317276	0,0%	0,0%	0,0%	0,0%	0,0%	0,0%	0,0%	0,0%	0,0%	Bacteria	Proteobacteria	Betaproteobacteria	Burkholderiales	Comamonadaceae	Leptothrix	-	napA	-	-	-	nirS	-	norB	nosZ	nifH	nifD	-	ureA	-	-	-	-	-	mmoX	-	-	-	-	-	-
850305	0,0%	0,0%	0,0%	0,0%	0,0%	0,0%	0,0%	0,0%	0,0%	Bacteria	Proteobacteria	Alphaproteobacteria	Rhizobiales		alpha proteobacterium SK200a-2	-	-	-	-	-	-	-	-	-	-	-	-	-	-	-	-	-	-	-	-	-	-	-	-	-
4391560	0,0%	0,0%	0,0%	0,0%	0,0%	0,0%	0,0%	0,0%	0,0%	Bacteria	Proteobacteria	Alphaproteobacteria	Sphingomonadales	Erythrobacteraceae	Porphyrobacter	-	napA	-	-	-	-	-	-	-	-	-	-	-	-	-	-	-	-	-	-	-	-	-	-	ppo
4455962	0,0%	0,0%	0,0%	0,0%	0,0%	0,0%	0,0%	0,0%	0,0%	Bacteria	Proteobacteria	Epsilonproteobacteria	Campylobacterales	Campylobacteraceae	Arcobacter	-	napA	-	-	-	-	-	norB	-	nifH	nifD	nrfA	ureA	-	-	SoxB	-	-	mmoX	-	-	-	-	-	-
331282	0,0%	0,0%	0,0%	0,0%	0,0%	0,0%	0,0%	0,0%	0,0%	Bacteria	Proteobacteria	Alphaproteobacteria	Sphingomonadales	Sphingomonadaceae	Sphingomonas	-	napA	narG	nirA	-	-	nirK	norB	-	nifH	nifD	-	ureA	-	-	-	-	chb	mmoX	-	laccase_Asco	laccase_Basidio	-	-	ppo
113913	0,0%	0,0%	0,0%	0,0%	0,0%	0,0%	0,0%	0,0%	0,0%	Bacteria	Proteobacteria	Alphaproteobacteria	BD7-3		uncultured bacterium	-	-	-	-	-	-	-	-	-	-	-	-	-	-	-	-	-	-	-	-	-	-	-	-	-
148040	0,0%	0,0%	0,0%	0,0%	0,0%	0,0%	0,0%	0,0%	0,0%	Bacteria	Proteobacteria	Alphaproteobacteria	Rhizobiales		uncultured bacterium	-	-	-	-	-	-	-	-	-	-	-	-	-	-	-	-	-	-	-	-	-	-	-	-	-
1896092	0,0%	0,0%	0,0%	0,0%	0,0%	0,1%	0,0%	0,0%	0,0%	Bacteria	Planctomycetes	Planctomycetia	Gemmatales	Gemmataceae	Gemmata	-	-	-	-	-	-	-	-	-	-	-	-	-	-	-	-	-	-	-	-	-	-	-	-	-
151716	0,0%	0,0%	0,0%	0,0%	0,0%	0,0%	0,0%	0,0%	0,0%	Bacteria	Acidobacteria	Acidobacteriia	Acidobacteriales	Acidobacteriaceae	uncultured	cellulolytic	-	-	-	-	-	-	-	-	-	-	-	-	-	-	-	-	-	-	-	-	-	-	lip	-
3327947	0,0%	0,0%	0,0%	0,0%	0,0%	0,0%	0,0%	0,0%	0,0%	Bacteria	Planctomycetes	Planctomycetia	Pirellulales	Pirellulaceae	Pirellula	-	napA	-	-	-	-	-	norB	-	-	-	-	-	-	-	-	-	-	-	-	-	-	-	-	-
553058	0,0%	0,0%	0,0%	0,0%	0,0%	0,0%	0,0%	0,0%	0,0%	Bacteria	Verrucomicrobia	[Spartobacteria]	[Chthoniobacterales]	[Chthoniobacteraceae]	Chthoniobacter	-	napA	-	-	-	-	nirK	-	-	-	-	nrfA	ureA	-	-	-	-	-	-	-	-	-	-	-	-
4308094	0,0%	0,0%	0,0%	0,0%	0,0%	0,0%	0,0%	0,0%	0,0%	Bacteria	Actinobacteria	Actinobacteria	Actinomycetales	Streptomycetaceae	Streptomyces	cellulolytic	napA	narG	nirA	-	-	-	norB	-	nifH	-	-	ureA	-	-	-	-	chb	mmoX	-	laccase_Asco	laccase_Basidio	-	-	ppo
4425629	0,0%	0,0%	0,0%	0,0%	0,0%	0,0%	0,0%	0,0%	0,0%	Bacteria	Proteobacteria	Betaproteobacteria	Burkholderiales	Oxalobacteraceae	Massilia	-	napA	narG	-	-	-	-	norB	nosZ	-	-	-	ureA	-	-	-	-	chb	mmoX	-	laccase_Asco	laccase_Basidio	-	-	ppo
4448558	0,0%	0,0%	0,0%	0,0%	0,0%	0,0%	0,0%	0,0%	0,0%	Bacteria	Proteobacteria	Deltaproteobacteria	Syntrophobacterales	Syntrophobacteraceae	uncultured bacterium	-	-	-	-	-	-	-	-	-	-	-	-	-	-	-	-	-	-	-	-	-	-	-	-	-
823684	0,0%	0,0%	0,0%	0,0%	0,0%	0,0%	0,0%	0,0%	0,0%	Bacteria	Proteobacteria	Alphaproteobacteria	Rhodospirillales	Rhodospirillaceae	uncultured bacterium	-	-	-	-	-	-	-	-	-	-	-	-	-	-	-	-	-	-	-	-	-	-	-	-	-
1141745	0,0%	0,0%	0,0%	0,0%	0,0%	0,0%	0,0%	0,0%	0,0%	Bacteria	Proteobacteria	Betaproteobacteria	Burkholderiales	Comamonadaceae	Variovorax	-	napA	narG	nirA	-	-	-	norB	-	-	-	-	ureA	-	-	-	-	-	mmoX	-	-	-	-	-	-
254949	0,0%	0,0%	0,0%	0,0%	0,0%	0,0%	0,0%	0,0%	0,0%	Bacteria	Proteobacteria	Deltaproteobacteria	Myxococcales		uncultured bacterium	-	-	-	-	-	-	-	-	-	-	-	-	-	-	-	-	-	-	-	-	-	-	-	-	-
1076279	0,0%	0,0%	0,0%	0,0%	0,0%	0,0%	0,0%	0,0%	0,0%	Bacteria	Bacteroidetes	Cytophagia	Cytophagales	Cytophagaceae	Spirosoma	cellulolytic	napA	-	nirA	-	-	-	-	-	-	-	-	-	-	-	-	-	chb	-	-	laccase_Asco	laccase_Basidio	-	-	ppo
4468218	0,0%	0,0%	0,0%	0,0%	0,0%	0,0%	0,0%	0,0%	0,0%	Bacteria	Proteobacteria	Betaproteobacteria	Burkholderiales	Oxalobacteraceae	Chitinimonas	-	-	-	-	-	-	-	-	-	-	-	-	-	-	-	-	-	-	-	-	-	-	-	-	-
640589	0,0%	0,0%	0,0%	0,0%	0,0%	0,0%	0,0%	0,0%	0,0%	Bacteria	Proteobacteria	Betaproteobacteria	Burkholderiales	Comamonadaceae	uncultured	-	-	-	-	-	-	-	-	-	-	-	-	-	-	-	-	-	-	-	-	-	-	-	lip	-
236397	0,0%	0,0%	0,0%	0,0%	0,0%	0,0%	0,0%	0,0%	0,0%	Bacteria	Proteobacteria	Alphaproteobacteria	Rhodospirillales	Rhodospirillaceae	uncultured alpha proteobacterium	-	-	-	-	-	-	-	-	-	-	-	-	-	-	-	-	-	-	-	-	-	-	-	-	-
7954	0,0%	0,0%	0,0%	0,0%	0,0%	0,0%	0,0%	0,0%	0,0%	Bacteria	Proteobacteria	Gammaproteobacteria	Legionellales	Legionellaceae	Legionella	-	-	-	-	-	nirS	-	norB	-	-	-	-	-	-	-	-	-	-	-	-	laccase_Asco	laccase_Basidio	-	-	ppo
4457067	0,0%	0,0%	0,0%	0,0%	0,0%	0,0%	0,0%	0,0%	0,0%	Bacteria	Proteobacteria	Deltaproteobacteria	Desulfuromonadales	Geobacteraceae	uncultured bacterium	-	-	-	-	-	-	-	-	-	-	-	-	-	-	-	-	-	-	-	-	-	-	-	-	-
279138	0,0%	0,0%	0,0%	0,0%	0,0%	0,0%	0,0%	0,0%	0,0%	Bacteria	Proteobacteria	Gammaproteobacteria	Xanthomonadales	Sinobacteraceae	uncultured bacterium	-	-	-	-	-	-	-	-	-	-	-	-	-	-	-	-	-	-	-	-	-	-	-	-	-
138826	0,0%	0,0%	0,0%	0,0%	0,0%	0,0%	0,0%	0,0%	0,0%	Bacteria	Proteobacteria	Gammaproteobacteria	Enterobacteriales	Enterobacteriaceae	Erwinia	cellulolytic	napA	narG	-	-	-	-	-	-	nifH	-	-	-	-	-	-	-	chb	-	-	laccase_Asco	laccase_Basidio	-	-	ppo
4467742	0,0%	0,0%	0,0%	0,0%	0,0%	0,0%	0,0%	0,0%	0,0%	Bacteria	Proteobacteria	Gammaproteobacteria	Legionellales	Coxiellaceae	Aquicella	-	-	-	-	-	-	-	-	-	-	-	-	-	-	-	-	-	-	-	-	-	-	-	-	-
1119166	0,0%	0,0%	0,0%	0,0%	0,0%	0,0%	0,0%	0,0%	0,0%	Bacteria	Proteobacteria	Betaproteobacteria	IS-44		uncultured	-	-	-	-	-	-	-	-	-	-	-	-	-	-	-	-	-	-	-	-	-	-	-	lip	-
273056	0,0%	0,0%	0,0%	0,0%	0,0%	0,0%	0,0%	0,0%	0,0%	Bacteria	Proteobacteria	Betaproteobacteria	Rhodocyclales	Rhodocyclaceae	Denitratisoma	-	-	-	-	-	-	-	-	-	-	-	-	-	-	-	-	-	-	-	-	-	-	-	-	-
4376608	0,0%	0,0%	0,0%	0,0%	0,0%	0,0%	0,0%	0,0%	0,0%	Bacteria	Actinobacteria	Actinobacteria	Actinomycetales	Micromonosporaceae	Actinoplanes	cellulolytic	napA	narG	-	-	-	nirK	-	-	-	-	-	ureA	-	-	-	-	-	-	-	laccase_Asco	laccase_Basidio	-	-	ppo
2655362	0,0%	0,0%	0,0%	0,0%	0,0%	0,0%	0,0%	0,0%	0,0%	Bacteria	Proteobacteria	Alphaproteobacteria	Rhizobiales		Ancalomicrobium	-	-	-	-	-	-	-	-	-	-	-	-	-	-	-	-	-	-	-	-	-	-	-	-	-
4308657	0,0%	0,0%	0,0%	0,0%	0,0%	0,0%	0,0%	0,0%	0,0%	Bacteria	Proteobacteria	Betaproteobacteria	SC-I-84		uncultured bacterium	-	-	-	-	-	-	-	-	-	-	-	-	-	-	-	-	-	-	-	-	-	-	-	-	-
2770485	0,0%	0,0%	0,0%	0,0%	0,0%	0,0%	0,0%	0,0%	0,0%	Bacteria	Actinobacteria	Actinobacteria	Actinomycetales	ACK-M1	hgcI clade	-	-	-	-	-	-	-	-	-	-	-	-	-	-	-	-	-	-	-	-	-	-	-	-	-
217822	0,0%	0,0%	0,0%	0,0%	0,0%	0,0%	0,0%	0,0%	0,0%	Bacteria	Verrucomicrobia	[Pedosphaerae]	[Pedosphaerales]	Ellin515	uncultured Verrucomicrobia subdivision 3 bacterium	-	-	-	-	-	-	-	-	-	-	-	-	-	-	-	-	-	-	-	-	-	-	-	-	-
808071	0,0%	0,0%	0,0%	0,0%	0,0%	0,0%	0,0%	0,0%	0,0%	Bacteria	Bacteroidetes	[Saprospirae]	[Saprospirales]	Chitinophagaceae	Sediminibacterium	-	-	-	-	-	-	-	-	-	-	-	-	-	-	-	-	-	-	-	-	-	-	-	-	-
136883	0,0%	0,0%	0,0%	0,0%	0,0%	0,0%	0,0%	0,0%	0,0%	Bacteria	Proteobacteria	Deltaproteobacteria			uncultured soil bacterium	-	-	-	-	-	-	-	-	-	-	-	-	-	-	-	-	-	-	-	-	-	-	-	-	-
4453524	0,0%	0,0%	0,0%	0,0%	0,0%	0,0%	0,0%	0,0%	0,0%	Bacteria	Proteobacteria	Betaproteobacteria	Burkholderiales	Comamonadaceae	Massilia	-	napA	narG	-	-	-	-	norB	nosZ	-	-	-	ureA	-	-	-	-	chb	mmoX	-	laccase_Asco	laccase_Basidio	-	-	ppo
814609	0,0%	0,0%	0,0%	0,0%	0,0%	0,0%	0,0%	0,0%	0,0%	Bacteria	Actinobacteria	Actinobacteria	Actinomycetales		Acidothermus	-	napA	narG	-	-	-	nirK	-	-	-	-	-	-	-	-	-	-	-	-	-	-	-	-	-	-
3798902	0,0%	0,0%	0,0%	0,0%	0,0%	0,0%	0,0%	0,0%	0,0%	Bacteria	Proteobacteria	Alphaproteobacteria	Rhizobiales	Methylocystaceae	uncultured forest soil bacterium	-	-	-	-	-	-	-	-	-	-	-	-	-	-	-	-	-	-	-	-	-	-	-	-	-
4469855	0,0%	0,0%	0,0%	0,0%	0,0%	0,0%	0,0%	0,0%	0,0%	Bacteria	Proteobacteria	Gammaproteobacteria	Legionellales	Coxiellaceae	Aquicella	-	-	-	-	-	-	-	-	-	-	-	-	-	-	-	-	-	-	-	-	-	-	-	-	-
147658	0,0%	0,0%	0,0%	0,0%	0,0%	0,0%	0,0%	0,0%	0,0%	Archaea	Euryarchaeota	Thermoplasmata	E2	Marine group II	uncultured euryarchaeote	-	-	-	-	-	-	-	-	-	-	-	-	-	-	-	-	-	-	-	-	-	-	-	-	-
360500	0,0%	0,0%	0,0%	0,0%	0,0%	0,0%	0,0%	0,0%	0,0%	Bacteria	Proteobacteria	Alphaproteobacteria	Rhodospirillales	Acetobacteraceae	Acidiphilium	-	napA	narG	-	-	-	-	-	-	nifH	-	-	-	-	-	SoxB	-	-	mmoX	-	laccase_Asco	laccase_Basidio	-	-	ppo
713397	0,0%	0,0%	0,0%	0,0%	0,0%	0,0%	0,0%	0,0%	0,0%	Bacteria	Proteobacteria	Alphaproteobacteria	Rhizobiales	Hyphomicrobiaceae	Hyphomicrobium	-	napA	narG	nirA	-	-	nirK	norB	nosZ	nifH	nifD	-	ureA	-	-	-	-	-	mmoX	-	laccase_Asco	laccase_Basidio	-	-	ppo
1011210	0,0%	0,0%	0,0%	0,0%	0,0%	0,0%	0,0%	0,0%	0,0%	Bacteria	Proteobacteria	Betaproteobacteria	Burkholderiales	Comamonadaceae	uncultured	-	-	-	-	-	-	-	-	-	-	-	-	-	-	-	-	-	-	-	-	-	-	-	lip	-
137429	0,0%	0,0%	0,0%	0,0%	0,0%	0,0%	0,0%	0,0%	0,0%	Bacteria	Planctomycetes	Planctomycetia	Pirellulales	Pirellulaceae	uncultured	-	-	-	-	-	-	-	-	-	-	-	-	-	-	-	-	-	-	-	-	-	-	-	lip	-
971457	0,0%	0,0%	0,0%	0,0%	0,0%	0,0%	0,0%	0,0%	0,0%	Bacteria	Bacteroidetes	Flavobacteriia	Flavobacteriales	Flavobacteriaceae	Flavobacterium	cellulolytic	napA	-	nirA	-	-	nirK	norB	nosZ	-	-	-	ureA	-	-	-	-	-	-	-	laccase_Asco	laccase_Basidio	-	-	ppo
4344786	0,0%	0,0%	0,0%	0,0%	0,0%	0,0%	0,0%	0,0%	0,0%	Bacteria	Actinobacteria	Thermoleophilia	Gaiellales	Gaiellaceae	uncultured bacterium	-	-	-	-	-	-	-	-	-	-	-	-	-	-	-	-	-	-	-	-	-	-	-	-	-
805022	0,0%	0,0%	0,0%	0,0%	0,0%	0,0%	0,0%	0,0%	0,0%	Bacteria	Proteobacteria	Gammaproteobacteria	Aeromonadales	Aeromonadaceae	uncultured bacterium	-	-	-	-	-	-	-	-	-	-	-	-	-	-	-	-	-	-	-	-	-	-	-	-	-
1119093	0,0%	0,0%	0,0%	0,0%	0,0%	0,0%	0,0%	0,0%	0,0%	Bacteria	Chloroflexi	Thermomicrobia	JG30-KF-CM45		uncultured soil bacterium	-	-	-	-	-	-	-	-	-	-	-	-	-	-	-	-	-	-	-	-	-	-	-	-	-
867450	0,0%	0,0%	0,0%	0,0%	0,0%	0,0%	0,0%	0,0%	0,0%	Bacteria	Bacteroidetes	Flavobacteriia	Flavobacteriales	Flavobacteriaceae	Flavobacterium	cellulolytic	napA	-	nirA	-	-	nirK	norB	nosZ	-	-	-	ureA	-	-	-	-	-	-	-	laccase_Asco	laccase_Basidio	-	-	ppo
4453636	0,0%	0,0%	0,0%	0,0%	0,0%	0,0%	0,0%	0,0%	0,0%	Bacteria	Proteobacteria	Deltaproteobacteria	Desulfuromonadales	Geobacteraceae	Pelobacter	-	napA	-	-	-	-	-	-	-	nifH	nifD	nrfA	-	-	-	-	-	-	-	-	-	-	-	-	-
46818	0,0%	0,0%	0,0%	0,0%	0,0%	0,1%	0,0%	0,0%	0,0%	Bacteria	Proteobacteria	Alphaproteobacteria	Caulobacterales	Caulobacteraceae	Phenylobacterium	-	napA	narG	-	-	-	nirK	norB	-	-	-	-	-	-	-	-	-	-	-	-	laccase_Asco	laccase_Basidio	-	-	ppo
4413439	0,0%	0,0%	0,0%	0,0%	0,0%	0,0%	0,0%	0,0%	0,0%	Bacteria	Proteobacteria	Betaproteobacteria	Burkholderiales	Comamonadaceae	uncultured	-	-	-	-	-	-	-	-	-	-	-	-	-	-	-	-	-	-	-	-	-	-	-	lip	-
954737	0,0%	0,0%	0,0%	0,0%	0,0%	0,0%	0,0%	0,0%	0,1%	Bacteria	Proteobacteria	Gammaproteobacteria	Methylococcales	Methylococcaceae	Methylomonas	-	napA	-	nirA	-	nirS	nirK	norB	-	nifH	nifD	-	ureA	-	-	-	amoA_AOB	-	mmoX	pmoA	laccase_Asco	laccase_Basidio	-	-	ppo
4306457	0,0%	0,0%	0,0%	0,0%	0,0%	0,0%	0,0%	0,0%	0,0%	Bacteria	Proteobacteria	Alphaproteobacteria	Rhizobiales		Pleomorphomonas	-	-	-	-	-	-	-	-	-	nifH	-	-	-	-	-	-	-	-	-	-	-	-	-	-	-
1046397	0,0%	0,0%	0,0%	0,0%	0,0%	0,0%	0,0%	0,0%	0,0%	Bacteria	Bacteroidetes	Flavobacteriia	Flavobacteriales	Cryomorphaceae	Fluviicola	-	-	-	nirA	-	-	-	norB	-	-	-	-	-	-	-	-	-	-	-	-	laccase_Asco	laccase_Basidio	-	-	ppo
114130	0,0%	0,0%	0,0%	0,0%	0,0%	0,0%	0,0%	0,0%	0,1%	Bacteria	Proteobacteria	Alphaproteobacteria	Rhodospirillales	Rhodospirillaceae	uncultured forest soil bacterium	-	-	-	-	-	-	-	-	-	-	-	-	-	-	-	-	-	-	-	-	-	-	-	-	-
868948	0,0%	0,0%	0,0%	0,0%	0,0%	0,0%	0,0%	0,0%	0,0%	Bacteria	Proteobacteria	Alphaproteobacteria	Rhodobacterales	Rhodobacteraceae	uncultured	-	-	-	-	-	-	-	-	-	-	-	-	-	-	-	-	-	-	-	-	-	-	-	lip	-
2947338	0,0%	0,0%	0,0%	0,0%	0,0%	0,0%	0,0%	0,0%	0,0%	Bacteria	Proteobacteria	Alphaproteobacteria	Sphingomonadales	Sphingomonadaceae	Sphingomonas	-	napA	narG	nirA	-	-	nirK	norB	-	nifH	nifD	-	ureA	-	-	-	-	chb	mmoX	-	laccase_Asco	laccase_Basidio	-	-	ppo
783224	0,0%	0,0%	0,0%	0,0%	0,0%	0,0%	0,0%	0,0%	0,0%	Bacteria	Proteobacteria	Alphaproteobacteria	Rhodobacterales	Rhodobacteraceae	Rhodobacter	-	napA	-	nirA	-	nirS	nirK	norB	nosZ	nifH	nifD	-	ureA	-	-	SoxB	-	-	mmoX	-	laccase_Asco	laccase_Basidio	-	-	ppo
73506	0,0%	0,0%	0,0%	0,0%	0,0%	0,0%	0,0%	0,0%	0,0%	Bacteria	Proteobacteria	Gammaproteobacteria	Legionellales	Coxiellaceae	Diplorickettsia	-	-	-	-	-	-	-	-	-	-	-	-	-	-	-	-	-	-	-	-	-	-	-	-	-
4393266	0,0%	0,0%	0,0%	0,0%	0,0%	0,0%	0,0%	0,0%	0,0%	Bacteria	Actinobacteria	Acidimicrobiia	Acidimicrobiales	JdFBGBact	uncultured bacterium	-	-	-	-	-	-	-	-	-	-	-	-	-	-	-	-	-	-	-	-	-	-	-	-	-
995583	0,0%	0,0%	0,0%	0,0%	0,0%	0,0%	0,0%	0,0%	0,0%	Bacteria	Bacteroidetes	Sphingobacteriia	Sphingobacteriales		uncultured bacterium	-	-	-	-	-	-	-	-	-	-	-	-	-	-	-	-	-	-	-	-	-	-	-	-	-
4409771	0,0%	0,0%	0,0%	0,0%	0,0%	0,0%	0,0%	0,0%	0,0%	Bacteria	Acidobacteria	Acidobacteriia	Acidobacteriales	Koribacteraceae	uncultured	-	-	-	-	-	-	-	-	-	-	-	-	-	-	-	-	-	-	-	-	-	-	-	lip	-
762780	0,0%	0,0%	0,0%	0,0%	0,0%	0,0%	0,0%	0,0%	0,0%	Bacteria	Proteobacteria	Betaproteobacteria	Rhodocyclales	Rhodocyclaceae	Denitratisoma	-	-	-	-	-	-	-	-	-	-	-	-	-	-	-	-	-	-	-	-	-	-	-	-	-
1119239	0,0%	0,0%	0,0%	0,0%	0,0%	0,0%	0,0%	0,0%	0,0%	Bacteria	Proteobacteria	Gammaproteobacteria	Legionellales	Coxiellaceae	Aquicella	-	-	-	-	-	-	-	-	-	-	-	-	-	-	-	-	-	-	-	-	-	-	-	-	-
230607	0,0%	0,0%	0,0%	0,0%	0,0%	0,0%	0,0%	0,0%	0,0%	Bacteria	Proteobacteria	Betaproteobacteria	Burkholderiales	Comamonadaceae	uncultured	-	-	-	-	-	-	-	-	-	-	-	-	-	-	-	-	-	-	-	-	-	-	-	lip	-
1068847	0,0%	0,0%	0,0%	0,0%	0,0%	0,0%	0,0%	0,0%	0,0%	Bacteria	Bacteroidetes	[Saprospirae]	[Saprospirales]	Chitinophagaceae	uncultured	-	-	-	-	-	-	-	-	-	-	-	-	-	-	-	-	-	-	-	-	-	-	-	lip	-
156946	0,0%	0,0%	0,0%	0,0%	0,0%	0,0%	0,0%	0,0%	0,0%	Bacteria	Proteobacteria	Gammaproteobacteria	Legionellales		uncultured bacterium	-	-	-	-	-	-	-	-	-	-	-	-	-	-	-	-	-	-	-	-	-	-	-	-	-
4468432	0,0%	0,0%	0,0%	0,0%	0,0%	0,0%	0,0%	0,0%	0,0%	Bacteria	Proteobacteria	Betaproteobacteria	Ellin6067		uncultured	-	-	-	-	-	-	-	-	-	-	-	-	-	-	-	-	-	-	-	-	-	-	-	lip	-
212454	0,0%	0,0%	0,0%	0,0%	0,0%	0,0%	0,0%	0,0%	0,0%	Bacteria	Proteobacteria	Gammaproteobacteria	Methylococcales	Crenotrichaceae	Crenothrix	-	-	-	-	-	-	-	-	-	-	-	-	-	-	-	-	-	-	-	pmoA	-	-	-	-	-
151454	0,0%	0,0%	0,0%	0,0%	0,0%	0,0%	0,0%	0,0%	0,0%	Bacteria	Acidobacteria	Acidobacteria-6	iii1-15	RB40	uncultured bacterium	-	-	-	-	-	-	-	-	-	-	-	-	-	-	-	-	-	-	-	-	-	-	-	-	-
4321427	0,0%	0,0%	0,0%	0,0%	0,0%	0,0%	0,0%	0,0%	0,1%	Bacteria	Proteobacteria	Alphaproteobacteria	Sphingomonadales	Sphingomonadaceae	Sphingomonas	-	napA	narG	nirA	-	-	nirK	norB	-	nifH	nifD	-	ureA	-	-	-	-	chb	mmoX	-	laccase_Asco	laccase_Basidio	-	-	ppo
821690	0,0%	0,0%	0,0%	0,0%	0,0%	0,0%	0,0%	0,0%	0,0%	Bacteria	Proteobacteria	Betaproteobacteria	Burkholderiales	Burkholderiaceae	Burkholderia	-	napA	narG	nirA	-	nirS	nirK	norB	nosZ	nifH	nifD	-	ureA	-	-	-	-	chb	mmoX	-	laccase_Asco	laccase_Basidio	-	-	ppo
4341461	0,0%	0,0%	0,0%	0,0%	0,0%	0,0%	0,0%	0,0%	0,0%	Bacteria	Proteobacteria	Alphaproteobacteria	Caulobacterales	Caulobacteraceae	uncultured	-	-	-	-	-	-	-	-	-	-	-	-	-	-	-	-	-	-	-	-	-	-	-	lip	-
4341460	0,0%	0,0%	0,0%	0,0%	0,0%	0,0%	0,0%	0,0%	0,0%	Bacteria	Proteobacteria	Alphaproteobacteria	Rhizobiales	Beijerinckiaceae	uncultured	-	-	-	-	-	-	-	-	-	-	-	-	-	-	-	-	-	-	-	-	-	-	-	lip	-
4450360	0,0%	0,0%	0,0%	0,0%	0,0%	0,0%	0,0%	0,0%	0,0%	Bacteria	Proteobacteria	Alphaproteobacteria	Sphingomonadales	Sphingomonadaceae	Sphingomonas	-	napA	narG	nirA	-	-	nirK	norB	-	nifH	nifD	-	ureA	-	-	-	-	chb	mmoX	-	laccase_Asco	laccase_Basidio	-	-	ppo
142996	0,0%	0,0%	0,0%	0,0%	0,0%	0,0%	0,0%	0,0%	0,0%	Bacteria	Cyanobacteria	Synechococcophycideae	Pseudanabaenales	Pseudanabaenaceae	Synechococcus	-	napA	-	nirA	-	-	-	-	-	nifH	nifD	-	ureA	-	-	-	-	-	-	-	laccase_Asco	laccase_Basidio	-	-	ppo
240664	0,0%	0,0%	0,0%	0,0%	0,0%	0,0%	0,0%	0,0%	0,0%	Bacteria	Proteobacteria	Alphaproteobacteria	Rhodobacterales	Rhodobacteraceae	Gemmobacter	-	-	-	-	-	-	-	-	-	-	-	-	-	-	-	-	-	-	-	-	-	-	-	-	-
818668	0,0%	0,0%	0,0%	0,0%	0,0%	0,0%	0,0%	0,0%	0,0%	Bacteria	Fusobacteria	Fusobacteriia	Fusobacteriales	Fusobacteriaceae	Psychrilyobacter	-	-	-	-	-	-	-	-	-	-	-	-	-	-	-	-	-	-	-	-	-	-	-	-	-
1124779	0,0%	0,0%	0,0%	0,0%	0,0%	0,0%	0,0%	0,0%	0,0%	Bacteria	Proteobacteria	Betaproteobacteria	Burkholderiales	Comamonadaceae	Thiomonas	-	napA	narG	-	-	-	nirK	norB	-	-	-	-	ureA	-	-	SoxB	-	-	-	-	laccase_Asco	laccase_Basidio	-	-	ppo
610756	0,0%	0,0%	0,0%	0,0%	0,0%	0,0%	0,0%	0,0%	0,0%	Bacteria	Cyanobacteria	Gloeobacterophycideae	Gloeobacterales	Gloeobacteraceae	uncultured bacterium	-	-	-	-	-	-	-	-	-	-	-	-	-	-	-	-	-	-	-	-	-	-	-	-	-
586639	0,0%	0,0%	0,0%	0,0%	0,0%	0,0%	0,0%	0,0%	0,1%	Bacteria	Proteobacteria	Gammaproteobacteria	Chromatiales	Chromatiaceae	Arenicella	-	-	-	-	-	-	-	-	-	-	-	-	-	-	-	-	-	-	-	-	-	-	-	-	-
751973	0,0%	0,0%	0,0%	0,0%	0,0%	0,0%	0,0%	0,0%	0,0%	Bacteria	Proteobacteria	Gammaproteobacteria	Pseudomonadales	Pseudomonadaceae	Pseudomonas	cellulolytic	napA	narG	nirA	-	nirS	nirK	norB	nosZ	nifH	nifD	-	ureA	-	dsrB	SoxB	-	-	mmoX	-	laccase_Asco	laccase_Basidio	-	-	ppo
1125608	0,0%	0,0%	0,0%	0,0%	0,0%	0,0%	0,0%	0,0%	0,0%	Bacteria	Proteobacteria	Gammaproteobacteria	Xanthomonadales	Xanthomonadaceae	Dokdonella	cellulolytic	-	-	-	-	-	-	-	-	-	-	-	-	-	-	-	-	-	-	-	-	-	-	-	-
563462	0,0%	0,0%	0,0%	0,0%	0,0%	0,0%	0,0%	0,0%	0,1%	Bacteria	Proteobacteria	Gammaproteobacteria	Xanthomonadales	Sinobacteraceae	uncultured gamma proteobacterium	-	-	-	-	-	-	-	-	-	-	-	-	-	-	-	-	-	-	-	-	-	-	-	-	-
1108954	0,0%	0,0%	0,0%	0,0%	0,0%	0,0%	0,0%	0,0%	0,0%	Bacteria	Actinobacteria	Actinobacteria	Actinomycetales		Acidothermus	-	napA	narG	-	-	-	nirK	-	-	-	-	-	-	-	-	-	-	-	-	-	-	-	-	-	-
52859	0,0%	0,0%	0,0%	0,0%	0,0%	0,0%	0,0%	0,0%	0,0%	Bacteria	Proteobacteria	Alphaproteobacteria	Rhizobiales	Hyphomicrobiaceae	Hyphomicrobium	-	napA	narG	nirA	-	-	nirK	norB	nosZ	nifH	nifD	-	ureA	-	-	-	-	-	mmoX	-	laccase_Asco	laccase_Basidio	-	-	ppo
4445338	0,0%	0,0%	0,0%	0,0%	0,0%	0,0%	0,0%	0,0%	0,0%	Bacteria	Proteobacteria	Deltaproteobacteria	Myxococcales	Nannocystaceae	Nannocystis	-	-	-	-	-	-	-	-	-	-	-	-	-	-	-	-	-	-	-	-	-	-	-	-	-
4474906	0,0%	0,0%	0,0%	0,0%	0,0%	0,0%	0,0%	0,0%	0,0%	Bacteria	Planctomycetes	Planctomycetia	Gemmatales	Isosphaeraceae	Singulisphaera	-	napA	-	-	-	-	-	-	-	-	-	-	ureA	-	-	-	-	-	-	-	-	-	-	-	-
639323	0,0%	0,0%	0,0%	0,0%	0,0%	0,1%	0,0%	0,0%	0,0%	Bacteria	Verrucomicrobia	Opitutae	Opitutales	Opitutaceae	Opitutus	-	napA	-	nirA	-	-	-	-	nosZ	-	-	nrfA	ureA	-	-	-	-	-	-	-	-	laccase_Basidio	-	-	-
3667538	0,0%	0,0%	0,0%	0,0%	0,0%	0,0%	0,0%	0,0%	0,0%	Bacteria	Proteobacteria	Alphaproteobacteria	Rhizobiales	Hyphomicrobiaceae	uncultured	-	-	-	-	-	-	-	-	-	-	-	-	-	-	-	-	-	-	-	-	-	-	-	lip	-
758346	0,0%	0,0%	0,0%	0,0%	0,0%	0,0%	0,0%	0,0%	0,0%	Bacteria	Bacteroidetes	Sphingobacteriia	Sphingobacteriales		uncultured bacterium	-	-	-	-	-	-	-	-	-	-	-	-	-	-	-	-	-	-	-	-	-	-	-	-	-
952163	0,0%	0,0%	0,0%	0,0%	0,0%	0,0%	0,0%	0,0%	0,0%	Bacteria	Proteobacteria	Betaproteobacteria	Neisseriales	Neisseriaceae	Jeongeupia	-	-	-	-	-	-	-	-	-	-	-	-	-	-	-	-	-	-	-	-	-	-	-	-	-
786086	0,0%	0,0%	0,0%	0,0%	0,0%	0,0%	0,0%	0,0%	0,0%	Bacteria	Bacteroidetes	Sphingobacteriia	Sphingobacteriales		uncultured bacterium	-	-	-	-	-	-	-	-	-	-	-	-	-	-	-	-	-	-	-	-	-	-	-	-	-
1110791	0,0%	0,0%	0,0%	0,0%	0,0%	0,0%	0,0%	0,0%	0,0%	Bacteria	Bacteroidetes	[Saprospirae]	[Saprospirales]	Chitinophagaceae	uncultured	-	-	-	-	-	-	-	-	-	-	-	-	-	-	-	-	-	-	-	-	-	-	-	lip	-
4479579	0,0%	0,0%	0,0%	0,0%	0,0%	0,0%	0,0%	0,0%	0,0%	Bacteria	Proteobacteria	Deltaproteobacteria	MIZ46		uncultured Syntrophobacteraceae bacterium	-	-	-	-	-	-	-	-	-	-	-	-	-	-	-	-	-	-	-	-	-	-	-	-	-
2279387	0,0%	0,0%	0,0%	0,0%	0,0%	0,0%	0,0%	0,0%	0,0%	Bacteria	Proteobacteria	Betaproteobacteria	Burkholderiales	Oxalobacteraceae	Noviherbaspirillum	-	napA	-	-	-	-	-	norB	-	-	-	-	ureA	-	-	-	-	-	-	-	-	-	-	-	-
836732	0,0%	0,0%	0,0%	0,0%	0,0%	0,0%	0,0%	0,0%	0,0%	Bacteria	Proteobacteria	Alphaproteobacteria	Rhizobiales		uncultured bacterium	-	-	-	-	-	-	-	-	-	-	-	-	-	-	-	-	-	-	-	-	-	-	-	-	-
510406	0,0%	0,0%	0,0%	0,0%	0,0%	0,0%	0,0%	0,0%	0,0%	Bacteria	Verrucomicrobia	[Spartobacteria]	[Chthoniobacterales]	[Chthoniobacteraceae]	uncultured bacterium	-	-	-	-	-	-	-	-	-	-	-	-	-	-	-	-	-	-	-	-	-	-	-	-	-
589010	0,0%	0,0%	0,0%	0,0%	0,0%	0,0%	0,0%	0,0%	0,0%	Bacteria	Proteobacteria	Betaproteobacteria	Neisseriales	Neisseriaceae	Vogesella	-	napA	narG	-	-	nirS	-	-	-	-	-	-	-	-	-	-	-	-	-	-	laccase_Asco	laccase_Basidio	-	-	ppo
4369159	0,0%	0,0%	0,0%	0,0%	0,0%	0,0%	0,0%	0,0%	0,0%	Bacteria	Acidobacteria	Acidobacteriia	Acidobacteriales	Acidobacteriaceae	Acidicapsa	cellulolytic	-	-	-	-	-	-	-	-	-	-	-	-	-	-	-	-	-	-	-	-	-	-	-	-
167760	0,0%	0,0%	0,0%	0,0%	0,0%	0,0%	0,0%	0,0%	0,0%	Bacteria	Proteobacteria	Gammaproteobacteria	Aeromonadales	Aeromonadaceae	uncultured bacterium	-	-	-	-	-	-	-	-	-	-	-	-	-	-	-	-	-	-	-	-	-	-	-	-	-
1107517	0,0%	0,0%	0,0%	0,0%	0,0%	0,0%	0,0%	0,0%	0,0%	Bacteria	Actinobacteria	Acidimicrobiia	Acidimicrobiales		uncultured bacterium	-	-	-	-	-	-	-	-	-	-	-	-	-	-	-	-	-	-	-	-	-	-	-	-	-
4301044	0,0%	0,0%	0,0%	0,0%	0,0%	0,0%	0,0%	0,0%	0,0%	Bacteria	Gemmatimonadetes	Gemmatimonadetes			uncultured	-	-	-	-	-	-	-	-	-	-	-	-	-	-	-	-	-	-	-	-	-	-	-	lip	-
4378592	0,0%	0,0%	0,0%	0,0%	0,0%	0,0%	0,0%	0,0%	0,0%	Bacteria	Proteobacteria	Alphaproteobacteria	Rhodobacterales	Rhodobacteraceae	Defluviimonas	-	napA	narG	-	-	-	-	-	-	-	-	-	ureA	-	-	-	-	-	-	-	-	laccase_Basidio	-	-	ppo
558742	0,0%	0,0%	0,0%	0,0%	0,0%	0,0%	0,0%	0,0%	0,0%	Bacteria	Proteobacteria	Alphaproteobacteria	Rickettsiales		Candidatus Captivus	-	-	-	-	-	-	-	-	-	-	-	-	-	-	-	-	-	-	-	-	-	-	-	-	-
4386920	0,0%	0,0%	0,0%	0,0%	0,0%	0,0%	0,0%	0,0%	0,0%	Bacteria	Proteobacteria	Gammaproteobacteria	Pseudomonadales	Pseudomonadaceae	Pseudomonas	cellulolytic	napA	narG	nirA	-	nirS	nirK	norB	nosZ	nifH	nifD	-	ureA	-	dsrB	SoxB	-	-	mmoX	-	laccase_Asco	laccase_Basidio	-	-	ppo
509124	0,0%	0,0%	0,0%	0,0%	0,0%	0,0%	0,0%	0,0%	0,0%	Bacteria	Verrucomicrobia	[Spartobacteria]	[Chthoniobacterales]	[Chthoniobacteraceae]	uncultured eubacterium WCHD3-88	-	-	-	-	-	-	-	-	-	-	-	-	-	-	-	-	-	-	-	-	-	-	-	-	-
917888	0,0%	0,0%	0,0%	0,0%	0,0%	0,0%	0,0%	0,0%	0,0%	Bacteria	Bacteroidetes	Sphingobacteriia	Sphingobacteriales	Sphingobacteriaceae	Mucilaginibacter	-	-	-	-	-	-	-	-	-	-	-	-	-	-	-	-	-	-	-	-	-	-	-	-	-
205044	0,0%	0,0%	0,0%	0,0%	0,0%	0,0%	0,0%	0,0%	0,0%	Bacteria	Proteobacteria	Betaproteobacteria	Ellin6067		uncultured	-	-	-	-	-	-	-	-	-	-	-	-	-	-	-	-	-	-	-	-	-	-	-	lip	-
590507	0,0%	0,0%	0,0%	0,0%	0,0%	0,0%	0,0%	0,0%	0,0%	Bacteria	Proteobacteria	Alphaproteobacteria			alpha proteobacterium A0839	-	-	-	-	-	-	-	-	-	-	-	-	-	-	-	-	-	-	-	-	-	-	-	-	-
2866157	0,0%	0,0%	0,0%	0,0%	0,0%	0,0%	0,0%	0,0%	0,0%	Bacteria	Spirochaetes	Spirochaetes	Spirochaetales	Spirochaetaceae	uncultured	-	-	-	-	-	-	-	-	-	-	-	-	-	-	-	-	-	-	-	-	-	-	-	lip	-
98258	0,0%	0,0%	0,0%	0,0%	0,0%	0,0%	0,0%	0,0%	0,0%	Bacteria	Cyanobacteria	4C0d-2	MLE1-12		uncultured bacterium	-	-	-	-	-	-	-	-	-	-	-	-	-	-	-	-	-	-	-	-	-	-	-	-	-
154249	0,0%	0,0%	0,0%	0,0%	0,0%	0,0%	0,0%	0,0%	0,0%	Bacteria	Proteobacteria	Alphaproteobacteria	Rhodospirillales	Rhodospirillaceae	Dongia	-	-	-	-	-	-	-	-	-	-	-	-	-	-	-	-	-	-	-	-	-	-	-	-	-
4305160	0,0%	0,0%	0,0%	0,0%	0,0%	0,0%	0,0%	0,0%	0,0%	Bacteria	Proteobacteria	Betaproteobacteria	Burkholderiales	Comamonadaceae	uncultured	-	-	-	-	-	-	-	-	-	-	-	-	-	-	-	-	-	-	-	-	-	-	-	lip	-
21795	0,0%	0,0%	0,0%	0,0%	0,0%	0,0%	0,0%	0,0%	0,0%	Bacteria	Proteobacteria	Alphaproteobacteria	Rhodospirillales	Acetobacteraceae	Acidisphaera	-	napA	-	-	-	-	-	-	-	-	-	-	ureA	-	-	-	-	-	mmoX	-	laccase_Asco	laccase_Basidio	-	-	ppo
741010	0,0%	0,0%	0,0%	0,0%	0,0%	0,0%	0,0%	0,0%	0,0%	Bacteria	Proteobacteria	Gammaproteobacteria	Alteromonadales	Alteromonadaceae	Cellvibrio	-	napA	narG	-	-	-	nirK	norB	-	-	-	-	-	-	-	-	-	chb	-	-	-	laccase_Basidio	-	-	-
4379512	0,0%	0,0%	0,0%	0,0%	0,0%	0,0%	0,0%	0,0%	0,0%	Bacteria	Verrucomicrobia	Opitutae	Opitutales	Opitutaceae	Opitutus	-	napA	-	nirA	-	-	-	-	nosZ	-	-	nrfA	ureA	-	-	-	-	-	-	-	-	laccase_Basidio	-	-	-
141085	0,0%	0,0%	0,0%	0,0%	0,0%	0,0%	0,0%	0,0%	0,0%	Bacteria	Proteobacteria	Alphaproteobacteria	Rhodospirillales	Acetobacteraceae	Acidocella	-	napA	narG	nirA	-	-	-	-	-	-	-	-	ureA	-	-	-	-	-	-	-	laccase_Asco	laccase_Basidio	-	-	ppo
105699	0,0%	0,0%	0,0%	0,0%	0,0%	0,0%	0,0%	0,0%	0,0%	Bacteria	Proteobacteria	Gammaproteobacteria	Xanthomonadales	Xanthomonadaceae	Rhodanobacter	cellulolytic	napA	narG	-	-	nirS	nirK	norB	nosZ	-	-	-	-	-	-	-	-	-	mmoX	-	-	laccase_Basidio	-	-	ppo
4476570	0,0%	0,0%	0,0%	0,0%	0,0%	0,0%	0,0%	0,0%	0,0%	Bacteria	Firmicutes	Clostridia	Clostridiales	Peptococcaceae	uncultured	-	-	-	-	-	-	-	-	-	-	-	-	-	-	-	-	-	-	-	-	-	-	-	lip	-
816602	0,0%	0,0%	0,0%	0,0%	0,0%	0,0%	0,0%	0,0%	0,0%	Bacteria	Proteobacteria	Betaproteobacteria	SBla14		Sideroxydans	-	napA	-	-	-	nirS	-	norB	-	nifH	nifD	-	-	dsrA	dsrB	-	-	-	-	-	-	-	-	-	-
4463045	0,0%	0,0%	0,0%	0,0%	0,0%	0,0%	0,0%	0,0%	0,0%	Bacteria	Proteobacteria	Alphaproteobacteria	Sphingomonadales	Sphingomonadaceae	Sphingomonas	-	napA	narG	nirA	-	-	nirK	norB	-	nifH	nifD	-	ureA	-	-	-	-	chb	mmoX	-	laccase_Asco	laccase_Basidio	-	-	ppo
673645	0,0%	0,0%	0,0%	0,0%	0,0%	0,0%	0,0%	0,0%	0,0%	Bacteria	Cyanobacteria	Nostocophycideae	Nostocales	Nostocaceae	Tolypothrix sp. CAWBG76	-	-	-	-	-	-	-	-	-	-	-	-	-	-	-	-	-	-	-	-	-	-	-	-	-
821325	0,0%	0,0%	0,0%	0,0%	0,0%	0,0%	0,0%	0,0%	0,0%	Bacteria	Firmicutes	Bacilli	Bacillales	Planococcaceae	uncultured	-	-	-	-	-	-	-	-	-	-	-	-	-	-	-	-	-	-	-	-	-	-	-	lip	-
81873	0,0%	0,0%	0,0%	0,0%	0,0%	0,0%	0,0%	0,0%	0,1%	Bacteria	Verrucomicrobia	[Pedosphaerae]	[Pedosphaerales]	auto67_4W	uncultured Verrucomicrobia bacterium	-	-	-	-	-	-	-	-	-	-	-	-	-	-	-	-	-	-	-	-	-	-	-	-	-
250637	0,0%	0,0%	0,0%	0,0%	0,0%	0,0%	0,0%	0,0%	0,0%	Bacteria	Actinobacteria	Thermoleophilia	Solirubrobacterales	Patulibacteraceae	Patulibacter	-	napA	narG	-	-	-	-	-	-	-	-	-	ureA	-	-	-	-	-	mmoX	-	-	-	-	-	-
1097610	0,0%	0,0%	0,0%	0,0%	0,0%	0,0%	0,0%	0,0%	0,0%	Bacteria	Proteobacteria	Alphaproteobacteria	Rhizobiales		Methylocella	-	napA	narG	-	-	-	nirK	norB	-	nifH	nifD	-	ureA	-	-	-	-	-	mmoX	-	laccase_Asco	laccase_Basidio	-	-	ppo
1701833	0,0%	0,0%	0,0%	0,0%	0,0%	0,0%	0,0%	0,0%	0,0%	Bacteria	Elusimicrobia	Elusimicrobia	Elusimicrobiales		uncultured bacterium	-	-	-	-	-	-	-	-	-	-	-	-	-	-	-	-	-	-	-	-	-	-	-	-	-
2773722	0,0%	0,0%	0,0%	0,0%	0,0%	0,0%	0,0%	0,1%	0,0%	Bacteria	Actinobacteria	Actinobacteria	Actinomycetales		uncultured	-	-	-	-	-	-	-	-	-	-	-	-	-	-	-	-	-	-	-	-	-	-	-	lip	-
1142584	0,0%	0,0%	0,0%	0,0%	0,0%	0,0%	0,0%	0,0%	0,0%	Bacteria	Proteobacteria	Alphaproteobacteria	Rhodospirillales	Rhodospirillaceae	uncultured bacterium	-	-	-	-	-	-	-	-	-	-	-	-	-	-	-	-	-	-	-	-	-	-	-	-	-
1009763	0,0%	0,0%	0,0%	0,0%	0,0%	0,0%	0,0%	0,0%	0,0%	Bacteria	Planctomycetes	Planctomycetia	Gemmatales	Gemmataceae	uncultured	-	-	-	-	-	-	-	-	-	-	-	-	-	-	-	-	-	-	-	-	-	-	-	lip	-
4448095	0,0%	0,0%	0,0%	0,0%	0,0%	0,0%	0,0%	0,0%	0,0%	Bacteria	Actinobacteria	Actinobacteria	Actinomycetales	Mycobacteriaceae	uncultured bacterium	-	-	-	-	-	-	-	-	-	-	-	-	-	-	-	-	-	-	-	-	-	-	-	-	-
4419276	0,0%	0,0%	0,0%	0,0%	0,0%	0,0%	0,0%	0,0%	0,0%	Bacteria	Proteobacteria	Gammaproteobacteria	Pseudomonadales	Pseudomonadaceae	Pseudomonas	cellulolytic	napA	narG	nirA	-	nirS	nirK	norB	nosZ	nifH	nifD	-	ureA	-	dsrB	SoxB	-	-	mmoX	-	laccase_Asco	laccase_Basidio	-	-	ppo
582391	0,0%	0,0%	0,0%	0,0%	0,0%	0,0%	0,0%	0,0%	0,0%	Bacteria	Acidobacteria	Acidobacteria-6	iii1-15		uncultured bacterium	-	-	-	-	-	-	-	-	-	-	-	-	-	-	-	-	-	-	-	-	-	-	-	-	-
4354822	0,0%	0,0%	0,0%	0,0%	0,0%	0,0%	0,0%	0,0%	0,0%	Bacteria	Acidobacteria	Holophagae	Holophagales	Holophagaceae	Geothrix	-	-	-	-	-	-	-	-	-	-	-	-	-	-	-	-	-	-	-	-	-	-	-	-	-
376348	0,0%	0,0%	0,0%	0,0%	0,0%	0,0%	0,0%	0,0%	0,0%	Bacteria	Proteobacteria	Deltaproteobacteria	Myxococcales		Phaselicystis	-	-	-	-	-	-	-	-	-	-	-	-	-	-	-	-	-	-	-	-	-	-	-	-	-
568179	0,0%	0,0%	0,0%	0,0%	0,0%	0,0%	0,0%	0,0%	0,0%	Bacteria	Proteobacteria	Alphaproteobacteria	Rhodospirillales	Acetobacteraceae	Rhodopila	-	-	-	-	-	-	-	-	-	-	-	-	-	-	-	-	-	-	-	-	-	-	-	-	-
4321318	0,0%	0,0%	0,0%	0,0%	0,0%	0,0%	0,0%	0,0%	0,0%	Bacteria	Proteobacteria	Alphaproteobacteria	Rhodospirillales	Acetobacteraceae	uncultured	-	-	-	-	-	-	-	-	-	-	-	-	-	-	-	-	-	-	-	-	-	-	-	lip	-
2391398	0,0%	0,0%	0,0%	0,0%	0,0%	0,0%	0,0%	0,0%	0,0%	Bacteria	Verrucomicrobia	[Spartobacteria]	[Chthoniobacterales]	[Chthoniobacteraceae]	Chthoniobacter	-	napA	-	-	-	-	nirK	-	-	-	-	nrfA	ureA	-	-	-	-	-	-	-	-	-	-	-	-
4345285	0,0%	0,0%	0,0%	0,0%	0,0%	0,0%	0,0%	0,0%	0,0%	Bacteria	Firmicutes	Bacilli	Bacillales	Staphylococcaceae	Staphylococcus	-	napA	narG	-	-	-	nirK	norB	-	-	-	-	ureA	-	-	-	-	-	-	-	laccase_Asco	laccase_Basidio	-	-	-
4042106	0,0%	0,0%	0,0%	0,0%	0,0%	0,0%	0,0%	0,0%	0,0%	Bacteria	Bacteroidetes	Sphingobacteriia	Sphingobacteriales	Sphingobacteriaceae	Mucilaginibacter	-	-	-	-	-	-	-	-	-	-	-	-	-	-	-	-	-	-	-	-	-	-	-	-	-
4460853	0,0%	0,0%	0,0%	0,0%	0,0%	0,0%	0,0%	0,0%	0,0%	Bacteria	Actinobacteria	Actinobacteria	Actinomycetales	Nocardiaceae	Rhodococcus	-	napA	narG	nirA	-	-	nirK	norB	-	-	-	-	ureA	-	-	-	-	-	mmoX	-	laccase_Asco	laccase_Basidio	-	-	ppo

3542602	0,0%	0,0%	0,0%	0,0%	0,0%	0,0%	0,0%	0,0%	0,0%	Bacteria	Proteobacteria	Gammaproteobacteria	Legionellales	Legionellaceae	Legionella	-	-	-	-	-	nirS	-	norB	-	-	-	-	-	-	-	-	-	-	-	-	laccase_Asco	laccase_Basidio	-	-	ppo
1029521	0,0%	0,0%	0,0%	0,0%	0,0%	0,0%	0,0%	0,0%	0,0%	Bacteria	Proteobacteria	Betaproteobacteria	Rhodocyclales	Rhodocyclaceae	Dechloromonas	-	napA	narG	-	-	nirS	-	norB	nosZ	nifH	nifD	-	ureA	-	-	SoxB	-	-	mmoX	-	-	-	-	-	-
3682324	0,0%	0,0%	0,0%	0,0%	0,0%	0,0%	0,0%	0,0%	0,0%	Bacteria	Proteobacteria	Betaproteobacteria	Burkholderiales	Comamonadaceae	Rhizobacter	-	napA	narG	nirA	-	-	-	norB	-	-	-	-	ureA	-	-	-	-	-	-	-	-	-	-	-	-
90217	0,0%	0,0%	0,0%	0,0%	0,0%	0,0%	0,0%	0,0%	0,0%	Bacteria	WPS-2				uncultured bacterium	-	-	-	-	-	-	-	-	-	-	-	-	-	-	-	-	-	-	-	-	-	-	-	-	-
254402	0,0%	0,0%	0,0%	0,0%	0,0%	0,0%	0,0%	0,0%	0,0%	Bacteria	Planctomycetes	Planctomycetia	Gemmatales	Gemmataceae	uncultured	-	-	-	-	-	-	-	-	-	-	-	-	-	-	-	-	-	-	-	-	-	-	-	lip	-
224222	0,0%	0,0%	0,0%	0,0%	0,0%	0,0%	0,0%	0,0%	0,0%	Bacteria	Firmicutes	Clostridia	Clostridiales	[Tissierellaceae]	Anaerococcus	-	napA	-	-	-	-	-	-	-	-	-	nrfA	ureA	-	-	-	-	-	-	-	-	-	-	-	-
102295	0,0%	0,0%	0,0%	0,0%	0,0%	0,0%	0,0%	0,1%	0,0%	Bacteria	Proteobacteria	Alphaproteobacteria	Rhizobiales	Methylocystaceae	uncultured bacterium	-	-	-	-	-	-	-	-	-	-	-	-	-	-	-	-	-	-	-	-	-	-	-	-	-
113368	0,0%	0,0%	0,0%	0,0%	0,0%	0,0%	0,0%	0,0%	0,0%	Bacteria	Proteobacteria	Deltaproteobacteria			uncultured bacterium	-	-	-	-	-	-	-	-	-	-	-	-	-	-	-	-	-	-	-	-	-	-	-	-	-
512153	0,0%	0,0%	0,0%	0,0%	0,0%	0,0%	0,0%	0,0%	0,0%	Bacteria	Verrucomicrobia	[Pedosphaerae]	[Pedosphaerales]	[Pedosphaeraceae]	uncultured bacterium	-	-	-	-	-	-	-	-	-	-	-	-	-	-	-	-	-	-	-	-	-	-	-	-	-
4416962	0,0%	0,0%	0,0%	0,0%	0,0%	0,0%	0,0%	0,0%	0,0%	Bacteria	Bacteroidetes	[Saprospirae]	[Saprospirales]	Chitinophagaceae	uncultured	-	-	-	-	-	-	-	-	-	-	-	-	-	-	-	-	-	-	-	-	-	-	-	lip	-
676616	0,0%	0,0%	0,0%	0,0%	0,0%	0,0%	0,0%	0,0%	0,0%	Bacteria	Bacteroidetes	Flavobacteriia	Flavobacteriales	Cryomorphaceae	Fluviicola	-	-	-	nirA	-	-	-	norB	-	-	-	-	-	-	-	-	-	-	-	-	laccase_Asco	laccase_Basidio	-	-	ppo
216748	0,0%	0,0%	0,0%	0,0%	0,0%	0,0%	0,0%	0,0%	0,0%	Bacteria	Proteobacteria	Alphaproteobacteria	Rhizobiales	Hyphomicrobiaceae	Rhodoblastus	-	-	-	-	-	-	-	-	-	nifH	-	-	-	-	-	-	-	-	-	-	-	-	-	-	-
4416964	0,0%	0,0%	0,0%	0,0%	0,0%	0,0%	0,0%	0,0%	0,0%	Bacteria	Proteobacteria	Deltaproteobacteria	Desulfuromonadales	Geobacteraceae	Geobacter	-	napA	narG	-	-	-	-	norB	-	nifH	nifD	nrfA	-	-	-	-	-	-	-	-	laccase_Asco	laccase_Basidio	-	-	ppo
2125432	0,0%	0,0%	0,0%	0,0%	0,0%	0,0%	0,0%	0,0%	0,0%	Bacteria	Proteobacteria	Deltaproteobacteria	Bdellovibrionales	Bdellovibrionaceae	Bdellovibrio	-	-	-	-	-	-	nirK	norB	-	-	-	nrfA	-	-	-	-	-	-	-	-	laccase_Asco	laccase_Basidio	-	-	ppo
4480952	0,0%	0,0%	0,0%	0,0%	0,0%	0,0%	0,0%	0,0%	0,0%	Bacteria	Proteobacteria	Betaproteobacteria	Burkholderiales	Oxalobacteraceae	Janthinobacterium	-	napA	narG	nirA	-	nirS	-	norB	nosZ	-	-	-	ureA	-	-	SoxB	-	chb	-	-	-	laccase_Basidio	-	-	-
810287	0,0%	0,0%	0,0%	0,0%	0,0%	0,0%	0,0%	0,0%	0,0%	Bacteria	Proteobacteria	Alphaproteobacteria	Sphingomonadales		Altererythrobacter	-	napA	narG	-	-	-	-	-	-	-	-	-	-	-	-	-	-	-	-	-	laccase_Asco	laccase_Basidio	-	-	ppo
4029797	0,0%	0,0%	0,0%	0,0%	0,0%	0,0%	0,0%	0,1%	0,0%	Bacteria	Proteobacteria	Alphaproteobacteria	Rhodospirillales	Rhodospirillaceae	uncultured alpha proteobacterium	-	-	-	-	-	-	-	-	-	-	-	-	-	-	-	-	-	-	-	-	-	-	-	-	-
113214	0,0%	0,0%	0,0%	0,0%	0,0%	0,0%	0,0%	0,0%	0,0%	Bacteria	Verrucomicrobia	[Pedosphaerae]	[Pedosphaerales]	Ellin517	uncultured soil bacterium	-	-	-	-	-	-	-	-	-	-	-	-	-	-	-	-	-	-	-	-	-	-	-	-	-
1128399	0,0%	0,0%	0,0%	0,0%	0,0%	0,0%	0,0%	0,0%	0,0%	Bacteria	Proteobacteria	Gammaproteobacteria	Legionellales	Coxiellaceae	Aquicella	-	-	-	-	-	-	-	-	-	-	-	-	-	-	-	-	-	-	-	-	-	-	-	-	-
105470	0,0%	0,0%	0,0%	0,0%	0,0%	0,0%	0,0%	0,0%	0,0%	Bacteria	Proteobacteria	Alphaproteobacteria	Rhizobiales	Methylobacteriaceae	Methylobacterium	-	napA	narG	-	-	-	nirK	-	nosZ	nifH	nifD	-	ureA	-	-	-	-	-	-	-	laccase_Asco	laccase_Basidio	-	-	ppo
281742	0,0%	0,0%	0,0%	0,0%	0,0%	0,0%	0,0%	0,0%	0,0%	Bacteria	Proteobacteria	Alphaproteobacteria	Sphingomonadales	Sphingomonadaceae	Novosphingobium	-	napA	-	-	-	-	-	norB	-	nifH	nifD	-	ureA	-	-	-	-	-	mmoX	-	laccase_Asco	laccase_Basidio	-	-	ppo
855812	0,0%	0,0%	0,0%	0,0%	0,0%	0,0%	0,0%	0,0%	0,0%	Bacteria	Proteobacteria	Gammaproteobacteria	Legionellales	Legionellaceae	Legionella	-	-	-	-	-	nirS	-	norB	-	-	-	-	-	-	-	-	-	-	-	-	laccase_Asco	laccase_Basidio	-	-	ppo
41463	0,0%	0,0%	0,0%	0,0%	0,0%	0,0%	0,0%	0,0%	0,0%	Bacteria	Proteobacteria	Alphaproteobacteria	Rhizobiales	Hyphomicrobiaceae	Hyphomicrobium	-	napA	narG	nirA	-	-	nirK	norB	nosZ	nifH	nifD	-	ureA	-	-	-	-	-	mmoX	-	laccase_Asco	laccase_Basidio	-	-	ppo
4431590	0,0%	0,0%	0,0%	0,0%	0,0%	0,0%	0,0%	0,0%	0,0%	Bacteria	Actinobacteria	Actinobacteria	Actinomycetales	Kineosporiaceae	Kineococcus	-	napA	-	-	-	-	-	-	-	-	-	-	ureA	-	-	-	-	-	-	-	-	-	-	-	-
104612	0,0%	0,0%	0,0%	0,0%	0,0%	0,0%	0,0%	0,0%	0,0%	Bacteria	Proteobacteria	Betaproteobacteria	Rhodocyclales	Rhodocyclaceae	Uliginosibacterium	-	-	-	-	-	-	-	-	-	-	-	-	-	-	-	-	-	-	-	-	-	-	-	-	-
1862310	0,0%	0,0%	0,0%	0,0%	0,0%	0,0%	0,0%	0,0%	0,0%	Bacteria	Proteobacteria	Betaproteobacteria			Achromobacter	-	napA	narG	-	-	nirS	nirK	norB	nosZ	-	-	nrfA	ureA	-	-	-	-	-	mmoX	-	laccase_Asco	laccase_Basidio	-	-	ppo
153948	0,0%	0,0%	0,0%	0,0%	0,0%	0,0%	0,0%	0,0%	0,0%	Bacteria	Proteobacteria	Gammaproteobacteria	Legionellales	Legionellaceae	Massilia	-	napA	narG	-	-	-	-	norB	nosZ	-	-	-	ureA	-	-	-	-	chb	mmoX	-	laccase_Asco	laccase_Basidio	-	-	ppo
3778553	0,0%	0,0%	0,0%	0,0%	0,0%	0,0%	0,0%	0,0%	0,0%	Bacteria	Bacteroidetes	Flavobacteriia	Flavobacteriales	[Weeksellaceae]	Chryseobacterium	-	napA	-	-	-	-	nirK	norB	nosZ	nifH	-	-	ureA	-	-	-	-	chb	-	-	laccase_Asco	laccase_Basidio	-	-	ppo
306436	0,0%	0,0%	0,0%	0,0%	0,0%	0,0%	0,0%	0,0%	0,0%	Bacteria	Proteobacteria	Betaproteobacteria	Burkholderiales	Comamonadaceae	Variovorax	-	napA	narG	nirA	-	-	-	norB	-	-	-	-	ureA	-	-	-	-	-	mmoX	-	-	-	-	-	-
556364	0,0%	0,0%	0,0%	0,0%	0,0%	0,0%	0,0%	0,0%	0,0%	Bacteria	Proteobacteria	Deltaproteobacteria	Myxococcales	Myxococcaceae	Anaeromyxobacter	-	napA	narG	nirA	-	nirS	-	norB	nosZ	nifH	nifD	nrfA	ureA	-	-	SoxB	-	-	-	-	-	laccase_Basidio	-	-	-
4334052	0,0%	0,0%	0,0%	0,0%	0,0%	0,0%	0,0%	0,0%	0,0%	Bacteria	Proteobacteria	Gammaproteobacteria	Aeromonadales	Aeromonadaceae	uncultured bacterium	-	-	-	-	-	-	-	-	-	-	-	-	-	-	-	-	-	-	-	-	-	-	-	-	-
4248179	0,0%	0,0%	0,0%	0,0%	0,0%	0,0%	0,0%	0,0%	0,0%	Bacteria	BRC1	PRR-11			uncultured bacterium	-	-	-	-	-	-	-	-	-	-	-	-	-	-	-	-	-	-	-	-	-	-	-	-	-
1898675	0,0%	0,0%	0,0%	0,0%	0,0%	0,0%	0,0%	0,0%	0,0%	Bacteria	Proteobacteria	Betaproteobacteria	Burkholderiales	Comamonadaceae	Ramlibacter	-	napA	-	nirA	-	-	-	-	-	-	-	-	ureA	-	-	-	-	-	-	-	laccase_Asco	laccase_Basidio	-	-	ppo
565046	0,0%	0,0%	0,0%	0,0%	0,0%	0,0%	0,0%	0,0%	0,0%	Bacteria	Proteobacteria	Deltaproteobacteria	Myxococcales	Polyangiaceae	Chondromyces	-	napA	-	-	-	-	-	-	-	-	-	-	ureA	-	-	-	-	chb	-	-	laccase_Asco	laccase_Basidio	-	-	ppo
3552535	0,0%	0,0%	0,0%	0,0%	0,0%	0,0%	0,0%	0,0%	0,0%	Bacteria	Proteobacteria	Alphaproteobacteria	Rhizobiales	Bradyrhizobiaceae	Nitrobacter	-	napA	narG	-	nxrB	-	nirK	-	-	-	-	-	-	-	-	SoxB	-	-	-	-	laccase_Asco	laccase_Basidio	-	-	ppo
4389053	0,0%	0,0%	0,0%	0,0%	0,0%	0,0%	0,0%	0,0%	0,0%	Bacteria	Proteobacteria	Betaproteobacteria	Neisseriales	Neisseriaceae	uncultured	-	-	-	-	-	-	-	-	-	-	-	-	-	-	-	-	-	-	-	-	-	-	-	lip	-
695365	0,0%	0,0%	0,0%	0,0%	0,0%	0,0%	0,0%	0,0%	0,0%	Bacteria	Proteobacteria	Betaproteobacteria	Burkholderiales	Comamonadaceae	Rhizobacter	-	napA	narG	nirA	-	-	-	norB	-	-	-	-	ureA	-	-	-	-	-	-	-	-	-	-	-	-
333495	0,0%	0,0%	0,0%	0,0%	0,0%	0,0%	0,0%	0,0%	0,0%	Bacteria	Proteobacteria	Betaproteobacteria	Burkholderiales	Comamonadaceae	Aquincola	-	-	-	-	-	-	-	-	-	-	-	-	-	-	-	-	-	-	-	-	-	-	-	-	-
840275	0,0%	0,0%	0,0%	0,0%	0,0%	0,0%	0,0%	0,0%	0,0%	Bacteria	Actinobacteria	Acidimicrobiia	Acidimicrobiales		uncultured bacterium	-	-	-	-	-	-	-	-	-	-	-	-	-	-	-	-	-	-	-	-	-	-	-	-	-
1122860	0,0%	0,0%	0,0%	0,0%	0,0%	0,0%	0,0%	0,0%	0,0%	Bacteria	Proteobacteria	Alphaproteobacteria	Rhodobacterales	Hyphomonadaceae	Woodsholea	-	-	-	-	-	-	-	-	-	-	-	-	-	-	-	-	-	-	-	-	-	-	-	-	-
4304512	0,0%	0,0%	0,0%	0,0%	0,0%	0,0%	0,0%	0,0%	0,0%	Bacteria	Proteobacteria	Alphaproteobacteria	Caulobacterales	Caulobacteraceae	Brevundimonas	-	-	-	nirA	-	-	-	norB	-	nifH	-	-	-	-	-	-	-	-	mmoX	-	laccase_Asco	laccase_Basidio	-	-	ppo
715800	0,0%	0,0%	0,0%	0,0%	0,0%	0,0%	0,0%	0,0%	0,0%	Bacteria	Verrucomicrobia	Opitutae	Opitutales	Opitutaceae	Opitutus	-	napA	-	nirA	-	-	-	-	nosZ	-	-	nrfA	ureA	-	-	-	-	-	-	-	-	laccase_Basidio	-	-	-
4446058	0,0%	0,0%	0,0%	0,0%	0,0%	0,0%	0,0%	0,0%	0,0%	Bacteria	Firmicutes	Bacilli	Bacillales	Staphylococcaceae	Staphylococcus	-	napA	narG	-	-	-	nirK	norB	-	-	-	-	ureA	-	-	-	-	-	-	-	laccase_Asco	laccase_Basidio	-	-	-
2954724	0,0%	0,0%	0,0%	0,0%	0,0%	0,0%	0,0%	0,0%	0,0%	Bacteria	Proteobacteria	Alphaproteobacteria	Rhodospirillales	Acetobacteraceae	uncultured	-	-	-	-	-	-	-	-	-	-	-	-	-	-	-	-	-	-	-	-	-	-	-	lip	-
4322321	0,0%	0,0%	0,0%	0,0%	0,0%	0,0%	0,0%	0,0%	0,0%	Bacteria	Cyanobacteria	Oscillatoriophycideae	Oscillatoriales	Phormidiaceae	Phormidium	-	napA	-	nirA	-	-	-	norB	-	-	nifD	-	ureA	-	-	-	-	-	-	-	-	-	-	-	-
3847840	0,0%	0,0%	0,0%	0,0%	0,0%	0,0%	0,0%	0,0%	0,0%	Bacteria	Proteobacteria	Betaproteobacteria	Neisseriales	Neisseriaceae	Vogesella	-	napA	narG	-	-	nirS	-	-	-	-	-	-	-	-	-	-	-	-	-	-	laccase_Asco	laccase_Basidio	-	-	ppo
3214060	0,0%	0,0%	0,0%	0,0%	0,0%	0,0%	0,0%	0,0%	0,0%	Bacteria	Bacteroidetes	Flavobacteriia	Flavobacteriales	Flavobacteriaceae	Dokdonia	cellulolytic	-	-	-	-	-	-	-	-	-	-	-	-	-	-	-	-	-	-	-	-	-	-	-	-
540593	0,0%	0,0%	0,0%	0,0%	0,0%	0,0%	0,0%	0,0%	0,0%	Bacteria	Acidobacteria	Solibacteres	Solibacterales		Bryobacter	-	-	-	-	-	-	-	-	-	-	-	-	-	-	-	-	-	-	-	-	-	-	-	-	-
4477202	0,0%	0,0%	0,0%	0,0%	0,0%	0,0%	0,0%	0,0%	0,0%	Bacteria	Bacteroidetes	Sphingobacteriia	Sphingobacteriales		uncultured bacterium	-	-	-	-	-	-	-	-	-	-	-	-	-	-	-	-	-	-	-	-	-	-	-	-	-
4408820	0,0%	0,0%	0,0%	0,0%	0,0%	0,0%	0,0%	0,0%	0,0%	Bacteria	Proteobacteria	Deltaproteobacteria	Desulfuromonadales	Geobacteraceae	uncultured bacterium	-	-	-	-	-	-	-	-	-	-	-	-	-	-	-	-	-	-	-	-	-	-	-	-	-
4389705	0,0%	0,0%	0,0%	0,0%	0,0%	0,0%	0,0%	0,0%	0,0%	Bacteria	Proteobacteria	Betaproteobacteria	Burkholderiales	Comamonadaceae	uncultured	-	-	-	-	-	-	-	-	-	-	-	-	-	-	-	-	-	-	-	-	-	-	-	lip	-
1052283	0,0%	0,0%	0,0%	0,0%	0,0%	0,0%	0,0%	0,0%	0,0%	Bacteria	Planctomycetes	Planctomycetia	Gemmatales	Isosphaeraceae	uncultured	-	-	-	-	-	-	-	-	-	-	-	-	-	-	-	-	-	-	-	-	-	-	-	lip	-
4480536	0,0%	0,0%	0,0%	0,0%	0,0%	0,0%	0,0%	0,0%	0,0%	Bacteria	Proteobacteria	Gammaproteobacteria	Thiotrichales	Piscirickettsiaceae	uncultured bacterium	-	-	-	-	-	-	-	-	-	-	-	-	-	-	-	-	-	-	-	-	-	-	-	-	-
735087	0,0%	0,0%	0,0%	0,0%	0,0%	0,0%	0,0%	0,0%	0,0%	Bacteria	Bacteroidetes	Cytophagia	Cytophagales	Cytophagaceae	uncultured	cellulolytic	-	-	-	-	-	-	-	-	-	-	-	-	-	-	-	-	-	-	-	-	-	-	lip	-
2655357	0,0%	0,0%	0,0%	0,0%	0,0%	0,0%	0,0%	0,0%	0,0%	Bacteria	Proteobacteria	Betaproteobacteria	Burkholderiales	Comamonadaceae	uncultured	-	-	-	-	-	-	-	-	-	-	-	-	-	-	-	-	-	-	-	-	-	-	-	lip	-
94511	0,0%	0,0%	0,0%	0,0%	0,0%	0,0%	0,0%	0,0%	0,0%	Bacteria	Proteobacteria	Alphaproteobacteria			uncultured	-	-	-	-	-	-	-	-	-	-	-	-	-	-	-	-	-	-	-	-	-	-	-	lip	-
218567	0,0%	0,0%	0,0%	0,0%	0,0%	0,0%	0,0%	0,0%	0,0%	Bacteria	Proteobacteria	Deltaproteobacteria	Myxococcales		Sorangium	-	napA	-	-	-	-	nirK	norB	-	-	-	nrfA	ureA	-	-	-	-	chb	-	-	laccase_Asco	laccase_Basidio	-	-	ppo
1029421	0,0%	0,0%	0,0%	0,0%	0,0%	0,0%	0,0%	0,0%	0,0%	Bacteria	Bacteroidetes	[Saprospirae]	[Saprospirales]	Chitinophagaceae	uncultured	-	-	-	-	-	-	-	-	-	-	-	-	-	-	-	-	-	-	-	-	-	-	-	lip	-
83643	0,0%	0,0%	0,0%	0,0%	0,0%	0,0%	0,0%	0,0%	0,0%	Bacteria	Proteobacteria	Alphaproteobacteria	Rhodobacterales	Rhodobacteraceae	Rhodobacter	-	napA	-	nirA	-	nirS	nirK	norB	nosZ	nifH	nifD	-	ureA	-	-	SoxB	-	-	mmoX	-	laccase_Asco	laccase_Basidio	-	-	ppo
4426961	0,0%	0,0%	0,0%	0,0%	0,0%	0,0%	0,0%	0,0%	0,0%	Bacteria	Proteobacteria	Betaproteobacteria	Burkholderiales	Comamonadaceae	Variovorax	-	napA	narG	nirA	-	-	-	norB	-	-	-	-	ureA	-	-	-	-	-	mmoX	-	-	-	-	-	-
771632	0,0%	0,0%	0,0%	0,0%	0,0%	0,0%	0,0%	0,0%	0,0%	Bacteria	Proteobacteria	Alphaproteobacteria	Sphingomonadales	Sphingomonadaceae	Sandarakinorhabdus	-	-	-	-	-	-	-	-	-	-	-	-	-	-	-	-	-	-	-	-	-	-	-	-	-
156660	0,0%	0,0%	0,0%	0,0%	0,0%	0,0%	0,0%	0,0%	0,0%	Bacteria	Verrucomicrobia	[Methylacidiphilae]	Methylacidiphilales		Candidatus Methylacidiphilum	-	-	-	-	-	-	-	-	-	-	-	-	-	-	-	-	-	-	-	-	-	-	-	-	-
1115336	0,0%	0,0%	0,0%	0,0%	0,0%	0,0%	0,0%	0,0%	0,0%	Bacteria	Proteobacteria	Gammaproteobacteria	Aeromonadales	Aeromonadaceae	uncultured bacterium	-	-	-	-	-	-	-	-	-	-	-	-	-	-	-	-	-	-	-	-	-	-	-	-	-
1115110	0,0%	0,0%	0,0%	0,0%	0,0%	0,0%	0,0%	0,0%	0,0%	Bacteria	Proteobacteria	Deltaproteobacteria	Myxococcales		Sorangium	-	napA	-	-	-	-	nirK	norB	-	-	-	nrfA	ureA	-	-	-	-	chb	-	-	laccase_Asco	laccase_Basidio	-	-	ppo
589382	0,0%	0,0%	0,0%	0,0%	0,0%	0,0%	0,0%	0,0%	0,0%	Bacteria	Proteobacteria	Alphaproteobacteria	Caulobacterales	Caulobacteraceae	uncultured	-	-	-	-	-	-	-	-	-	-	-	-	-	-	-	-	-	-	-	-	-	-	-	lip	-
239649	0,0%	0,0%	0,0%	0,0%	0,0%	0,0%	0,0%	0,0%	0,0%	Bacteria	Acidobacteria	Acidobacteriia	Acidobacteriales	Acidobacteriaceae	Granulicella	cellulolytic	napA	-	-	-	-	-	-	-	-	-	-	-	-	-	-	-	-	-	-	laccase_Asco	laccase_Basidio	-	-	ppo
3239984	0,0%	0,0%	0,0%	0,0%	0,0%	0,0%	0,0%	0,0%	0,0%	Bacteria	Proteobacteria	Alphaproteobacteria	Rhodobacterales	Rhodobacteraceae	Amaricoccus	-	-	-	-	-	-	-	-	-	-	-	-	-	-	-	-	-	-	-	-	-	-	-	-	-
540202	0,0%	0,0%	0,0%	0,0%	0,0%	0,0%	0,0%	0,0%	0,0%	Bacteria	Proteobacteria	Gammaproteobacteria	Chromatiales	Chromatiaceae	Thiocystis	-	napA	-	-	-	-	-	norB	-	nifH	nifD	-	ureA	dsrA	dsrB	-	-	-	-	-	-	-	-	-	-
3125462	0,0%	0,0%	0,0%	0,0%	0,0%	0,0%	0,0%	0,0%	0,0%	Bacteria	Bacteroidetes	Bacteroidia	Bacteroidales		uncultured bacterium	-	-	-	-	-	-	-	-	-	-	-	-	-	-	-	-	-	-	-	-	-	-	-	-	-
4426965	0,0%	0,0%	0,0%	0,0%	0,0%	0,0%	0,0%	0,0%	0,0%	Bacteria	Proteobacteria	Betaproteobacteria			OM43 clade	-	-	-	-	-	-	-	-	-	-	-	-	-	-	-	-	-	-	-	-	-	-	-	-	-
3447865	0,0%	0,0%	0,0%	0,0%	0,0%	0,0%	0,0%	0,0%	0,0%	Bacteria	Bacteroidetes	Sphingobacteriia	Sphingobacteriales		uncultured bacterium	-	-	-	-	-	-	-	-	-	-	-	-	-	-	-	-	-	-	-	-	-	-	-	-	-
3447863	0,0%	0,0%	0,0%	0,0%	0,0%	0,0%	0,0%	0,0%	0,0%	Bacteria	Bacteroidetes	[Saprospirae]	[Saprospirales]	Chitinophagaceae	Sediminibacterium	-	-	-	-	-	-	-	-	-	-	-	-	-	-	-	-	-	-	-	-	-	-	-	-	-
1107044	0,0%	0,0%	0,0%	0,0%	0,0%	0,0%	0,0%	0,0%	0,0%	Bacteria	Verrucomicrobia	[Pedosphaerae]	[Pedosphaerales]	auto67_4W	uncultured Verrucomicrobia bacterium	-	-	-	-	-	-	-	-	-	-	-	-	-	-	-	-	-	-	-	-	-	-	-	-	-
315994	0,0%	0,0%	0,0%	0,0%	0,0%	0,0%	0,0%	0,0%	0,0%	Bacteria	Proteobacteria	Gammaproteobacteria	Legionellales	Legionellaceae	Legionella	-	-	-	-	-	nirS	-	norB	-	-	-	-	-	-	-	-	-	-	-	-	laccase_Asco	laccase_Basidio	-	-	ppo
4328432	0,0%	0,0%	0,0%	0,0%	0,0%	0,0%	0,0%	0,0%	0,0%	Bacteria	Proteobacteria	Alphaproteobacteria	Rhizobiales	Methylobacteriaceae	Methylobacterium	-	napA	narG	-	-	-	nirK	-	nosZ	nifH	nifD	-	ureA	-	-	-	-	-	-	-	laccase_Asco	laccase_Basidio	-	-	ppo
109707	0,0%	0,0%	0,0%	0,0%	0,0%	0,0%	0,0%	0,0%	0,0%	Bacteria	Planctomycetes	Phycisphaerae	WD2101		uncultured bacterium	-	-	-	-	-	-	-	-	-	-	-	-	-	-	-	-	-	-	-	-	-	-	-	-	-
631818	0,0%	0,0%	0,0%	0,0%	0,0%	0,0%	0,0%	0,0%	0,0%	Bacteria	Proteobacteria	Gammaproteobacteria	Alteromonadales	Alteromonadaceae	Marinobacter	-	napA	narG	nirA	-	nirS	nirK	norB	nosZ	-	nifD	-	ureA	-	-	-	-	-	mmoX	-	laccase_Asco	laccase_Basidio	-	-	ppo
4373022	0,0%	0,0%	0,0%	0,0%	0,0%	0,0%	0,0%	0,0%	0,0%	Bacteria	TM6	SJA-4			uncultured organism	-	-	-	-	-	-	-	-	-	-	-	-	-	-	-	-	-	-	-	-	-	-	-	-	-
544356	0,0%	0,0%	0,0%	0,0%	0,0%	0,0%	0,0%	0,0%	0,0%	Bacteria	Proteobacteria	Betaproteobacteria	Burkholderiales	Comamonadaceae	uncultured	-	-	-	-	-	-	-	-	-	-	-	-	-	-	-	-	-	-	-	-	-	-	-	lip	-
4394922	0,0%	0,0%	0,0%	0,0%	0,0%	0,0%	0,0%	0,0%	0,0%	Bacteria	Proteobacteria	Betaproteobacteria	Ellin6067		uncultured	-	-	-	-	-	-	-	-	-	-	-	-	-	-	-	-	-	-	-	-	-	-	-	lip	-
4453194	0,0%	0,0%	0,0%	0,0%	0,0%	0,0%	0,0%	0,0%	0,0%	Bacteria	Proteobacteria	Alphaproteobacteria	Rhizobiales		uncultured	-	-	-	-	-	-	-	-	-	-	-	-	-	-	-	-	-	-	-	-	-	-	-	lip	-
4417475	0,0%	0,0%	0,0%	0,0%	0,0%	0,0%	0,0%	0,0%	0,0%	Bacteria	Proteobacteria	Alphaproteobacteria	Ellin329		Candidatus Liberibacter	-	-	-	-	-	-	-	-	-	-	-	-	-	-	-	-	-	-	-	-	-	-	-	-	-
2720390	0,0%	0,0%	0,0%	0,0%	0,0%	0,0%	0,0%	0,0%	0,0%	Bacteria	Proteobacteria	Alphaproteobacteria			uncultured bacterium	-	-	-	-	-	-	-	-	-	-	-	-	-	-	-	-	-	-	-	-	-	-	-	-	-
216235	0,0%	0,0%	0,0%	0,0%	0,0%	0,0%	0,0%	0,0%	0,0%	Bacteria	Proteobacteria	Betaproteobacteria	Burkholderiales	Comamonadaceae	Acidovorax	-	napA	narG	nirA	-	nirS	nirK	norB	nosZ	-	-	-	ureA	-	-	-	-	-	mmoX	-	-	laccase_Basidio	-	-	ppo
4418495	0,0%	0,0%	0,0%	0,0%	0,0%	0,0%	0,0%	0,0%	0,0%	Bacteria	Proteobacteria	Alphaproteobacteria	BD7-3		uncultured bacterium	-	-	-	-	-	-	-	-	-	-	-	-	-	-	-	-	-	-	-	-	-	-	-	-	-
4301105	0,0%	0,0%	0,0%	0,0%	0,0%	0,0%	0,0%	0,0%	0,0%	Bacteria	Actinobacteria	Actinobacteria	Actinomycetales	Mycobacteriaceae	uncultured Mycobacterium sp.	-	-	-	-	-	-	-	-	-	-	-	-	-	-	-	-	-	-	-	-	-	-	-	-	-
4453355	0,0%	0,0%	0,0%	0,0%	0,0%	0,0%	0,0%	0,0%	0,0%	Bacteria	Proteobacteria	Betaproteobacteria	Burkholderiales	Oxalobacteraceae	Massilia	-	napA	narG	-	-	-	-	norB	nosZ	-	-	-	ureA	-	-	-	-	chb	mmoX	-	laccase_Asco	laccase_Basidio	-	-	ppo
170172	0,0%	0,0%	0,0%	0,0%	0,0%	0,0%	0,0%	0,0%	0,0%	Bacteria	Proteobacteria	Alphaproteobacteria	Rhodospirillales	Rhodospirillaceae	Telmatospirillum	-	-	-	-	-	-	-	-	-	nifH	-	-	-	-	-	-	-	-	-	-	-	-	-	-	-
222900	0,0%	0,0%	0,0%	0,0%	0,0%	0,0%	0,0%	0,0%	0,0%	Bacteria	Proteobacteria	Deltaproteobacteria	Myxococcales	Polyangiaceae	Sorangium	-	napA	-	-	-	-	nirK	norB	-	-	-	nrfA	ureA	-	-	-	-	chb	-	-	laccase_Asco	laccase_Basidio	-	-	ppo
217349	0,0%	0,0%	0,0%	0,0%	0,0%	0,0%	0,0%	0,0%	0,0%	Bacteria	Planctomycetes	BD7-11			uncultured bacterium	-	-	-	-	-	-	-	-	-	-	-	-	-	-	-	-	-	-	-	-	-	-	-	-	-
762797	0,0%	0,0%	0,0%	0,0%	0,0%	0,0%	0,0%	0,0%	0,0%	Bacteria	Proteobacteria	Alphaproteobacteria	Rhizobiales	Hyphomicrobiaceae	Hyphomicrobium	-	napA	narG	nirA	-	-	nirK	norB	nosZ	nifH	nifD	-	ureA	-	-	-	-	-	mmoX	-	laccase_Asco	laccase_Basidio	-	-	ppo
223237	0,0%	0,0%	0,0%	0,0%	0,0%	0,0%	0,0%	0,0%	0,0%	Bacteria	Proteobacteria	Alphaproteobacteria			uncultured bacterium	-	-	-	-	-	-	-	-	-	-	-	-	-	-	-	-	-	-	-	-	-	-	-	-	-
833957	0,0%	0,0%	0,0%	0,0%	0,0%	0,0%	0,0%	0,0%	0,0%	Bacteria	Chloroflexi	Ellin6529			uncultured bacterium	-	-	-	-	-	-	-	-	-	-	-	-	-	-	-	-	-	-	-	-	-	-	-	-	-
235634	0,0%	0,0%	0,0%	0,0%	0,0%	0,0%	0,0%	0,0%	0,0%	Bacteria	Proteobacteria	Gammaproteobacteria	Xanthomonadales	Sinobacteraceae	uncultured bacterium	-	-	-	-	-	-	-	-	-	-	-	-	-	-	-	-	-	-	-	-	-	-	-	-	-
4397023	0,0%	0,0%	0,0%	0,0%	0,0%	0,0%	0,0%	0,0%	0,0%	Bacteria	Proteobacteria	Betaproteobacteria	Rhodocyclales	Rhodocyclaceae	Denitratisoma	-	-	-	-	-	-	-	-	-	-	-	-	-	-	-	-	-	-	-	-	-	-	-	-	-
1135313	0,0%	0,0%	0,0%	0,0%	0,0%	0,0%	0,0%	0,0%	0,0%	Bacteria	Bacteroidetes	Sphingobacteriia	Sphingobacteriales	Sphingobacteriaceae	Mucilaginibacter	-	-	-	-	-	-	-	-	-	-	-	-	-	-	-	-	-	-	-	-	-	-	-	-	-
913174	0,0%	0,0%	0,0%	0,0%	0,0%	0,0%	0,0%	0,0%	0,0%	Bacteria	Bacteroidetes	Cytophagia	Cytophagales	Cytophagaceae	Leadbetterella	cellulolytic	napA	-	nirA	-	-	-	-	nosZ	-	-	-	-	-	-	-	-	chb	-	-	laccase_Asco	laccase_Basidio	-	-	ppo
446213	0,0%	0,0%	0,0%	0,0%	0,0%	0,0%	0,0%	0,0%	0,0%	Bacteria	Actinobacteria	Thermoleophilia	Solirubrobacterales		uncultured Actinomycetales bacterium	-	-	-	-	-	-	-	-	-	-	-	-	-	-	-	-	-	-	-	-	-	-	-	-	-
4318046	0,0%	0,0%	0,0%	0,0%	0,0%	0,0%	0,0%	0,0%	0,0%	Bacteria	Acidobacteria	Solibacteres	Solibacterales		uncultured Acidobacteria bacterium	-	-	-	-	-	-	-	-	-	-	-	-	-	-	-	-	-	-	-	-	-	-	-	-	-
151283	0,0%	0,0%	0,0%	0,0%	0,0%	0,0%	0,0%	0,0%	0,0%	Bacteria	Acidobacteria	Acidobacteriia	Acidobacteriales	Acidobacteriaceae	uncultured	cellulolytic	-	-	-	-	-	-	-	-	-	-	-	-	-	-	-	-	-	-	-	-	-	-	lip	-
541884	0,0%	0,0%	0,0%	0,0%	0,0%	0,0%	0,0%	0,0%	0,0%	Bacteria	Proteobacteria	Betaproteobacteria	Neisseriales	Neisseriaceae	Chitinimonas	-	-	-	-	-	-	-	-	-	-	-	-	-	-	-	-	-	-	-	-	-	-	-	-	-
4477244	0,0%	0,0%	0,0%	0,0%	0,0%	0,0%	0,0%	0,0%	0,0%	Bacteria	Proteobacteria	Gammaproteobacteria	Pseudomonadales	Moraxellaceae	Perlucidibaca	-	-	-	-	-	-	-	-	-	-	-	-	-	-	-	-	-	-	-	-	-	-	-	-	-
1113025	0,0%	0,0%	0,0%	0,0%	0,0%	0,0%	0,0%	0,0%	0,0%	Bacteria	Proteobacteria	Alphaproteobacteria	Rhodospirillales	Rhodospirillaceae	Inquilinus	-	-	-	-	-	-	-	-	-	-	-	-	-	-	-	-	-	-	-	-	-	-	-	-	-
156710	0,0%	0,0%	0,0%	0,0%	0,0%	0,0%	0,0%	0,0%	0,0%	Bacteria	Proteobacteria	Alphaproteobacteria	Rhodospirillales	Rhodospirillaceae	Elstera	-	-	-	-	-	-	-	-	-	-	-	-	-	-	-	-	-	-	-	-	-	-	-	-	-
221349	0,0%	0,0%	0,0%	0,0%	0,0%	0,0%	0,0%	0,0%	0,0%	Bacteria	Gemmatimonadetes	Gemmatimonadetes	N1423WL		uncultured	-	-	-	-	-	-	-	-	-	-	-	-	-	-	-	-	-	-	-	-	-	-	-	lip	-
735466	0,0%	0,0%	0,0%	0,0%	0,0%	0,0%	0,0%	0,0%	0,0%	Bacteria	Actinobacteria	Actinobacteria	Actinomycetales	Mycobacteriaceae	Nocardia	-	napA	narG	-	-	-	nirK	-	-	-	-	-	ureA	-	-	-	-	-	mmoX	-	laccase_Asco	laccase_Basidio	-	-	ppo
760885	0,0%	0,0%	0,0%	0,0%	0,0%	0,0%	0,0%	0,0%	0,0%	Bacteria	Proteobacteria	Gammaproteobacteria	Legionellales	Coxiellaceae	Aquicella	-	-	-	-	-	-	-	-	-	-	-	-	-	-	-	-	-	-	-	-	-	-	-	-	-
1114358	0,0%	0,0%	0,0%	0,0%	0,0%	0,0%	0,0%	0,0%	0,0%	Bacteria	Actinobacteria	Actinobacteria	Actinomycetales		uncultured	-	-	-	-	-	-	-	-	-	-	-	-	-	-	-	-	-	-	-	-	-	-	-	lip	-
1000986	0,0%	0,0%	0,0%	0,0%	0,0%	0,0%	0,0%	0,0%	0,0%	Bacteria	Actinobacteria	Actinobacteria	Actinomycetales	Corynebacteriaceae	Corynebacterium	-	napA	narG	-	-	-	nirK	norB	-	-	-	nrfA	ureA	-	-	-	-	-	-	-	laccase_Asco	laccase_Basidio	-	-	ppo
4478413	0,0%	0,0%	0,0%	0,0%	0,0%	0,0%	0,0%	0,0%	0,0%	Bacteria	Bacteroidetes	[Saprospirae]	[Saprospirales]	Chitinophagaceae	Chitinophaga	-	napA	-	-	-	-	-	norB	-	-	-	-	ureA	-	-	-	-	chb	-	-	laccase_Asco	laccase_Basidio	-	-	ppo
279874	0,0%	0,0%	0,0%	0,0%	0,0%	0,0%	0,0%	0,0%	0,0%	Bacteria	Cyanobacteria	ML635J-21			uncultured bacterium	-	-	-	-	-	-	-	-	-	-	-	-	-	-	-	-	-	-	-	-	-	-	-	-	-
3874043	0,0%	0,0%	0,0%	0,0%	0,0%	0,0%	0,0%	0,0%	0,0%	Bacteria	[Thermi]	Deinococci	Deinococcales	Deinococcaceae	Deinococcus	-	napA	-	-	-	-	nirK	-	nosZ	-	-	-	ureA	-	-	-	-	-	-	-	laccase_Asco	laccase_Basidio	-	-	ppo
590476	0,0%	0,0%	0,0%	0,0%	0,0%	0,0%	0,0%	0,0%	0,1%	Bacteria	Proteobacteria	Gammaproteobacteria			uncultured bacterium	-	-	-	-	-	-	-	-	-	-	-	-	-	-	-	-	-	-	-	-	-	-	-	-	-
2202350	0,0%	0,0%	0,0%	0,0%	0,0%	0,0%	0,0%	0,0%	0,0%	Bacteria	Firmicutes	Bacilli	Bacillales	Staphylococcaceae	Staphylococcus	-	napA	narG	-	-	-	nirK	norB	-	-	-	-	ureA	-	-	-	-	-	-	-	laccase_Asco	laccase_Basidio	-	-	-
4444215	0,0%	0,0%	0,0%	0,0%	0,0%	0,0%	0,0%	0,0%	0,0%	Bacteria	Firmicutes	Clostridia	Clostridiales	Lachnospiraceae	Incertae Sedis	cellulolytic	-	-	-	-	-	-	-	-	-	-	-	-	-	-	-	-	-	-	-	-	-	-	-	-
4476579	0,0%	0,0%	0,0%	0,0%	0,0%	0,0%	0,0%	0,0%	0,0%	Bacteria	Proteobacteria	Alphaproteobacteria	Rhizobiales	Hyphomicrobiaceae	uncultured	-	-	-	-	-	-	-	-	-	-	-	-	-	-	-	-	-	-	-	-	-	-	-	lip	-
4306200	0,0%	0,0%	0,0%	0,0%	0,0%	0,0%	0,0%	0,0%	0,0%	Bacteria	Proteobacteria	Betaproteobacteria	Burkholderiales	Oxalobacteraceae	Undibacterium	-	-	-	-	-	-	-	-	-	-	-	-	-	-	-	-	-	-	-	-	-	-	-	-	-
200854	0,0%	0,0%	0,0%	0,0%	0,0%	0,0%	0,0%	0,0%	0,0%	Bacteria	Planctomycetes	Planctomycetia	Gemmatales	Gemmataceae	Gemmata	-	-	-	-	-	-	-	-	-	-	-	-	-	-	-	-	-	-	-	-	-	-	-	-	-
4297171	0,0%	0,0%	0,0%	0,0%	0,0%	0,0%	0,0%	0,0%	0,0%	Bacteria	Proteobacteria	Betaproteobacteria	Burkholderiales	Comamonadaceae	Leptothrix	-	napA	-	-	-	nirS	-	norB	nosZ	nifH	nifD	-	ureA	-	-	-	-	-	mmoX	-	-	-	-	-	-
2473794	0,0%	0,0%	0,0%	0,0%	0,0%	0,0%	0,0%	0,0%	0,0%	Bacteria	Planctomycetes	Planctomycetia	Gemmatales	Isosphaeraceae	Isosphaera	-	napA	-	-	-	-	-	-	-	-	-	-	-	-	-	-	-	-	-	-	-	-	-	-	-
605399	0,0%	0,0%	0,0%	0,0%	0,0%	0,0%	0,0%	0,0%	0,0%	Bacteria	Proteobacteria	Betaproteobacteria	Burkholderiales	Comamonadaceae	Xylophilus	-	napA	-	nirA	-	-	-	-	-	-	-	-	ureA	-	-	-	-	-	-	-	-	-	-	-	-
1023075	0,0%	0,0%	0,0%	0,0%	0,0%	0,0%	0,0%	0,0%	0,0%	Bacteria	Firmicutes	Clostridia	Clostridiales	Veillonellaceae	Veillonella	-	napA	narG	-	-	-	-	norB	-	-	nifD	-	-	-	-	-	-	-	-	-	laccase_Asco	laccase_Basidio	-	-	ppo
4339720	0,0%	0,0%	0,0%	0,0%	0,0%	0,0%	0,0%	0,0%	0,0%	Bacteria	Actinobacteria	Actinobacteria	Actinomycetales	Microbacteriaceae	Schumannella	cellulolytic	-	-	-	-	-	-	-	-	-	-	-	-	-	-	-	-	-	-	-	-	-	-	-	-
3412843	0,0%	0,0%	0,0%	0,0%	0,0%	0,0%	0,0%	0,0%	0,0%	Bacteria	Proteobacteria	Alphaproteobacteria	Rhodospirillales	Rhodospirillaceae	Reyranella	-	-	-	-	-	-	-	-	-	-	-	-	-	-	-	-	-	-	-	-	-	-	-	-	-
881422	0,0%	0,0%	0,0%	0,0%	0,0%	0,0%	0,0%	0,0%	0,0%	Bacteria	Bacteroidetes	[Saprospirae]	[Saprospirales]	Chitinophagaceae	Ferruginibacter	-	-	-	-	-	-	-	-	-	-	-	-	-	-	-	-	-	-	-	-	-	-	-	-	-
609236	0,0%	0,0%	0,0%	0,0%	0,0%	0,0%	0,0%	0,0%	0,0%	Bacteria	FCPU426				uncultured bacterium	-	-	-	-	-	-	-	-	-	-	-	-	-	-	-	-	-	-	-	-	-	-	-	-	-
50624	0,0%	0,0%	0,0%	0,0%	0,0%	0,0%	0,0%	0,0%	0,0%	Bacteria	Proteobacteria	Betaproteobacteria	Methylophilales	Methylophilaceae	uncultured	-	-	-	-	-	-	-	-	-	-	-	-	-	-	-	-	-	-	-	-	-	-	-	lip	-
3580423	0,0%	0,0%	0,0%	0,0%	0,0%	0,0%	0,0%	0,0%	0,0%	Bacteria	Firmicutes	Clostridia	Clostridiales	Clostridiaceae	Fonticella	cellulolytic	-	-	-	-	-	-	-	-	-	-	-	-	-	-	-	-	-	-	-	-	-	-	-	-
4383705	0,0%	0,0%	0,0%	0,0%	0,0%	0,0%	0,0%	0,0%	0,0%	Bacteria	Proteobacteria	Gammaproteobacteria	Alteromonadales	HTCC2188	Pseudospirillum	-	-	-	-	-	-	-	-	-	-	-	-	-	-	-	-	-	-	-	-	-	-	-	-	-
60573	0,0%	0,0%	0,0%	0,0%	0,0%	0,0%	0,0%	0,0%	0,0%	Bacteria	Planctomycetes	OM190	CL500-15		uncultured bacterium	-	-	-	-	-	-	-	-	-	-	-	-	-	-	-	-	-	-	-	-	-	-	-	-	-
766306	0,0%	0,0%	0,0%	0,0%	0,0%	0,0%	0,0%	0,0%	0,0%	Bacteria	Proteobacteria	Betaproteobacteria	Burkholderiales	Comamonadaceae	uncultured	-	-	-	-	-	-	-	-	-	-	-	-	-	-	-	-	-	-	-	-	-	-	-	lip	-
4333206	0,0%	0,0%	0,0%	0,0%	0,0%	0,0%	0,0%	0,0%	0,0%	Bacteria	Proteobacteria	Alphaproteobacteria	Rhizobiales	Rhizobiaceae	Rhizobium	-	napA	narG	nirA	-	-	nirK	norB	nosZ	nifH	nifD	nrfA	ureA	-	-	-	-	chb	mmoX	-	laccase_Asco	laccase_Basidio	-	-	ppo
813557	0,0%	0,0%	0,0%	0,0%	0,0%	0,0%	0,0%	0,0%	0,0%	Bacteria	Verrucomicrobia	Opitutae	Opitutales	Opitutaceae	Opitutus	-	napA	-	nirA	-	-	-	-	nosZ	-	-	nrfA	ureA	-	-	-	-	-	-	-	-	laccase_Basidio	-	-	-
813823	0,0%	0,0%	0,0%	0,0%	0,0%	0,0%	0,0%	0,0%	0,0%	Bacteria	Proteobacteria	Alphaproteobacteria	Rhodobacterales	Rhodobacteraceae	Rhodobacter	-	napA	-	nirA	-	nirS	nirK	norB	nosZ	nifH	nifD	-	ureA	-	-	SoxB	-	-	mmoX	-	laccase_Asco	laccase_Basidio	-	-	ppo
4424907	0,0%	0,0%	0,0%	0,0%	0,0%	0,0%	0,0%	0,0%	0,0%	Bacteria	Proteobacteria	Alphaproteobacteria	Rhizobiales	Rhizobiaceae	Rhizobium	-	napA	narG	nirA	-	-	nirK	norB	nosZ	nifH	nifD	nrfA	ureA	-	-	-	-	chb	mmoX	-	laccase_Asco	laccase_Basidio	-	-	ppo
22594	0,0%	0,0%	0,0%	0,0%	0,0%	0,0%	0,0%	0,0%	0,0%	Bacteria	Proteobacteria	Deltaproteobacteria	Bdellovibrionales	Bacteriovoracaceae	Bacteriovorax	-	-	-	-	-	-	-	norB	-	-	-	-	-	-	-	-	-	-	-	-	-	-	-	-	-
494906	0,0%	0,0%	0,0%	0,0%	0,0%	0,0%	0,0%	0,0%	0,0%	Bacteria	Firmicutes	Clostridia	Clostridiales	[Tissierellaceae]	Peptoniphilus	-	napA	-	-	-	-	-	-	-	nifH	nifD	-	-	-	-	-	-	-	-	-	-	-	-	-	-
664476	0,0%	0,0%	0,0%	0,0%	0,0%	0,0%	0,0%	0,0%	0,1%	Bacteria	Proteobacteria	Alphaproteobacteria	Rhodospirillales	Acetobacteraceae	uncultured	-	-	-	-	-	-	-	-	-	-	-	-	-	-	-	-	-	-	-	-	-	-	-	lip	-
973295	0,0%	0,0%	0,0%	0,0%	0,0%	0,0%	0,0%	0,0%	0,0%	Bacteria	Actinobacteria	Actinobacteria	Actinomycetales		Acidothermus	-	napA	narG	-	-	-	nirK	-	-	-	-	-	-	-	-	-	-	-	-	-	-	-	-	-	-
721703	0,0%	0,0%	0,0%	0,0%	0,0%	0,0%	0,0%	0,0%	0,0%	Bacteria	Proteobacteria	Betaproteobacteria	Burkholderiales	Comamonadaceae	Leptothrix	-	napA	-	-	-	nirS	-	norB	nosZ	nifH	nifD	-	ureA	-	-	-	-	-	mmoX	-	-	-	-	-	-
3850831	0,0%	0,0%	0,0%	0,0%	0,0%	0,0%	0,0%	0,0%	0,1%	Bacteria	Planctomycetes	Planctomycetia	Pirellulales	Pirellulaceae	Pir4 lineage	-	-	-	-	-	-	-	-	-	-	-	-	-	-	-	-	-	-	-	-	-	-	-	-	-
530275	0,0%	0,0%	0,0%	0,0%	0,0%	0,0%	0,0%	0,0%	0,0%	Bacteria	Proteobacteria	Betaproteobacteria	Burkholderiales	Comamonadaceae	uncultured	-	-	-	-	-	-	-	-	-	-	-	-	-	-	-	-	-	-	-	-	-	-	-	lip	-
4475886	0,0%	0,0%	0,0%	0,0%	0,0%	0,0%	0,0%	0,0%	0,0%	Bacteria	Proteobacteria	Betaproteobacteria	Burkholderiales	Oxalobacteraceae	Massilia	-	napA	narG	-	-	-	-	norB	nosZ	-	-	-	ureA	-	-	-	-	chb	mmoX	-	laccase_Asco	laccase_Basidio	-	-	ppo
4334437	0,0%	0,0%	0,0%	0,0%	0,0%	0,0%	0,0%	0,0%	0,0%	Bacteria	Proteobacteria	Betaproteobacteria	Rhodocyclales	Rhodocyclaceae	Denitratisoma	-	-	-	-	-	-	-	-	-	-	-	-	-	-	-	-	-	-	-	-	-	-	-	-	-
4456735	0,0%	0,0%	0,0%	0,0%	0,0%	0,0%	0,0%	0,0%	0,0%	Bacteria	Proteobacteria	Betaproteobacteria	Rhodocyclales	Rhodocyclaceae	Propionivibrio	-	-	-	-	-	-	-	-	-	-	-	-	-	-	-	-	-	-	-	-	-	-	-	-	-
3120500	0,0%	0,0%	0,0%	0,0%	0,0%	0,0%	0,0%	0,0%	0,0%	Bacteria	Proteobacteria	Gammaproteobacteria	Xanthomonadales	Sinobacteraceae	Nevskia	-	-	-	-	-	-	-	-	-	-	-	-	-	-	-	-	-	-	-	-	-	-	-	-	-
583912	0,0%	0,0%	0,0%	0,0%	0,0%	0,0%	0,0%	0,0%	0,0%	Bacteria	Proteobacteria	Alphaproteobacteria			uncultured	-	-	-	-	-	-	-	-	-	-	-	-	-	-	-	-	-	-	-	-	-	-	-	lip	-
831098	0,0%	0,0%	0,0%	0,0%	0,0%	0,0%	0,0%	0,0%	0,0%	Bacteria	Proteobacteria	Alphaproteobacteria	Rhodobacterales	Rhodobacteraceae	Falsirhodobacter	-	-	-	-	-	-	-	-	-	-	-	-	-	-	-	-	-	-	-	-	-	-	-	-	-
13568	0,0%	0,0%	0,0%	0,0%	0,0%	0,0%	0,0%	0,0%	0,0%	Bacteria	Actinobacteria	Actinobacteria	Actinomycetales	Nocardiaceae	Rhodococcus	-	napA	narG	nirA	-	-	nirK	norB	-	-	-	-	ureA	-	-	-	-	-	mmoX	-	laccase_Asco	laccase_Basidio	-	-	ppo
4403854	0,0%	0,0%	0,0%	0,0%	0,0%	0,0%	0,0%	0,0%	0,0%	Bacteria	Firmicutes	Clostridia	Clostridiales	Veillonellaceae	Veillonella	-	napA	narG	-	-	-	-	norB	-	-	nifD	-	-	-	-	-	-	-	-	-	laccase_Asco	laccase_Basidio	-	-	ppo
152978	0,0%	0,0%	0,0%	0,0%	0,0%	0,0%	0,0%	0,0%	0,0%	Bacteria	Proteobacteria	Alphaproteobacteria	Rhizobiales	Bradyrhizobiaceae	Nitrobacter	-	napA	narG	-	nxrB	-	nirK	-	-	-	-	-	-	-	-	SoxB	-	-	-	-	laccase_Asco	laccase_Basidio	-	-	ppo
231088	0,0%	0,0%	0,0%	0,0%	0,0%	0,0%	0,0%	0,0%	0,0%	Bacteria	Proteobacteria	Alphaproteobacteria	Sphingomonadales	Sphingomonadaceae	Sphingopyxis	-	napA	-	-	-	-	-	norB	-	-	-	-	-	-	-	-	-	chb	-	-	laccase_Asco	laccase_Basidio	-	-	ppo
786420	0,0%	0,0%	0,0%	0,0%	0,0%	0,0%	0,0%	0,0%	0,1%	Bacteria	Verrucomicrobia	[Spartobacteria]	[Chthoniobacterales]	[Chthoniobacteraceae]	uncultured bacterium	-	-	-	-	-	-	-	-	-	-	-	-	-	-	-	-	-	-	-	-	-	-	-	-	-
657718	0,0%	0,0%	0,0%	0,0%	0,0%	0,0%	0,0%	0,0%	0,0%	Bacteria	Proteobacteria	Alphaproteobacteria	Rhizobiales	Hyphomicrobiaceae	uncultured	-	-	-	-	-	-	-	-	-	-	-	-	-	-	-	-	-	-	-	-	-	-	-	lip	-
4422568	0,0%	0,0%	0,0%	0,0%	0,0%	0,0%	0,0%	0,0%	0,0%	Bacteria	Proteobacteria	Alphaproteobacteria	Rhodobacterales	Rhodobacteraceae	Rhodobacter	-	napA	-	nirA	-	nirS	nirK	norB	nosZ	nifH	nifD	-	ureA	-	-	SoxB	-	-	mmoX	-	laccase_Asco	laccase_Basidio	-	-	ppo
4383980	0,0%	0,0%	0,0%	0,0%	0,0%	0,0%	0,0%	0,0%	0,0%	Bacteria	Proteobacteria	Alphaproteobacteria	BD7-3		uncultured bacterium	-	-	-	-	-	-	-	-	-	-	-	-	-	-	-	-	-	-	-	-	-	-	-	-	-
349981	0,0%	0,0%	0,0%	0,0%	0,0%	0,0%	0,0%	0,0%	0,0%	Bacteria	Proteobacteria	Gammaproteobacteria	Pseudomonadales	Pseudomonadaceae	Pseudomonas	cellulolytic	napA	narG	nirA	-	nirS	nirK	norB	nosZ	nifH	nifD	-	ureA	-	dsrB	SoxB	-	-	mmoX	-	laccase_Asco	laccase_Basidio	-	-	ppo
337426	0,0%	0,0%	0,0%	0,0%	0,0%	0,0%	0,0%	0,0%	0,0%	Bacteria	Verrucomicrobia	[Pedosphaerae]	[Pedosphaerales]	R4-41B	uncultured bacterium	-	-	-	-	-	-	-	-	-	-	-	-	-	-	-	-	-	-	-	-	-	-	-	-	-
4475319	0,0%	0,0%	0,0%	0,0%	0,0%	0,0%	0,0%	0,0%	0,0%	Bacteria	Verrucomicrobia	[Pedosphaerae]	[Pedosphaerales]	[Pedosphaeraceae]	Pedosphaera	-	napA	-	-	-	-	nirK	-	-	-	-	-	-	-	-	-	-	-	-	-	-	-	-	-	-
1131545	0,0%	0,0%	0,0%	0,0%	0,0%	0,0%	0,0%	0,0%	0,0%	Bacteria	Proteobacteria	Deltaproteobacteria	Myxococcales		Sorangium	-	napA	-	-	-	-	nirK	norB	-	-	-	nrfA	ureA	-	-	-	-	chb	-	-	laccase_Asco	laccase_Basidio	-	-	ppo
153174	0,0%	0,0%	0,0%	0,0%	0,0%	0,0%	0,0%	0,0%	0,0%	Bacteria	Verrucomicrobia	Opitutae	Opitutales	Opitutaceae	Opitutus	-	napA	-	nirA	-	-	-	-	nosZ	-	-	nrfA	ureA	-	-	-	-	-	-	-	-	laccase_Basidio	-	-	-
705559	0,0%	0,0%	0,0%	0,0%	0,0%	0,0%	0,0%	0,0%	0,0%	Bacteria	Proteobacteria	Betaproteobacteria	Rhodocyclales	Rhodocyclaceae	Denitratisoma	-	-	-	-	-	-	-	-	-	-	-	-	-	-	-	-	-	-	-	-	-	-	-	-	-
1106653	0,0%	0,0%	0,0%	0,0%	0,0%	0,0%	0,0%	0,0%	0,0%	Bacteria	Proteobacteria	Gammaproteobacteria	Xanthomonadales	Sinobacteraceae	uncultured bacterium	-	-	-	-	-	-	-	-	-	-	-	-	-	-	-	-	-	-	-	-	-	-	-	-	-
4468812	0,0%	0,0%	0,0%	0,0%	0,0%	0,0%	0,0%	0,0%	0,0%	Bacteria	Proteobacteria	Betaproteobacteria	Burkholderiales	Comamonadaceae	Chlorochromatium	-	-	-	-	-	-	-	-	-	-	-	-	-	-	-	-	-	-	-	-	-	-	-	-	-
70614	0,0%	0,0%	0,0%	0,0%	0,0%	0,0%	0,0%	0,0%	0,0%	Bacteria	Acidobacteria	DA052	Ellin6513		uncultured eubacterium WD2123	-	-	-	-	-	-	-	-	-	-	-	-	-	-	-	-	-	-	-	-	-	-	-	-	-
592129	0,0%	0,0%	0,0%	0,0%	0,0%	0,0%	0,0%	0,0%	0,0%	Bacteria	Verrucomicrobia	Opitutae	Opitutales	Opitutaceae	Opitutus	-	napA	-	nirA	-	-	-	-	nosZ	-	-	nrfA	ureA	-	-	-	-	-	-	-	-	laccase_Basidio	-	-	-
60591	0,0%	0,0%	0,0%	0,0%	0,0%	0,0%	0,0%	0,0%	0,0%	Bacteria	Elusimicrobia	Elusimicrobia	Elusimicrobiales		uncultured bacterium	-	-	-	-	-	-	-	-	-	-	-	-	-	-	-	-	-	-	-	-	-	-	-	-	-

219369	0,0%	0,0%	0,0%	0,0%	0,0%	0,0%	0,0%	0,0%	0,0%	Bacteria	Proteobacteria	Deltaproteobacteria	Myxococcales		Sorangium	-	napA	-	-	-	-	nirK	norB	-	-	-	nrfA	ureA	-	-	-	-	chb	-	-	laccase_Asco	laccase_Basidio	-	-	ppo
3833244	0,0%	0,0%	0,0%	0,0%	0,0%	0,0%	0,0%	0,0%	0,0%	Bacteria	Proteobacteria	Betaproteobacteria	Rhodocyclales	Rhodocyclaceae	Propionivibrio	-	-	-	-	-	-	-	-	-	-	-	-	-	-	-	-	-	-	-	-	-	-	-	-	-
4418885	0,0%	0,0%	0,0%	0,0%	0,0%	0,0%	0,0%	0,0%	0,0%	Bacteria	Proteobacteria	Deltaproteobacteria	Bdellovibrionales	Bacteriovoracaceae	Peredibacter	-	-	-	-	-	-	-	-	-	-	-	-	-	-	-	-	-	-	-	-	-	-	-	-	-
1564851	0,0%	0,0%	0,0%	0,0%	0,0%	0,0%	0,0%	0,0%	0,0%	Bacteria	Proteobacteria	Gammaproteobacteria	Legionellales	Legionellaceae	Legionella	-	-	-	-	-	nirS	-	norB	-	-	-	-	-	-	-	-	-	-	-	-	laccase_Asco	laccase_Basidio	-	-	ppo
4483892	0,0%	0,0%	0,0%	0,0%	0,0%	0,0%	0,0%	0,0%	0,0%	Bacteria	Firmicutes	Clostridia	Clostridiales	Peptococcaceae	Desulfosporosinus	-	napA	narG	-	-	-	nirK	-	nosZ	nifH	nifD	nrfA	ureA	dsrA	dsrB	-	-	-	-	-	-	-	-	-	-
512148	0,0%	0,0%	0,0%	0,0%	0,0%	0,0%	0,0%	0,0%	0,1%	Bacteria	Actinobacteria	Thermoleophilia	Solirubrobacterales		uncultured bacterium	-	-	-	-	-	-	-	-	-	-	-	-	-	-	-	-	-	-	-	-	-	-	-	-	-
514112	0,0%	0,0%	0,0%	0,0%	0,0%	0,0%	0,0%	0,0%	0,0%	Bacteria	Proteobacteria	Gammaproteobacteria			uncultured	-	-	-	-	-	-	-	-	-	-	-	-	-	-	-	-	-	-	-	-	-	-	-	lip	-
99390	0,0%	0,0%	0,0%	0,0%	0,0%	0,0%	0,0%	0,0%	0,1%	Bacteria	Proteobacteria	Gammaproteobacteria	Legionellales	Coxiellaceae	Aquicella	-	-	-	-	-	-	-	-	-	-	-	-	-	-	-	-	-	-	-	-	-	-	-	-	-
2010530	0,0%	0,0%	0,0%	0,0%	0,0%	0,0%	0,0%	0,0%	0,0%	Bacteria	Proteobacteria	Alphaproteobacteria	Rhodospirillales	Rhodospirillaceae	uncultured	-	-	-	-	-	-	-	-	-	-	-	-	-	-	-	-	-	-	-	-	-	-	-	lip	-
252876	0,0%	0,0%	0,0%	0,0%	0,0%	0,0%	0,0%	0,0%	0,0%	Bacteria	Proteobacteria	Betaproteobacteria	Burkholderiales	Comamonadaceae	uncultured	-	-	-	-	-	-	-	-	-	-	-	-	-	-	-	-	-	-	-	-	-	-	-	lip	-
211089	0,0%	0,0%	0,0%	0,0%	0,0%	0,0%	0,0%	0,0%	0,0%	Bacteria	Bacteroidetes	Cytophagia	Cytophagales	Cytophagaceae	Cytophaga	cellulolytic	napA	-	-	-	-	-	norB	-	-	nifD	-	ureA	-	-	-	-	-	-	-	-	-	-	-	-
4389775	0,0%	0,0%	0,0%	0,0%	0,0%	0,0%	0,0%	0,0%	0,0%	Bacteria	Proteobacteria	Gammaproteobacteria	Legionellales	Coxiellaceae	Aquicella	-	-	-	-	-	-	-	-	-	-	-	-	-	-	-	-	-	-	-	-	-	-	-	-	-
4370059	0,0%	0,0%	0,0%	0,0%	0,0%	0,0%	0,0%	0,0%	0,0%	Bacteria	Proteobacteria	Alphaproteobacteria	Sphingomonadales	Sphingomonadaceae	Sphingopyxis	-	napA	-	-	-	-	-	norB	-	-	-	-	-	-	-	-	-	chb	-	-	laccase_Asco	laccase_Basidio	-	-	ppo
278226	0,0%	0,0%	0,0%	0,0%	0,0%	0,0%	0,0%	0,0%	0,0%	Bacteria	Proteobacteria	Alphaproteobacteria	Caulobacterales	Caulobacteraceae	Phenylobacterium	-	napA	narG	-	-	-	nirK	norB	-	-	-	-	-	-	-	-	-	-	-	-	laccase_Asco	laccase_Basidio	-	-	ppo
164431	0,0%	0,0%	0,0%	0,0%	0,0%	0,0%	0,0%	0,0%	0,1%	Bacteria	Fusobacteria	Fusobacteriia	Fusobacteriales	Fusobacteriaceae	Propionigenium	-	-	-	-	-	-	-	-	-	-	-	-	-	-	-	-	-	-	-	-	-	-	-	-	-
3533825	0,0%	0,0%	0,0%	0,0%	0,0%	0,0%	0,0%	0,0%	0,0%	Bacteria	Proteobacteria	Alphaproteobacteria	BD7-3		uncultured bacterium	-	-	-	-	-	-	-	-	-	-	-	-	-	-	-	-	-	-	-	-	-	-	-	-	-
1106817	0,0%	0,0%	0,0%	0,0%	0,0%	0,0%	0,0%	0,0%	0,0%	Bacteria	Verrucomicrobia	[Spartobacteria]	[Chthoniobacterales]	[Chthoniobacteraceae]	uncultured bacterium	-	-	-	-	-	-	-	-	-	-	-	-	-	-	-	-	-	-	-	-	-	-	-	-	-
509929	0,0%	0,0%	0,0%	0,0%	0,0%	0,0%	0,0%	0,0%	0,0%	Bacteria	Proteobacteria	Gammaproteobacteria	Methylococcales	Crenotrichaceae	Crenothrix	-	-	-	-	-	-	-	-	-	-	-	-	-	-	-	-	-	-	-	pmoA	-	-	-	-	-
824783	0,0%	0,0%	0,0%	0,0%	0,0%	0,0%	0,0%	0,0%	0,0%	Bacteria	Proteobacteria	Deltaproteobacteria	Myxococcales		Sorangium	-	napA	-	-	-	-	nirK	norB	-	-	-	nrfA	ureA	-	-	-	-	chb	-	-	laccase_Asco	laccase_Basidio	-	-	ppo
4349094	0,0%	0,0%	0,0%	0,0%	0,0%	0,0%	0,0%	0,0%	0,0%	Bacteria	Proteobacteria	Gammaproteobacteria	Xanthomonadales	Sinobacteraceae	uncultured bacterium	-	-	-	-	-	-	-	-	-	-	-	-	-	-	-	-	-	-	-	-	-	-	-	-	-
4304357	0,0%	0,0%	0,0%	0,0%	0,0%	0,0%	0,0%	0,0%	0,0%	Bacteria	Proteobacteria	Alphaproteobacteria	Rhizobiales	Phyllobacteriaceae	Nitratireductor	-	napA	-	nirA	-	-	nirK	-	nosZ	nifH	-	-	ureA	-	-	-	-	-	-	-	laccase_Asco	laccase_Basidio	-	-	ppo
3509162	0,0%	0,0%	0,0%	0,0%	0,0%	0,0%	0,0%	0,0%	0,0%	Bacteria	Bacteroidetes	[Saprospirae]	[Saprospirales]	Chitinophagaceae	Lacibacter	-	-	-	-	-	-	-	-	-	-	-	-	-	-	-	-	-	-	-	-	-	-	-	-	-
4347269	0,0%	0,0%	0,0%	0,0%	0,0%	0,0%	0,0%	0,0%	0,0%	Bacteria	Verrucomicrobia	[Spartobacteria]	[Chthoniobacterales]	[Chthoniobacteraceae]	uncultured bacterium	-	-	-	-	-	-	-	-	-	-	-	-	-	-	-	-	-	-	-	-	-	-	-	-	-
4304697	0,0%	0,0%	0,0%	0,0%	0,0%	0,0%	0,0%	0,0%	0,0%	Bacteria	Proteobacteria	Betaproteobacteria	Burkholderiales	Comamonadaceae	uncultured	-	-	-	-	-	-	-	-	-	-	-	-	-	-	-	-	-	-	-	-	-	-	-	lip	-
203364	0,0%	0,0%	0,0%	0,0%	0,0%	0,0%	0,0%	0,0%	0,0%	Bacteria	Proteobacteria	Gammaproteobacteria	Aeromonadales	Aeromonadaceae	Aeromonas	-	napA	narG	-	-	-	-	-	nosZ	nifH	-	nrfA	ureA	-	dsrB	-	-	chb	-	-	laccase_Asco	laccase_Basidio	-	-	ppo
809307	0,0%	0,0%	0,0%	0,0%	0,0%	0,0%	0,0%	0,0%	0,0%	Bacteria	Proteobacteria	Betaproteobacteria	Burkholderiales	Comamonadaceae	Leptothrix	-	napA	-	-	-	nirS	-	norB	nosZ	nifH	nifD	-	ureA	-	-	-	-	-	mmoX	-	-	-	-	-	-
3582212	0,0%	0,0%	0,0%	0,0%	0,0%	0,0%	0,0%	0,0%	0,0%	Bacteria	Verrucomicrobia	Verrucomicrobiae	Verrucomicrobiales	Verrucomicrobiaceae	Verrucomicrobium	-	-	-	-	-	-	-	-	-	-	-	-	-	-	-	-	-	-	-	-	laccase_Asco	laccase_Basidio	-	-	ppo
826250	0,0%	0,0%	0,0%	0,0%	0,0%	0,0%	0,0%	0,0%	0,0%	Bacteria	Firmicutes	Bacilli	Bacillales	Bacillaceae	Incertae Sedis	cellulolytic	-	-	-	-	-	-	-	-	-	-	-	-	-	-	-	-	-	-	-	-	-	-	-	-
155778	0,0%	0,0%	0,0%	0,0%	0,0%	0,0%	0,0%	0,0%	0,1%	Bacteria	Verrucomicrobia	[Pedosphaerae]	[Pedosphaerales]		uncultured bacterium	-	-	-	-	-	-	-	-	-	-	-	-	-	-	-	-	-	-	-	-	-	-	-	-	-
1131767	0,0%	0,0%	0,0%	0,0%	0,0%	0,0%	0,0%	0,0%	0,0%	Bacteria	Verrucomicrobia	Verrucomicrobiae	Verrucomicrobiales	Verrucomicrobiaceae	uncultured	-	-	-	-	-	-	-	-	-	-	-	-	-	-	-	-	-	-	-	-	-	-	-	lip	-
53533	0,0%	0,0%	0,0%	0,0%	0,0%	0,0%	0,0%	0,0%	0,0%	Bacteria	Proteobacteria	Alphaproteobacteria	Rhizobiales		Methylorosula	-	-	-	-	-	-	-	-	-	-	-	-	-	-	-	-	-	-	-	-	-	-	-	-	-
912148	0,0%	0,0%	0,0%	0,0%	0,0%	0,0%	0,0%	0,0%	0,0%	Bacteria	Proteobacteria	Betaproteobacteria	Burkholderiales	Comamonadaceae	Chlorochromatium	-	-	-	-	-	-	-	-	-	-	-	-	-	-	-	-	-	-	-	-	-	-	-	-	-
791926	0,0%	0,0%	0,0%	0,0%	0,0%	0,0%	0,0%	0,0%	0,0%	Bacteria	Proteobacteria	Deltaproteobacteria	MIZ46		uncultured bacterium	-	-	-	-	-	-	-	-	-	-	-	-	-	-	-	-	-	-	-	-	-	-	-	-	-
640341	0,0%	0,0%	0,0%	0,0%	0,0%	0,0%	0,0%	0,0%	0,0%	Bacteria	Proteobacteria	Deltaproteobacteria	Myxococcales	OM27	OM27 clade	-	-	-	-	-	-	-	-	-	-	-	-	-	-	-	-	-	-	-	-	-	-	-	-	-
1043812	0,0%	0,0%	0,0%	0,0%	0,0%	0,0%	0,0%	0,0%	0,0%	Bacteria	Actinobacteria	Actinobacteria	Actinomycetales		Acidothermus	-	napA	narG	-	-	-	nirK	-	-	-	-	-	-	-	-	-	-	-	-	-	-	-	-	-	-
4372979	0,0%	0,0%	0,0%	0,0%	0,0%	0,0%	0,0%	0,0%	0,0%	Bacteria	Nitrospirae	Nitrospira	Nitrospirales	FW	uncultured bacterium	-	-	-	-	-	-	-	-	-	-	-	-	-	-	-	-	-	-	-	-	-	-	-	-	-
588867	0,0%	0,0%	0,0%	0,0%	0,0%	0,0%	0,0%	0,0%	0,0%	Bacteria	Bacteroidetes	Cytophagia	Cytophagales	Cytophagaceae	Fibrella	cellulolytic	napA	-	-	-	-	-	-	-	-	-	-	-	-	-	-	-	chb	-	-	laccase_Asco	laccase_Basidio	-	-	ppo
13034	0,0%	0,0%	0,0%	0,0%	0,0%	0,0%	0,0%	0,0%	0,0%	Bacteria	Actinobacteria	Actinobacteria	Actinomycetales	Mycobacteriaceae	Mycobacterium	-	napA	narG	nirA	-	-	-	norB	-	-	-	-	ureA	-	-	-	amoA_AOB	-	mmoX	pmoA	laccase_Asco	laccase_Basidio	-	-	ppo
528336	0,0%	0,0%	0,0%	0,0%	0,0%	0,0%	0,0%	0,0%	0,0%	Bacteria	Bacteroidetes	[Saprospirae]	[Saprospirales]	Chitinophagaceae	Ferruginibacter	-	-	-	-	-	-	-	-	-	-	-	-	-	-	-	-	-	-	-	-	-	-	-	-	-
4481011	0,0%	0,0%	0,0%	0,0%	0,0%	0,0%	0,0%	0,0%	0,0%	Bacteria	Actinobacteria	Actinobacteria	Actinomycetales	Sporichthyaceae	uncultured Pseudonocardiaceae bacterium	-	-	-	-	-	-	-	-	-	-	-	-	-	-	-	-	-	-	-	-	-	-	-	-	-
567840	0,0%	0,0%	0,0%	0,0%	0,0%	0,0%	0,0%	0,0%	0,0%	Bacteria	Proteobacteria	Alphaproteobacteria	Rhizobiales	Bradyrhizobiaceae	Nitrobacter	-	napA	narG	-	nxrB	-	nirK	-	-	-	-	-	-	-	-	SoxB	-	-	-	-	laccase_Asco	laccase_Basidio	-	-	ppo
4310868	0,0%	0,0%	0,0%	0,0%	0,0%	0,0%	0,0%	0,0%	0,0%	Bacteria	Proteobacteria	Alphaproteobacteria	Sphingomonadales	Sphingomonadaceae	Altererythrobacter	-	napA	narG	-	-	-	-	-	-	-	-	-	-	-	-	-	-	-	-	-	laccase_Asco	laccase_Basidio	-	-	ppo
2195967	0,0%	0,0%	0,0%	0,0%	0,0%	0,0%	0,0%	0,0%	0,0%	Bacteria	Verrucomicrobia	[Pedosphaerae]	[Pedosphaerales]	auto67_4W	uncultured bacterium	-	-	-	-	-	-	-	-	-	-	-	-	-	-	-	-	-	-	-	-	-	-	-	-	-
810595	0,0%	0,0%	0,0%	0,0%	0,0%	0,0%	0,0%	0,0%	0,1%	Bacteria	Proteobacteria	Deltaproteobacteria	Syntrophobacterales	Syntrophobacteraceae	uncultured bacterium	-	-	-	-	-	-	-	-	-	-	-	-	-	-	-	-	-	-	-	-	-	-	-	-	-
4428924	0,0%	0,0%	0,0%	0,0%	0,0%	0,0%	0,0%	0,0%	0,0%	Bacteria	Proteobacteria	Betaproteobacteria	Methylophilales	Methylophilaceae	Methylophilus	-	napA	-	-	-	nirS	-	norB	-	-	-	-	ureA	-	-	-	-	-	-	-	-	-	-	-	-
4468234	0,0%	0,0%	0,0%	0,0%	0,0%	0,0%	0,0%	0,0%	0,0%	Bacteria	Bacteroidetes	Bacteroidia	Bacteroidales	Bacteroidaceae	Bacteroides	cellulolytic	-	narG	-	-	-	-	-	-	-	nifD	nrfA	-	-	dsrB	-	-	chb	-	-	-	-	-	-	-
138840	0,0%	0,0%	0,0%	0,0%	0,0%	0,0%	0,0%	0,0%	0,0%	Bacteria	Proteobacteria	Gammaproteobacteria	Pseudomonadales	Pseudomonadaceae	Pseudomonas	cellulolytic	napA	narG	nirA	-	nirS	nirK	norB	nosZ	nifH	nifD	-	ureA	-	dsrB	SoxB	-	-	mmoX	-	laccase_Asco	laccase_Basidio	-	-	ppo
789797	0,0%	0,0%	0,0%	0,0%	0,0%	0,0%	0,0%	0,0%	0,0%	Bacteria	Acidobacteria	Holophagae	Holophagales	Holophagaceae	Geothrix	-	-	-	-	-	-	-	-	-	-	-	-	-	-	-	-	-	-	-	-	-	-	-	-	-
2806	0,0%	0,0%	0,0%	0,0%	0,0%	0,0%	0,0%	0,0%	0,0%	Bacteria	Planctomycetes	Planctomycetia	Pirellulales	Pirellulaceae	Pirellula	-	napA	-	-	-	-	-	norB	-	-	-	-	-	-	-	-	-	-	-	-	-	-	-	-	-
334140	0,0%	0,0%	0,0%	0,0%	0,0%	0,0%	0,0%	0,0%	0,0%	Bacteria	Actinobacteria	Actinobacteria	Actinomycetales		Acidothermus	-	napA	narG	-	-	-	nirK	-	-	-	-	-	-	-	-	-	-	-	-	-	-	-	-	-	-
4434807	0,0%	0,0%	0,0%	0,0%	0,0%	0,0%	0,0%	0,0%	0,0%	Bacteria	Actinobacteria	Actinobacteria	Actinomycetales	Micromonosporaceae	Spirilliplanes	cellulolytic	-	-	-	-	-	-	-	-	-	-	-	-	-	-	-	-	-	-	-	-	-	-	-	-
769696	0,0%	0,0%	0,0%	0,0%	0,0%	0,0%	0,0%	0,0%	0,0%	Bacteria	Chloroflexi	Ellin6529			uncultured bacterium	-	-	-	-	-	-	-	-	-	-	-	-	-	-	-	-	-	-	-	-	-	-	-	-	-
164404	0,0%	0,0%	0,0%	0,0%	0,0%	0,0%	0,0%	0,0%	0,0%	Bacteria	Proteobacteria	Alphaproteobacteria	Rhodobacterales	Hyphomonadaceae	Hirschia	-	napA	-	-	-	-	-	-	-	-	-	-	ureA	-	-	-	-	-	-	-	laccase_Asco	laccase_Basidio	-	-	ppo
975760	0,0%	0,0%	0,0%	0,0%	0,0%	0,0%	0,0%	0,0%	0,0%	Bacteria	Planctomycetes	Planctomycetia	Gemmatales	Isosphaeraceae	Isosphaera	-	napA	-	-	-	-	-	-	-	-	-	-	-	-	-	-	-	-	-	-	-	-	-	-	-
573663	0,0%	0,0%	0,0%	0,0%	0,0%	0,0%	0,0%	0,0%	0,0%	Bacteria	Proteobacteria	Alphaproteobacteria	Rhodospirillales	Acetobacteraceae	Roseomonas	-	napA	-	-	-	-	-	-	-	-	nifD	-	ureA	-	-	-	-	-	-	-	-	laccase_Basidio	-	-	-
1143820	0,0%	0,0%	0,0%	0,0%	0,0%	0,0%	0,0%	0,0%	0,0%	Bacteria	Proteobacteria	Betaproteobacteria	Burkholderiales	Comamonadaceae	Rhizobacter	-	napA	narG	nirA	-	-	-	norB	-	-	-	-	ureA	-	-	-	-	-	-	-	-	-	-	-	-
655141	0,0%	0,0%	0,0%	0,0%	0,0%	0,0%	0,0%	0,0%	0,0%	Bacteria	Proteobacteria	Betaproteobacteria	SBla14		Candidatus Nitrotoga	-	-	-	-	-	-	-	-	-	-	-	-	-	-	-	-	-	-	-	-	-	-	-	-	-
1060958	0,0%	0,0%	0,0%	0,0%	0,0%	0,0%	0,0%	0,0%	0,0%	Bacteria	Proteobacteria	Alphaproteobacteria	Rhizobiales	Methylocystaceae	uncultured organism	-	-	-	-	-	-	-	-	-	-	-	-	-	-	-	-	-	-	-	-	-	-	-	-	-
136719	0,0%	0,0%	0,0%	0,0%	0,0%	0,0%	0,0%	0,0%	0,0%	Bacteria	Chlamydiae	Chlamydiia	Chlamydiales	Parachlamydiaceae	Candidatus Protochlamydia	-	-	-	-	-	-	-	-	-	-	-	-	-	-	-	-	-	-	-	-	-	-	-	-	-
4322206	0,0%	0,0%	0,0%	0,0%	0,0%	0,0%	0,0%	0,0%	0,0%	Bacteria	Planctomycetes	OM190	CL500-15		uncultured bacterium	-	-	-	-	-	-	-	-	-	-	-	-	-	-	-	-	-	-	-	-	-	-	-	-	-
4322207	0,0%	0,0%	0,0%	0,0%	0,0%	0,0%	0,0%	0,0%	0,0%	Bacteria	Proteobacteria	Alphaproteobacteria	Rhizobiales	Bradyrhizobiaceae	Bradyrhizobium	-	napA	narG	nirA	-	nirS	nirK	norB	nosZ	nifH	nifD	-	ureA	-	-	SoxB	-	-	mmoX	-	laccase_Asco	laccase_Basidio	-	-	ppo
4308189	0,0%	0,0%	0,0%	0,0%	0,0%	0,0%	0,0%	0,0%	0,0%	Bacteria	Proteobacteria	Betaproteobacteria	Burkholderiales	Comamonadaceae	uncultured	-	-	-	-	-	-	-	-	-	-	-	-	-	-	-	-	-	-	-	-	-	-	-	lip	-
4481986	0,0%	0,0%	0,0%	0,0%	0,0%	0,0%	0,0%	0,0%	0,0%	Bacteria	Acidobacteria	Solibacteres	Solibacterales	Solibacteraceae	Candidatus Solibacter	-	-	-	-	-	-	-	-	-	-	-	-	-	-	-	-	-	-	-	-	-	-	-	-	-
979344	0,0%	0,0%	0,0%	0,0%	0,0%	0,0%	0,0%	0,0%	0,0%	Bacteria	Proteobacteria	Alphaproteobacteria	Rhizobiales	Methylobacteriaceae	Methylobacterium	-	napA	narG	-	-	-	nirK	-	nosZ	nifH	nifD	-	ureA	-	-	-	-	-	-	-	laccase_Asco	laccase_Basidio	-	-	ppo
583293	0,0%	0,0%	0,0%	0,0%	0,0%	0,0%	0,0%	0,0%	0,0%	Bacteria	Actinobacteria	Thermoleophilia	Solirubrobacterales		uncultured actinobacterium	-	-	-	-	-	-	-	-	-	-	-	-	-	-	-	-	-	-	-	-	-	-	-	-	-
4418001	0,0%	0,0%	0,0%	0,0%	0,0%	0,0%	0,0%	0,0%	0,0%	Bacteria	Proteobacteria	Alphaproteobacteria	Rhizobiales	Hyphomicrobiaceae	uncultured	-	-	-	-	-	-	-	-	-	-	-	-	-	-	-	-	-	-	-	-	-	-	-	lip	-
245345	0,0%	0,0%	0,0%	0,0%	0,0%	0,0%	0,0%	0,0%	0,0%	Bacteria	Bacteroidetes	Sphingobacteriia	Sphingobacteriales		uncultured Bacteroidetes bacterium	-	-	-	-	-	-	-	-	-	-	-	-	-	-	-	-	-	-	-	-	-	-	-	-	-
4418009	0,0%	0,0%	0,0%	0,0%	0,0%	0,0%	0,0%	0,0%	0,0%	Bacteria	Proteobacteria	Betaproteobacteria	Burkholderiales	Oxalobacteraceae	Massilia	-	napA	narG	-	-	-	-	norB	nosZ	-	-	-	ureA	-	-	-	-	chb	mmoX	-	laccase_Asco	laccase_Basidio	-	-	ppo
4336253	0,0%	0,0%	0,0%	0,0%	0,0%	0,0%	0,0%	0,0%	0,0%	Bacteria	Actinobacteria	Actinobacteria	Actinomycetales	Mycobacteriaceae	Mycobacterium	-	napA	narG	nirA	-	-	-	norB	-	-	-	-	ureA	-	-	-	amoA_AOB	-	mmoX	pmoA	laccase_Asco	laccase_Basidio	-	-	ppo
4462802	0,0%	0,0%	0,0%	0,0%	0,0%	0,0%	0,0%	0,0%	0,0%	Bacteria	Proteobacteria	Betaproteobacteria	Burkholderiales	Comamonadaceae	uncultured	-	-	-	-	-	-	-	-	-	-	-	-	-	-	-	-	-	-	-	-	-	-	-	lip	-
4462801	0,0%	0,0%	0,0%	0,0%	0,0%	0,0%	0,0%	0,0%	0,0%	Bacteria	Proteobacteria	Betaproteobacteria	Burkholderiales	Comamonadaceae	uncultured	-	-	-	-	-	-	-	-	-	-	-	-	-	-	-	-	-	-	-	-	-	-	-	lip	-
1116890	0,0%	0,0%	0,0%	0,0%	0,0%	0,0%	0,0%	0,0%	0,0%	Bacteria	Bacteroidetes	[Saprospirae]	[Saprospirales]	Chitinophagaceae	Ferruginibacter	-	-	-	-	-	-	-	-	-	-	-	-	-	-	-	-	-	-	-	-	-	-	-	-	-
658707	0,0%	0,0%	0,0%	0,0%	0,0%	0,0%	0,0%	0,0%	0,0%	Bacteria	Verrucomicrobia	[Pedosphaerae]	[Pedosphaerales]	auto67_4W	uncultured bacterium	-	-	-	-	-	-	-	-	-	-	-	-	-	-	-	-	-	-	-	-	-	-	-	-	-
4344815	0,0%	0,0%	0,0%	0,0%	0,0%	0,0%	0,0%	0,0%	0,0%	Bacteria	Proteobacteria	Gammaproteobacteria	Legionellales		uncultured soil bacterium	-	-	-	-	-	-	-	-	-	-	-	-	-	-	-	-	-	-	-	-	-	-	-	-	-
4411140	0,0%	0,0%	0,0%	0,0%	0,0%	0,0%	0,0%	0,0%	0,0%	Bacteria	Proteobacteria	Deltaproteobacteria	Myxococcales		uncultured bacterium	-	-	-	-	-	-	-	-	-	-	-	-	-	-	-	-	-	-	-	-	-	-	-	-	-
4343459	0,0%	0,0%	0,0%	0,0%	0,0%	0,0%	0,0%	0,0%	0,0%	Bacteria	Acidobacteria	Acidobacteriia	Acidobacteriales	Koribacteraceae	uncultured	-	-	-	-	-	-	-	-	-	-	-	-	-	-	-	-	-	-	-	-	-	-	-	lip	-
819396	0,0%	0,0%	0,0%	0,0%	0,0%	0,0%	0,0%	0,0%	0,1%	Bacteria	Proteobacteria	Deltaproteobacteria	Myxococcales	Cystobacterineae	uncultured bacterium	-	-	-	-	-	-	-	-	-	-	-	-	-	-	-	-	-	-	-	-	-	-	-	-	-
4305126	0,0%	0,0%	0,0%	0,0%	0,0%	0,0%	0,0%	0,0%	0,0%	Bacteria	Proteobacteria	Alphaproteobacteria	Rickettsiales		Candidatus Captivus	-	-	-	-	-	-	-	-	-	-	-	-	-	-	-	-	-	-	-	-	-	-	-	-	-
1113028	0,0%	0,0%	0,0%	0,0%	0,0%	0,0%	0,0%	0,0%	0,0%	Bacteria	Actinobacteria	Actinobacteria	Actinomycetales		Acidothermus	-	napA	narG	-	-	-	nirK	-	-	-	-	-	-	-	-	-	-	-	-	-	-	-	-	-	-
4367292	0,0%	0,0%	0,0%	0,0%	0,0%	0,0%	0,0%	0,0%	0,0%	Bacteria	Acidobacteria	Solibacteres	Solibacterales		Bryobacter	-	-	-	-	-	-	-	-	-	-	-	-	-	-	-	-	-	-	-	-	-	-	-	-	-
4418266	0,0%	0,0%	0,0%	0,0%	0,0%	0,0%	0,0%	0,0%	0,0%	Bacteria	Proteobacteria	Gammaproteobacteria	Pseudomonadales	Moraxellaceae	Perlucidibaca	-	-	-	-	-	-	-	-	-	-	-	-	-	-	-	-	-	-	-	-	-	-	-	-	-
940630	0,0%	0,0%	0,0%	0,0%	0,0%	0,0%	0,0%	0,0%	0,0%	Bacteria	Planctomycetes	Planctomycetia	Gemmatales	Isosphaeraceae	Isosphaera	-	napA	-	-	-	-	-	-	-	-	-	-	-	-	-	-	-	-	-	-	-	-	-	-	-
3944484	0,0%	0,0%	0,0%	0,0%	0,0%	0,0%	0,0%	0,0%	0,0%	Bacteria	Proteobacteria	Gammaproteobacteria	Enterobacteriales	Enterobacteriaceae	Yersinia	cellulolytic	napA	narG	-	-	-	-	-	-	-	-	nrfA	ureA	-	-	-	-	chb	-	-	laccase_Asco	laccase_Basidio	-	-	ppo
4438990	0,0%	0,0%	0,0%	0,0%	0,0%	0,0%	0,0%	0,0%	0,0%	Bacteria	Verrucomicrobia	Verrucomicrobiae	Verrucomicrobiales	Verrucomicrobiaceae	uncultured bacterium	-	-	-	-	-	-	-	-	-	-	-	-	-	-	-	-	-	-	-	-	-	-	-	-	-
417101	0,0%	0,0%	0,0%	0,0%	0,0%	0,0%	0,0%	0,0%	0,0%	Bacteria	Proteobacteria	Alphaproteobacteria	Ellin329		Rhizomicrobium	-	-	-	-	-	-	-	-	-	-	-	-	-	-	-	-	-	-	-	-	-	-	-	-	-
677165	0,0%	0,0%	0,0%	0,0%	0,0%	0,0%	0,0%	0,0%	0,0%	Bacteria	Proteobacteria	Alphaproteobacteria	Rhizobiales	Methylobacteriaceae	Methylobacterium	-	napA	narG	-	-	-	nirK	-	nosZ	nifH	nifD	-	ureA	-	-	-	-	-	-	-	laccase_Asco	laccase_Basidio	-	-	ppo
4318690	0,0%	0,0%	0,0%	0,0%	0,0%	0,0%	0,0%	0,0%	0,0%	Bacteria	Proteobacteria	Betaproteobacteria	Burkholderiales	Comamonadaceae	uncultured	-	-	-	-	-	-	-	-	-	-	-	-	-	-	-	-	-	-	-	-	-	-	-	lip	-
114599	0,0%	0,0%	0,0%	0,0%	0,0%	0,0%	0,0%	0,0%	0,0%	Bacteria	Firmicutes	Clostridia	Clostridiales	Clostridiaceae	Clostridium sensu stricto 5	cellulolytic	-	-	-	-	-	-	-	-	-	-	-	-	-	-	-	-	-	-	-	-	-	-	-	-
912160	0,0%	0,0%	0,0%	0,0%	0,0%	0,0%	0,0%	0,0%	0,0%	Bacteria	Proteobacteria	Deltaproteobacteria	Spirobacillales		uncultured bacterium	-	-	-	-	-	-	-	-	-	-	-	-	-	-	-	-	-	-	-	-	-	-	-	-	-
866836	0,0%	0,0%	0,0%	0,0%	0,0%	0,0%	0,0%	0,0%	0,0%	Bacteria	Bacteroidetes	[Saprospirae]	[Saprospirales]	Chitinophagaceae	uncultured	-	-	-	-	-	-	-	-	-	-	-	-	-	-	-	-	-	-	-	-	-	-	-	lip	-
1039594	0,0%	0,0%	0,0%	0,0%	0,0%	0,0%	0,0%	0,0%	0,0%	Bacteria	Proteobacteria	Betaproteobacteria	Burkholderiales	Comamonadaceae	Pelomonas	-	napA	narG	nirA	-	nirS	-	norB	nosZ	nifH	-	-	ureA	-	-	-	-	-	-	-	-	-	-	-	-
542690	0,0%	0,0%	0,0%	0,0%	0,0%	0,0%	0,0%	0,0%	0,0%	Bacteria	Proteobacteria	Gammaproteobacteria	Thiotrichales	Thiotrichaceae	Thiothrix	-	-	-	-	-	nirS	-	-	-	-	-	-	-	dsrA	dsrB	-	-	-	-	-	-	-	-	-	-
4408610	0,0%	0,0%	0,0%	0,0%	0,0%	0,0%	0,0%	0,0%	0,0%	Bacteria	Proteobacteria	Alphaproteobacteria	Rhodobacterales	Rhodobacteraceae	Sulfitobacter	-	napA	narG	-	-	-	-	-	nosZ	nifH	-	-	ureA	-	-	-	-	-	-	-	laccase_Asco	laccase_Basidio	-	-	ppo
4477254	0,0%	0,0%	0,0%	0,0%	0,0%	0,0%	0,0%	0,0%	0,0%	Bacteria	Proteobacteria	Alphaproteobacteria	Sphingomonadales	Sphingomonadaceae	Altererythrobacter	-	napA	narG	-	-	-	-	-	-	-	-	-	-	-	-	-	-	-	-	-	laccase_Asco	laccase_Basidio	-	-	ppo
212306	0,0%	0,0%	0,0%	0,0%	0,0%	0,0%	0,0%	0,0%	0,0%	Bacteria	Planctomycetes	Planctomycetia	Gemmatales	Gemmataceae	uncultured	-	-	-	-	-	-	-	-	-	-	-	-	-	-	-	-	-	-	-	-	-	-	-	lip	-
4480598	0,0%	0,0%	0,0%	0,0%	0,0%	0,0%	0,0%	0,0%	0,0%	Bacteria	Firmicutes	Bacilli	Bacillales	[Exiguobacteraceae]	Exiguobacterium	-	napA	-	-	-	-	-	-	nosZ	-	-	-	-	-	-	-	-	-	-	-	laccase_Asco	laccase_Basidio	-	-	ppo
228557	0,0%	0,0%	0,0%	0,0%	0,0%	0,0%	0,0%	0,0%	0,0%	Bacteria	Bacteroidetes	Bacteroidia	Bacteroidales		uncultured bacterium	-	-	-	-	-	-	-	-	-	-	-	-	-	-	-	-	-	-	-	-	-	-	-	-	-
4473935	0,0%	0,0%	0,0%	0,0%	0,0%	0,0%	0,0%	0,0%	0,0%	Bacteria	Proteobacteria	Betaproteobacteria	SBla14		uncultured	-	-	-	-	-	-	-	-	-	-	-	-	-	-	-	-	-	-	-	-	-	-	-	lip	-
4473936	0,0%	0,0%	0,0%	0,0%	0,0%	0,0%	0,0%	0,0%	0,0%	Bacteria	Proteobacteria	Gammaproteobacteria	Methylococcales	Methylococcaceae	uncultured bacterium	-	-	-	-	-	-	-	-	-	-	-	-	-	-	-	-	-	-	-	-	-	-	-	-	-
551756	0,0%	0,0%	0,0%	0,0%	0,0%	0,0%	0,0%	0,0%	0,0%	Bacteria	Proteobacteria	Alphaproteobacteria	Rhizobiales	Rhizobiaceae	Kaistia	-	-	-	-	-	-	-	-	-	-	-	-	-	-	-	-	-	-	-	-	-	-	-	-	-
182489	0,0%	0,0%	0,0%	0,0%	0,0%	0,0%	0,0%	0,0%	0,0%	Bacteria	Proteobacteria	Alphaproteobacteria	Rhizobiales		uncultured alpha proteobacterium	-	-	-	-	-	-	-	-	-	-	-	-	-	-	-	-	-	-	-	-	-	-	-	-	-
1113998	0,0%	0,0%	0,0%	0,0%	0,0%	0,0%	0,0%	0,0%	0,0%	Bacteria	Actinobacteria	Thermoleophilia	Gaiellales		uncultured soil bacterium	-	-	-	-	-	-	-	-	-	-	-	-	-	-	-	-	-	-	-	-	-	-	-	-	-
4432889	0,0%	0,0%	0,0%	0,0%	0,0%	0,0%	0,0%	0,0%	0,0%	Bacteria	Actinobacteria	Actinobacteria	Actinomycetales	Intrasporangiaceae	Janibacter	-	napA	narG	-	-	-	-	-	-	-	-	-	ureA	-	-	-	-	-	mmoX	-	-	laccase_Basidio	-	-	-
287179	0,0%	0,0%	0,0%	0,0%	0,0%	0,0%	0,0%	0,0%	0,0%	Bacteria	Proteobacteria	Betaproteobacteria	Burkholderiales	Comamonadaceae	Aquabacterium	-	-	-	-	-	-	-	-	-	-	-	-	-	-	-	-	-	-	-	-	-	-	-	-	-
843254	0,0%	0,0%	0,0%	0,0%	0,0%	0,0%	0,0%	0,0%	0,0%	Bacteria	Proteobacteria	Alphaproteobacteria	Rhizobiales	Methylocystaceae	Pleomorphomonas	-	-	-	-	-	-	-	-	-	nifH	-	-	-	-	-	-	-	-	-	-	-	-	-	-	-
783687	0,0%	0,0%	0,0%	0,0%	0,0%	0,0%	0,0%	0,0%	0,0%	Bacteria	Proteobacteria	Betaproteobacteria	Burkholderiales	Comamonadaceae	Rhizobacter	-	napA	narG	nirA	-	-	-	norB	-	-	-	-	ureA	-	-	-	-	-	-	-	-	-	-	-	-
831407	0,0%	0,0%	0,0%	0,0%	0,0%	0,0%	0,0%	0,0%	0,0%	Bacteria	Proteobacteria	Alphaproteobacteria	Rhodospirillales	Rhodospirillaceae	Reyranella	-	-	-	-	-	-	-	-	-	-	-	-	-	-	-	-	-	-	-	-	-	-	-	-	-
201597	0,0%	0,0%	0,0%	0,0%	0,0%	0,0%	0,0%	0,0%	0,0%	Bacteria	Planctomycetes	Planctomycetia	Gemmatales	Gemmataceae	uncultured	-	-	-	-	-	-	-	-	-	-	-	-	-	-	-	-	-	-	-	-	-	-	-	lip	-
4439836	0,0%	0,0%	0,0%	0,0%	0,0%	0,0%	0,0%	0,0%	0,0%	Bacteria	Proteobacteria	Alphaproteobacteria			uncultured alpha proteobacterium	-	-	-	-	-	-	-	-	-	-	-	-	-	-	-	-	-	-	-	-	-	-	-	-	-
254401	0,0%	0,0%	0,0%	0,0%	0,0%	0,0%	0,0%	0,0%	0,0%	Bacteria	Proteobacteria	Alphaproteobacteria	Caulobacterales	Caulobacteraceae	Phenylobacterium	-	napA	narG	-	-	-	nirK	norB	-	-	-	-	-	-	-	-	-	-	-	-	laccase_Asco	laccase_Basidio	-	-	ppo
161263	0,0%	0,0%	0,0%	0,0%	0,0%	0,0%	0,0%	0,0%	0,0%	Bacteria	Verrucomicrobia	[Pedosphaerae]	[Pedosphaerales]	auto67_4W	bacterium enrichment culture clone auto10_4W	-	-	-	-	-	-	-	-	-	-	-	-	-	-	-	-	-	-	-	-	-	-	-	-	-
4456104	0,0%	0,0%	0,0%	0,0%	0,0%	0,0%	0,0%	0,0%	0,0%	Bacteria	Proteobacteria	Betaproteobacteria	Burkholderiales	Comamonadaceae	Acidovorax	-	napA	narG	nirA	-	nirS	nirK	norB	nosZ	-	-	-	ureA	-	-	-	-	-	mmoX	-	-	laccase_Basidio	-	-	ppo
509440	0,0%	0,0%	0,0%	0,0%	0,0%	0,0%	0,0%	0,0%	0,0%	Bacteria	Proteobacteria	Deltaproteobacteria	MIZ46		uncultured Syntrophobacteraceae bacterium	-	-	-	-	-	-	-	-	-	-	-	-	-	-	-	-	-	-	-	-	-	-	-	-	-
4379780	0,0%	0,0%	0,0%	0,0%	0,0%	0,0%	0,0%	0,0%	0,0%	Bacteria	Proteobacteria	Deltaproteobacteria	Myxococcales		uncultured bacterium	-	-	-	-	-	-	-	-	-	-	-	-	-	-	-	-	-	-	-	-	-	-	-	-	-
4453762	0,0%	0,0%	0,0%	0,0%	0,0%	0,0%	0,0%	0,0%	0,0%	Bacteria	Bacteroidetes	Cytophagia	Cytophagales	Cytophagaceae	Runella	cellulolytic	napA	-	nirA	-	-	-	norB	nosZ	-	-	-	-	-	-	-	-	chb	-	-	laccase_Asco	laccase_Basidio	-	-	ppo
2939253	0,0%	0,0%	0,0%	0,0%	0,0%	0,0%	0,0%	0,0%	0,0%	Bacteria	Proteobacteria	Deltaproteobacteria	Myxococcales	Haliangiaceae	Haliangium	-	-	-	-	-	-	-	-	-	-	-	-	ureA	-	-	-	-	chb	-	-	-	-	-	-	-
277693	0,0%	0,0%	0,0%	0,0%	0,0%	0,0%	0,0%	0,0%	0,0%	Bacteria	Planctomycetes	Planctomycetia	Gemmatales	Gemmataceae	uncultured	-	-	-	-	-	-	-	-	-	-	-	-	-	-	-	-	-	-	-	-	-	-	-	lip	-
4375328	0,0%	0,0%	0,0%	0,0%	0,0%	0,0%	0,0%	0,0%	0,0%	Bacteria	Proteobacteria	Alphaproteobacteria	Caulobacterales	Caulobacteraceae	Phenylobacterium	-	napA	narG	-	-	-	nirK	norB	-	-	-	-	-	-	-	-	-	-	-	-	laccase_Asco	laccase_Basidio	-	-	ppo
3163059	0,0%	0,0%	0,0%	0,0%	0,0%	0,0%	0,0%	0,0%	0,0%	Bacteria	Acidobacteria	Acidobacteriia	Acidobacteriales	Koribacteraceae	uncultured Acidobacteria bacterium	-	-	-	-	-	-	-	-	-	-	-	-	-	-	-	-	-	-	-	-	-	-	-	-	-
2105446	0,0%	0,0%	0,0%	0,0%	0,0%	0,0%	0,0%	0,0%	0,0%	Bacteria	Proteobacteria	Betaproteobacteria	Procabacteriales	Procabacteriaceae	uncultured	-	-	-	-	-	-	-	-	-	-	-	-	-	-	-	-	-	-	-	-	-	-	-	lip	-
686752	0,0%	0,0%	0,0%	0,0%	0,0%	0,0%	0,0%	0,0%	0,0%	Bacteria	Actinobacteria	Actinobacteria	Actinomycetales	Microbacteriaceae	Kineosporia	cellulolytic	-	-	-	-	-	-	-	-	-	-	-	-	-	-	-	-	-	-	-	-	-	-	-	-
531285	0,0%	0,0%	0,0%	0,0%	0,0%	0,0%	0,0%	0,0%	0,0%	Bacteria	Planctomycetes	Planctomycetia	Pirellulales	Pirellulaceae	Pirellula	-	napA	-	-	-	-	-	norB	-	-	-	-	-	-	-	-	-	-	-	-	-	-	-	-	-
1140085	0,0%	0,0%	0,0%	0,0%	0,0%	0,0%	0,0%	0,0%	0,0%	Bacteria	Proteobacteria	Alphaproteobacteria			uncultured	-	-	-	-	-	-	-	-	-	-	-	-	-	-	-	-	-	-	-	-	-	-	-	lip	-
1142742	0,0%	0,0%	0,0%	0,0%	0,0%	0,0%	0,0%	0,0%	0,0%	Bacteria	Proteobacteria	Alphaproteobacteria	Sphingomonadales	Sphingomonadaceae	Novosphingobium	-	napA	-	-	-	-	-	norB	-	nifH	nifD	-	ureA	-	-	-	-	-	mmoX	-	laccase_Asco	laccase_Basidio	-	-	ppo
804798	0,0%	0,0%	0,0%	0,0%	0,0%	0,0%	0,0%	0,0%	0,0%	Bacteria	Proteobacteria	Gammaproteobacteria	Xanthomonadales	Sinobacteraceae	uncultured	-	-	-	-	-	-	-	-	-	-	-	-	-	-	-	-	-	-	-	-	-	-	-	lip	-
366851	0,0%	0,0%	0,0%	0,0%	0,0%	0,0%	0,0%	0,0%	0,1%	Bacteria	Proteobacteria	Gammaproteobacteria	Xanthomonadales	Sinobacteraceae	uncultured bacterium	-	-	-	-	-	-	-	-	-	-	-	-	-	-	-	-	-	-	-	-	-	-	-	-	-
257891	0,0%	0,0%	0,0%	0,0%	0,0%	0,0%	0,0%	0,0%	0,0%	Bacteria	Proteobacteria	Alphaproteobacteria			Candidatus Liberibacter	-	-	-	-	-	-	-	-	-	-	-	-	-	-	-	-	-	-	-	-	-	-	-	-	-
640833	0,0%	0,0%	0,0%	0,0%	0,0%	0,0%	0,0%	0,0%	0,0%	Bacteria	Proteobacteria	Gammaproteobacteria	Chromatiales	Halothiobacillaceae	Thiovirga	-	-	-	-	-	-	-	-	-	-	-	-	-	-	-	-	-	-	-	-	-	-	-	-	-
958654	0,0%	0,0%	0,0%	0,0%	0,0%	0,0%	0,0%	0,0%	0,0%	Bacteria	Planctomycetes	Planctomycetia	Gemmatales	Gemmataceae	uncultured	-	-	-	-	-	-	-	-	-	-	-	-	-	-	-	-	-	-	-	-	-	-	-	lip	-
99071	0,0%	0,0%	0,0%	0,0%	0,0%	0,0%	0,0%	0,0%	0,0%	Bacteria	Proteobacteria	Betaproteobacteria	Burkholderiales		Derxia	-	-	-	-	-	-	-	-	-	nifH	-	-	-	-	-	-	-	-	-	-	-	-	-	-	-
4234522	0,0%	0,0%	0,0%	0,0%	0,0%	0,0%	0,0%	0,0%	0,0%	Bacteria	Proteobacteria	Deltaproteobacteria	Myxococcales	Myxococcaceae	Anaeromyxobacter	-	napA	narG	nirA	-	nirS	-	norB	nosZ	nifH	nifD	nrfA	ureA	-	-	SoxB	-	-	-	-	-	laccase_Basidio	-	-	-
515709	0,0%	0,0%	0,0%	0,0%	0,0%	0,0%	0,0%	0,0%	0,0%	Bacteria	Proteobacteria	Alphaproteobacteria	Rhizobiales	Hyphomicrobiaceae	uncultured	-	-	-	-	-	-	-	-	-	-	-	-	-	-	-	-	-	-	-	-	-	-	-	lip	-
553713	0,0%	0,0%	0,0%	0,0%	0,0%	0,0%	0,0%	0,0%	0,0%	Bacteria	Verrucomicrobia	Opitutae	Opitutales	Opitutaceae	Opitutus	-	napA	-	nirA	-	-	-	-	nosZ	-	-	nrfA	ureA	-	-	-	-	-	-	-	-	laccase_Basidio	-	-	-
550247	0,0%	0,0%	0,0%	0,0%	0,0%	0,0%	0,0%	0,0%	0,0%	Bacteria	Acidobacteria	Acidobacteriia	Acidobacteriales	Koribacteraceae	uncultured	-	-	-	-	-	-	-	-	-	-	-	-	-	-	-	-	-	-	-	-	-	-	-	lip	-
566185	0,0%	0,0%	0,0%	0,0%	0,0%	0,0%	0,0%	0,0%	0,0%	Bacteria	Planctomycetes	Planctomycetia	Pirellulales	Pirellulaceae	Pirellula	-	napA	-	-	-	-	-	norB	-	-	-	-	-	-	-	-	-	-	-	-	-	-	-	-	-
4440396	0,0%	0,0%	0,0%	0,0%	0,0%	0,0%	0,0%	0,0%	0,0%	Bacteria	Acidobacteria	Acidobacteriia	Acidobacteriales	Acidobacteriaceae	Frigoribacterium	cellulolytic	napA	-	-	-	-	-	-	-	-	-	-	-	-	-	-	-	-	-	-	-	-	-	-	-
2690238	0,0%	0,0%	0,0%	0,0%	0,0%	0,0%	0,0%	0,0%	0,0%	Bacteria	Proteobacteria	Alphaproteobacteria	Rhodobacterales	Rhodobacteraceae	Defluviimonas	-	napA	narG	-	-	-	-	-	-	-	-	-	ureA	-	-	-	-	-	-	-	-	laccase_Basidio	-	-	ppo
736771	0,0%	0,0%	0,0%	0,0%	0,0%	0,0%	0,0%	0,0%	0,0%	Bacteria	Bacteroidetes	[Saprospirae]	[Saprospirales]	Chitinophagaceae	Ferruginibacter	-	-	-	-	-	-	-	-	-	-	-	-	-	-	-	-	-	-	-	-	-	-	-	-	-
4424806	0,0%	0,0%	0,0%	0,0%	0,0%	0,0%	0,0%	0,0%	0,0%	Bacteria	Planctomycetes	Planctomycetia	Gemmatales	Isosphaeraceae	uncultured	-	-	-	-	-	-	-	-	-	-	-	-	-	-	-	-	-	-	-	-	-	-	-	lip	-
817714	0,0%	0,0%	0,0%	0,0%	0,0%	0,0%	0,0%	0,0%	0,0%	Bacteria	Proteobacteria	Deltaproteobacteria	Myxococcales		Haliangium	-	-	-	-	-	-	-	-	-	-	-	-	ureA	-	-	-	-	chb	-	-	-	-	-	-	-
725727	0,0%	0,0%	0,0%	0,0%	0,0%	0,0%	0,0%	0,0%	0,0%	Bacteria	Bacteroidetes	Cytophagia	Cytophagales	Cytophagaceae	uncultured	cellulolytic	-	-	-	-	-	-	-	-	-	-	-	-	-	-	-	-	-	-	-	-	-	-	lip	-
4388981	0,0%	0,0%	0,0%	0,0%	0,0%	0,0%	0,0%	0,0%	0,0%	Bacteria	Proteobacteria	Alphaproteobacteria			uncultured bacterium	-	-	-	-	-	-	-	-	-	-	-	-	-	-	-	-	-	-	-	-	-	-	-	-	-
245832	0,0%	0,0%	0,0%	0,0%	0,0%	0,0%	0,0%	0,0%	0,0%	Bacteria	Planctomycetes	Planctomycetia	Pirellulales	Pirellulaceae	uncultured	-	-	-	-	-	-	-	-	-	-	-	-	-	-	-	-	-	-	-	-	-	-	-	lip	-
719335	0,0%	0,0%	0,0%	0,0%	0,0%	0,0%	0,0%	0,0%	0,0%	Bacteria	Actinobacteria	Actinobacteria	Actinomycetales	Streptomycetaceae	uncultured bacterium	cellulolytic	-	-	-	-	-	-	-	-	-	-	-	-	-	-	-	-	-	-	-	-	-	-	-	-
985179	0,0%	0,0%	0,0%	0,0%	0,0%	0,0%	0,0%	0,0%	0,0%	Bacteria	Proteobacteria	Alphaproteobacteria	Sphingomonadales	Erythrobacteraceae	Altererythrobacter	-	napA	narG	-	-	-	-	-	-	-	-	-	-	-	-	-	-	-	-	-	laccase_Asco	laccase_Basidio	-	-	ppo
101392	0,0%	0,0%	0,0%	0,0%	0,0%	0,0%	0,0%	0,0%	0,0%	Bacteria	Proteobacteria	Betaproteobacteria	Ellin6067		uncultured	-	-	-	-	-	-	-	-	-	-	-	-	-	-	-	-	-	-	-	-	-	-	-	lip	-
2662591	0,0%	0,0%	0,0%	0,0%	0,0%	0,0%	0,0%	0,0%	0,0%	Bacteria	Proteobacteria	Alphaproteobacteria	Sphingomonadales	Sphingomonadaceae	Altererythrobacter	-	napA	narG	-	-	-	-	-	-	-	-	-	-	-	-	-	-	-	-	-	laccase_Asco	laccase_Basidio	-	-	ppo
581747	0,0%	0,0%	0,0%	0,0%	0,0%	0,0%	0,0%	0,0%	0,0%	Bacteria	Proteobacteria	Alphaproteobacteria	Rhodospirillales	Acetobacteraceae	uncultured bacterium	-	-	-	-	-	-	-	-	-	-	-	-	-	-	-	-	-	-	-	-	-	-	-	-	-
311942	0,0%	0,0%	0,0%	0,0%	0,0%	0,0%	0,0%	0,0%	0,0%	Bacteria	Proteobacteria	Gammaproteobacteria	Legionellales	Legionellaceae	Legionella	-	-	-	-	-	nirS	-	norB	-	-	-	-	-	-	-	-	-	-	-	-	laccase_Asco	laccase_Basidio	-	-	ppo
836238	0,0%	0,0%	0,0%	0,0%	0,0%	0,0%	0,0%	0,0%	0,1%	Bacteria	Acidobacteria	Acidobacteriia	Acidobacteriales	Acidobacteriaceae	Granulicella	cellulolytic	napA	-	-	-	-	-	-	-	-	-	-	-	-	-	-	-	-	-	-	laccase_Asco	laccase_Basidio	-	-	ppo
2333137	0,0%	0,0%	0,0%	0,0%	0,0%	0,0%	0,0%	0,0%	0,0%	Bacteria	Firmicutes	Bacilli	Bacillales	Paenibacillaceae	Paenibacillus	-	napA	narG	-	-	nirS	nirK	norB	nosZ	nifH	nifD	-	ureA	-	-	-	-	-	-	-	laccase_Asco	laccase_Basidio	-	-	ppo
723100	0,0%	0,0%	0,0%	0,0%	0,0%	0,0%	0,0%	0,0%	0,0%	Bacteria	Proteobacteria	Alphaproteobacteria	Rhizobiales	Hyphomicrobiaceae	uncultured	-	-	-	-	-	-	-	-	-	-	-	-	-	-	-	-	-	-	-	-	-	-	-	lip	-
4481957	0,0%	0,0%	0,0%	0,0%	0,0%	0,0%	0,0%	0,0%	0,0%	Bacteria	Firmicutes	Clostridia	Clostridiales	Peptococcaceae	Desulfosporosinus	-	napA	narG	-	-	-	nirK	-	nosZ	nifH	nifD	nrfA	ureA	dsrA	dsrB	-	-	-	-	-	-	-	-	-	-
4441855	0,0%	0,0%	0,0%	0,0%	0,0%	0,0%	0,0%	0,0%	0,0%	Bacteria	Firmicutes	Bacilli	Lactobacillales	Streptococcaceae	Streptococcus	-	napA	narG	-	-	nirS	nirK	norB	nosZ	-	-	-	ureA	-	-	-	-	-	-	-	laccase_Asco	laccase_Basidio	-	-	ppo
4385936	0,0%	0,0%	0,0%	0,0%	0,0%	0,0%	0,0%	0,0%	0,0%	Bacteria	Proteobacteria	Deltaproteobacteria	Desulfobacterales	Desulfobacteraceae	Sva0081 sediment group	-	-	-	-	-	-	-	-	-	-	-	-	-	-	-	-	-	-	-	-	-	-	-	-	-
4405710	0,0%	0,0%	0,0%	0,0%	0,0%	0,0%	0,0%	0,0%	0,0%	Bacteria	Acidobacteria	Acidobacteriia	Acidobacteriales	Acidobacteriaceae	Telmatobacter	cellulolytic	-	-	-	-	-	-	-	-	-	-	-	-	-	-	-	-	-	-	-	-	-	-	-	-
350585	0,0%	0,0%	0,0%	0,0%	0,0%	0,0%	0,0%	0,0%	0,0%	Bacteria	Proteobacteria	Deltaproteobacteria	Myxococcales		Phaselicystis	-	-	-	-	-	-	-	-	-	-	-	-	-	-	-	-	-	-	-	-	-	-	-	-	-
2792176	0,0%	0,0%	0,0%	0,0%	0,0%	0,0%	0,0%	0,0%	0,1%	Bacteria	Proteobacteria	Betaproteobacteria	Burkholderiales	Comamonadaceae	uncultured	-	-	-	-	-	-	-	-	-	-	-	-	-	-	-	-	-	-	-	-	-	-	-	lip	-
4326823	0,0%	0,0%	0,0%	0,0%	0,0%	0,0%	0,0%	0,0%	0,0%	Bacteria	Proteobacteria	Alphaproteobacteria	Rhodospirillales	Rhodospirillaceae	uncultured forest soil bacterium	-	-	-	-	-	-	-	-	-	-	-	-	-	-	-	-	-	-	-	-	-	-	-	-	-
2785	0,0%	0,0%	0,0%	0,0%	0,0%	0,0%	0,0%	0,0%	0,0%	Bacteria	Planctomycetes	Planctomycetia	Pirellulales	Pirellulaceae	Rhodopirellula	-	napA	-	nirA	-	-	-	norB	-	-	-	nrfA	-	-	-	-	-	-	-	-	-	laccase_Basidio	-	-	-
4325328	0,0%	0,0%	0,0%	0,0%	0,0%	0,0%	0,0%	0,0%	0,0%	Bacteria	Proteobacteria	Betaproteobacteria	Rhodocyclales	Rhodocyclaceae	uncultured	-	-	-	-	-	-	-	-	-	-	-	-	-	-	-	-	-	-	-	-	-	-	-	lip	-
255112	0,0%	0,0%	0,0%	0,0%	0,0%	0,0%	0,0%	0,0%	0,0%	Bacteria	Verrucomicrobia	Verrucomicrobiae	Verrucomicrobiales	Verrucomicrobiaceae	uncultured	-	-	-	-	-	-	-	-	-	-	-	-	-	-	-	-	-	-	-	-	-	-	-	lip	-
3488180	0,0%	0,0%	0,0%	0,0%	0,0%	0,0%	0,0%	0,0%	0,0%	Bacteria	Proteobacteria	Betaproteobacteria	Burkholderiales	Oxalobacteraceae	Oxalobacter	-	napA	-	-	-	-	-	-	-	-	-	-	-	-	-	-	-	-	-	-	-	-	-	-	-
249216	0,0%	0,0%	0,0%	0,0%	0,0%	0,0%	0,0%	0,0%	0,0%	Bacteria	Cyanobacteria	Synechococcophycideae	Pseudanabaenales	Pseudanabaenaceae	Leptolyngbya	-	napA	-	nirA	-	-	-	-	-	nifH	nifD	-	ureA	-	-	-	-	-	-	-	laccase_Asco	laccase_Basidio	-	-	ppo
4451935	0,0%	0,0%	0,0%	0,0%	0,0%	0,0%	0,0%	0,0%	0,0%	Bacteria	Proteobacteria	Betaproteobacteria	Methylophilales	Methylophilaceae	OM43 clade	-	-	-	-	-	-	-	-	-	-	-	-	-	-	-	-	-	-	-	-	-	-	-	-	-
203626	0,0%	0,0%	0,0%	0,0%	0,0%	0,0%	0,0%	0,0%	0,0%	Bacteria	Proteobacteria	Alphaproteobacteria	Ellin329		Rhizomicrobium	-	-	-	-	-	-	-	-	-	-	-	-	-	-	-	-	-	-	-	-	-	-	-	-	-
856343	0,0%	0,0%	0,0%	0,0%	0,0%	0,0%	0,0%	0,0%	0,0%	Bacteria	Proteobacteria	Betaproteobacteria	Burkholderiales	Comamonadaceae	Aquincola	-	-	-	-	-	-	-	-	-	-	-	-	-	-	-	-	-	-	-	-	-	-	-	-	-

4482374	0,0%	0,0%	0,0%	0,0%	0,0%	0,0%	0,0%	0,0%	0,0%	Bacteria	Proteobacteria	Gammaproteobacteria	Pseudomonadales	Moraxellaceae	Acinetobacter	-	napA	narG	nirA	-	-	-	-	-	nifH	-	-	ureA	-	-	-	-	-	mmoX	-	laccase_Asco	laccase_Basidio	-	-	ppo
834766	0,0%	0,0%	0,0%	0,0%	0,0%	0,0%	0,0%	0,0%	0,0%	Bacteria	Proteobacteria	Gammaproteobacteria	Aeromonadales	Aeromonadaceae	uncultured bacterium	-	-	-	-	-	-	-	-	-	-	-	-	-	-	-	-	-	-	-	-	-	-	-	-	-
6303	0,0%	0,0%	0,0%	0,0%	0,0%	0,0%	0,0%	0,0%	0,0%	Bacteria	Proteobacteria	Alphaproteobacteria	Sphingomonadales	Sphingomonadaceae	Novosphingobium	-	napA	-	-	-	-	-	norB	-	nifH	nifD	-	ureA	-	-	-	-	-	mmoX	-	laccase_Asco	laccase_Basidio	-	-	ppo
2769196	0,0%	0,0%	0,0%	0,0%	0,0%	0,0%	0,0%	0,0%	0,0%	Bacteria	Proteobacteria	Gammaproteobacteria	Aeromonadales	Aeromonadaceae	uncultured bacterium	-	-	-	-	-	-	-	-	-	-	-	-	-	-	-	-	-	-	-	-	-	-	-	-	-
679077	0,0%	0,0%	0,0%	0,0%	0,0%	0,0%	0,0%	0,0%	0,0%	Bacteria	Proteobacteria	Gammaproteobacteria	Chromatiales	Chromatiaceae	Thiocystis	-	napA	-	-	-	-	-	norB	-	nifH	nifD	-	ureA	dsrA	dsrB	-	-	-	-	-	-	-	-	-	-
4362183	0,0%	0,0%	0,0%	0,0%	0,0%	0,0%	0,0%	0,0%	0,0%	Bacteria	Proteobacteria	Gammaproteobacteria	Legionellales	Legionellaceae	Massilia	-	napA	narG	-	-	-	-	norB	nosZ	-	-	-	ureA	-	-	-	-	chb	mmoX	-	laccase_Asco	laccase_Basidio	-	-	ppo
654155	0,0%	0,0%	0,0%	0,0%	0,0%	0,0%	0,0%	0,0%	0,0%	Bacteria	Proteobacteria	Alphaproteobacteria	Caulobacterales	Caulobacteraceae	uncultured	-	-	-	-	-	-	-	-	-	-	-	-	-	-	-	-	-	-	-	-	-	-	-	lip	-
4394695	0,0%	0,0%	0,0%	0,0%	0,0%	0,0%	0,0%	0,0%	0,0%	Bacteria	Proteobacteria	Betaproteobacteria	Burkholderiales	Comamonadaceae	Xylophilus	-	napA	-	nirA	-	-	-	-	-	-	-	-	ureA	-	-	-	-	-	-	-	-	-	-	-	-
4482598	0,0%	0,0%	0,0%	0,0%	0,0%	0,0%	0,0%	0,0%	0,0%	Bacteria	Proteobacteria	Gammaproteobacteria	Pseudomonadales	Moraxellaceae	Acinetobacter	-	napA	narG	nirA	-	-	-	-	-	nifH	-	-	ureA	-	-	-	-	-	mmoX	-	laccase_Asco	laccase_Basidio	-	-	ppo
250967	0,0%	0,0%	0,0%	0,0%	0,0%	0,0%	0,0%	0,0%	0,1%	Bacteria	Proteobacteria	Deltaproteobacteria	Myxococcales		Haliangium	-	-	-	-	-	-	-	-	-	-	-	-	ureA	-	-	-	-	chb	-	-	-	-	-	-	-
4461030	0,0%	0,0%	0,0%	0,0%	0,0%	0,0%	0,0%	0,0%	0,0%	Bacteria	Proteobacteria	Gammaproteobacteria	Enterobacteriales	Enterobacteriaceae	Enterobacter	cellulolytic	napA	narG	-	-	-	-	-	-	nifH	nifD	nrfA	ureA	-	-	-	-	chb	-	-	laccase_Asco	laccase_Basidio	-	-	ppo
454145	0,0%	0,0%	0,0%	0,0%	0,0%	0,0%	0,0%	0,0%	0,0%	Bacteria	Spirochaetes	MVP-15	PL-11B10		uncultured bacterium	-	-	-	-	-	-	-	-	-	-	-	-	-	-	-	-	-	-	-	-	-	-	-	-	-
4481484	0,0%	0,0%	0,0%	0,0%	0,0%	0,0%	0,0%	0,0%	0,0%	Bacteria	Bacteroidetes	Flavobacteriia	Flavobacteriales	Flavobacteriaceae	Flavobacterium	cellulolytic	napA	-	nirA	-	-	nirK	norB	nosZ	-	-	-	ureA	-	-	-	-	-	-	-	laccase_Asco	laccase_Basidio	-	-	ppo
974817	0,0%	0,0%	0,0%	0,0%	0,0%	0,0%	0,0%	0,0%	0,0%	Bacteria	Actinobacteria	Thermoleophilia	Solirubrobacterales		uncultured bacterium	-	-	-	-	-	-	-	-	-	-	-	-	-	-	-	-	-	-	-	-	-	-	-	-	-
219226	0,0%	0,0%	0,0%	0,0%	0,0%	0,0%	0,0%	0,0%	0,0%	Bacteria	Gemmatimonadetes	Gemmatimonadetes	Gemmatimonadales	A1-B1	Gemmatimonas	-	napA	-	-	-	-	nirK	norB	nosZ	-	-	nrfA	-	dsrA	dsrB	-	-	chb	-	-	-	-	-	-	-
564911	0,0%	0,0%	0,0%	0,0%	0,0%	0,0%	0,0%	0,0%	0,0%	Bacteria	Planctomycetes	Planctomycetia	Gemmatales	Isosphaeraceae	Isosphaera	-	napA	-	-	-	-	-	-	-	-	-	-	-	-	-	-	-	-	-	-	-	-	-	-	-
4467425	0,0%	0,0%	0,0%	0,0%	0,0%	0,0%	0,0%	0,0%	0,0%	Bacteria	Proteobacteria	Alphaproteobacteria	Rhizobiales	Hyphomicrobiaceae	uncultured	-	-	-	-	-	-	-	-	-	-	-	-	-	-	-	-	-	-	-	-	-	-	-	lip	-
2731070	0,0%	0,0%	0,0%	0,0%	0,0%	0,0%	0,0%	0,0%	0,0%	Bacteria	Proteobacteria	Alphaproteobacteria	Rhodobacterales	Rhodobacteraceae	Rhodovulum	-	napA	-	nirA	-	-	-	-	-	nifH	nifD	-	ureA	-	-	-	-	-	-	-	-	-	-	-	-
4462101	0,0%	0,0%	0,0%	0,0%	0,0%	0,0%	0,0%	0,0%	0,0%	Bacteria	Proteobacteria	Alphaproteobacteria	Rhodospirillales	Rhodospirillaceae	Azospirillum	-	napA	-	nirA	-	nirS	nirK	norB	nosZ	nifH	nifD	-	ureA	-	-	-	-	-	mmoX	-	laccase_Asco	laccase_Basidio	-	-	ppo
357366	0,0%	0,0%	0,0%	0,0%	0,0%	0,0%	0,0%	0,0%	0,0%	Bacteria	Proteobacteria	Alphaproteobacteria	Sphingomonadales	Sphingomonadaceae	Altererythrobacter	-	napA	narG	-	-	-	-	-	-	-	-	-	-	-	-	-	-	-	-	-	laccase_Asco	laccase_Basidio	-	-	ppo
419579	0,0%	0,0%	0,0%	0,0%	0,0%	0,0%	0,0%	0,0%	0,0%	Bacteria	Proteobacteria	Betaproteobacteria			uncultured	-	-	-	-	-	-	-	-	-	-	-	-	-	-	-	-	-	-	-	-	-	-	-	lip	-
1125052	0,0%	0,0%	0,0%	0,0%	0,0%	0,0%	0,0%	0,0%	0,0%	Bacteria	Proteobacteria	Betaproteobacteria	SBla14		Sideroxydans	-	napA	-	-	-	nirS	-	norB	-	nifH	nifD	-	-	dsrA	dsrB	-	-	-	-	-	-	-	-	-	-
619348	0,0%	0,0%	0,0%	0,0%	0,0%	0,0%	0,0%	0,0%	0,0%	Bacteria	Proteobacteria	Gammaproteobacteria	Methylococcales	Crenotrichaceae	Methylobacter	-	-	-	-	-	-	-	-	-	nifH	nifD	-	-	-	-	-	-	-	-	pmoA	-	-	-	-	-
512654	0,0%	0,0%	0,0%	0,0%	0,0%	0,0%	0,0%	0,0%	0,0%	Bacteria	OP3	PBS-25			uncultured Verrucomicrobia bacterium	-	-	-	-	-	-	-	-	-	-	-	-	-	-	-	-	-	-	-	-	-	-	-	-	-
232270	0,0%	0,0%	0,0%	0,0%	0,0%	0,0%	0,0%	0,0%	0,0%	Bacteria	Proteobacteria	Alphaproteobacteria	Rhodospirillales	Rhodospirillaceae	uncultured	-	-	-	-	-	-	-	-	-	-	-	-	-	-	-	-	-	-	-	-	-	-	-	lip	-
1117736	0,0%	0,0%	0,0%	0,0%	0,0%	0,0%	0,0%	0,0%	0,0%	Bacteria	Firmicutes	Clostridia	Clostridiales	Ruminococcaceae	Incertae Sedis	cellulolytic	-	-	-	-	-	-	-	-	-	-	-	-	-	-	-	-	-	-	-	-	-	-	-	-
986448	0,0%	0,0%	0,0%	0,0%	0,0%	0,0%	0,0%	0,0%	0,0%	Bacteria	Proteobacteria	Deltaproteobacteria	Bdellovibrionales	Bdellovibrionaceae	Bdellovibrio	-	-	-	-	-	-	nirK	norB	-	-	-	nrfA	-	-	-	-	-	-	-	-	laccase_Asco	laccase_Basidio	-	-	ppo
4337197	0,0%	0,0%	0,0%	0,0%	0,0%	0,0%	0,0%	0,0%	0,0%	Bacteria	Bacteroidetes	[Saprospirae]	[Saprospirales]	Chitinophagaceae	uncultured	-	-	-	-	-	-	-	-	-	-	-	-	-	-	-	-	-	-	-	-	-	-	-	lip	-
278075	0,0%	0,0%	0,0%	0,0%	0,0%	0,0%	0,0%	0,0%	0,0%	Bacteria	Proteobacteria	Alphaproteobacteria	Sphingomonadales	Sphingomonadaceae	Sandarakinorhabdus	-	-	-	-	-	-	-	-	-	-	-	-	-	-	-	-	-	-	-	-	-	-	-	-	-
4398067	0,0%	0,0%	0,0%	0,0%	0,0%	0,0%	0,0%	0,0%	0,0%	Bacteria	Actinobacteria	Actinobacteria	Actinomycetales	Nocardiaceae	Nocardia	-	napA	narG	-	-	-	nirK	-	-	-	-	-	ureA	-	-	-	-	-	mmoX	-	laccase_Asco	laccase_Basidio	-	-	ppo
4377697	0,0%	0,0%	0,0%	0,0%	0,0%	0,0%	0,0%	0,0%	0,0%	Bacteria	Proteobacteria	Betaproteobacteria	Burkholderiales	Comamonadaceae	Paucibacter	-	-	-	-	-	-	-	-	-	-	-	-	-	-	-	-	-	-	-	-	-	-	-	-	-
822806	0,0%	0,0%	0,0%	0,0%	0,0%	0,0%	0,0%	0,0%	0,0%	Bacteria	Chloroflexi	Anaerolineae	SBR1031	A4b	uncultured	-	-	-	-	-	-	-	-	-	-	-	-	-	-	-	-	-	-	-	-	-	-	-	lip	-
247304	0,0%	0,0%	0,0%	0,0%	0,0%	0,0%	0,0%	0,0%	0,0%	Bacteria	Proteobacteria	Gammaproteobacteria	Oceanospirillales		Kangiella	-	-	narG	-	-	nirS	nirK	norB	-	-	-	-	-	-	-	-	-	-	-	-	laccase_Asco	laccase_Basidio	-	-	-
408706	0,0%	0,0%	0,0%	0,0%	0,0%	0,0%	0,0%	0,0%	0,0%	Bacteria	Bacteroidetes	Flavobacteriia	Flavobacteriales	[Weeksellaceae]	Chryseobacterium	-	napA	-	-	-	-	nirK	norB	nosZ	nifH	-	-	ureA	-	-	-	-	chb	-	-	laccase_Asco	laccase_Basidio	-	-	ppo
779751	0,0%	0,0%	0,0%	0,0%	0,0%	0,0%	0,0%	0,0%	0,0%	Bacteria	Proteobacteria	Betaproteobacteria	Rhodocyclales	Rhodocyclaceae	Dechloromonas	-	napA	narG	-	-	nirS	-	norB	nosZ	nifH	nifD	-	ureA	-	-	SoxB	-	-	mmoX	-	-	-	-	-	-
99452	0,0%	0,0%	0,0%	0,0%	0,0%	0,0%	0,0%	0,0%	0,0%	Bacteria	Proteobacteria	Gammaproteobacteria	Legionellales	Legionellaceae	Legionella	-	-	-	-	-	nirS	-	norB	-	-	-	-	-	-	-	-	-	-	-	-	laccase_Asco	laccase_Basidio	-	-	ppo
4308264	0,0%	0,0%	0,0%	0,0%	0,0%	0,0%	0,0%	0,0%	0,0%	Bacteria	Proteobacteria	Deltaproteobacteria	Bdellovibrionales	Bdellovibrionaceae	Bdellovibrio	-	-	-	-	-	-	nirK	norB	-	-	-	nrfA	-	-	-	-	-	-	-	-	laccase_Asco	laccase_Basidio	-	-	ppo
112562	0,0%	0,0%	0,0%	0,0%	0,0%	0,0%	0,0%	0,0%	0,0%	Bacteria	Proteobacteria	Deltaproteobacteria	Myxococcales		Haliangium	-	-	-	-	-	-	-	-	-	-	-	-	ureA	-	-	-	-	chb	-	-	-	-	-	-	-
210410	0,0%	0,0%	0,0%	0,0%	0,0%	0,0%	0,0%	0,0%	0,0%	Bacteria	Actinobacteria	Actinobacteria	Actinomycetales		Acidothermus	-	napA	narG	-	-	-	nirK	-	-	-	-	-	-	-	-	-	-	-	-	-	-	-	-	-	-
1062909	0,0%	0,0%	0,0%	0,0%	0,0%	0,0%	0,0%	0,0%	0,0%	Bacteria	Bacteroidetes	[Saprospirae]	[Saprospirales]	Chitinophagaceae	Hydrotalea	-	-	-	-	-	-	-	-	-	-	-	-	-	-	-	-	-	-	-	-	-	-	-	-	-
285320	0,0%	0,0%	0,0%	0,0%	0,0%	0,0%	0,0%	0,0%	0,0%	Bacteria	Chloroflexi	Ktedonobacteria	Thermogemmatisporales	Thermogemmatisporaceae	uncultured bacterium	-	-	-	-	-	-	-	-	-	-	-	-	-	-	-	-	-	-	-	-	-	-	-	-	-
30929	0,0%	0,0%	0,0%	0,0%	0,0%	0,0%	0,0%	0,0%	0,0%	Bacteria	Proteobacteria	Gammaproteobacteria	Enterobacteriales	Enterobacteriaceae	Rahnella	cellulolytic	napA	narG	-	-	-	-	-	-	nifH	nifD	-	-	-	-	-	-	-	-	-	laccase_Asco	laccase_Basidio	-	-	-
986534	0,0%	0,0%	0,0%	0,0%	0,0%	0,0%	0,0%	0,0%	0,0%	Bacteria	Bacteroidetes	Cytophagia	Cytophagales		uncultured	-	-	-	-	-	-	-	-	-	-	-	-	-	-	-	-	-	-	-	-	-	-	-	lip	-
1012668	0,0%	0,0%	0,0%	0,0%	0,0%	0,0%	0,0%	0,0%	0,1%	Bacteria	Actinobacteria	Acidimicrobiia	Acidimicrobiales	EB1017	uncultured bacterium	-	-	-	-	-	-	-	-	-	-	-	-	-	-	-	-	-	-	-	-	-	-	-	-	-
4466646	0,0%	0,0%	0,0%	0,0%	0,0%	0,0%	0,0%	0,0%	0,0%	Bacteria	Proteobacteria	Betaproteobacteria	Burkholderiales	Comamonadaceae	uncultured	-	-	-	-	-	-	-	-	-	-	-	-	-	-	-	-	-	-	-	-	-	-	-	lip	-
1148343	0,0%	0,0%	0,0%	0,0%	0,0%	0,0%	0,0%	0,0%	0,0%	Bacteria	Planctomycetes	Planctomycetia	Gemmatales	Gemmataceae	Gemmata	-	-	-	-	-	-	-	-	-	-	-	-	-	-	-	-	-	-	-	-	-	-	-	-	-
4418677	0,0%	0,0%	0,0%	0,0%	0,0%	0,0%	0,0%	0,0%	0,0%	Bacteria	Proteobacteria	Betaproteobacteria	Burkholderiales	Comamonadaceae	Leptothrix	-	napA	-	-	-	nirS	-	norB	nosZ	nifH	nifD	-	ureA	-	-	-	-	-	mmoX	-	-	-	-	-	-
3900312	0,0%	0,0%	0,0%	0,0%	0,0%	0,0%	0,0%	0,0%	0,0%	Bacteria	Proteobacteria	Alphaproteobacteria	Sphingomonadales	Sphingomonadaceae	Novosphingobium	-	napA	-	-	-	-	-	norB	-	nifH	nifD	-	ureA	-	-	-	-	-	mmoX	-	laccase_Asco	laccase_Basidio	-	-	ppo
4381473	0,0%	0,0%	0,0%	0,0%	0,0%	0,0%	0,0%	0,0%	0,0%	Bacteria	Proteobacteria	Alphaproteobacteria	Sphingomonadales	Sphingomonadaceae	Altererythrobacter	-	napA	narG	-	-	-	-	-	-	-	-	-	-	-	-	-	-	-	-	-	laccase_Asco	laccase_Basidio	-	-	ppo
666038	0,0%	0,0%	0,0%	0,0%	0,0%	0,0%	0,0%	0,0%	0,0%	Bacteria	Acidobacteria	Solibacteres	Solibacterales	Solibacteraceae	Bryobacter	-	-	-	-	-	-	-	-	-	-	-	-	-	-	-	-	-	-	-	-	-	-	-	-	-
226314	0,0%	0,0%	0,0%	0,0%	0,0%	0,0%	0,0%	0,0%	0,0%	Bacteria	Proteobacteria	Deltaproteobacteria			uncultured delta proteobacterium	-	-	-	-	-	-	-	-	-	-	-	-	-	-	-	-	-	-	-	-	-	-	-	-	-
1121640	0,0%	0,0%	0,0%	0,0%	0,0%	0,0%	0,0%	0,0%	0,0%	Bacteria	Proteobacteria	Betaproteobacteria			uncultured	-	-	-	-	-	-	-	-	-	-	-	-	-	-	-	-	-	-	-	-	-	-	-	lip	-
94840	0,0%	0,0%	0,0%	0,0%	0,0%	0,0%	0,0%	0,0%	0,0%	Bacteria	Proteobacteria	Gammaproteobacteria	Methylococcales	Crenotrichaceae	Crenothrix	-	-	-	-	-	-	-	-	-	-	-	-	-	-	-	-	-	-	-	pmoA	-	-	-	-	-
226312	0,0%	0,0%	0,0%	0,0%	0,0%	0,0%	0,0%	0,0%	0,0%	Bacteria	Proteobacteria	Alphaproteobacteria	Rhodospirillales	Acetobacteraceae	uncultured	-	-	-	-	-	-	-	-	-	-	-	-	-	-	-	-	-	-	-	-	-	-	-	lip	-
4480289	0,0%	0,0%	0,0%	0,0%	0,0%	0,0%	0,0%	0,0%	0,0%	Bacteria	Chloroflexi	S085			uncultured bacterium	-	-	-	-	-	-	-	-	-	-	-	-	-	-	-	-	-	-	-	-	-	-	-	-	-
4389643	0,0%	0,0%	0,0%	0,0%	0,0%	0,0%	0,0%	0,0%	0,0%	Bacteria	Proteobacteria	Betaproteobacteria	Rhodocyclales	Rhodocyclaceae	Denitratisoma	-	-	-	-	-	-	-	-	-	-	-	-	-	-	-	-	-	-	-	-	-	-	-	-	-
630306	0,0%	0,0%	0,0%	0,0%	0,0%	0,0%	0,0%	0,0%	0,0%	Bacteria	Verrucomicrobia	[Spartobacteria]	[Chthoniobacterales]	[Chthoniobacteraceae]	Candidatus Xiphinematobacter	-	-	-	-	-	-	-	-	-	-	-	-	-	-	-	-	-	-	-	-	-	-	-	-	-
4452118	0,0%	0,0%	0,0%	0,0%	0,0%	0,0%	0,0%	0,0%	0,0%	Bacteria	Proteobacteria	Betaproteobacteria	Burkholderiales	Comamonadaceae	Pelomonas	-	napA	narG	nirA	-	nirS	-	norB	nosZ	nifH	-	-	ureA	-	-	-	-	-	-	-	-	-	-	-	-
4469134	0,0%	0,0%	0,0%	0,0%	0,0%	0,0%	0,0%	0,0%	0,0%	Bacteria	Proteobacteria	Deltaproteobacteria	Myxococcales	Polyangiaceae	Byssovorax	-	-	-	-	-	-	-	-	-	-	-	-	-	-	-	-	-	-	-	-	-	-	-	-	-
4320526	0,0%	0,0%	0,0%	0,0%	0,0%	0,0%	0,0%	0,0%	0,0%	Bacteria	Bacteroidetes	Sphingobacteriia	Sphingobacteriales	Sphingobacteriaceae	Mucilaginibacter	-	-	-	-	-	-	-	-	-	-	-	-	-	-	-	-	-	-	-	-	-	-	-	-	-
4429335	0,0%	0,0%	0,0%	0,0%	0,0%	0,0%	0,0%	0,0%	0,0%	Bacteria	Firmicutes	Clostridia	Clostridiales	[Tissierellaceae]	Peptoniphilus	-	napA	-	-	-	-	-	-	-	nifH	nifD	-	-	-	-	-	-	-	-	-	-	-	-	-	-
804686	0,0%	0,0%	0,0%	0,0%	0,0%	0,0%	0,0%	0,0%	0,0%	Bacteria	Proteobacteria	Gammaproteobacteria	Aeromonadales	Aeromonadaceae	Aeromonas	-	napA	narG	-	-	-	-	-	nosZ	nifH	-	nrfA	ureA	-	dsrB	-	-	chb	-	-	laccase_Asco	laccase_Basidio	-	-	ppo
795955	0,0%	0,0%	0,0%	0,0%	0,0%	0,0%	0,0%	0,0%	0,0%	Bacteria	Bacteroidetes	Sphingobacteriia	Sphingobacteriales	Sphingobacteriaceae	Mucilaginibacter	-	-	-	-	-	-	-	-	-	-	-	-	-	-	-	-	-	-	-	-	-	-	-	-	-
685193	0,0%	0,0%	0,0%	0,0%	0,0%	0,0%	0,0%	0,0%	0,0%	Bacteria	Planctomycetes	Planctomycetia	Gemmatales	Isosphaeraceae	uncultured bacterium	-	-	-	-	-	-	-	-	-	-	-	-	-	-	-	-	-	-	-	-	-	-	-	-	-
79659	0,0%	0,0%	0,0%	0,0%	0,0%	0,0%	0,0%	0,0%	0,0%	Bacteria	Proteobacteria	Betaproteobacteria	Burkholderiales	Comamonadaceae	Pelomonas	-	napA	narG	nirA	-	nirS	-	norB	nosZ	nifH	-	-	ureA	-	-	-	-	-	-	-	-	-	-	-	-
72607	0,0%	0,0%	0,0%	0,0%	0,0%	0,0%	0,0%	0,0%	0,0%	Bacteria	Proteobacteria	Betaproteobacteria	Burkholderiales	Comamonadaceae	Limnohabitans	-	napA	narG	-	-	-	-	-	-	-	-	-	ureA	-	-	-	-	-	-	-	-	-	-	-	-
111304	0,0%	0,0%	0,0%	0,0%	0,0%	0,0%	0,0%	0,0%	0,0%	Bacteria	Proteobacteria	Alphaproteobacteria	Sphingomonadales	Sphingomonadaceae	Altererythrobacter	-	napA	narG	-	-	-	-	-	-	-	-	-	-	-	-	-	-	-	-	-	laccase_Asco	laccase_Basidio	-	-	ppo
4432360	0,0%	0,0%	0,0%	0,0%	0,0%	0,0%	0,0%	0,0%	0,0%	Bacteria	Cyanobacteria	Synechococcophycideae	Pseudanabaenales	Pseudanabaenaceae	Leptolyngbya	-	napA	-	nirA	-	-	-	-	-	nifH	nifD	-	ureA	-	-	-	-	-	-	-	laccase_Asco	laccase_Basidio	-	-	ppo
4302753	0,0%	0,0%	0,0%	0,0%	0,0%	0,0%	0,0%	0,0%	0,0%	Bacteria	Proteobacteria	Deltaproteobacteria	Myxococcales		uncultured Cystobacteraceae bacterium	-	-	-	-	-	-	-	-	-	-	-	-	-	-	-	-	-	-	-	-	-	-	-	-	-
1126149	0,0%	0,0%	0,0%	0,0%	0,0%	0,0%	0,0%	0,0%	0,0%	Bacteria	Proteobacteria	Alphaproteobacteria	Sphingomonadales	Sphingomonadaceae	Sphingomonas	-	napA	narG	nirA	-	-	nirK	norB	-	nifH	nifD	-	ureA	-	-	-	-	chb	mmoX	-	laccase_Asco	laccase_Basidio	-	-	ppo
4301491	0,0%	0,0%	0,0%	0,0%	0,0%	0,0%	0,0%	0,0%	0,0%	Bacteria	Firmicutes	Bacilli	Bacillales	Bacillaceae	Anoxybacillus	cellulolytic	napA	narG	-	-	-	-	-	nosZ	-	-	nrfA	ureA	-	-	-	-	-	-	-	laccase_Asco	laccase_Basidio	-	-	-
4164931	0,0%	0,0%	0,0%	0,0%	0,0%	0,0%	0,0%	0,0%	0,0%	Bacteria	Proteobacteria	Alphaproteobacteria	Sphingomonadales	Sphingomonadaceae	Sphingopyxis	-	napA	-	-	-	-	-	norB	-	-	-	-	-	-	-	-	-	chb	-	-	laccase_Asco	laccase_Basidio	-	-	ppo
212223	0,0%	0,0%	0,0%	0,0%	0,0%	0,0%	0,0%	0,0%	0,0%	Bacteria	Proteobacteria	Alphaproteobacteria	Ellin329		Rhizomicrobium	-	-	-	-	-	-	-	-	-	-	-	-	-	-	-	-	-	-	-	-	-	-	-	-	-
4478148	0,0%	0,0%	0,0%	0,0%	0,0%	0,0%	0,0%	0,0%	0,0%	Bacteria	Proteobacteria	Betaproteobacteria	Burkholderiales	Comamonadaceae	Xylophilus	-	napA	-	nirA	-	-	-	-	-	-	-	-	ureA	-	-	-	-	-	-	-	-	-	-	-	-
747226	0,0%	0,0%	0,0%	0,0%	0,0%	0,0%	0,0%	0,0%	0,0%	Bacteria	Bacteroidetes	Cytophagia	Cytophagales	Cytophagaceae	Cytophaga	cellulolytic	napA	-	-	-	-	-	norB	-	-	nifD	-	ureA	-	-	-	-	-	-	-	-	-	-	-	-
99343	0,0%	0,0%	0,0%	0,0%	0,0%	0,0%	0,0%	0,0%	0,0%	Bacteria	Proteobacteria	Deltaproteobacteria	Myxococcales	Haliangiaceae	Haliangium	-	-	-	-	-	-	-	-	-	-	-	-	ureA	-	-	-	-	chb	-	-	-	-	-	-	-
4296820	0,0%	0,0%	0,0%	0,0%	0,0%	0,0%	0,0%	0,0%	0,0%	Bacteria	Proteobacteria	Deltaproteobacteria	Myxococcales		uncultured Myxococcales bacterium	-	-	-	-	-	-	-	-	-	-	-	-	-	-	-	-	-	-	-	-	-	-	-	-	-
4011130	0,0%	0,0%	0,0%	0,0%	0,0%	0,0%	0,0%	0,0%	0,0%	Bacteria	Proteobacteria	Gammaproteobacteria			Pseudospirillum	-	-	-	-	-	-	-	-	-	-	-	-	-	-	-	-	-	-	-	-	-	-	-	-	-
242577	0,0%	0,0%	0,0%	0,0%	0,0%	0,0%	0,0%	0,0%	0,0%	Bacteria	Actinobacteria	Actinobacteria	Actinomycetales	ACK-M1	hgcI clade	-	-	-	-	-	-	-	-	-	-	-	-	-	-	-	-	-	-	-	-	-	-	-	-	-
217328	0,0%	0,0%	0,0%	0,0%	0,0%	0,0%	0,0%	0,0%	0,0%	Bacteria	Proteobacteria	Deltaproteobacteria	Myxococcales	Haliangiaceae	Haliangium	-	-	-	-	-	-	-	-	-	-	-	-	ureA	-	-	-	-	chb	-	-	-	-	-	-	-
216255	0,0%	0,0%	0,0%	0,0%	0,0%	0,0%	0,0%	0,0%	0,0%	Bacteria	Verrucomicrobia	[Spartobacteria]	[Chthoniobacterales]	[Chthoniobacteraceae]	Chthoniobacter	-	napA	-	-	-	-	nirK	-	-	-	-	nrfA	ureA	-	-	-	-	-	-	-	-	-	-	-	-
585070	0,0%	0,0%	0,0%	0,0%	0,0%	0,0%	0,0%	0,0%	0,0%	Bacteria	Proteobacteria	Alphaproteobacteria	Sphingomonadales	Sphingomonadaceae	uncultured bacterium	-	-	-	-	-	-	-	-	-	-	-	-	-	-	-	-	-	-	-	-	-	-	-	-	-
1040109	0,0%	0,0%	0,0%	0,0%	0,0%	0,0%	0,0%	0,0%	0,0%	Bacteria	Chloroflexi	Thermomicrobia	Ellin6537		uncultured bacterium	-	-	-	-	-	-	-	-	-	-	-	-	-	-	-	-	-	-	-	-	-	-	-	-	-
3507207	0,0%	0,0%	0,0%	0,0%	0,0%	0,0%	0,0%	0,0%	0,0%	Bacteria	Proteobacteria	Alphaproteobacteria	Rhodospirillales	Rhodospirillaceae	Azospirillum	-	napA	-	nirA	-	nirS	nirK	norB	nosZ	nifH	nifD	-	ureA	-	-	-	-	-	mmoX	-	laccase_Asco	laccase_Basidio	-	-	ppo
4386593	0,0%	0,0%	0,0%	0,0%	0,0%	0,0%	0,0%	0,0%	0,0%	Bacteria	Firmicutes	Clostridia	Clostridiales	Clostridiaceae	Clostridium sensu stricto 9	cellulolytic	-	-	-	-	-	-	-	-	-	-	-	-	-	-	-	-	-	-	-	-	-	-	-	-
846667	0,0%	0,0%	0,0%	0,0%	0,0%	0,0%	0,0%	0,0%	0,0%	Bacteria	Acidobacteria	Acidobacteriia	Acidobacteriales	Acidobacteriaceae	Acidicapsa	cellulolytic	-	-	-	-	-	-	-	-	-	-	-	-	-	-	-	-	-	-	-	-	-	-	-	-
825276	0,0%	0,0%	0,0%	0,0%	0,0%	0,0%	0,0%	0,0%	0,0%	Bacteria	Proteobacteria	Betaproteobacteria	SC-I-84		uncultured Burkholderia sp.	-	-	-	-	-	-	-	-	-	-	-	-	-	-	-	-	-	-	-	-	-	-	-	-	-
1064392	0,0%	0,0%	0,0%	0,0%	0,0%	0,0%	0,0%	0,0%	0,0%	Bacteria	Proteobacteria	Gammaproteobacteria	Legionellales	Coxiellaceae	Rickettsiella	-	-	-	-	-	-	-	-	-	-	-	-	-	-	-	-	-	-	-	-	-	laccase_Basidio	-	-	-
712582	0,0%	0,0%	0,0%	0,0%	0,0%	0,0%	0,0%	0,0%	0,0%	Bacteria	Fibrobacteres	Fibrobacteria	258ds10		uncultured	-	-	-	-	-	-	-	-	-	-	-	-	-	-	-	-	-	-	-	-	-	-	-	lip	-
513834	0,0%	0,0%	0,0%	0,0%	0,0%	0,0%	0,0%	0,0%	0,0%	Bacteria	Planctomycetes	Planctomycetia	Gemmatales	Gemmataceae	Gemmata	-	-	-	-	-	-	-	-	-	-	-	-	-	-	-	-	-	-	-	-	-	-	-	-	-
2912622	0,0%	0,0%	0,0%	0,0%	0,0%	0,0%	0,0%	0,0%	0,0%	Bacteria	Proteobacteria	Betaproteobacteria	Neisseriales	Neisseriaceae	Aquitalea	-	-	-	-	-	-	-	-	-	-	-	-	-	-	-	-	-	-	-	-	-	-	-	-	-
4480061	0,0%	0,0%	0,0%	0,0%	0,0%	0,0%	0,0%	0,0%	0,0%	Bacteria	Proteobacteria	Alphaproteobacteria	Rhodobacterales	Rhodobacteraceae	Rhodovulum	-	napA	-	nirA	-	-	-	-	-	nifH	nifD	-	ureA	-	-	-	-	-	-	-	-	-	-	-	-
1006382	0,0%	0,0%	0,0%	0,0%	0,0%	0,0%	0,0%	0,0%	0,0%	Bacteria	Bacteroidetes	Sphingobacteriia	Sphingobacteriales		uncultured bacterium	-	-	-	-	-	-	-	-	-	-	-	-	-	-	-	-	-	-	-	-	-	-	-	-	-
1634029	0,0%	0,0%	0,0%	0,0%	0,0%	0,0%	0,0%	0,0%	0,0%	Bacteria	Proteobacteria	Betaproteobacteria	Burkholderiales	Comamonadaceae	Variovorax	-	napA	narG	nirA	-	-	-	norB	-	-	-	-	ureA	-	-	-	-	-	mmoX	-	-	-	-	-	-
4434213	0,0%	0,0%	0,0%	0,0%	0,0%	0,0%	0,0%	0,0%	0,0%	Bacteria	Proteobacteria	Deltaproteobacteria	Desulfuromonadales	Geobacteraceae	uncultured bacterium	-	-	-	-	-	-	-	-	-	-	-	-	-	-	-	-	-	-	-	-	-	-	-	-	-
359924	0,0%	0,0%	0,0%	0,0%	0,0%	0,0%	0,0%	0,0%	0,0%	Bacteria	Planctomycetes	Planctomycetia	Gemmatales	Gemmataceae	Gemmata	-	-	-	-	-	-	-	-	-	-	-	-	-	-	-	-	-	-	-	-	-	-	-	-	-
553387	0,0%	0,0%	0,0%	0,0%	0,0%	0,0%	0,0%	0,0%	0,0%	Bacteria	Acidobacteria	Acidobacteriia	Acidobacteriales	Acidobacteriaceae	Telmatobacter	cellulolytic	-	-	-	-	-	-	-	-	-	-	-	-	-	-	-	-	-	-	-	-	-	-	-	-
228545	0,0%	0,0%	0,0%	0,0%	0,0%	0,0%	0,0%	0,0%	0,0%	Bacteria	Proteobacteria	Gammaproteobacteria	Legionellales	Legionellaceae	Legionella	-	-	-	-	-	nirS	-	norB	-	-	-	-	-	-	-	-	-	-	-	-	laccase_Asco	laccase_Basidio	-	-	ppo
New.ReferenceOTU913	0,0%	0,0%	0,0%	0,0%	0,0%	0,0%	0,0%	0,0%	0,0%	Bacteria	Proteobacteria	Deltaproteobacteria	Bdellovibrionales	Bdellovibrionaceae	Bdellovibrio	-	-	-	-	-	-	nirK	norB	-	-	-	nrfA	-	-	-	-	-	-	-	-	laccase_Asco	laccase_Basidio	-	-	ppo
New.ReferenceOTU914	0,0%	0,0%	0,0%	0,0%	0,0%	0,0%	0,0%	0,0%	0,0%	Bacteria	Proteobacteria	Deltaproteobacteria	Myxococcales		uncultured Myxococcales bacterium	-	-	-	-	-	-	-	-	-	-	-	-	-	-	-	-	-	-	-	-	-	-	-	-	-
New.ReferenceOTU915	0,0%	0,0%	0,0%	0,0%	0,0%	0,0%	0,0%	0,0%	0,0%	Bacteria	Proteobacteria	Betaproteobacteria	Thiobacterales		Burkholderia	-	napA	narG	nirA	-	nirS	nirK	norB	nosZ	nifH	nifD	-	ureA	-	-	-	-	chb	mmoX	-	laccase_Asco	laccase_Basidio	-	-	ppo
New.ReferenceOTU916	0,0%	0,0%	0,0%	0,0%	0,0%	0,0%	0,0%	0,0%	0,0%	Bacteria	Verrucomicrobia	[Spartobacteria]	[Chthoniobacterales]	[Chthoniobacteraceae]	uncultured Verrucomicrobia bacterium	-	-	-	-	-	-	-	-	-	-	-	-	-	-	-	-	-	-	-	-	-	-	-	-	-
New.ReferenceOTU917	0,0%	0,0%	0,0%	0,0%	0,0%	0,0%	0,0%	0,0%	0,0%	Bacteria	Proteobacteria	Betaproteobacteria	Burkholderiales	Comamonadaceae	Aquincola	-	-	-	-	-	-	-	-	-	-	-	-	-	-	-	-	-	-	-	-	-	-	-	-	-
New.ReferenceOTU919	0,0%	0,0%	0,0%	0,0%	0,0%	0,0%	0,0%	0,0%	0,0%	Bacteria	Proteobacteria	Gammaproteobacteria	Legionellales		uncultured bacterium	-	-	-	-	-	-	-	-	-	-	-	-	-	-	-	-	-	-	-	-	-	-	-	-	-
New.ReferenceOTU1124	0,0%	0,0%	0,0%	0,0%	0,0%	0,0%	0,0%	0,0%	0,0%	Bacteria	Proteobacteria	Alphaproteobacteria	Rhodobacterales	Rhodobacteraceae	Rhodovulum	-	napA	-	nirA	-	-	-	-	-	nifH	nifD	-	ureA	-	-	-	-	-	-	-	-	-	-	-	-
New.ReferenceOTU497	0,0%	0,0%	0,0%	0,0%	0,0%	0,0%	0,0%	0,0%	0,0%	Bacteria	Proteobacteria	Alphaproteobacteria	Rickettsiales	Rickettsiaceae	uncultured bacterium	-	-	-	-	-	-	-	-	-	-	-	-	-	-	-	-	-	-	-	-	-	-	-	-	-
New.ReferenceOTU496	0,0%	0,0%	0,0%	0,0%	0,0%	0,0%	0,0%	0,0%	0,0%	Unassigned					Thalassospira	-	napA	narG	-	-	nirS	-	-	nosZ	-	-	-	ureA	-	-	-	-	-	-	-	laccase_Asco	laccase_Basidio	-	-	ppo
New.ReferenceOTU1269	0,0%	0,0%	0,0%	0,0%	0,0%	0,0%	0,0%	0,0%	0,0%	Bacteria	Bacteroidetes	[Saprospirae]	[Saprospirales]	Saprospiraceae	uncultured	-	-	-	-	-	-	-	-	-	-	-	-	-	-	-	-	-	-	-	-	-	-	-	lip	-
New.ReferenceOTU1264	0,0%	0,0%	0,0%	0,0%	0,0%	0,0%	0,0%	0,0%	0,0%	Bacteria	Proteobacteria	Gammaproteobacteria	Xanthomonadales	Sinobacteraceae	uncultured	-	-	-	-	-	-	-	-	-	-	-	-	-	-	-	-	-	-	-	-	-	-	-	lip	-
New.ReferenceOTU1265	0,0%	0,0%	0,0%	0,0%	0,0%	0,0%	0,0%	0,0%	0,0%	Bacteria	Bacteroidetes	Flavobacteriia	Flavobacteriales	[Weeksellaceae]	Epilithonimonas	-	-	-	-	-	-	-	-	-	-	-	-	-	-	-	-	-	-	-	-	-	-	-	-	-
New.ReferenceOTU1266	0,0%	0,0%	0,0%	0,0%	0,0%	0,0%	0,0%	0,0%	0,0%	Bacteria	Proteobacteria	Alphaproteobacteria			uncultured bacterium	-	-	-	-	-	-	-	-	-	-	-	-	-	-	-	-	-	-	-	-	-	-	-	-	-
New.ReferenceOTU1260	0,0%	0,0%	0,0%	0,0%	0,0%	0,0%	0,0%	0,0%	0,0%	Unassigned					Solanum lycopersicum (tomato)	-	-	-	-	-	-	-	-	-	-	-	-	-	-	-	-	-	-	-	-	-	-	-	-	-
New.ReferenceOTU1261	0,0%	0,0%	0,0%	0,0%	0,0%	0,0%	0,0%	0,0%	0,0%	Bacteria	GN02	BD1-5			uncultured bacterium	-	-	-	-	-	-	-	-	-	-	-	-	-	-	-	-	-	-	-	-	-	-	-	-	-
New.ReferenceOTU1262	0,0%	0,0%	0,0%	0,0%	0,0%	0,0%	0,0%	0,0%	0,0%	Bacteria	Proteobacteria	Deltaproteobacteria			Deferrisoma	-	-	-	-	-	-	-	-	-	-	-	-	-	-	-	-	-	-	-	-	-	-	-	-	-
New.ReferenceOTU1263	0,0%	0,0%	0,0%	0,0%	0,0%	0,0%	0,0%	0,0%	0,0%	Bacteria	Verrucomicrobia	Opitutae	Opitutales	Opitutaceae	Opitutus	-	napA	-	nirA	-	-	-	-	nosZ	-	-	nrfA	ureA	-	-	-	-	-	-	-	-	laccase_Basidio	-	-	-
New.ReferenceOTU1068	0,0%	0,0%	0,0%	0,0%	0,0%	0,0%	0,0%	0,0%	0,0%	Bacteria	WS3	PRR-12	Sediment-1	PRR-10	uncultured Latescibacteria bacterium	-	-	-	-	-	-	-	-	-	-	-	-	-	-	-	-	-	-	-	-	-	-	-	-	-
New.ReferenceOTU1066	0,0%	0,0%	0,0%	0,0%	0,0%	0,0%	0,0%	0,0%	0,0%	Bacteria	Proteobacteria	Deltaproteobacteria	MIZ46		uncultured proteobacterium	-	-	-	-	-	-	-	-	-	-	-	-	-	-	-	-	-	-	-	-	-	-	-	-	-
New.ReferenceOTU1064	0,0%	0,0%	0,0%	0,0%	0,0%	0,0%	0,0%	0,0%	0,0%	Unassigned					Thermotomaculum	-	-	-	-	-	-	-	-	-	-	-	-	-	-	-	-	-	-	-	-	-	-	-	-	-
New.ReferenceOTU1065	0,0%	0,0%	0,0%	0,0%	0,0%	0,0%	0,0%	0,0%	0,0%	Bacteria	Verrucomicrobia	Verrucomicrobiae	Verrucomicrobiales	Verrucomicrobiaceae	Verrucomicrobium	-	-	-	-	-	-	-	-	-	-	-	-	-	-	-	-	-	-	-	-	laccase_Asco	laccase_Basidio	-	-	ppo
New.ReferenceOTU1061	0,0%	0,0%	0,0%	0,0%	0,0%	0,0%	0,0%	0,0%	0,0%	Bacteria	Proteobacteria	Betaproteobacteria	Methylophilales	Methylophilaceae	uncultured	-	-	-	-	-	-	-	-	-	-	-	-	-	-	-	-	-	-	-	-	-	-	-	lip	-
New.ReferenceOTU1660	0,0%	0,0%	0,0%	0,0%	0,0%	0,0%	0,0%	0,0%	0,0%	Bacteria	Gemmatimonadetes	Gemmatimonadetes	Gemmatimonadales	A1-B1	Gemmatimonas	-	napA	-	-	-	-	nirK	norB	nosZ	-	-	nrfA	-	dsrA	dsrB	-	-	chb	-	-	-	-	-	-	-
New.ReferenceOTU1663	0,0%	0,0%	0,0%	0,0%	0,0%	0,0%	0,0%	0,0%	0,0%	Bacteria	Proteobacteria	Deltaproteobacteria	Myxococcales		Sandaracinus	-	napA	-	-	-	-	-	-	-	-	-	-	-	-	-	-	-	-	-	-	-	laccase_Basidio	-	-	ppo
New.ReferenceOTU1665	0,0%	0,0%	0,0%	0,0%	0,0%	0,0%	0,0%	0,0%	0,0%	Bacteria	Proteobacteria	Gammaproteobacteria	Legionellales	Legionellaceae	Legionella	-	-	-	-	-	nirS	-	norB	-	-	-	-	-	-	-	-	-	-	-	-	laccase_Asco	laccase_Basidio	-	-	ppo
New.ReferenceOTU1667	0,0%	0,0%	0,0%	0,0%	0,0%	0,0%	0,0%	0,0%	0,0%	Unassigned					uncultured bacterium	-	-	-	-	-	-	-	-	-	-	-	-	-	-	-	-	-	-	-	-	-	-	-	-	-
New.ReferenceOTU1669	0,0%	0,0%	0,0%	0,0%	0,0%	0,0%	0,0%	0,0%	0,0%	Bacteria	TM6	SJA-4			uncultured bacterium	-	-	-	-	-	-	-	-	-	-	-	-	-	-	-	-	-	-	-	-	-	-	-	-	-
New.ReferenceOTU679	0,0%	0,0%	0,0%	0,0%	0,0%	0,0%	0,0%	0,0%	0,1%	Bacteria	Proteobacteria	Deltaproteobacteria	Bdellovibrionales	Bdellovibrionaceae	Bdellovibrio	-	-	-	-	-	-	nirK	norB	-	-	-	nrfA	-	-	-	-	-	-	-	-	laccase_Asco	laccase_Basidio	-	-	ppo
New.ReferenceOTU674	0,0%	0,0%	0,0%	0,0%	0,0%	0,0%	0,0%	0,0%	0,0%	Bacteria	Proteobacteria	Alphaproteobacteria	Rhodospirillales	Rhodospirillaceae	Skermanella	-	napA	-	-	-	-	-	-	-	nifH	nifD	-	ureA	-	-	-	-	-	mmoX	-	laccase_Asco	laccase_Basidio	-	-	ppo
New.ReferenceOTU677	0,0%	0,0%	0,0%	0,0%	0,0%	0,0%	0,0%	0,0%	0,0%	Unassigned					Incertae Sedis	-	-	-	-	-	-	-	-	-	-	-	-	-	-	-	-	-	-	-	-	-	-	-	-	-
New.ReferenceOTU676	0,0%	0,0%	0,0%	0,0%	0,0%	0,0%	0,0%	0,0%	0,0%	Bacteria	Proteobacteria	Deltaproteobacteria	Myxococcales	Polyangiaceae	Byssovorax	-	-	-	-	-	-	-	-	-	-	-	-	-	-	-	-	-	-	-	-	-	-	-	-	-
New.ReferenceOTU670	0,0%	0,0%	0,0%	0,0%	0,0%	0,0%	0,0%	0,0%	0,0%	Bacteria	Planctomycetes	OM190	CL500-15		uncultured bacterium	-	-	-	-	-	-	-	-	-	-	-	-	-	-	-	-	-	-	-	-	-	-	-	-	-
New.ReferenceOTU673	0,0%	0,0%	0,0%	0,0%	0,0%	0,0%	0,0%	0,0%	0,0%	Bacteria	Proteobacteria	Alphaproteobacteria	BD7-3		uncultured bacterium	-	-	-	-	-	-	-	-	-	-	-	-	-	-	-	-	-	-	-	-	-	-	-	-	-
New.ReferenceOTU672	0,0%	0,0%	0,0%	0,0%	0,0%	0,0%	0,0%	0,0%	0,0%	Bacteria	Proteobacteria	Betaproteobacteria			uncultured	-	-	-	-	-	-	-	-	-	-	-	-	-	-	-	-	-	-	-	-	-	-	-	lip	-
New.ReferenceOTU587	0,0%	0,0%	0,0%	0,0%	0,0%	0,0%	0,0%	0,0%	0,0%	Bacteria	Actinobacteria	Thermoleophilia	Gaiellales	Gaiellaceae	uncultured bacterium	-	-	-	-	-	-	-	-	-	-	-	-	-	-	-	-	-	-	-	-	-	-	-	-	-
New.ReferenceOTU1409	0,0%	0,0%	0,0%	0,0%	0,0%	0,0%	0,0%	0,0%	0,0%	Bacteria	Proteobacteria	Betaproteobacteria	Burkholderiales	Oxalobacteraceae	Herminiimonas	-	napA	narG	-	-	-	nirK	norB	-	-	-	-	-	-	-	SoxB	-	-	-	-	laccase_Asco	laccase_Basidio	-	-	ppo
New.ReferenceOTU585	0,0%	0,0%	0,0%	0,0%	0,0%	0,0%	0,0%	0,0%	0,0%	Bacteria	Proteobacteria	Alphaproteobacteria	Rhodobacterales	Hyphomonadaceae	uncultured	-	-	-	-	-	-	-	-	-	-	-	-	-	-	-	-	-	-	-	-	-	-	-	lip	-
New.ReferenceOTU584	0,0%	0,0%	0,0%	0,0%	0,0%	0,0%	0,0%	0,0%	0,0%	Unassigned					Phaseolus acutifolius (tepary bean)	-	-	-	-	-	-	-	-	-	-	-	-	-	-	-	-	-	-	-	-	-	-	-	-	-
New.ReferenceOTU583	0,0%	0,0%	0,0%	0,0%	0,0%	0,0%	0,0%	0,0%	0,0%	Bacteria	Verrucomicrobia	[Pedosphaerae]	[Pedosphaerales]	R4-41B	uncultured bacterium	-	-	-	-	-	-	-	-	-	-	-	-	-	-	-	-	-	-	-	-	-	-	-	-	-
New.ReferenceOTU582	0,0%	0,0%	0,0%	0,0%	0,0%	0,0%	0,0%	0,0%	0,0%	Bacteria	Verrucomicrobia	[Spartobacteria]	[Chthoniobacterales]	[Chthoniobacteraceae]	uncultured Verrucomicrobia bacterium	-	-	-	-	-	-	-	-	-	-	-	-	-	-	-	-	-	-	-	-	-	-	-	-	-
New.ReferenceOTU581	0,0%	0,0%	0,0%	0,0%	0,0%	0,0%	0,0%	0,0%	0,0%	Bacteria	Proteobacteria	Betaproteobacteria	Methylophilales	Methylophilaceae	uncultured	-	-	-	-	-	-	-	-	-	-	-	-	-	-	-	-	-	-	-	-	-	-	-	lip	-
New.ReferenceOTU580	0,0%	0,0%	0,0%	0,0%	0,0%	0,0%	0,0%	0,0%	0,0%	Bacteria	Acidobacteria	Solibacteres	Solibacterales		Bryobacter	-	-	-	-	-	-	-	-	-	-	-	-	-	-	-	-	-	-	-	-	-	-	-	-	-
New.ReferenceOTU1401	0,0%	0,0%	0,0%	0,0%	0,0%	0,0%	0,0%	0,0%	0,0%	Bacteria	Firmicutes	Clostridia	Clostridiales	Peptostreptococcaceae	Incertae Sedis	-	-	-	-	-	-	-	-	-	-	-	-	-	-	-	-	-	-	-	-	-	-	-	-	-
New.ReferenceOTU145	0,0%	0,0%	0,0%	0,0%	0,0%	0,0%	0,0%	0,0%	0,0%	Bacteria	Proteobacteria	Gammaproteobacteria	Oceanospirillales		Kangiella	-	-	narG	-	-	nirS	nirK	norB	-	-	-	-	-	-	-	-	-	-	-	-	laccase_Asco	laccase_Basidio	-	-	-
New.ReferenceOTU144	0,0%	0,0%	0,0%	0,0%	0,0%	0,0%	0,0%	0,0%	0,0%	Bacteria	Planctomycetes	Planctomycetia	Gemmatales	Gemmataceae	uncultured	-	-	-	-	-	-	-	-	-	-	-	-	-	-	-	-	-	-	-	-	-	-	-	lip	-
New.ReferenceOTU143	0,0%	0,0%	0,0%	0,0%	0,0%	0,0%	0,0%	0,0%	0,0%	Unassigned					uncultured bacterium	-	-	-	-	-	-	-	-	-	-	-	-	-	-	-	-	-	-	-	-	-	-	-	-	-
New.ReferenceOTU414	0,0%	0,0%	0,0%	0,0%	0,0%	0,0%	0,0%	0,0%	0,0%	Unassigned					Spirochaeta	-	-	-	nirA	-	-	-	-	-	nifH	nifD	-	-	-	-	-	-	-	-	-	-	-	-	-	-
New.ReferenceOTU1314	0,0%	0,0%	0,0%	0,0%	0,0%	0,0%	0,0%	0,0%	0,0%	Bacteria	OD1	ZB2			uncultured bacterium	-	-	-	-	-	-	-	-	-	-	-	-	-	-	-	-	-	-	-	-	-	-	-	-	-
New.ReferenceOTU1315	0,0%	0,0%	0,0%	0,0%	0,0%	0,0%	0,0%	0,0%	0,0%	Bacteria	Proteobacteria	TA18	CV90		uncultured bacterium	-	-	-	-	-	-	-	-	-	-	-	-	-	-	-	-	-	-	-	-	-	-	-	-	-
New.ReferenceOTU1316	0,0%	0,0%	0,0%	0,0%	0,0%	0,0%	0,0%	0,0%	0,0%	Bacteria	Proteobacteria	Deltaproteobacteria	MIZ46		uncultured bacterium	-	-	-	-	-	-	-	-	-	-	-	-	-	-	-	-	-	-	-	-	-	-	-	-	-
New.ReferenceOTU1317	0,0%	0,0%	0,0%	0,0%	0,0%	0,0%	0,0%	0,0%	0,0%	Bacteria	Armatimonadetes	[Fimbriimonadia]	[Fimbriimonadales]	[Fimbriimonadaceae]	uncultured bacterium	-	-	-	-	-	-	-	-	-	-	-	-	-	-	-	-	-	-	-	-	-	-	-	-	-
New.ReferenceOTU1318	0,0%	0,0%	0,0%	0,0%	0,0%	0,0%	0,0%	0,0%	0,0%	Bacteria	Proteobacteria	Deltaproteobacteria			uncultured bacterium	-	-	-	-	-	-	-	-	-	-	-	-	-	-	-	-	-	-	-	-	-	-	-	-	-
New.ReferenceOTU457	0,0%	0,0%	0,0%	0,0%	0,0%	0,0%	0,0%	0,0%	0,0%	Bacteria	Planctomycetes	Planctomycetia	Gemmatales	Gemmataceae	Gemmata	-	-	-	-	-	-	-	-	-	-	-	-	-	-	-	-	-	-	-	-	-	-	-	-	-
New.ReferenceOTU108	0,0%	0,0%	0,0%	0,0%	0,0%	0,0%	0,0%	0,0%	0,0%	Bacteria	Proteobacteria	Gammaproteobacteria	Methylococcales	Methylococcaceae	Methylocaldum	-	-	-	-	-	-	-	-	-	nifH	-	-	-	-	-	-	-	-	mmoX	pmoA	-	-	-	-	-
New.ReferenceOTU451	0,0%	0,0%	0,0%	0,0%	0,0%	0,0%	0,0%	0,0%	0,0%	Bacteria	Cyanobacteria	4C0d-2	MLE1-12		uncultured bacterium	-	-	-	-	-	-	-	-	-	-	-	-	-	-	-	-	-	-	-	-	-	-	-	-	-
New.ReferenceOTU453	0,0%	0,0%	0,0%	0,0%	0,0%	0,0%	0,0%	0,0%	0,0%	Bacteria	Proteobacteria	TA18	PHOS-HD29		uncultured bacterium	-	-	-	-	-	-	-	-	-	-	-	-	-	-	-	-	-	-	-	-	-	-	-	-	-
New.ReferenceOTU452	0,0%	0,0%	0,0%	0,0%	0,0%	0,0%	0,0%	0,0%	0,1%	Bacteria	Proteobacteria	Gammaproteobacteria	Legionellales	Legionellaceae	Legionella	-	-	-	-	-	nirS	-	norB	-	-	-	-	-	-	-	-	-	-	-	-	laccase_Asco	laccase_Basidio	-	-	ppo
New.ReferenceOTU103	0,0%	0,0%	0,0%	0,0%	0,0%	0,0%	0,0%	0,0%	0,0%	Bacteria	Proteobacteria	Betaproteobacteria	Procabacteriales	Procabacteriaceae	uncultured	-	-	-	-	-	-	-	-	-	-	-	-	-	-	-	-	-	-	-	-	-	-	-	lip	-
New.ReferenceOTU101	0,0%	0,0%	0,0%	0,0%	0,0%	0,0%	0,0%	0,0%	0,0%	Bacteria	Proteobacteria	Alphaproteobacteria	BD7-3		uncultured bacterium	-	-	-	-	-	-	-	-	-	-	-	-	-	-	-	-	-	-	-	-	-	-	-	-	-
New.ReferenceOTU100	0,0%	0,0%	0,0%	0,0%	0,0%	0,0%	0,0%	0,0%	0,0%	Bacteria	Verrucomicrobia	Verrucomicrobiae	Verrucomicrobiales	Verrucomicrobiaceae	Verrucomicrobium	-	-	-	-	-	-	-	-	-	-	-	-	-	-	-	-	-	-	-	-	laccase_Asco	laccase_Basidio	-	-	ppo
New.ReferenceOTU459	0,0%	0,0%	0,0%	0,0%	0,0%	0,0%	0,0%	0,0%	0,0%	Bacteria	Verrucomicrobia	[Spartobacteria]	[Chthoniobacterales]	[Chthoniobacteraceae]	uncultured bacterium	-	-	-	-	-	-	-	-	-	-	-	-	-	-	-	-	-	-	-	-	-	-	-	-	-
New.ReferenceOTU458	0,0%	0,0%	0,0%	0,0%	0,0%	0,0%	0,0%	0,0%	0,0%	Unassigned					Geobacter	-	napA	narG	-	-	-	-	norB	-	nifH	nifD	nrfA	-	-	-	-	-	-	-	-	laccase_Asco	laccase_Basidio	-	-	ppo
New.ReferenceOTU105	0,0%	0,0%	0,0%	0,0%	0,0%	0,0%	0,0%	0,0%	0,0%	Bacteria	Proteobacteria	Deltaproteobacteria	Myxococcales	OM27	OM27 clade	-	-	-	-	-	-	-	-	-	-	-	-	-	-	-	-	-	-	-	-	-	-	-	-	-
New.ReferenceOTU1084	0,0%	0,0%	0,0%	0,0%	0,0%	0,0%	0,0%	0,0%	0,0%	Bacteria	Proteobacteria	Gammaproteobacteria	Legionellales	Coxiellaceae	Aquicella	-	-	-	-	-	-	-	-	-	-	-	-	-	-	-	-	-	-	-	-	-	-	-	-	-
New.ReferenceOTU298	0,0%	0,0%	0,0%	0,0%	0,0%	0,0%	0,0%	0,0%	0,0%	Bacteria	Verrucomicrobia	Opitutae	Opitutales	Opitutaceae	Opitutus	-	napA	-	nirA	-	-	-	-	nosZ	-	-	nrfA	ureA	-	-	-	-	-	-	-	-	laccase_Basidio	-	-	-
New.ReferenceOTU1080	0,0%	0,0%	0,0%	0,0%	0,0%	0,0%	0,0%	0,0%	0,0%	Unassigned					uncultured	-	-	-	-	-	-	-	-	-	-	-	-	-	-	-	-	-	-	-	-	-	-	-	lip	-
New.ReferenceOTU1081	0,0%	0,0%	0,0%	0,0%	0,0%	0,0%	0,0%	0,0%	0,0%	Bacteria	Proteobacteria	Deltaproteobacteria	Myxococcales	Myxococcaceae	Anaeromyxobacter	-	napA	narG	nirA	-	nirS	-	norB	nosZ	nifH	nifD	nrfA	ureA	-	-	SoxB	-	-	-	-	-	laccase_Basidio	-	-	-
New.ReferenceOTU1083	0,0%	0,0%	0,0%	0,0%	0,0%	0,0%	0,0%	0,0%	0,0%	Unassigned					Buchnera	-	-	-	-	-	-	-	-	-	-	-	-	-	-	-	-	-	-	-	-	-	-	-	-	-
New.ReferenceOTU293	0,0%	0,0%	0,0%	0,0%	0,0%	0,0%	0,0%	0,0%	0,0%	Bacteria	Proteobacteria	Betaproteobacteria	Burkholderiales	Burkholderiaceae	uncultured bacterium	-	-	-	-	-	-	-	-	-	-	-	-	-	-	-	-	-	-	-	-	-	-	-	-	-

New.ReferenceOTU292	0,0%	0,0%	0,0%	0,0%	0,0%	0,0%	0,0%	0,0%	0,0%	Bacteria	Proteobacteria	Deltaproteobacteria	Myxococcales	Myxococcaceae	Anaeromyxobacter	-	napA	narG	nirA	-	nirS	-	norB	nosZ	nifH	nifD	nrfA	ureA	-	-	SoxB	-	-	-	-	-	laccase_Basidio	-	-	-
New.ReferenceOTU291	0,0%	0,0%	0,0%	0,0%	0,0%	0,0%	0,0%	0,0%	0,0%	Unassigned					uncultured	-	-	-	-	-	-	-	-	-	-	-	-	-	-	-	-	-	-	-	-	-	-	-	lip	-
New.ReferenceOTU290	0,0%	0,0%	0,0%	0,0%	0,0%	0,0%	0,0%	0,0%	0,0%	Bacteria	Proteobacteria	Alphaproteobacteria	Caulobacterales	Caulobacteraceae	Phenylobacterium	-	napA	narG	-	-	-	nirK	norB	-	-	-	-	-	-	-	-	-	-	-	-	laccase_Asco	laccase_Basidio	-	-	ppo
New.ReferenceOTU297	0,0%	0,0%	0,0%	0,0%	0,0%	0,0%	0,0%	0,0%	0,0%	Bacteria	Armatimonadetes	[Fimbriimonadia]	[Fimbriimonadales]	[Fimbriimonadaceae]	uncultured Armatimonadetes bacterium	-	-	-	-	-	-	-	-	-	-	-	-	-	-	-	-	-	-	-	-	-	-	-	-	-
New.ReferenceOTU296	0,0%	0,0%	0,0%	0,0%	0,0%	0,0%	0,0%	0,0%	0,0%	Bacteria	Proteobacteria	Alphaproteobacteria	Rhizobiales		uncultured bacterium	-	-	-	-	-	-	-	-	-	-	-	-	-	-	-	-	-	-	-	-	-	-	-	-	-
New.ReferenceOTU295	0,0%	0,0%	0,0%	0,0%	0,0%	0,0%	0,0%	0,0%	0,0%	Bacteria	Proteobacteria	Deltaproteobacteria	Myxococcales		uncultured bacterium	-	-	-	-	-	-	-	-	-	-	-	-	-	-	-	-	-	-	-	-	-	-	-	-	-
New.ReferenceOTU294	0,0%	0,0%	0,0%	0,0%	0,0%	0,0%	0,0%	0,0%	0,0%	Bacteria	Proteobacteria	Gammaproteobacteria	Methylococcales		Methylovulum	-	-	-	-	-	-	-	-	-	nifH	-	-	-	-	-	-	-	-	mmoX	pmoA	-	-	-	-	-
New.ReferenceOTU850	0,0%	0,0%	0,0%	0,0%	0,0%	0,0%	0,0%	0,0%	0,0%	Bacteria	Proteobacteria	Alphaproteobacteria	Caulobacterales	Caulobacteraceae	Caulobacter	-	napA	narG	nirA	-	-	nirK	norB	-	-	-	-	-	-	-	-	-	chb	-	-	laccase_Asco	laccase_Basidio	-	-	ppo
New.ReferenceOTU853	0,0%	0,0%	0,0%	0,0%	0,0%	0,0%	0,0%	0,0%	0,0%	Bacteria	Proteobacteria	Alphaproteobacteria	BD7-3		uncultured bacterium	-	-	-	-	-	-	-	-	-	-	-	-	-	-	-	-	-	-	-	-	-	-	-	-	-
New.ReferenceOTU852	0,0%	0,0%	0,0%	0,0%	0,0%	0,0%	0,0%	0,0%	0,0%	Bacteria	Proteobacteria	Betaproteobacteria	Burkholderiales	Comamonadaceae	Leptothrix	-	napA	-	-	-	nirS	-	norB	nosZ	nifH	nifD	-	ureA	-	-	-	-	-	mmoX	-	-	-	-	-	-
New.ReferenceOTU855	0,0%	0,0%	0,0%	0,0%	0,0%	0,0%	0,0%	0,0%	0,0%	Bacteria	Proteobacteria	Alphaproteobacteria	Rhizobiales		uncultured Brucella sp.	-	-	-	-	-	-	-	-	-	-	-	-	-	-	-	-	-	-	-	-	-	-	-	-	-
New.ReferenceOTU854	0,0%	0,0%	0,0%	0,0%	0,0%	0,0%	0,0%	0,0%	0,0%	Bacteria	Proteobacteria	Alphaproteobacteria	Rhodospirillales	Rhodospirillaceae	Elstera	-	-	-	-	-	-	-	-	-	-	-	-	-	-	-	-	-	-	-	-	-	-	-	-	-
New.ReferenceOTU857	0,0%	0,0%	0,0%	0,0%	0,0%	0,0%	0,0%	0,0%	0,0%	Bacteria	Proteobacteria	Deltaproteobacteria	Myxococcales	OM27	OM27 clade	-	-	-	-	-	-	-	-	-	-	-	-	-	-	-	-	-	-	-	-	-	-	-	-	-
New.ReferenceOTU856	0,0%	0,0%	0,0%	0,0%	0,0%	0,0%	0,0%	0,0%	0,0%	Bacteria	Proteobacteria	Deltaproteobacteria	Myxococcales		Sorangium	-	napA	-	-	-	-	nirK	norB	-	-	-	nrfA	ureA	-	-	-	-	chb	-	-	laccase_Asco	laccase_Basidio	-	-	ppo
New.ReferenceOTU859	0,0%	0,0%	0,0%	0,0%	0,0%	0,0%	0,0%	0,0%	0,0%	Bacteria	Proteobacteria	Betaproteobacteria	Burkholderiales		Leeia	-	-	-	-	-	-	-	-	-	-	-	-	-	-	-	-	-	-	-	-	-	-	-	-	-
New.ReferenceOTU858	0,0%	0,0%	0,0%	0,0%	0,0%	0,0%	0,0%	0,0%	0,0%	Bacteria	Actinobacteria	Actinobacteria	Actinomycetales	Sporichthyaceae	Jatrophihabitans	-	-	-	-	-	-	-	-	-	-	-	-	-	-	-	-	-	-	-	-	-	-	-	-	-
New.ReferenceOTU155	0,0%	0,0%	0,0%	0,0%	0,0%	0,0%	0,0%	0,0%	0,0%	Unassigned					uncultured organism	-	-	-	-	-	-	-	-	-	-	-	-	-	-	-	-	-	-	-	-	-	-	-	-	-
New.ReferenceOTU925	0,0%	0,0%	0,0%	0,0%	0,0%	0,0%	0,0%	0,0%	0,0%	Bacteria	Proteobacteria	Deltaproteobacteria	Myxococcales	Cystobacterineae	uncultured bacterium	-	-	-	-	-	-	-	-	-	-	-	-	-	-	-	-	-	-	-	-	-	-	-	-	-
New.ReferenceOTU924	0,0%	0,0%	0,0%	0,0%	0,0%	0,0%	0,0%	0,0%	0,0%	Bacteria	Proteobacteria	Gammaproteobacteria	Aeromonadales	Aeromonadaceae	Aeromonas	-	napA	narG	-	-	-	-	-	nosZ	nifH	-	nrfA	ureA	-	dsrB	-	-	chb	-	-	laccase_Asco	laccase_Basidio	-	-	ppo
New.ReferenceOTU926	0,0%	0,0%	0,0%	0,0%	0,0%	0,0%	0,0%	0,0%	0,0%	Unassigned					uncultured bacterium	-	-	-	-	-	-	-	-	-	-	-	-	-	-	-	-	-	-	-	-	-	-	-	-	-
New.ReferenceOTU921	0,0%	0,0%	0,0%	0,0%	0,0%	0,0%	0,0%	0,0%	0,0%	Bacteria	Chloroflexi	Anaerolineae	SBR1031	A4b	uncultured	-	-	-	-	-	-	-	-	-	-	-	-	-	-	-	-	-	-	-	-	-	-	-	lip	-
New.ReferenceOTU923	0,0%	0,0%	0,0%	0,0%	0,0%	0,0%	0,0%	0,0%	0,0%	Bacteria	Chlamydiae	Chlamydiia	Chlamydiales	Parachlamydiaceae	Candidatus Protochlamydia	-	-	-	-	-	-	-	-	-	-	-	-	-	-	-	-	-	-	-	-	-	-	-	-	-
New.ReferenceOTU922	0,0%	0,0%	0,0%	0,0%	0,0%	0,0%	0,0%	0,0%	0,1%	Bacteria	Actinobacteria	Actinobacteria	Actinomycetales	Mycobacteriaceae	Mycobacterium	-	napA	narG	nirA	-	-	-	norB	-	-	-	-	ureA	-	-	-	amoA_AOB	-	mmoX	pmoA	laccase_Asco	laccase_Basidio	-	-	ppo
New.ReferenceOTU928	0,0%	0,0%	0,0%	0,0%	0,0%	0,0%	0,0%	0,0%	0,0%	Unassigned					Rhizomicrobium	-	-	-	-	-	-	-	-	-	-	-	-	-	-	-	-	-	-	-	-	-	-	-	-	-
New.ReferenceOTU615	0,0%	0,0%	0,0%	0,0%	0,0%	0,0%	0,0%	0,0%	0,0%	Bacteria	Planctomycetes	Planctomycetia	Gemmatales	Gemmataceae	Gemmata	-	-	-	-	-	-	-	-	-	-	-	-	-	-	-	-	-	-	-	-	-	-	-	-	-
New.ReferenceOTU614	0,0%	0,0%	0,0%	0,0%	0,0%	0,0%	0,0%	0,0%	0,0%	Unassigned					Anderseniella	-	-	-	-	-	-	-	-	-	-	-	-	-	-	-	-	-	-	-	-	-	-	-	-	-
New.ReferenceOTU619	0,0%	0,0%	0,0%	0,0%	0,0%	0,0%	0,0%	0,0%	0,0%	Bacteria	Proteobacteria	Betaproteobacteria	Methylophilales	Methylophilaceae	LD28 freshwater group	-	-	-	-	-	-	-	-	-	-	-	-	-	-	-	-	-	-	-	-	-	-	-	-	-
New.ReferenceOTU1245	0,0%	0,0%	0,0%	0,0%	0,0%	0,0%	0,0%	0,0%	0,0%	Bacteria	Proteobacteria	Deltaproteobacteria	Bdellovibrionales	Bacteriovoracaceae	Peredibacter	-	-	-	-	-	-	-	-	-	-	-	-	-	-	-	-	-	-	-	-	-	-	-	-	-
New.ReferenceOTU662	0,0%	0,0%	0,0%	0,0%	0,0%	0,0%	0,0%	0,0%	0,0%	Bacteria	Planctomycetes	vadinHA49	DH61		uncultured bacterium	-	-	-	-	-	-	-	-	-	-	-	-	-	-	-	-	-	-	-	-	-	-	-	-	-
New.ReferenceOTU660	0,0%	0,0%	0,0%	0,0%	0,0%	0,0%	0,0%	0,0%	0,0%	Bacteria	Proteobacteria	Alphaproteobacteria	Rickettsiales		Candidatus Odyssella	-	-	-	-	-	-	-	-	-	-	-	-	-	-	-	-	-	-	-	-	-	-	-	-	-
New.ReferenceOTU667	0,0%	0,0%	0,0%	0,0%	0,0%	0,0%	0,0%	0,0%	0,0%	Bacteria	Proteobacteria	Betaproteobacteria	Burkholderiales	Comamonadaceae	Simplicispira	-	-	-	-	-	nirS	-	-	-	-	-	-	-	-	-	-	-	-	-	-	-	-	-	-	-
New.ReferenceOTU665	0,0%	0,0%	0,0%	0,0%	0,0%	0,0%	0,0%	0,0%	0,0%	Bacteria	Proteobacteria	Gammaproteobacteria	Methylococcales	Crenotrichaceae	Crenothrix	-	-	-	-	-	-	-	-	-	-	-	-	-	-	-	-	-	-	-	pmoA	-	-	-	-	-
New.ReferenceOTU1276	0,0%	0,0%	0,0%	0,0%	0,0%	0,0%	0,0%	0,0%	0,0%	Bacteria	Proteobacteria	Gammaproteobacteria	Methylococcales	Methylococcaceae	Methylomonas	-	napA	-	nirA	-	nirS	nirK	norB	-	nifH	nifD	-	ureA	-	-	-	amoA_AOB	-	mmoX	pmoA	laccase_Asco	laccase_Basidio	-	-	ppo
New.ReferenceOTU1275	0,0%	0,0%	0,0%	0,0%	0,0%	0,0%	0,0%	0,0%	0,0%	Bacteria	Proteobacteria	Betaproteobacteria	Burkholderiales	Comamonadaceae	Variovorax	-	napA	narG	nirA	-	-	-	norB	-	-	-	-	ureA	-	-	-	-	-	mmoX	-	-	-	-	-	-
New.ReferenceOTU669	0,0%	0,0%	0,0%	0,0%	0,0%	0,0%	0,0%	0,0%	0,0%	Bacteria	Proteobacteria	Deltaproteobacteria			uncultured bacterium	-	-	-	-	-	-	-	-	-	-	-	-	-	-	-	-	-	-	-	-	-	-	-	-	-
New.ReferenceOTU1272	0,0%	0,0%	0,0%	0,0%	0,0%	0,0%	0,0%	0,0%	0,0%	Unassigned					Bdellovibrio	-	-	-	-	-	-	nirK	norB	-	-	-	nrfA	-	-	-	-	-	-	-	-	laccase_Asco	laccase_Basidio	-	-	ppo
New.ReferenceOTU1271	0,0%	0,0%	0,0%	0,0%	0,0%	0,0%	0,0%	0,0%	0,0%	Bacteria	Proteobacteria	Deltaproteobacteria	MIZ46		uncultured bacterium	-	-	-	-	-	-	-	-	-	-	-	-	-	-	-	-	-	-	-	-	-	-	-	-	-
New.ReferenceOTU1071	0,0%	0,0%	0,0%	0,0%	0,0%	0,0%	0,0%	0,0%	0,0%	Bacteria	Proteobacteria	Alphaproteobacteria	Caulobacterales	Caulobacteraceae	Caulobacter	-	napA	narG	nirA	-	-	nirK	norB	-	-	-	-	-	-	-	-	-	chb	-	-	laccase_Asco	laccase_Basidio	-	-	ppo
New.ReferenceOTU1075	0,0%	0,0%	0,0%	0,0%	0,0%	0,0%	0,0%	0,0%	0,0%	Bacteria	Proteobacteria	Deltaproteobacteria	Myxococcales	Polyangiaceae	Byssovorax	-	-	-	-	-	-	-	-	-	-	-	-	-	-	-	-	-	-	-	-	-	-	-	-	-
New.ReferenceOTU1077	0,0%	0,0%	0,0%	0,0%	0,0%	0,0%	0,0%	0,0%	0,0%	Bacteria	Proteobacteria	Deltaproteobacteria	Sva0853	JTB36	uncultured bacterium	-	-	-	-	-	-	-	-	-	-	-	-	-	-	-	-	-	-	-	-	-	-	-	-	-
New.ReferenceOTU1076	0,0%	0,0%	0,0%	0,0%	0,0%	0,0%	0,0%	0,0%	0,0%	Unassigned					uncultured organism	-	-	-	-	-	-	-	-	-	-	-	-	-	-	-	-	-	-	-	-	-	-	-	-	-
New.ReferenceOTU1676	0,0%	0,0%	0,0%	0,0%	0,0%	0,0%	0,0%	0,0%	0,0%	Bacteria	Proteobacteria	Gammaproteobacteria	Pseudomonadales	Moraxellaceae	Perlucidibaca	-	-	-	-	-	-	-	-	-	-	-	-	-	-	-	-	-	-	-	-	-	-	-	-	-
New.ReferenceOTU1674	0,0%	0,0%	0,0%	0,0%	0,0%	0,0%	0,0%	0,0%	0,0%	Bacteria	Proteobacteria	Alphaproteobacteria	Rhodospirillales	Acetobacteraceae	Acidocella	-	napA	narG	nirA	-	-	-	-	-	-	-	-	ureA	-	-	-	-	-	-	-	laccase_Asco	laccase_Basidio	-	-	ppo
New.ReferenceOTU1679	0,0%	0,0%	0,0%	0,0%	0,0%	0,0%	0,0%	0,0%	0,0%	Bacteria	Proteobacteria	Betaproteobacteria	Burkholderiales		Azospira	-	-	narG	-	-	-	nirK	-	-	nifH	-	-	-	-	-	-	-	-	-	-	-	-	-	-	-
New.ReferenceOTU595	0,0%	0,0%	0,0%	0,0%	0,0%	0,0%	0,0%	0,0%	0,0%	Bacteria	Verrucomicrobia	Opitutae	Opitutales	Opitutaceae	Opitutus	-	napA	-	nirA	-	-	-	-	nosZ	-	-	nrfA	ureA	-	-	-	-	-	-	-	-	laccase_Basidio	-	-	-
New.ReferenceOTU1419	0,0%	0,0%	0,0%	0,0%	0,0%	0,0%	0,0%	0,0%	0,0%	Bacteria	Armatimonadetes	[Fimbriimonadia]	[Fimbriimonadales]	[Fimbriimonadaceae]	uncultured bacterium	-	-	-	-	-	-	-	-	-	-	-	-	-	-	-	-	-	-	-	-	-	-	-	-	-
New.ReferenceOTU597	0,0%	0,0%	0,0%	0,0%	0,0%	0,0%	0,0%	0,0%	0,0%	Bacteria	Proteobacteria	Deltaproteobacteria	Spirobacillales		uncultured bacterium	-	-	-	-	-	-	-	-	-	-	-	-	-	-	-	-	-	-	-	-	-	-	-	-	-
New.ReferenceOTU590	0,0%	0,0%	0,0%	0,0%	0,0%	0,0%	0,0%	0,0%	0,0%	Bacteria	Proteobacteria	Gammaproteobacteria	Xanthomonadales	Sinobacteraceae	uncultured	-	-	-	-	-	-	-	-	-	-	-	-	-	-	-	-	-	-	-	-	-	-	-	lip	-
New.ReferenceOTU591	0,0%	0,0%	0,0%	0,0%	0,0%	0,0%	0,0%	0,0%	0,0%	Unassigned					uncultured bacterium	-	-	-	-	-	-	-	-	-	-	-	-	-	-	-	-	-	-	-	-	-	-	-	-	-
New.ReferenceOTU592	0,0%	0,0%	0,0%	0,0%	0,0%	0,0%	0,0%	0,0%	0,0%	Unassigned					Staphylococcus	-	napA	narG	-	-	-	nirK	norB	-	-	-	-	ureA	-	-	-	-	-	-	-	laccase_Asco	laccase_Basidio	-	-	-
New.ReferenceOTU593	0,0%	0,0%	0,0%	0,0%	0,0%	0,0%	0,0%	0,0%	0,0%	Bacteria	Verrucomicrobia	Opitutae	Opitutales	Opitutaceae	Opitutus	-	napA	-	nirA	-	-	-	-	nosZ	-	-	nrfA	ureA	-	-	-	-	-	-	-	-	laccase_Basidio	-	-	-
New.ReferenceOTU598	0,0%	0,0%	0,0%	0,0%	0,0%	0,0%	0,0%	0,0%	0,0%	Bacteria	Bacteroidetes	Cytophagia	Cytophagales	Cytophagaceae	Sporocytophaga	cellulolytic	-	-	-	-	-	-	-	-	-	-	-	-	-	-	-	-	-	-	-	-	-	-	-	-
New.ReferenceOTU599	0,0%	0,0%	0,0%	0,0%	0,0%	0,0%	0,0%	0,0%	0,0%	Bacteria	Proteobacteria	Gammaproteobacteria			Gallaecimonas	-	-	-	-	-	-	-	-	-	-	-	-	-	-	-	-	-	chb	-	-	-	-	-	-	-
New.ReferenceOTU1415	0,0%	0,0%	0,0%	0,0%	0,0%	0,0%	0,0%	0,0%	0,0%	Bacteria	Proteobacteria	Betaproteobacteria	Burkholderiales	Comamonadaceae	Roseateles	-	-	-	-	-	-	-	-	-	nifH	-	-	-	-	-	-	-	-	-	-	-	-	-	-	-
New.ReferenceOTU1306	0,0%	0,0%	0,0%	0,0%	0,0%	0,0%	0,0%	0,0%	0,0%	Bacteria	Verrucomicrobia	[Pedosphaerae]	[Pedosphaerales]		uncultured bacterium	-	-	-	-	-	-	-	-	-	-	-	-	-	-	-	-	-	-	-	-	-	-	-	-	-
New.ReferenceOTU1304	0,0%	0,0%	0,0%	0,0%	0,0%	0,0%	0,0%	0,0%	0,0%	Bacteria	Chlamydiae	Chlamydiia			Neochlamydia	-	-	-	-	-	-	-	-	-	-	-	-	-	-	-	-	-	-	-	-	-	-	-	-	-
New.ReferenceOTU1302	0,0%	0,0%	0,0%	0,0%	0,0%	0,0%	0,0%	0,0%	0,0%	Bacteria	Proteobacteria	Deltaproteobacteria	Myxococcales		uncultured bacterium	-	-	-	-	-	-	-	-	-	-	-	-	-	-	-	-	-	-	-	-	-	-	-	-	-
New.ReferenceOTU1301	0,0%	0,0%	0,0%	0,0%	0,0%	0,0%	0,0%	0,0%	0,0%	Unassigned					Pleomorphomonas	-	-	-	-	-	-	-	-	-	nifH	-	-	-	-	-	-	-	-	-	-	-	-	-	-	-
New.ReferenceOTU1309	0,0%	0,0%	0,0%	0,0%	0,0%	0,0%	0,0%	0,0%	0,0%	Bacteria	Planctomycetes	Planctomycetia	Gemmatales	Gemmataceae	Gemmata	-	-	-	-	-	-	-	-	-	-	-	-	-	-	-	-	-	-	-	-	-	-	-	-	-
New.ReferenceOTU118	0,0%	0,0%	0,0%	0,0%	0,0%	0,0%	0,0%	0,0%	0,0%	Bacteria	Bacteroidetes	[Saprospirae]	[Saprospirales]	Saprospiraceae	uncultured	-	-	-	-	-	-	-	-	-	-	-	-	-	-	-	-	-	-	-	-	-	-	-	lip	-
New.ReferenceOTU119	0,0%	0,0%	0,0%	0,0%	0,0%	0,0%	0,0%	0,0%	0,0%	Unassigned					uncultured bacterium	-	-	-	-	-	-	-	-	-	-	-	-	-	-	-	-	-	-	-	-	-	-	-	-	-
New.ReferenceOTU449	0,0%	0,0%	0,0%	0,0%	0,0%	0,0%	0,0%	0,0%	0,0%	Bacteria	Planctomycetes	vadinHA49	DH61		uncultured bacterium	-	-	-	-	-	-	-	-	-	-	-	-	-	-	-	-	-	-	-	-	-	-	-	-	-
New.ReferenceOTU110	0,0%	0,0%	0,0%	0,0%	0,0%	0,0%	0,0%	0,0%	0,0%	Unassigned					uncultured	-	-	-	-	-	-	-	-	-	-	-	-	-	-	-	-	-	-	-	-	-	-	-	lip	-
New.ReferenceOTU114	0,0%	0,0%	0,0%	0,0%	0,0%	0,0%	0,0%	0,0%	0,0%	Bacteria	Proteobacteria	Alphaproteobacteria			uncultured alpha proteobacterium	-	-	-	-	-	-	-	-	-	-	-	-	-	-	-	-	-	-	-	-	-	-	-	-	-
New.ReferenceOTU115	0,0%	0,0%	0,0%	0,0%	0,0%	0,0%	0,0%	0,0%	0,0%	Bacteria	Verrucomicrobia	[Pedosphaerae]	[Pedosphaerales]		uncultured bacterium	-	-	-	-	-	-	-	-	-	-	-	-	-	-	-	-	-	-	-	-	-	-	-	-	-
New.ReferenceOTU116	0,0%	0,0%	0,0%	0,0%	0,0%	0,0%	0,0%	0,0%	0,0%	Bacteria	Proteobacteria	Alphaproteobacteria	Rhizobiales		Meganema	-	-	-	-	-	-	-	-	-	-	-	-	-	-	-	-	-	-	-	-	-	-	-	-	-
New.ReferenceOTU117	0,0%	0,0%	0,0%	0,0%	0,0%	0,0%	0,0%	0,0%	0,0%	Bacteria	Planctomycetes	Planctomycetia	Gemmatales	Gemmataceae	Gemmata	-	-	-	-	-	-	-	-	-	-	-	-	-	-	-	-	-	-	-	-	-	-	-	-	-
New.ReferenceOTU288	0,0%	0,0%	0,0%	0,0%	0,0%	0,0%	0,0%	0,0%	0,0%	Unassigned					uncultured bacterium	-	-	-	-	-	-	-	-	-	-	-	-	-	-	-	-	-	-	-	-	-	-	-	-	-
New.ReferenceOTU1094	0,0%	0,0%	0,0%	0,0%	0,0%	0,0%	0,0%	0,0%	0,0%	Bacteria	Proteobacteria	Alphaproteobacteria	Caulobacterales	Caulobacteraceae	Asticcacaulis	-	napA	narG	nirA	-	-	-	-	-	-	-	-	-	-	-	-	-	chb	-	-	laccase_Asco	laccase_Basidio	-	-	ppo
New.ReferenceOTU1093	0,0%	0,0%	0,0%	0,0%	0,0%	0,0%	0,0%	0,0%	0,0%	Bacteria	Proteobacteria	Betaproteobacteria	Burkholderiales	Comamonadaceae	uncultured	-	-	-	-	-	-	-	-	-	-	-	-	-	-	-	-	-	-	-	-	-	-	-	lip	-
New.ReferenceOTU282	0,0%	0,0%	0,0%	0,0%	0,0%	0,0%	0,0%	0,0%	0,0%	Bacteria	Proteobacteria	TA18	CV90		uncultured bacterium	-	-	-	-	-	-	-	-	-	-	-	-	-	-	-	-	-	-	-	-	-	-	-	-	-
New.ReferenceOTU283	0,0%	0,0%	0,0%	0,0%	0,0%	0,0%	0,0%	0,0%	0,0%	Bacteria	Proteobacteria	Alphaproteobacteria	Rickettsiales	Rickettsiaceae	uncultured	-	-	-	-	-	-	-	-	-	-	-	-	-	-	-	-	-	-	-	-	-	-	-	lip	-
New.ReferenceOTU285	0,0%	0,0%	0,0%	0,0%	0,0%	0,0%	0,0%	0,0%	0,0%	Bacteria	Proteobacteria	Betaproteobacteria	Burkholderiales	Comamonadaceae	Variovorax	-	napA	narG	nirA	-	-	-	norB	-	-	-	-	ureA	-	-	-	-	-	mmoX	-	-	-	-	-	-
New.ReferenceOTU1099	0,0%	0,0%	0,0%	0,0%	0,0%	0,0%	0,0%	0,0%	0,0%	Bacteria	Proteobacteria	Alphaproteobacteria	Rhizobiales	Methylocystaceae	uncultured Rhizobiales bacterium	-	-	-	-	-	-	-	-	-	-	-	-	-	-	-	-	-	-	-	-	-	-	-	-	-
New.ReferenceOTU846	0,0%	0,0%	0,0%	0,0%	0,0%	0,0%	0,0%	0,0%	0,0%	Unassigned					uncultured organism	-	-	-	-	-	-	-	-	-	-	-	-	-	-	-	-	-	-	-	-	-	-	-	-	-
New.ReferenceOTU847	0,0%	0,0%	0,0%	0,0%	0,0%	0,0%	0,0%	0,0%	0,0%	Bacteria	Proteobacteria	Deltaproteobacteria	Myxococcales		uncultured bacterium	-	-	-	-	-	-	-	-	-	-	-	-	-	-	-	-	-	-	-	-	-	-	-	-	-
New.ReferenceOTU845	0,0%	0,0%	0,0%	0,0%	0,0%	0,0%	0,0%	0,0%	0,0%	Bacteria	Proteobacteria	Gammaproteobacteria	Xanthomonadales	Sinobacteraceae	uncultured bacterium	-	-	-	-	-	-	-	-	-	-	-	-	-	-	-	-	-	-	-	-	-	-	-	-	-
New.ReferenceOTU842	0,0%	0,0%	0,0%	0,0%	0,0%	0,0%	0,0%	0,0%	0,0%	Bacteria	Chlamydiae	Chlamydiia	Chlamydiales	Rhabdochlamydiaceae	Candidatus Rhabdochlamydia	-	-	-	-	-	-	-	-	-	-	-	-	-	-	-	-	-	-	-	-	-	-	-	-	-
New.ReferenceOTU843	0,0%	0,0%	0,0%	0,0%	0,0%	0,0%	0,0%	0,0%	0,0%	Bacteria	Proteobacteria	Alphaproteobacteria	Rhizobiales	Beijerinckiaceae	Meganema	-	-	-	-	-	-	-	-	-	-	-	-	-	-	-	-	-	-	-	-	-	-	-	-	-
New.ReferenceOTU840	0,0%	0,0%	0,0%	0,0%	0,0%	0,0%	0,0%	0,0%	0,0%	Unassigned					uncultured Verrucomicrobia bacterium	-	-	-	-	-	-	-	-	-	-	-	-	-	-	-	-	-	-	-	-	-	-	-	-	-
New.ReferenceOTU841	0,0%	0,0%	0,0%	0,0%	0,0%	0,0%	0,0%	0,0%	0,0%	Bacteria	Planctomycetes	Planctomycetia	Gemmatales	Gemmataceae	Gemmata	-	-	-	-	-	-	-	-	-	-	-	-	-	-	-	-	-	-	-	-	-	-	-	-	-
New.ReferenceOTU1542	0,0%	0,0%	0,0%	0,0%	0,0%	0,0%	0,0%	0,0%	0,0%	Bacteria	Planctomycetes	Planctomycetia	Gemmatales	Gemmataceae	uncultured	-	-	-	-	-	-	-	-	-	-	-	-	-	-	-	-	-	-	-	-	-	-	-	lip	-
New.ReferenceOTU849	0,0%	0,0%	0,0%	0,0%	0,0%	0,0%	0,0%	0,0%	0,0%	Bacteria	Planctomycetes	Planctomycetia	Gemmatales	Gemmataceae	Zavarzinella	-	-	-	-	-	-	-	-	-	-	-	-	-	-	-	-	-	-	-	-	-	-	-	-	-
New.ReferenceOTU932	0,0%	0,0%	0,0%	0,0%	0,0%	0,0%	0,0%	0,0%	0,0%	Unassigned					uncultured bacterium	-	-	-	-	-	-	-	-	-	-	-	-	-	-	-	-	-	-	-	-	-	-	-	-	-
New.ReferenceOTU933	0,0%	0,0%	0,0%	0,0%	0,0%	0,0%	0,0%	0,0%	0,0%	Unassigned					Anderseniella	-	-	-	-	-	-	-	-	-	-	-	-	-	-	-	-	-	-	-	-	-	-	-	-	-
New.ReferenceOTU930	0,0%	0,0%	0,0%	0,0%	0,0%	0,0%	0,0%	0,0%	0,0%	Bacteria	Proteobacteria	Betaproteobacteria	Burkholderiales	Oxalobacteraceae	Herbaspirillum	-	napA	narG	-	-	nirS	nirK	norB	nosZ	nifH	nifD	-	ureA	-	-	-	-	-	-	-	-	laccase_Basidio	-	-	-
New.ReferenceOTU931	0,0%	0,0%	0,0%	0,0%	0,0%	0,0%	0,0%	0,0%	0,0%	Bacteria	Proteobacteria	Alphaproteobacteria			uncultured	-	-	-	-	-	-	-	-	-	-	-	-	-	-	-	-	-	-	-	-	-	-	-	lip	-
New.ReferenceOTU936	0,0%	0,0%	0,0%	0,0%	0,0%	0,0%	0,0%	0,0%	0,0%	Bacteria	Proteobacteria	Deltaproteobacteria	Bdellovibrionales	Bdellovibrionaceae	Bdellovibrio	-	-	-	-	-	-	nirK	norB	-	-	-	nrfA	-	-	-	-	-	-	-	-	laccase_Asco	laccase_Basidio	-	-	ppo
New.ReferenceOTU1497	0,0%	0,0%	0,0%	0,0%	0,0%	0,0%	0,0%	0,0%	0,0%	Unassigned					uncultured phototrophic eukaryote	-	-	-	-	-	-	-	-	-	-	-	-	-	-	-	-	-	-	-	-	-	-	-	-	-
New.ReferenceOTU686	0,0%	0,0%	0,0%	0,0%	0,0%	0,0%	0,0%	0,0%	0,0%	Bacteria	Proteobacteria	Gammaproteobacteria			uncultured gamma proteobacterium	-	-	-	-	-	-	-	-	-	-	-	-	-	-	-	-	-	-	-	-	-	-	-	-	-
New.ReferenceOTU141	0,0%	0,0%	0,0%	0,0%	0,0%	0,0%	0,0%	0,0%	0,0%	Bacteria	Armatimonadetes	Armatimonadia	Armatimonadales	Armatimonadaceae	uncultured bacterium	-	-	-	-	-	-	-	-	-	-	-	-	-	-	-	-	-	-	-	-	-	-	-	-	-
New.ReferenceOTU1249	0,0%	0,0%	0,0%	0,0%	0,0%	0,0%	0,0%	0,0%	0,0%	Bacteria	Proteobacteria	Alphaproteobacteria	Rickettsiales		Candidatus Gortzia	-	-	-	-	-	-	-	-	-	-	-	-	-	-	-	-	-	-	-	-	-	-	-	-	-
New.ReferenceOTU613	0,0%	0,0%	0,0%	0,0%	0,0%	0,0%	0,0%	0,0%	0,0%	Bacteria	Proteobacteria	Deltaproteobacteria	MIZ46		uncultured bacterium	-	-	-	-	-	-	-	-	-	-	-	-	-	-	-	-	-	-	-	-	-	-	-	-	-
New.ReferenceOTU611	0,0%	0,0%	0,0%	0,0%	0,0%	0,0%	0,0%	0,0%	0,0%	Bacteria	Proteobacteria	Betaproteobacteria	Burkholderiales	Comamonadaceae	Novosphingobium	-	napA	-	-	-	-	-	norB	-	nifH	nifD	-	ureA	-	-	-	-	-	mmoX	-	laccase_Asco	laccase_Basidio	-	-	ppo
New.ReferenceOTU1240	0,0%	0,0%	0,0%	0,0%	0,0%	0,0%	0,0%	0,0%	0,0%	Unassigned					Pirellula	-	napA	-	-	-	-	-	norB	-	-	-	-	-	-	-	-	-	-	-	-	-	-	-	-	-
New.ReferenceOTU1247	0,0%	0,0%	0,0%	0,0%	0,0%	0,0%	0,0%	0,0%	0,0%	Bacteria	Proteobacteria	Alphaproteobacteria	Rhodospirillales	Rhodospirillaceae	Elstera	-	-	-	-	-	-	-	-	-	-	-	-	-	-	-	-	-	-	-	-	-	-	-	-	-
New.ReferenceOTU1244	0,0%	0,0%	0,0%	0,0%	0,0%	0,0%	0,0%	0,0%	0,0%	Bacteria	Planctomycetes	Planctomycetia	Gemmatales	Gemmataceae	uncultured	-	-	-	-	-	-	-	-	-	-	-	-	-	-	-	-	-	-	-	-	-	-	-	lip	-
New.ReferenceOTU618	0,0%	0,0%	0,0%	0,0%	0,0%	0,0%	0,0%	0,0%	0,0%	Bacteria	Gemmatimonadetes	Gemmatimonadetes	Gemmatimonadales	A1-B1	Gemmatimonas	-	napA	-	-	-	-	nirK	norB	nosZ	-	-	nrfA	-	dsrA	dsrB	-	-	chb	-	-	-	-	-	-	-
New.ReferenceOTU1648	0,0%	0,0%	0,0%	0,0%	0,0%	0,0%	0,0%	0,0%	0,0%	Bacteria	Proteobacteria	Alphaproteobacteria	Rhodospirillales	Rhodospirillaceae	uncultured	-	-	-	-	-	-	-	-	-	-	-	-	-	-	-	-	-	-	-	-	-	-	-	lip	-
New.ReferenceOTU1647	0,0%	0,0%	0,0%	0,0%	0,0%	0,0%	0,0%	0,0%	0,0%	Bacteria	Proteobacteria	Deltaproteobacteria	Bdellovibrionales	Bdellovibrionaceae	Bdellovibrio	-	-	-	-	-	-	nirK	norB	-	-	-	nrfA	-	-	-	-	-	-	-	-	laccase_Asco	laccase_Basidio	-	-	ppo
New.ReferenceOTU1645	0,0%	0,0%	0,0%	0,0%	0,0%	0,0%	0,0%	0,0%	0,0%	Bacteria	Actinobacteria	Acidimicrobiia	Acidimicrobiales	C111	uncultured	-	-	-	-	-	-	-	-	-	-	-	-	-	-	-	-	-	-	-	-	-	-	-	lip	-
New.ReferenceOTU1641	0,0%	0,0%	0,0%	0,0%	0,0%	0,0%	0,0%	0,0%	0,0%	Unassigned					Bdellovibrio	-	-	-	-	-	-	nirK	norB	-	-	-	nrfA	-	-	-	-	-	-	-	-	laccase_Asco	laccase_Basidio	-	-	ppo
New.ReferenceOTU1427	0,0%	0,0%	0,0%	0,0%	0,0%	0,0%	0,0%	0,0%	0,0%	Bacteria	Proteobacteria	Alphaproteobacteria	BD7-3		uncultured alpha proteobacterium	-	-	-	-	-	-	-	-	-	-	-	-	-	-	-	-	-	-	-	-	-	-	-	-	-
New.ReferenceOTU1424	0,0%	0,0%	0,0%	0,0%	0,0%	0,0%	0,0%	0,0%	0,0%	Bacteria	Planctomycetes	vadinHA49	DH61		uncultured bacterium	-	-	-	-	-	-	-	-	-	-	-	-	-	-	-	-	-	-	-	-	-	-	-	-	-
New.ReferenceOTU1425	0,0%	0,0%	0,0%	0,0%	0,0%	0,0%	0,0%	0,0%	0,0%	Bacteria	Proteobacteria	Deltaproteobacteria	Myxococcales	Cystobacterineae	uncultured bacterium	-	-	-	-	-	-	-	-	-	-	-	-	-	-	-	-	-	-	-	-	-	-	-	-	-
New.ReferenceOTU1420	0,0%	0,0%	0,0%	0,0%	0,0%	0,0%	0,0%	0,0%	0,0%	Unassigned					uncultured eukaryote	-	-	-	-	-	-	-	-	-	-	-	-	-	-	-	-	-	-	-	-	-	-	-	-	-
New.ReferenceOTU1428	0,0%	0,0%	0,0%	0,0%	0,0%	0,0%	0,0%	0,0%	0,0%	Bacteria	Proteobacteria	Alphaproteobacteria	Rhodospirillales	Acetobacteraceae	Rhodovastum	-	-	-	-	-	-	-	-	-	-	-	-	-	-	-	-	-	-	-	-	-	-	-	-	-
New.ReferenceOTU1429	0,0%	0,0%	0,0%	0,0%	0,0%	0,0%	0,0%	0,0%	0,0%	Bacteria	Proteobacteria	Deltaproteobacteria	Myxococcales	Cystobacterineae	uncultured bacterium	-	-	-	-	-	-	-	-	-	-	-	-	-	-	-	-	-	-	-	-	-	-	-	-	-
New.ReferenceOTU721	0,0%	0,0%	0,0%	0,0%	0,0%	0,0%	0,0%	0,0%	0,0%	Bacteria	Verrucomicrobia	[Spartobacteria]	[Chthoniobacterales]	[Chthoniobacteraceae]	Chthoniobacter	-	napA	-	-	-	-	nirK	-	-	-	-	nrfA	ureA	-	-	-	-	-	-	-	-	-	-	-	-
New.ReferenceOTU720	0,0%	0,0%	0,0%	0,0%	0,0%	0,0%	0,0%	0,0%	0,0%	Bacteria	Proteobacteria	Deltaproteobacteria	Myxococcales		uncultured delta proteobacterium	-	-	-	-	-	-	-	-	-	-	-	-	-	-	-	-	-	-	-	-	-	-	-	-	-
New.ReferenceOTU727	0,0%	0,0%	0,0%	0,0%	0,0%	0,0%	0,0%	0,0%	0,0%	Bacteria	Proteobacteria	Gammaproteobacteria	Legionellales	Coxiellaceae	Coxiella	-	-	-	-	-	-	-	-	-	-	-	-	-	dsrA	dsrB	-	-	-	-	-	laccase_Asco	laccase_Basidio	-	-	ppo
New.ReferenceOTU726	0,0%	0,0%	0,0%	0,0%	0,0%	0,0%	0,0%	0,0%	0,0%	Bacteria	Proteobacteria	Deltaproteobacteria	Myxococcales		uncultured bacterium	-	-	-	-	-	-	-	-	-	-	-	-	-	-	-	-	-	-	-	-	-	-	-	-	-
New.ReferenceOTU725	0,0%	0,0%	0,0%	0,0%	0,0%	0,0%	0,0%	0,0%	0,0%	Bacteria	Proteobacteria	Deltaproteobacteria	Myxococcales	Haliangiaceae	Haliangium	-	-	-	-	-	-	-	-	-	-	-	-	ureA	-	-	-	-	chb	-	-	-	-	-	-	-
New.ReferenceOTU1332	0,0%	0,0%	0,0%	0,0%	0,0%	0,0%	0,0%	0,0%	0,0%	Bacteria	Proteobacteria	Alphaproteobacteria			uncultured	-	-	-	-	-	-	-	-	-	-	-	-	-	-	-	-	-	-	-	-	-	-	-	lip	-
New.ReferenceOTU1331	0,0%	0,0%	0,0%	0,0%	0,0%	0,0%	0,0%	0,0%	0,0%	Bacteria	Proteobacteria	Alphaproteobacteria	Rhodospirillales	Rhodospirillaceae	Skermanella	-	napA	-	-	-	-	-	-	-	nifH	nifD	-	ureA	-	-	-	-	-	mmoX	-	laccase_Asco	laccase_Basidio	-	-	ppo
New.ReferenceOTU1337	0,0%	0,0%	0,0%	0,0%	0,0%	0,0%	0,0%	0,0%	0,0%	Bacteria	Proteobacteria	Deltaproteobacteria	Myxococcales	Polyangiaceae	Byssovorax	-	-	-	-	-	-	-	-	-	-	-	-	-	-	-	-	-	-	-	-	-	-	-	-	-
New.ReferenceOTU1334	0,0%	0,0%	0,0%	0,0%	0,0%	0,0%	0,0%	0,0%	0,0%	Bacteria	Proteobacteria	Alphaproteobacteria	Rhodospirillales	Rhodospirillaceae	uncultured	-	-	-	-	-	-	-	-	-	-	-	-	-	-	-	-	-	-	-	-	-	-	-	lip	-
New.ReferenceOTU1335	0,0%	0,0%	0,0%	0,0%	0,0%	0,0%	0,0%	0,0%	0,0%	Bacteria	Chlamydiae	Chlamydiia	Chlamydiales		uncultured bacterium	-	-	-	-	-	-	-	-	-	-	-	-	-	-	-	-	-	-	-	-	-	-	-	-	-
New.ReferenceOTU1338	0,0%	0,0%	0,0%	0,0%	0,0%	0,0%	0,0%	0,0%	0,0%	Bacteria	Proteobacteria	Gammaproteobacteria	Alteromonadales	OM60	uncultured bacterium	-	-	-	-	-	-	-	-	-	-	-	-	-	-	-	-	-	-	-	-	-	-	-	-	-
New.ReferenceOTU1339	0,0%	0,0%	0,0%	0,0%	0,0%	0,0%	0,0%	0,0%	0,0%	Unassigned					Crenothrix	-	-	-	-	-	-	-	-	-	-	-	-	-	-	-	-	-	-	-	pmoA	-	-	-	-	-
New.ReferenceOTU129	0,0%	0,0%	0,0%	0,0%	0,0%	0,0%	0,0%	0,0%	0,0%	Bacteria	Proteobacteria	Gammaproteobacteria	Chromatiales		uncultured	-	-	-	-	-	-	-	-	-	-	-	-	-	-	-	-	-	-	-	-	-	-	-	lip	-
New.ReferenceOTU128	0,0%	0,0%	0,0%	0,0%	0,0%	0,0%	0,0%	0,0%	0,0%	Bacteria	Armatimonadetes	Armatimonadia	Armatimonadales	Armatimonadaceae	uncultured bacterium	-	-	-	-	-	-	-	-	-	-	-	-	-	-	-	-	-	-	-	-	-	-	-	-	-
New.ReferenceOTU439	0,0%	0,0%	0,0%	0,0%	0,0%	0,0%	0,0%	0,0%	0,0%	Bacteria	Planctomycetes	Planctomycetia	Gemmatales	Gemmataceae	uncultured	-	-	-	-	-	-	-	-	-	-	-	-	-	-	-	-	-	-	-	-	-	-	-	lip	-
New.ReferenceOTU438	0,0%	0,0%	0,0%	0,0%	0,0%	0,0%	0,0%	0,0%	0,0%	Bacteria	Proteobacteria	Betaproteobacteria	Burkholderiales	Comamonadaceae	Thauera	-	napA	narG	nirA	-	nirS	-	norB	nosZ	-	-	-	ureA	-	-	-	-	-	mmoX	-	-	-	-	-	-
New.ReferenceOTU437	0,0%	0,0%	0,0%	0,0%	0,0%	0,0%	0,0%	0,0%	0,0%	Bacteria	Proteobacteria	Alphaproteobacteria	Rhizobiales	Rhizobiaceae	Rhizobium	-	napA	narG	nirA	-	-	nirK	norB	nosZ	nifH	nifD	nrfA	ureA	-	-	-	-	chb	mmoX	-	laccase_Asco	laccase_Basidio	-	-	ppo
New.ReferenceOTU127	0,0%	0,0%	0,0%	0,0%	0,0%	0,0%	0,0%	0,0%	0,0%	Bacteria	Planctomycetes	Phycisphaerae	WD2101		uncultured bacterium	-	-	-	-	-	-	-	-	-	-	-	-	-	-	-	-	-	-	-	-	-	-	-	-	-
New.ReferenceOTU433	0,0%	0,0%	0,0%	0,0%	0,0%	0,0%	0,0%	0,0%	0,0%	Bacteria	Proteobacteria	Deltaproteobacteria	MIZ46		uncultured bacterium	-	-	-	-	-	-	-	-	-	-	-	-	-	-	-	-	-	-	-	-	-	-	-	-	-
New.ReferenceOTU120	0,0%	0,0%	0,0%	0,0%	0,0%	0,0%	0,0%	0,0%	0,0%	Bacteria	Proteobacteria	Alphaproteobacteria	Sphingomonadales	Sphingomonadaceae	Sandarakinorhabdus	-	-	-	-	-	-	-	-	-	-	-	-	-	-	-	-	-	-	-	-	-	-	-	-	-
New.ReferenceOTU123	0,0%	0,0%	0,0%	0,0%	0,0%	0,0%	0,0%	0,0%	0,0%	Bacteria	Proteobacteria	Alphaproteobacteria	Rhodospirillales	Rhodospirillaceae	Defluviicoccus	-	-	-	-	-	-	-	-	-	-	-	-	-	-	-	-	-	-	-	-	-	-	-	-	-
New.ReferenceOTU125	0,0%	0,0%	0,0%	0,0%	0,0%	0,0%	0,0%	0,0%	0,0%	Bacteria	Planctomycetes	Planctomycetia	Pirellulales	Pirellulaceae	Rhodopirellula	-	napA	-	nirA	-	-	-	norB	-	-	-	nrfA	-	-	-	-	-	-	-	-	-	laccase_Basidio	-	-	-
New.ReferenceOTU436	0,0%	0,0%	0,0%	0,0%	0,0%	0,0%	0,0%	0,0%	0,0%	Bacteria	Proteobacteria	Alphaproteobacteria	Rhizobiales	Rhizobiaceae	Rhizobium	-	napA	narG	nirA	-	-	nirK	norB	nosZ	nifH	nifD	nrfA	ureA	-	-	-	-	chb	mmoX	-	laccase_Asco	laccase_Basidio	-	-	ppo
New.ReferenceOTU126	0,0%	0,0%	0,0%	0,0%	0,0%	0,0%	0,0%	0,0%	0,0%	Unassigned					Solanum tuberosum (potato)	-	-	-	-	-	-	-	-	-	-	-	-	-	-	-	-	-	-	-	-	-	-	-	-	-
New.ReferenceOTU121	0,0%	0,0%	0,0%	0,0%	0,0%	0,0%	0,0%	0,0%	0,0%	Unassigned					uncultured bacterium	-	-	-	-	-	-	-	-	-	-	-	-	-	-	-	-	-	-	-	-	-	-	-	-	-
New.ReferenceOTU430	0,0%	0,0%	0,0%	0,0%	0,0%	0,0%	0,0%	0,0%	0,0%	Unassigned					uncultured bacterium	-	-	-	-	-	-	-	-	-	-	-	-	-	-	-	-	-	-	-	-	-	-	-	-	-
New.ReferenceOTU543	0,0%	0,0%	0,0%	0,0%	0,0%	0,0%	0,0%	0,0%	0,0%	Bacteria	Proteobacteria	Deltaproteobacteria	Bdellovibrionales	Bdellovibrionaceae	Bdellovibrio	-	-	-	-	-	-	nirK	norB	-	-	-	nrfA	-	-	-	-	-	-	-	-	laccase_Asco	laccase_Basidio	-	-	ppo
New.ReferenceOTU542	0,0%	0,0%	0,0%	0,0%	0,0%	0,0%	0,0%	0,0%	0,0%	Bacteria	Proteobacteria	Deltaproteobacteria	Bdellovibrionales	Bdellovibrionaceae	Bdellovibrio	-	-	-	-	-	-	nirK	norB	-	-	-	nrfA	-	-	-	-	-	-	-	-	laccase_Asco	laccase_Basidio	-	-	ppo
