# Supplementary material for: Hunters or farmers? Microbiome characteristics help elucidate the diet composition in an aquatic carnivorous plant
Source: Microbiome. 2018 Dec 17;6:225. doi: 10.1186/s40168-018-0600-7 (PMC6297986; doi:10.1186/s40168-018-0600-7)
Supplement: Supplementary file 6 — Table S9. Results of the adonis analysis for U. australis and U.vulgaris prokaryotic community based on 16S rDNA gene sequencing. (PDF 459 kb) [file 40168_2018_600_MOESM6_ESM.pdf]

Table S9: Results of the adonis analysis for *U. australis* and *U.vulgaris* prokaryotic community based on 16S rDNA gene sequencing

**Group significance (adonis)**	**Df**	**SumsOfSqs**	**MeanSqs**	**F.Model**	**R^2**	**Pr(>F)**	**Signif.**				
Plant (U.vulgaris, U.australis)	1	0.77165	0.77165	15.48	0.38241	0.001	***				
Residuals	*25*	*1.24622*	*0.04985*	*0.61759*							
Total	*26*	*2.01787*	*1.00000*								
Age (young, medium, old)	2	0.09585	0.047924	0.59842	0.0475	0.825					
Residuals	*24*	*1.92202*	*0.080084*	*0.9525*							
Total	*26*	*2.01787*	*1.0000*								
Treatment (trap,periphyton)	1	0.07595	0.075945	0.9777	0.03764	0.341					
Residuals	*25*	*1.94192*	*0.077677*	*0.96236*							
Total	*26*	*2.01787*	*1.00000*								
codes:	0	‘***’	0.001	‘**’	0.01	‘*’	0.05	‘.’	0.1	‘ ’	1
